# Macroecology of Australian Tall Eucalypt Forests: Baseline Data from a Continental-Scale Permanent Plot Network

**DOI:** 10.1371/journal.pone.0137811

**Published:** 2015-09-14

**Authors:** Sam W. Wood, Lynda D. Prior, Helen C. Stephens, David M. J. S. Bowman

**Affiliations:** 1 School of Biological Sciences, University of Tasmania, Hobart, Tasmania, Australia; 2 Terrestrial Ecosystem Research Network, Brisbane, Queensland, Australia; University of New South Wales, AUSTRALIA

## Abstract

Tracking the response of forest ecosystems to climate change demands large (≥1 ha) monitoring plots that are repeatedly measured over long time frames and arranged across macro-ecological gradients. Continental scale networks of permanent forest plots have identified links between climate and carbon fluxes by monitoring trends in tree growth, mortality and recruitment. The relationship between tree growth and climate in Australia has been recently articulated through analysis of data from smaller forest plots, but conclusions were limited by (a) absence of data on recruitment and mortality, (b) exclusion of non-eucalypt species, and (c) lack of knowledge of stand age or disturbance histories. To remedy these gaps we established the Ausplots Forest Monitoring Network: a continental scale network of 48 1 ha permanent plots in highly productive tall eucalypt forests in the mature growth stage. These plots are distributed across cool temperate, Mediterranean, subtropical and tropical climates (mean annual precipitation 850 to 1900 mm per year; mean annual temperature 6 to 21°C). Aboveground carbon stocks (AGC) in these forests are dominated by eucalypts (90% of AGC) whilst non-eucalypts in the understorey dominated species diversity and tree abundance (84% of species; 60% of stems). Aboveground carbon stocks were negatively related to mean annual temperature, with forests at the warm end of the temperature range storing approximately half the amount of carbon as forests at the cool end of the temperature range. This may reflect thermal constraints on tree growth detected through other plot networks and physiological studies. Through common protocols and careful sampling design, the Ausplots Forest Monitoring Network will facilitate the integration of tall eucalypt forests into established global forest monitoring initiatives. In the context of projections of rapidly warming and drying climates in Australia, this plot network will enable detection of links between climate and growth, mortality and carbon dynamics of eucalypt forests.

## Introduction

Forests play a central role in the global carbon cycle and understanding how carbon stored in forest ecosystems responds to changes in climate, land use and atmospheric composition is a key challenge confronting land managers, scientists and policy makers [1,2,3]. Globally, significant changes in carbon stocks have been documented for forests spanning the boreal, temperate and tropical regions through the examination of changes in tree growth, recruitment and mortality in permanent monitoring plots distributed across macro-ecological gradients [4,5,6,7,8]. For example, forests measured by large (1 ha) monitoring plots in African (AfriTRON; [6]) and Amazonian tropical forests (RAINFOR; [9]) exhibited an increase in biomass over recent decades, whereby biomass increment through tree growth and recruitment exceeded biomass losses through mortality. These pan-tropical trends were linked to variation in resource availability, with an increase in atmospheric CO_2_ a plausible driver [9].

There are some indications, however, that the forest carbon sink in these systems may be weakening. Recent drought events in the Amazon drastically increased mortality rates and may have caused a switch of the Amazon rainforest from a net carbon sink to a carbon source [10,11]. Indeed, pervasive increases in tree mortality are becoming increasingly recognised as consequence of a changing climate worldwide [12]. Forest monitoring plots have revealed links between increases in tree mortality rates and widespread drought or temperature stress in the Western United States [13,14] and Canada’s boreal forests [15]. Worryingly, these widespread climate-triggered mortality events may alter the structure, function and composition of ecological communities in ways that are distinct from other natural forest disturbances [16], and have the potential to trigger transitions to non-forest ecosystems, especially when coupled with wildfire [17].

Until recently, Australian eucalypt forest ecosystems have been conspicuously absent from global analyses of changes in forest productivity and biomass (e.g. [2,4]). This represents a clear knowledge gap for one of the key global forest systems given that eucalypt forests dominate (75%) the vast forest estate of the Australian continent (124 million ha; [18]). Prior et al. [19] recently compiled a continental dataset of 2409 forestry inventory plots (average 0.15 ha) in the Australian production forest estate (i.e. forests that are primarily managed for timber harvesting), enabling examination of tree growth patterns in relation to macro-climatic gradients and stand structural attributes [20,21,22]. They showed that tree growth is constrained by a warmer and drier climate, particularly for large trees, and that projected warmer temperatures will probably reduce rates of productivity and carbon sequestration in Australian eucalypt forests [20,21]. Based on these findings, and the narrow thermal range of most eucalypt species [23,24], Bowman et al. [20] also predicted that projected long term changes in climate will lead to widespread declines in the abundance of many eucalypt forest species. Unfortunately, incorporating stand-level recruitment and mortality processes into their analysis was not possible due to (a) small plot sizes; (b), methodological inconsistencies amongst state forest agencies that collected the data; and (c) insufficient metadata relating to recruitment and mortality within plots [19]. Furthermore, this dataset did not consistently include non-commercial tree species, thus largely excluding key elements of the diverse non-eucalypt understorey. Therefore, our understanding of the effect of a changing climate on carbon storage and the structure and composition of eucalypt forests remains incomplete.

Here we describe the establishment of the Ausplots Forest Monitoring Network: a long-term forest monitoring plot network that aims to examine the effect of current and future climate on the dynamics (growth, mortality and recruitment) and carbon fluxes of the tall eucalypt forests of Australia. The aim of this paper is to: (1) outline the sampling design and methodological approach of the plot network; (2) present baseline data describing the climatic profile, stand structure, floristic composition and aboveground carbon stocks of 48 forest plots;(3) contextualise the plot network within the wider eucalypt forest ecosystem; and (4) demonstrate the power of the network for macro-ecological studies by analysing the relationship between three climatic variables and aboveground carbon storage in tall eucalypt forests across the Australian continent. Long term monitoring projects must be underpinned by clear and coherent background information and thorough, accessible documentation [25,26, 27,28,29]. Thus, an underlying intention of this paper is to provide open access reference material to inform and encourage the utilisation of the plot network now and into the future.

## Methods

### Sampling Design

The plot network focusses on the tall eucalypt forests—the subset of the Australian forest estate that includes eucalypt dominated forests >30 m tall [18]. Tall eucalypt forests occupy around 4% of the Australian forest estate [18] and are a useful case study for macro-ecological studies because they are widely distributed in a discontinuous arc across southern and eastern Australia, including cool temperate, Mediterranean, subtropical and humid tropical climates [30]. Tall eucalypt forests are renowned for their diversity [31], gigantic size (up to 70 m tall; [32,33]) and high carbon density [34]. These forests are central to native timber production [18] and provide important ecosystem services such as water catchments [35]. Preliminary analyses by Wood et al. [36] indicated that the predicted reductions in productivity for the wider eucalypt forest estate [20] were especially relevant to the tall eucalypt forest ecosystem and that this would have consequences for this globally significant and economically valuable forest ecosystem.

The overall aim of the Ausplots Forest Monitoring Network is to investigate the effects of a continental-scale climatic gradient on the growth and dynamics of tall eucalypt forests. To achieve this, we sought to minimise the effects of variation in forest type, growth stage and site quality on forest growth and dynamics, whilst sampling plots across a wide climatic range. We targeted forest stands that were: (1) arranged within regions that span a large macroclimatic gradient; (2) dominated by archetypal tall forest species belonging to the *Eucalyptus* genus (*Eucalyptus regnans*, *E*. *obliqua*, *E*. *delegatensis*, *E*. *diversicolor*, *E*. *jacksonii*, *E*. *pilularis*, *E*. *fastigata*, *E*. *grandis*); (3) dominated by a cohort of mature trees regenerated following a disturbance event approximately 75–160 years ago; (4) on highly productive sites with canopy dominants >45 m height. We also prioritised forest stands on secure tenure (i.e. National Parks, Forest Reserves) with minimal harvesting history and—where possible—existing permanent inventory plot infrastructure or a history of forest ecology research.

We sought to hold growth stage constant by targeting stands that were dominated by a cohort of trees of similar age. Stands in different growth stages (i.e. juvenile, sapling, pole, mature, old growth) are likely to have markedly different growth and turnover rates, which would confound our investigation of the effects of the macroclimatic gradient. We therefore explicitly targeted stands dominated by trees in the mature growth stage, defined here as trees >70 years old that have reached (or are approaching) maximum height with crowns that have reached (or are approaching) full lateral development. Stands dominated by young regrowth trees exhibiting strong apical dominance or very large trees with signs of senescence such as hollows, burls, distorted branches and broken tops were not considered. The ecology of the tall eucalypt species targeted by the Ausplots Forest Monitoring Network is tightly coupled to high or medium intensity fire [30,36]. Such fires tend to be strongly dominated by single or multi-aged cohorts of trees [37,38,39,40,41]. Where possible, we targeted stands dominated by at least one cohort of tall eucalypt trees that regenerated after known fire events between 1852 and 1939 (e.g. 1850’s, 1917 and 1937 in Western Australia; 1898 and 1934 in Tasmania; 1898 and 1939 Victoria). In regions where stand replacing fire events are relatively rare and/or poorly documented (Far North Queensland, Northern and Southern NSW) we targeted stands that were dominated by at least one cohort of trees in the mature growth stage that were structurally analogous to stands of known age in Western Australia, Tasmania and Victoria.

### Sampling Protocols

The Ausplots Forest Monitoring Network uses consistent and repeatable sampling protocols that enable robust comparisons of forest dynamics across Australia and integration into other established forest plot networks around the world. We based tree measurements and coding protocols on the RAINFOR, AfriTRON and GEM plot methodologies [27,6,42,43]. These protocols have many overlaps with those used in monitoring plots maintained by Australian State Forest management agencies, allowing direct comparisons with existing Australian forest inventory data such as the Permanent Growth Plot Network dataset compiled by Prior et al. [19].

The field protocols are outlined in detail in the Ausplots Forest Monitoring Network Survey Protocols Manual (S1 Text) and are summarised briefly here. All plots are 100 m x 100 m (1 ha)–divided into twenty five 20x20m subplots—with the corners demarcated with steel posts. Geospatial information was acquired using a GPS and ancillary information about the landform, geology and disturbance history was recorded. Within the 1 ha plot all live stems ≥ 10cm diameter are: (a) identified to species level; (b) measured for diameter at 1.3m (diameter at breast height, DBH), unless buttressed or deformed, where strict rules determine an alternative point of measurement; (c) tagged with a permanent, unique identifier; (d) assigned a stem form (e.g. broken top, double leader, etc.); (e) assigned a position in the canopy (e.g. suppressed, dominant, co-dominant, emergent) and a growth stage (e.g. regrowth, regenerating, mature, senescent); and (f) attributed a X and Y co-ordinate between 0 and 100 in relation to a georeferenced corner of the plot (0,0). Dead trees are measured as above and assigned with codes describing the physical mechanism for mortality according to RAINFOR coding [27,42]. Tree height is measured for a subset of overstorey and understorey trees across the observed diameter range using a rangefinding digital hypsometer. Hemispherical photographs of the canopy are collected at sixteen internal fixed posts at intervals of 20 m within the plot. All field measurements were conducted under scientific research permits (S4 Text) on reserved land tenures with some degree of protection. Protected flora species were not sampled.

The initial set of plots were measured between 2012 and 2015. Plots will be remeasured on a 3–5 year cycle, although impromptu censuses may be made following extreme weather events or fire.

### Data Analyses

To demonstrate that our plot network spans the climatic range of the tall eucalypt forest ecosystem we examined the distribution of our plots within the climate envelope of the tall eucalypt forest estate. The WORLDCLIM climate dataset [44] was used to obtain mean annual temperature (MAT) and mean annual precipitation (MAP) data for each plot in the Ausplots Forest Monitoring Network, and to define the climate envelope for the mapped extent of tall eucalypt forests [18] (MPIG 2013). Pan evaporation was also derived for each plot in the Ausplots Forest Monitoring Network from ANUCLIM 6.1 [45] from which the ratio of precipitation and evaporation was calculated (P:E; an index of water availability; [21]). We also contextualised the plot network in relation to the agro-climatic bioregions of Hutchinson et al. [46]. These agro-climatic bioregions reflect major patterns in plant growth, temperature and moisture indices and seasonality across Australia (see S3 Table).

We contextualised the stand structure of the Ausplots Forest Monitoring Network within the wider eucalypt forest estate by comparing basal area and diameter-height curves of the Ausplots Forest Monitoring Network in relation to the Permanent Growth Plot (PGP) Network of Prior et al. [19]. We first partitioned the PGP Network into height classes (25–35 m, 35–45 m, >45 m) based on a predictive model relating tree height to climatic data (see S3 Text). We then calculated average basal area for each of the predicted height classes for both plot networks. We generated diameter-height curves for the two plot networks using a linear model of the form *height ~ log(DBH)*. Tree height is an effective integrator of the key determinants of tree growth [47] and we used the relationship between diameter and height as a proxy for site productivity: trees in highly productive forests will be taller for a given diameter compared to trees in low productivity forests.

To describe the floristics of the Ausplots Forest Monitoring Network we calculated the Importance Value of each species [48,49]. The importance value ranks the relative dominance of species in a forest community based on their relative frequency, relative density and relative size [48]. Relative frequency is the percentage of plots occupied by a species. Relative density is the number of individuals per area as a percent of the number of individuals of all species. Relative size is the total basal area of a species as a percent of the total basal area of all species. Each of these values is expressed as a percent ranging from 0 to 100. The Importance Value is the sum of these three measures, and can range from 0 to 300. We ranked the most important species for each region in the plot network. We also calculated the importance of three species guilds commonly recognised in tall eucalypt forests: (1) *Eucalyptus* species; (2) wet sclerophyll species and (3) rainforest species. Species were classified into these guilds (S1 Table) based on the literature (e.g. [50,51,52,30]) and online resources [53].

We used sixteen hemispherical canopy photographs to calculate baseline canopy cover for each plot. We calculated percent canopy cover for each photograph using the ECBTOOLS package in the R statistical program [54]. This program partitions each photo into canopy and sky based on the contrast between the colour and light intensity of each pixel. We used a cut-off value of 125 to delineate canopy from sky.

We calculated aboveground live biomass carbon (tC ha^-1^) for each plot to provide baseline carbon density estimates for the Ausplots Forest Monitoring Network. Because there are insufficient high-quality species-specific allometric equations to calculate the biomass (and subsequently the mass of carbon) for each tree species, we used generic allometric equations developed by Keith et al. [55]. The general equation for eucalypts averages a range of equations developed for common eucalypt species growing in native forests [55], including those targeted by the Ausplots Forest Monitoring Network (*E*. *obliqua*, *E*. *delegatensis*, *E*. *pilularis*, *E*. *regnans*). The general equations for rainforest combine equations for world-wide tropical forests (wet, moist and dry tropical) and site-specific equations from Australia (temperate and subtropical). The biomass of all eucalypt and non-eucalypt trees was calculated from diameter measurements using the general eucalypt and rainforest equations, respectively [55]. The individual biomass of eucalypt and non-eucalypt trees was summed to estimate plot level live aboveground biomass (AGB; t ha^-1^) and multiplied by 0.50 to convert to live aboveground carbon density (AGC: tC ha^-1^). Biomass and AGC were not presented for two plots in Western Australia (WAFWAR005 and WAFWAR008) that were dominated by *E*. *jacksonii* trees with pronounced buttressing. In these plots, over-inflated diameter measurements on the buttressed trees resulted in conspicuously large biomass estimates when using diameter-based allometric equations.

To explore the effect of climate on carbon storage in tall eucalypt forests we investigated the relationships between AGC and three climatic variables: MAT, MAP and P:E. We used linear models to test these relationships using model selection based on the Akaike Information Criterion, AIC (the lower the AIC value the better the model), which balances model fit and parsimony [56]. The R statistical program was used for all analyses.

## Results

### Climate

**Fig 1:**
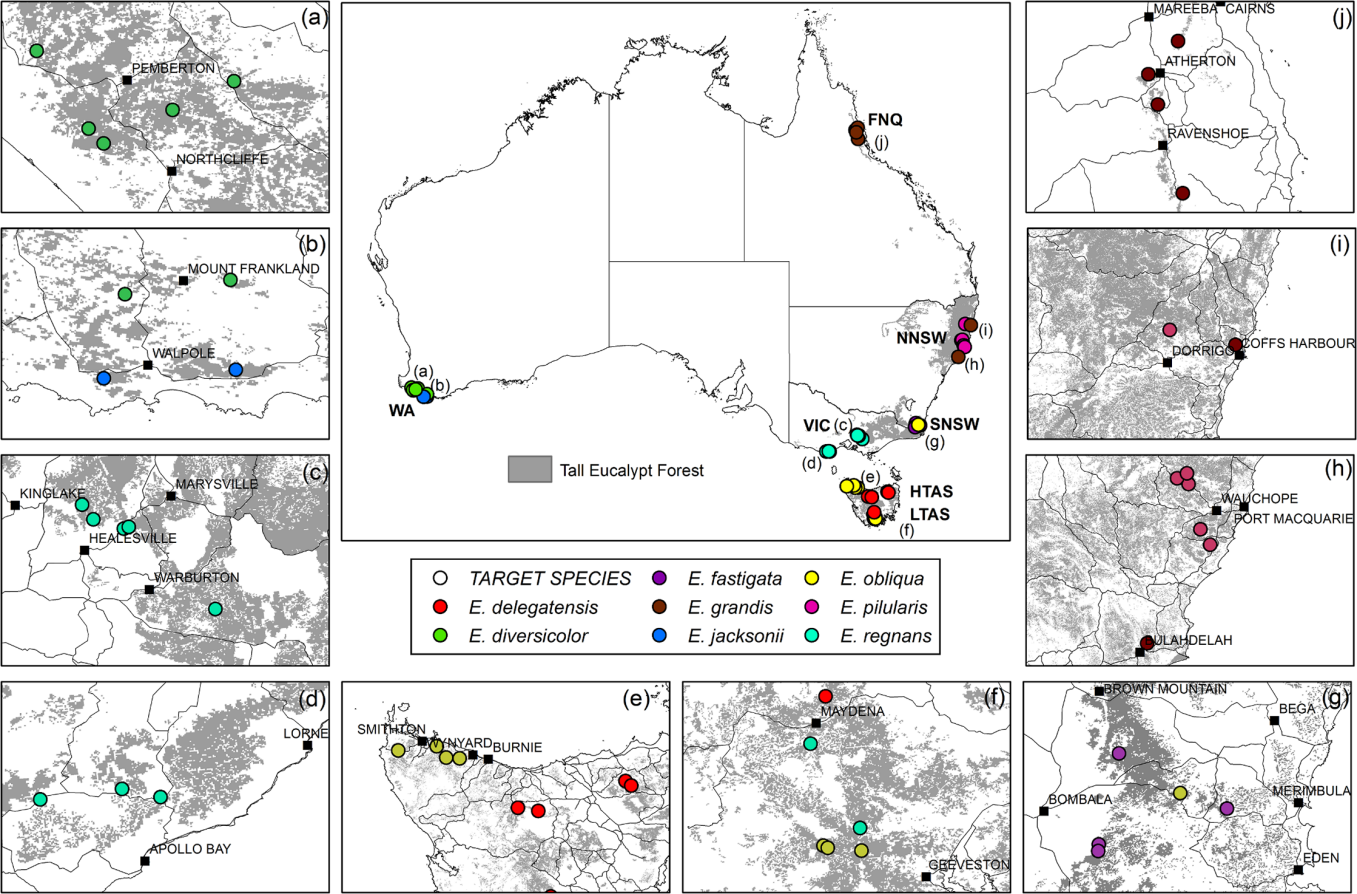
Continental (centre) and regional (a-j) distribution of 48 plots in the Ausplots Forest Monitoring Network. Grey shading indicates mapped tall eucalypt forests (>30 m MPIG 2014). Anticlockwise from left: (a,b) Western Australia (WA), *Eucalyptus diversicolor*, *E*. *jacksonii*; (c,d) Victoria (VIC), *E*. *regnans*; (e,f) Low and high elevation Tasmania (LTAS,HTAS), *E*. *obliqua*, *E*. *regnans*, *E*. *delegatensis*; (g) Southern NSW (SNSW), *E*. *fastigata*, *E*. *obliqua*; (h,i) Northern NSW (NSW), *E*. *pilularis*, *E*. *grandis*; (j) Far North Queensland (FNQ), *E*. *grandis*.

**Fig 2:**
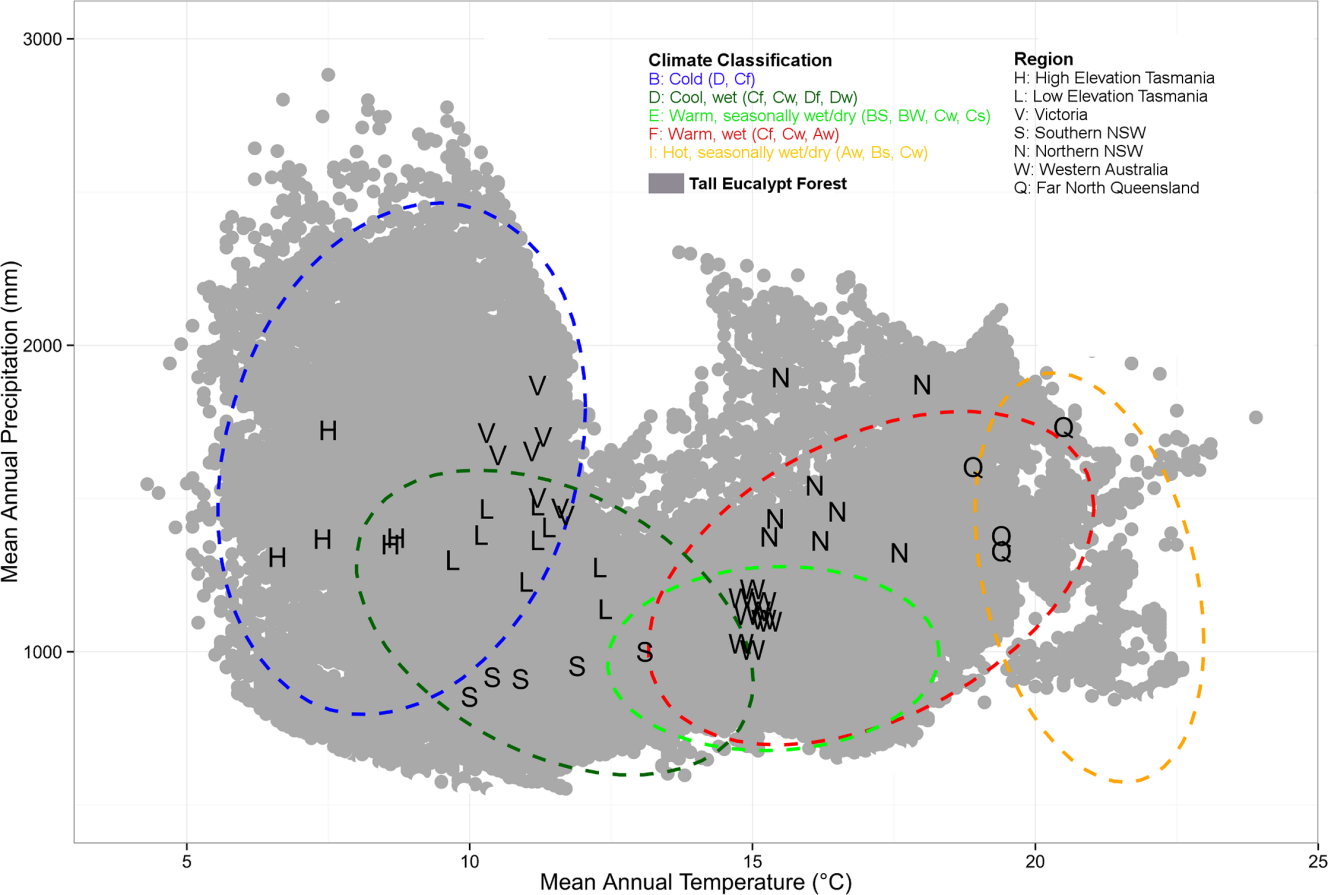
Distribution of 48 plots in the Ausplots Forest Monitoring Network within the climate envelope of tall eucalypt forests. Tall eucalypt forests are defined as eucalypt dominated forests with trees >30 m tall (MPIG 2014) and are shown geographically in Fig 1. Plots are represented as symbols that coincide with seven regions with distinct species and/or climatic attributes (see Table 1). Coloured ellipses represent the climate envelope of Hutchinson et al. [46] agro-climatic classes (see S3 Table).

The Ausplots Forest Monitoring Network consists of 48 1 ha plots within seven regions distributed across Australia (Fig 1; Table 1; S2 Text and S1 File; S1 and S2 Tables). The plot network spans the climatic space of the very tall (> 45 m) eucalypt forest estate (Fig 2), with a MAT range of 6.6 to 20.5°C and a MAP range of 853 to 1895 mm. The plot network includes tropical, sub-tropical, temperate and Mediterranean climates and spans five agro-climatic bioregions: (1) cold, (2) cool/wet, (3) warm, seasonally dry, (4) warm/wet and (5) hot/seasonally dry.

**Table 1:** Details and environmental parameters of the Ausplots Forest Monitoring Network summarised by seven regions which represent distinct target eucalypt species and/or climatic groupings. The means (and range) of climatic variables and elevation are presented for each region. MAT = Mean Annual Temperature. MAP = Mean Annual Precipitation. Plot-level details are presented in S2 Text, S1 File and S1 Table.

Region	No. Plots	Census Date	Target Species	Growth Stage^	Stand Establishment Date	IBRA Bioregion^+^	Climate Bioregion^$^	MAT	MAP	Elevation
								(°C)	(mm)	(m)
Northern NSW (NNSW)	8	2013	*E*. *pilularis*, *E*.*grandis*	Mature	Unknown	NNC	Warm, wet	16.3 *(15*.*3–18)*	1533 (1323–1895)	418 *(75–683)*
Southern NSW (SNSW)	5	2014	*E*. *fastigata*, *E*. *obliqua*	Mature	Unknown	SEC	Cool, wet	11.3 *(10–13*.*1)*	927 (853–1000)	739 *(420–955)*
Victoria (Vic)	8	2014	*E*.*regnans*	Mature	1898, 1939	SEH	Cool, wet	11.1 *(10*.*3–11*.*7)*	1624 (1445–1869)	578 *(337–863)*
Far North Queensland (FNQ)	4	2014	*E*. *grandis*	Mature	Unknown	WET	Hot, seasonal	19.6 *(18*.*9–20*.*5)*	1609 *(1376–1732)*	1012 *(795–1148)*
Western Australia (WA)	9	2012	*E*.*diversicolor*, *E*. *jacksonii*	Mature + OG	1852–1857,1917,1937	WAR	Warm, seasonal	14.8 *(14*.*8–15*.*3)*	1114 (1006–1204)	153 *(93–239)*
Low Elevation Tasmania (LTAS)	9	2012–2014	*E*. *obliqua*, *E*. *regnans*	Mature	1898,1920,1934	KIN,TNS,TSR	Cool, wet	11.1 *(9*.*7–12*.*4)*	1337 (1139–1477)	201 *(49–560)*
High Elevation Tasmania (HTAS)	5	2015	*E*. *delegatensis*	Mature	Unknown	BEL,TNS,TSR	Cold	7.8 *(6*.*6–8*.*7)*	1424 (1309–1723)	797 *(691–910)*
Ausplots Network	48	2012–2015	*-*	-	-		-	13.0 *(6*.*6–20*.*5)*	1364 *(853–1895)*	476 *(49–1148)*

$ Hutchinson Agro-Climate Classifications are presented in S3 Table

***+*** *IBRA Interim Biogeographic Regionalisation for Australia*: *BEL = Ben Lomond; KIN = King; NNC = NSW North Coast; SEC = South East Corner; SEH = South East Highlands; TNS = Tasmanian North Slopes; TSR = Tasmanian Southern Ranges; WAR = Warren; WET = Wet Tropics*.

***^*** *Growth stage of the dominant cohort of trees was classified as Regrowth*, *Regenerating*, *Mature or Old Growth (OG)–see text*.

### Stand Structure

The Ausplots Forest Monitoring Network currently includes 20931 individually tagged stems, censused between 2012 and 2015. The forest stands included in the network are characterised by the presence of at least one cohort of very tall trees with the mean height of dominant and co-dominant canopy trees across the plot network being 50 m (Table 2). Most stands had emergent eucalypt trees that extended above the forest canopy with over 50% of plots having at least one tree >60 m and 30% of plots having at least one tree >70 m (Table 2; S2 Table). The tallest trees were found in the *E*. *regnans* forests of Victoria (72–89 m) and the shortest trees were found in the *E*. *grandis* forests of Far North Queensland (40–56 m; Table 2). Non-eucalypt trees formed a distinct understorey layer with maximum heights shorter than the eucalypt canopy for all 48 plots (Table 2). Canopy cover ranged from 48% to 84%, with the highest values recorded in Victorian and Tasmanian forests (Table 2).

**Table 2:** Stand structural attributes of the Ausplots Forest Monitoring Network, summarised by seven regions which represent distinct target eucalypt species and/or climatic groupings. The means (and range) are presented for each region for eucalypt (including *Eucalyptus* spp. *and Corymbia* spp.) and non-eucalypt species. Plot-level details are presented in S2 Text and S2 Table.

Region	Target Species	Canopy Cover	Height Dominants	Maximum Height		Stocking density^&^		Basal Area^#^		Live Aboveground Carbon^#^ ^@^	
		(%)	(m)	(m)		(stems ha^-1^)		(m^2^ ha^-1^)		(tC ha^-1^)	
		All Trees	Eucalypt	Eucalypt	Non-Eucalypt	Eucalypt	Non-Eucalypt	Eucalypt	Non-Eucalypt	Eucalypt	Non-Eucalypt
Northern NSW (NNSW)	*E*. *pilularis*, *E*.*grandis*	70 *(48–80)*	53 *(45–59)*	70 *(57–77)*	31 *(19–44)*	187 *(86–336)*	351 *(227–627)*	48 *(38–68)*	9 *(5–17)*	260 *(169–399)*	30 *(15–58)*
Southern NSW (SNSW)	*E*. *fastigata*, *E*. *obliqua*	63 *(56–71)*	45 *(40–50)*	56 *(49–64)*	12 *(8–21)*	215 *(121–318)*	12 *(0–40)*	60 *(50–64)*	0.3 *(0*.*1–0*.*8)*	324 *(279–354)*	1 *(0*.*1–3)*
Victoria (Vic)	*E*.*regnans*	77 *(68–84)*	66 *(60–74)*	82 *(72–89)*	36 *(22–52)*	160 *(80–246)*	266 *(6–573)*	56 *(49–66)*	9 *(0*.*1–22)*	289 *(232–351)*	30 *(0–80)*
Far North Queensland (FNQ)	*E*. *grandis*	66 *(55–78)*	36 *(28–41)*	48 *(40–56)*	28 *(17–46)*	130 (100–199)	150 *(42–280)*	24 *(17–32)*	6 *(1–15)*	117 *(77–170)*	21 *(2–53)*
Western Australia (WA)	*E*.*diversicolor*, *E*. *jacksonii*	69 *(62–82)*	65 *(40–65)*	64 *(53–77)*	20 *(15–28)*	116 *(47–226)*	71 *(26–218)*	46 *(28–61)* *	5 *(0*.*3–14)*	270 *(155–380)* *	11 *(1–43)*
Low Elevation Tasmania (LTAS)	*E*. *obliqua*, *E*. *regnans*	78 *(71–84)*	48 *(39–54)*	61 *(57–66)*	36 *(24–49)*	158 *(60–310)*	394 *(170–787)*	51 *(24–70)*	15 *(8–22)*	294 *(113–400)*	50 *(25–82)*
High Elevation Tasmania (HTAS)	*E*. *delegatensis*	73 *(72–74)*	40 *(33–43)*	48 *(40–52)*	29 *(23–37)*	290 *(184–381)*	519 *(87–1561)*	61 *(48–85)*	14 *(2–33)*	310 *(232–426)*	44 *(6–90)*
Ausplots Network	*-*	71 *(48–84)*	50 *(28–74)*	63 *(40–89)*	28 *(8–52)*	172 *(47–381)*	257 *(0–1561)*	50 *(17–85)*	9 *(0*.*1–32)*	274 *(77–426)*	30 *(0–90)*

* Aboveground carbon (AGC) of two plots in Western Australia (WAFWAR005 and WAFWAR008) was not included because prominent buttressing of Eucalyptus jacksonii led to severe overestimates of AGC derived from diameter measurements.

*# Calculated using diameter measurements at 1*.*3m*, *with the exception of (a) trees measured above a prominent buttress or (b) deformed trees at 1*.*3m*.

*@ Live tree biomass (t ha* ^*-1*^ *) was calculated using generic allometric equations for eucalypt and non-eucalypt species developed by Keith et al. [55]–see text.*

*& Includes all stems associated with multi-stemmed trees*, *thus overestimates the number of individual trees in the plot*.

**Fig 3:**
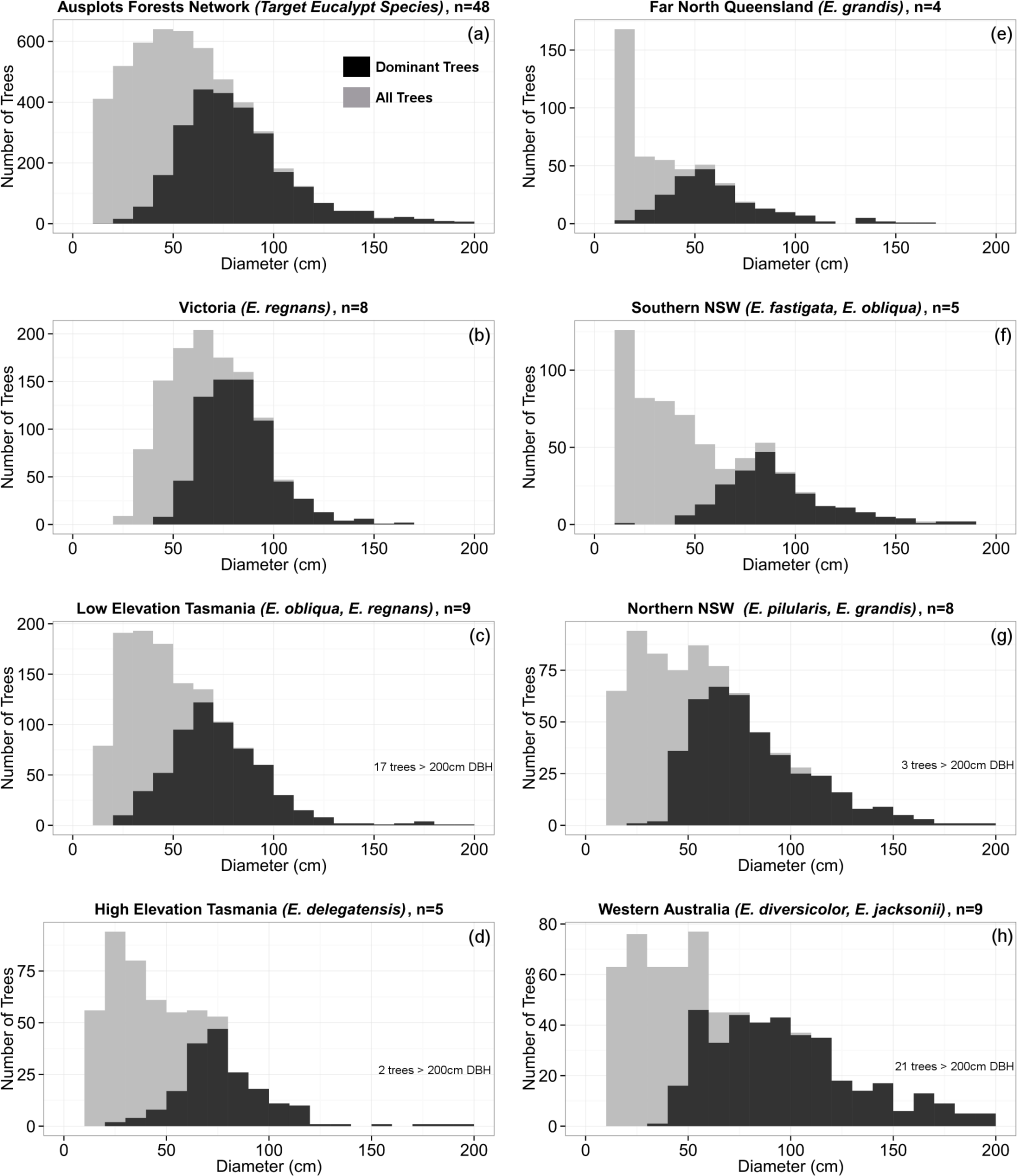
Diameter distribution of target tall eucalypt species in the Ausplots Forest Monitoring Network and for each region. Trees are grouped into 10 cm diameter bins. Stand structures are shown for all trees (grey bars) and for a subset of trees classified into ‘dominant’ or ‘co-dominant’ canopy position in the field (black bars). The number of plots and target eucalypt species for each region are indicated.

In each region the dominant and co-dominant canopy trees consistently formed distinctive cohorts of mature trees with DBH of 50–100 cm (Fig 3A–3H; black bars). For 23 plots in Victoria, Low Elevation Tasmania and Western Australia, these cohorts can be attributed to regeneration following stand replacing fires between the 1850’s and 1939 (Table 1; S1 Table). Very large diameter trees >150 cm DBH (Fig 3) were uncommon in the Ausplots Forest Monitoring Network (n = 138, 0.7% of all trees; 1.8% of eucalypts; Fig 3). Non-dominant *E*. *regnans* and *E*. *obliqua* trees currently growing below the canopy formed peaked distributions in Victoria and Tasmania (Fig 3B–3D; grey bars), consistent with pulsed recruitment, whereas non-dominant trees in Western Australia, Far North Queensland, Southern NSW and Northern NSW had a left-skewed distribution (Fig 3E–3H; grey bars), suggesting prolific recent recruitment.

### Floristics

**Fig 4:**
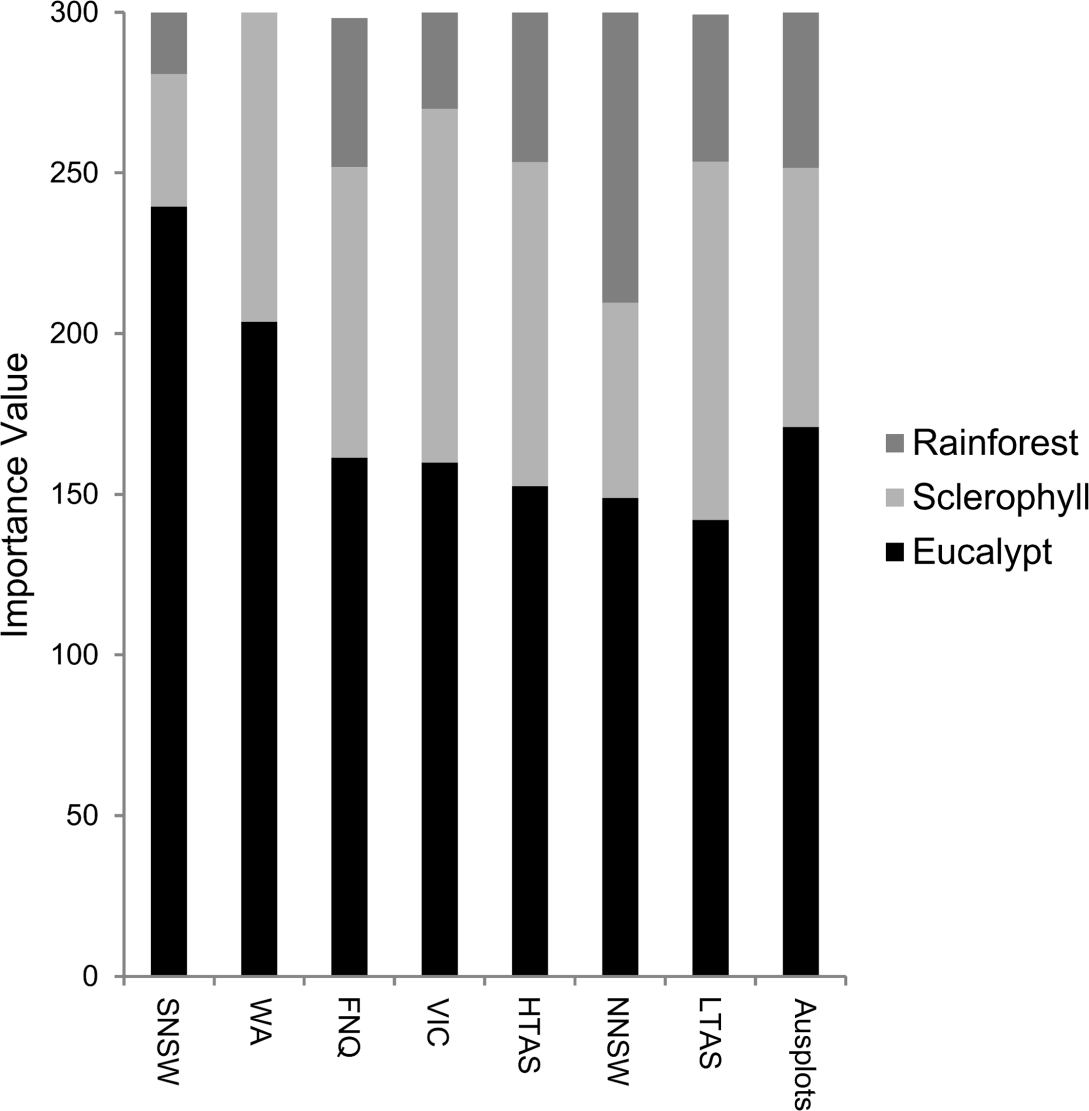
Importance Value of three community guilds (Eucalypt, Rainforest, Wet Sclerophyll) in the Ausplots Forest Monitoring Network and for each region. Calculations of the Importance Value are presented in S5 Table.

There were 185 tree species recorded in the 48 plots, including 30 eucalypts, 47 wet sclerophyll species and 108 rainforest species (S4 Table). The target tall eucalypts species clearly dominated the forest stands within their respective regions, with markedly higher importance values than other eucalypt and non-eucalypt species (Table 3). Eucalypts were clearly the most important forest community across the plot network (Fig 4). Wet sclerophyll species dominated the non-eucalypt understorey in most regions, although Far North Queensland, Northern NSW and Tasmania included several plots with a distinct tropical, subtropical and temperate rainforest flora, respectively (Fig 4). Important wet sclerophyll species include *Allocasuarina torlosa* (NNSW, FNQ), *Allocasuarina decussata* (WA), *Acacia melanoxylon* (LTAS, VIC, FNQ), *Pomaderris apetala* (LTAS, HTAS) and *Trymalium odoratissimum* (WA) and important rainforest species include *Nothofagus cunningamii* (TAS) and *Cryptocarya rigida* (NNSW) (Table 3).

**Table 3:** The five most important tree species in each region calculated using the Importance Index. Target eucalypt species are indicated with an asterisk (*). Community guilds are eucalypts (Euc), wet sclerophyll (Scl) and rainforest (RF). Frq = relative frequency, RDe = relative density, RDo = relative dominance, IV = Importance Value.

Species	Guild	Frq	RDe	RDo	IV
***Western Australia***					
*Eucalyptus diversicolor*	Euc	25.7	38.1	61.8	125.6
*Allocasuarina decussate*	Scl	17.1	25.0	7.7	49.8
*Trymalium odoratissimum*	Scl	5.7	10.5	25.6	41.8
*Eucalyptus jacksonii*	Euc	20.0	11.5	0.5	32.0
*Corymbia colophylla*	Euc	14.3	6.3	1.3	21.9
**Victoria**					
Eucalyptus regnans*	Euc	12.9	35.5	85.4	133.8
*Acacia melanoxylon*	Scl	11.3	19.5	7.9	38.8
*Nematolepis squamea*	Scl	4.8	16.6	3.1	24.5
*Olearia argophylla*	Scl	8.1	11.5	1.5	21.1
*Pomaderris aspera*	Scl	8.1	7.0	0.7	15.8
**Low Elevation Tasmania**					
Eucalyptus obliqua*	Euc	9.0	19.8	61.5	90.2
*Pomaderris apetala*	Scl	6.7	27.9	4.6	39.3
*Acacia melanoxylon*	Scl	9.0	9.0	8.9	26.9
Eucalyptus regnans*	Euc	4.5	6.3	13.4	24.3
*Nothofagus cunninghamii*	RF	6.7	9.6	3.6	20.0
**High Elevation Tasmania**					
Eucalyptus delegatensis*	Euc	9.8	26.9	65.5	102.2
*Pomaderris apetala*	Scl	3.9	22.0	3.8	29.8
*Acacia dealbata*	Scl	7.8	6.0	4.8	18.6
*Nothofagus cunninghamii*	RF	7.8	7.2	2.7	17.8
*Leptospermum lanigerum*	Scl	7.8	6.4	2.7	16.9
**SNSW**					
Eucalyptus fastigata*	Euc	15.2	33.4	36.6	85.2
Eucalyptus obliqua*	Euc	12.1	23.4	29.7	65.2
*Eucalyptus cypellocarpa*	Euc	12.1	14.6	17.4	44.1
*Eucalyptus viminalis*	Euc	6.1	11.0	10.1	27.1
*Eucalyptus radiata*	Euc	6.1	9.9	3.4	19.3
**Northern NSW**					
Eucalyptus pilularis*	Euc	4.4	10.7	48.0	63.2
*Allocasuarina torulosa*	Scl	5.0	18.5	8.2	31.7
Eucalyptus grandis*	Euc	1.3	6.1	12.5	19.9
*Syncarpia glomulifera*	RF	3.8	6.6	7.8	18.2
*Eucalyptus microcorys*	Euc	3.1	5.5	7.6	16.3
**Far North Queensland**					
Eucalyptus grandis*	Euc	5.6	26.6	62.5	94.8
*Allocasuarina torulosa*	Scl	2.8	16.6	12.4	31.8
*Corymbia intermedia*	Euc	5.6	15.7	10.1	31.5
*Acacia melanoxylon*	Scl	4.2	20.0	3.4	27.6
*Syncarpia glomulifera*	RF	1.4	3.1	4.7	9.2

### Representativeness

**Fig 5:**
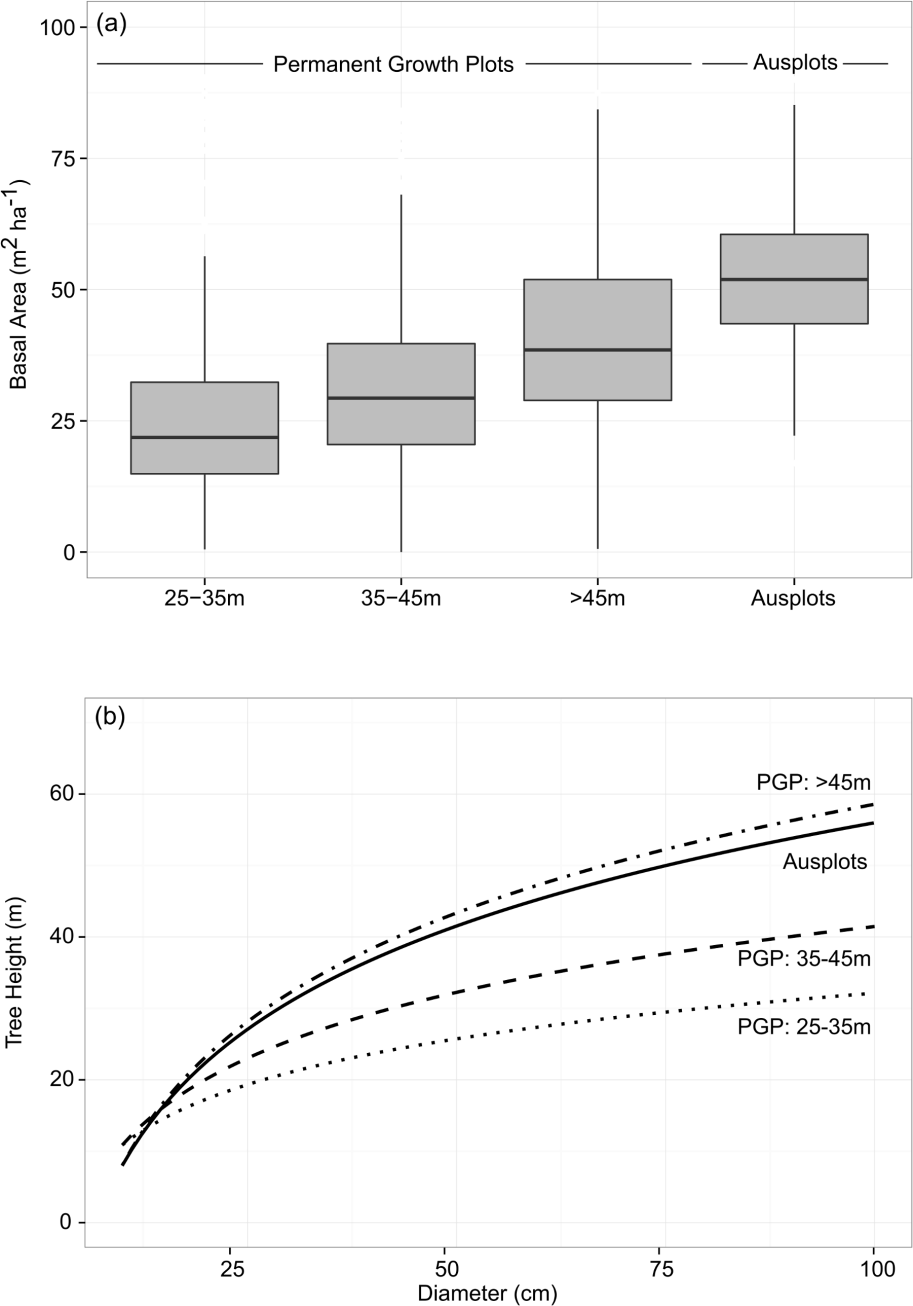
(a) Stand basal area and (b) diameter-height relationships for eucalypts the Ausplots Forest Monitoring Network (n = 48) compared to plots in the Permanent Growth Plot Network (PGP: n = 2409) assembled by Prior et al. [19]. Permanent Growth Plots were assigned height classes based on modelled relationships between maximum height and climatic variables (see S3 Text). Diameter-height relationships for both plot networks are based on measured (not modelled) heights. The Permanent Growth Plot Network included only eucalyptus species and therefore all non-eucalypt species were excluded from the Ausplots Forest Monitoring Network for this analysis.

The Ausplots Forest Monitoring Network is broadly representative of eucalypt forests classified as >45 m height, based on our height-climate relationship (Fig 5). Median basal area for the Ausplots Network was higher than forests classified as >45 m height, although their ranges had substantial overlaps (Fig 5A). For a given diameter, trees in the Ausplots Network achieved similar heights to those growing in highly productive forests classified as >45 m height and were considerably taller than trees growing in lower productivity forests classified as 25–35 m and 35–45 m (Fig 5B).

### Aboveground Carbon

**Fig 6:**
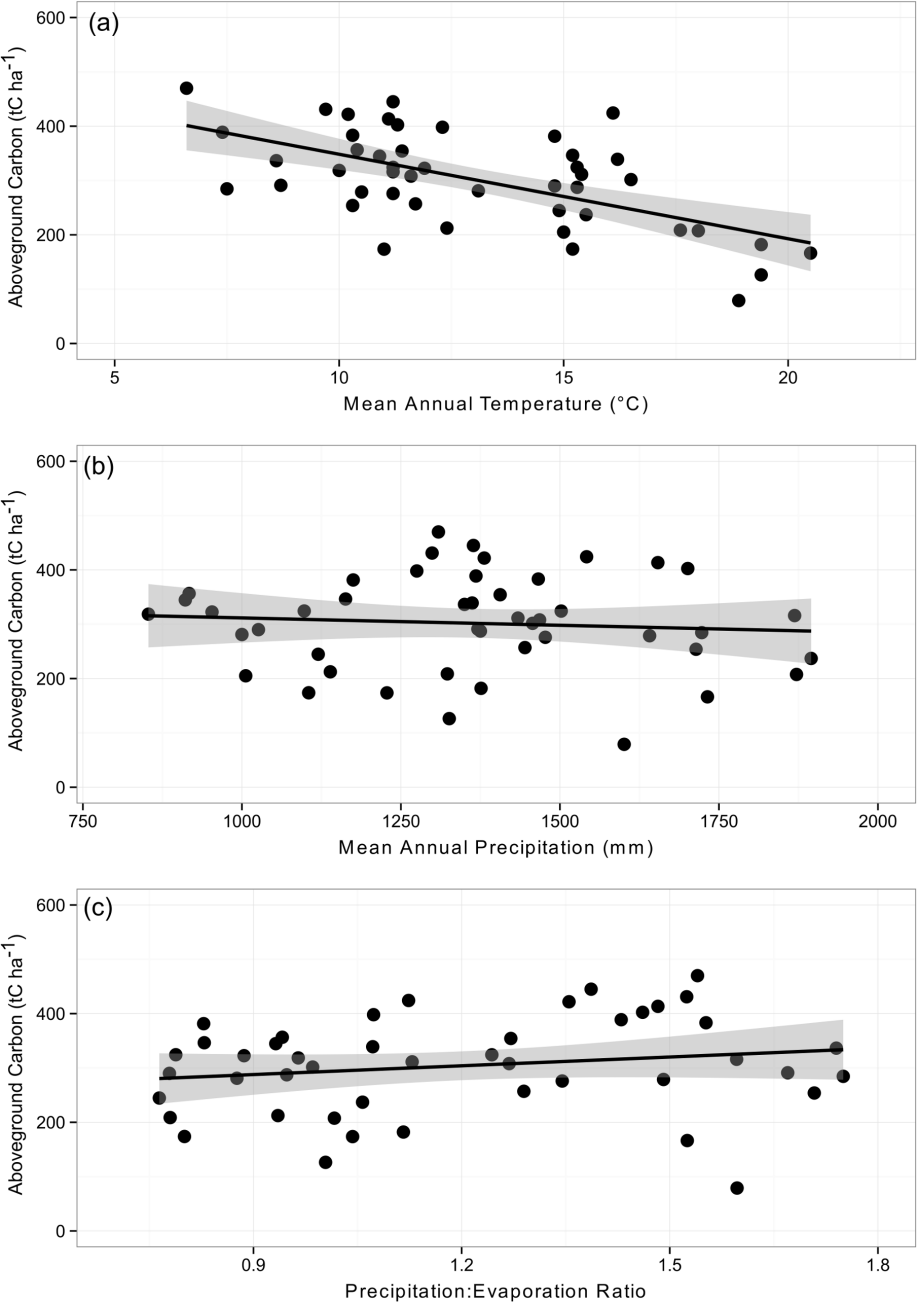
Relationships between aboveground carbon in living trees (AGC, tC ha^-1^) and (a) mean annual temperature (MAT; r^2^ = 0.36), (b) mean annual precipitation (MAP; r^2^ = 0.01) and (c) precipitation:evaporation ratio (P:E; r^2^ = 0.02). Linear models were fitted to the data and 95% confidence intervals are shown. AGC of two plots in Western Australia (WAFWAR005 and WAFWAR008) was not included because prominent buttressing of *Eucalyptus jacksonii* led to overestimates of AGC derived from diameter measurements.

AGC across the Ausplots Forest Monitoring Network was 302 tC ha^-1^ and ranged from 79 tC ha^-1^ in the *E*. *grandis* forests of Far North Queensland to 470 tC ha^-1^ in the *E*. *delegatensis* forests of Tasmania (Table 2; S2 Table). AGC was dominated by the eucalypts, which made up 90% of AGC (Table 2). For the eucalypts, most (49%) of the AGC was found in the 50–100 cm diameter range which dominated the stand structure of these forests (Fig 3), with the remainder in the 10–50 cm (9%), 100–150 cm (27%) and >150 cm (15%) trees (S1 Fig). The strongest climatic explanator for AGC was MAT (r^2^ = 0.36, AIC = 528, compared with AIC = 546 for the null model) (Fig 6). According to the linear model, AGC steadily decreases from approximately 400 tC ha^-1^ in cool climates with a MAT of 6°C to approximately 200 tC ha^-1^ in warm climates with a MAT of 20°C (Fig 6). There was very little support for the relationship between AGC and MAP (r^2^ = 0.01, AIC = 548) or P:E (r^2^ = 0.04, AIC = 546) (Fig 6).

## Discussion

Through the establishment of the Ausplots Forest Monitoring Network, the *Eucalyptus* forests that dominate the forests of the Australian continent now have the infrastructure and baseline measurements to facilitate their integration into global networks of forest monitoring plots. Relatively small numbers (n = 50–100) of carefully measured large (1 ha) monitoring plots have detected changes in tree growth, mortality and recruitment in the world’s forests, and have been used to understand how carbon fluxes, mortality events and compositional changes are controlled by spatial and temporal trends in climate (e.g. [5,9,14,15,27,57]). The plot network introduced here is of a similar scope to these well-established plot networks and by closely following their measurement protocols we have provided an unprecedented opportunity to compare the dynamics of tall eucalypt forests with forests on other continents. Tall eucalypt forests are strongly fire-driven ecosystems [36], and provide a model system for exploring the relative influence of climatic factors and post-fire successional stage on forest growth and dynamics (e.g. [58,59]). Thus, the Ausplots Forest Monitoring Network provides an important counterpoint to well-studied tropical forests that function largely independently of landscape scale disturbance events [9,29,60].

The Ausplots Forest Monitoring Network currently focusses on the very tall (>45 m) eucalypt forests of Australia. By standardising the network for forest type (tall forest species from the genus *Eucalyptus*), growth stage (mature forests; Fig 3) and site quality (high basal area on productive sites; Fig 4) this new plot network has maximised the potential to identify the effects of a wide macroclimatic gradient (Fig 2) on forest dynamics and carbon fluxes. The Ausplots Forest Monitoring Network complements the existing Australian continental-scale Permanent Growth Plot network (which only includes eucalypts monitored within small plots and is limited to analyses of tree growth; [20,21]) by allowing for the comprehensive tracking of *all* aspects of forest dynamics (including mortality and recruitment) for *all* tree species (eucalypt and non-eucalypt) within a relatively large area of forest (1 ha) appropriate to investigating stand-level forest dynamics.

### Eucalypt mortality

The Ausplots Forest Monitoring Network will deliver robust estimates of background mortality rates for mature eucalypt forests and is well placed to investigate the relationship between climate and trends in mortality over time. The mature forest system targeted by the plot network is likely to be at the tail end of the self-thinning process [61] and background eucalypt mortality rates in the short term are likely to be low and limited to suppressed trees growing below the forest canopy. In the long-term, mortality rates may increase (e.g. [62]) as the canopy dominants approach their maximum age (~350–500 years; [40,63]). Widespread mortality associated with periodic intense fires is extremely important in these systems but inherently difficult to quantify through plot-based monitoring because of their rare and highly episodic nature [47]. These infrequent landscape scale events are best quantified through remote sensing (e.g. [64,65,66]) and post-hoc ecological studies (e.g. [38,67,68]). However, opportunities may arise to characterise variation in post-fire responses of individual trees and forest stands if a regional subset of plots are burned at different intensities within a single fire event.

Studies using permanent plot networks have shown that rising temperatures and drought over the past few decades are driving increases in the background mortality in tropical [10], temperate [14] and boreal systems [15]. The importance of monitoring background mortality is highlighted by observations that increased background mortality rates could be symptomatic of forests that are stressed and vulnerable to abrupt and widespread mortality and dieback [69,12]. Eucalypts have a limited adaptive capacity [23,24,70], and Bowman et al. [20] speculated that in the long term, decreases in growth rates associated with a warming, drying climate could render eucalypts less resilient to climate induced stressors and disturbance events. The localised dieback recorded for tall eucalypt species such as *E*. *delegatensis* in Tasmania [71], together with widespread drought-induced mortality in *E*. *marginata* forests (~30 m height) in south-western Australia [72,73], provide considerable motivation for using the Ausplots Forest Monitoring Network to detect upward trends in mortality rates in tall eucalypt forests in response to climate.

### Understorey Dynamics

Tall eucalypt forest stands in the plot network consist of a distinct overstorey of 30–70 m tall eucalypt trees over a second stratum of 10–40 m high wet sclerophyll or rainforest trees (Table 2). Whilst the non-eucalypt species play only a minor role in the aboveground carbon stocks of tall forest stands (10% of AGC, Table 2), they form an important component of tree biodiversity (84% of tree species; S4 Table) and overall tree abundance (60% of all trees; Table 2). Little is known of the long term growth rates or compositional turnover of understorey trees in eucalypt forests and especially how they relate to climate. Wet sclerophyll species (e.g. *Acacia* spp.*; Allocasuarina* spp.) may be the most dynamic components of the tall forest system. Their relatively short life spans and shade tolerance are likely to translate to relatively high rates of mortality and recruitment, respectively. On the other hand, long-lived and continuously regenerating rainforests that occur in all tall forest environments (except Western Australia) may be less dynamic, but are ecologically important because they have the capacity to succeed the eucalypt overstorey in the absence of fire [63,33]. In the long-term the Ausplots Forest Monitoring Network will be able to quantify the magnitude and direction of the changes in carbon storage associated with this transition from eucalypt to rainforest across the macroecological gradient [74,75].

### Aboveground Carbon

Our estimate of mean AGC (302 t ha^-1^) is at the lower end of estimates for tall eucalypt forests (199–1053 t ha^-1^; reviewed by [34]; see also [75,76,77]). The considerable variation in estimates of AGC reflects the natural variability within the tall eucalypt forest system, and it is possible that relatively low estimates for the Ausplots Forest Monitoring Network may reflect a lack of very large trees >150 cm that dominate carbon budgets in higher biomass forests [34,76]. Considerable uncertainty in the magnitude of AGC is also introduced by the choice of allometric equations used to predict AGB [78,79]. Our baseline estimates of AGB (and therefore AGC) were derived from general equations for eucalypt and rainforest trees and will be improved as region- and species-specific allometric equations from new or existing data becomes available. For example, allometric equations using both diameter and height as predictors of AGB based on large numbers (up to 98) of directly weighed trees are currently being developed for very tall *E*. *regnans* and *E*. *pilularis* forests within regions targeted by the Ausplots Forest Monitoring Network [80].

AGC is strongly negatively correlated with MAT in tall eucalypt forests, whereby forests experiencing cool climates have approximately double the carbon stocks of those in warm climates. This is supported by other macro-ecological analyses that have reported maximum AGC in cool, wet environments [34,81,82]. Liu et al. [82] also found that AGC decreased along a temperature gradient ((MAT 8°C—30°C) across the world's mature forests. However, there may be regional exceptions to this relationship [82], such as the temperate forests of the Americas [81] and some tropical biomes [83,81]. The implication of a strong negative relationship between AGC and MAT is that a predicted warming climate in Australia may reduce carbon storage in tall eucalypt forests [20,82]. However,this relationship is merely correlative at this stage and the mechanisms that determine this relationship require closer examination.

The negative relationship between AGC and MAT may be partly explained by thermal constraints on individual tree growth. Bowman et al. [20] showed that eucalypt growth rates are maximal in forests experiencing a MAT around 11°C and that growth rates decreased in both cooler and warmer climates. These trends were also apparent in growth rates of individual tall eucalypt species [36], and were especially pronounced in larger trees that dominate this forest type [21]. However, MAT effects on growth rates do not explain the increase in biomass as MAT falls from 11° to 6°C. This could be explained by (a) macro-climatic differences in recruitment and mortality rates that have yet to be elucidated from long-term tree measurements; (b) differences in disturbance histories; (c) site-specific factors such as soil quality or landscape setting; or (d) other climatic drivers or untested interactions between climatic variables. Importantly, the high quality data required to test many of the scenarios we put forward here will become available through future censuses of the Ausplots Forest Monitoring Network and as more detailed information on site-based parameters (e.g. soil nutrition, fire history, climatic variables) become available.

### Future Opportunities

The current set of 48 plots provides a framework around which a more comprehensive network can be developed. Expansion of the plot array could focus on other growth stages within the tall eucalypt forest system (e.g. young regenerating regrowth <70 years old or old growth >250 years old) to test the relative importance of climate and disturbance history on forest dynamics (e.g. [58]). Alternatively, the plot network may extend to other forest types at the wetter (e.g. rainforest) or drier ends of the climate spectrum (e.g. dry sclerophyll forest, savannah and woodlands). Additional measurements can also be made within each plot to contribute to our understanding of potential feedbacks between a warming climate, increased mortality and the build-up of fuels in these inherently flammable forests [33]. We are currently (a) collecting soil samples (to quantify soil nutrition) and forest micro-climate data as additional explanatory factors in models of forest dynamics, (b) collecting data on other components of the carbon pool (i.e. litter and coarse woody debris) and (c) measuring seedlings and saplings <10 cm DBH to generate a more comprehensive picture of stand dynamics and carbon stocks.

The high resolution spatial mapping of all trees within each plot (S2 Text) provides opportunities for exploring the effect of intraspecific and interspecific competition in shaping the ecology of tall eucalypt forests. For instance, Prior and Bowman [22] showed that the strength of inter- and intra-specific competition amongst eucalypts is particularly high given these species are strongly light-demanding, resulting in two tiered canopies described as ‘hyper-emergence’ by Tng et al. [33]. Mapped locations of individual trees provides an opportunity to develop detailed indices of tree competition and use the rapidly developing techniques of spatial pattern analysis [84,85] to analyse how dominant eucalypt tree species in a forest compete and partition space in relation to other eucalypt species and their non-eucalypt understorey neighbours (e.g. [86,87]). Spatial maps of trees will also be crucial for validation of aerial and ground based remote sensing (e.g. light detection and ranging; LIDAR) that is becoming increasingly important in providing landscape scale structural information on forest ecosystems [88].

### Conclusions

We have established a network of 48 1 ha forest plots across a macro-climatic gradient in the tall eucalypt forests of Australia. By carefully standardising vegetation type and growth stage, the plot network is well placed to track trends in tree growth, mortality and recruitment of forest trees to elucidate the effects of climate on forest structure, composition and carbon dynamics over large spatial scales. Measurement protocols closely follow those established for well established, state-of-the-art forest monitoring networks elsewhere in the world (e.g. RAINFOR, AfriTRON) allowing for the inclusion of eucalypt dominated forests into global meta-analyses. The plots have already yielded useful baseline data that have been used to demonstrate a strong negative relationship between AGC and MAT, whereby tall eucalypt forests in warm climates store approximately half the amount of carbon in live aboveground biomass than those growing in cooler climates. In the long term, the Ausplots Forest Monitoring Network will be particularly important for determining whether drought and temperature induced increases in background mortality found elsewhere in the world are occurring in the tall eucalypt forest ecosystem.

The success of long term monitoring initiatives is contingent on robust design, easily relocatable infrastructure, thorough documentation and the accessibility of data [25,26]. We have outlined the design and rationale for the Ausplots Forest Monitoring Network including detailed metadata on each plot (S2 Text and S1 File; S1 and S2 Tables) and a comprehensive survey protocols manual (S1 Text). The dataset is publically available through the Australian Ecological Knowledge and Observation System Data Portal (AEKOS; [89]).

## Supporting Information

S1 Text Ausplots Forest Monitoring Network Survey Protocols Manual. Protocols for plot establishment, tree survey, voucher specimen collection, canopy photography, soil sampling and fuel surveys (90 pages).
(PDF)

S2 Text Ausplots Forest Monitoring Network Plot Establishment Report. Location, configuration, stand attributes and baseline data for 48 forest plots (115 pages).
(PDF)

S3 Text Predicting eucalypt height as a function of climate for the Prior et al. (2011) Permanent Growth Plot Network. Description of methodology and analysis undertaken to classify the Prior et al. (2011) Permanent Growth Plot network into three height classes based on mean annual temperature and mean annual precipitation.
(PDF)

S4 Text Scientific Research Permits issued during the establishment of the Ausplots Forest Monitoring Network 2012–2015. (PDF)

S1 Table Summary of the details and environmental parameters of 48 plots in the Ausplots Forest Monitoring Network. The means (and range) of climatic variables and elevation are presented. MAT = Mean Annual Temperature. MAP = Mean Annual Precipitation.
(PDF)

S2 Table Stand structural attributes of 48 plots in the Ausplots Forest Monitoring Network. The means (and range) are presented for eucalypt (including Eucalyptus spp. and Corymbia spp.) and non-eucalypt species.
(PDF)

S3 Table The Hutchinson agro-climatic classification and its relationship to the global Koppen-Geiger climate classification. Only those classificatons relevant to the Ausplots Forests Monitoring Network are shown.
(PDF)

S4 Table List of species censused in the Ausplots Forest Monitoring Network and their respective community guild. Euc = Eucalypt, Scl = Wet Sclerophyll, RF = Rainforest. Unidentified species are prefixed with UNN and have been vouchered for identification by local herbaria.
(PDF)

S5 Table Importance Values for forest community guilds (Eucalypt, Rainforest, Wet Sclerophyll) for each region and for the Ausplots Forest Monitoring Network. RDe = relative density, RF = relative frequency, RDo = relative dominance, IV = Importance Value.
(PDF)

S1 Fig Distribution of live aboveground carbon (AGC; tC ha^-1^) in *Eucalyptus* species across diameter classes in the Ausplots Forests Monitoring Network. (PDF)

S1 File Google Earth file (.KMZ) of the location of 48 plots in the Ausplots Forest Monitoring Network. Geolocation of each corner (0,0), (0,100), (100,0), (100,100) of the 100 m x 100 m plot with an accuracy of ±10 m.
(KML)
