# Supplementary material for: Macroecology of Australian Tall Eucalypt Forests: Baseline Data from a Continental-Scale Permanent Plot Network
Source: PLoS One. 2015 Sep 14;10(9):e0137811. doi: 10.1371/journal.pone.0137811 (PMC4569531; doi:10.1371/journal.pone.0137811)
Supplement: S1 Text — Protocols for plot establishment, tree survey, voucher specimen collection, canopy photography, soil sampling and fuel surveys (90 pages). (PDF) [file pone.0137811.s001.pdf]

Ausplots Forest Monitoring Network

Survey Protocols Manual

Version 1.6.

Sam Wood, Helen Stephens, Jeff Foulkes, Elinor Ebsworth,

David Bowman.

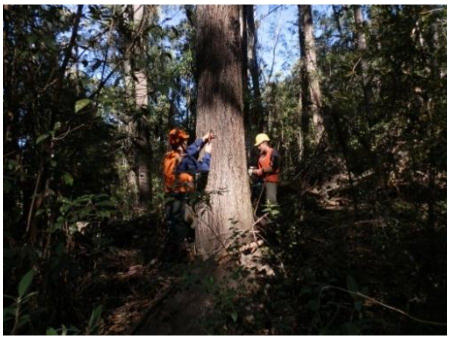
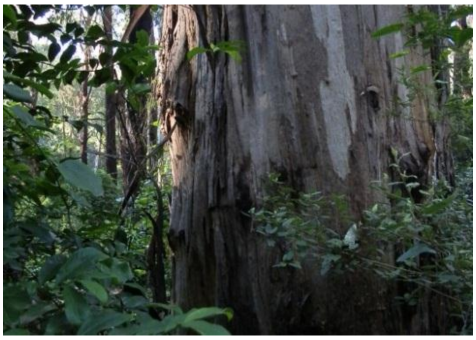
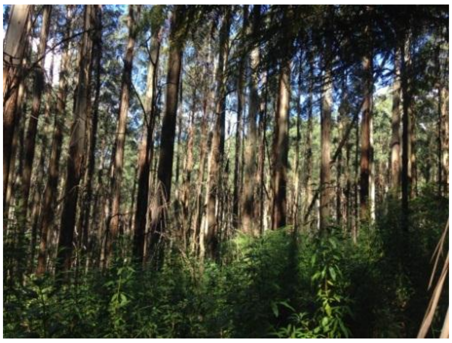

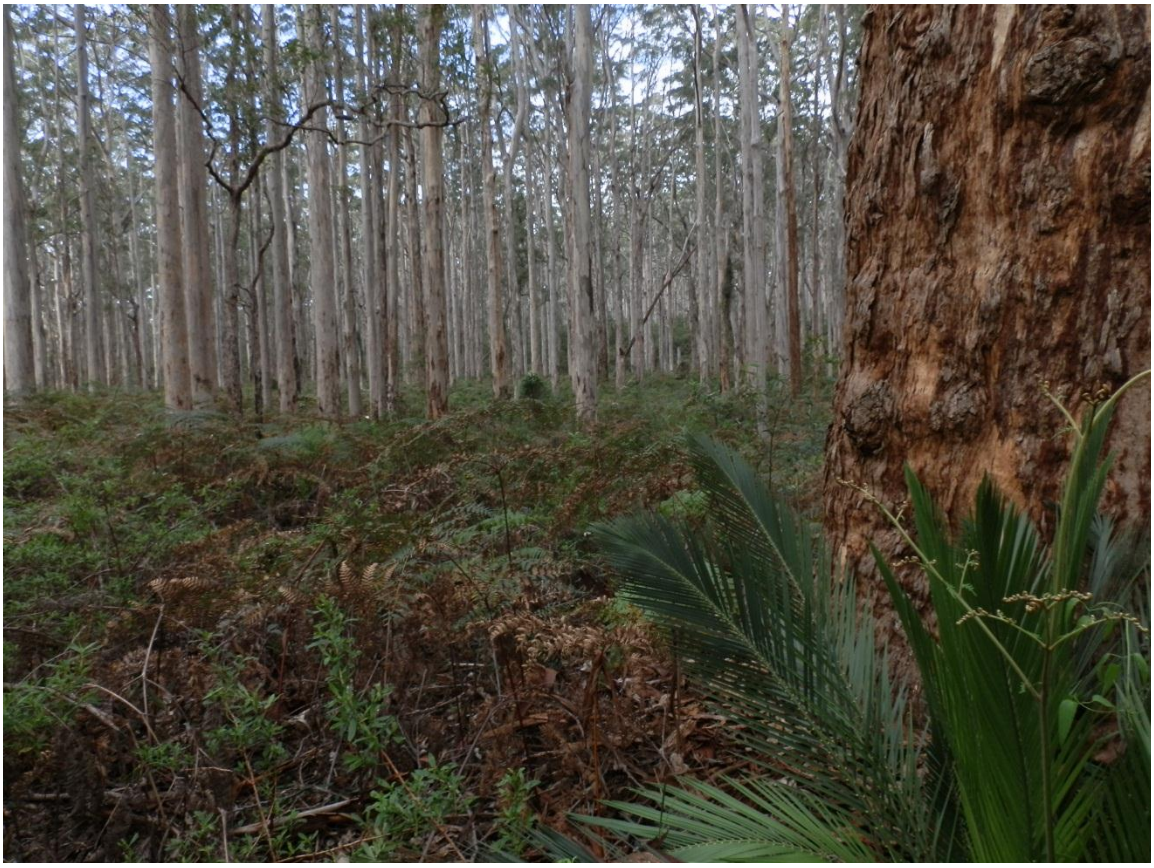

Ausplots Forest Monitoring Network	Terrestrial Ecosystem Research Network
School of Biological Sciences<br>University of Tasmania<br>Private Bag 55,<br>Hobart, Tasmania 7001<br><br>www.tern.org.au/AusPlots	University of Adelaide<br>Level 12 Schulz Building<br>North Terrace Campus<br>Adelaide, South Australia 5005.<br><br>www.tern.org.au

Publication details

This online edition of the TERN Ausplots Forest Monitoring Network Monitoring Network Survey Protocols Manual was produced and published by the University of Tasmania. It is provided as an online resource. Please do not republish material from this document or its attachments without written consent.

Table of Contents

1. INTRODUCTION 5

1.1. BACKGROUND AND RATIONALE 5

1.2. SCOPE OF SURVEY PROTOCOLS MANUAL 6

1.3. LINKS TO OTHER SURVEY PROTOCOLS. 7

1.4. OVERVIEW OF SURVEY PROTOCOLS MANUAL 7

2. TRIP PLANNING 10

2.1. GUIDELINES 10

2.2. PERMITS AND QUARANTINE 10

2.3. FIELD EQUIPMENT, VEHICLES AND CHECKLISTS 10

2.4. VOUCHERS AND BARCODES 11

2.5. SURVEY PARTICIPANTS 11

2.6. PRE-SURVEY MEETING 11

2.7. SCHEDULED CALL-INS 12

2.8. DATA COLLECTION/RETURN 12

2.9. TIME REQUIREMENTS 12

2.10. EQUIPMENT LISTS 13

3. PLOT LAYOUT AND POSITIONING 16

3.1. GENERAL PLOT CONFIGURATION 16

3.2. PLOT LOCATION AND CONFIGURATION IN THE FIELD 17

3.2.1. LOCATION AND ORIENTATION OF A NEW PLOT 17

3.2.2. LOCATION AND ORIENTATION WITH AN EXISTING PLOT 19

3.3. PEGGING OUT THE PLOT. 21

4. SITE DESCRIPTION MODULE 23

4.1. PLOT IDENTIFICATION 23

4.2. GPS COORDINATES OF CORNER POSTS AND COMPASS BEARINGS OF X AND Y AXES 25

4.3. MUD MAP OF THE SITE AND PLOT 26

4.4. LANDFORM OF PLOT 27

4.5. SPECIES LIST AND UNDERSTOREY DESCRIPTION 31

5. LARGE TREE SURVEY MODULE 32

5.1. PLOT INFORMATION 35

5.2. TREE ID, TREE STATUS (DEAD/ALIVE) AND SPECIES 35

5.3. TREE DIAMETER AND POINT OF MEASUREMENT 37

5.4. X and Y coordinates 39
5.5. Tree Growth Stage and Crown Class 42
5.6. Tree Condition and Mode of Death. 44
5.7. Tagging Trees 47
5.8. Tree and Bole Height 49

6. VOUCHERING OF SPECIMENS AND GENETIC/ISOTOPE SAMPLES 51

6.1. Voucher specimens 51
6.2. Genetic and isotope sample vouchering 54

7. SOIL METAGENOMICS 57

8. CANOPY PHOTOGRAPHY 59

9. FUEL LOAD SURVEY 61

9.1 Fuel transect allocation 61
9.2 Fuel Survey datasheets 64
9.3 Fuel and grass height measurements 65
9.4 Woody fuel counts 67
9.5 Shrub biomass 68
9.6 Fine litter, grass, herbs and vines measurement 70

10. SOIL SAMPLING 72

11. INSTALLATION OF LITTERFALL TRAPS 73

12. INSTALLATION OF TEMPERATURE AND HUMIDITY LOGGERS 74

13. INSTALLATION OF LITTER DECOMPOSITION BAGS 75

14. REFERENCES 77

15. APPENDICES 79

15.1. Large Tree Survey Data Sheet (One Page) 79
15.2. Site Description Data Sheet (Six Pages) 79
15.3. Voucher Specimen Data Sheet (One Page) 79
15.4. Soil Metagenomics Data Sheet (One Page) 79
15.5 Fuel Survey Data Sheet (Two Pages) 79

1. Introduction

1.1. Background and rationale

Understanding long term patterns of forest dynamics is fundamental to managing for the multiple values of the Australia's forest estate. Since the 1950's, forest monitoring in Australia has been undertaken for a variety of reasons. Traditionally forest agencies collected forest inventory data from plot networks (see Prior *et al.* 2011) to measure the growth, recruitment and mortality of trees in production forests to develop yield models to maximise sustainable timber extraction (Weiskittel *et al.* 2011). More recently the focus of monitoring plots in Australian have shifted toward understanding stocks and fluxes of carbon in forests (e.g. Ximines *et al.* 2013; Moroni, 2012); tree cover (Wood *et al.* 2006); ecosystem function and biodiversity (e.g. McCaw *et al.* 2011, Murphy *et al.* 2013); and the disturbance ecology of fire-driven forest ecosystems (e.g. Lindenmayer 2009; Turner *et al.* 2009).

Until recently, forest monitoring plot networks in Australia were always arranged at local to regional scales and have been insufficient to track continental scale trends in a range of forest values including tree growth, biodiversity, timber resources, soil and water, carbon and forest health (Wood *et al.* 2006). Networks of strategically located forest monitoring plots arranged across broad geographic scales can be used to track regional trends in tree growth (Bowman *et al.* 2013) and forest dynamics (Condit *et al.* 2000). For example, macro-ecological studies based on continental-scale plot networks in tropical forest of South America and the temperate forests of North America and Europe have shed light on the effects of climate and climate change on forest growth (e.g. Lewis *et al.*, 2004; Reich and Oleksyn 2008; Phillips *et al.*, 2008; Pan *et al.*, 2011), tree mortality (e.g. van Mantgem & Stephenson, 2007; van Mantgem *et al.*, 2009; Lewis *et al.*, 2004b; Phillips *et al.*, 2009) and ecosystem function and biodiversity.

Drawing on this work, three recent macro-ecological studies (Prior et al. 2013, Bowman *et al.* 2014, Prior and Bowman 2014) exploited the wide temperature and rainfall gradients across the Australian continent to investigate relationships between tree growth and climate to make predictions of how eucalypt forests may respond to climate change. They found that eucalypt growth is positively correlated with water availability but negatively related to mean annual temperatures in excess of 11°C (Bowman *et al.* 2014) This work predicted that increased temperatures may reduce tree growth across eucalypt forests (Bowman *et al.* 2014) and that large trees may be more vulnerable (Prior and Bowman 2014). These studies were based on observations from a compilation of 2409 permanent plots in Australia's eucalypt dominated, temperate mesic forests spanning climates ranging from cool temperate wet to subtropical dry (Prior *et al.* 2011).

Whilst the retrospective studies of Prior and Bowman (2014) and Bowman *et al.* (2014) provide an important starting point for macro-ecological studies of forest dynamics in Australia, it is likely that they constitute an opportunistic 'one-off' study because a significant proportion of the plots in the network the assembled have been

discontinued due to shifting priorities of some of the State-based management agencies responsible for their re-measurement. Furthermore, inconsistent measurement protocols between States have hindered the interpretation of continental trends for other important aspects of forest dynamics (other than individual tree growth) such as (a) recruitment and mortality of trees, (b) the dynamics of understorey trees, seedlings and saplings; (c) carbon stocks in non-tree pools; (d) floristic and biodiversity measures; (e) fuel loads, etc.

Given the limitations and uncertainties surrounding the ongoing measurement of the only continental-scale forest plot network in Australia, Ausplots Forest Monitoring Network was formed under the auspices of the Terrestrial Ecosystem Research Network (TERN). TERN is a national collaboration of researchers, infrastructure and processes that enables the collection, storage, sharing and use of long-term ecosystem data sets and knowledge. TERN is establishing continental scale data collection processes and mechanisms to facilitate sharing of long term ecosystem data sets across disciplines.

The overall objective of TERN is to "provide a national institutional infrastructure network for terrestrial ecosystem research" under which AusPlots Forest aims to:

Establish a continental-scale plot based monitoring network that improves our understanding of tree growth, forest productivity and carbon dynamics in eucalypt forests in relation to macro-environmental gradients across Australia.

A key component of Ausplots Forest Monitoring Network is to establish a common set of attributes to be measured, to consistent standards at regular intervals. Critically, the nationally consistent methodology should be adaptable for a range of forest types and facilitate unambiguous comparisons of changes in forest dynamics across the Australian continent over time.

1.2. Scope of Survey Protocols Manual

The Ausplots Forest Monitoring Network Survey Protocols Manual outlines the field methodology for Ausplots Forest Monitoring Network. The background and rationale of the project and details of bioregional stratification and site selection will be outlined in a separate document. For the purposes of this manual, it is assumed that plot locations have been identified.

Version 1.0 of the manual will detail the methodology for (a) the installation of plot infrastructure, (b) the description of the site and (c) the core measurements that form the minimum dataset for Ausplots Forest Monitoring Network plots (Table 1)

Version 1.6 of the manual will detail the methodology for other forest ecology measures such as (a) coarse woody debris, (b) canopy cover and floristics; (c) seedlings and saplings; (d) soil attributes; (e) fuel loadings, etc. (Table 1).

1.3. Links to other Survey Protocols.

Ausplots Forest Monitoring Network has closely followed survey protocols developed by established national and international plot networks. This methodological consistency ensures that the data can be seamlessly integrated into existing forest inventory databases (e.g. Forestplots.net) and contribute to global meta-analysis of forest dynamics (e.g. Pan *et al.*, 2011; Stephenson *et al.* 2014). The protocol regarding the large tree survey draws heavily on the RAINFOR initiative (www.rainfor.org) but also includes methodological protocols from (a) State Forest Agency methodologies, including the recently established Victorian Forest Monitoring Program; (b) Australian government guidelines such as the Continental Forest Monitoring Framework (Wood *et al.* 2006) and AusPlots Rangelands (White *et al.* 2013); and other international projects (TEAM, TROBIT, GEM).

1.4. Overview of Survey Protocols Manual

The Ausplots Forest survey process is outlined in Figure 1. This manual documents the methodology for each step in a separate chapter (Table 1). Datasheets for recording data in the field are included in the Appendices.

Figure 1: Diagram of Ausplots Forest Monitoring Network survey process.

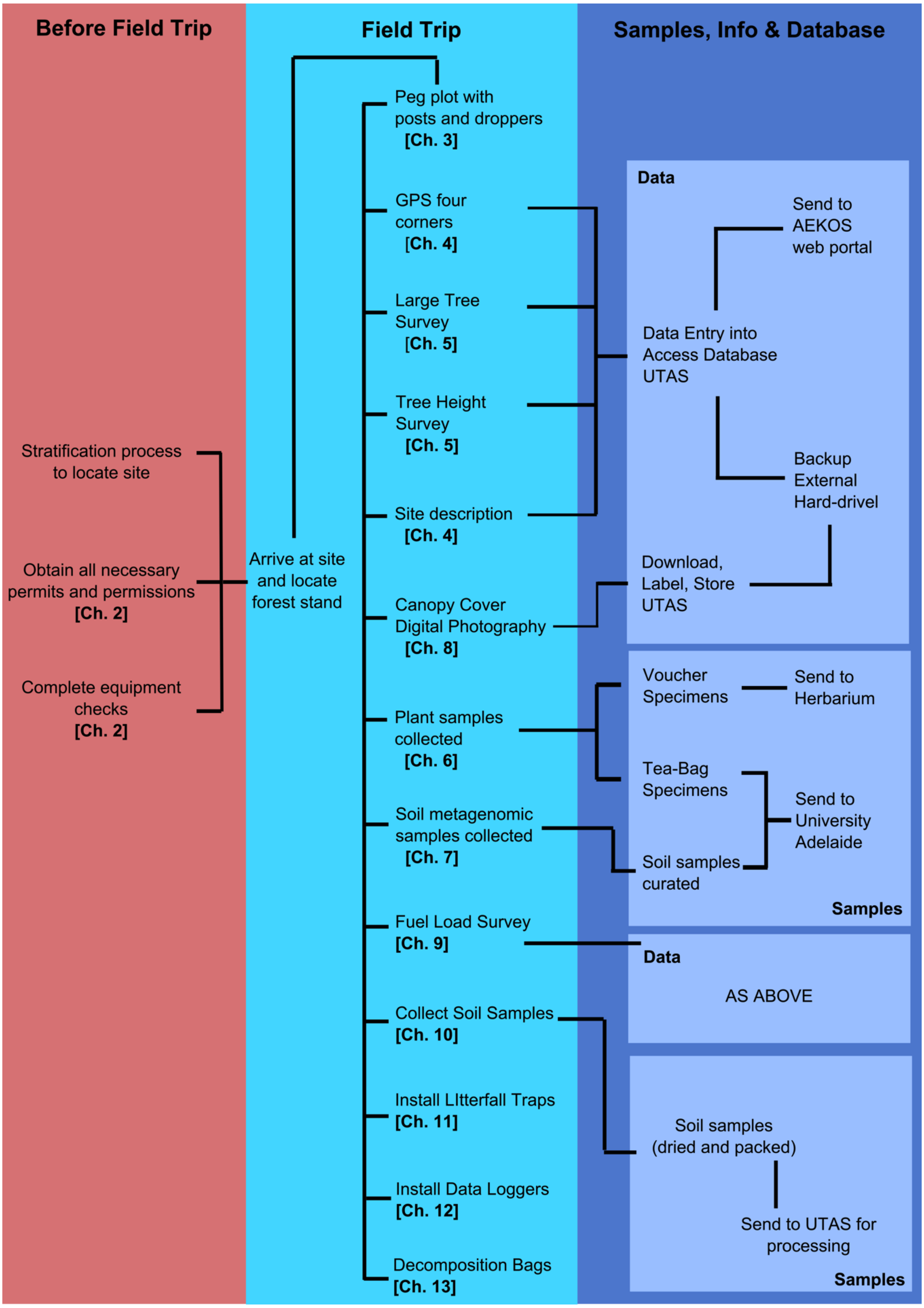

Table 1: Summary of methods, protocols and estimated timing for an average plot. These modules can be completed in separate site visits (i.e. Survey 1, Survey 2, Survey 3).

Survey	Chapter	Method	Protocol	Details	Time
**1**	**2**	Trip Planning	Prepare for field	OHAS, Equipment, permits	2 days
**1**	**3**	Peg out plot	Construct grid using tapes and compass	Star picket each corner and steel droppers at 20x20m intersections	3-5 hrs*
**1**	**4**	Plot Description	Details of site	Includes terrain, disturbance and site history classifications	30 min
**1**	**4**	GPS corners	GPS and data sheet	Use DGPS if available, record accuracy if GPS	30 min
**1**	**5**	Large Tree Survey	Diameter, location, description of trees >10cm DBH.	x,y coordinate, species, form, DBH, crown class.	10-20 hrs**
**1**	**5**	Tree Height Survey	Tree height across DBH range	Vertex for ~40-60 dominant' and ~40 subdominant trees	3-6 hrs
**1**	**6**	Plant Collection	Voucher samples for each species	Barcoded vouchers identified to species level by Herbarium	1-2 hrs + 2-3 hrs
**1**	**6**	Genetic samples	Sub-sample from vouchers	Barcoded Samples in tea bags sent for genetic analysis	1 hr
**1**	**7**	Soil samples - metagenomics	Surface soil samples at nine locations	200g sample taken to 3cm, barcoded and bagged with silica gel	1 hr
**1**	**8**	Canopy Photography	Sixteen canopy photos at internal points	Using fisheye lens.	1 hr
**2**	**9**	Fuel Loads	Understorey and litter biomass	Fuel height, shrub biomass, woody fuel counts, biomass quadrats.	4-6 hrs
**2**	**10**	Soils	Soil and duff samples	Soil carbon, nitrogen and nutrient content.	0.5 hrs
**2**	**11**	Litterfall traps	Installation of litterfall traps	Assemble litterfall traps on-site.	1 hr
**2**	**12**	Dataloggers	Install dataloggers	Installation of temperature and humidity dataloggers.	1 hr
**2**	**13**	Decomposition	Litter decomposition rate	Manufacturing and installation of litter decomposition bags.	1 hr
**3**	**Manual 2.0**	Seedling and Sapling Survey	AusPlots Survey Protocols Manual 2.0	20x20m or 4 x 100m subplots for saplings; 2x2m subplots for seedlings	TBD
**3**	**Manual 2.0**	Annual litter accumulation	Collection of litter from litterfall traps	Mass of litter from litterfall traps. Determine moisture content.	TBD
**3**	**Manual 2.0**	Annual climate variation	Temperature and humidity data	Collect temperature and humidity dataloggers	TBD
**3**	**Manual 2.0**	Decomposition rate	Collection of litter decomposition bags	Determine moisture content and biomass loss.	TBD

* depending on understorey density; ** depending on tree density; TBD = To Be Determined

2. Trip Planning

2.1. Guidelines

These trip planning guidelines should not replace local guidelines and operating procedures, but rather ensure that field teams have considered all the requirements for conducting Ausplots Forest Monitoring Network surveys. Where these guidelines conflict with local guidelines it is recommended that local guidelines are used, except sections relating to specific methods and equipment.

Please follow the Occupational Health and Safety (OH&S) procedures of your organisation.

Each team will have a requirement to complete a field trip approval/advice prior to conducting field surveys, with associated standard operating procedures or local guidelines for communication, vehicles, equipment, etc. These requirements must be fulfilled.

2.2. Permits and Quarantine.

Conducting Ausplots Forest Monitoring Network surveys may require several permits to be obtained from local institutions such as:

- Permit to collect
- Permit to conduct scientific research
- Permit to access Aboriginal Lands
- Aboriginal Areas Protection Authority approval
- Import and Export permits – Quarantine
- Defence Permits
- Parks Permits
- Quarantine areas: weeds, pathogens, etc.

The relevant land management agencies should be contacted early and briefed on the planned field activities. Carefully check the permit process for each jurisdiction and note the nominated time taken to evaluate applications (often 4-8 weeks). Ensure that formal applications for permits are submitted in a timely manner to prevent delays.

2.3. Field Equipment, Vehicles and Checklists

Equipment lists and checklists are provided at the end of this section (see 2.10) to serve as both an indicative minimum requirement and also as a basis from which to develop individual requirements. The Ausplots Forest Monitoring Network Team can be contacted for advice on suppliers of equipment and materials required for plot

establishment. Ausplots Forest Monitoring Network bases data collection on hardcopy datasheets printed on waterproof paper (see Appendices). As well as generic field equipment sheets, additional checklists and inventory sheets should be developed by individual operators to ensure a complete field equipment complement is carried on each trip.

Field operation will usually need a 4WD vehicle that is equipped appropriately for the environment where the work is to be undertaken. All vehicles should have suitably stocked first aid kits. In some instances, i.e. trips for long duration, a trailer may be needed for transportation of samples collected over the trip. Ensure organisational procedures and guidelines developed for 4WD use and remote area work are followed. This manual makes the assumption that local guidelines will be followed.

2.4. Vouchers and barcodes

Adhesive barcode labels with voucher labels will be assigned and provided to each Ausplots Forest Monitoring Network team by TERN headquarters in Adelaide (contact details on Page 2). Code conventions for each label follow strict protocols based on state, IBRA bioregion and plot type. Vouchering protocols are discussed in detail in Chapter 6.

2.5. Survey participants

Surveys should have a minimum of two participants with relevant expertise in vegetation survey. Work flow is most efficient with three survey participants and some sections of these protocols assume this number of people. Where volunteers are included, the necessary arrangements need to be completed prior to the trip with the necessary forms, approvals and notifications finalised. These will differ from jurisdiction to jurisdiction. Field teams should include participants with current Senior First Aid Certificate and experience and/or qualifications for operating a 4WD in off-road situations.

2.6. Pre-survey meeting

Conduct at least one pre-survey meeting to ensure all participants are in agreement regarding the aims and objectives of the trip, equipment provided, likely timelines, trip duration and flexibility on return times etc. This is also necessary for planning logistics for the trip and for assigning responsibilities between trip participants. This will become routine after completion of the first couple of trips. At this meeting an inventory should be compiled of relevant data available for the areas being surveyed e.g. plant lists for the area obtained from the local herbarium, details of past biological surveys etc. and copies made to take into the field. The Atlas of Living Australia (www.ala.org.au) is a useful starting point for this information.

2.7. Scheduled call-ins

Scheduled call-ins are essential to satisfy occupational health and safety requirements, though in most cases there will be local requirements for this in remote areas. You should routinely call the relevant land management agency responsible for the forest block you are working in before, during and after the field trip.

2.8. Data collection/return

Most of the data is collected on hardcopy data collection sheets (see Appendices). Participants should routinely photograph each field sheet at the end of each day as a backup copy. Field sheets should be scanned and photocopied at the earliest possible opportunity. Some digital information is collected in the GPS and digital camera and these should be backed up on a laptop at the end of each day. Specific details will be provided within each section of the manual. All vouchers need to be prepared i.e. changing paper or silica granules or drying soils and putting them into approved containers, and then submitted to relevant institutions.

2.9. Time requirements

Depending on the nature of the forest stand, three survey participants (two vegetation experts and one generalist) should be able to complete one plot in 3-5 days. For example, in the low density *Eucalyptus diversicolor* forests of Western Australia with a sparse understorey, plots can be completed in 3-4 days. In Tasmania, with >1000 stems/ha and a dense shrubby understorey, plots may take 5 days. This assumes easy access to the plots and short travel distances between plots. There are also time requirements for preparation and dispatch of samples as well as updating databases when results are returned.

2.10. Equipment Lists

Safety (Chapter 2)

First Aid Kit	Wet Weather Gear
Satellite Phone	Spare Keys for 4x4
EPIRB	Sunscreen, repellent
Risk Assessment	Emergency Contacts
Permits	

Plot Layout Module (Chapter 3)

Maps and GPS location details	Flagging Tape
Handheld GPS	4 x star pickets
Compass	32 x steel droppers
2 x 20m tape	36 yellow caps for steel droppers
1 x 100m tape	Sledgehammer
Waterproof notebook	Laminated Plot Layout

Site Description Module (Chapter 4)

Maps and imagery of site	Handheld GPS
Laminated reference tables	Compass
Clipboard and pen/pencil	Clinometer
Site Description Datasheet	Waterproof notebook

Large Tree Survey Module (Chapter 5)

1 x clipboard and pencil/pens	1 x hammer
35 x Data Sheets (waterproof paper)	2 x toolbelt (nails & tags - tapes & paint)
Sharpies	3 x tree marking paint
1 x 20m tape (metric on each side)	4 x diameter tapes (2m and 5m)
1 x Vertex Hypsometer and Transponder	1.5m measuring pole (marked every 10cm)
Field Guides to trees and plants	3-5m ladder for buttressed trees
Laminated reference tables	Aluminium tags (n=1-1000)
Flagging Tape	Aluminium nails (n=1000)

Voucher Specimen Module (Chapter 6)

Secateurs	Newspaper (tabloid size) and cardboard
Hand trowel	Adhesive voucher labels (with barcodes)
Paper bags (small) for temporary storage	Plant identification references
Envelopes for small plants	Voucher Specimen Data Sheet.
Plastic bags for storage and transport	Extendable tree pruners
Plant presses and straps	Tarpaulin gardening bag

Genetic Sample Module (Chapter 6)

Tea bags
Sealable airtight lunch boxes
Silica granules: self indicating (10%) + standard (90%) mix
Adhesive voucher labels (with barcodes)
Voucher Specimen Data Sheet.

Soil Metagenomics Module (Chapter 7)

Hand trowel or small shovel	1 x large calico bag
9 x small calico bags	Adhesive voucher labels (with barcodes)
9 x medium zip lock bags	Soil Metagenomics Data Sheet.
Silica granules: self indicating (10%) + standard (90%)	

Digital Canopy Cover Module (Chapter 8)

Digital Camera	Tripod
Fisheye Lens	Coordinate Cards (0,0 to 100,100)

Fuel Survey Module (Chapter 9)

30 m tape	Builders' ruler
Clipboard	Measuring Tape
Fuel datasheets	1x1 m PVC pipe quadrat
Digital Camera	Paper bags

Compass	Secateurs
Riggers' gloves	Green supermarket bags
300 g Pesola spring balance	2.5 kg Pesola spring balance

Soil Sampling Module (Chapter 10)

Soil corer	Calico soil bags
Mallet	Permanent marker

Litterfall Trap Installation Module (Chapter 11)

4 x pieces shadecloth (1.8 x 1.8 m)	16 x 32 mm PVC 'T' joins
16 x 57 cm lengths 32 mm PVC pipe	16 x 32 mm PVC right angle elbow joins
16 x 6.8 cm lengths 32 mm PVC pipe	PVC adhesive cement
16 x 47 cm lengths 32 mm PVC pipe	16 x 7 mm tent pegs

Temperature and Humidity Datalogger Installation Module (Chapter 12)

2 x Thermochron DS1922L	Tie wire
1 x Hygrochron DS1923	Electrical tape
Thermodata software	Scissors

Litter Decomposition Bag Installation Module (Chapter 13)

21 x Litter decomposition bags	Balance
6 x Unbleached organic cotton calico pieces (10 x 10 cm)	Sewing machine
	21 x aluminium identification tags

3. Plot Layout and Positioning

The general plot location is determined before going into the field and is dependent on the hypotheses and research questions that motivate the establishment of the AusPlot. This initially involves speaking to local forest ecologists and a desktop exercise using GIS layers of vegetation type (and structure) and satellite imagery, followed by a reconnaissance field trip to assess a range of identified forest stands.

This manual assumes that the plot centre or the plot corners have been accurately located and stored in a GPS or recorded on a high quality map or imagery.

3.1. General Plot Configuration

The one hectare plot is 100m x 100m and is divided up into twenty-five 20m x 20m subplots. Star pickets mark the corners of the plot and steel droppers mark the corners of subplots. Pink flagging tape is tied to all posts for visibility and yellow caps are placed on top of all posts for safety reasons. Label each cap with the correct coordinate.

Figure 2: Ausplots Forest Monitoring Network survey plot layout.

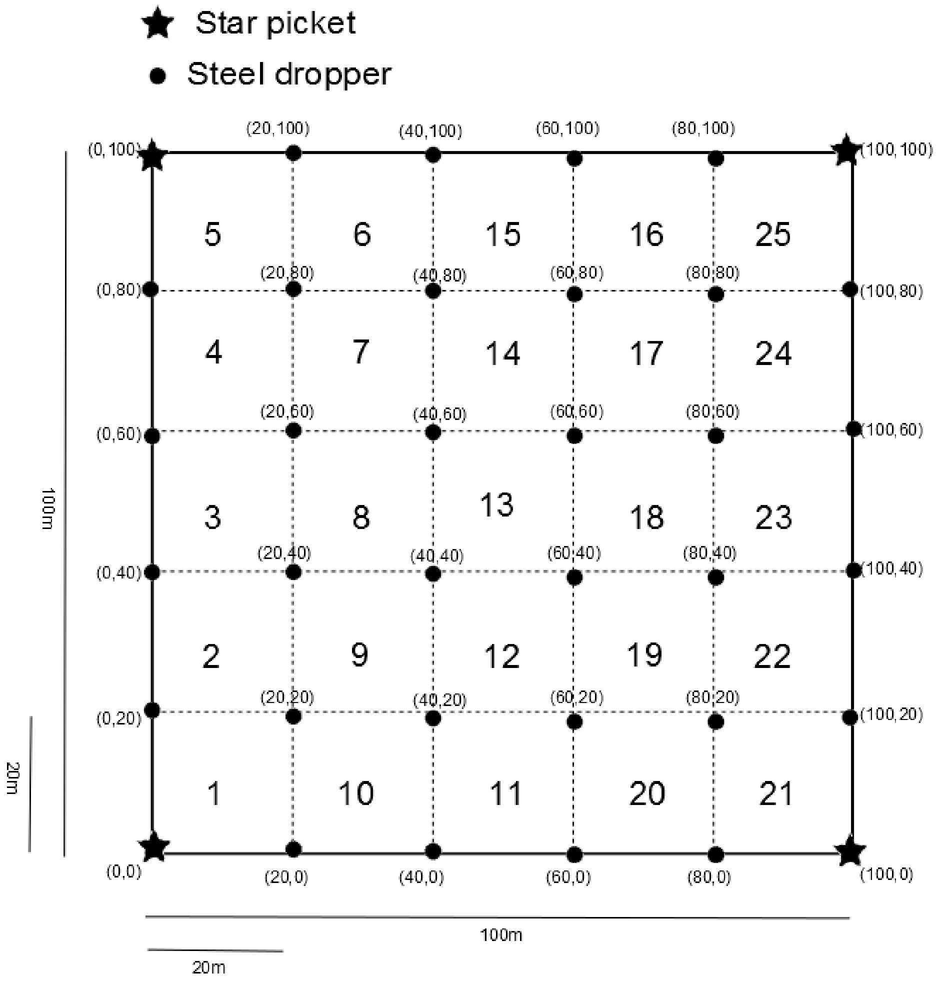

3.2. Plot Location and Configuration in the Field

Decision making regarding the placement of an AusPlot depends on whether the plot is:

- **newly established (see 3.2.1)**: the location and orientation of the plot in the field must be determined prior to plot establishment; or,
- **co-located with an existing plot (see 3.2.2)**: the orientation of the plot is predetermined. Co-locate the new plot so that the existing plot occurs in either (i) the middle or (ii) a corner of the AusPlot.

3.2.1. Location and orientation of a new plot

Guidelines

Guidance as to the approximate orientation of the 100x100m plot should be determined prior to the field campaign based on stand and topographic features identifiable from maps and satellite imagery (Figure 3). This can be facilitated by online products such as Google Earth and state-based map catalogues (e.g. ForestExplorer in Victoria and ListMAP in Tasmania). A basic map and GPS coordinates of plot corners based should be provided to the survey team.

The project leader and the field crew must determine the location and orientation of the plot in the lab and in the field such that it does not include major anthropogenic features (roads, stumps) or topographic anomalies (cliffs, major changes in slope). The location should be representative of the target vegetation type and as homogenous as possible in terms of topography, stand structure and floristics. There is no requirement to align to a N/S, E/W line on the map grid.

Procedure

Several weeks before the field campaign (preferred) or immediately before plot establishment a site-visit should be conducted to determine the origin (0,0) and bearings of baselines.

1. Starting at the (0,0) GPS coordinate provided by the pre-survey mapping, step out an *approximate* 100x100 plot using the track, mark and 'go to' features of the GPS (or a 100m tape and a compass) making careful note of the vegetation, topography and anthropogenic features in the vicinity.
2. Repeat this procedure until satisfied that the plot captures a relatively homogenous forest stand and topography with as little human disturbance as possible.
3. Based on this reconnaissance, select a location for the 0,0 corner of the 1ha plot, insert a flagged post and record the GPS position and bearings for plot baselines (e.g.0,0 to 0,100 = 40 ^{o} and 0,0 to 100,0 = 130 ^{o}).

Figure 3: Using Google Earth to identify the orientation of the 100x100m plot. The plot is 50m from the road and avoids creeklines and rainforest vegetation. The plot is in a homogenous vegetation type.

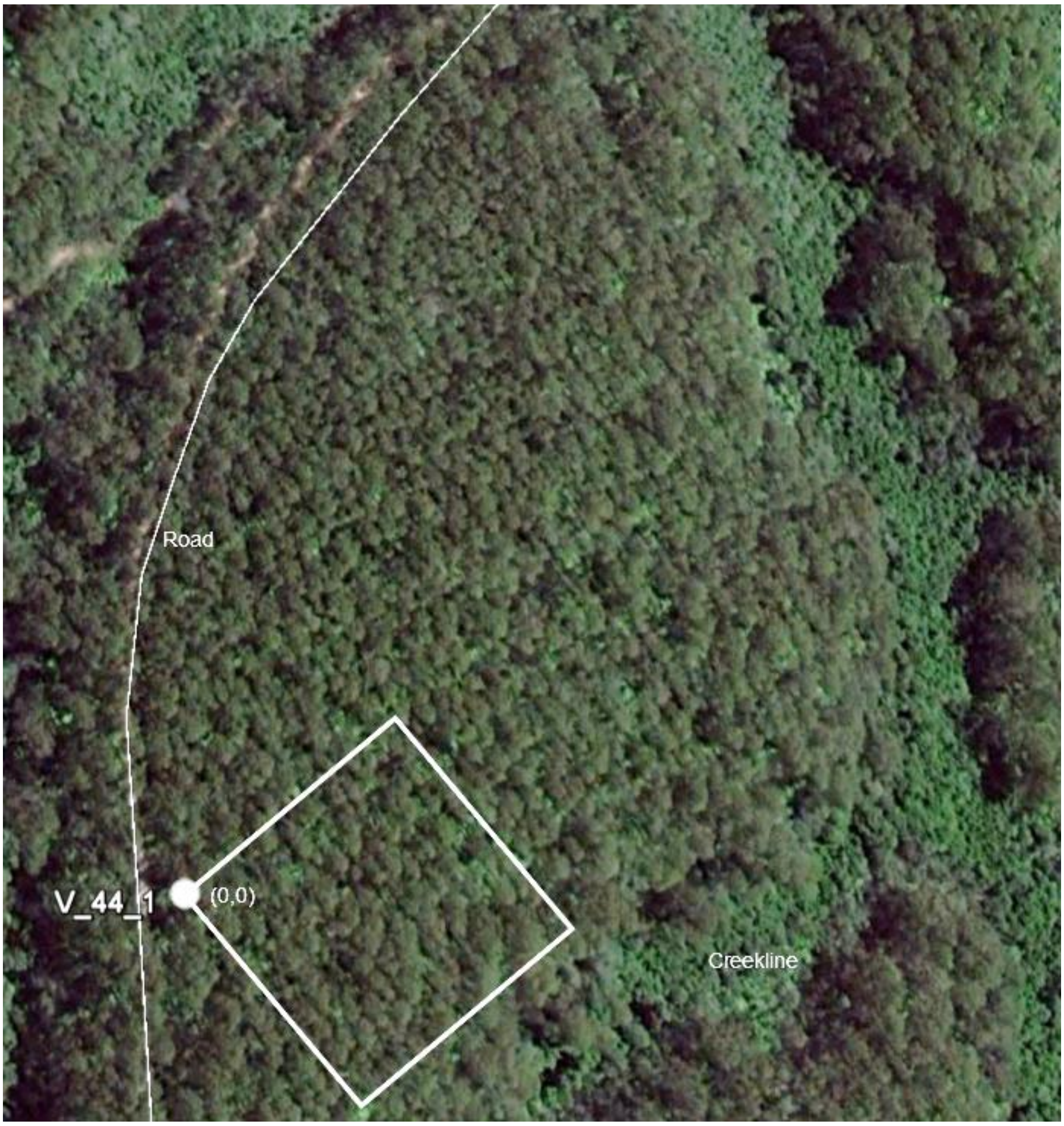

3.2.2. Location and orientation with an existing plot

Guidelines

Where possible, Ausplots Forest Monitoring Network co-locates plot infrastructure with existing monitoring plots to capitalise on historical measurements of forest dynamics. Existing plot infrastructure varies widely in shape (rectangular, square or circular) and size (0.04ha to 1ha). The management agency responsible for the existing plot should provide historical data, formal plot maps and GPS coordinates for planning purposes.

CONSIDERATIONS FOR EXISTING PLOT INFRASTRUCTURE

When co-locating Ausplots with existing plots, considerable care must be taken not to compromise the original plot infrastructure. This may not be an issue in discontinued plots, but is particularly relevant if the management agency intends to continue measurements on their plots. Permission must be sought with the management agency responsible for the existing plots (usually through the formal permit process) and this will require careful considerations about how the plots will be overlayed. The following strictures may be considered:

- *"Ausplots Forest Monitoring Network will not place any new plot infrastructure within existing plot"*
- *"Ausplots Forest Monitoring Network will not remore or modify plot infrastructure that is currently within the existing plot."*
- *"Ausplots Forest Monitoring Network will not remove or modify any markings, tree, numbers or tree tags from the trees within the existing plot."*

Procedure

Whilst the broad location of the plot is pre-determined, the configuration and orientation of the AusPlot *around* the existing plot must be determined in the field.

1) Find all corner markers (or centre markers) of the existing plot and record their GPS position.
2) Record the bearings and distances between markers for the existing plot. Check these against information provided by the management agency. These bearings will be used to inform the baselines of the AusPlot.
3) Decide on how to best co-locate the Ausplot such that the existing plot is either (i) in the (0,0) corner, (ii) in the centre or (iii) along an edge (see Figure 4). The topography, homogeneity of the stand and disturbance features should be considered in decision making.

4) Sketch the plot configuration and how it relates to the existing plot in the 'Plot Description' datasheet (see Appendices).

5) This step depends on the point of origin in relation to the existing plot:

   a. If the existing plot is located in the bottom left hand corner (e.g. Figure 4a,b), select a location for the 0,0 corner of the 1ha plot and record the GPS position and bearings for the baselines of the plot (e.g.0,0 to 0,100 = 40° and 0,0 to 100,0 = 130 ^{o}).

   b. If the existing plot is located in the centre or along the edges (e.g. Figure 4c,d) it is good practice to have the point of origin for plot construction associated with a marker within the existing plot (e.g. (60,60) in subplot 17 in 4d) to ensure that bearings and distances are consistent between plots.

Figure 4: Examples of AusPlot configurations around an existing monitoring plot.

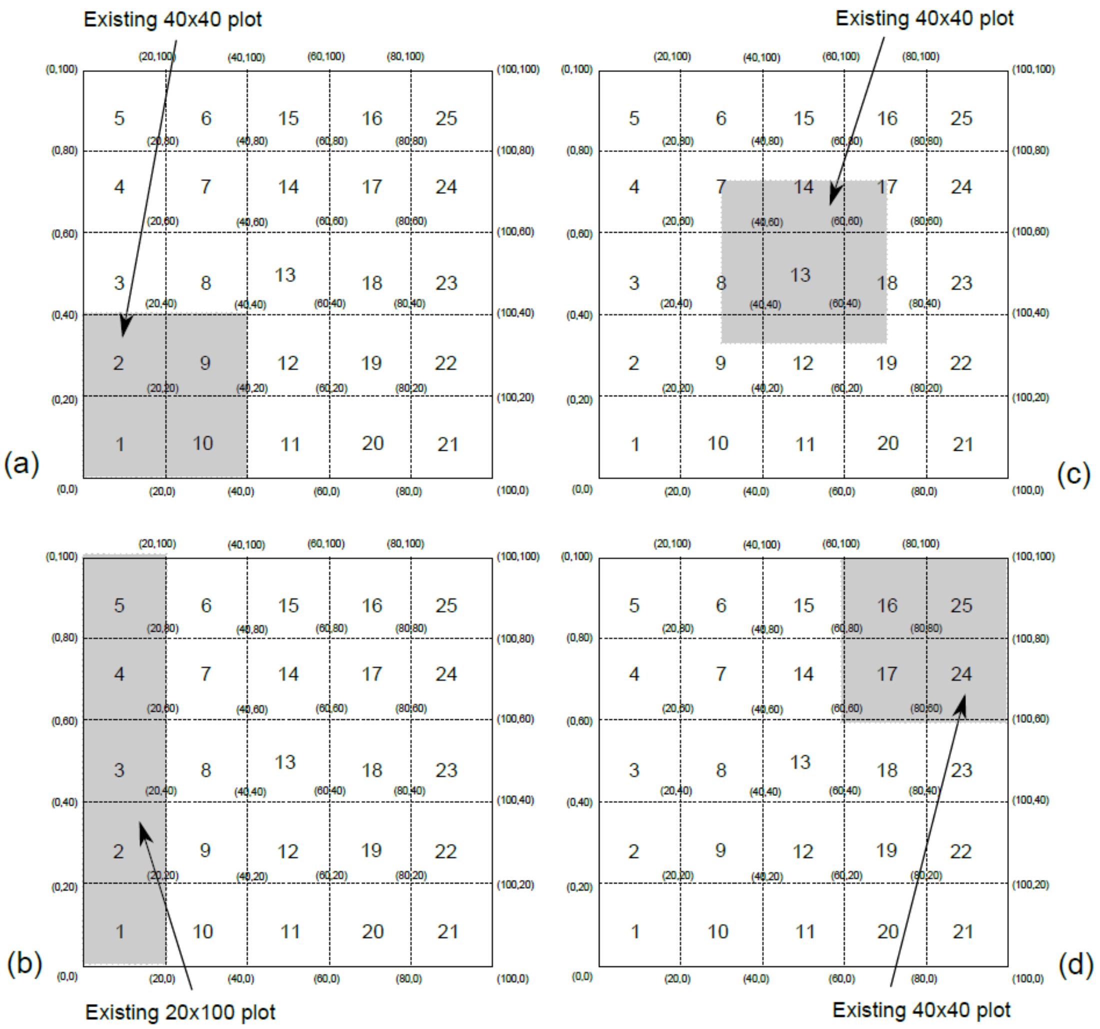

3.3. Pegging out the plot.

Guidelines

There are several methods for surveying out a plot and setting up the 100x100 grid. In woodlands and savannah forest with an open canopy and therefore good signal, a Differential Geographic Positioning System (DGPS) may be the most efficient method. In these cases, the surveyor is referred to the AusPlots Rangelands Survey Protocols Manual (White *et al.* 2012) for a worked example of a DGPS based setup. DGPS signal is generally weak or inconsistent in forests with a dense overstorey. Therefore, in closed canopy forests, survey methods involving tapes and a compass offer a practical, albeit time-consuming, alternative. In this section we outline the preferred method for surveying out a plot with a compass and tape. Other approaches can be found elsewhere (i.e. http://www.teamnetwork.org/protocols).

SLOPE CORRECTION

Ausplots Forest Monitoring Network should be set up to sample one hectare of land surface and therefore there is no need to incorporate slope corrections whilst surveying out the plot. This ensures that all Ausplots are one hectare in size on the ground thus facilitating comparisons across similar sized plots. Some protocols for other plot-based research measure a planar projection of one hectare of forest for which slope corrections need to be applied. Plots laid out in this way on steep slopes or complex topography will always tend to include a greater surface area of land surface and correction factors allowing comparison between plots on the basis of land surface need to be calculated. The lack of slope correction may have consequences for remote sensing studies based on these plots and we advise that plot size should be adjusted accordingly if such research is undertaken.

Procedure

1) Navigate to the pre-determined 0,0 corner (or the corner of an existing plot, see 3.2.2) and hammer in a star picket post. Tie flagging tape to the star picket, apply spray paint and put a yellow cap on the top.
2) Use a compass to check the bearings that will constitute the baselines of your plot (0,0 to 100,0) and (0,0 to 0,100). These bearings were recorded during plot reconnaissance (see 3.2.1. or 3.2.2).
3) Two (preferably three) people now iteratively peg out 20x20m subplots using two 20m tapes and a compass (Figure 5). One person stands at the corner post with a compass and directs the person with the tape out 20m at the relevant bearing (e.g. 45°). The tape is pulled tight, the bearing is checked, and a steel dropper is hammered into the ground. Tie flagging tape to the steel dropper, apply spray paint and put a yellow cap on the top.

4) Repeat for the next post at the 'right angle bearing' (e.g. 135°). Tie flagging tape to the star picket, apply spray paint and put a yellow cap on the top.
5) 'Close the box'. Tie 20m tapes to the (20,0) and (0,20) posts. Run each tape out at approximate bearings using the compass as a general guide. Where the two tapes meet at 20m is the correct position for the steel dropper (20,20). Double check back-bearings as errors here will be propagated through all 25 subplots. A diagonal line between (0,0) and (20,20) should be 28.3m in length and is a useful 'double check' for subplot geometry.
6) Repeat this procedure for the rest of the 25 subplots using the axes of the subplot 1 as the baselines of your survey.

Figure 5: Steps involved in setting up a subplot (from right to left): (1) run the baseline (2) right angle bearing (3) 'close the box'.

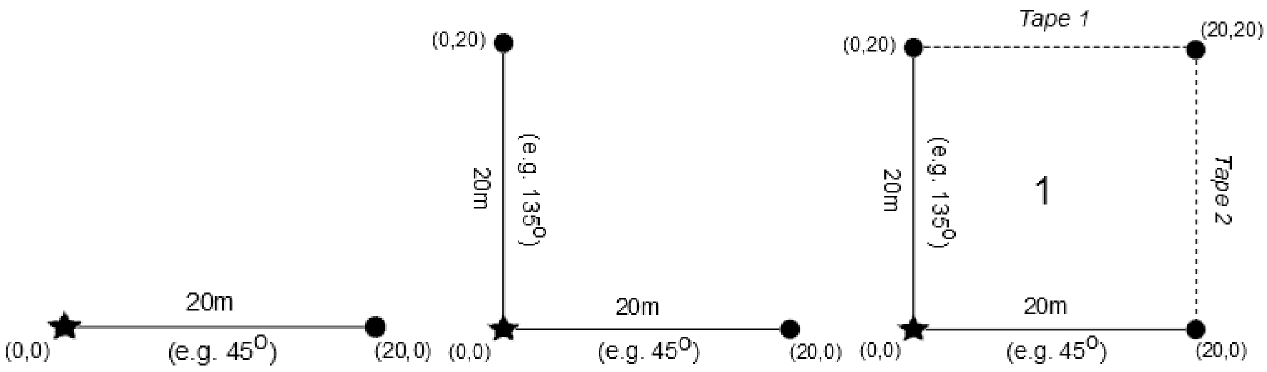

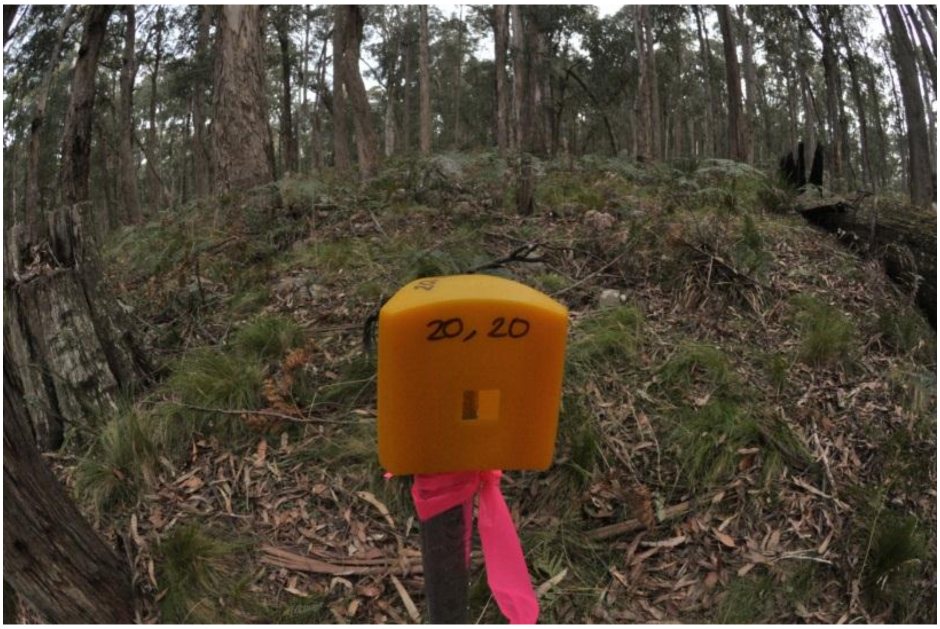

4. Site Description Module

The Site Description Module describes the site and is undertaken to (a) identify the plot and the date of measurement, (b) record location data and (c) collect observational data on terrain attributes and disturbance. This information provides basic plot metadata and will be used for contextualising forest dynamics observed through more detailed measurements of the vegetation.

Observations for the Site Description Module are collected on the Site Description Data Sheet (see Appendices). Plot identification (see 4.1), GPS coordinates and bearings (see 4.2) should be completed *after pegging out the plot* (see 3.3). Mud maps (see 4.3), terrain attributes and species lists (see 4.4) should be completed *after the large tree survey* (see Chapter 5) because the field crew will be more familiar with the plot.

4.1. Plot identification

Guidelines

Information on establishment dates, observers and plot nomenclature is collected as basic metadata for the plot. Plot identification codes follow those established for the Terrestrial Ecosystem Research Network and effectively tie in Ausplots Forest Monitoring Network infrastructure with other TERN facilities such as AusPlots Rangelands, Supersites, Transects and LTERN.

Procedure

1. **Site Name:** record the Forest Block or National Park or Reserve Name. This name will be used as a 'nickname' to familiarise the plot to the end-user.
2. **Dates of Installation:** record the dates that the plot was installed. Record as DD/MM/YYYY.
3. **Team Members**: record the first initial and last name of all people involved in data collection for the plot.
4. **AusPlotsID:** Record the 10 digit code using the following convention: State (2 letters), Plot type (1 letter), Bioregion (3 letters) Plot number (4 numbers). e.g. NSFNNC0001 translates to NSW, Forest, North Coast NSW Bioregion, Plot 1.

Figure 6. Code conventions for Ausplots Forest Monitoring Network.

State/Territory		TERN Plot Types	
Northern Territory	NT	Ausplots Forest Monitoring Network	F
South Australia	SA	AusPlots Rangelands	A
New South Wales	NS	LTERN	L
Queensland	QD	Transects	T
Western Australia	WA	Supersites	G
ACT	CT	General Use	S
Tasmania	TC	Training	TRA
Victoria	VC		

IBRA BIOREGIONS

Arnhem Coast	ARC	Furneaux	FUR	Ord Victoria Plain	OVP
Arnhem Plateau	ARP	Gascoyne	GAS	Pine Creek	PCK
Australian Alps	AUA	Gawler	GAW	Pilbara	PIL
Avon Wheatbelt	AVW	Geraldton Sandplains	GES	Pacific Subtropical Islands	PSI
Brigalow Belt North	BBN	Gulf Fall and Uplands	GFU	Riverina	RIV
Brigalow Belt South	BBS	Gibson Desert	GID	Subantarctic Islands	SAI
Ben Lomond	BEL	Great Sandy Desert	GSD	South East Coastal Plain	SCP
Broken Hill Complex	BHC	Gulf Coastal	GUC	South East Corner	SEC
Burt Plain	BRT	Gulf Plains	GUP	South Eastern Highlands	SEH
Carnarvon	CAR	Great Victoria Desert	GVD	South Eastern Queensland	SEQ
Central Arnhem	CEA	Hampton	HAM	Simpson Strzelecki Dunefields	SSD
Central Kimberley	CEK	Indian Tropical Islands	ITI	Stony Plains	STP
Central Ranges	CER	Jarrah Forest	JAF	Sturt Plateau	STU
Channel Country	CHC	Kanmantoo	KAN	Southern Volcanic Plain	SVP
Central Mackay Coast	CMC	King	KIN	Swan Coastal Plain	SWA
Coolgardie	COO	Little Sandy Desert	LSD	Sydney Basin	SYB
Cobar Peneplain	COP	MacDonnell Ranges	MAC	Tanami	TAN
Coral Sea	COS	Mallee	MAL	Tasmanian Central Highlands	TCH
Cape York Peninsula	CYP	Murray Darling Dep’n	MDD	Tiwi Cobourg	TIW
Daly Basin	DAB	Mitchell Grass Downs	MGD	Tasmanian Nth Midlands	TNM
Darwin Coastal	DAC	Mount Isa Inlier	MII	Tasmanian Nth Slopes	TNS
Dampierland	DAL	Mulga Lands	MUL	Tasmanian South East	TSE
Desert Uplands	DEU	Murchison	MUR	Tasmanian Sth Ranges	TSR
Davenport Murchison Ranges	DMR	Nandewar	NAN	Tasmanian West	TWE
Darling Riverine Plains	DRP	Naracoorte Coastal Plain	NCP	Victoria Bonaparte	VIB
Einasleigh Uplands	EIU	New England Tablelands	NET	Victorian Midlands	VIM
Esperance Plains	ESP	NSW North Coast	NNC	Warren	WAR
Eyre York Block	EYB	Northern Kimberley	NOK	Wet Tropics	WET
Finke	FIN	NSW SW Slopes	NSS	Yalgoo	YAL
Flinders Lofty Block	FLB	Nullarbor	NUL		

4.2. GPS coordinates of corner posts and compass bearings of X and Y axes

Guidelines

Ausplots Forest Monitoring Network is a long-term monitoring project and it is imperative for plots to be relocatable. This is achieved through four GPS locations recorded at each corner post. If the field crew have access to Differential GPS (DGPS) then this should be utilised in this step. However, the positional accuracy of a handheld GPS (in conjunction with stem maps and tree tags) should be sufficient to relocate plot markers in all but the densest understories.

Procedure

1. Walk to each corner post (star picket) and record the GPS coordinate (in UTM's e.g. 470985E 5229220S), its spatial accuracy and projection/coordinate system on the data sheet. It may be necessary to stand at each corner for several minutes to allow the accuracy to improve. It should be possible for accuracy better than 10m.
2. Mark the point in the GPS. Press Mark and record as AusPlotsID (e.g. NSFNNC0001) followed by the location (0,100). For example: NSFNNC0001-0,100. These are downloaded at the end of the trip.
3. Bearings are required for relocating the plot in future surveys. Stand on the 0,0 post and record the compass bearing Up the plot (0,0) to (0,100) and Across the plot (0,0) to (100,0) as per Figure 7.

Figure 7: Diagram of plot bearings to be recorded

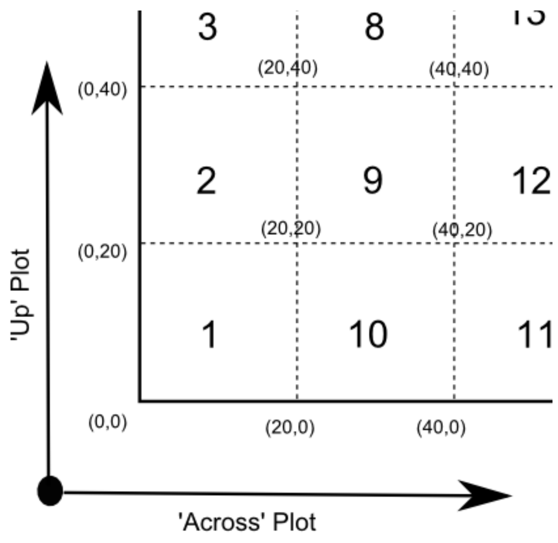

4.3. Mud Map of the Site and Plot

Guidelines

A mud map is an informal sketch of the basic features of the landscape and forest stand. These maps assist in the relocation of the plot and can assist in the interpretation of spatial patterning and stand scale processes, for example, canopy gaps may be associated with drainage features or rock outcrops. This step should be completed *after* the large tree survey so that observers are familiar with the plot and the landscape.

Procedure

1. Sketch a map showing the location of the plot in the landscape, including topographic features, roads and other information to aid navigation to the plot (e.g. Figure 8). Include GPS coordinates for important features, including the Take Off Point (where the car is parked) and equipment assembly points.
2. On the plot diagram, show key features of the plot such as large trees, logs, water bodies, rock outcrops, burned area, human disturbances and the location of existing plots (e.g. Figure 8).

Figure 8: Example of a mud map of the site and the plot.

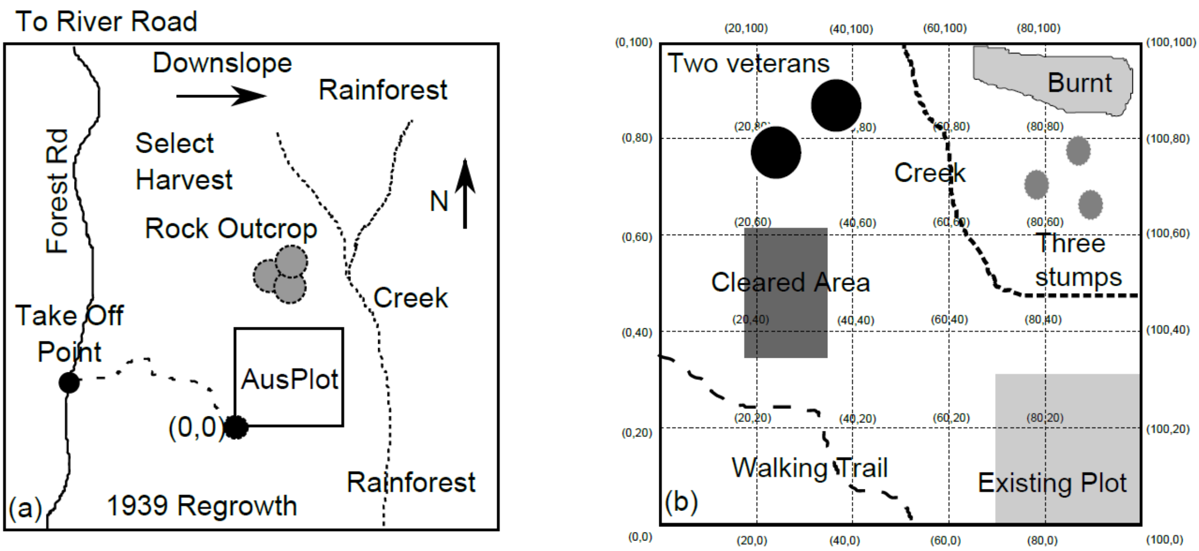

4.4. Landform of plot

Guidelines

This information provides a physical description of the landscape for baseline metadata and for comparison between plots. Landform descriptions are based on 'The Yellow Book' (McDonald *et al.* 1990). These steps should be completed *after* the large tree survey so that observers are familiar with the species, the plot layout and the landscape.

Procedure

1. **Landform pattern**: Record the landform pattern within a large circle of 500m radius using Table 2.
2. **Landform element**: Record the landform situation within a small circle with a radius of 100m using Figure 9
3. **Slope (Class):** Record the slope class using the codes in Table 3.
4. **Slope (Degrees)**: Record the slope of the plot using the clinometer.
5. **Aspect**: Record the compass direction, in degrees, of the main downward slope of the plot
6. **Lithology:** Record the rock type in the field if known according to Table 4. This information can be obtained from geology maps in the lab if necessary.
7. **Disturbance Measures**: provide a qualitative assessment of disturbance, which is done over the whole plot area according to Table 5.

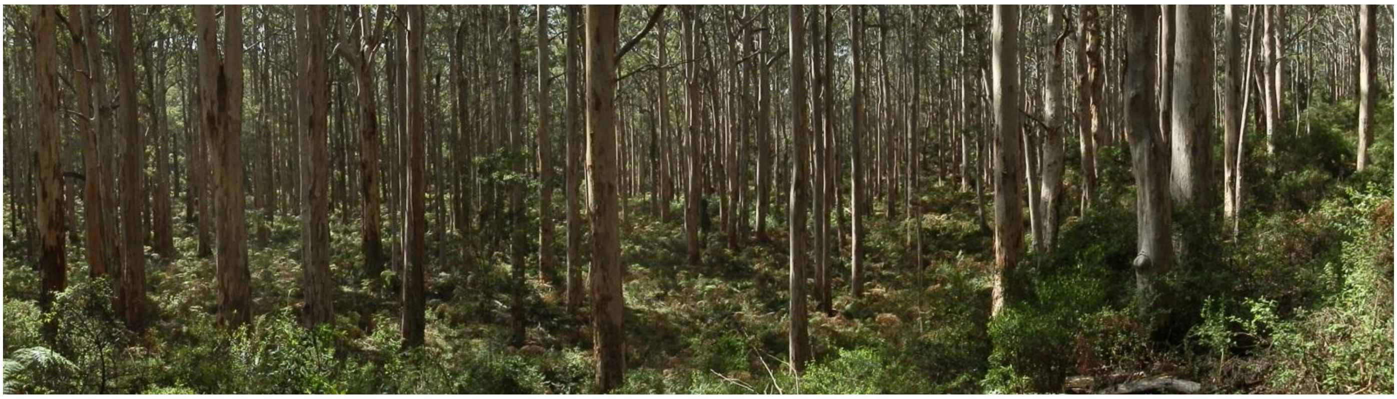

Table 2: Landform Pattern Codes

Description	CODE	Description	CODE
Alluvial fan	ALF	Made land	MAD
Allubial plain	ALP	Marine plain	MAR
Anastamotic plain	ANA	Meander plain	MEA
Badlands	BAD	Meteor crater	MET
Bar plain	BAR	Mountains	MOU
Beach ridge plain	BEA	Parabolic dunefield	PAR
Caldera	CAL	Pediment	PED
Chenier plain	CHE	Pediplain	PEP
Coral reef	COR	Peneplain	PNP
Covered Plain	COV	Plain	PLA
Delta	DEL	Plateau	PLT
Dunefield	DUN	Playa plain	PLY
Escarpment	ESC	Rises	RIS
Flood plain	FLO	Sand plain	SAN
Hills	HIL	Sheet flood fan	SHF
Karst	KAR	Stagant alluvial plain	STA
Lacustrine plain	LAC	Terrace	TER
Lava	LAV	Terraced land	TEL
Longitudinal dunefield	LON	Tidal flat	TID
Low hills	LOW	Volcano	VOL

Table 3: Slope Classes

Slope Class	Slope(^{o})	Slope (%)	Code
Level	0-1°	0.6	LE
Very gently inclined	1°	1	VG
Gently inclined	3°	6	GE
Moderately inclined	10°	20	MO
Steep	23°	40	ST
Very steep	37°	70	VS
Precipitous	60°	170	PR
Cliff	80°	500	CL

Figure 9: Landform elements

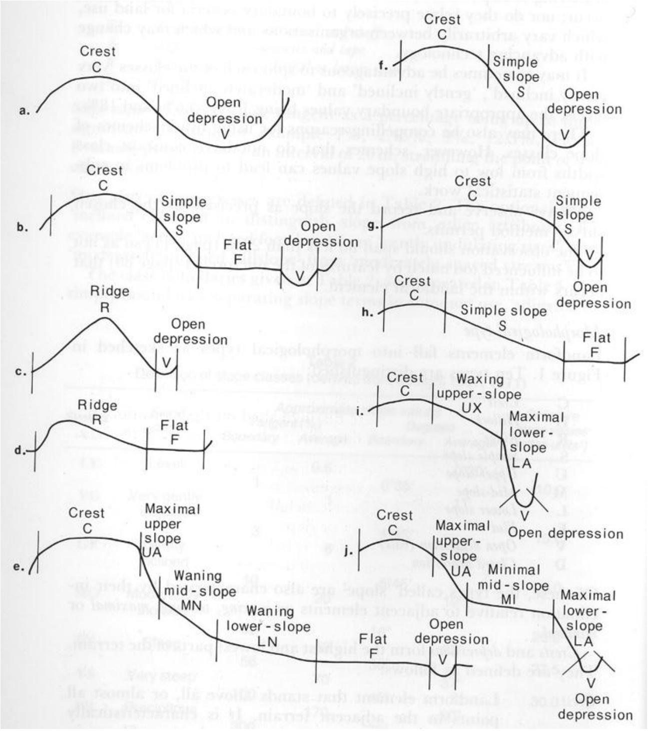

Table 4: Lithological type. Adapted from Wood *et al.* 2006

Code	Name	Genetic Type *	Code	Name	Genetic Type *
AD	Adamellite	Ig	JA	Jasper	Sc
AG	Agglomerate	Sp	LI	Limestone	Sd
AC	Alcrete (bauxite)	Sc	MB	Marble	Me
AM	Amphibolite	Me	ML	Marl	Uc
AN	Andesite	Ig	ME	Metamorphic rock (unidentified)	
AH	Anhydrite	Sc			
AP	Aplite	Ig	MD	Microdiorate	Lg
AR	Arkose	Sd	MG	Microgranite	Ig
AF	Ash (fine)	Uc	MS	Microsyenite	Ig
AS	Ash (sandy)	Uc	MI	Migmatite	Me
BA	Basalt	Ig	MU	Mudstone	Sd
BB	Bombs (volcanic)	Uc	MY	Mylonite	Me
BR	Breccia	Sd	PG	Pegmatite	Ig
KA	Calcarenite	Sd	PE	Peridotite	Ig
KM	Calcareous mudstone	Sd	PL	Phonolite	Ig
KS	Calcareous sand	Uc	PH	Phyllite	Me
KL	Calcilutite	Sd	PC	Porcellanite	Sc
KR	Calcirudite	Sd	PO	Porphyry	Ig
KC	Calcrete	Sc	PY	Pyroxenite	Ig
CH	Chert	Sc	QZ	Quartz	Ig
C	Clay	Uc	QU	Quartzite	Me
CO	Coal	Sc	QP	Quartz porphyry	Ig
CG	Conglomerate	Sd	QS	Quartz sandstone	Sd
CU	Consolidated rock (unidentified)		RB	Red-brown hardpan	Sc
			RH	Rhyolite	Ig
SD	Detrital sedimentary rock (unidentified)		S	Sand	Uc
			SA	Sandstone	Sd
DI	Diorite	Ig	ST	Schist	Me
DR	Dolerite	Ig	SK	Scoria	Uc
DM	Dolomite	Sd	SR	Serpentinite	Ig
FC	Ferricrete	Sc	SH	Shale	Ig
GA	Gabbro	Ig	LC	Silcrete	Sc
GS	Gneiss	Me	Z	Silt	Sd
GN	Granite	Ig	ZS	Siltstone	Uc
GD	Granodiorite	Ig	SL	Slate	Sd
GR	Granulite	Me	SY	Syenite	Me
GV	Gravel	Uc	TR	Trachyte	Ig
GW	Graywacke	Sd	TU	Tuff	Ig
GE	Greenstone	Me	UC	Unconsolidated material (unidentified)	
GY	Gypsum	Sc			
HA	Halite	Sc	VB	Volcanic breccia	Sp
HO	Hornfels	Me	VG	Volcanic glass	Ig
IG	Igneous rock (unidentified)				

). *Genetic types: Ig – Igneous rocks; Me – Metamorphic rocks; Sd – Sedimentary rocks, detrital; Sp – Sedimentary rocks, pyroclastic; Sc – Sedimentary rocks, chemical or organic; Uc – Unconsolidated material.

Table 5. Disturbance measures. Adapted from Wood *et al.* 2006.

**Wildfire**	Refers to major previous hot fire disturbance, the severity of which can be based on the extent of fire scars on standing trees relative to their height and diameter. Time since such an event can be estimated on the height of any post-burn regeneration, charring on ground woody material which may have fallen since the event, diameter growth around fire scars on standing live trees, extent of crown recovery. Record also the mean height of fire scars on standing stems. Severity 1 = minor scorching on logs and lower trunk; 2 = lack of understorey and scorching up to 2m; 3 = wild fire scorching greater than 5m height and or crown damage
**Prescribed Burn**	Refers to the cooler, perennial burns used to reduce fuel loads and/or increase grazing potential of the grassy understorey. The periodic nature of these burns dictate that the intensity of this disturbance would rarely be recorded as severe.
**Logging**	Record information on past logging events. Severity should be the total of all logging events and time for the latest. If there have been several logging events record details in the notes section.
**Treatment**	Treatment is defined as the destruction of individual trees by ringbarking or poisoning, in contrast to 'logging' of individual trees for product harvesting and 'clearing' by mechanical means. Dead and fallen trees should be examined closely for marks indicating past treatment. These can be at waist height (ringbarking or tomahawk cuts) or near ground level for basal injection treatment.
**Grazing**	Grazing impact can be assessed by the presence of manure, compaction and stock trails. It will probably not be possible to estimate grazing severity for older grazing events. However inspection of fencing and stock infrastructure in the vicinity may give some indication of the time grazing has been conducted on an area.
**Clearing**	Record information for perceived mechanical clearing events.
**Weeds**	Record for current weed infestations. Weeds are defined as exotic species declared or assumed to be noxious (eg lantana, balloon cotton bush), but not native 'woody weeds' such as Dodonaea. Any of the latter resulting from disturbance is recorded as regeneration.
**Erosion**	Record information on erosion seen in the plot, eg. Gully erosion. Erosion outside the plot but in the vicinity should be noted with approx distance from plot.
**Mining/quarries**	Record information on any activity seen in the plot. Activity outside but in the vicinity should also be noted.
**Storm**	Record information on evident storm damage. This is characterised by broken off stems and excessive uprooting in the one direction.
**Salinity**	Record evidence of salinity affecting trees or vegetation
**Other**	Specify any other disturbance types noted eg. Dieback, soil disturbance, snig tracks.
**Regeneration**	Record information about regeneration resulting from disturbance eg wattle following wildfire or regrowth following clearing. Detail in notes as required.

4.5. Species List and Understorey Description

Guidelines

During the large tree survey, tree species are recorded according to a six letter code (e.g. EUCREG for *Eucalyptus regnans*). This section provides the necessary documentation of the link between the code and the species. A qualitative description of the understorey is provided to contextualise the plot and aid in planning for subsequent quantitative measures of this important forest component.

Procedure

1. **Large Tree Survey Species List**: *After the tree survey*, list the genus and species of all trees recorded in the survey and the six letter code used to identify them on the Large Tree Survey Data Sheet. Use consistent taxonomy across jurisdictions following published names in APNI (Australian Plant Names Index at http://www.cpbr.gov.au/databases/apni-about/index.html.). A list of possible species should be provided to the field crew prior to the trip as a guide. Also record any notes on trees that could not be identified, for example, the 'best guess' for genus and species of SPP001, SPP002 SPP003 (etc.), the voucher specimen number or the photo ID.
2. **Description of Understorey**: Provide a brief qualitative description of the floristic and structural nature of the understorey. Include information on the dominant guild (e.g. sclerophyll, fern, grass, rainforest), dominant species, approximate cover and height and stratum. This information provides useful context when interpreting the dynamics of the overstorey trees. Use subplots to detail spatial distribution of notable vegetation communities.
3. **Notes:** Document any problems or important information that would help in the interpretation of results and data.

5. Large Tree Survey Module

The purpose of this module is to obtain information on the species, diameter, height, spatial location and general characteristics of each tree ≥10cm in the 100 x 100m plot (Figure 10). This information will be used for monitoring changes in tree growth, basal area growth, biomass tree mortality and recruitment, and floristic information for measures of tree diversity.

This module forms the basic framework of the Ausplots Forest Monitoring Network plot network and comprises the minimum dataset for Ausplots Forest Monitoring Network measurements.

The protocols and coding used in this module are drawn directly from international forest inventory protocols (e.g. RAINFOR, TEAM, GEM and TROBIT) and are consistent with most other Australian forest inventory methodologies (e.g. CFMF, State Permanent Growth Plots). This will allow seamless comparisons between different forest types in Australia and facilitate participation in global meta-analyses of forest biomass, forest growth and forest dynamics such as mortality and recruitment.

The following measurements within each 20x20m subplot are recorded on the Large Tree Survey Data Sheet (Appendices):

- Stem ID number: Tag each stem at 1.6m (or 30cm above Point of measurement) and record the ID of the tag;
- Genus and Species. Six letter codes used i.e. *Eucalyptus regnans* is EUCREG;
- Diameter at Breast Height (1.3m), unless a problem tree;
- Point of Measurement (POM), if different from 1.3m;
- X and Y coordinates from the bottom left hand corner of the subplot;
- Alive Status;
- Growth Stage;
- Mode of Death, if dead.

A measurement team of three people is most efficient: one to scribe, one to measure diameters, and one to tag and paint each tree. The most efficient work flow is shown in Figure 11.

In the field, this module is most efficiently completed if broken up into 20x20m subplots. In dense forests, each subplot can be further divided in a negative and positive side (see Box "Moving through the subplot").

Figure 10: Example of data derived from Large Tree Survey. Each species is represented with a different colour and each individual is plotted in X,Y space. The width of each cylinder corresponds to tree diameter and the height of each cylinder represents measured tree height.

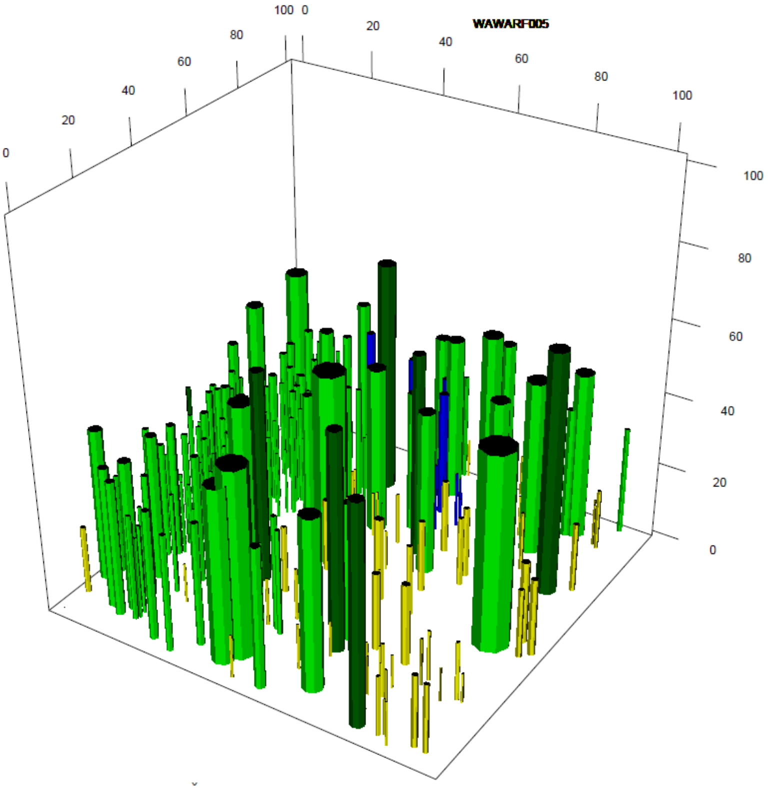

MOVING THROUGH THE SUBPLOT

In the field, teams should move through the subplots starting at subplot 1 and ending at subplot 25. In dense undergrowth typical of some forests it is difficult to see further than ten meters. As such, the most efficient method of collecting data in each subplot is to divide the plot into a negative (–ve) and positive (+ve) side by running a 20m tape through the centre of the plot. The –ve side is always measured first, followed by the +ve side.

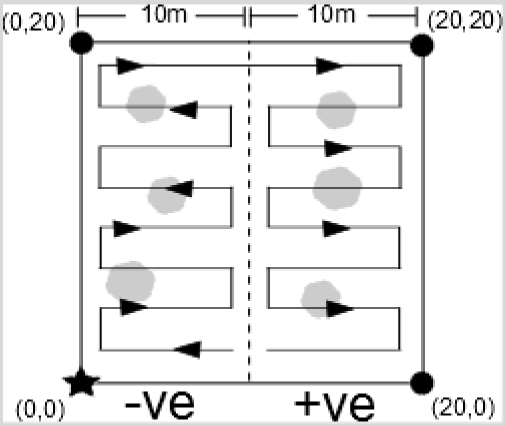

Figure 11: Work Flow for Large Tree Survey

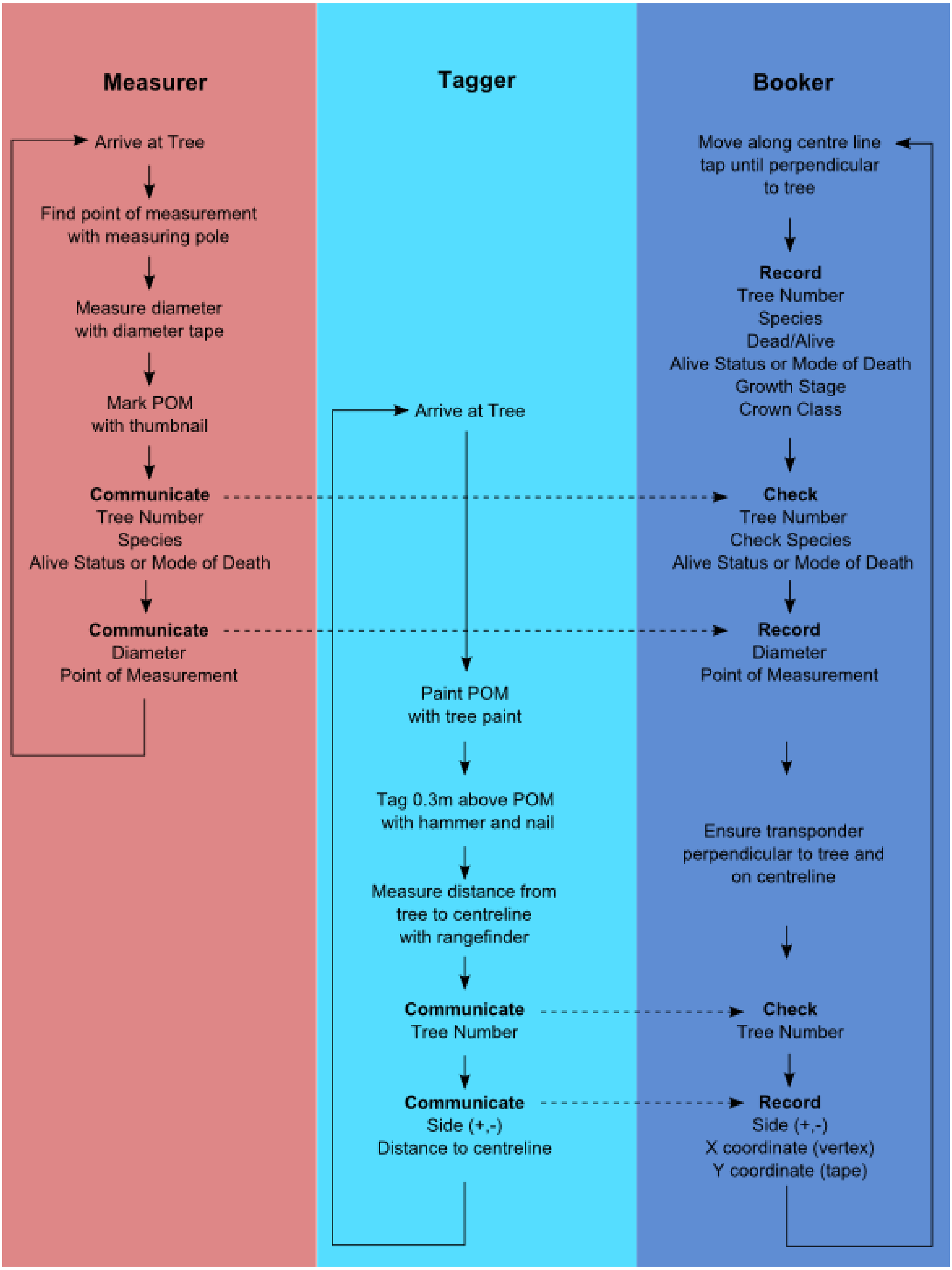

5.1. Plot information

Procedure

1. **Date:** record the date of measurement as DD/MM/YYYY
2. **Site Code**: Record the site code according to Ausplots Conventions (i.e. NSFNNC001)
3. **Site Name:** and site 'nickname' based on the forest block, the national park or the reserve.
4. **Subplot:** Record the subplot currently being measured (i.e. 1, 2, 3…..25).
5. **Start-Time, End Time** (optional): record the start and end time of the subplot measurement. This information is used for planning purposes.
6. **Measurers**: record the scribe (s=HS), the tagger (t=ER) and the measurer (m=CO).

5.2. Tree ID, Tree Status (Dead/Alive) and Species

Guidelines

Each live tree has a sequential numeric identifier starting at 1 near the (0,0) corner and the final tree (e.g. 999) in the (100,100) corner. Trees with a smaller number are in subplot 1 and the highest numbered trees are in subplot 25. Importantly, dead trees in the initial survey are given an identifier of 'D' and tree ferns are denoted 'DA' or 'CA' (i.e. *Dicksonia antarctica or Cyathea australis*)

When co-locating an AusPlot with an existing plot be wary of the numbering system already in place and ensure that there is no duplication of tree numbers. For example, if the existing plot has already tagged trees as 1-187, consider starting AusPlots tags from 300-999 to distinguish the two labelling systems and avoid duplication.

A tree is measured if it is ≥10cm diameter at 1.3m and more than 50% of the base of the trunk is within the subplot (Figure 12). The tree number is recorded on the datasheet and subsequent details of the tree are measured and recorded (e.g. diameter, growth stage, crown class).

Each live tree is assigned a genus and species. Before the trip, a potential species list should be prepared for each plot based on published species lists, herbaria records, past surveys and local knowledge. Use consistent taxonomy across jurisdictions following published names in APNI (Australian Plant Names Index at http://www.cpbr.gov.au/databases/apni-about/index.html.).

Procedure

1. Record the **TreeID** for each tree ≥10cm DBH inside the subplot. Live trees are given a sequential number 1-999. Dead trees in the initial survey are recorded as 'D' and tree ferns are recorded as 'DA' (i.e. *Dicksonia antarctica*)
2. Record the **tree status** as either dead (D), Alive (A) or Resprouting (R). Resprouting trees have a dead trunk, but have coppicing live foliage sprouting from the base.
3. Record the **genus and species** of each tree ≥10cm DBH using a six letter code with three letters from the genus and three letters from the species (i.e. *Eucalyptus regnans* is EUCREG).
4. Record the species and six letter code in the Species List section of the Plot Establishment Data Sheet.
5. For trees that cannot be identified, take a photo and a well labelled voucher specimen for identification at a relevant Herbarium. This can be included in the voucher specimens outlined in Section 6 of this manual. Record unidentified trees on the Datasheet as SPP001, SPP002, SPP003 etc. and make a note at the end of the Plot Establishment Data Sheet (see Appendices).

Figure 12: Rule set for deciding whether a tree is included in a plot.

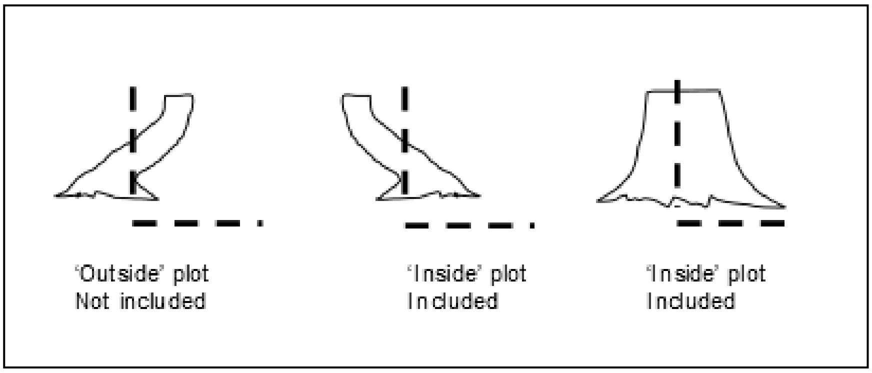

5.3. Tree Diameter and Point of Measurement

Guidelines

The diameter of trees is the standard forest inventory measurement that is used to calculate fundamental forest metrics such as growth rates, basal area, volume and biomass. This information constitutes the prime focus of the Ausplots Forest Monitoring Network survey. A cut off size of diameter 10cm at breast height is a global forest ecology standard for defining 'a tree' and the solutions to problem trees are consistent with other international and Australian forest inventory protocols.

Procedure

1. Record the **diameter of each tree** ≥10cm at 1.3m, unless they are a 'problem tree' (i.e. leaning, on a slope, buttressed etc. see Solutions to Problem Trees Box). Use a measuring pole pushed firmly into the leaf litter to define 1.3m. Pull the diameter tape around the tree trunk such that it is perpendicular to the main axis of the tree trunk (i.e. not parallel to the ground). Clear any moss, loose bark or anything else that might distort the diameter tape.
2. Record the **point of measurement** as 1.3m unless they are a 'problem tree', for which a different point of measurement will be necessary.

Solutions to Problem Trees

The standard diameter measurement height for AusPlots is 1.3m. Exceptions to this rule are as follows:

Buttressed Trees: Buttressed trees are a significant source of error in repeat tree measurements and require careful attention in the field. Buttressed trees are measured several times.

(1) at 1.3m

(2) at the highest point you can reach (e.g. ~2.2m). Record measurement in the 'Comments' column of the datasheet using the following notation: *"DBH 136.3cm @ POM 2.2m"*.

(3) 50cm above the top of the buttress. On some trees this may require the use of a ladder. This step can be time consuming and is generally conducted on a subsequent plot visit. Record measurement in the 'Comments' column of the datasheet using the following notation: *"DBH 122.3cm @ POM 5.2m"*.

Every effort must be made for (1) and (2) in the initial survey. Buttressed trees receive a 'X' code in the Alive Status (see section 5.6) to flag stems that require more detailed measurements above the buttress.

Multi-Stemmed Trees: These are treated as single trees (e.g. Tree 118) with multiple stems (Stem A, Stem B, Stem C) and are labelled with the following ID's: 118A, 118B, 118C. Label in order of size and tag Stem A. The spatial arrangement of each branch should be carefully noted in the X and Y coordinates. Multiple stemmed trees are flagged with an 'H' code in the Alive Status (see 5.6).

Trees on a slope: if a tree is on a slope, then 1.3m should be measured on the uphill side of the tree.

Leaning trees: should be measured on the inside of the lean, starting at the ground next to the base of the tree. Leaning trees are flagged with a 'C' code in the Alive Status (see 5.6).

Deformed trees. Should be measured *either* above or below 1.3m and the point of measurement recorded.

Dead Trees in the initial survey: Dead trees in the initial baseline survey are assigned a TreeID of **'D'** and all other attributes are measured as normal. Dead trees are not tagged.

Tree Ferns: the soft texture of tree ferns trunks are not conducive to DBH measurements and therefore tree ferns are not measured for diameter. Instead, an X and Y coordinate is provided for a stem map and they are labelled with a generic TreeID of **'DA' or 'CA'** (i.e. *Dicksonia antarctica or Cyathea australis*). Record heights of each Tree Fern in the Height column.

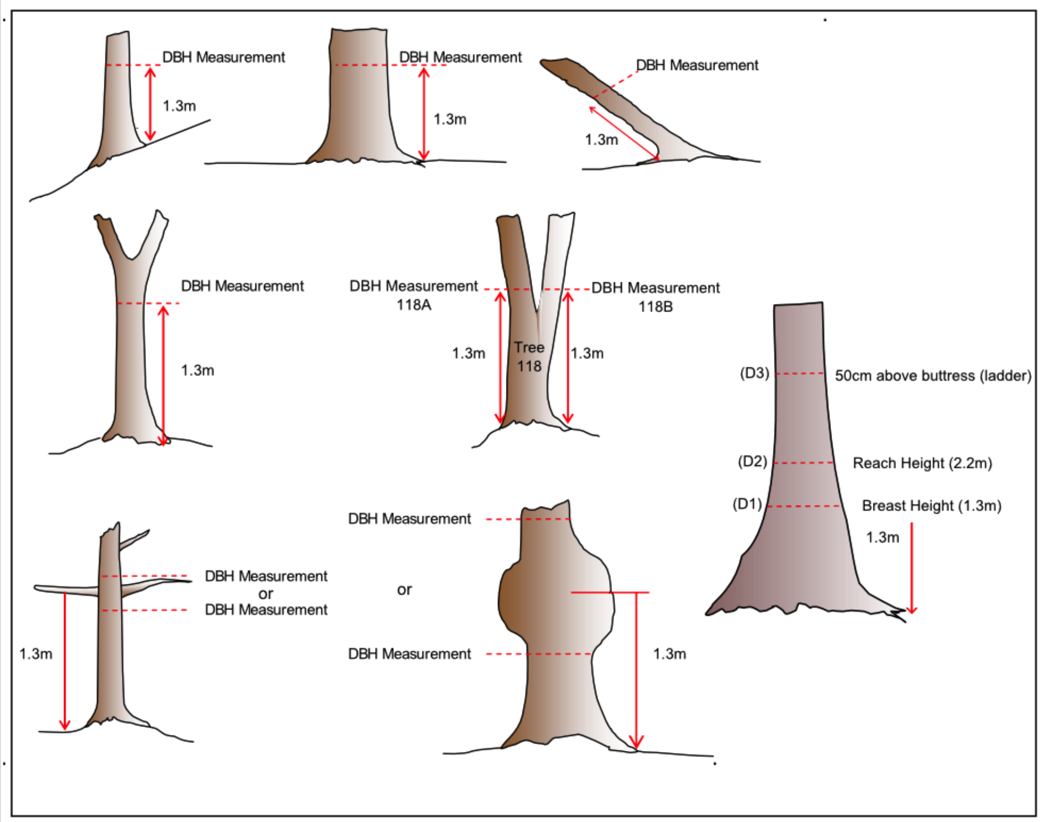

5.4. X and Y coordinates

Guidelines

The X and Y coordinates of each tree ≥10cm DBH needs to be recorded to generate a spatial map of the location of each tree in the 100x100m plot (e.g. Figure 13). This information will be used for (a) relocation of trees in the subsequent measurements and (b) for spatial analyses of tree competition, gap dynamics and facilitation.

Ausplots Forest Monitoring Network records X,Y coordinates within each of the twenty five 20x20m subplots which are later converted to the 100m x 100m X,Y grid in the laboratory (see Figure 13). Other methodologies include recording bearings and distances from a known location (such as the corner post) but this is difficult in dense understories.

Figure 13: Example of a stem map for a 100x100m Ausplot.

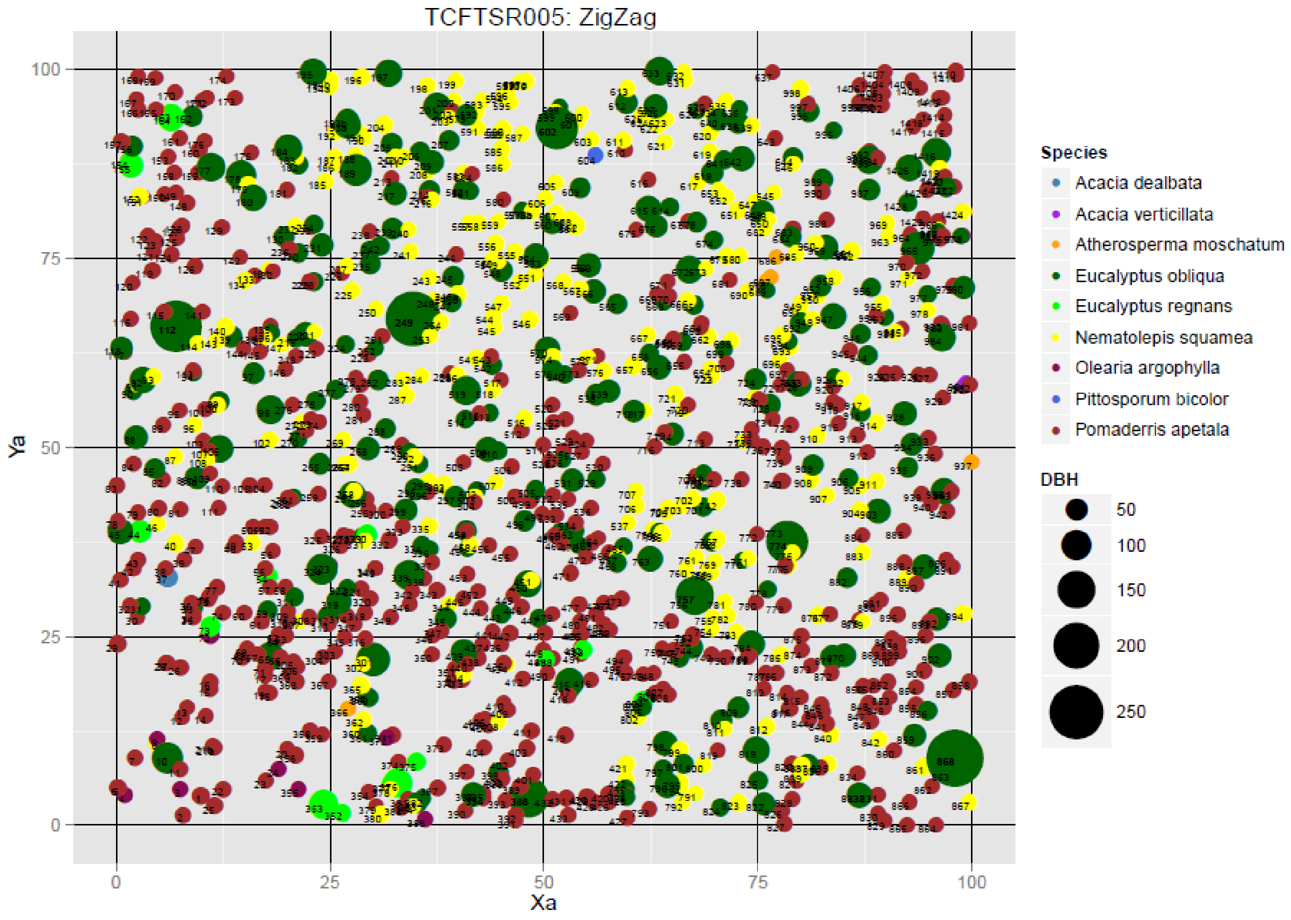

Procedure

1. Run a 20m tape up the middle of the subplot (see Box "Moving through the subplot").
2. **Side:** record whether the current measurement is on the negative (–ve) or positive (+ve) side of the subplot (see Figure 14).
3. **Y coordinate** record the distance along the taped centre line.
4. **X coordinate** record the distance between the tree and the centre-line using a Vertex Hypsometer (Figure 14). The transponder is attached to the scribe who stands perpendicular to the tree on the centre line. The tagger stands next to the centre of the tree and measures the distance with the vertex by holding down the '>' button until the vertex beeps and shows the measurement on the screen. The vertex is reset by holding the '<' and '>' (i.e. Off) button down at the same time. Alternatively, a 10m tape can be used to determine the X coordinate.

Figure 14: Procedure for recording the X,Y coordinates of a tree within a 20x20m subplot.

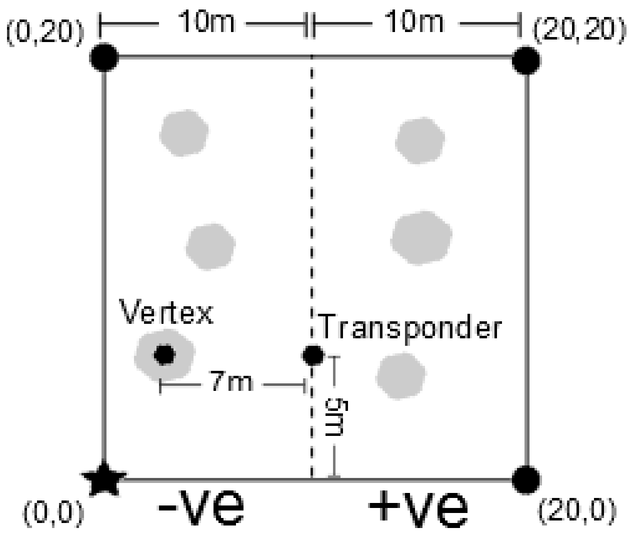

VERTEX HYPSOMETER

The Vertex Hypsometer is a rangefinding instrument for measuring height, distance and horizontal distance in the field. The instrument uses an ultrasonic measuring system has a distinct advantage over other laser-based instruments because it can effectively 'see around' obstacles such as branches and tree stems. A transponder is fastened to the tree to be measured and the distance to the instrument is calculated. A height function can be used to calculate tree height using horizontal distance and angle to the transponder.

For the best results, the Vertex Hypsometer should be calibrated regularly during the field campaign.

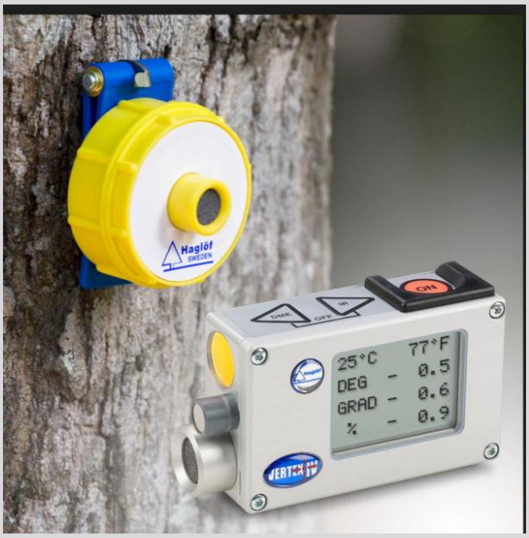

5.5. Tree Growth Stage and Crown Class

Guidelines

The growth of trees in a forest stand will depend upon the age of the tree and its position in the canopy. In general, young regrowth trees will have fast growth rates compared to mature and senescent trees. The canopy position partly determines the resources available to the tree and therefore their overall productivity: a dominant tree receiving full sunlight may have higher growth rates than a supressed tree in the full shade of other trees. Information collected here will be used to help interpret growth patterns over time.

Procedure

The growth stage (i.e. approximate age) and crown class (i.e. canopy position) are subjectively observed in the field according to standard forestry codes.

1. Record the growth stage of the tree according to codes in Table 6 and Figure 15.
2. Record the crown class of the tree according to codes in Table 7 and Figure 16.

Table 6: Growth Stage Codes and Descriptions

Code	Stage	Description
**R**	Regeneration	Juvenile and sapling stages where tree is very small and crown exhibits apical dominance – approx. 0-20 years
**Y**	Regrowth	Tree has a well developed stem (pole) with a crown of small branches, below maximum height for a stand, apical dominance apparent in vigorous trees – approx.. 20-30 years
**M**	Mature Phase	Tree has reached maximum height and crown has reached full lateral development although branch thickening can occur. Apical dominance lost – approx. 30-80 years
**O**	Senescence Phase	Crown form contracting and becoming 'stag headed' decrease in crown diameter and crown leaf area. Distorted branches and burls common – approx. >80 years
**S1**	Dead	Tertiary branches are still present, bark may still be present
**S2**	Dead	Tertiary branches are largely missing, bark is absent or, if present, is very loose and falling away.
**S3**	Dead	No crown structure remains, bark is absent.

Figure 15: Growth Stage Codes and Diagrams

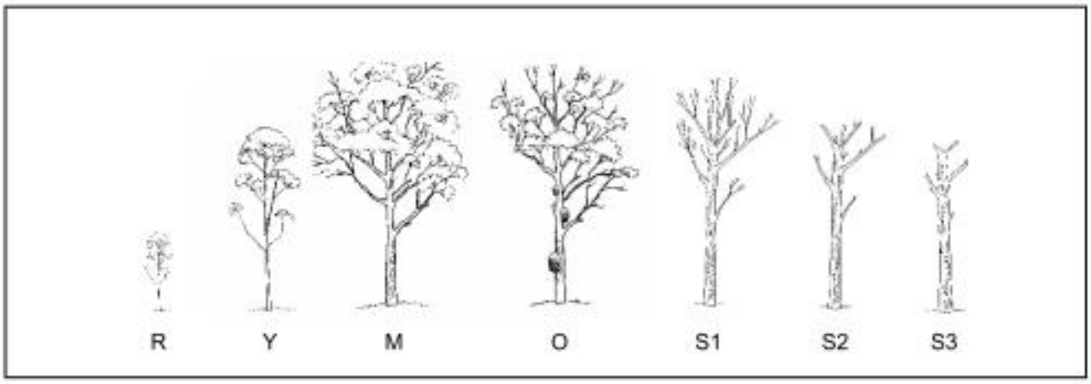

Table 7: Crown Class Codes and Descriptions

Code	Crown Class	Description
**D**	Dominant	Trees with well-developed crowns extending above the general level of the forest canopy. The crown receives full sunlight from above and partly from the sides
**C**	Co-dominant	Trees with medium sized crown forming the general level of the forest canopy. Each tree receives full sunlight from above but very little from sides
**I**	Intermediate	Trees shorter than dominant and co-dominant trees and have small crowns extending into the forest canopy. Each tree receives a little direct light from holes in the canopy and very little light from the sides
**S**	Suppressed	Trees with crowns more or less entirely below the forest canopy and receiving very little direct light either from above or from the sides
**E**	Emergent	Trees with crowns totally above the canopy of the stand and receiving full sunlight from both above and from all sides
**OG**	Open grown	Trees not growing near any other tree and with crowns receiving full sunlight from both above and from all sides.

Figure 16: Growth Stage Codes and Descriptions

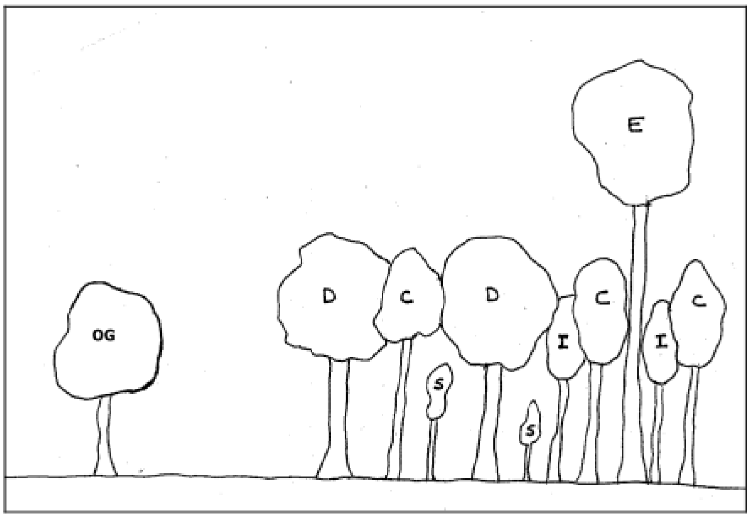

5.6. Tree Condition and Mode of Death.

Guidelines

The characteristics of the tree bole and canopy can be useful for the interpretation of growth trends and assist in the relocation of trees in subsequent surveys.

As AusPlots are remeasured over time, some trees will senesce and die. Understanding trends in mortality and how they relate to external factors like climate and disturbance is central to the aims of Ausplots Forest Monitoring Network. Mode of death codes have been developed by other forest monitoring projects to assist in the interpretation of these mortality trends. Mode of death codes are best suited to trees that have moved from 'Alive' to 'Dead' between a series of surveys. It is often difficult to ascribe a Mode of Death code to dead standing trees in the initial AusPlots survey because evidence for the cause of death has sometime vanished with time. In this case, a 'best guess' is applied or the Mode of Death is recorded as unknown.

Procedure

1. For all live tagged trees assign an 'alive status' class which best characterises and describes the tagged tree according to the codes in Table 8.

2. For all dead trees assign a Mode of Death according to Table 9. On the initial survey, this may be a 'best guess' or recorded as 'unknown' (i.e. ARS).

Table 8: Alive Status Codes. Tree status codes can be used together in whatever combination is necessary. Thus for a multi stemmed, leaning and broken tree would be coded BCH. The only exceptions are codes ‘A’, ‘C’ and ‘D’. Please read the italised notes when using these codes. *Non-RAINFOR codes adapted from FPRIMIS.

Code	Description
**0**	Dead tree.
**A**	Alive, normal
**B**	Alive, broken stem/top and resprouting. Note at what height stem is broken
**C**	Alive, leaning by >10%
**D**	Alive, fallen (e.g. on ground)
**E**	Alive, tree fluted
**F**	Alive, hollow
**G**	Alive, rotten
**H**	Multiple stemmed individual (each stem >10cm gets a number), *always use with another code – e.g. if a tree is normal and with multiple stems use ‘AH’, etc.*
**I**	Alive, no leaves/few leaves
**J**	Alive, burnt
**K**	Alive, snapped <1.3m
**L**	Alive, has liana >10cm on stem or in canopy
**M**	Covered by lianas
**N**	New recruit, *always use with another code – e.g. if a tree is normal and new the code = ‘AN’, if a tree is broken and new the code is ‘BN’, etc.*
**O**	Lightning damage
**P**	Cut
**Q**	Bark loose of flaking off
**S**	Has a strangler
**T**	Is a strangler
**U***	Butt scar or fire scar
**V***	Dead top
**W***	Sweep
**X***	Buttressed
**Y***	Deformed at 1.3m
**Z**	Alive, declining productivity (i.e. nearing death, diseased etc.)

Figure 9: Mode of Death Codes: Select one code from each category. For example a dead tree that is standing, died alone and was killed by lighting would be 'APO'. For multiple deaths, the numbers of trees that died should be recorded and written in the comments column. For broken trees the height at which the breakage occurred should be recorded in the comments column.

**Physical mechanism of mortality (how the tree died)**	
**A**	Standing
**B**	Broken (snapped trunk)
**C**	Uprooted (root tip up)
**D**	Standing or broken, probably standing (not uprooted)
**E**	Standing or broken, probably broken (not uprooted)
**F**	Standing or broken (not uprooted)
**G**	Broken or uprooted, probably uprooted
**H**	Broken or uprooted, probably broken
**I**	Broken or uprooted (not standing)
**K**	Vanished (found location, tree looked for but not found
**L**	Presumed dead (location of tree not found, e.g. problems, poor maps, etc.)
**M**	Unknown
**Number of trees in mortality event**	
**P**	Died alone
**Q**	One of multiple deaths
**R**	Unknown
**Killed or killer?**	
**J**	Anthropogenic
**N**	Burnt
**O**	Lightning
**S**	Unknown whether killer or killed
**T**	Killer
**U**	Killed, no more information
**V**	Killed by tree that died broken
**W**	Killed by another tree that uprooted
**X**	Killed by branches from dead standing tree
**Y**	Killed by branches fallen from living tree
**Z**	Killed by strangler
**2**	Killed by liana
**3**	killed by strangler/liana weight
**4**	Killed by strangler/liana competition
**5**	Insect attack
**6**	Drought

5.7. Tagging Trees

Guidelines

Tagging trees is a critical step of the Ausplots Forest Monitoring Network methodology because it allows (a) the accurate relocation of each tree for repeat measurements and (b) the demarcation of the point of measurement such that the tree is always measured at the same height (i.e. 30cm below the tag). In the case that the tagging procedure fails due to the loss of nails and tags, Ausplots Forest Monitoring Network has high quality tree maps to relocate all trees in the plot.

There is no ideal method for attaching tags to trees. Conventions for tagging trees change from State to State in Australia depending on forest type and the experience of local field technicians. Most agencies use aluminium tags which can be either custom made in the lab or purchased from forestry suppliers. Tags can be attached to the tree with nails (aluminium or other) or wire (either wrapped around or embedded into the wood of the tree). The default method for Ausplots Forest Monitoring Network is to use nails long enough to allow for the growth of the tree. Aluminium nails are recommended because they cause less damage to chainsaws if the trees are eventually harvested. For most species, nails should be embedded in the cambium of the tree, although with some species (e.g. *Eucalyptus obliqua* in Tasmania) it is good practice to push the nail into the thick bark only such that the nail is slowly pushed out by the cambium as the tree grows. Local field technicians should be consulted to determine the adequacy of the proposed technique.

Procedure

After the diameter is measured and POM painted, a numbered aluminium tag is attached to the tree using a long aluminium nail according to the following procedure.

1) Tag the trees **exactly 30cm above the point of measurement (POM)**. For most trees this will be at 1.6m. In the initial survey, dead standing trees and tree ferns are not tagged in AusPlots.
2) Tag the trees such that the nail and tag is always facing the centreline of the subplot (i.e. where the Booker is located; Figure 17). This will help with relocating trees when sites are revisited.
3) Leave enough space on the nail for tree growth, whilst also securing the tag (See Figure 17).
4) Call out the number loudly so the Booker can record (and call back) that number.

Figure 17: Tagging procedure for AusPlots. Put the tag facing the centreline, exactly 30cm above the point of measurement

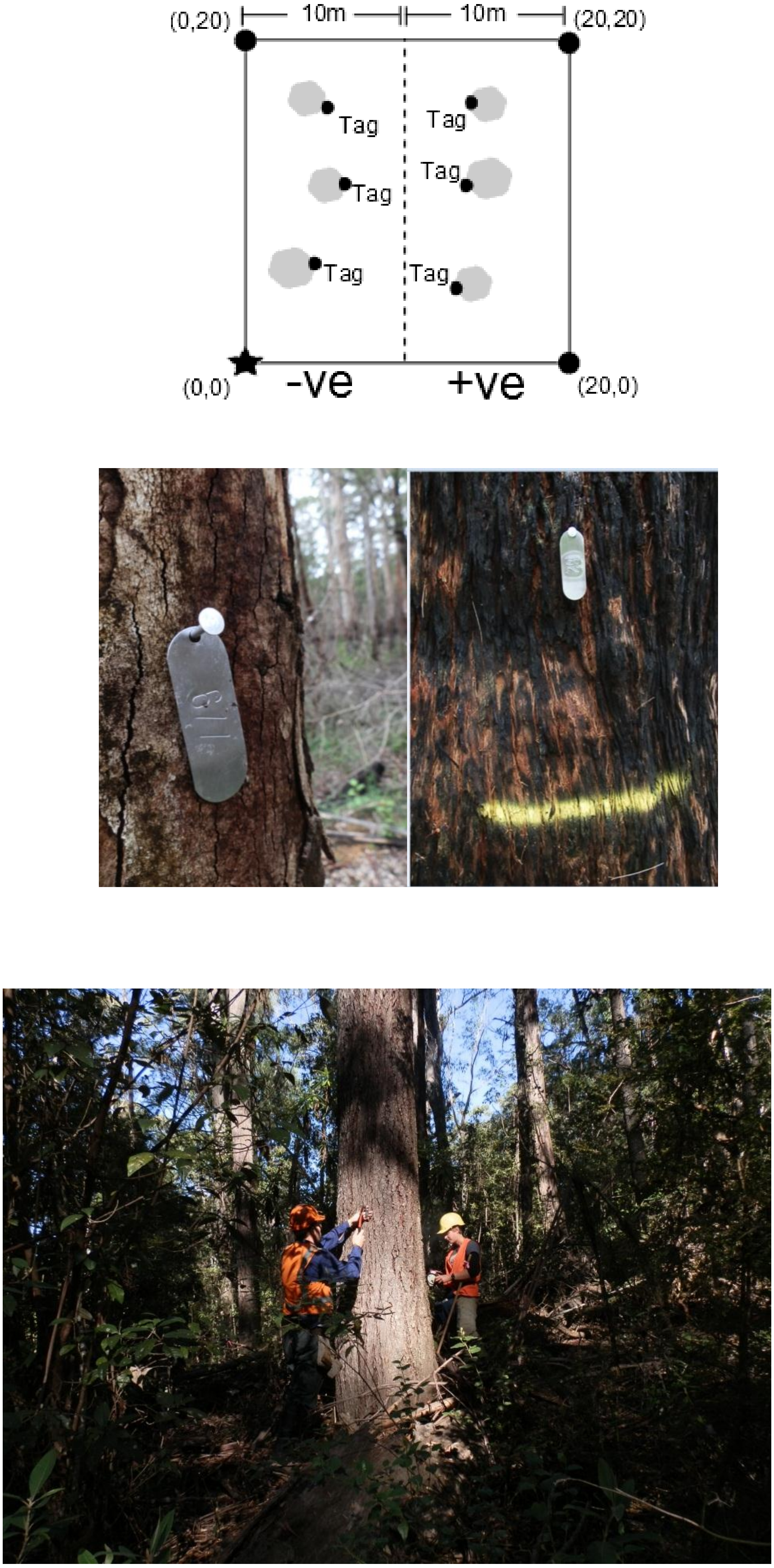

5.8. Tree and Bole Height

Guidelines

Note: Tree heights are most effectively measured after the diameter survey

Tree height is a basic forest inventory measurement that refers to the distance along the axis of the tree from the base to its uppermost point. Tree heights will be used in calculations of biomass and site productivity. Accurately measuring height is a difficult prospect in tall forests and AusPlots does not intend to track height growth in detail over time, although decadal trends may be picked up in young regenerating forests. Bole height refers to the height to the first major green branch, not epicormic in origin and which supports live foliage.

Depending on time constraints tree height and bole height (i.e. height to first major branch) is recorded for either:

a. all trees on the plot; or
b. a representative sample of all major tree species across their diameter range. 40-60 overstorey trees are measured, with ten trees in five DBH classes (i.e. 10-30cm DBH; 30-50cm DBH; 50-70cm DBH; 70-90cm DBH). The height of 20 trees from each of the most frequent understory species are also collected. Site specific diameter-height relationships are generated from this data and applied to unmeasured trees in the plot.

Procedure

Height is measured using trigonometric principles using a Vertex Hypsometer. The hypsometer calculates tree height using (a) horizontal distance to tree and (b) angle to top of tree. Two people are required for this procedure.

1. If you are measuring a subset of trees, use data from the large tree survey to nominate 40-60 trees across the diameter range of the most frequently observed overstorey trees and 20-30 trees across the diameter range of the most frequently observed understorey trees. Aim for 10 trees in each diameter size category (i.e. 10-30cm DBH; 30-50cm DBH; 50-70cm DBH; 70-90cm DBH). Exclude trees that are leaning, rotten, broken, forked below 5m, fallen or resprouted. Dominant or co-dominant trees should be selected where possible.
2. Field crew member 2 puts the transponder on the tree at 1.3m.
3. Field crew member 1 stands 10-30m from the tree (depending on tree height). The best results are achieved when the observer is approximately ‘one tree height’ away from the tree.
4. Press the orange ‘On’ button and use the ‘>’ and ‘<’ arrows to navigate to ‘Height’.
5. Look through the sighter and aim the crosshair at the transponder, ensuring that the vertex is upright. .
6. Hold the orange ‘On’ button down until the Vertex beeps and the crosshair begins flashing. The screen should show an approximate distance.

7. Point the crosshairs at the highest leaf directly above the main trunk of the tree.

   Importantly, the line of sight to the top of the tree should be made through the crown rather than the outside of the canopy. For trees that are leaning, the observer may have to 'reproject' the tree to vertical before making measurements.

8. Hold the orange 'On' button down until the Vertex beeps and the crosshair stops flashing.
9. **Record height and bole height** in the Large Tree Survey Data Sheet (Appendices)
10. Repeat steps 7 and 8 for the bole height by pointing the crosshair at the first main branch.
11. Press the '>' and '<' arrows together to reset the Vertex.

Figure 18: Trigonometric principals for measuring tree height.

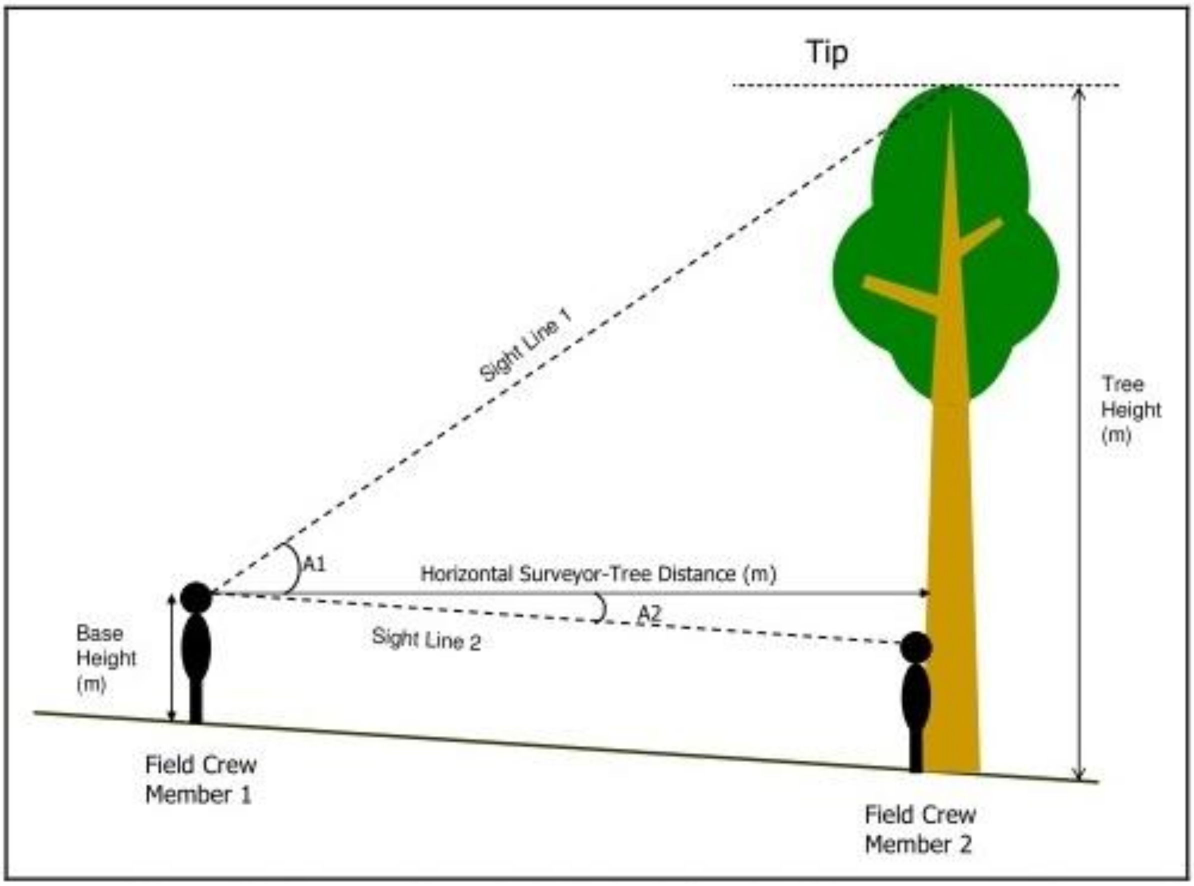

6. Vouchering of Specimens and Genetic/Isotope samples

6.1. Voucher specimens

Guidelines

Systematic collection and identification of voucher specimens across forests will add to knowledge about the distribution of Australian plant species and groups. This is especially powerful when linked with vegetation genetic profiles from leaves collected from each species.

Collection and expert identification of vascular flora is an important component of the AusPlots survey methods. Plant classification is constantly changing and shifts in species alignments and groupings are made as new evidence comes to light. Identifications are subject to change and voucher specimens help cross-reference these changes to previous research. They also ensure the currency and longevity of the data collected at sites.

It is intended that plant specimens will be identified by herbarium botanists and a subset of specimens will be included in respective local herbaria, subject to standards prescribed by each institution.

All information should be recorded on the Voucher Specimen and Genetic/Isotope Sample Data Sheet (Appendices)

Procedure.

1. Collect specimens of each different plant species with enough material to fill an A3 size herbarium sheet. Each sample should ideally contain flowers or buds, leaves, fruit, bark (for trees) and should be represented by as few separate pieces as possible. Where possible, ensure young, actively growing material is collected for genetic subsampling. During some surveys only sterile or vegetative material will be available – such material is still to be collected. The quantity of leaf material collected needs to be sufficient to enable removal of samples for genetic profiling (see 6.2).

2. In the field, large specimens can be collected into large plastic bags and smaller samples should be collected into smaller bags. Use paper bags to store smaller specimens separately and avoid contamination or mixing of specimens. To assist in the drying process, especially with wet plants, wrap each specimen with newspaper. Smaller plants can be kept in a small snap-lock bag with a little paper if the plant is wet until ready to put them in the press. Samples should be stored in the shade or in a cool place and transferred back to the lab for processing. Collection should be done later in the day and processing should be done on the day of collection.

3. In the lab, tag each specimen securely with a unique voucher label provided by AusPlots. Place the label on stems, away from any plant parts that are needed for examination during the identification process. Use paper envelopes for small specimens, with a voucher label attached to the envelope. Smaller specimens should be represented by a whole plant, including basal material and roots, particularly for Gramineae (Poaceae), Cyperaceae and Juncaceae. For smaller annuals and ephemerals, collect a number of individuals.

4. Record the voucher label number on the Voucher Specimen Data Sheet. Assign a field name for each sample. Where the collector is confident of the identification the assigned name should be a definitive species name. If the plant is unknown to the collector, it should be ascribed a descriptive name e.g. "yellow flowered wattle with bi-pinnate leaves". Use consistent taxonomy across jurisdictions following published names in APNI (Australian Plant Names Index at http://www.cpbr.gov.au/databases/apni-about/index.html.)

5. After labelling and recording, place specimens in a large sheet of newspaper and then into a plant press. At the time of pressing, remove a sub-sample of leaves for genetic analysis (see 6.2), taking care to minimalise handling. Be sure to wipe down the bench between samples. Specimens must be pressed by the end of each day's field work. Use one folded full tabloid sized newspaper sheet for each specimen (2-3 for succulents) and separate with corrugated cardboard dividers frequently, particularly between bulky specimens. Pressing plants on the day ensures that none are lost and improves identification, as diagnostic characters will be better preserved.

6. Change newspaper in the press regularly to prevent specimens becoming mouldy, particularly if the plants were damp when collected or were succulent species. Keep succulent plants in a separate press to facilitate easier changing of outside layers of paper and to reduce the risk of damaging other specimens.

7. Upon return from each trip, deliver voucher specimens to the local herbarium for identification and possible inclusion in herbarium collections. If posting, wrap each paper layer within another paper layer in the opposite direction to prevent samples falling out. The procedures for lodging specimens for identification vary with each herbarium and are detailed in separate agreements with the herbaria and the Council of the Heads of Australasian Herbaria. Details of agreements that relate to the delivery of samples can be released to collection team where this is appropriate.

8. For genetic profiling to enable DNA and isotope analyses, remove adequate leaf material (approximately $10cm^2$) from each voucher specimen. This process is covered in detail in section 6.2.

BARCODES ON VOUCHER LABELS

The use of voucher labels with barcodes in the field is an important part of the method that enables samples to be linked and tracked through all stages of collection, processing and storage. Barcodes will be used to identify:

- Each plant voucher specimen
- The leaf samples collected for genetic profiling (collected as a single sample in the field and subsequently divided into two samples, one for DNA and the other for isotope analyses. At the time of division and additional barcode is assigned to the isotope sample).
- Replicate leaf samples from dominant perennial species
- Each soil metagenomic sample

At a plot with 70 plant species, it is possible that almost 300 barcodes could be used for that plot.

Figure 19: Procedure for labelling voucher specimens

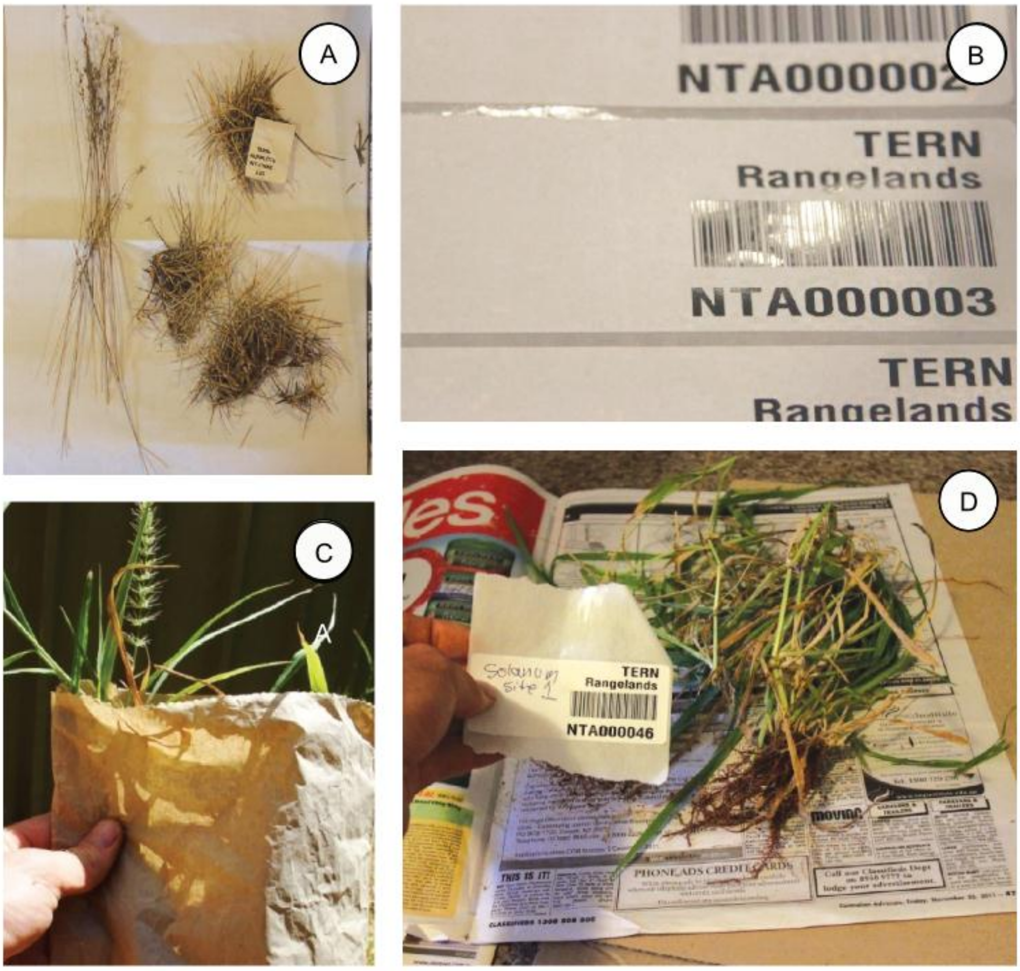

6.2. Genetic and isotope sample vouchering

Guidelines

DNA barcoding is a system designed to provide rapid, accurate, and automatable species identification by using short, standardised gene regions as internal species identifiers. As a consequence, it will make the Linnaean taxonomic system more accessible with benefits to ecologists and conservationists. By collecting multiple samples of dominant species within a plot and from different plots across regions and IBRA bioregions, variability in plant DNA can be determined. Ausplots represents the first attempt to undertake continental scale sampling to provide valuable genetic information on species and population connectivity and diversity both at fine and broad scales. This information is important for modelling communities.

Isotope analysis is conducted to find accurate information of carbon and nitrogen content of the leaves of the specimens. Collections at the plot scale and across regions and IBRA bioregions provide the opportunity for scale-based comparisons. Carbon isotopes vary depending on stress levels of a plant in a given locality. Relative to other localities and other species isotopes will provide an indication of where a plant is best adapted, where it is struggling and how this adaptation varies between and within species.

Isotope measures can also be combined with other data from the plant, such as physical signs of stress, morphological and genetic variations. These data can inform models of how each species will be affected by climate change. For a given area, it may be possible to predict which species are more likely to become extinct locally (i.e. those that are already struggling), and which are more likely to persevere.

As part of the AusPlots Sampling procedures, leaf samples will be collected from each plant species at each plot and dried to enable subsequent genetic analyses. By collecting these samples in synthetic tea bags and gathering a sufficiently large volume of material, one field sample can be subsampled to enable both genetic and isotope analyses to be undertaken. Smaller amounts of replicate leaf samples will also be taken from dominant perennial species present at each site to be used for genetic profiling only (not isotope).

Procedure.

1. Collect voucher specimens as a representative sample of each species occurring at each plot, for identification and lodging in state herbaria (as per the vouchering protocol, see 6.1)
2. From each voucher specimen, take a small sub-sample (equivalent to around 10cm^2 or five eucalypt leaves) of green leaves. The collected material should be young and free from disease, insect or fungal contamination wherever possible.
3. Handle the specimen to minimise skin contact and hence contact with other sources of organic carbon (important for the isotope analyses). For broad-leaf plants, hold the leaves by the petiole (leaf stem) or by the

base of the leaves for grasses where possible. This sample should then be carefully placed into a synthetic teabag and sealed.

4. Label the teabag with an adhesive voucher label. Record the voucher ID, the field name for the plant and the plot name in the Voucher Specimen Field Sheet.
5. Place the teabag in a sealable lunch box with 1cm of silica granules (10% self-indicating mixed with 90% standard non-indicating granules) and seal. If possible, a single lunchbox, clearly labelled with the plot identifier should be used for each plot.
6. Store the container in a cool location out of direct light.
7. Over the duration of the trip, replace the silica granules regularly. When the self-indicating granules change colour from blue to pink (or orange to clear) their moisture absorbing capacity has been reached and the granule mix should be replaced with fresh silica mix. Do not discard the used silica, as it can be oven dried at 100°C in foil trays and re-used.
8. For each dominant perennial species in the plot (i.e. foliage projected cover >2% or opaque canopy cover >5%) collect further leaf material (approximately 5cm^2) from an additional four individual plants of the same species distributed across the plot (i.e. a total of five replicates per species).
9. Place the leaf material from each replicate into a new teabag labelled with a new voucher label.
10. Record the barcode and the species name in the Voucher Specimen Field Sheet.
11. Store the teabags in an airtight container on silica granules (as for step 5).
12. On return from the field, forward the samples to AusPlots at the Universitiy of Adelaide (address on Page 2). If this is not immediately possible and the collected samples are stored for an extended period before being sent to AusPlots (i.e. weeks), samples should be stored in a freezer at -20°C until forwarding.
13. At the University of Adelaide, the leaf samples taken from the voucher specimens will be divided for both DNA and isotope analysis. All teabag samples will then be forwarded to approved analytical institutions using standard exchange or export/import procedures.

Figure 20: Steps involved in preparing tea bag samples for genetic analysis.

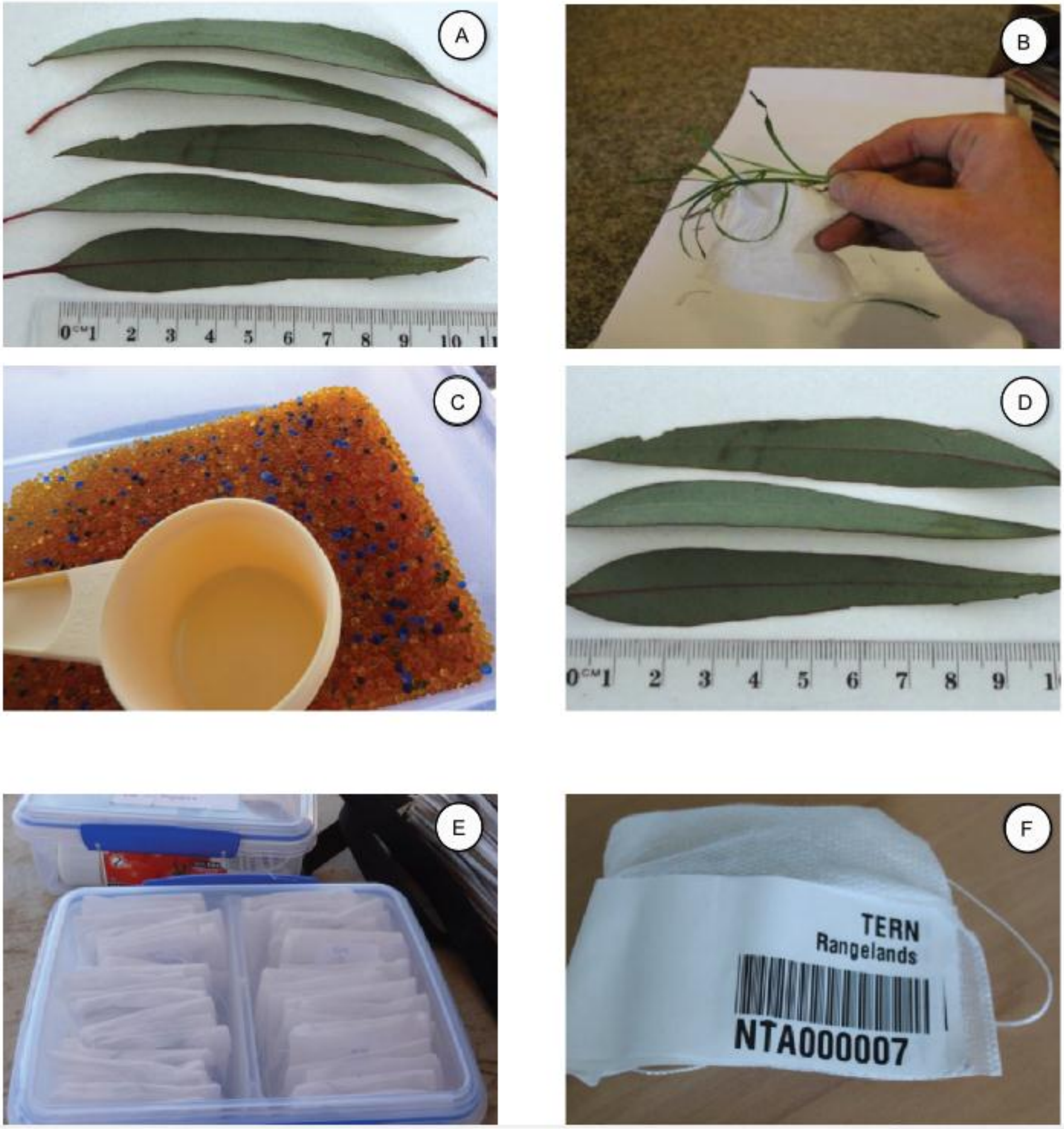

7. Soil Metagenomics

Guidelines

As part of TERN AusPlots there is a commitment to collect soil samples for metagenomic analysis. Metagenomics is the study of genetic material recovered directly from environmental samples. Soil metagenomics provides the opportunity to understand what organisms are present at survey sites and provides an indication of their abundance.

Soil samples are to be taken at nine soil observation locations across each plot. These soil samples should be located to cover the variety of micro-habitats within the plot.

Location details of all the soil samples collected are recorded on the Soil Metagenomics Data Sheet (see Appendices).

Procedure:

1) Scrape any loose and obvious plant material and animal scats from the soil surface.
2) Use a trowel or small shovel (ensuring that it is not contaminated with soil from another location) to remove approximately 200g of the soil surface layer (max 3cm depth).
3) Place this sample in a calico bag and label it with details of the plot, the sampling location and the date.
4) Place calico bag in a larger snap lock bag with half a cup of mixed silica granules (self indicating 10% mixed with standard non-indicating granules 90%) as used for the leaf DNA samples. More silica mix may be required if the sample is damp. Label this bag with the date (e.g. DD,MM,YYYY), site name (e.g. Dawson Forest Block), site ID (e.g. WAWARF0004), approximate X,Y sample location (e.g. (20,40) or (60,80)) and label "Metagenomics" (Figure 21). Silica will need to be checked regularly and changed until the self indicating granules retain their original color. A change in colour from blue to pink (or orange to clear) of the self-indicating granules reveals its moisture absorbing capacity has been reached and it needs to be replaced with fresh silica. Do not discard the used silica as it can be oven dried and re-used.
5) Place all nine samples in a large calico bag and label this clearly with the same details as recorded on the calico bag.
6) Details of all the soil samples collected are recorded on the Soil Metagenomics Data Sheet (see Appendices). Record the date (e.g. DD,MM,YYYY), site name (e.g. Dawson Forest Block), site ID (e.g. WAWARF0004), approximate X,Y sample location (e.g. (20,40) or (60,80)).
7) On return from the field forward the samples to AusPlots at the University of Adelaide. If this is not immediately possible and the collected samples are stored for an extended period (i.e. weeks) before being sent to AusPlots, samples should be stored in a freezer at -20°C until forwarding.

Figure 21: Example of a soil sample collected for metagenomics.

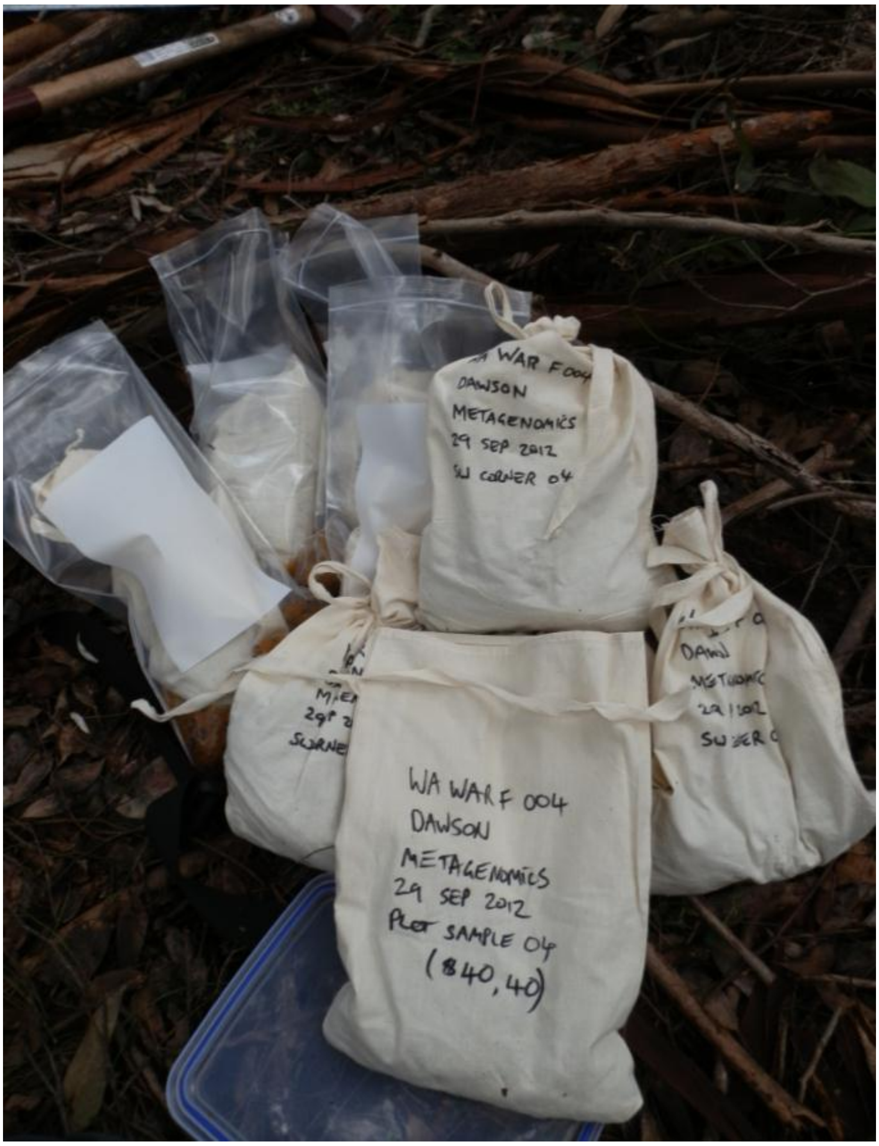

8. Canopy Photography

Guidelines

Canopy cover and leaf area are key parameters in models of stand level eucalypt growth and water use. Changes in canopy cover can be linked to changes in tree health and the effects of disturbance on forest stands. Hemispherical or fisheye photographs of the forest canopy (e.g. Figure 22) are easily attained from consumer-grade digital cameras making this approach inexpensive, rapid and simple. Canopy cover photos can also be used to validate remote sensing of the forest stand.

Ausplots Forest Monitoring Network collects two digital hemispherical photos from each of the sixteen interior sampling points in the grid (Figure 22). These photos are digitally tagged with a side identifier and coordinate information. These photos will be used as a baseline for measuring canopy cover change over time.

Figure 22: An example of a hemispherical photo of a tall eucalypt canopy and the distribution of 16 photo points within the sampling grid.

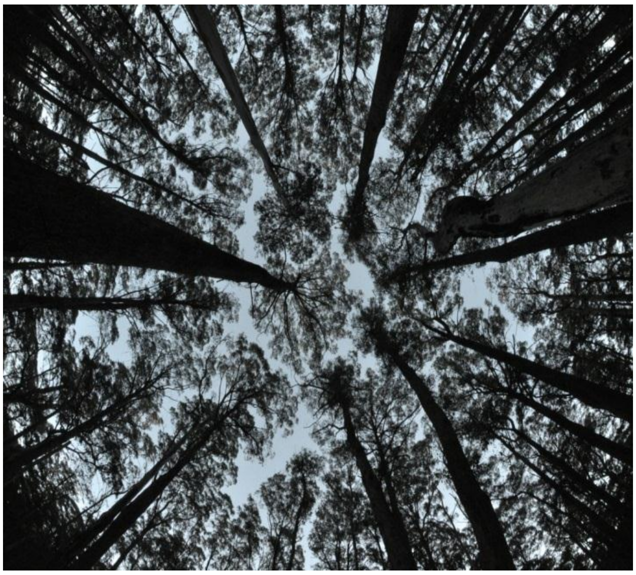

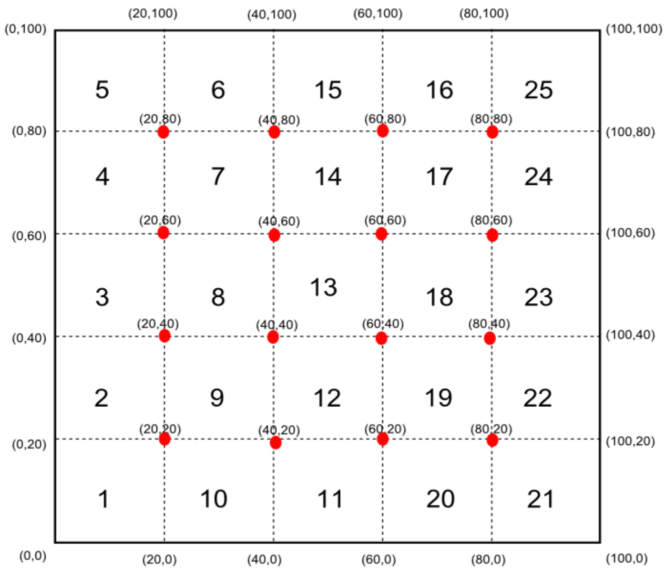

Procedure

1. Attach fisheye lens to camera (e.g. Nikon AF Fisheye NIKKOR 10.5mm). Camera settings should be as follows. Set to F2 (about 70mm focal length in 35mm equiv.), aperture priority, maximum F-stop, auto exposure, auto focus. All digital photographs should be collected as FINE JPEG images with maximum resolution.
2. Walk to the post and take a photo of the coordinate label or a coordinate card (e.g. 20,40; Figure 23). During the data management step, the next photo (or photos) in the sequence will be ascribed this coordinate.
3. Attach the camera to a tripod so the lens is pointing vertically upwards. Set tripod at 1.0 m. Level the tripod using a spirit bubble. Ensure that the photo is oriented such that the top of the photo (i.e. 12 O'Clock) is oriented facing 'up' the plot (i.e. parallel with the line from 0,0 to 0,100; see Figure 7)
4. Clear away any low foliage that is obviously obscuring the canopy.
5. Take **two** hemispherical photos. It is good practice to immediately check each photo on the camera screen.
6. Repeat steps 2-5 for each of the 16 internal posts (Figure 22).
7. Upon return to the lab, immediately download the photo's onto a computer, create a backup, and then label each canopy photo with their appropriate coordinates according to the following rule "SITEID_SITENAME_X_Y_PHOTONUMBER" (e.g. NSFSEC0001_Newline_20_40_1).

Figure 23: A photograph of the coordinate is taken before each canopy photo. During the data management step, the next photo (or photos) in the sequence will be ascribed this coordinate.

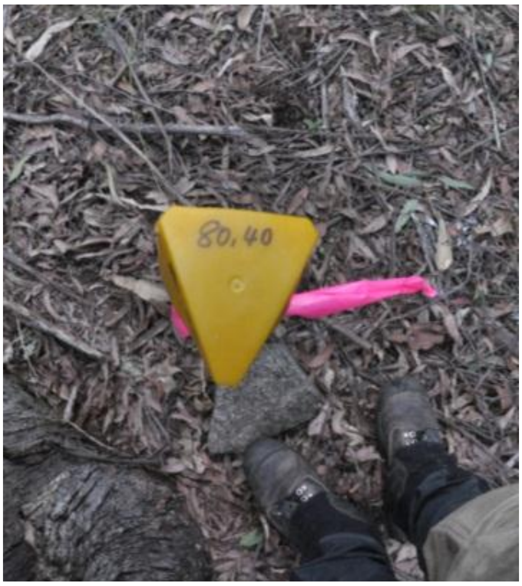

9. Fuel load survey

9.1 Fuel transect allocation

Guidelines

Fuel surveys are conducted along four 28.3 m transects across the TERN Ausplots Forest Monitoring Network sites. The existing plot configuration (marked out using star pickets and steel droppers in Survey 1) is used to locate the four transects. The transects run along the long edge of an isosceles triangle formed from two edges of the 20 x 20 m sub-plot (Figure 24). The four transects and their location are intended to provide a representative sample of fuels across the plot and ensure that transects are re-locatable for further surveys.

Procedure

1. Transect A is laid out using a 30 m tape from point 40,40 to the 20,20 steel dropper (Figure 24). Use a tent peg to secure the tape at 40,40, and tie off at 20,20.
2. A photograph along the transect is taken from the 20,20 steel dropper back towards the 40,40 steel dropper. A second photograph is taken from the 40,40 steel dropper towards the 20,20 steel dropper (see Figure 25). Both photo numbers are recorded on the fuel survey datasheet (see Section 9.2)
3. The compass bearing (°) between points 40,40 and 20,20 is taken and recorded on the fuel survey datasheet.
4. The slope (°) and aspect (°) of the site at the mid-point of the transect is taken and recorded on the fuel survey datasheet.
5. Following the completion of the fuel survey of Transect A, Transects B-D are laid out in turn, in the same manner described above, as per Figure 24 and Table 9.

Figure 24: Fuel survey transect layout.

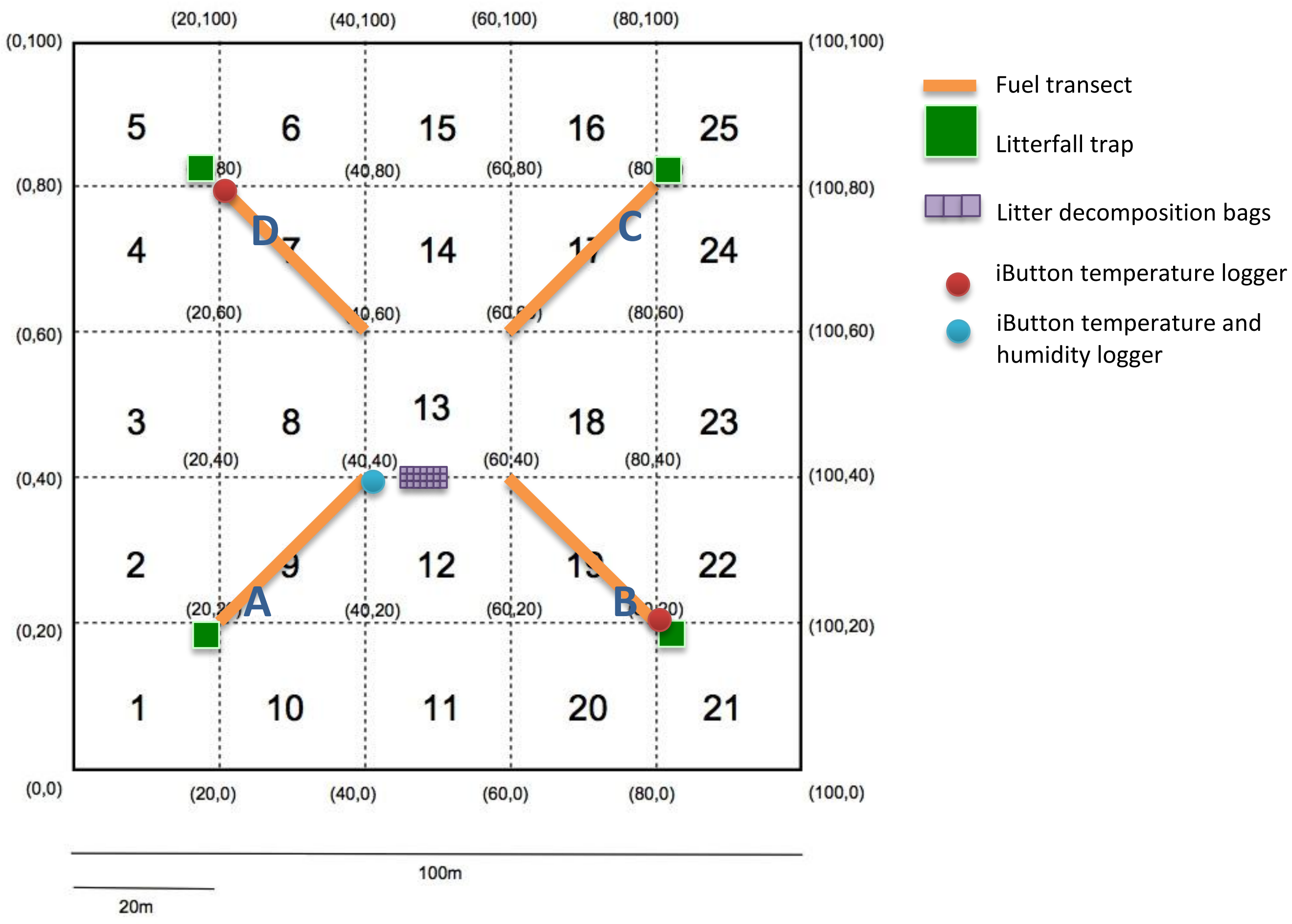

Table 9 – Transect start and end points

Transect	Start	End
A	40,40	20,20
B	60,40	80,20
C	60,60	80,80
D	40,60	20,80

Figure 25: Example photograph of a fuel survey transect, taken from the steel dropper along the transect.

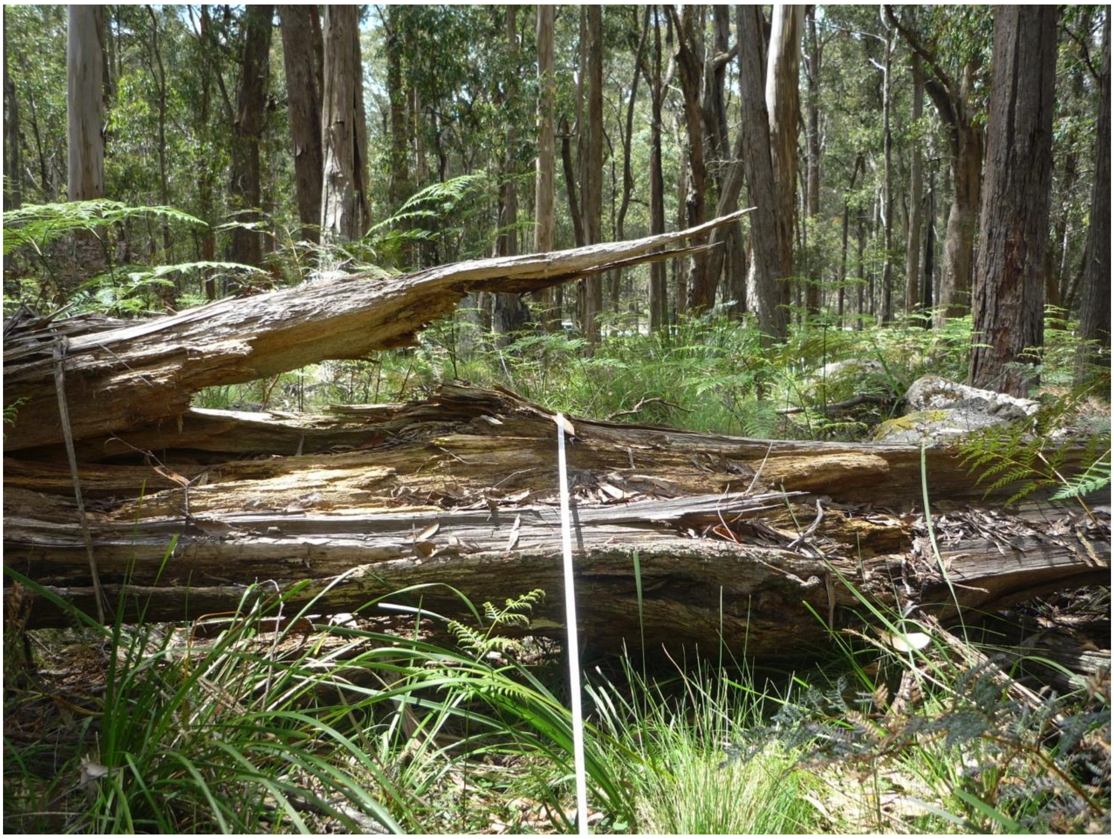

9.2 Fuel Survey datasheets

Guidelines

One datasheet is used per site. Each datasheet has provision for the four transects at each site. Datasheets are printed on waterproof paper. Spare datasheets should be kept on-hand. Fuel survey datasheets are attached as Appendix 15.5.

Procedure

1. Record the site name and the names of the observers.
2. Record the date.
3. Record the plot coordinate of the beginning and end of the transect (as per Table 9, Section 9.1, unless alterations are necessary).
4. Using a compass, take the bearing from the start to the end of the transect and record.
5. Take a photo from the start dropper towards the end dropper and record the number as the 'start photo'.
6. Take a second photo from the end dropper towards the start dropper and record the number as the 'end photo'.
7. Measure and record the slope and aspect of the site at the mid-point of the transect.
8. Measure and record the fuel height and grass height as per Section 9.3.
9. Measure and record the woody fuel measurements as per Section 9.4.
10. Measure and record the shrub biomass data as per Section 9.5.
11. Measure and record the fine litter, standing grass, herbs and vines data as per Section 9.6.
12. Collect and label soil samples as per Section 10. Measure and record the duff depth as per Section 10.
13. Install litterfall traps as per Section 11.
14. Install the iButton temperature and humidity loggers as per Section 12.
15. Prepare and label litter decomposition bags as per Section 13, and record the sample numbers on the datasheet. Install litter decomposition bags as per Section 13.

9.3 Fuel and grass height measurements

Guidelines

Litter includes leaves, wood and other plant parts that have become detached from the parent plant (or from the ground, where the entire plant is involved). Woody fuels are un-attached items of woody origin (i.e. from trees or shrubs). They may be twigs, sticks or logs.

Grass includes any attached (live or dead) graminoids (that is, plants with a grass-like growth form, including members of the Poaceae family as well as sedges and rushes).

The height of fuel (litter or woody fuels) and grass above the mineral soil are measured at eighteen points along the transect (10 cm increments from 7.0 m and 22.0 m) using a builders' ruler (see Figure 26 and 27).

Procedure

1. At ten centimetre intervals between 7.0 and 7.8 m, stand the builders' ruler perpendicular to the mineral soil surface (it may need wiggling to get through the litter and duff layers). Record the height of the standing grass and fuel layers at each interval on the fuel datasheet (see Section 9.2).
2. Repeat at 22.0 - 21.2.

Figure 26: Measurement of fuel and grass height from 7.0 and 22.0 m.

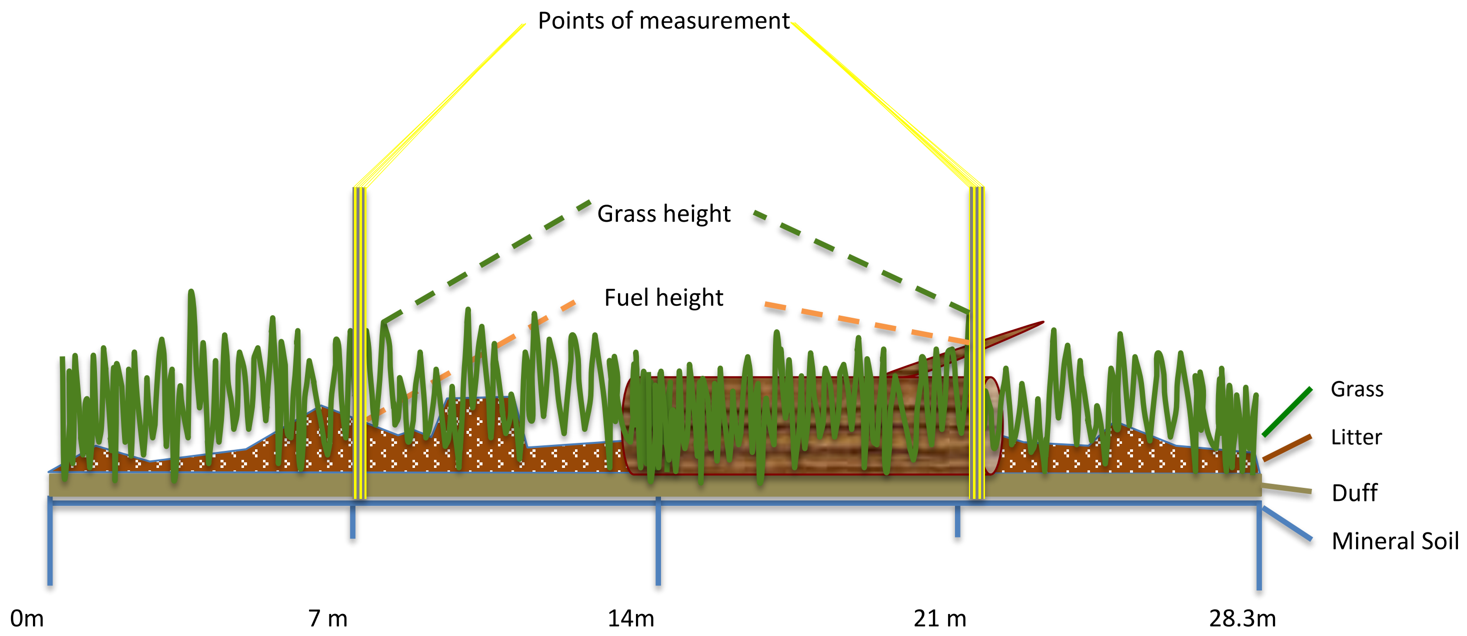

Figure 27: Example photograph showing the measurement of fuel and grass height

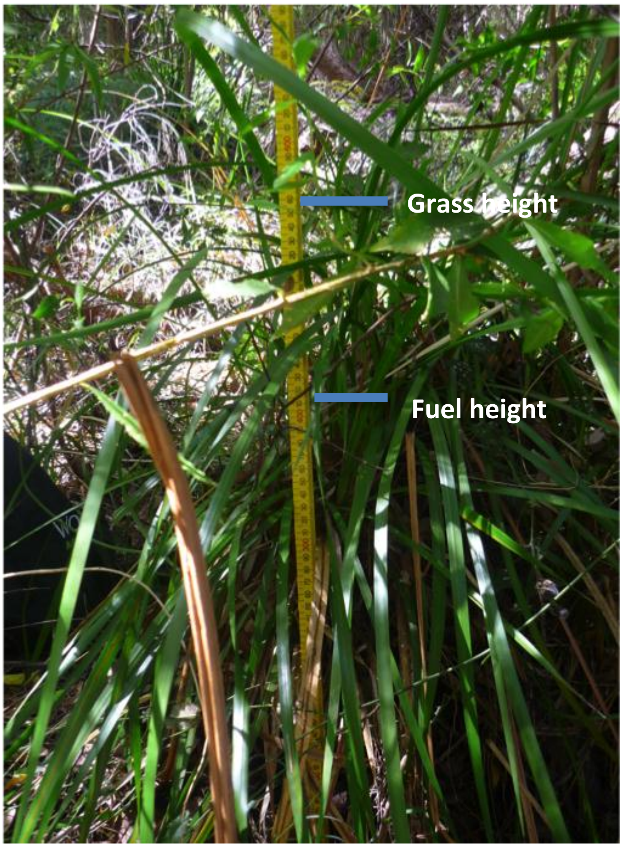

9.4 Woody fuel counts

Guidelines

Woody fuels are un-attached items of woody origin (i.e. from trees or shrubs) and represent a significant store of carbon in a forest. They may be twigs, sticks or logs.

Woody fuels are measured in four size classes [(1) 0-6 mm; (2) 6-25 mm; (3) 25-76 mm; and (4) >76 mm] at varying scales along the 28.3 m transect. Woody fuels are measured as counts of vertical planar intercepts. Size classes (1) and (2) are counted along two 2 m sub-transects between 6-8 m and 20-22 m (Figure 28). Size class (3) is counted in two 4m sub-transects between 5-9 m and 19-23 m (Figure 28). Size class (4) is counted along the entire 28.3 m transect (Figure 28). The diameter at intercept is also recorded for size class (4), as well as the estimated diameter of the hollow centre (if present), and whether the log is 'sound' or 'rotten'.

Procedure

1. Count the number of woody fuel intercepts with a diameter 0-6mm between 6 and 8 m, and 20 and 22 m. Record on the fuel datasheet (see Section 9.2).
2. Count the number of woody fuel intercepts with a diameter 6-25mm between 6 and 8 m, and 20 and 22 m. Record on the fuel datasheet.
3. Count the number of woody fuel intercepts with a diameter 25-76mm between 5 and 9 m, and 19 and 23 m. Record on the fuel datasheet.
4. Using a DBH tape, measure and record the diameter at intercept of woody fuels with a diameter >76mm along the entire 28.3 m transect. Estimate the diameter of the hollow (if present) for each log, as well as if it is 'sound' or 'rotten'. These three parameters should be recorded on the fuel datasheet.

Figure 28: Extent of various measurements along the fuel survey transect

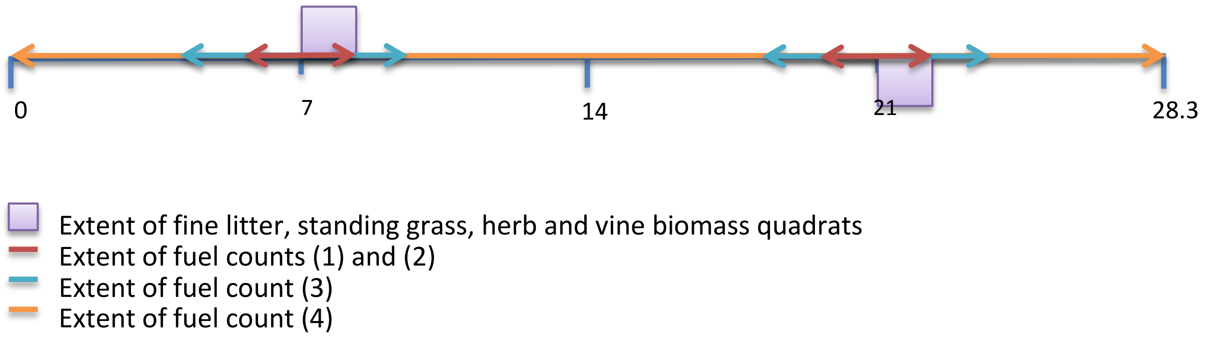

9.5 Shrub biomass

Guidelines

For the Ausplots Forest Monitoring Network sites, shrubs are considered any plants less than 10 cm DBH that 'snap' when broken, and as such may include ground ferns as well as woody plants.

Shrub biomass is estimated by recording the height (to the nearest cm, nearest 10 cm for shrubs over 2 m), life-form and density of 20 shrubs. 5 shrubs are recorded in every 7 m sub-transect. For shrubs 1.3 m and over in height that are included in the 20 shrub measurements, the diameter at 1.3 m (DBH) is also recorded.

Procedure

1. Identify the five shrubs closest to the transect between the 0 m and 7 m marks. Record the density of these five shrubs (Figures 29 and 30) to the nearest 10 cm increment.
2. Record the height and life-form of each of these 5 shrubs, as well as the DBH for shrubs over 1.3 m. The height should be measured with a measuring tape (where practicable), and recorded to the nearest cm increment (10 cm increments for plants over 2 m tall).
3. Repeat for the five shrubs nearest the transect between 7 - 14 m, 14 - 21 m and 21 – 28 m.

Figure 29: Shrub density measurements

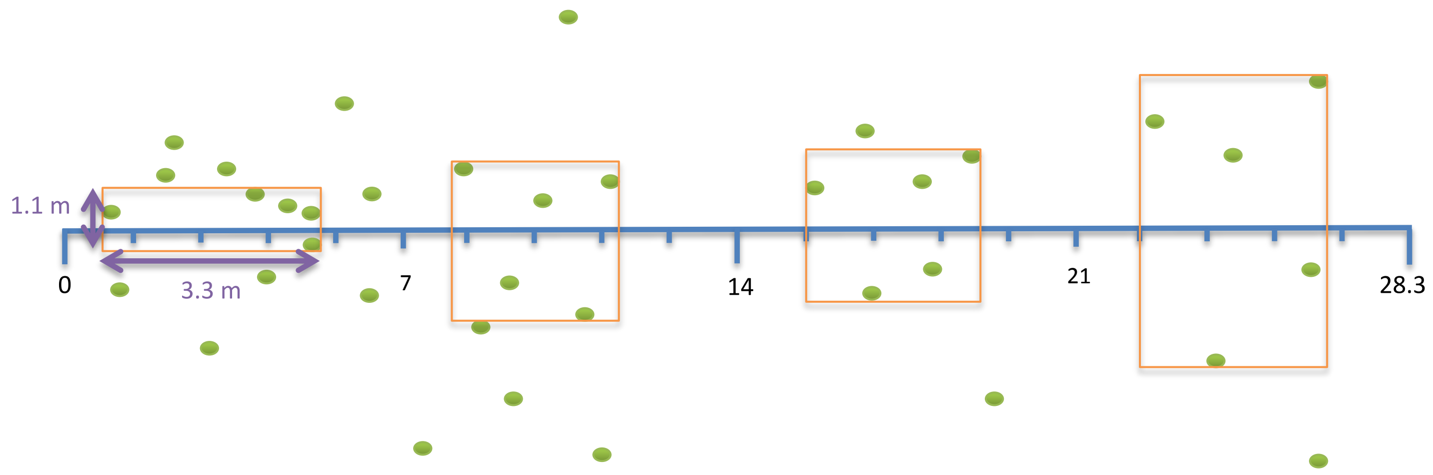

Figure 30: Photograph showing shrub density measurements

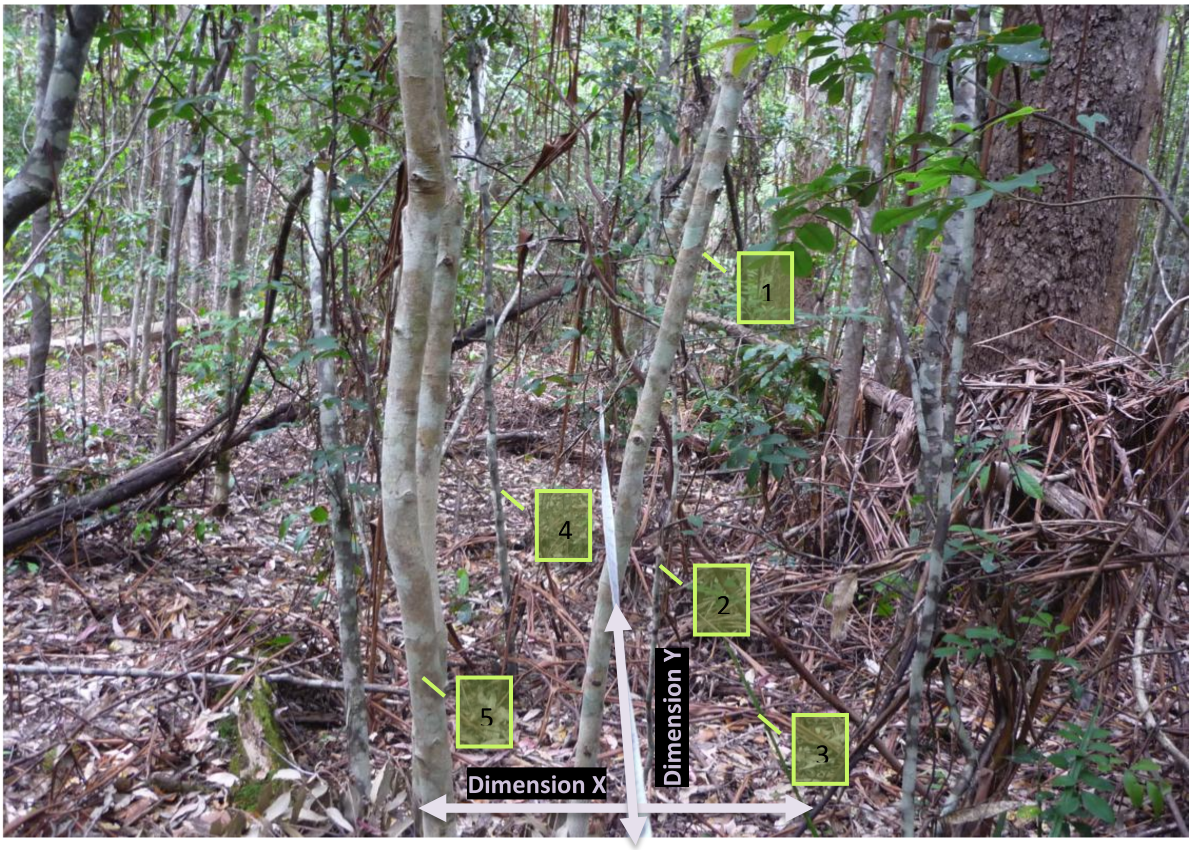

9.6 Fine litter, grass, herbs and vines measurement

Guidelines

Fine litter includes detached leaves of trees or shrubs, twigs ≤ 6 mm diameter and any detached part of grasses or herbs. Grass includes attached (live or dead) graminoids (that is, plants with a grass-like growth form, including members of the Poaceae family as well as sedges and rushes). Herbs are attached (live or dead) non-woody, non-graminoid plants, including bryophytes and forbs. Vines are twiners/climbers (live or dead) attached to the soil.

Fine litter, grass, herbs and vines are recorded separately from two 1x1 m quadrats at 7-8 m and 22-21 m (Figures 28 and 31). A representative sample of each component from the site should be collected, taken to the lab, weighed, dried to a constant weight at 70°C and re-weighed to estimate moisture content. Labelling protocol for sample bags is as follows:

Site _Sample type eg. Herberton_Litter

Procedure

1. Set-up the 1x1 m PVC quadrat with one edge along the 7-8 m section of the tape.
2. Cut and collect all herbs from the 1x1 m quadrat and weigh. Record the weight on the fuel datasheet (see Section 9.2). Weigh the sample bag and record this weight.
3. Cut and collect all grass from the 1x1 m quadrat and weigh. Record the weight on the fuel datasheet. Weigh the sample bag and record this weight.
4. Cut and collect all vines from the 1x1 m quadrat and weigh. Record the weight on the fuel datasheet. Weigh the sample bag and record this weight.
5. Collect all fine litter (non-attached) from the 1x1 m quadrat and weigh. Record the weight on the fuel datasheet. Weigh the sample bag and record this weight.
6. Repeat steps 1-4 for the second quadrat between 22-21 m.
7. Collect a sub-sample of each category from the site (grass, litter, herbs and vines) and record the fresh weight. The litter sub-sample should be at least 350 g to allow sufficient material for litter decomposition bags (see Section 13). Dry in a dehydrating oven at 70°C to a constant weight. Re-weigh, and record the weight of each sample on the fuel datasheet (see Section 9.2).
8. Reserve the litter sample for use in litter decomposition bags (Section 13).

Figure 31: Photographs showing 1 x 1 m quadrat before (L) and after (R) collection and measurement of litter, grass, herbs and vines

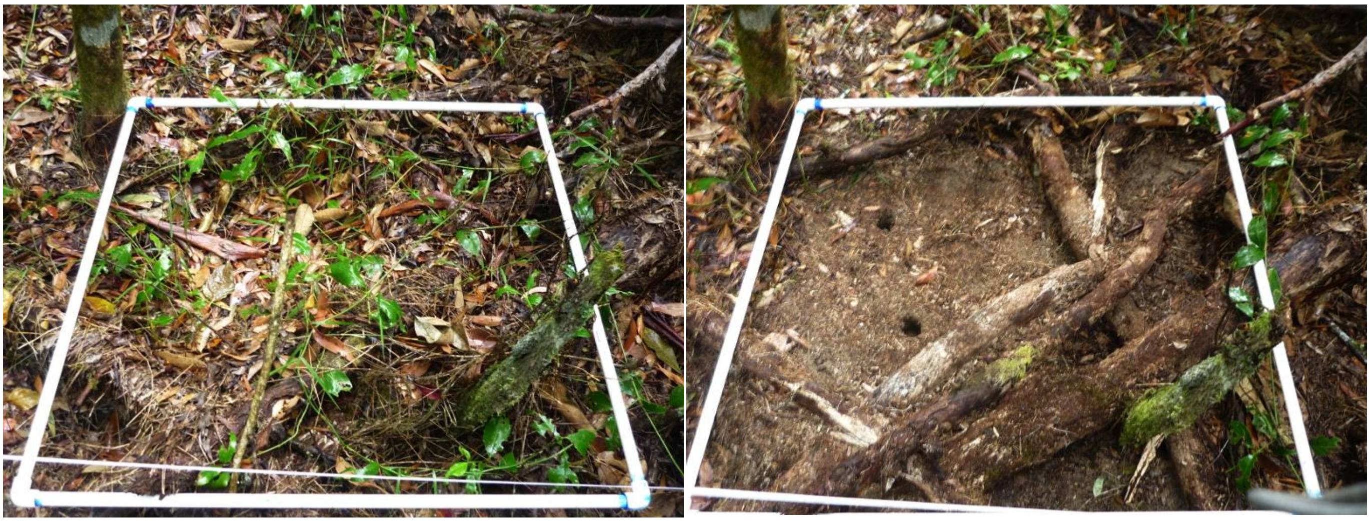

10. Soil sampling

Guidelines

Soil samples are collected from the 1 x 1 m quadrats utilised in Section 9.6, with four 10 cm soil cores being collected from each transect (two from each quadrat, Figure 32). Soil cores are bulked for each transect, kept in a cooler bag during the day, and dried on return to the lab.

The duff layer is composed of moderately to highly decomposed litter found between the mineral soil surface and litter layer. A duff sample for each site it bulked from the duff collected during soil sampling (Figure 32). The duff depth is measured with a builders' ruler following soil sampling.

Procedure

1. At the first quadrat (7-8 m) hammer the soil corer to 10 cm depth. Remove from ground, and place soil in labelled calico bag (one per transect). If duff is present, remove this from the top of the sample and place in a labelled calico bag (one per site).
2. Repeat step 1 with a second core at the first quadrat.
3. Measure the duff depth using a builders' ruler, and record on the fuel datasheet.
4. Repeat steps 1-2 at the second quadrat (22-21 m).
5. At the lab, dry the soil samples in the dehydrating oven for 48 hours at 105 degrees Celsius.
6. Once soil is dried, double bag in two zip-lock bags, and label with the TERN site code, site name and transect ID.

Figure 32: Photograph showing soil and duff sample

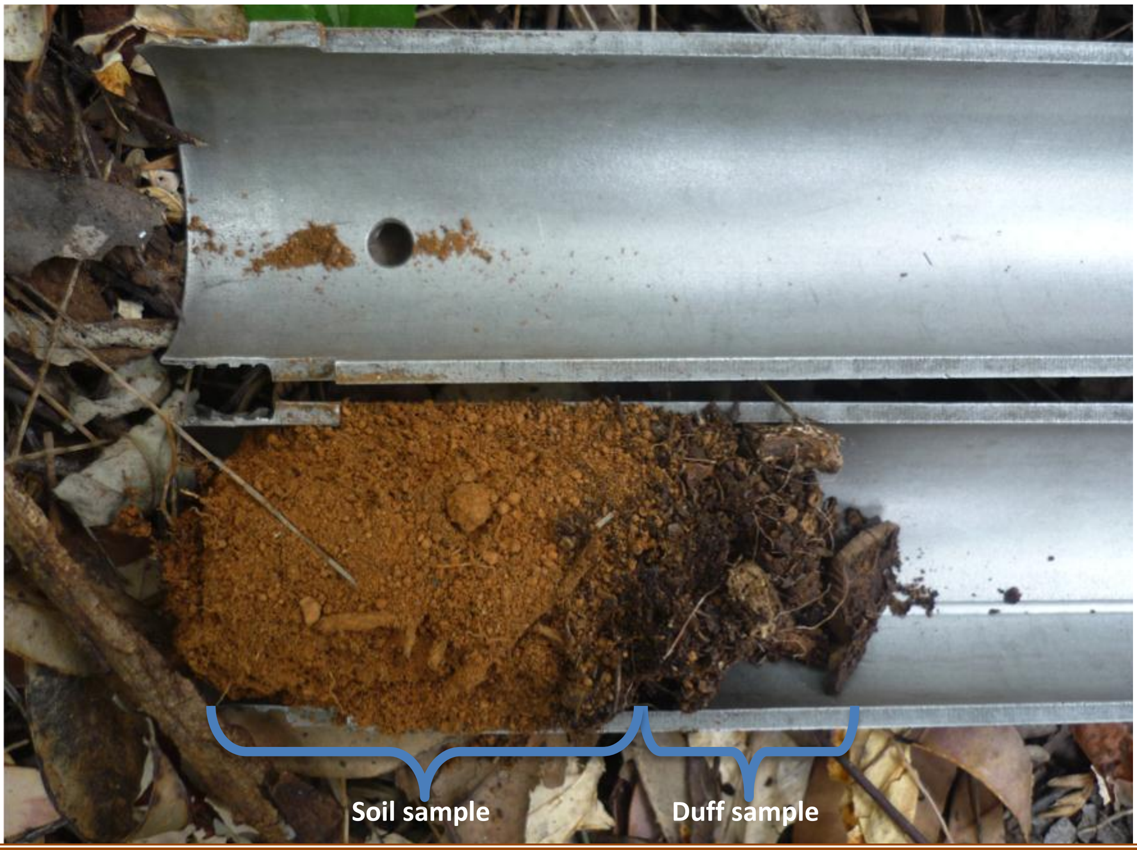

11. Installation of litterfall traps

Guidelines

The litterfall traps used for the Ausplots Forest Monitoring Network sites are 0.75 x 0.75 m in dimension and have an input area of 0.56 m^2. Four litterfall traps are placed across the site as per Figure 24. One corner of the litterfall trap should align with the steel dropper. The frame for each litterfall trap is constructed from 32mm diameter PVC pipe, including four 57 cm lengths and four 6.8 cm lengths (which form the sides), and four 47 cm lengths (which form the legs). These are joined with four right-angle elbow joins and four 'T' joins. The net for the litterfall trap is manufactured from a 1.8 x 1.8 m piece of shadecloth, which has splits along each side through which the side lengths of PVC pipe are threaded. The frame is assembled and secured with PVC cement solvent on- site (Figure 33). The legs have pre-drilled holes at the base, and are pinned to the ground using tent pegs

Procedure

1. Take the shadecloth net, and thread the side lengths of the litterfall trap frame through the splits.
2. Apply PVC cement solvent to the join piece and connect the corners of the frame side lengths together.
3. Attach the legs of the frame to the join piece with PVC cement solvent, with the pre-drilled holes at the opposite end.
4. Attach the legs of the frame to the ground with tent pegs through the pre-drilled hole.

Figure 33: Photographs showing litterfall trap materials (L) and an assembled litterfall trap (R)

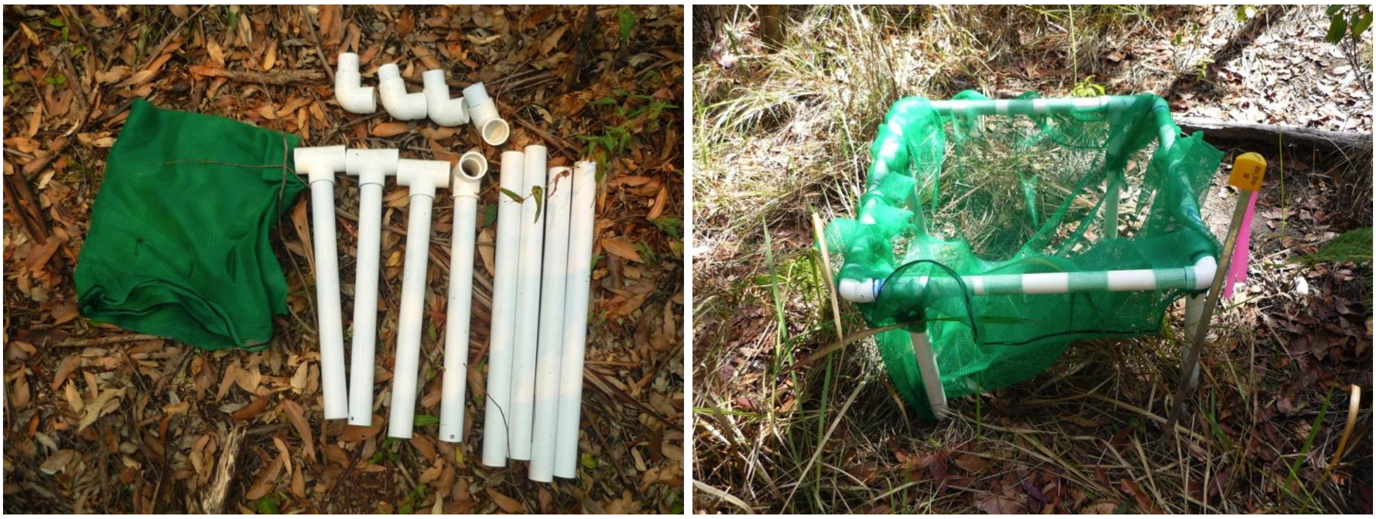

12. Installation of temperature and humidity loggers

Guidelines

Three iButton data loggers are installed at each site. Two are Thermochron DS1922L, which record temperature at four-hourly intervals, and one is a Hygrochron DS1923, which records both temperature and humidity at four-hourly intervals. iButtons are attached to plastic fobs, which are wired on to the top of the steel dropper and placed inside the yellow safety cap.

Procedure

1. Before travelling to site, attach the iButtons to their plastic fob.
2. Using the Thermodata software set the iButtons to record at 4-hourly intervals beginning at midnight the day after installation.
3. At the 20,80 and 80,20 steel droppers, remove the yellow plastic cap, attach the plastic fob with one of the Thermochron DS1922Ls to the steel dropper with wire, and replace the yellow safety cap (Figure 34).
4. At the 40,40 steel dropper, remove the yellow plastic cap, attach the plastic fob with the Hygrochron DS1923 to the steel dropper with wire, and replace the yellow safety cap (Figure 34).
5. Where the safety cap has holes in it, use electrical tape to cover the holes (Figure 34).

Figure 34: Photographs showing datalogger attached to a steel dropper (L) and the holes in a post cap covered with electrical tape (R)

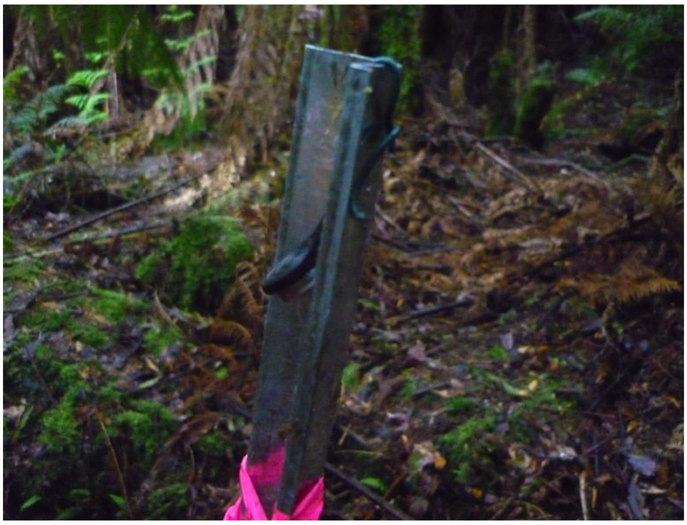
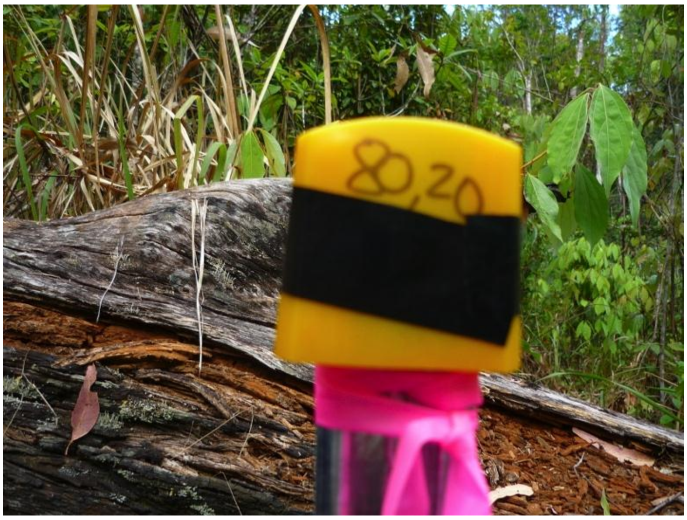

13. Installation of litter decomposition bags

Guidelines

The litter decomposition bags used for Ausplots Forest Monitoring Network sites are 20 x 20 cm in dimension. They are constructed from two pieces of fine nylon mesh. Three sides are sewn together, the bag filled with approximately 10 g dried fine litter (see Section9.6) and the fourth side sewn shut. Six bags per site are filled with a 10 x 10 cm piece of unbleached, organic cotton calico instead of leaf litter, to act as a standard for decomposition rates across all sites. The bags are then installed at the site, and kept in place using weed-matting pins. 21 litter decomposition bags are placed in a 3 x 7 grid from the 40,40 steel dropper towards the 60,40 steel dropper (Figures 24 and 35).

Procedure

1. At the lab, place a two 20 x 20 cm pieces of fine nylon mesh together.
2. Zig-Zag stitch three sides shut using a sewing machine.
3. Take approximately 10 g of litter collected and dried in Section 9.6, weigh and record the weight, and then place in bag. The sub-sample should be representative of the whole sample in terms of leaf/twig sizes and species. Spread the sample out in the bag to avoid it remaining in one clump.
4. Record the bag ID number (from the aluminium tag, making sure that all bags in each region have a unique number), and place the aluminium tag in the mesh bag.
5. Sew the fourth side shut.
6. Label the bag with its ID number by writing directly on the nylon mesh.
7. Make up 15 bags per site with this method.
8. For the remaining six bags, take a 10 x 10 cm piece of unbleached, organic cotton calico, weigh and record the weight. Place in the bag and sew shut.
9. Weigh the completed bags, and records the weights along-side the identification numbers on the fuel datasheet (see Section 9.2).
10. At the site, clear the leaf litter for a few meters from the 40,40 steel dropper towards the 60,40 steel dropper. Arrange the litter decomposition bags in a 3x7 grid along this line, keeping the bags in numerical order (Figure 35). Secure the corners of the decomposition bags with weed-matting pins. Bags should not overlap, but one pin can be used to secure bags adjacent to each other.

Figure 35: Photograph showing litter decomposition bags arranged in a 3x7 grid at site

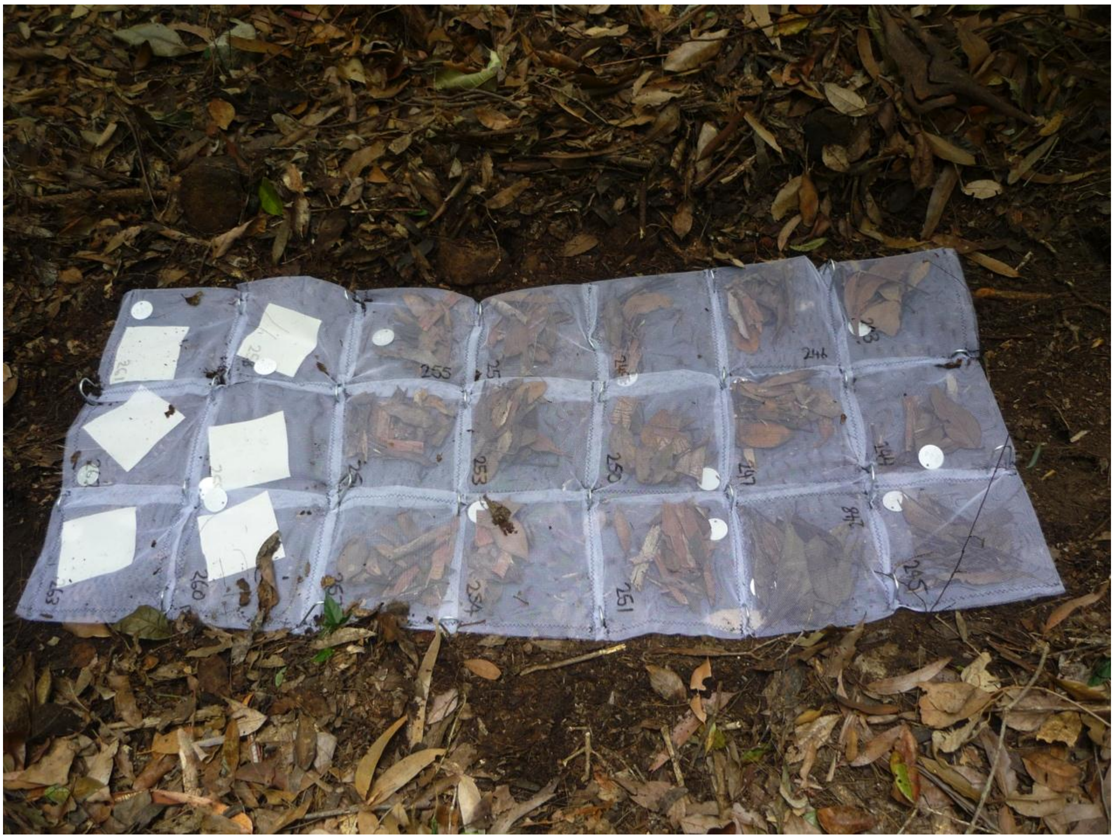

14. References

Bowman, D. M., Williamson, G. J., Keenan, R. J., & Prior, L. D. (2014). A warmer world will reduce tree growth in evergreen broadleaf forests: evidence from Australian temperate and subtropical eucalypt forests. Global Ecology and Biogeography. In Press. DOI 10.1111/geb.12171.

Condit, R., Ashton, P. S., Baker, P., Bunyavejchewin, S., Gunatilleke, S., Gunatilleke, N., ... & Yamakura, T. (2000). Spatial patterns in the distribution of tropical tree species. Science, 288(5470), 1414-1418.

DSE (2011) Strategic plan for implementing a Forests and Parks Monitoring and Reporting Information System in Victoria 2009-2014. Department of Sustainability and Environment, Melbourne, Victoria

GEM: http://gem.tropicalforests.ox.ac.uk/

Lindenmayer, D. (2009) Forest pattern and ecological process: a synthesis of 25 years of research. CSIRO Publishing, Collingwood, Victoria.

McCaw, W. L. (2011). Characteristics of jarrah (Eucalyptus marginata) forest at FORESTCHECK monitoring sites in south-west Western Australia: stand structure, litter, woody debris, soil and foliar nutrients. Australian Forestry,74(4), 254-265.

Moroni, M. T., Kelley, T. H., & McLarin, M. L. (2011). Carbon in trees in Tasmanian State forest. International Journal of Forestry Research, 2010.

Murphy, H. T., Bradford, M. G., Dalongeville, A., Ford, A. J., & Metcalfe, D. J. (2013). No evidence for long-term increases in biomass and stem density in the tropical rain forests of Australia. Journal of Ecology, 101(6), 1589-1597.

Lewis, S.L., Malhi, Y. & Phillips, O.L. (2004a) Fingerprinting the impacts of global change on tropical forests. Philosophical Transactions of the Royal Society of London. Series B: Biological Sciences, 359, 437-462

Lewis, S.L., Phillips, O.L., Sheil, D., Vinceti, B., Baker, T.R., Brown, S., Graham, A.W., Higuchi, N., Hilbert, D.W. & Laurance, W.F. (2004b) Tropical forest tree mortality, recruitment and turnover rates: calculation, interpretation and comparison when census intervals vary. Journal of Ecology, 92, 929-944

Pan, Y., Birdsey, R.A., Fang, J., Houghton, R., Kauppi, P.E., Kurz, W.A., Phillips, O.L., Shvidenko, A., Lewis, S.L. & Canadell, J.G. (2011) A large and persistent carbon sink in the world's forests. Science, 333, 988-993

Phillips, O.L., Lewis, S.L., Baker, T.R., Chao, K.J. & Higuchi, N. (2008) The changing Amazon forest. Philosophical Transactions of the Royal Society B: Biological Sciences, 363, 1819-1827

Phillips, O.L., Arag£O, L.E.O.C., Lewis, S.L., Fisher, J.B., Lloyd, J., Lãpez-Gonzã¡Lez, G., Malhi, Y., Monteagudo, A., Peacock, J. & Quesada, C.A. (2009) Drought sensitivity of the Amazon rainforest. Science, 323, 1344-1347

Prior, L.P., Williamson, G.D. & Bowman, D.M.J.S. (2011) Using permanent forestry plots to understand the possible effects of climate change on Australia's production forest estate. Department of Agriculture, Fisheries and Forestry. Canberra.

Prior, L.P & Bowman, D.M.J.S. (2014) Across a macro-ecological gradient forest competition is strongest at the most productive sites. Frontiers in Plant Science. DOI. 10.3389/fpls.2014.00260.

Prior, L. D., & Bowman, D. M. (2014). Big eucalypts grow more slowly in a warm climate: evidence of an interaction between tree size and temperature.Global Change Biology. In Press. DOI: 10.1111/gcb.12540

RAINFOR: http://www.rainfor.org/

Reich, P. B., & Oleksyn, J. (2008). Climate warming will reduce growth and survival of Scots pine except in the far north. Ecology letters, 11(6), 588-597.

TROBIT: http://www.geog.leeds.ac.uk/groups/trobit/

Turner, P.A.M., Grove, S.J. & Airey, C. (2007) Wildfire chronosequence project establishment report. Bushfire CRC Report No. 7. Canberra.

Ximenes, F.A., George, B.H., Cowie, A., Williams, J. and Kelly, G. (2012) Greenhouse gas balance of native forests in New South Wales, Australia. Forests 3 653-683.

van Mantgem, P.J. & Stephenson, N.L. (2007) Apparent climatically induced increase of tree mortality rates in a temperate forest. Ecology Letters, 10, 909-916

Weiskittel, A. R., Hann, D. W., Kershaw Jr, J. A., & Vanclay, J. K. (2011).Forest growth and yield modeling. John Wiley & Sons.

Wood, M., Keightley, E., Lee, A. & Norman, P. (2006) Continental Forest Monitoring Framework: technical report - design and pilot study. National Forest Inventory, Bureau of Rural Sciences, Canberra.

15. Appendices

15.1. Large Tree Survey Data Sheet (One Page)

15.2. Site Description Data Sheet (Six Pages)

15.3. Voucher Specimen Data Sheet (One Page)

15.4. Soil Metagenomics Data Sheet (One Page)

15.5 Fuel Survey Data Sheet (Two Pages)

LARGE TREE SURVEY DATA SHEET

**Date**:_________________<br>**Site Code**:_____________<br>**Site Name**:____________	**Subplot:**_________________<br>**Start, End Time**:___________<br>**Measurers:**______________	Tree ID: 118, 118a, 118b, D. Species: XXXYYY Dead/Alive: **A**, **D**. ModeDth: **see codes**<br>Growth: **R** Regen, **Y** Regrowth, **M** Mature, **O** Senes, **S1-3** Dead, **S4** Stump<br>Crown: **D** Dom, **C** Co-Dom, **I** Interm, **S** Supress, **E** Emerge, **OG** Open<br>Alive: **A** Alive, **B** Broken, **C** Lean, **D** Fall, **E** Flute, **F** Hollow, **G** Rotten, **H** Multi St, **I** Few leaves, **J** Burnt, **K** Snap, **N** new, **U** Scar, **V** Dead Top **X** Buttress, **Z** decline

Tree ID	Species	Dead Alive?	Alive Status	Mode Death	Growth stage	Crown Class	DBH (cm)	POM (m)	Hgt (m)	Bole (m)	Side (+,-)	X (m)	Y (m)	Comments

SITE DESCRIPTION DATA SHEET

PLOT LOCATION

Site (State and Forest Block): ______________________

AusPlots ID (10 Digit): ____________________________

Dates of Installation: _____________________________

Team Members: ________________________________

GPS COORDINATES OF CORNER POSTS

Walk to each corner post and record the GPS location

'Mark' the Location as a waypoint in the Garmin GPS (e.g) NSFNNC0001-0,0, NSFNNC0001-0,100)

	Easting	Northing	Zone	Datum*	Accuracy	GPS
	(e.g. 470985)	(e.g. 5229220)	(e.g. 55G)	(*)	(e.g. ±12m)	(GPS, diff'tial)
(0,0)						
(0,100)						
(100,0)						
(100,100						

*UTM: WGS84, AGD84, AGD66, GDA94

COMPASS BEARINGS OF X and Y AXES

Bearing (0,0) to (0,100) UP plot:___________________

Bearing (0,0) to (100,0)ACROSS plot:_______________

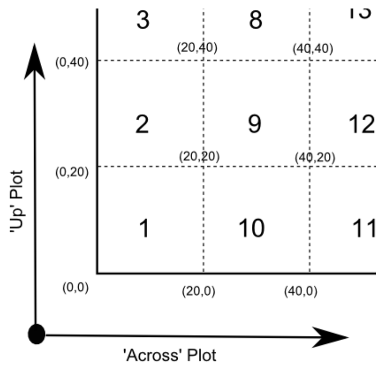

Mud Map of the __Site:__ to contextualise the location of the plot in the landscape and navigation aids to find the plot.

Mud Map of the __Plot:__ show key features of plot (i.e. large trees, logs, water bodies, rock outcrops etc.).To be completed *after* finishing plot subplot by subplot.

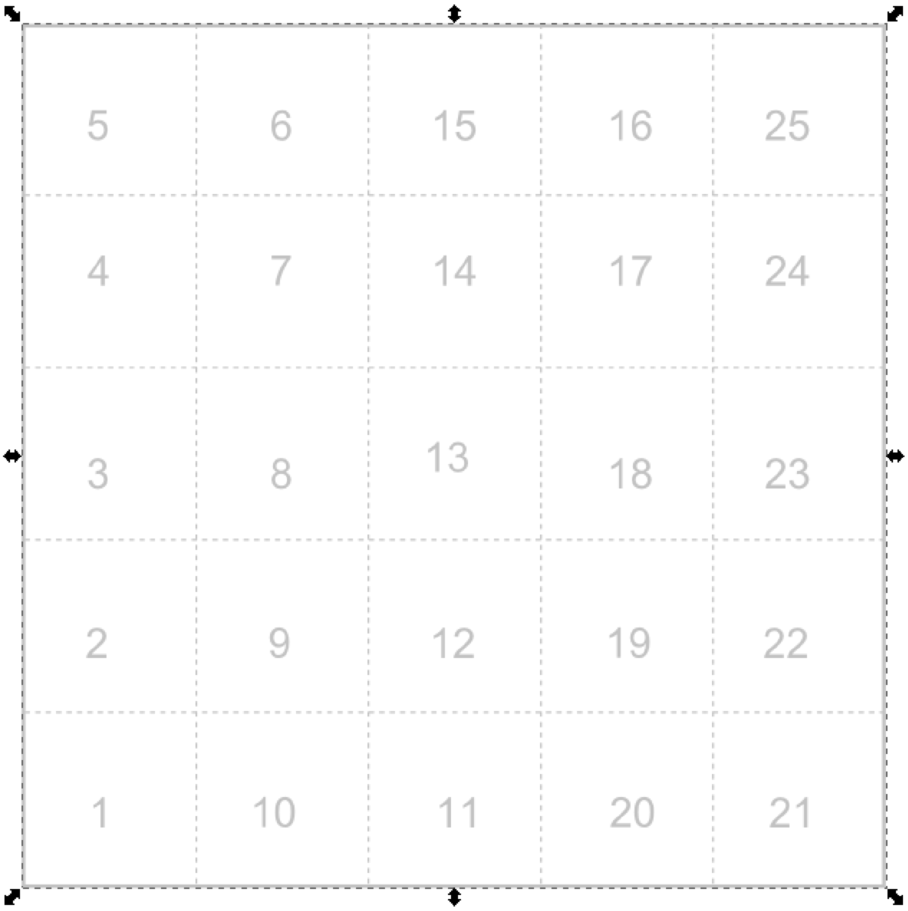

LANDFORM OF 100x100m PLOT

Record the landform attributes of the 100 x 100 plot

Landform Pattern (i.e. low hills, plateau, mountains): ______________________________

Landform Element (i.e. crest, ridge, slope):______________________________________

Slope Class (i.e. level, gently, moderately, steep):________________________________

Slope (degrees): ____________________________

Aspect (degrees): _______________________________

Lithological type (i.e. clay, sand, shale, granite):_________________________________

DISTURBANCE MEASURES

Qualitative assessment of disturbance is done over the whole 100 x 100m plot area..

**Disturbance type** (Refer **Table 8**)	**Severity** - rank its relative severity (from 0 = no discernible disturbance to 3 = severe)	**Time since event** - estimated time since the last event for each disturbance<br>A = < 5 years<br>B = 5-20 years<br>C = > 20 years	**Estimation type**<br>1 = visual estimate<br>2 = historical record<br>3 = informant	**Comments** (ie mean height of fire scars on standing stems)
Wild fire				
Prescribed Burn				
Logging				
Treatment				
Grazing				
Clearing				
Weeds				
Erosion				
Mining/quarries				
Storm				
Salinity				
Other				

Large Tree Survey Species List

Latin Name	Common Name	Code
Eucalyptus obliqua	*Messmate*	*EucObl*

Unidentified Species Notes

Description of Understorey:

Notes:

Notes:

VOUCHER SPECIMEN DATA SHEET

Ausplots ID	Plot name	Date	Voucher ID	Teabag ID	Species/description	Form	Notes	Herbarium ID
VCFSEH0001	*ANU101*	*03/02/14*	*VCA005000*	*VCA005001*	*Long, narrow, discolorous leaves*	*Vine*		*Clematis aristata*

SOIL METAGENOMICS DATA SHEET

Ausplots ID	Plot name	Date collected	Voucher ID	Subplot
VCFSEH0001	*ANU101*	*03/02/14*	*VCA000001*	*1-1*

Site:	Observer 1:	Observer 2:

Transect A	Start:	Start Photo:	Transect Bearing:	Slope:
Date:	End:	End Photo:		Aspect:

	7.0	7.1	7.2	7.3	7.4	7.5	7.6	7.7	7.8
Litter height									
Grass height									
	22.0	21.9	21.8	21.7	21.6	21.5	21.4	21.3	21.2
Litter height									
Grass height									

Woody Fuels				
0.0-0.6 cm	6-7 m:	7-8m:	20-21 m:	21-22 m:
0.6-2.5 cm	6-7 m:	7-8m:	20-21 m:	21-22 m:
2.5-7.6 cm	5-7 m:	7-9 m:	19-21 m:	21-23 m:
>7.6 cm (0-28.3 m): Outside diameter / diameter of every hollow for every intercept, plus sound/rotten (S/R) eg. 20/10S:				

Q1 (7-8m)	% Cover Live	Dead	Fresh weight	Bag weight	Q2 (22-21m)	% Cover Live	Dead	Fresh weight	Bag weight
Litter	N/A				Litter	N/A			
Herbs					Herbs				
Grass					Grass				
Vines					Vines				
Shrubs			N/A	N/A	Shrubs			N/A	N/A
Duff	i		ii		Duff	i		ii	

Measurement of 20 shrubs; 5 shrubs per 7 m sub-transect							
7-0 m density: _______ m x _______ m				14-7 m density: _______ m x _______ m			
	Lifeform	Height	DBH		Lifeform	Height	DBH
1				6			
2				7			
3				8			
4				9			
5				10			
21-14 m density: _______ m x _______ m				28-21 m density: _______ m x _______ m			
	Lifeform	Height	DBH		Lifeform	Height	DBH
11				16			
12				17			
13				18			
14				19			
15				20			

Transect B	Start:	Start Photo:	Transect Bearing:	Slope:
Date:	End:	End Photo:		Aspect:

	7.0	7.1	7.2	7.3	7.4	7.5	7.6	7.7	7.8
Litter height									
Grass height									
	22.0	21.9	21.8	21.7	21.6	21.5	21.4	21.3	21.2
Litter height									
Grass height									

Woody Fuels				
0.0-0.6 cm	6-7 m:	7-8m:	20-21 m:	21-22 m:
0.6-2.5 cm	6-7 m:	7-8m:	20-21 m:	21-22 m:
2.5-7.6 cm	5-7 m:	7-9 m:	19-21 m:	21-23 m:
>7.6 cm (0-28.3 m): Outside diameter / diameter of every hollow for every intercept, plus sound/rotten (S/R) eg. 20/10S:				

Q1 (7-8m)	% Cover Live	Dead	Fresh weight	Bag weight	Q2 (22-21m)	% Cover Live	Dead	Fresh weight	Bag weight
Litter	N/A				Litter	N/A			
Herbs					Herbs				
Grass					Grass				
Vines					Vines				
Shrubs			N/A	N/A	Shrubs			N/A	N/A
Duff	i		ii		Duff	i		ii	

Measurement of 20 shrubs; 5 shrubs per 7 m sub-transect							
7-0 m density: _______ m x _______ m				14-7 m density: _______ m x _______ m			
	Lifeform	Height	DBH		Lifeform	Height	DBH
1				6			
2				7			
3				8			
4				9			
5				10			
21-14 m density: _______ m x _______ m				28-21 m density: _______ m x _______ m			
	Lifeform	Height	DBH		Lifeform	Height	DBH
11				16			
12				17			
13				18			
14				19			
15				20			

**Moisture content samples**	**Fresh weight**	**Dry weight**	**Bag type**
Litter i			
Litter ii			
Grass			
Herbs			
Vines			

Litter decomposition bags

**Type**	**ID**	**Sample weight**	**Total weight**
**Litter**			
**Litter**			
**Litter**			
**Litter**			
**Litter**			
**Litter**			
**Litter**			
**Litter**			
**Litter**			
**Litter**			
**Litter**			
**Litter**			
**Litter**			
**Litter**			
**Litter**			
**Standard**			
**Standard**			
**Standard**			
**Standard**			
**Standard**			
**Standard**			

Transect C	Start:	Start Photo:	Transect Bearing:	Slope:
Date:	End:	End Photo:		Aspect:

	7.0	7.1	7.2	7.3	7.4	7.5	7.6	7.7	7.8
Litter height									
Grass height									
	22.0	21.9	21.8	21.7	21.6	21.5	21.4	21.3	21.2
Litter height									
Grass height									

Woody Fuels				
0.0-0.6 cm	6-7 m:	7-8m:	20-21 m:	21-22 m:
0.6-2.5 cm	6-7 m:	7-8m:	20-21 m:	21-22 m:
2.5-7.6 cm	5-7 m:	7-9 m:	19-21 m:	21-23 m:

>7.6 cm (0-28.3 m): Outside diameter / diameter of every hollow for every intercept, plus sound/rotten (S/R) eg. 20/10S:

Q1 (7-8m)	% Cover		Fresh weight	Bag weight	Q2 (22-21m)	% Cover		Fresh weight	Bag weight
	Live	Dead				Live	Dead		
Litter:	N/A				Litter:	N/A			
Herbs:					Herbs:				
Grass:					Grass:				
Vines:					Vines:				
Shrubs:			N/A	N/A	Shrubs:			N/A	N/A
Duff	i		ii		Duff:	i		ii	

Measurement of 20 shrubs; 5 shrubs per 7 m sub-transect

7-0 m density: _______ m x _______ m				14-7 m density: _______ m x _______ m			
	Lifeform	Height	DBH		Lifeform	Height	DBH
1				6			
2				7			
3				8			
4				9			
5				10			
21-14 m density: _______ m x _______ m				28-21 m density: _______ m x _______ m			
	Lifeform	Height	DBH		Lifeform	Height	DBH
11				16			
12				17			
13				18			
14				19			
15				20			

Transect D	Start:	Start Photo:	Transect Bearing:	Slope:
Date:	End:	End Photo:		Aspect:

	7.0	7.1	7.2	7.3	7.4	7.5	7.6	7.7	7.8
Litter height									
Grass height									
	22.0	21.9	21.8	21.7	21.6	21.5	21.4	21.3	21.2
Litter height									
Grass height									

Woody Fuels				
0.0-0.6 cm	6-7 m:	7-8m:	20-21 m:	21-22 m:
0.6-2.5 cm	6-7 m:	7-8m:	20-21 m:	21-22 m:
2.5-7.6 cm	5-7 m:	7-9 m:	19-21 m:	21-23 m:

>7.6 cm (0-28.3 m): Outside diameter / diameter of every hollow for every intercept, plus sound/rotten (S/R) eg. 20/10S:

Q1 (7-8m)	% Cover		Fresh weight	Bag weight	Q2 (22-21m)	% Cover		Fresh weight	Bag weight
	Live	Dead				Live	Dead		
Litter:	N/A				Litter:	N/A			
Herbs:					Herbs:				
Grass:					Grass:				
Vines:					Vines:				
Shrubs:			N/A	N/A	Shrubs:			N/A	N/A
Duff	i		ii		Duff:	i		ii	

Measurement of 20 shrubs; 5 shrubs per 7 m sub-transect

7-0 m density: _______ m x _______ m				14-7 m density: _______ m x _______ m			
	Lifeform	Height	DBH		Lifeform	Height	DBH
1				6			
2				7			
3				8			
4				9			
5				10			
21-14 m density: _______ m x _______ m				28-21 m density: _______ m x _______ m			
	Lifeform	Height	DBH		Lifeform	Height	DBH
11				16			
12				17			
13				18			
14				19			
15				20			
