# Supplementary material for: Macroecology of Australian Tall Eucalypt Forests: Baseline Data from a Continental-Scale Permanent Plot Network
Source: PLoS One. 2015 Sep 14;10(9):e0137811. doi: 10.1371/journal.pone.0137811 (PMC4569531; doi:10.1371/journal.pone.0137811)

Ausplots Forest Monitoring Network

Plot Establishment Report

Version 1.0.

Sam Wood, Helen Stephens, David Bowman.

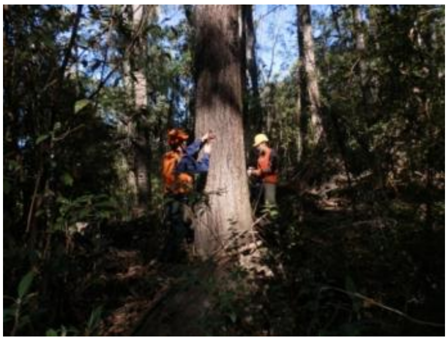

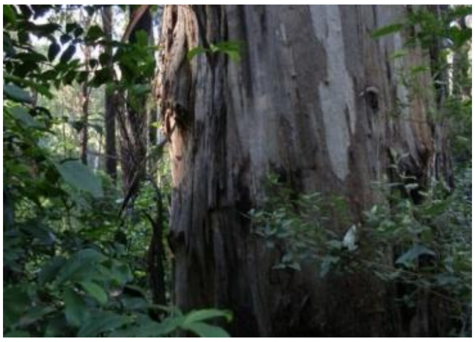

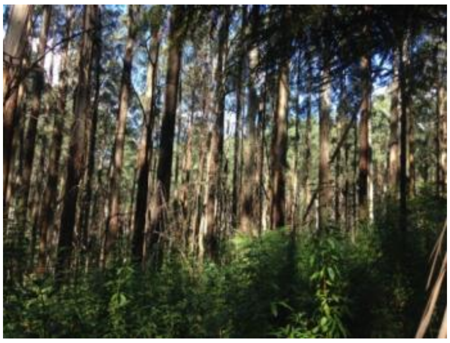

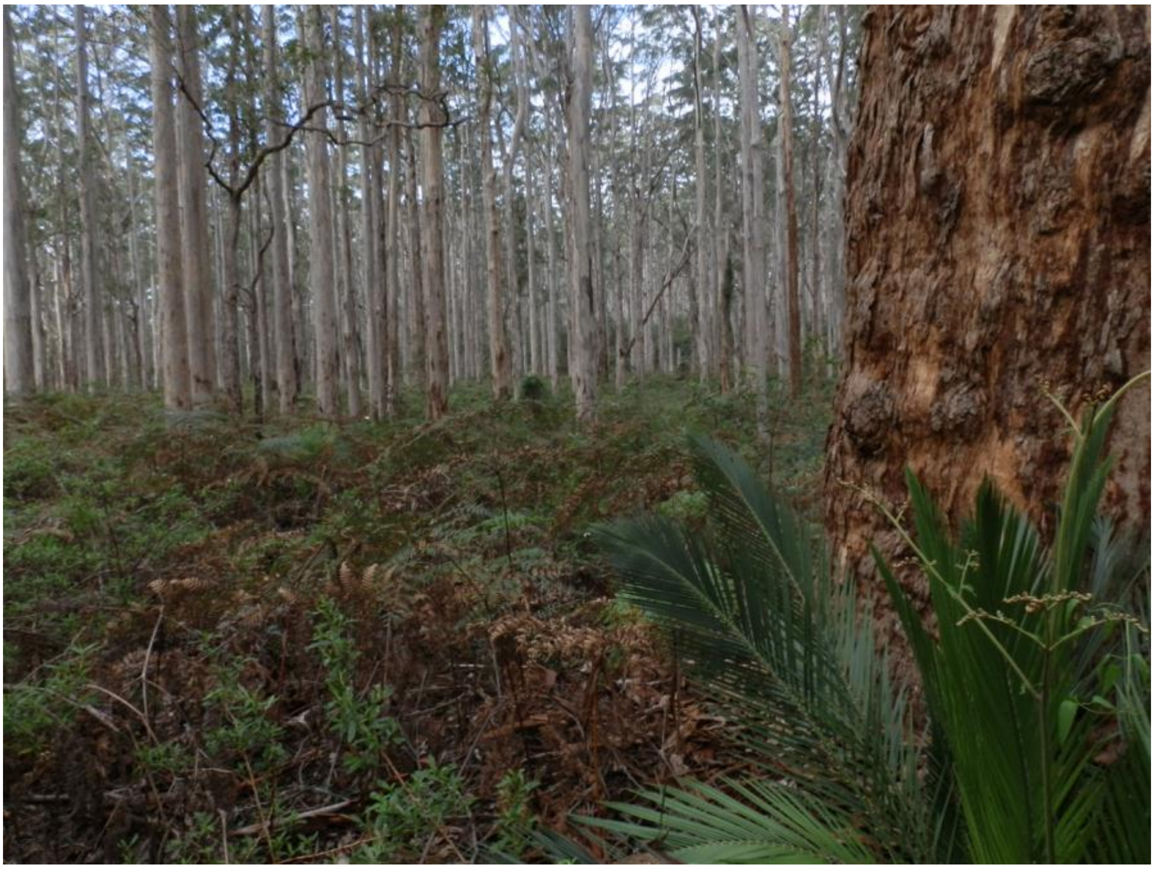

Ausplots Forests

School of Biological Sciences

University of Tasmania

Private Bag 55

Hobart, Tasmania, 7001

www.tern.org.au/AusPlots

Terrestrial Ecosystem Research Network

University of Adelaide

Level 12 Schulz Building

North Terrace Campus

Adelaide, South Australia 5005

www.tern.org.au

Publication details

This edition of the TERN AusPlots Forests Site Establishment Report was produced and published by the University of Tasmania. It is provided as an online resource. Please do not republish material from this document or its attachments without written consent.

Table of Contents

Section	Page
**Title Pages**	1-3
**Introduction**	4
**Acknowledgements**	5
**Overview Map**	6
**Western Australia (WAF)**	7
Map	8
Plot Descriptions (n=9)	9-26
**Victoria (VCF)**	27
Map	28
Plot Descriptions (n=8)	29-44
**Southern NSW (NSF)**	45
Map	46
Plot Descriptions (n=5)	47-56
**Northern NSW (NNC)**	57
Map	58
Plot Descriptions (n=8)	59-74
**Far North Queensland (QDF)**	75
Map	76
Plot Descriptions (n=4)	77-84
**Tasmania (TCF)**	85
Map	86
Plot Descriptions (n=14)	87-114

Introduction

As of March 2015, the Ausplots Forest Monitoring Network has established 48 permanent plots in tall eucalypt forests cross the continent of Australia. This document (a) compiles information on the location and configuration of each plot and (b) presents basic stand structural information. The intention of the document is to compile all the information required as background for data analysis, to facilitate the re-measurement of plots and encourage ecological studies using the plot network.

Complementary information on the rationale, experimental design, baseline data, methodology and protocols for the Ausplots Forest Monitoring project can be found in the following:

- *Wood, S.W, Stephens, H., Foulkes, J. and Bowman, D.M.J.S.B. (2014) AusPlots Forests Survey Protocols Manual, Version 1.5. available at www.tern.org.au/ausplots*
- *Wood, S.W., Stephens, H., Prior, L., Bowman, D.M.J.S.B. (in prep) Macroecology of Australian tall eucalypt forests: baseline data from a continental scale permanent plot network. Forest Ecology and Management.*

Notes:

- Tree Ferns (e.g. *Dicksonia* spp. and *Cyathea* spp.) were not measured consistently across sites and were removed from the dataset before analysis.
- Basal Area, and histograms and stem maps were calculated from diameter measurements at 1.3m, with the exception of (a) trees measured above a buttress or (b) deformed trees at 1.3m.
- Only live trees were included.
- No. stems includes all individual stems from multi-stemmed trees and may overestimate individual tree numbers.
- Unidentified species in the field are noted with the prefix UNN. Unidentified species from Northern New South Wales are currently being identified by local herbaria using voucher specimens.
- In this document “Eucalypt” includes all species from the genus *Eucalyptus* and closely related genus *Corymbia*, *Syncarpia* and *Lophostemon*.
- MAT = Mean Annual Temperature; MAP = Mean Annual Precipitaiton

The establishment and measurement of the Ausplots Forest Monitoring Network would not have been possible without the hard work of the field crew:

Sam Wood (Leader)

Helen Stephens (Leader)

Dom Neyland (Leader)

Tom Baker

Matt Bradford

Jenny Calder

James da Costa

Grant Daniels

Elinor Ebsworth

Sarah Fayed

Nick Fountain-Jones

Clare Hadrill

Rowena Hamer

Kaely Kreger

Matt Larcombe

Michael Lee

Harry McDermott

Scott Nichols

Chris Obst

Jeremy O'Wheel

Euan Rose

Jen Sanger

David Tng

Carly Ward

Ausplots Forest Monitoring Network

Overview Map

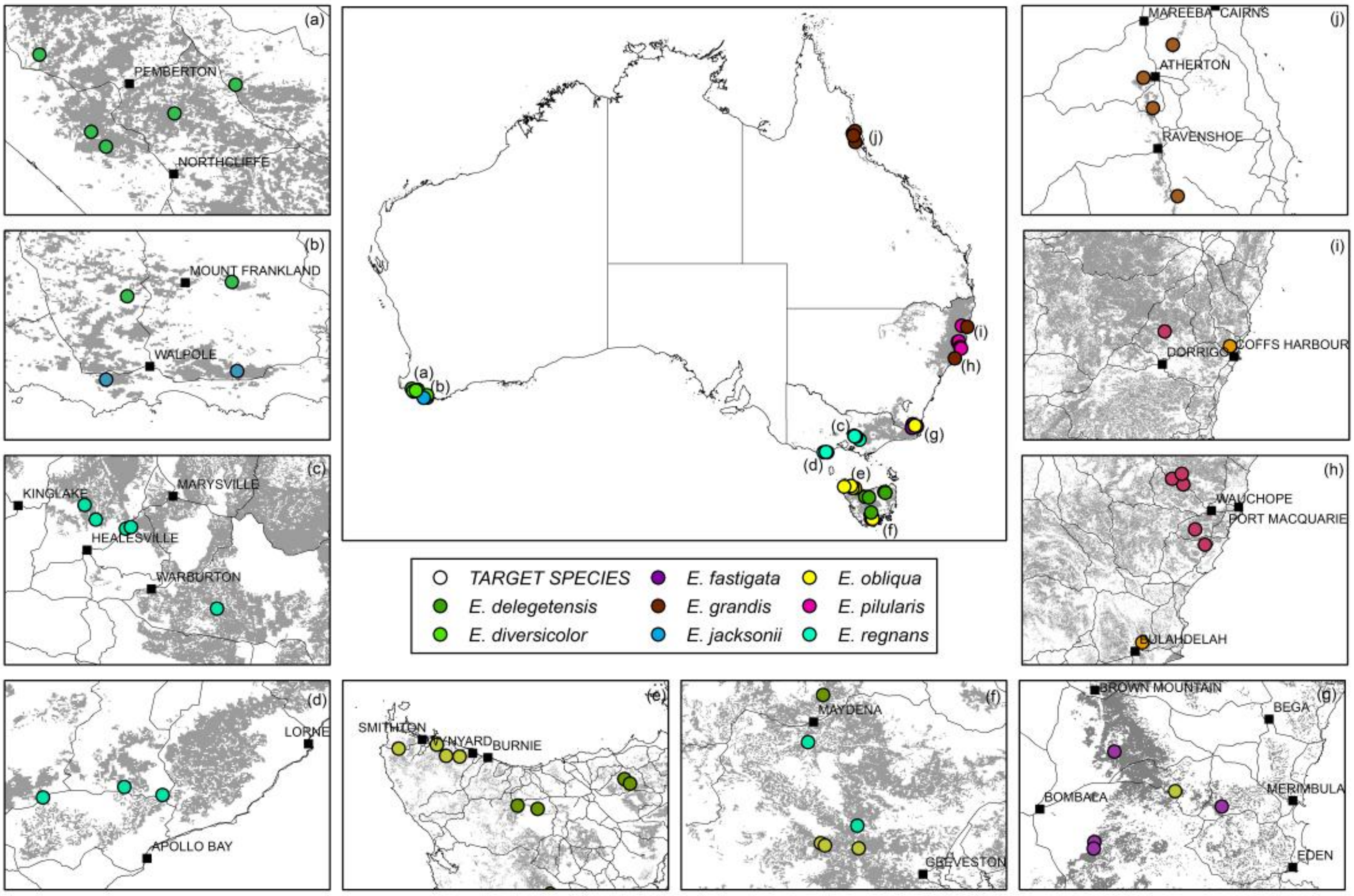

Ausplots Forest Monitoring Network

Summary Table

AusPlotID	Bioregion	Plot Name	Tenure	Long/Lat	Census Date	Plot size	Dominant Species
WAFWAR0001	Warren	Carey	Beedelup NP	115.8457 -34.3866	Sep 2012	1.0	*E. diversicolor*
WAFWAR0002	Warren	Dombakup	Forest Reserve	115.9829 -34.5764	Sep 2012	1.0	*E. diversicolor*
WAFWAR0003	Warren	Warren	Warren NP	115.9522 -34.5459	Sep 2012	1.0	*E. diversicolor*
WAFWAR0004	Warren	Dawson	Forest Reserve	116.6872 -34.8497	Sep 2012	1.0	*E. diversicolor*
WAFWAR0005	Warren	Giants	Walpole NP	116.8793 -34.9805	Oct 2012	1.0	*E. jacksonii*
WAFWAR0006	Warren	Sutton	National Park	116.2498 -34.4488	Nov 2012	1.0	*E. diversicolor*
WAFWAR0007	Warren	Mt Frankland	Mt Frankland NP	116.8734 -34.8247	Nov 2012	1.0	*E. diversicolor*
WAFWAR0008	Warren	Mt Clare	Walpole NP	116.6504 -34.9954	Nov 2012	1.0	*E. jacksonii*
WAFWAR0009	Warren	Collins	Forest Reserve	116.1242 -34.5078	Nov 2012	1.0	*E. diversicolor*
VCFSEH0001	SE Highlands	Toolangi	ANU Reserve	145.5167 -37.5308	Feb 2014	1.0	*E. regnans*
VCFSEH0002	SE Highlands	Ada	ANU Reserve	145.8672 -37.8049	Feb 2014	1.0	*E. regnans*
VCFSEH0003	SE Highlands	Weeaproinah	Otways NP	143.4749 -38.6437	Mar 2014	1.0	*E. regnans*
VCFSEH0004	SE Highlands	Turtons Rd	Otways NP	143.7014 -38.6390	Mar 2014	1.0	*E. regnans*
VCFSEH0005	SE Highlands	Lardners Rd	Otways NP	143.6290 -38.6238	Mar 2014	1.0	*E. regnans*
VCFSEH0006	SE Highlands	Black Spur	Maroondah NP	145.6263 -37.5936	Apr 2014	1.0	*E. regnans*
VCFSEH0007	SE Highlands	Monda Rd	Maroondah NP	145.6396 -37.5893	Apr 2014	1.0	*E. regnans*
VCFSEH0008	SE Highlands	HardyCreek	Forest Reserve	145.5465 -37.5693	Apr 2014	1.0	*E. regnans*
NSFSEC0001	SE Corner	Newline	SE Forests NP	149.4351 -36.7591	May 2014	1.0	*E. fastigata*
NSFSEC0002	SE Corner	Waratah Mix	SE Forests NP	149.3821 -36.9969	May 2014	1.0	*E. fastigata*
NSFSEC0003	SE Corner	Wog Way	SE Forests NP	149.3808 -37.0140	May 2014	1.0	*E. fastigata*
NSFSEC0004	SE Corner	Goodenia	SE Forests NP	149.7176 -36.9035	May 2014	1.0	*E. fastigata*
NSFSEC0005	SE Corner	Candelo	SE Forests NP	149.5949 -36.8631	May 2014	1.0	*E. fastigata*
NSFNNC0001	NSW Nth Coast	Mines Rd	Mines Rd FR	152.5368 -31.2803	Sep 2013	1.0	*E. pilularis*
NSFNNC0002	NSW Nth Coast	A-Tree	Willi Willi NP	152.4609 -31.2421	Sep 2013	1.0	*E. pilularis*
NSFNNC0003	NSW Nth Coast	Tinebank	Willi Willi NP	152.5267 -31.2086	Oct 2013	1.0	*E. pilularis*
NSFNNC0004	NSW Nth Coast	Lorne	Bago Bluff NP	152.6164 -31.5828	Oct 2013	1.0	*E. pilularis*
NSFNNC0005	NSW Nth Coast	Bird Tree	Middle Brother NP	152.6825 -31.6858	Nov 2013	1.0	*E. pilularis*
NSFNNC0006	NSW Nth Coast	Black Bull	Nymboi-Bderay NP	152.7270 -30.1578	Nov 2013	1.0	*E. pilularis*
NSFNNC0007	NSW Nth Coast	Bruxner	Bruxner FR	153.0918 -30.2401	Nov 2013	1.0	*E. grandis*
NSFNNC0008	NSW Nth Coast	OSullivans	Myall Lakes NP	152.2605 -32.3455	Nov 2013	1.0	*E. grandis*
QDWET0001	Wet Tropics	Mt Baldy	Baldy Mtn FR	145.4187 -17.2698	Aug 2014	1.0	*E. grandis*
QDWET0002	Wet Tropics	Longlands	Herberton R. NP	145.5609 -17.1107	Aug 2014	1.0	*E. grandis*
QDWET0003	Wet Tropics	Lamb Range	Danbulla NP	145.4644 -17.4158	Aug 2014	1.0	*E. grandis*
QDWET0004	Wet Tropics	Koombaloomba	Koombaloomba NP	145.5843 -17.8416	Aug 2014	1.0	*E. grandis*
TCFTNS001	Tas. Nth Slopes	Flowerdale	Flowerdale FR	145.5661 -41.0449	Mar 2012	1.0	*E. obliqua*
TCFTNS002	Tas. Nth Slopes	Dip River	Dip River FR	145.4055 -41.0345	Apr 2012	1.0	*E. obliqua*
TCFTNS003	Tas. Nth Slopes	McKenzie	TFA Future Reserve	146.2593 -41.6303	Jan 2015	1.0	*E. delegatensis*
TCFTNS004	Tas. Nth Slopes	Caveside	TFA Future Reserve	146.5007 -41.6697	Feb 2015	1.0	*E. delegatensis*
TCFTSR001	Tas. Sth Ranges	Bird Track	Warra FR	146.6435 -43.0891	Jan 2012	1.0	*E. obliqua*
TCFTSR002	Tas. Sth Ranges	Supersite	Warra FR	146.6534 -43.0959	Apr 2012	1.6	*E. obliqua*
TCFTSR003	Tas. Sth Ranges	Nth Styx	North Styx FR	146.6083 -42.8118	Apr 2013	1.0	*E. regnans*
TCFTSR004	Tas. Sth Ranges	Weld R.	Weld FR	146.7435 -43.0411	Apr 2013	1.0	*E. regnans*
TCFTSR005	Tas. Sth Ranges	Arve V.	Arve FR	146.7472 -43.1028	May 2013	1.0	*E. obliqua*
TCFTSR006	Tas. Sth Ranges	Mt Field	Mt Field NP	146.6492 -42.6829	Dec 2014	1.0	*E. delegatensis*
TCFKIN001	King	Black River	TFA Future Reserve	154.2852 -40.9525	Nov 2014	1.0	*E. obliqua*
TCFKIN002	King	Bond Tier	Regional Reserve	144.8420 -40.9526	Nov 2014	1.0	*E. obliqua*
TCFBEL001	Ben Lomond	Mt Maurice	TFA Future Reserve	147.5383 -41.3113	Dec 2014	1.0	*E. delegatensis*
TCFBEL002	Ben Lomond	Ben Ridge	TFA Future Reserve	147.6025 -41.3673	Jan 2015	1.0	*E. delegatensis*

Western Australia (WAF)

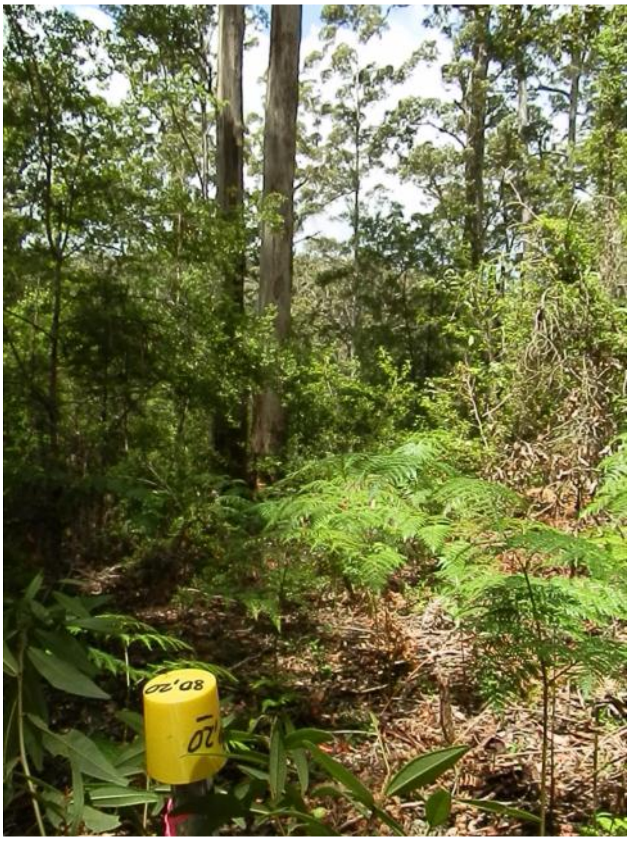

Eucalyptus diversicolor at WAFWAR009 (Collins)

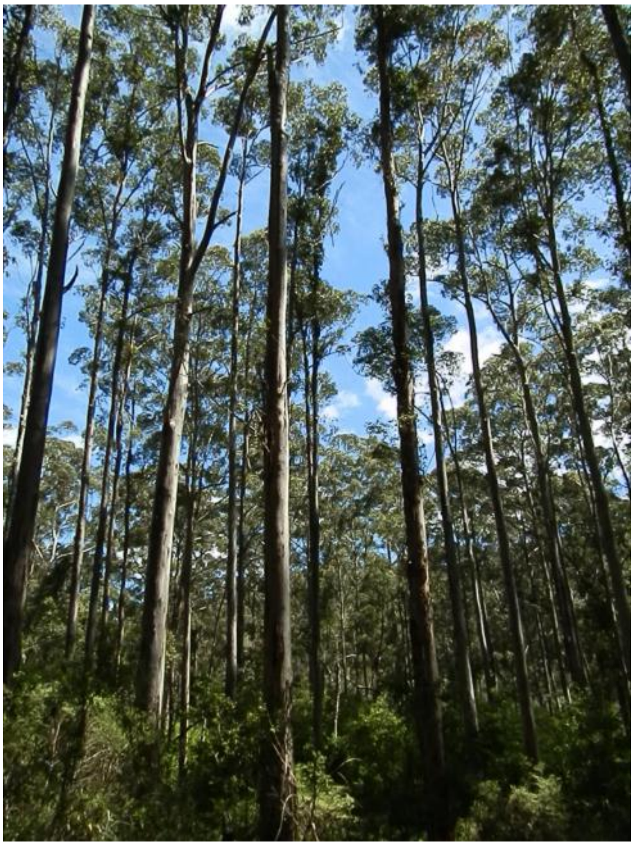

Eucalyptus diversicolor at WAFWAR006 (Sutton)

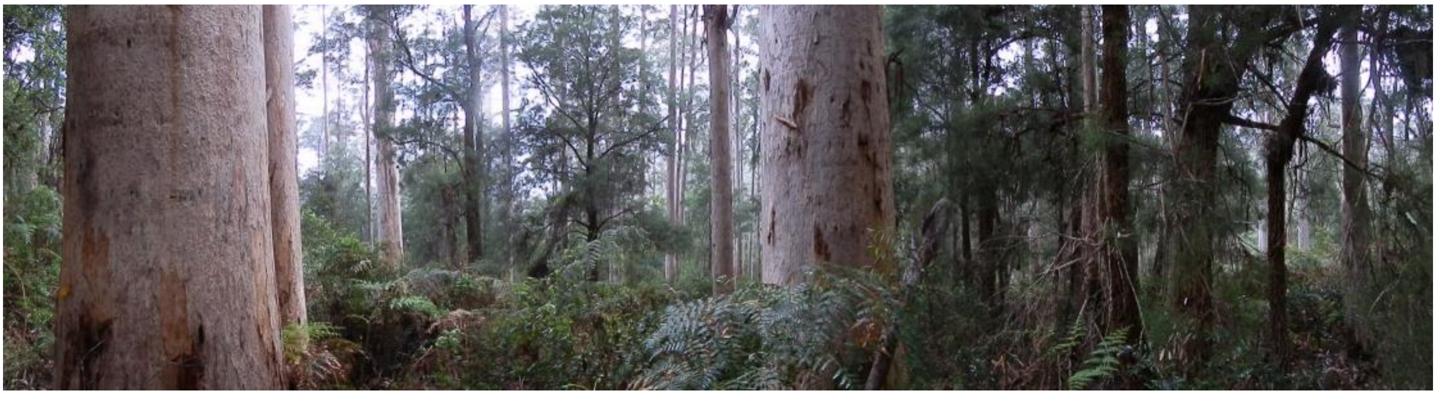

Eucalyptus diversicolor at WAFWAR002 (Dombakup)

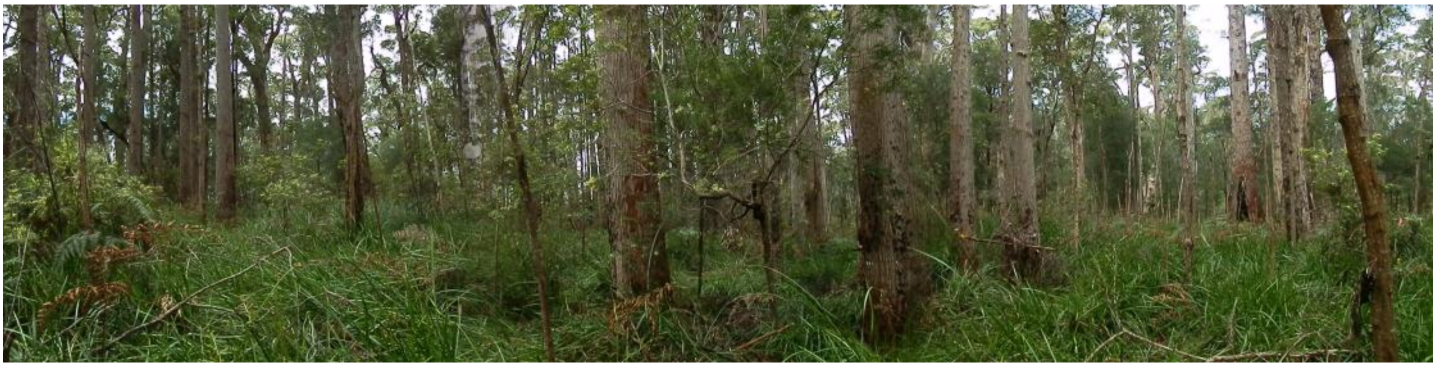

Eucalyptus jacksonii at WAFWAR005 (Giants)

Western Australia (WAF)

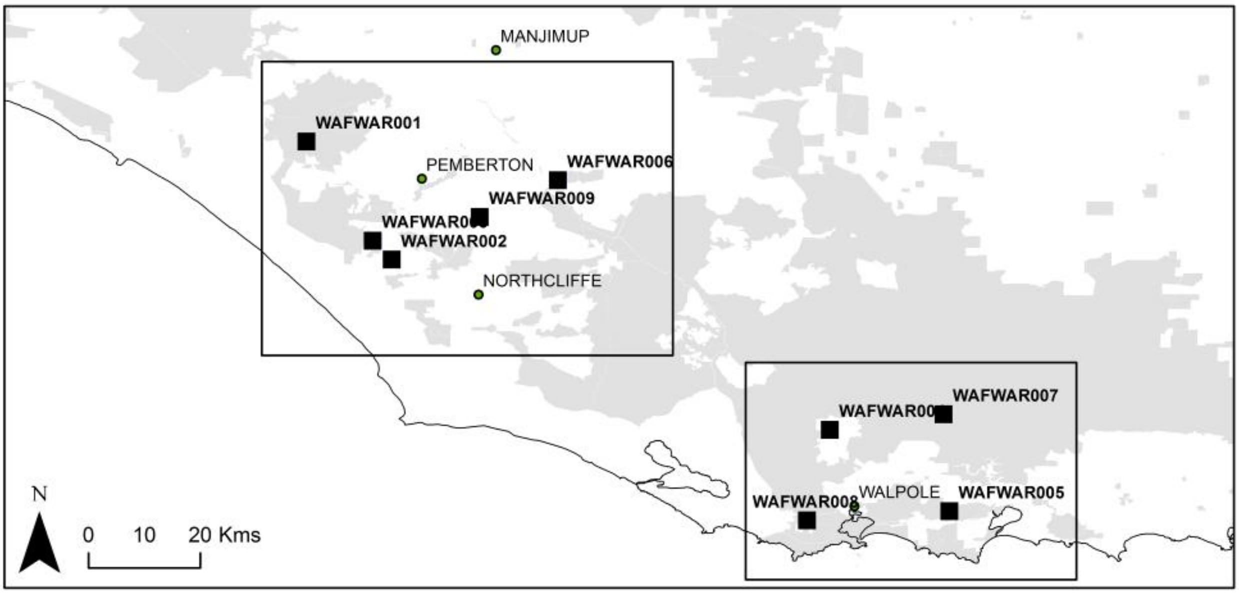

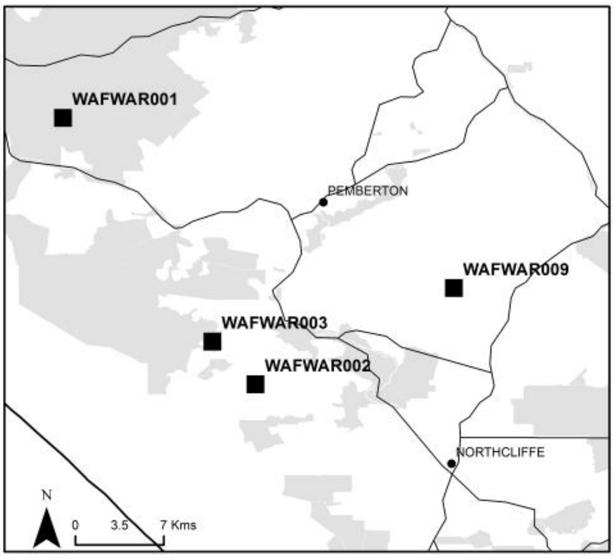

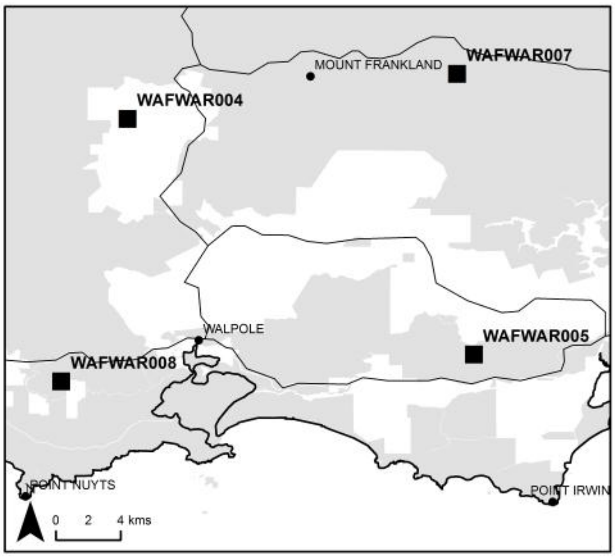

WAFWAR001: Carey

**AusPlot ID**	WAFWAR001	**Elevation**	164m
**AusPlot Name**	Carey	**Aspect**	187°
**State**	Western Australia	**Slope**	8°; Gently Inclined
**Bioregion**	Warren	**Landform Element**	Midslope
**Location (UTM)**	50 H 393886 6194367	**MAT, MAP**	15.3 ^{o}C, 1098 mm
**Location (Lat/Long)**	-34.3866 115.8457	**Existing Plot Custodian**	WA Department of Parks and Wildlife
**Tenure**	Beedelup National Park	**Existing Plot ID**	Permanent Inventory Plot 899
**Plot Est. Date**	12 September 2012	**Existing Plot Area**	0.64ha (80mx80m)
**Plot Size**	1.0ha (100mx100m)	**Existing Plot Census**	1982,1987,1992,2000

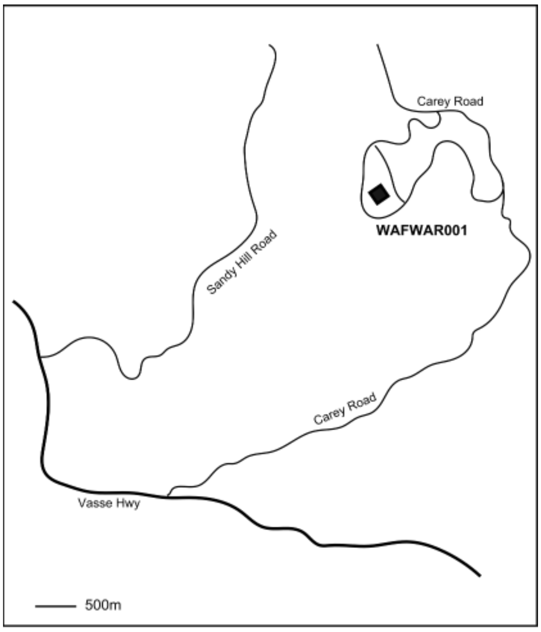

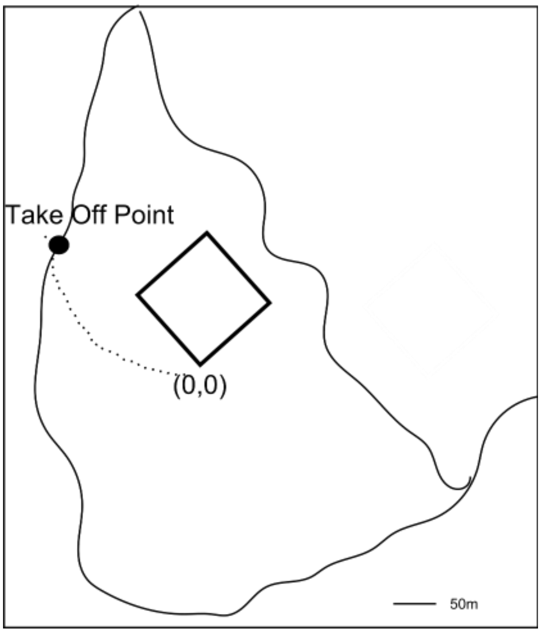

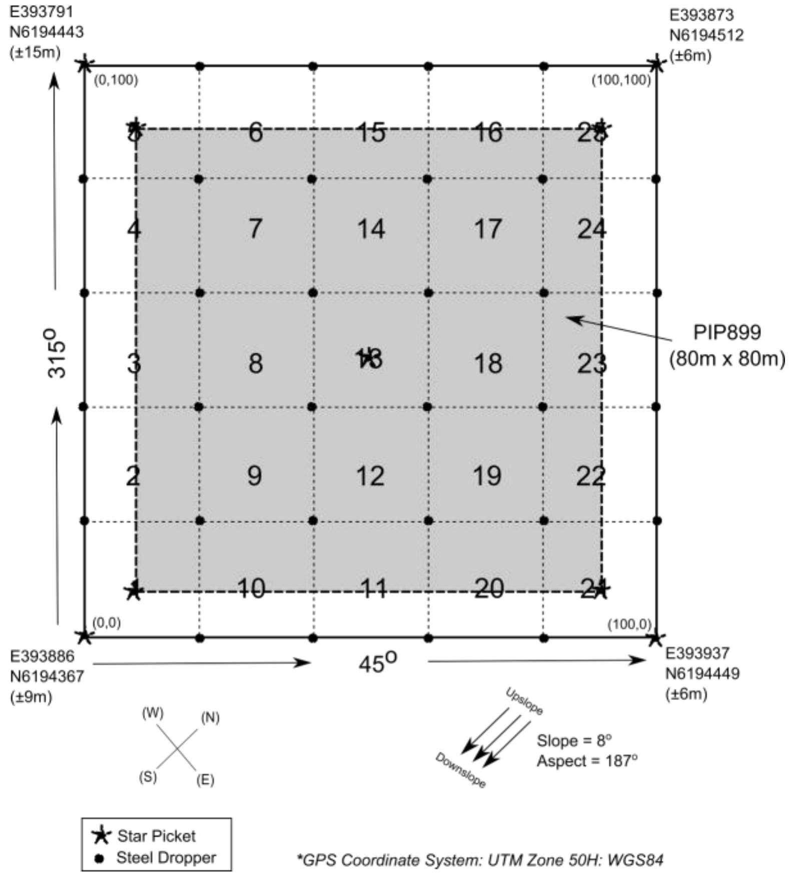

WAFWAR001: Carey

**Target Eucalypt Species:**	*Eucalyptus diversicolor*	**High severity fire?**	Yes, 1852 (PIP899 Ring Counts)
**Maximum Tree Height (m)**	76m	**Low severity fire?**	Yes, frequency unknown (Fire Scars)
**Target Species Growth Stage:**	Mature, 1852 regrowth	**Cut stumps?**	No
**Understorey:**	Wet Sclerophyll	**Other Disturbance?**	Clearing some understorey for PIP899

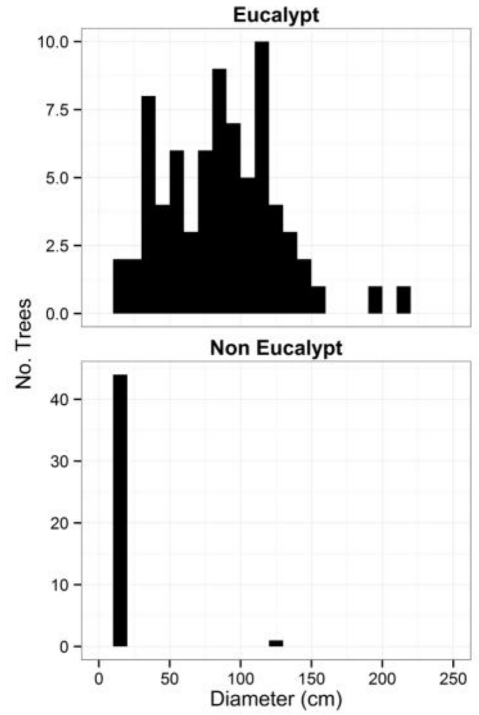

Species	No. Stems	BA (m^2/ha)
Eucalyptus diversicolor	58	49.9
Corymbia calophylla	16	2.1
Trymalium odoratissimum	45	1.7

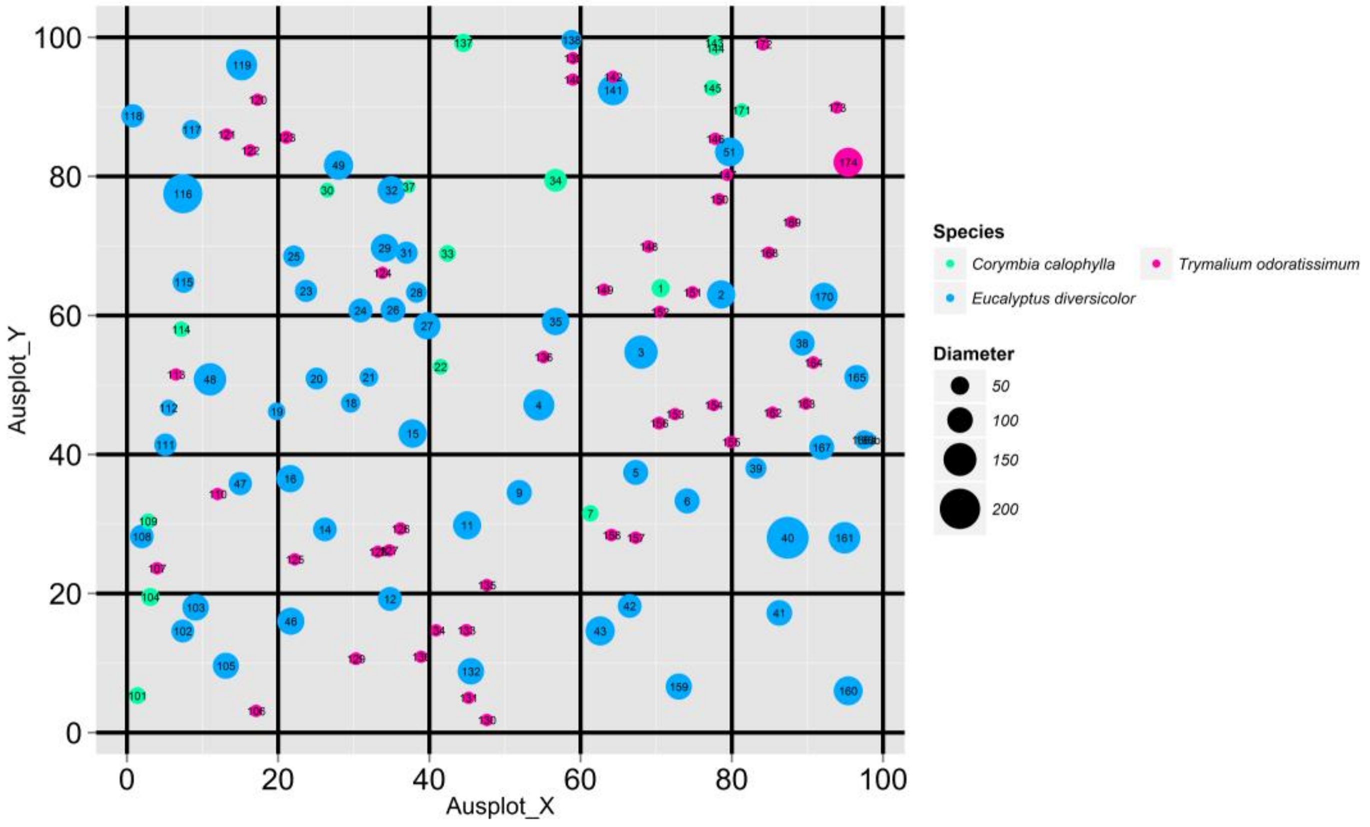

WAFWAR002: Dombakup

**AusPlot ID**	WAFWAR002	**Elevation**	93m
**AusPlot Name**	Dombakup	**Aspect**	180°
**State**	Western Australia	**Slope**	10°; Moderate
**Bioregion**	Warren	**Landform Element**	Midslope
**Location (UTM)**	50 H 406758 6173380	**MAT, MAP**	15.2 ^{o}C, 1163 mm
**Location (Lat/Long)**	-34.5764 115.9829	**Existing Plot Custodian**	WA Department of Parks and Wildlife
**Tenure**	Forest Reserve	**Existing Plot ID**	Permanent Inventory Plot 882
**Plot Est. Date**	18 September 2012	**Existing Plot Area**	0.49ha (70mx70m)
**Plot Size**	1.0ha (100mx100m)	**Existing Plot Census**	1981,1986,1991,1999

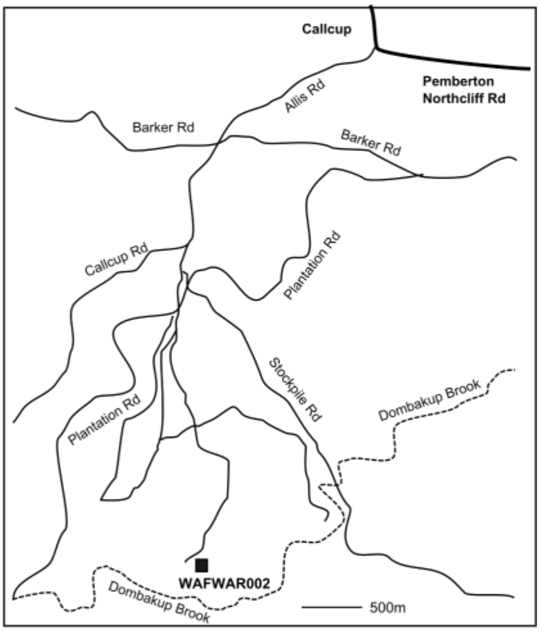

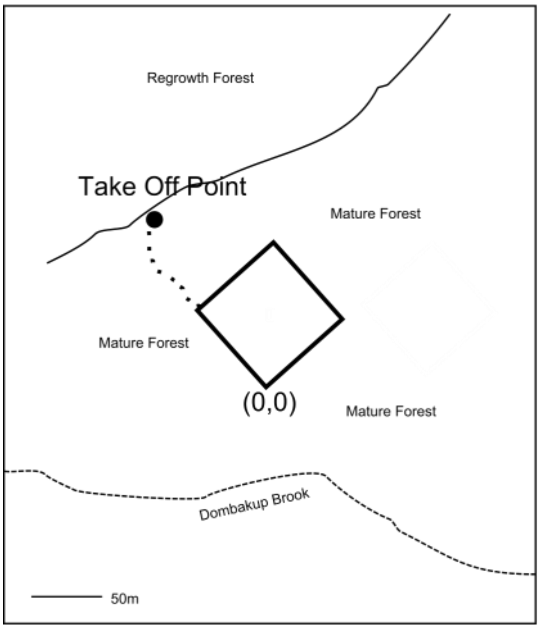

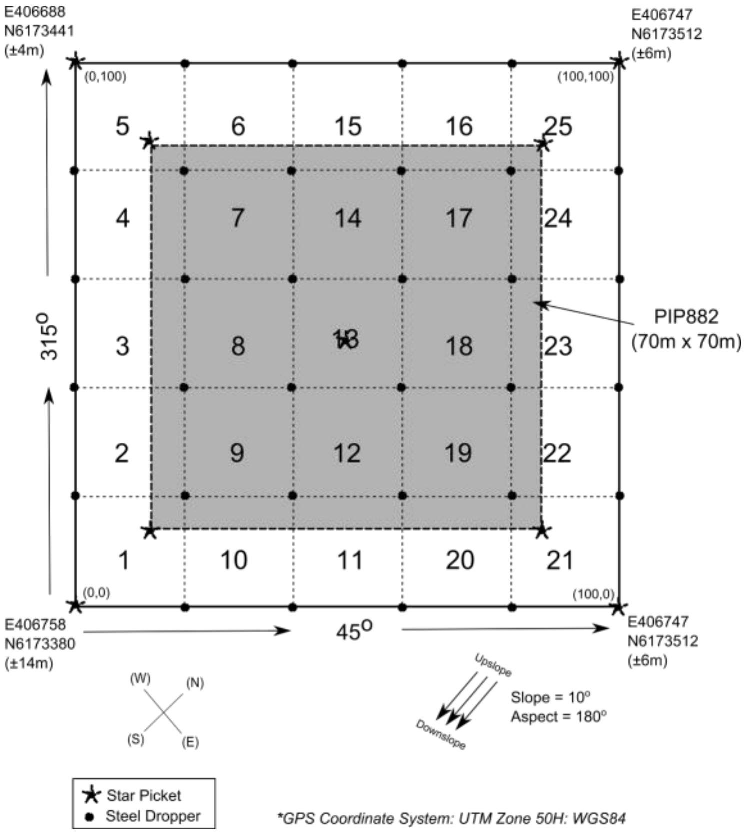

WAFWAR002: Dombakup

**Target Eucalypt Species:**	*Eucalyptus diversicolor*	**High severity fire?**	Yes, 1857 (PIP882 Ring Counts)
**Maximum Tree Height (m)**	68m	**Low severity fire?**	Yes, frequency unknown (Fire Scars)
**Target Species Growth Stage:**	Mature, 1857 regrowth	**Cut stumps?**	No
**Understorey:**	Wet Sclerophyll	**Other Disturbance?**	Clearing some understorey for PIP882

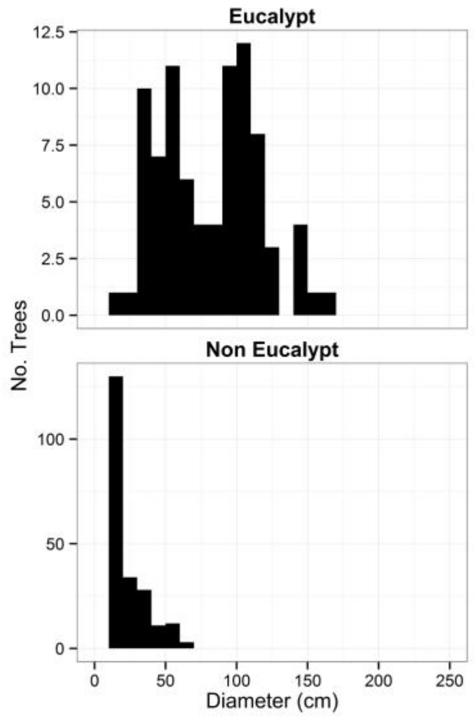

Species	No. Stems	BA (m^2/ha)
Eucalyptus diversicolor	82	51.1
Allocasuarina decussata	131	10.4
Trymalium odoratissimum	86	1.4
Agonis flexuosa	1	0.1
Corymbia calophylla	2	0.1

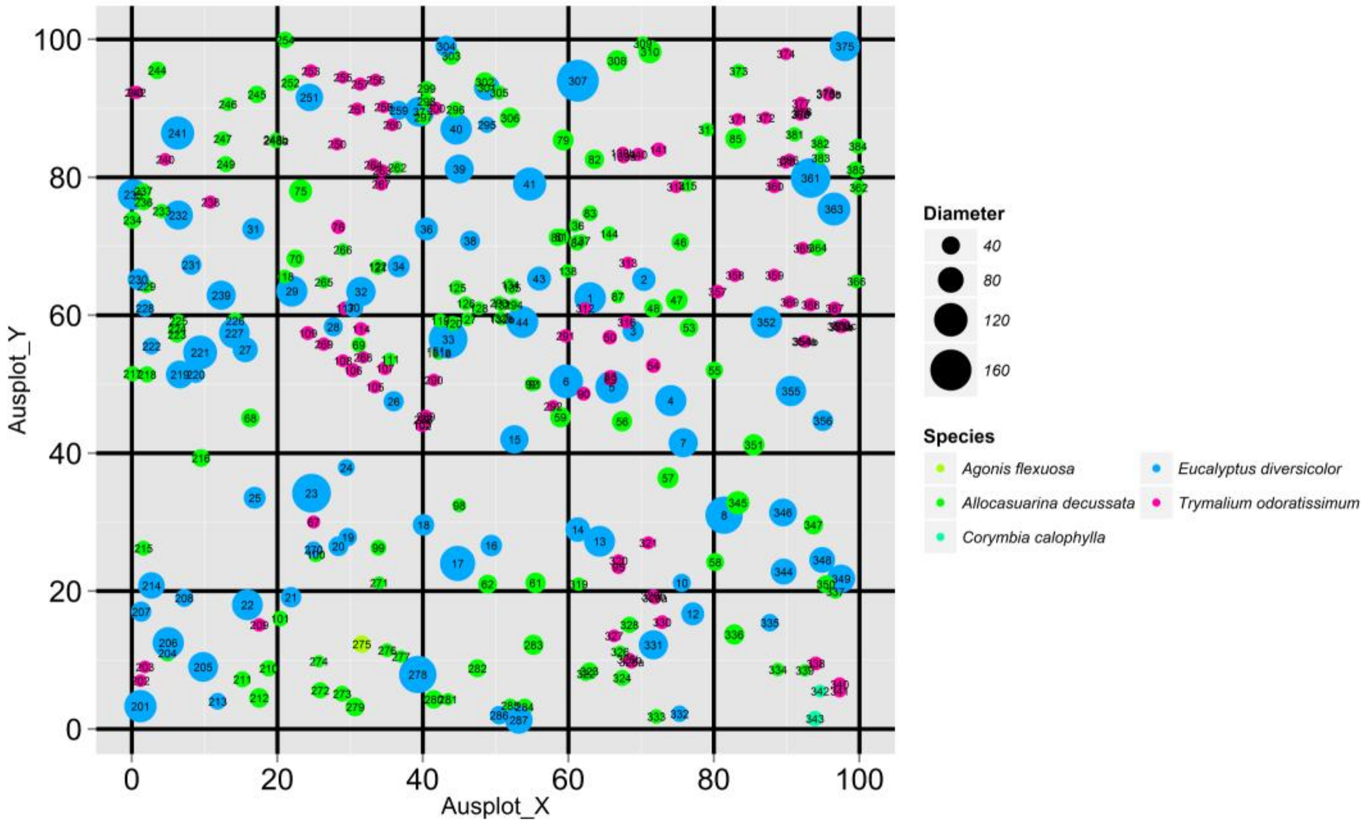

WAFWAR003: Warren

**AusPlot ID**	WAFWAR003	**Elevation**	134m
**AusPlot Name**	Warren	**Aspect**	314°
**State**	Western Australia	**Slope**	14°; Moderate
**Bioregion**	Warren	**Landform Element**	Midslope
**Location (UTM)**	50 H 403860 6176806	**MAT, MAP**	14.8 ^{o}C, 1175 mm
**Location (Lat/ Long)**	-34.5459 115.9522	**Existing Plot Custodian**	WA Department of Parks and Wildlife
**Tenure**	Proposed National Park	**Existing Plot ID**	Permanent Inventory Plot 873
**Plot Est. Date**	23 September 2012	**Existing Plot Area**	0.49ha (70mx70m)
**Plot Size**	1.0ha (100mx100m)	**Existing Plot Census**	1981,1986,1991,1999,2007

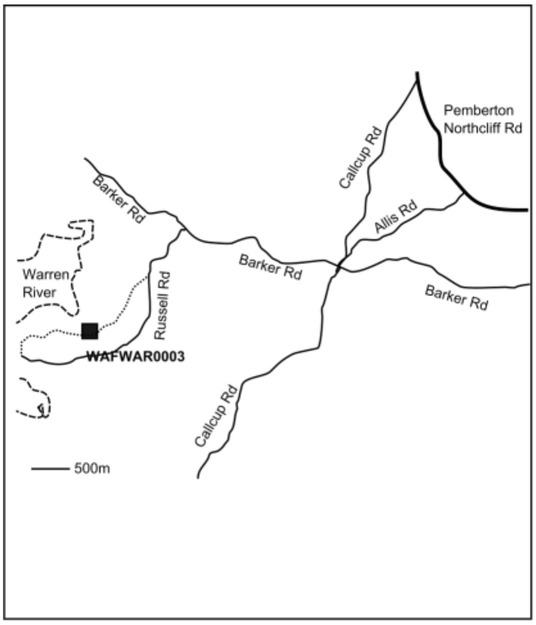

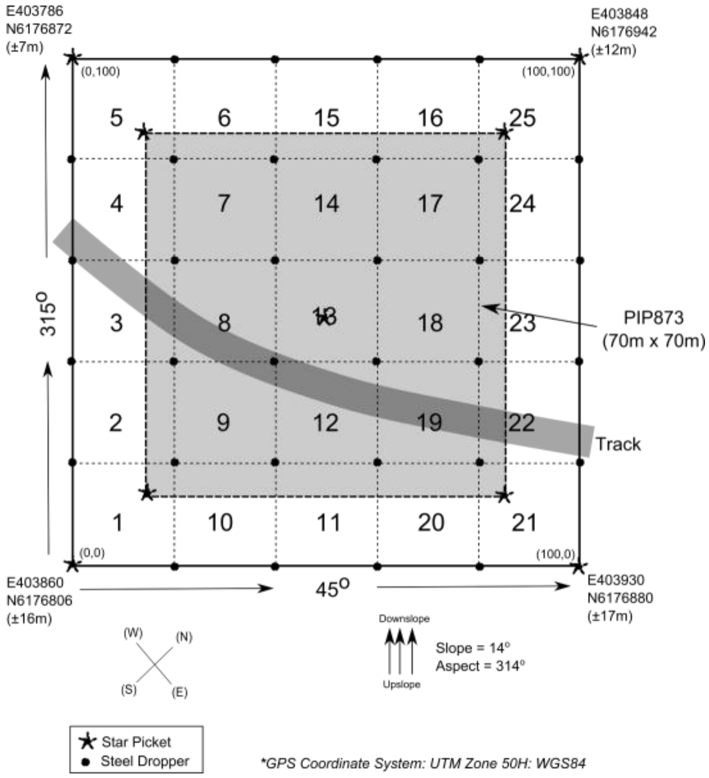

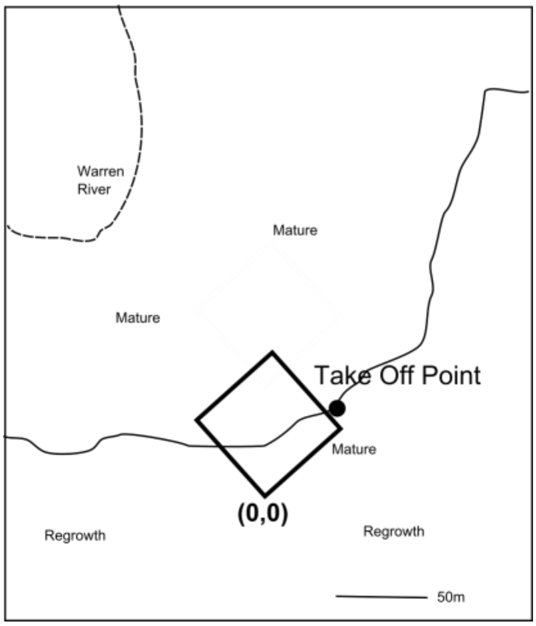

WAFWAR003: Warren

**Target Eucalypt Species:**	*Eucalyptus diversicolor*	**High severity fire?**	Yes, 1854 (PIP893 Ring Counts)
**Maximum Tree Height (m)**	65m	**Low severity fire?**	Yes, frequency unknown (Fire Scars)
**Target Species Growth Stage:**	Mature, 1857 regrowth	**Cut stumps?**	No
**Understorey:**	Wet Sclerophyll	**Other Disturbance?**	Clearing some understorey for PIP893
			Track through southern half of plot.

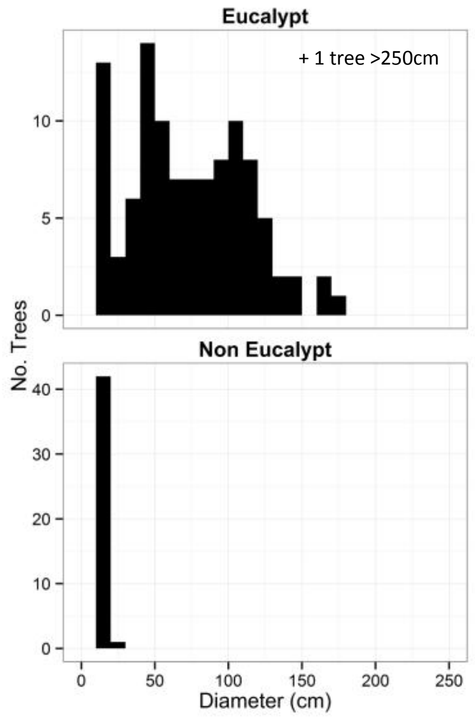

Species	No. Stems	BA (m^2/ha)
Eucalyptus diversicolor	106	61.3
Trymalium odoratissimum	42	0.5
Agonis flexuosa	1	<0.1

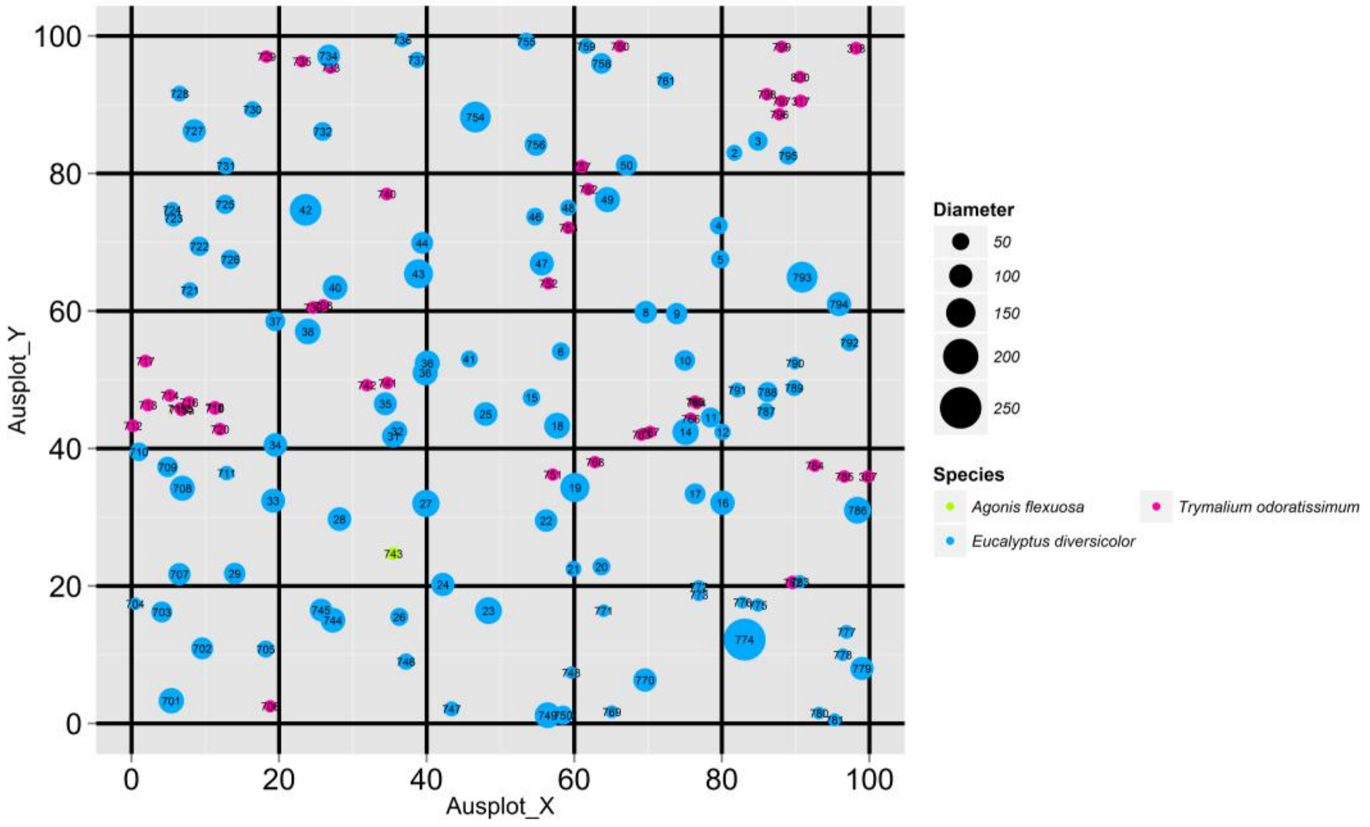

WAFWAR004: Dawson

**AusPlot ID**	WAFWAR004	**Elevation**	148m
**AusPlot Name**	Dawson	**Aspect**	67°
**State**	Western Australia	**Slope**	Gently Inclined
**Bioregion**	Warren	**Landform Element**	Upper Slope
**Location (UTM)**	50 H 471405 6143570	**MAT, MAP**	15.2 °C, 1105 mm
**Location (Lat/Long)**	-34.8497 116.6872	**Existing Plot Custodian**	WA Department of Parks and Wildlife
**Tenure**	Forest Reserve	**Existing Plot ID**	Permanent Inventory Plot 893
**Plot Est. Date**	27 September 2012	**Existing Plot Area**	0.16ha (40mx40m)
**Plot Size**	1.0ha (100mx100m)	**Existing Plot Census**	1982,1987,1995,2005

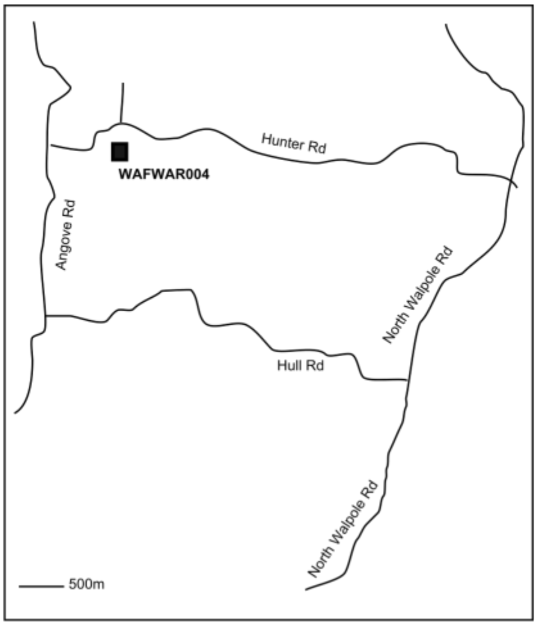

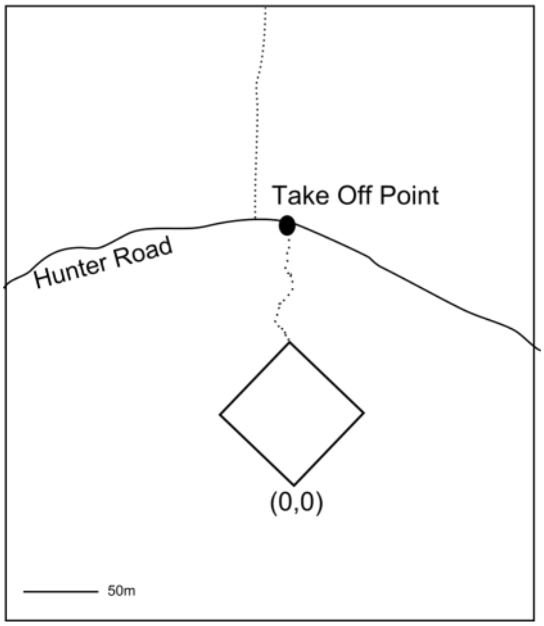

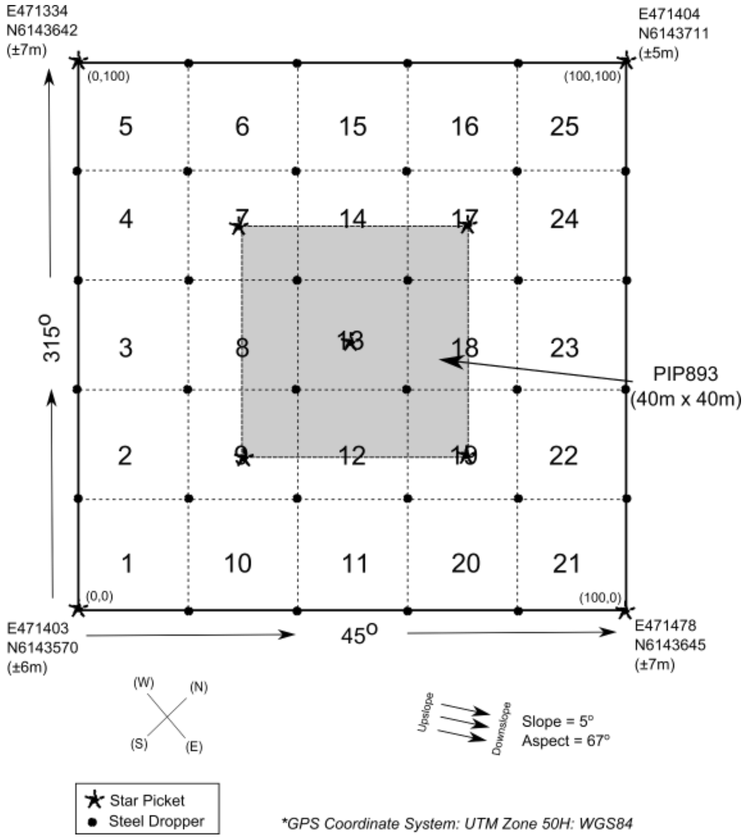

WAFWAR004: Dawson

**Target Eucalypt Species:**	*Eucalyptus diversicolor*	**High severity fire?**	Yes, 1937 (Known Fire Event)
**Maximum Tree Height (m)**	55m	**Low severity fire?**	Yes, frequency unknown (Fire Scars)
			Last fire approx. 2011-12
**Target Species Growth Stage:**	Mature, 1937 regrowth	**Cut stumps?**	No
**Understorey:**	Wet Sclerophyll	**Other Disturbance?**	Clearing some understorey for PIP873

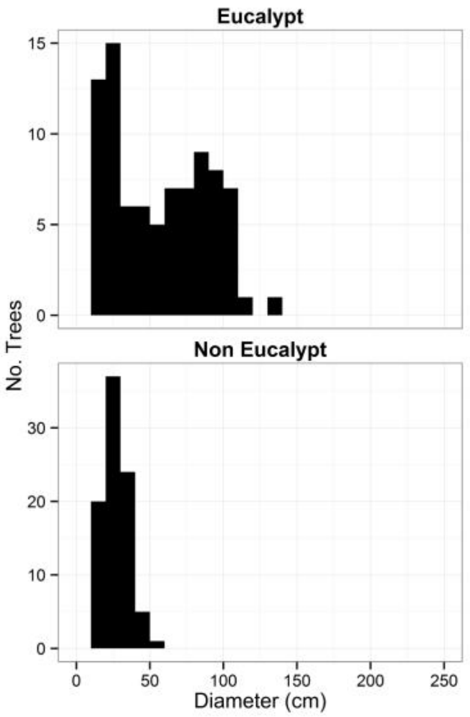

Species	No. Stems	BA (m^2/ha)
Eucalyptus diversicolor	83	28.8
Allocasuarina decussata	84	5.5
Corymbia calophylla	2	0.1
Trymalium odoratissimum	3	<0.1

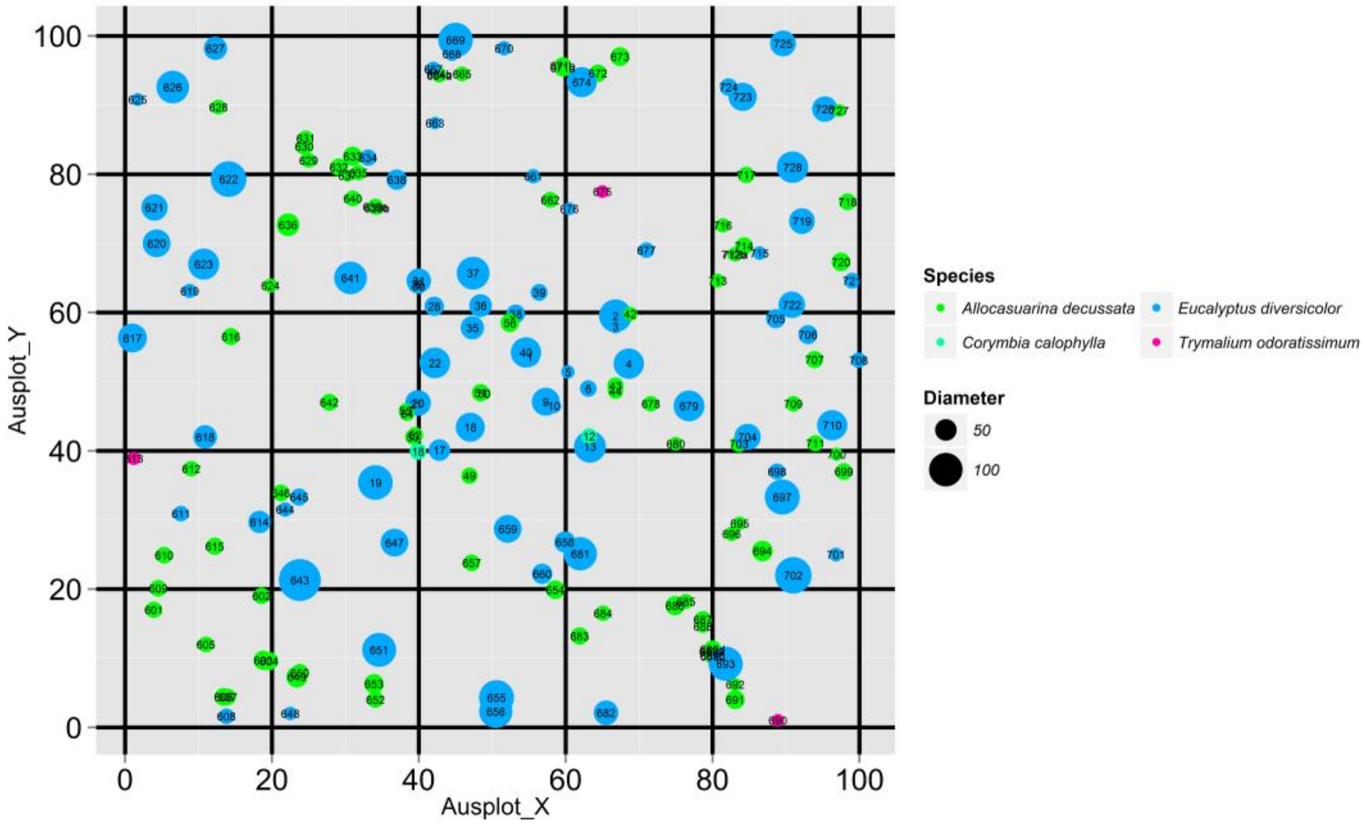

WAFWAR005: Giants

**AusPlot ID**	WAFWAR005	**Elevation**	158m
**AusPlot Name**	Giants	**Aspect**	180°
**State**	Western Australia	**Slope**	8°; Gently Inclined
**Bioregion**	Warren	**Landform Element**	Mid Slope
**Location (UTM)**	50 H 488986 6129108	**MAT, MAP**	15.1 ^{o}C, 1132 mm
**Location (Lat/Long)**	-34.9805 116.8793	**Existing Plot Custodian**	WA Department of Parks and Wildlife
**Tenure**	Walpole National Park	**Existing Plot ID**	Permanent Inventory Plot 639
**Plot Est. Date**	01 October 2012	**Existing Plot Area**	0.2ha (20mx100m)
**Plot Size**	1.0ha (100mx100m)	**Existing Plot Census**	1964,1972,1981,1986,1991,1999

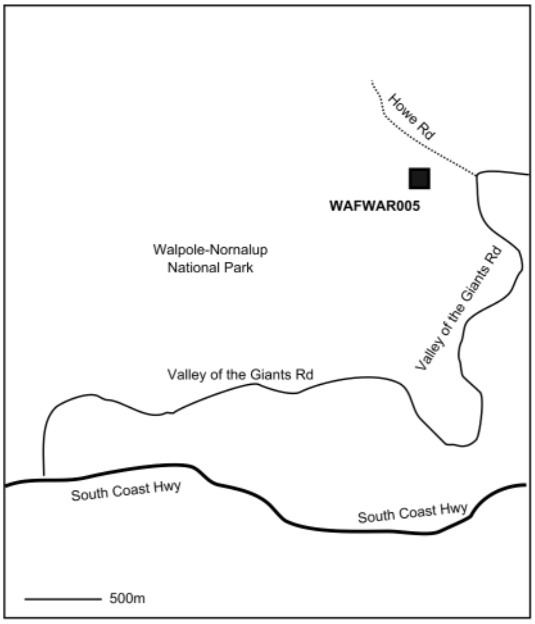

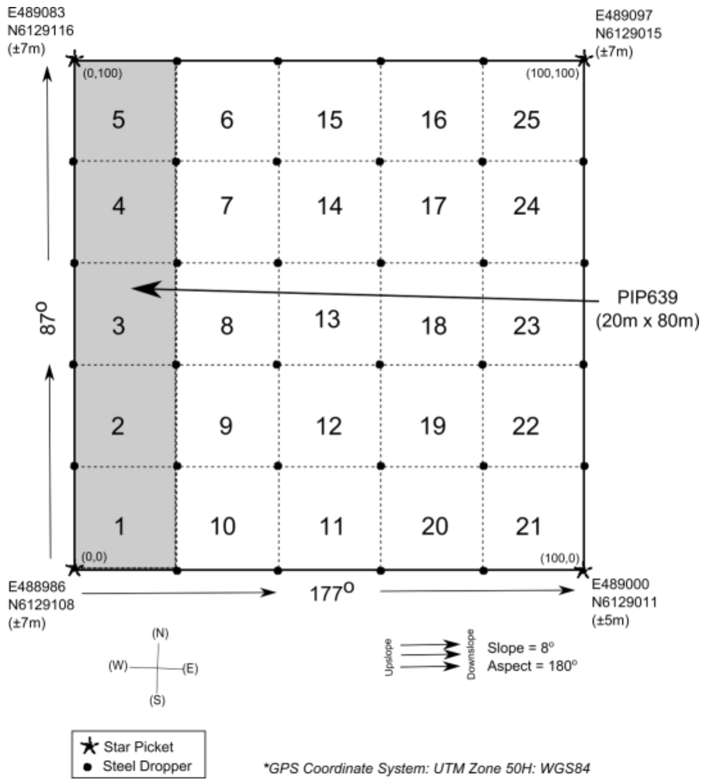

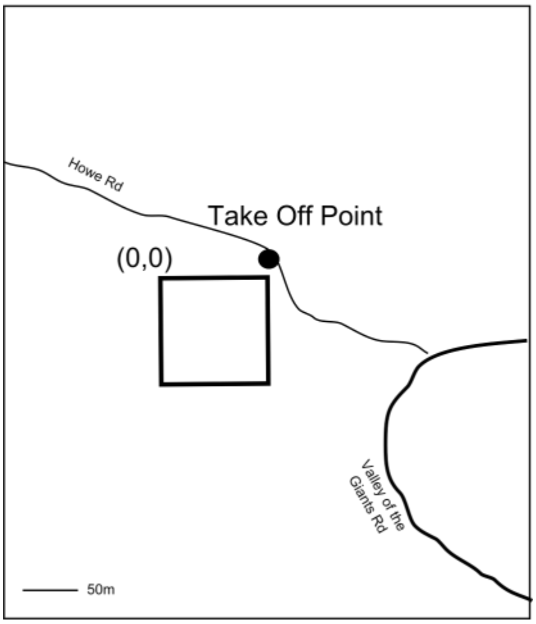

WAFWAR005: Giants

**Target Eucalypt Species:**	*Eucalyptus jacksonii*	**High severity fire?**	Yes, 1937 (Known Fire Event) in plots 1-5
**Maximum Tree Height (m)**	52m	**Low severity fire?**	Yes, frequency unknown (Fire Scars)
**Target Species Growth Stage:**	Old-Growth (Plots 6-25) + 1937 regrowth (Plots 1-5)	**Cut stumps?**	No
**Understorey:**	Wet Sclerophyll	**Other Disturbance?**	No

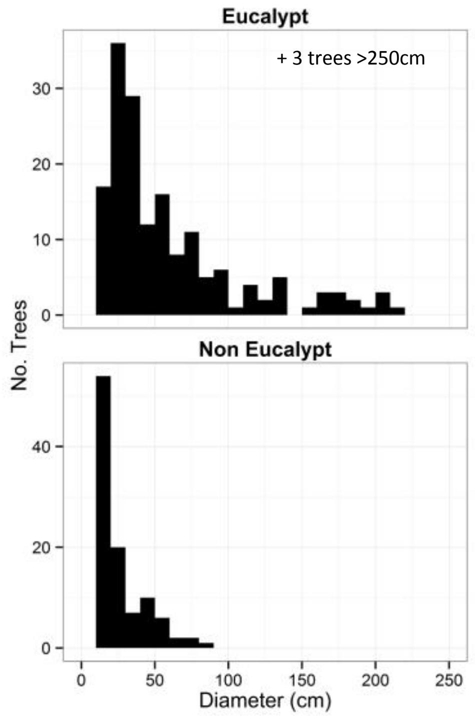

Note: Old Growth Eucalyptus jacksonii *trees at this site form pronounced buttresses and are likely to introduce considerable uncertainty into stand structural calculations based on diameter measurements.*

Species	No. Stems	BA (m^2/ha)
Eucalyptus jacksonii	151	68.4*
Eucalyptus diversicolor	8	10.1
Allocasuarina decussata	101	7.6
Corymbia calophylla	10	2.4
Acacia pentadenia	1	<0.1

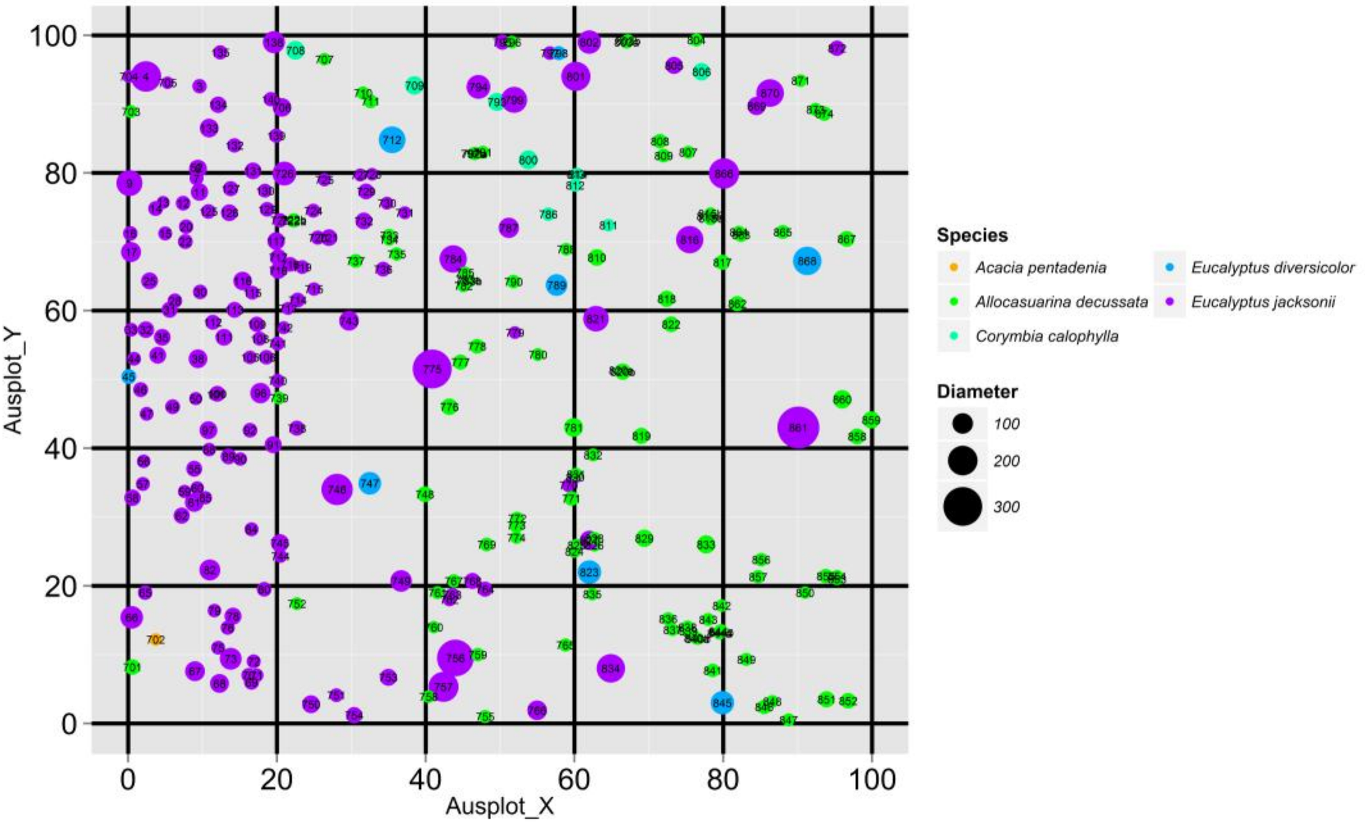

WAFWAR006: Sutton

**AusPlot ID**	WAFWAR006	**Elevation**	142m
**AusPlot Name**	Sutton	**Aspect**	90°
**State**	Western Australia	**Slope**	8°; Gently Inclined
**Bioregion**	Warren	**Landform Element**	Lower Slope
**Location (UTM)**	50 H 431090 6187818	**MAT, MAP**	15.0 ^{o}C, 1006 mm
**Location (Lat/Long)**	-34.4488 116.2498	**Existing Plot Custodian**	WA Department of Parks and Wildlife
**Tenure**	Forest Reserve	**Existing Plot ID**	Permanent Inventory Plot 887
**Plot Est. Date**	13 November 2012	**Existing Plot Area**	0.25ha (50mx50m)
**Plot Size**	1.0ha (100mx100m)	**Existing Plot Census**	1981,1986,1991,1999

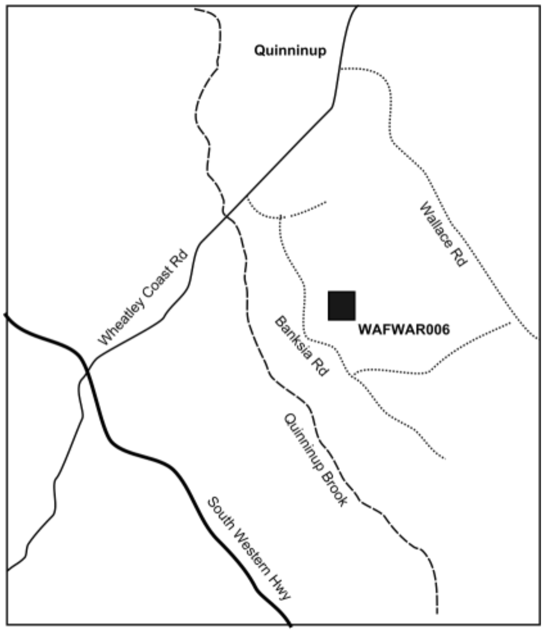

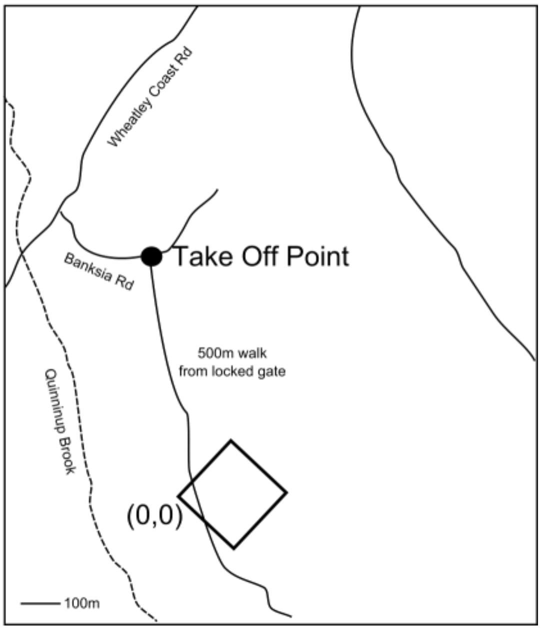

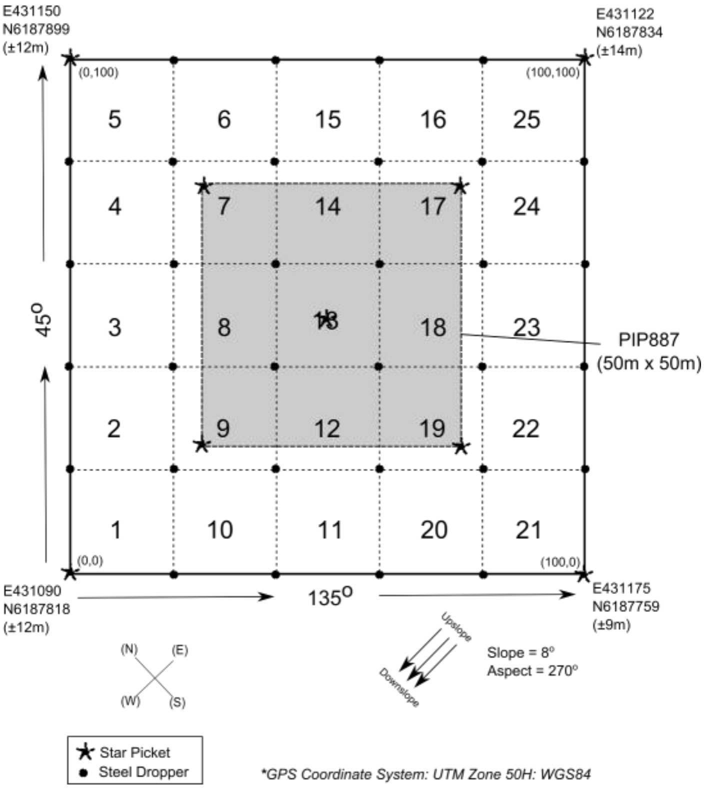

WAFWAR006: Sutton

**Target Eucalypt Species:**	*Eucalyptus diversicolor*	**High severity fire?**	Yes, 1917 (Known Fire Event)
**Maximum Tree Height (m)**	56m	**Low severity fire?**	Yes, frequency unknown (Fire Scars)
**Target Species Growth Stage:**	Mature 1917 regrowth	**Cut stumps?**	No
**Understorey:**	Wet Sclerophyll	**Other Disturbance?**	No

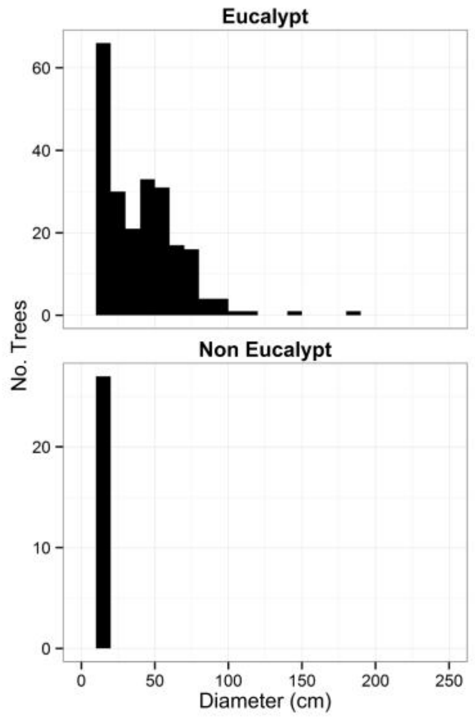

Species	No. Stems	BA (m^2/ha)
Eucalyptus diversicolor	150	39.3
Corymbia calophylla	76	2.4
Acacia melanoxylon	21	0.3
Trymalium odoratissimum	6	0.1

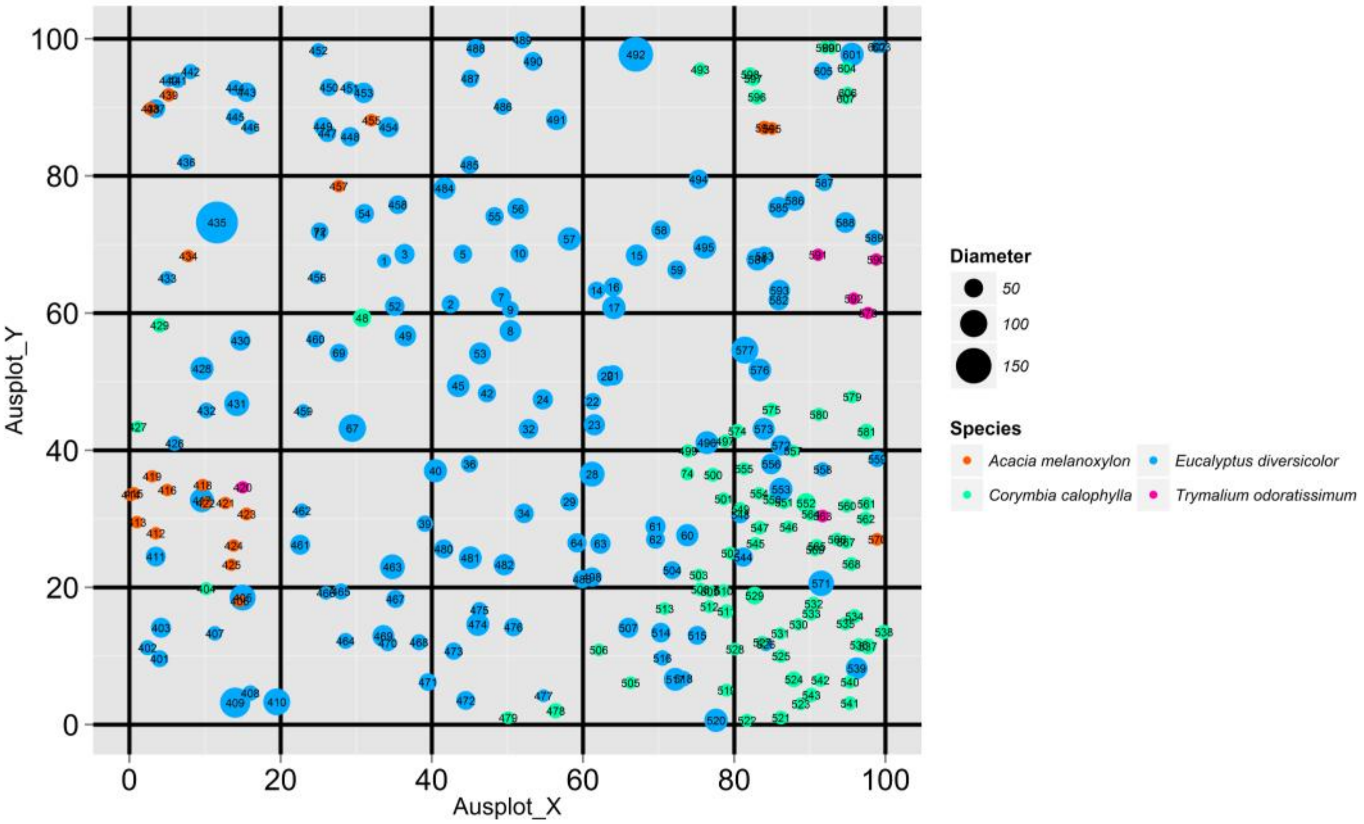

WAFWAR007: Frankland

**AusPlot ID**	WAFWAR007	**Elevation**	239m
**AusPlot Name**	Frankland	**Aspect**	280°
**State**	Western Australia	**Slope**	18°; Steep
**Bioregion**	Warren	**Landform Element**	Upper Slope
**Location (UTM)**	50 H 480432 6146385	**MAT, MAP**	14.8 ^{o}C, 1026 mm
**Location (Lat/Long)**	-34.8247 116.8734	**Existing Plot Custodian**	NA
**Tenure**	Frankland National Park	**Existing Plot ID**	NA
**Plot Est. Date**	18 November 2012	**Existing Plot Area**	NA
**Plot Size**	1.0ha (100mx100m)	**Existing Plot Census**	NA

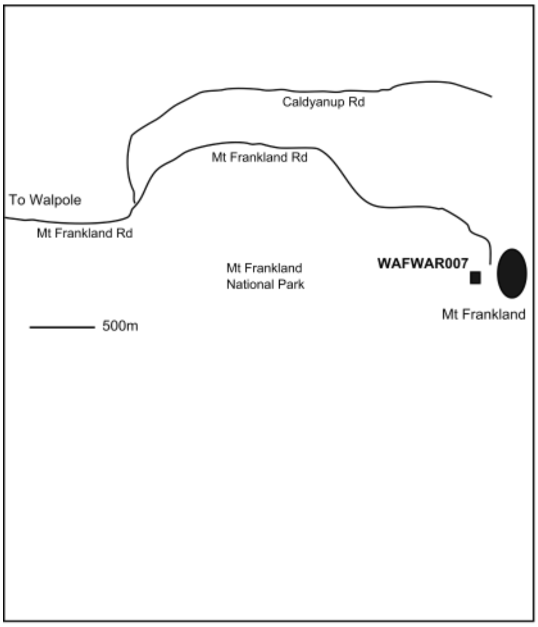

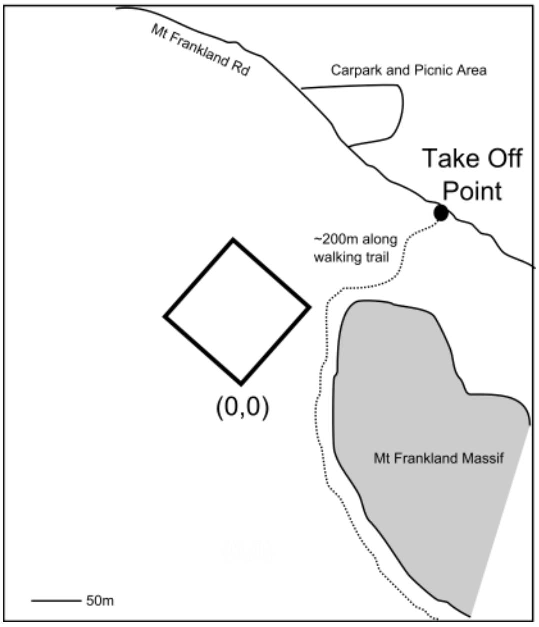

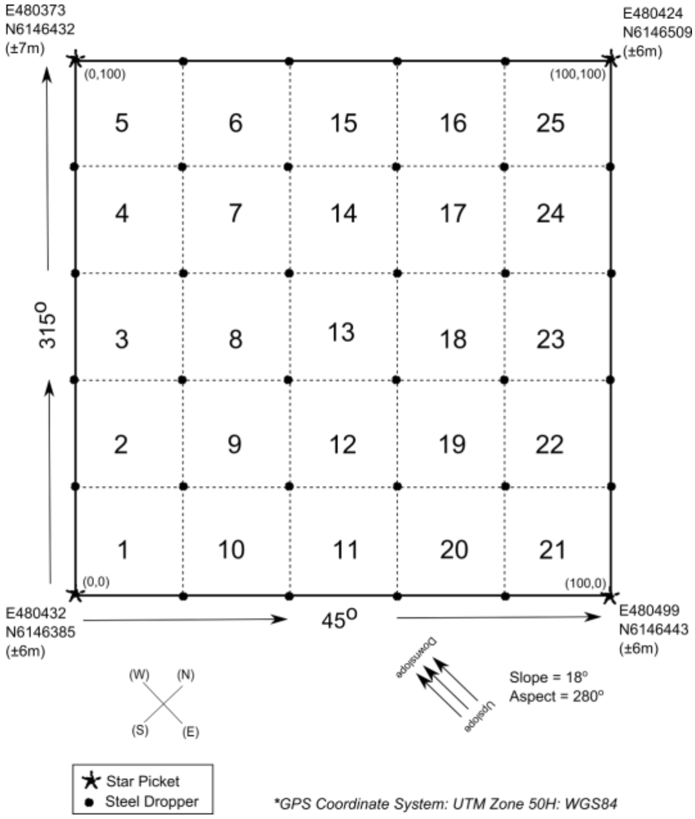

WAFWAR007: Frankland

**Target Eucalypt Species:**	*Eucalyptus diversicolor*	**High severity fire?**	Unknown
**Maximum Tree Height (m)**	65m	**Low severity fire?**	No
**Target Species Growth Stage:**	Mature	**Cut stumps?**	No
**Understorey:**	Wet Sclerophyll	**Other Disturbance?**	No

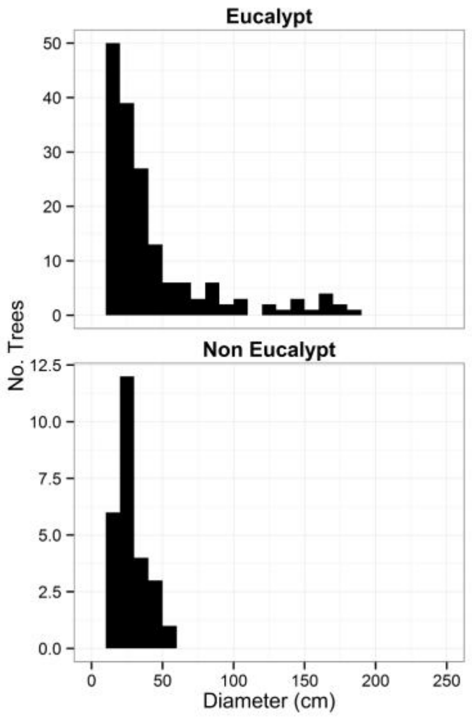

Species	No. Stems	BA (m^2/ha)
Eucalyptus diversicolor	61	35.6
Eucalyptus guilfoylei	108	10.8
Allocasuarina decussata	23	1.7
Trymalium odoratissimum	3	<0.1

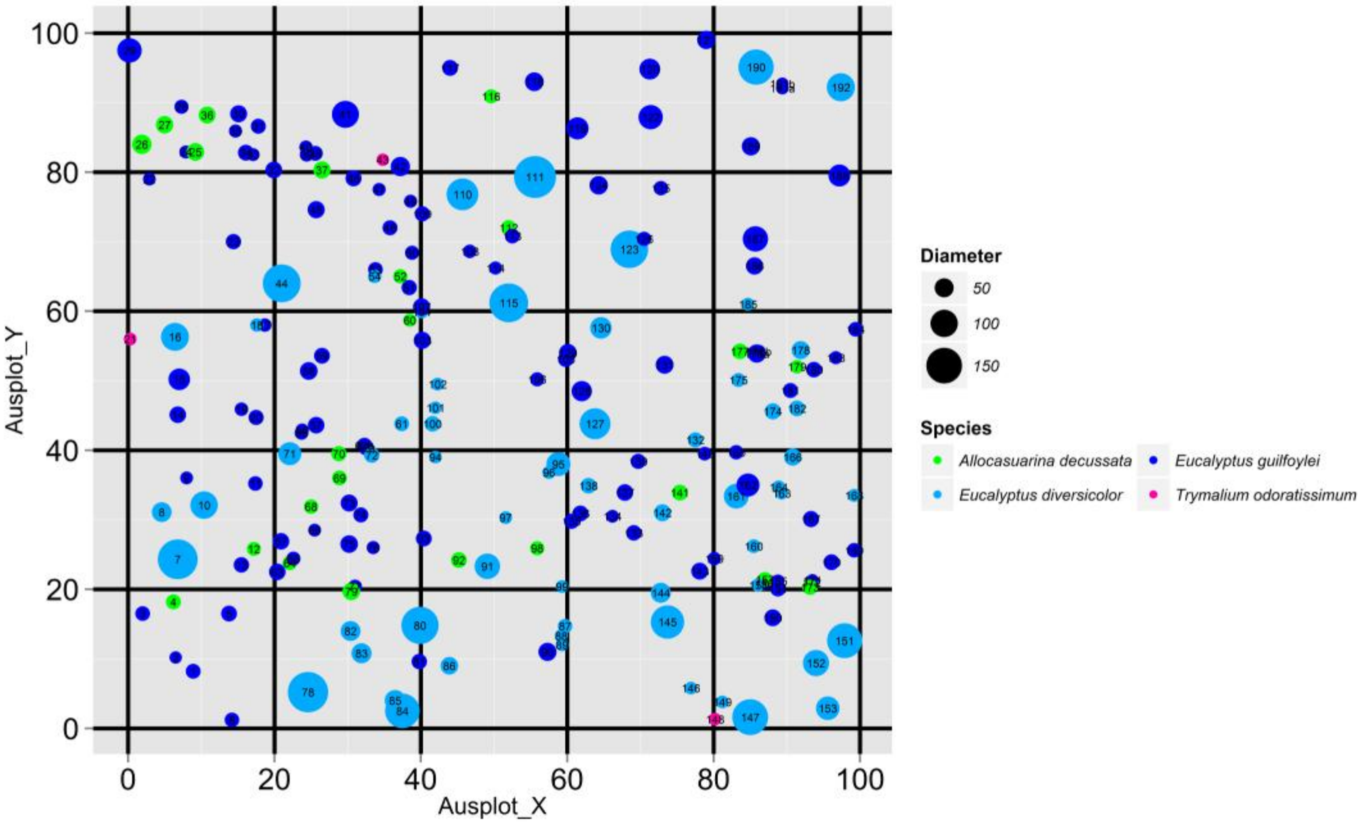

WAFWAR008: Clare

**AusPlot ID**	WAFWAR008	**Elevation**	165m
**AusPlot Name**	Clare	**Aspect**	335°
**State**	Western Australia	**Slope**	8°; Gently Inclined
**Bioregion**	Warren	**Landform Element**	Upper Slope
**Location (UTM)**	50 H 0468098 6127410	**MAT, MAP**	15.0 ^{o}C, 1204 mm
**Location (Lat/Long)**	-34.8247 116.6504	**Existing Plot Custodian**	NA
**Tenure**	Walpole National Park	**Existing Plot ID**	NA
**Plot Est. Date**	22 November 2012	**Existing Plot Area**	NA
**Plot Size**	1.0ha (100mx100m)	**Existing Plot Census**	NA

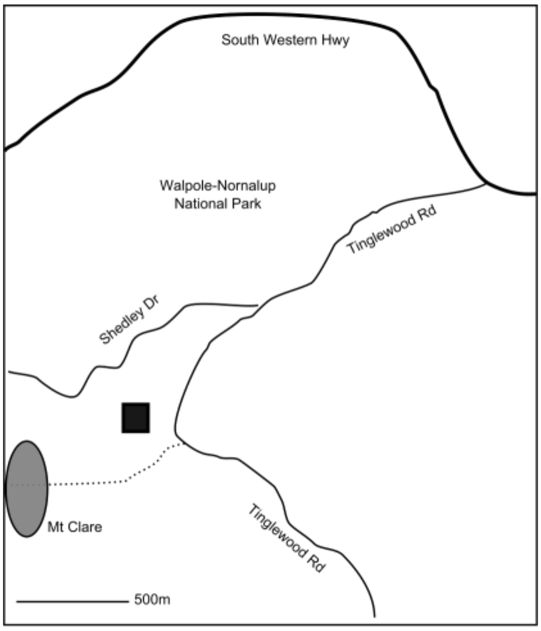

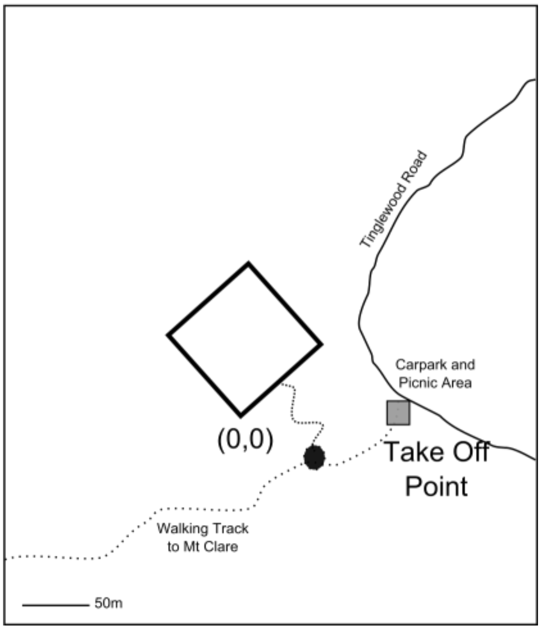

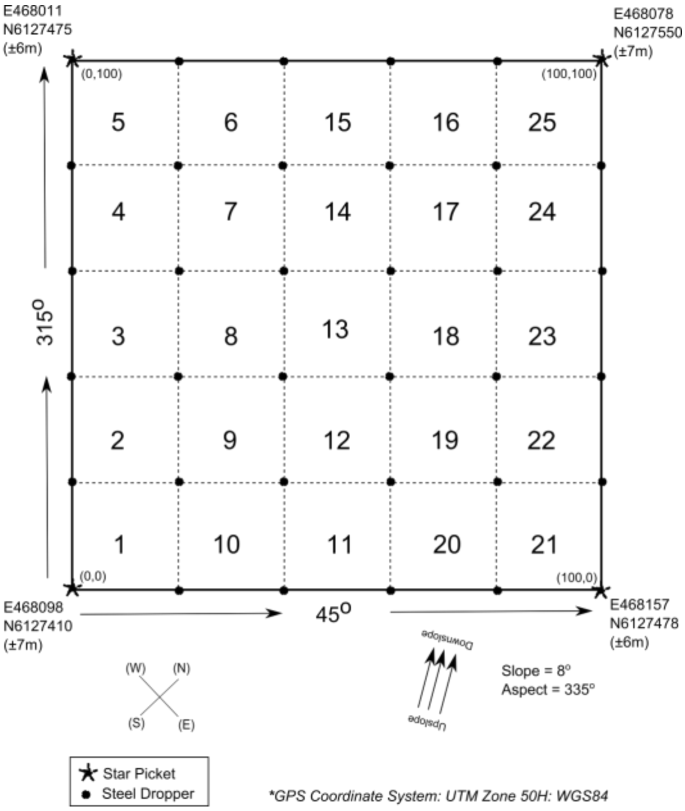

WAFWAR008: Clare

**Target Eucalypt Species:**	*Eucalyptus jacksonii*	**High severity fire?**	Unknown
**Maximum Tree Height (m)**	62m	**Low severity fire?**	Yes, frequency unknown (Fire Scars)
**Target Species Growth Stage:**	Old Growth	**Cut stumps?**	No
**Understorey:**	Wet Sclerophyll	**Other Disturbance?**	No

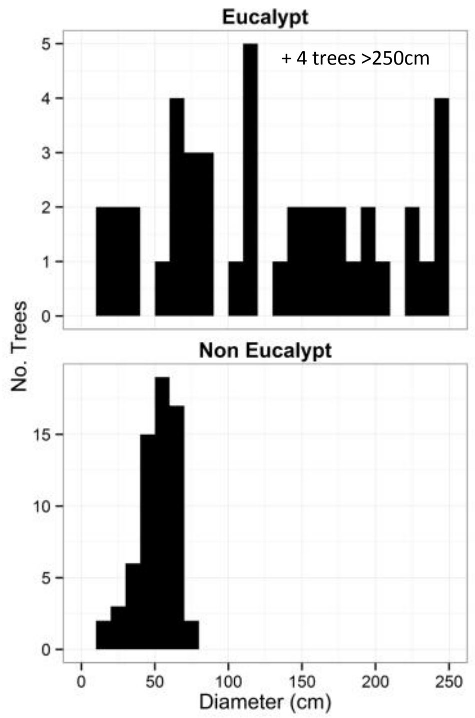

Note: Old Growth Eucalyptus jacksonii *trees at this site form pronounced buttresses and are likely to introduce considerable uncertainty into stand structural calculations based on diameter measurements.*

Species	No. Stems	BA (m^2/ha)
Eucalyptus jacksonii	27	66.8*
Allocasuarina decussata	64	14.3
Eucalyptus diversicolor	8	12.1
Eucalyptus guilfoylei	12	5.6

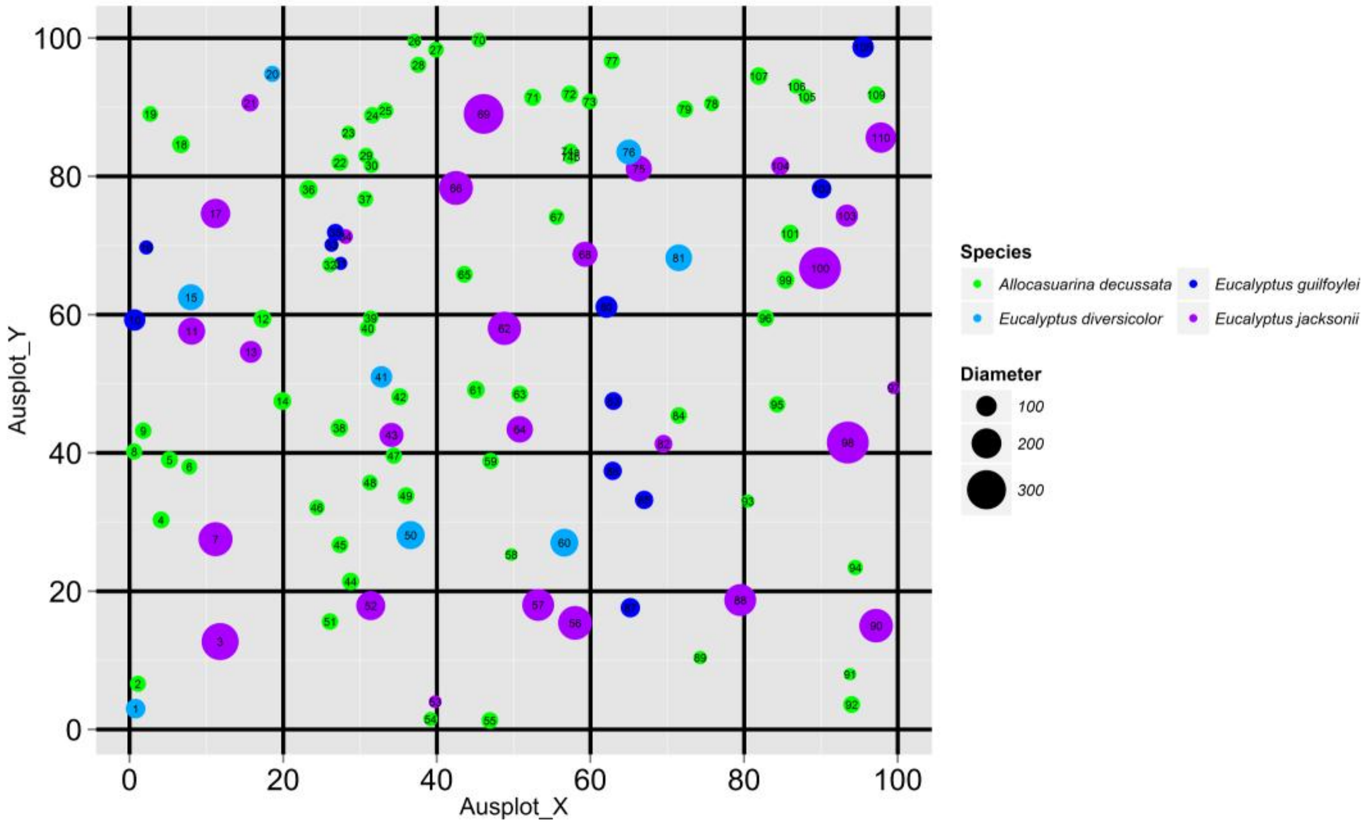

WAFWAR009: Collins

**AusPlot ID**	WAFWAR009	**Elevation**	136m
**AusPlot Name**	Collins	**Aspect**	185°
**State**	Western Australia	**Slope**	16°; Moderate to Steep
**Bioregion**	Warren	**Landform Element**	Lower Slope
**Location (UTM)**	50 H 419607 6181189	**MAT, MAP**	14.9 ^{o}C, 1120 mm
**Location (Lat/Long)**	–34.5078 116.1242	**Existing Plot Custodian**	WA Department of Parks and Wildlife
**Tenure**	Forest Reserve	**Existing Plot ID**	Permanent Inventory Plot 605
**Plot Est. Date**	26 November 2012	**Existing Plot Area**	0.8ha (400x20)
**Plot Size**	1.0ha (100mx100m)	**Existing Plot Census**	1964, 1971,1982,

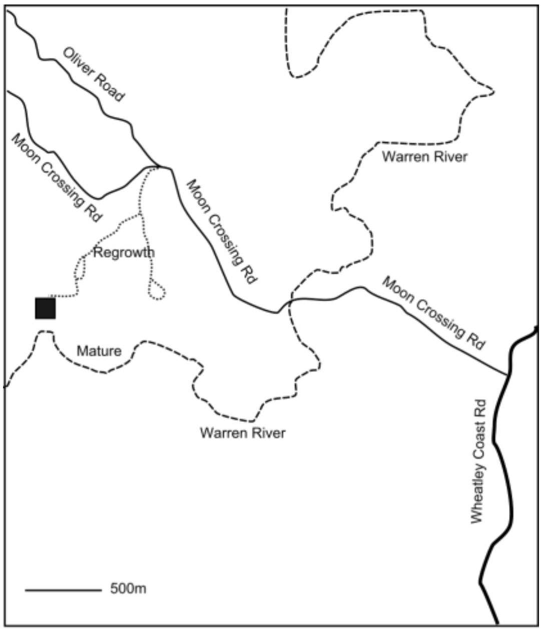

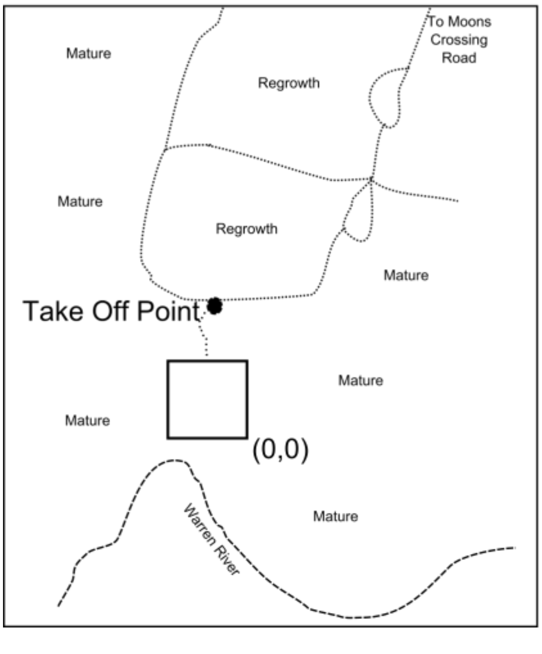

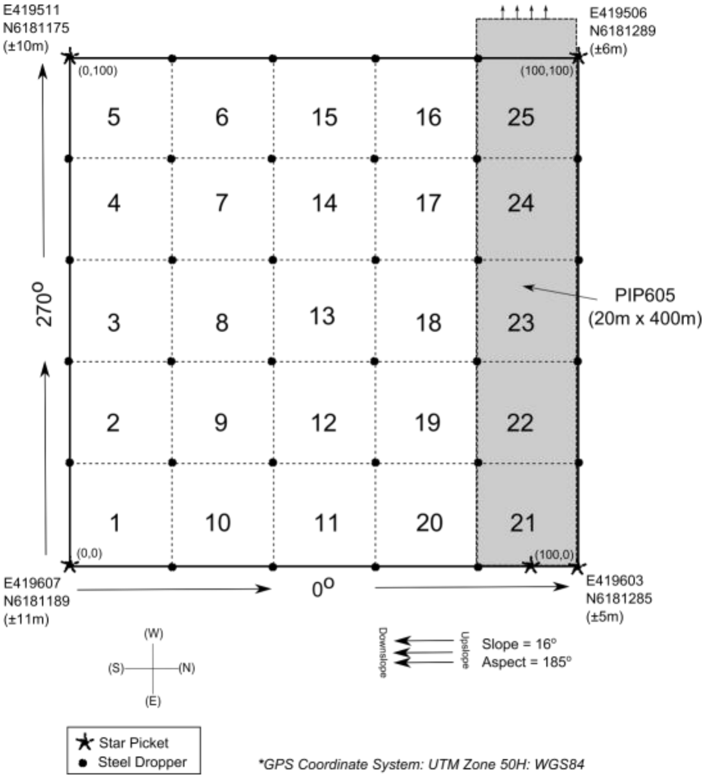

WAFWAR009: Collins

**Target Eucalypt Species:**	*Eucalyptus diversicolor*	**High severity fire?**	Unknown
**Maximum Tree Height (m)**	76m	**Low severity fire?**	Yes,
**Target Species Growth Stage:**	Mature	**Cut stumps?**	Yes, four stumps, “cutover in 1958”
**Understorey:**	Wet Sclerophyll	**Other Disturbance?**	No

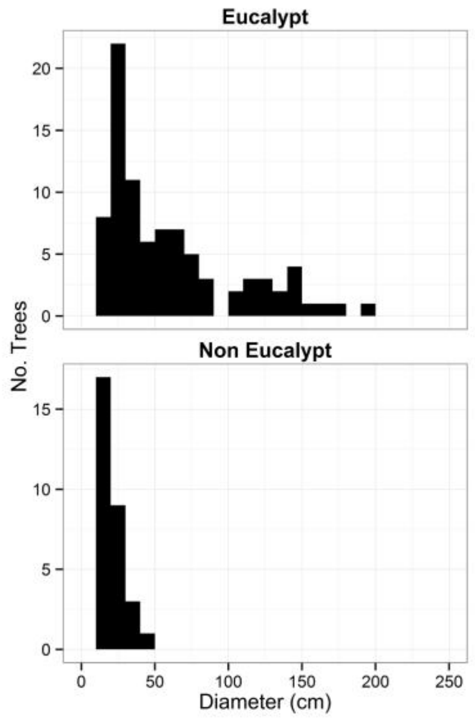

Species	No. Stems	BA (m^2/ha)
Eucalyptus diversicolor	87	38.6
Allocasuarina decussata	20	1.1
Trymalium odoratissimum	10	0.1

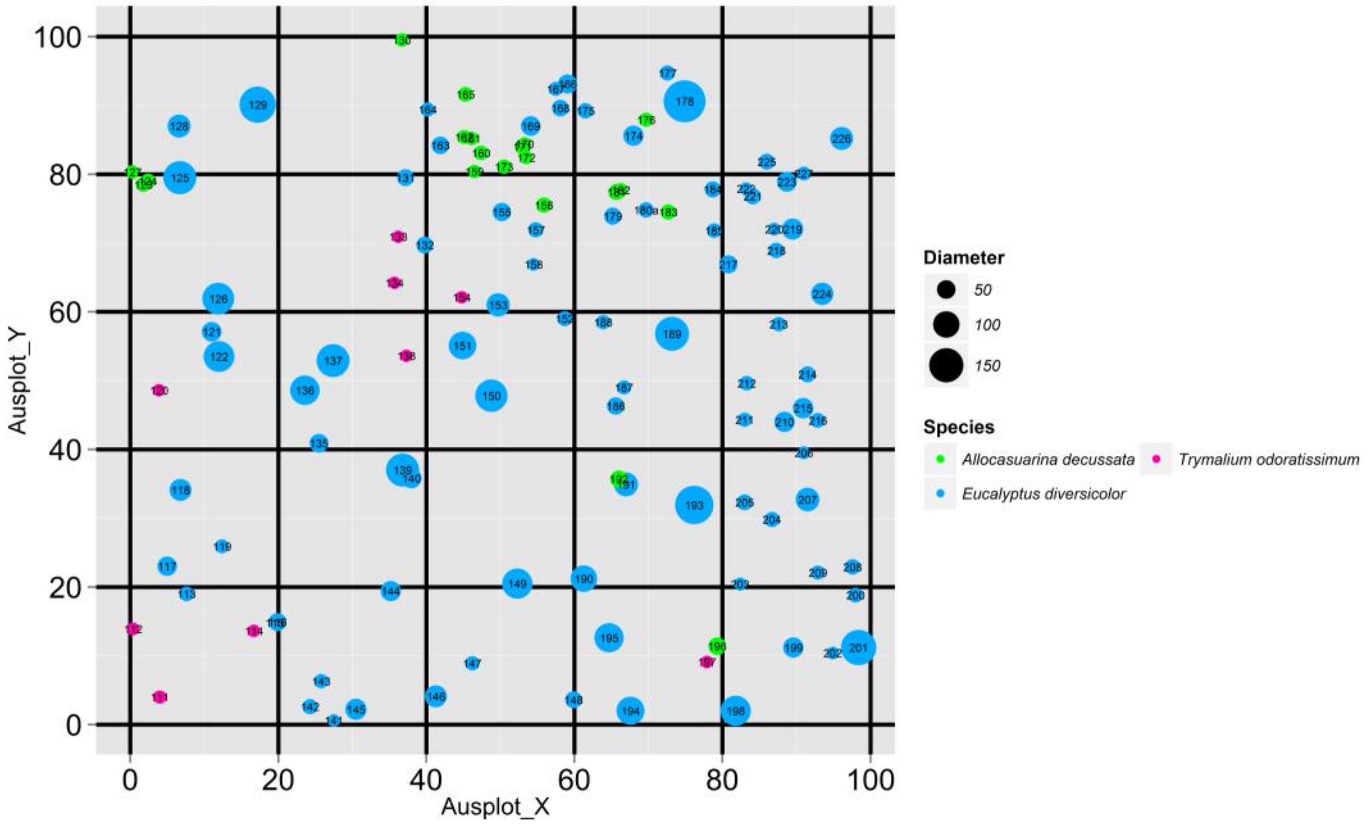

Victoria (VCF)

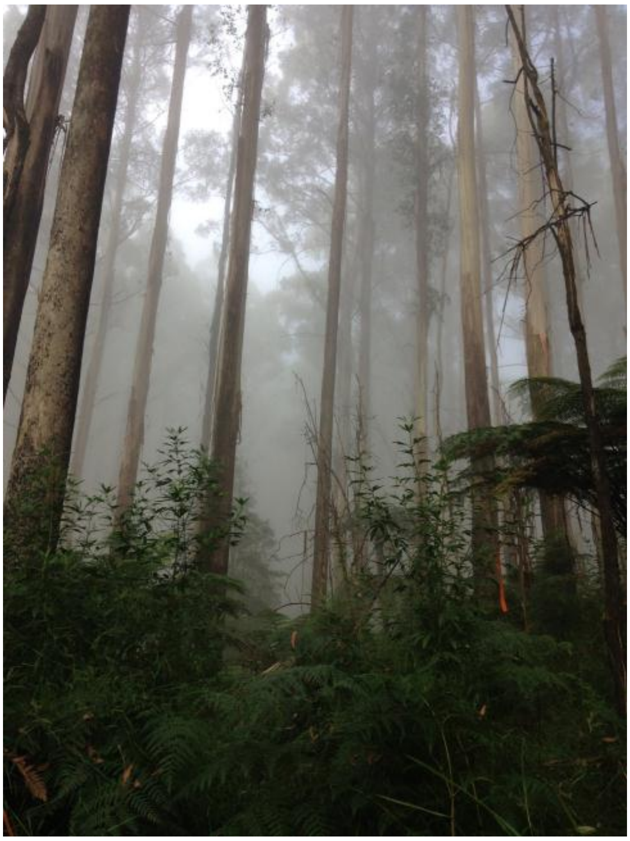

1939 *Eucalyptus regnans* at VCFSEH006 (Healesville)

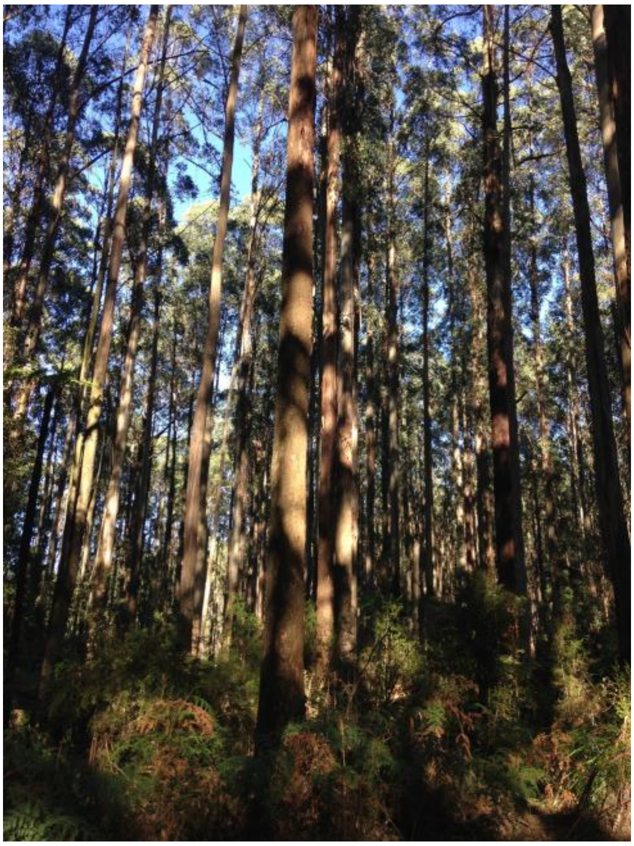

1939 *Eucalyptus regnans* at VCFSEH007 (Healesville)

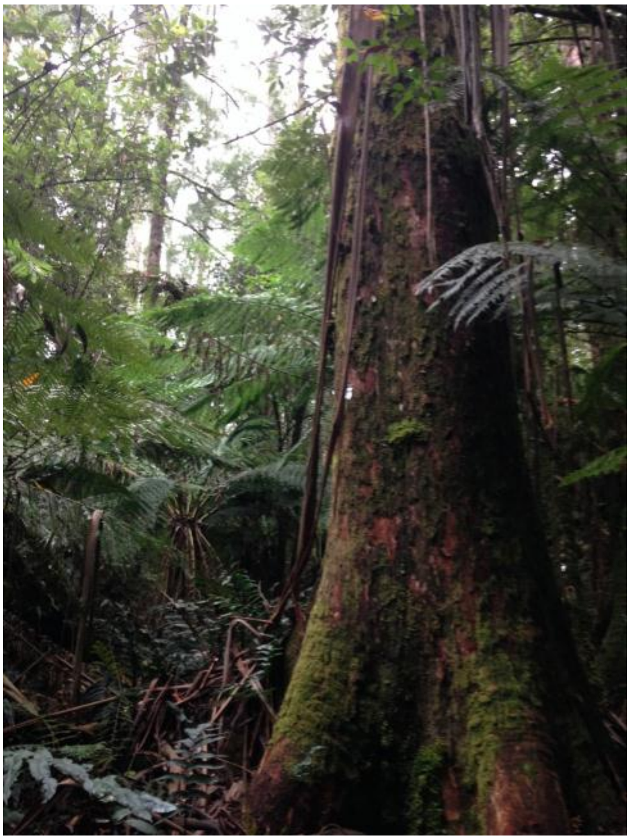

Eucalyptus regnans at VCFSEH005 (Otways)

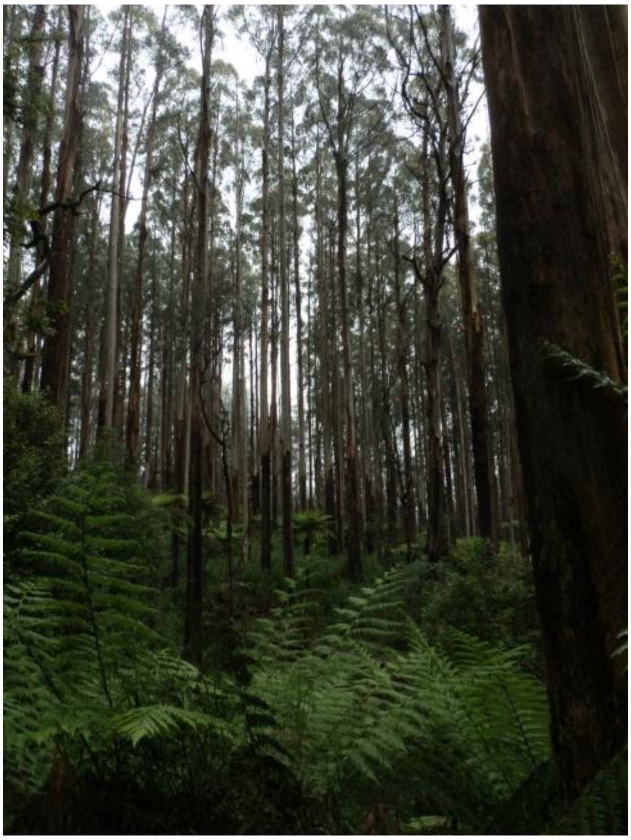

1939 Eucalyptus regnans at VCFSEH001 (Toolangi)

Victoria (VCF)

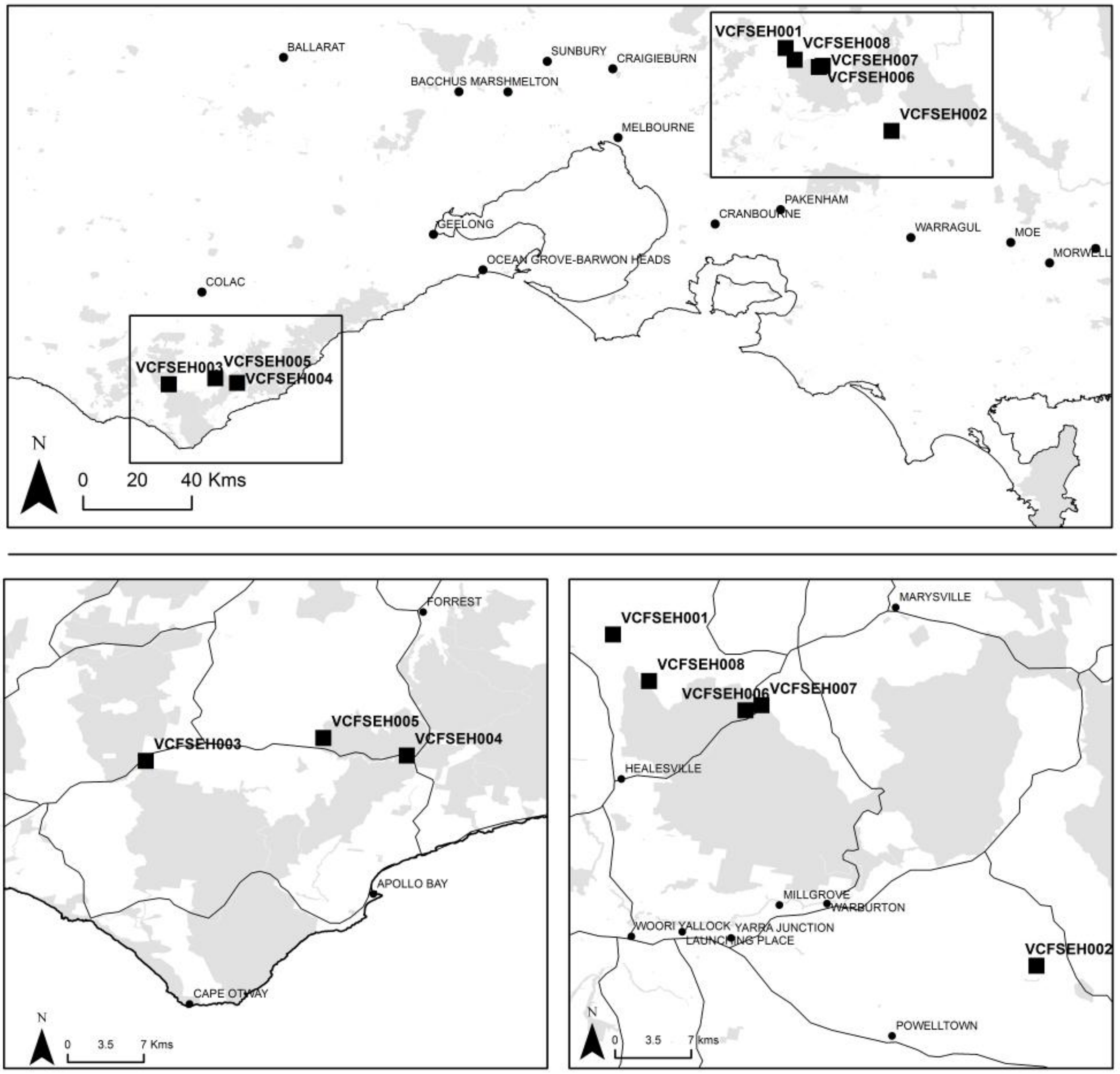

VCFSEH001: ANU101

**AusPlot ID**	VCFSEH001	**Elevation**	337m
**AusPlot Name**	ANU101	**Aspect**	290°
**State**	Victoria	**Slope**	Not recorded; Moderate
**Bioregion**	South East Highlands	**Landform Element**	Midslope
**Location (UTM)**	55 H 368942 5845203	**MAT, MAP**	11.2 ^{o}C, 1502 mm
**Location (Lat/Long)**	-37.5308 145.5167	**Existing Plot Custodian**	Australian National University (Lindenmayer)
**Tenure**	ANU Research Reserve	**Existing Plot ID**	Ecological Research Plot 101
**Plot Est. Date**	01 February 2014	**Existing Plot Area**	2.0 ha
**Plot Size**	1.0ha (100mx100m)	**Existing Plot Census**	Early 1980's, ongoing.

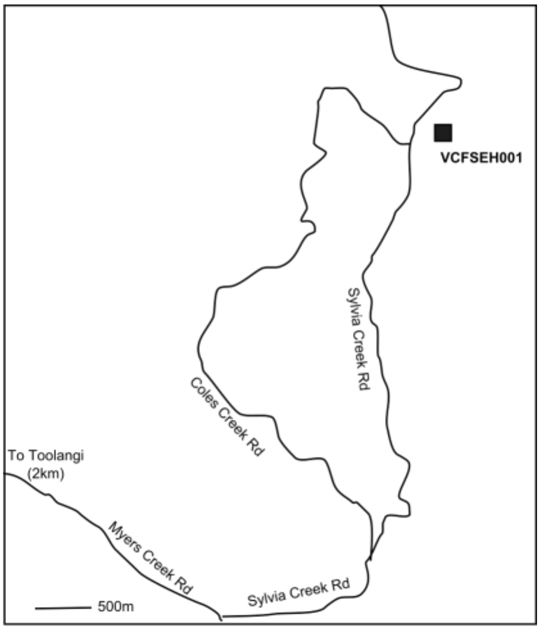

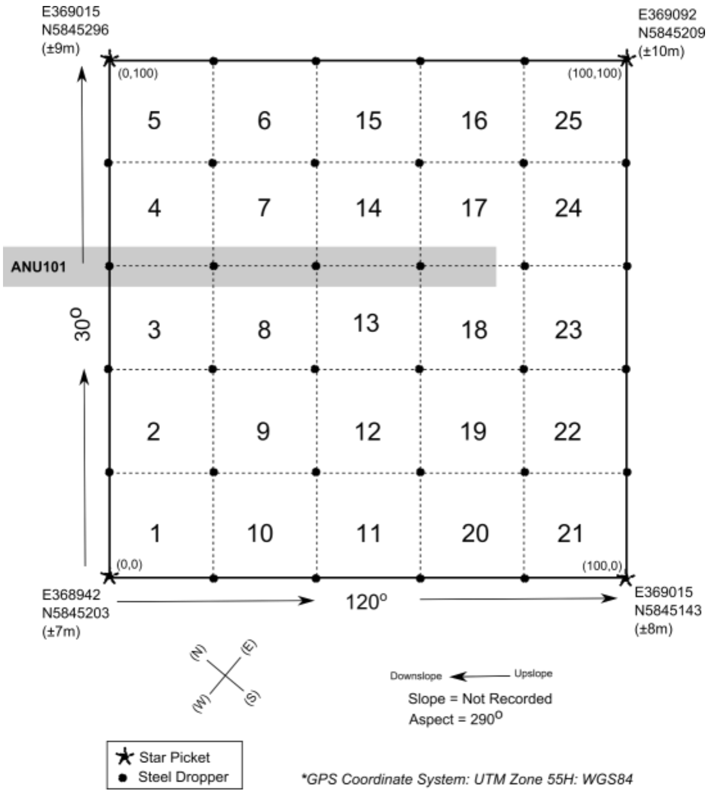

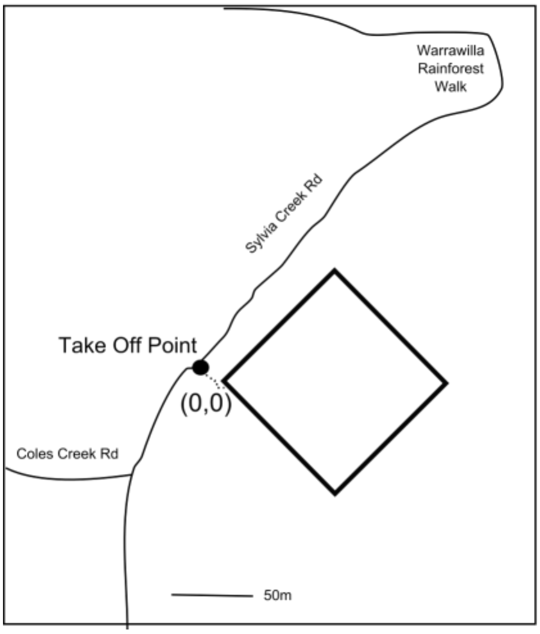

VCFSEH001: ANU101

Target Eucalypt Species: *Eucalyptus regnans*

Maximum Tree Height (m) 72m

Target Species Growth Stage: Mature, 1939 regrowth

Understorey: Wet Sclerophyll

High severity fire? Yes, 1939 (known fire event, ANU)

Low severity fire? No

Cut stumps? One stump, axe marks.

Other Disturbance? No

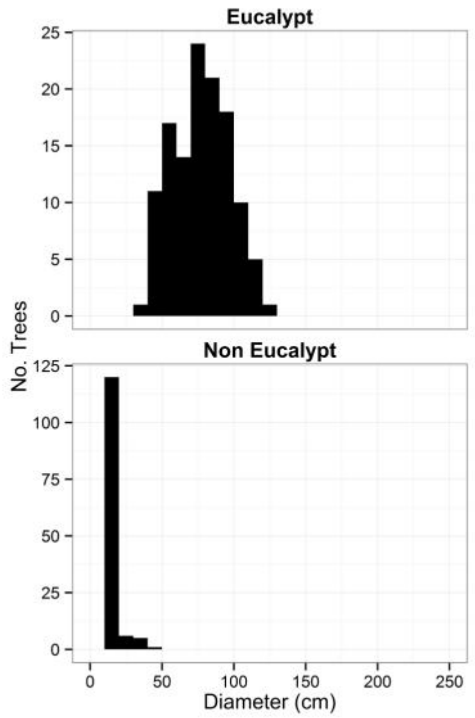

Species	No. Stems	BA (m^2/ha)
Eucalyptus regnans	122	59.5
Pomaderris aspera	103	1.1
Acacia dealbata	8	0.6
Acacia melanoxylon	5	0.2
Hedycarya angustifolia	12	0.2
Zieria arborescens	3	<0.1
UVCFUS2	1	<0.1

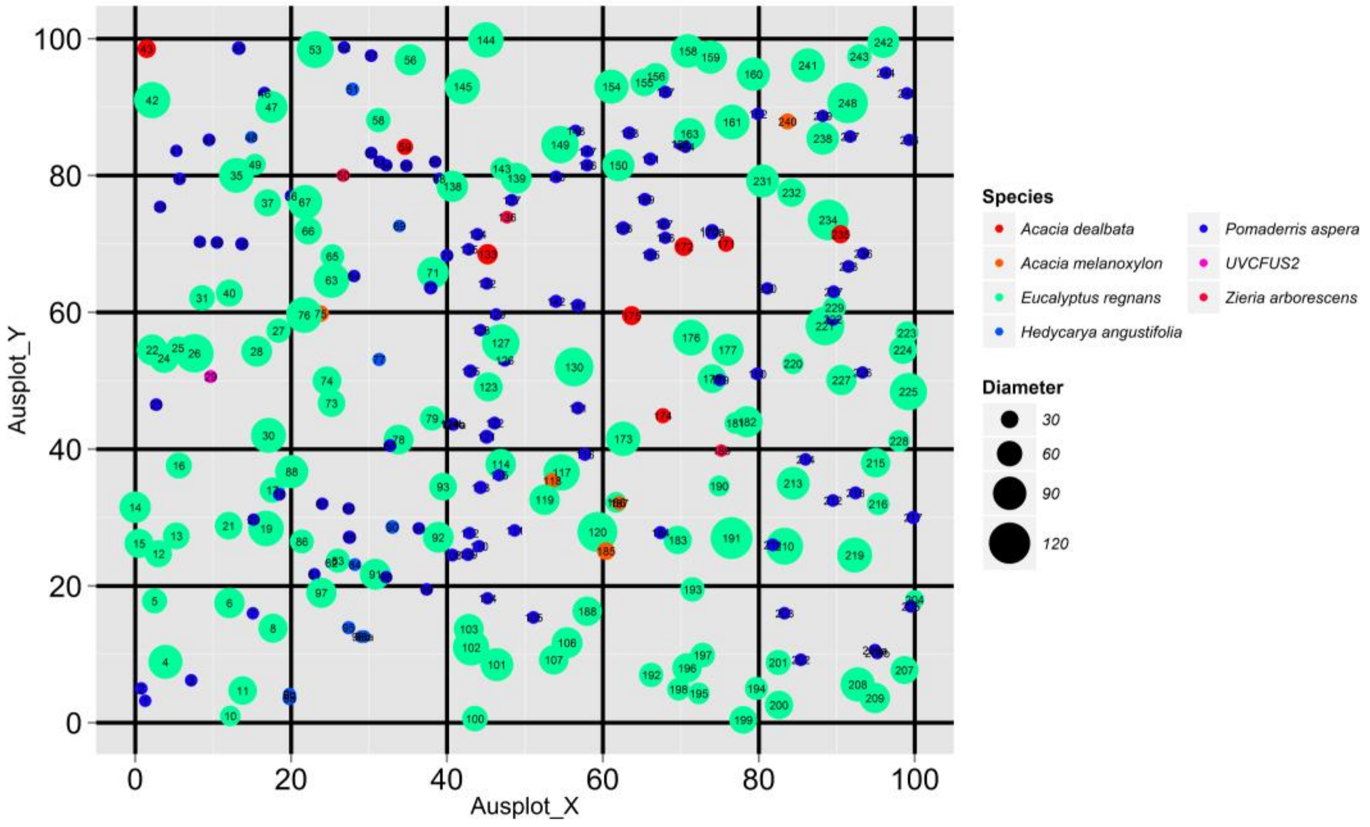

VCFSEH002: ADA TREE/ANU708

**AusPlot ID**	VCFSEH002	**Elevation**	784m
**AusPlot Name**	Ada Tree/ANU708	**Aspect**	225°
**State**	Victoria	**Slope**	Not recorded; Gently Inclined
**Bioregion**	South East Highlands	**Landform Element**	Midslope
**Location (UTM)**	55 H 400285 5815219	**MAT, MAP**	10.3 ^{o}C, 1714 mm
**Location (Lat/Long)**	-37.8049 145.8672	**Existing Plot Custodian**	Australian National University (Lindenmayer)
**Tenure**	ANU Research Reserve	**Existing Plot ID**	Ecological Research Plot 708
**Plot Est. Date**	5 February 2014	**Existing Plot Area**	2.0 ha
**Plot Size**	1.0ha (100mx100m)	**Existing Plot Census**	Early 1980's, ongoing.

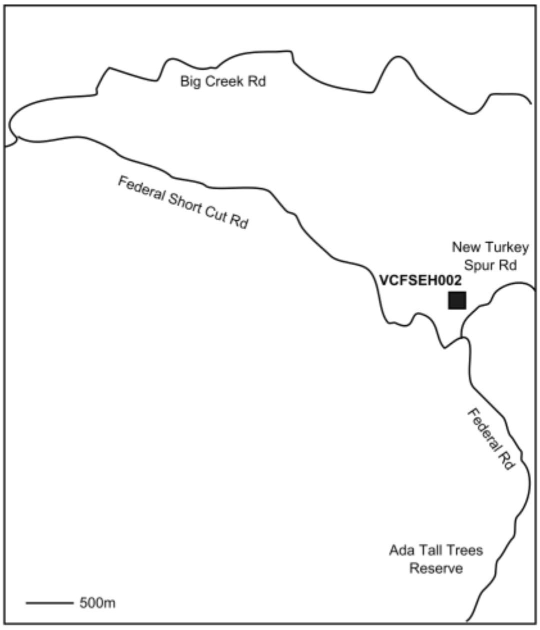

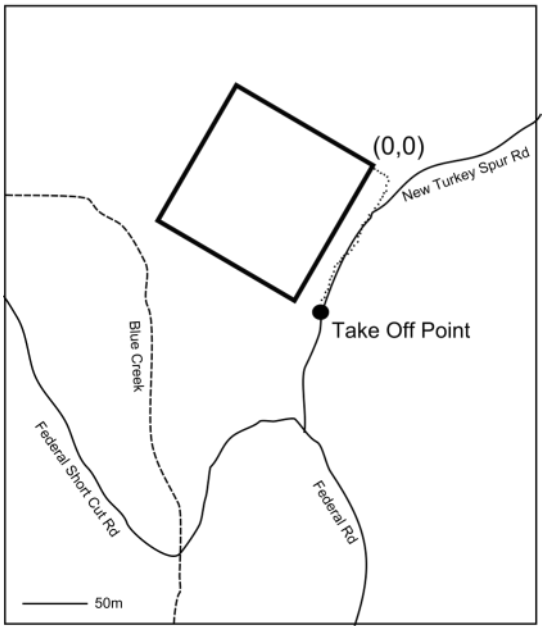

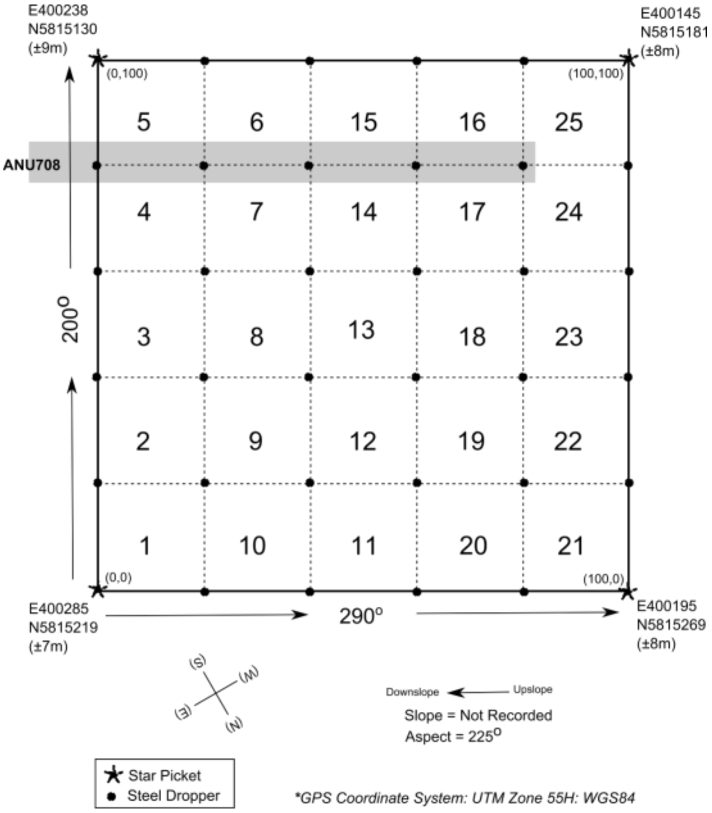

VCFSEH002: ADA TREE/ANU708

**Target Eucalypt Species:**	*Eucalyptus regnans*	**High severity fire?**	Yes, 1939 (known fire event, ANU)
**Maximum Tree Height (m)**	82m	**Low severity fire?**	No
**Target Species Growth Stage:**	Mature, 1939 regrowth	**Cut stumps?**	One cut stump, last 5-10 years
**Understorey:**	Wet Sclerophyll	**Other Disturbance?**	No

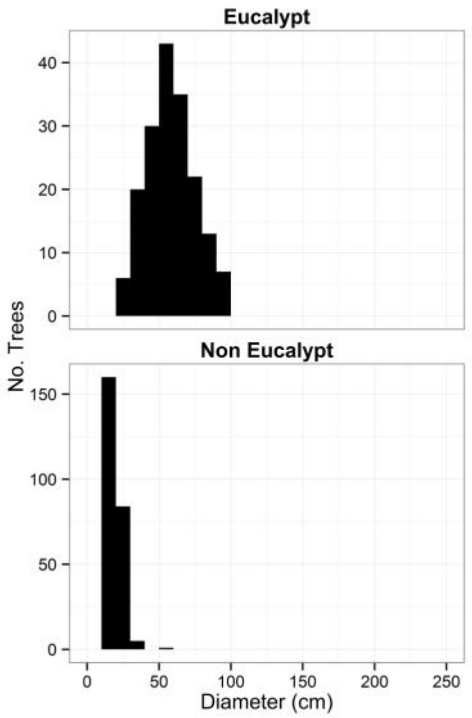

Species	No. Stems	BA (m^2/ha)
Eucalyptus regnans	177	49.1
Acacia melanoxylon	226	7.1
Pomaderris aspera	7	0.1
Persoonia arborea	4	0.1
Acacia dealbata	2	0.1
UVCFUS3	3	0.1
Olearia argophylla	3	<0.1
Leptospermum scoparium	3	<0.1
Hedycarya angustifolia	1	<0.1
Tasmannia lanceolata	1	<0.1

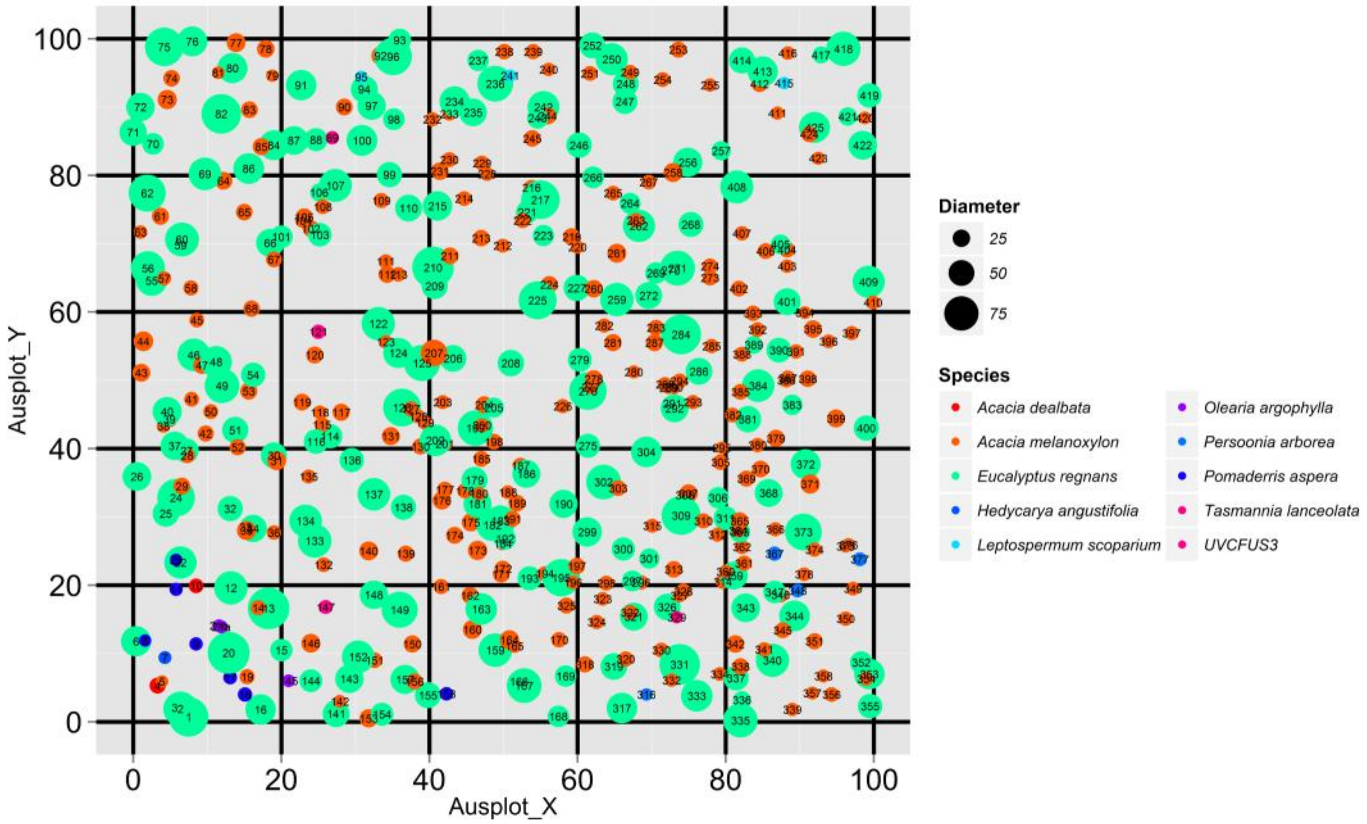

VCFSEH003: Weeaproinah

**AusPlot ID**	VCFSEH003	**Elevation**	471m
**AusPlot Name**	Weeaproinah	**Aspect**	290°
**State**	Victoria	**Slope**	4°; Gently Inclined
**Bioregion**	South East Highlands	**Landform Element**	Midslope
**Location (UTM)**	54 H 715400 5719853	**MAT, MAP**	11.2 ^{o}C, 1869 mm
**Location (Lat/Long)**	-38.6437 143.4749	**Existing Plot Custodian**	NA
**Tenure**	Otway National Park	**Existing Plot ID**	NA
**Plot Est. Date**	18 February 2014	**Existing Plot Area**	NA
**Plot Size**	1.0ha (100mx100m)	**Existing Plot Census**	NA

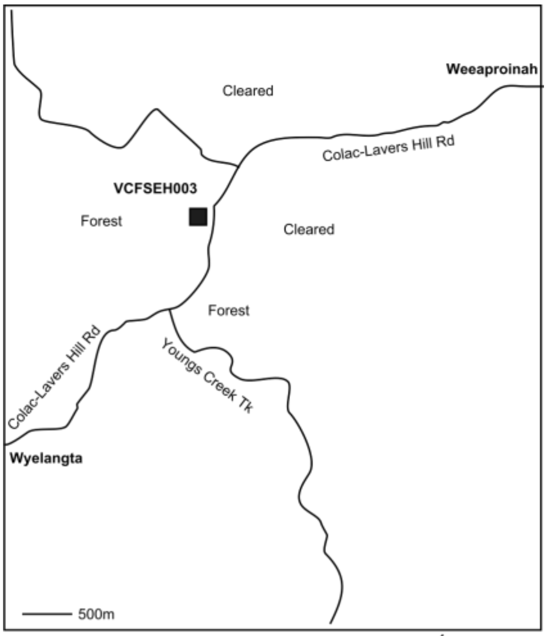

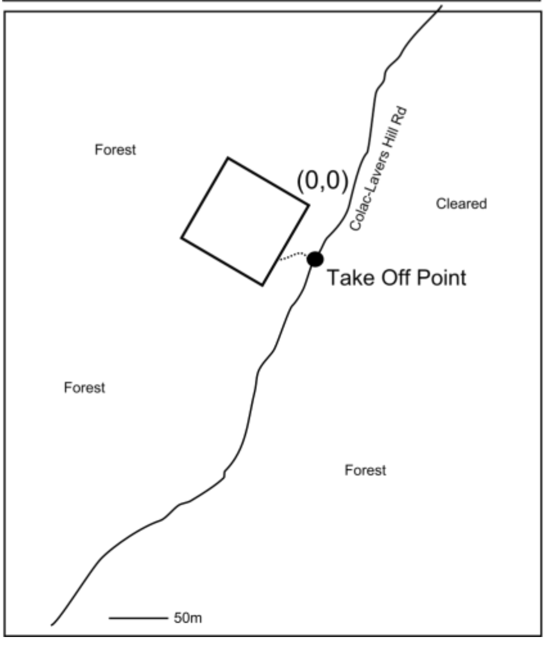

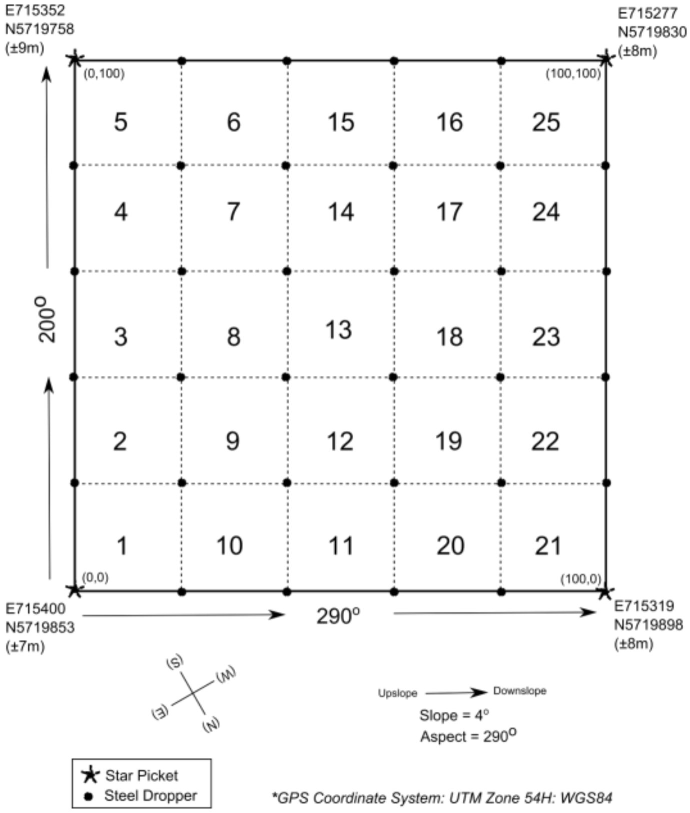

VCFSEH003: Weeaproinah

Target Eucalypt Species: *Eucalyptus regnans*

High severity fire? Yes, 1939 (known fire event)

Maximum Tree Height (m) 77m

Low severity fire? No

Target Species Growth Stage: Mature, 1939 regrowth

Cut stumps? No

Understorey: Wet schlerophyll

Other Disturbance? No

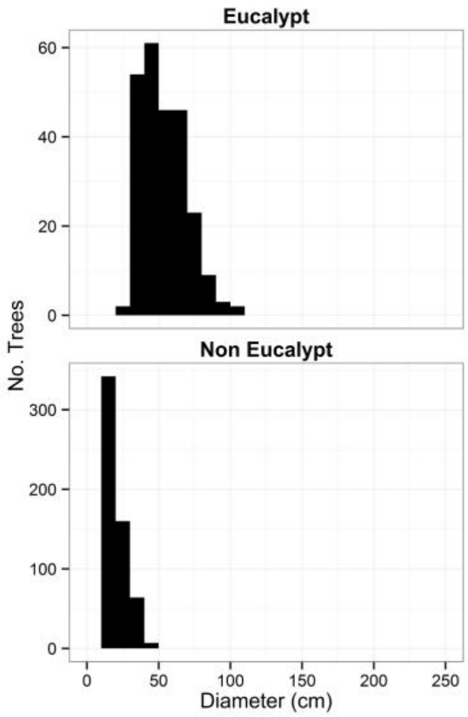

Species	No. Stems	BA (m^2/ha)
Eucalyptus regnans	243	55.8
Acacia melanoxylon	180	11.1
Nematolepis squamea	372	8.9
Eucalyptus obliqua	3	0.3
Olearia argophylla	15	0.2
Hedycarya angustifolia	3	<0.1
Pomaderris aspera	1	<0.1
Tasmannia lanceolata	1	<0.1
UVCFUS1	1	<0.1

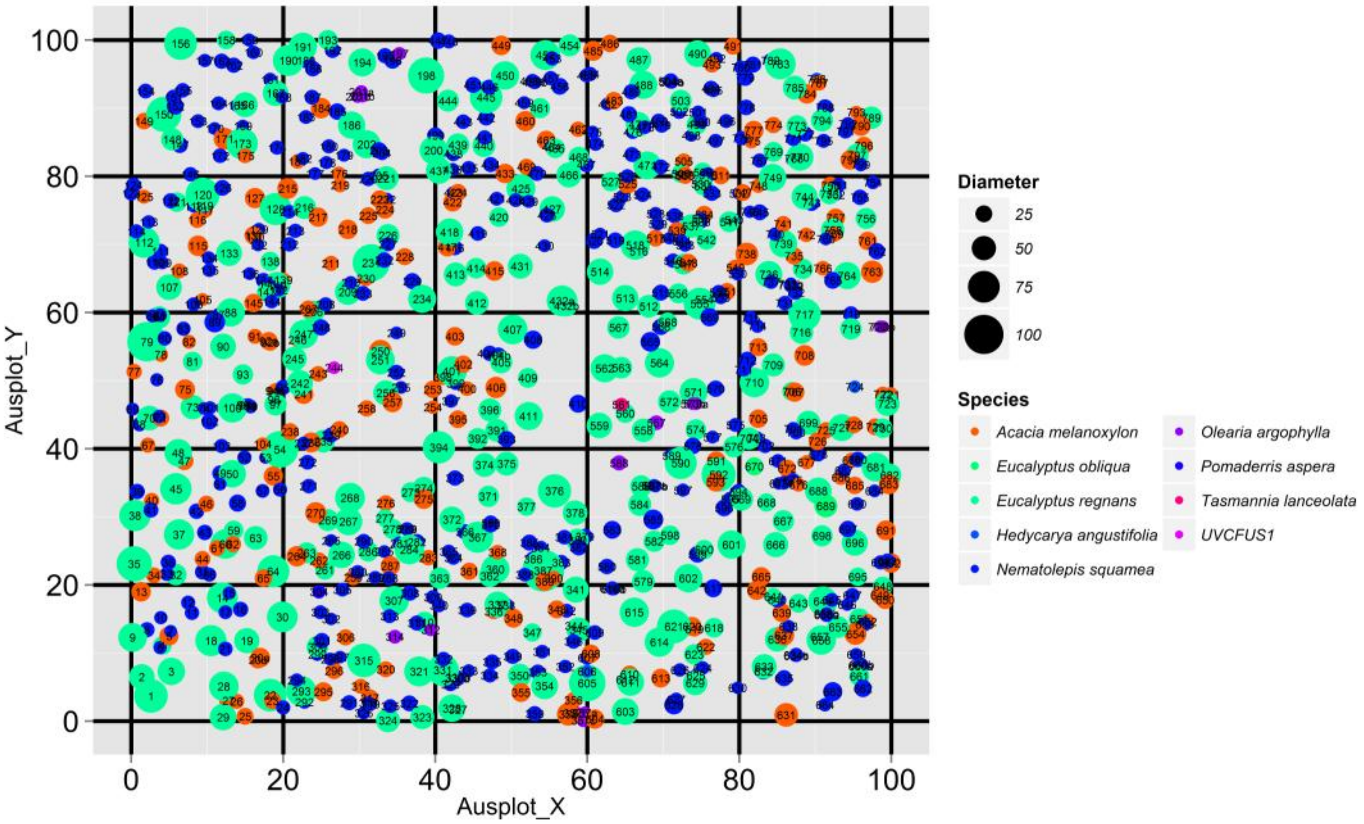

VCFSEH004: Turtons

**AusPlot ID**	VCFSEH004	**Elevation**	480m
**AusPlot Name**	Turtons	**Aspect**	245°
**State**	Victoria	**Slope**	Not recorded; Gently Inclined,Moderate
**Bioregion**	South East Highlands	**Landform Element**	Ridge,Midslope
**Location (UTM)**	54 H 735203 5719727	**MAT, MAP**	11.1 ^{o}C, 1654 mm
**Location (Lat/Long)**	-38.6390 143.7014	**Existing Plot Custodian**	NA
**Tenure**	Otway National Park	**Existing Plot ID**	NA
**Plot Est. Date**	20 February 2014	**Existing Plot Area**	NA
**Plot Size**	1.0ha (100mx100m)	**Existing Plot Census**	NA

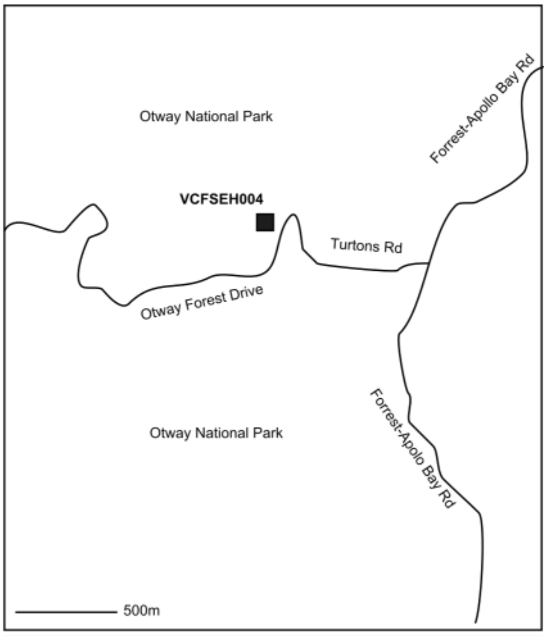

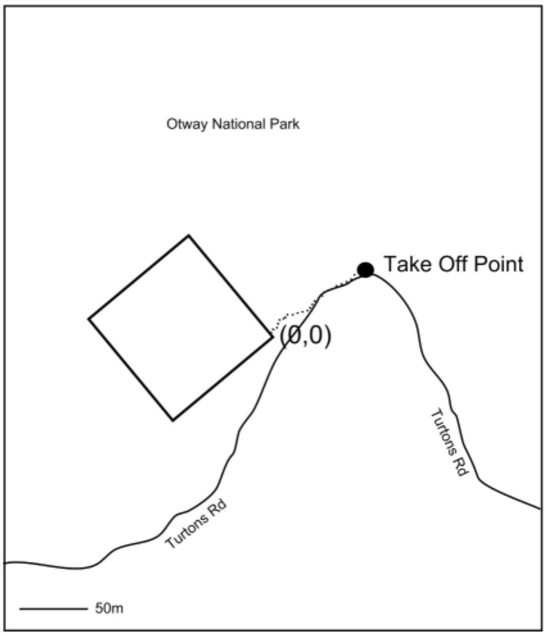

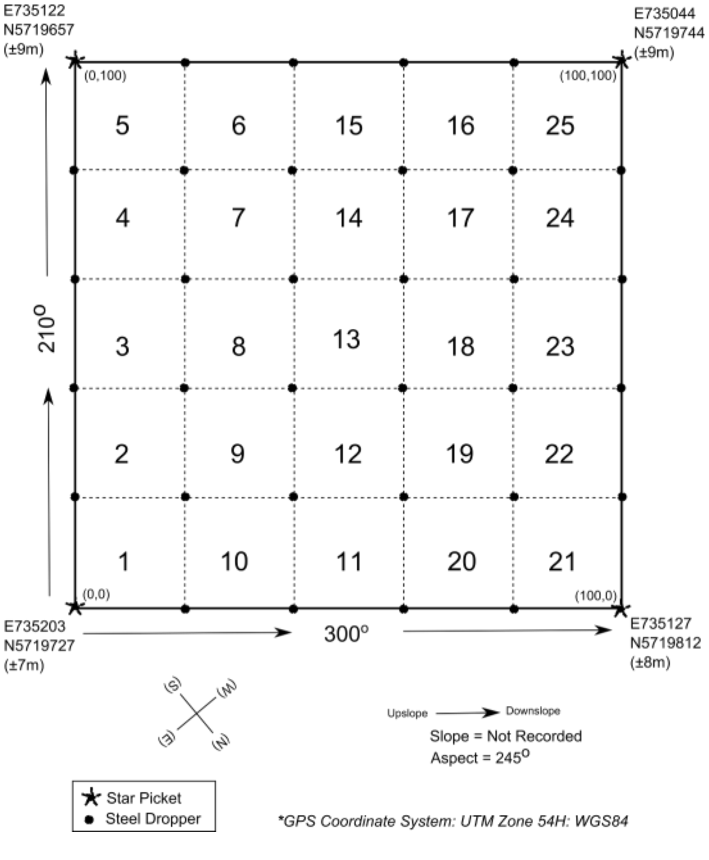

VCFSEH004: Turtons

**Target Eucalypt Species:**	*Eucalyptus regnans*	**High severity fire?**	Yes, 1898 (known fire event, ANU)
**Maximum Tree Height (m)**	88m	**Low severity fire?**	No
**Target Species Growth Stage:**	Mature, 1898 regrowth	**Cut stumps?**	No
**Understorey:**	Wet Sclerophyll	**Other Disturbance?**	No

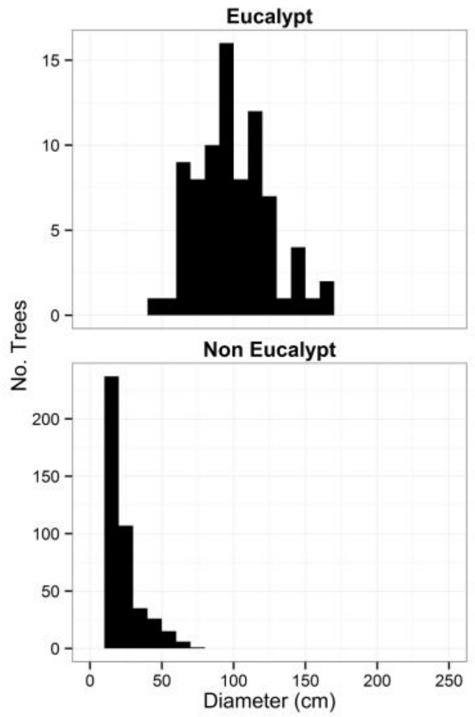

Species	No. Stems	BA (m^2/ha)
Eucalyptus regnans	80	56.7
Acacia melanoxylon	84	12.5
Nematolepis squamea	109	5.0
Olearia argophylla	95	2.5
Hedycarya angustifolia	127	2.2
Pittosporum bicolor	3	0.1
Lomatia fraseri	3	<0.1
Coprosma quadrifida	3	<0.1
Prostanthera lasianthos	2	<0.1
Bedfordia arborescens	1	<0.1

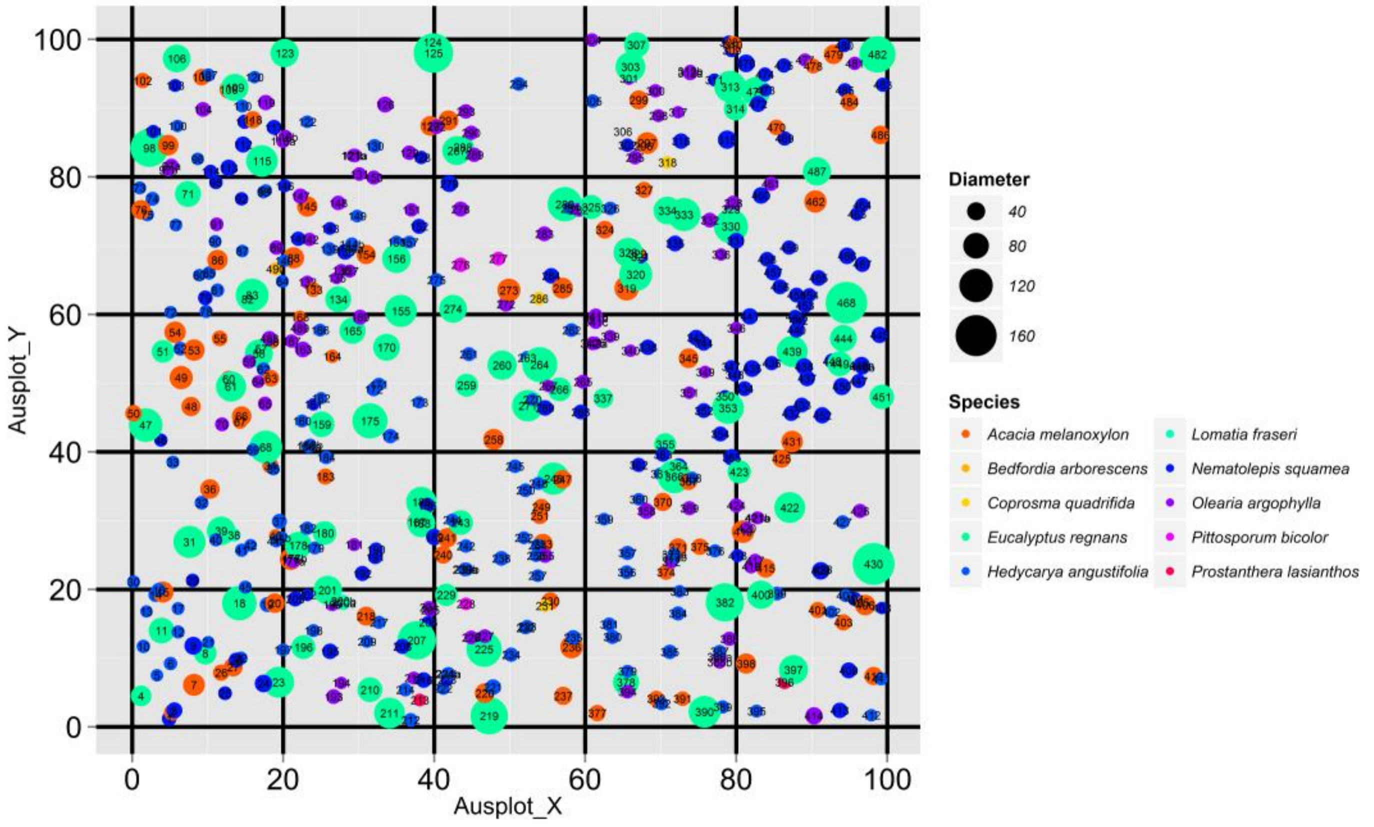

VCFSEH005: Lardner

**AusPlot ID**	VCFSEH005	**Elevation**	531m
**AusPlot Name**	Lardner	**Aspect**	75°
**State**	Victoria	**Slope**	Not recorded; Gently Inclined
**Bioregion**	South East Highlands	**Landform Element**	Upper Slope
**Location (UTM)**	54 H 728572 5721093	**MAT, MAP**	11.3 ^{o}C, 1701 mm
**Location (Lat/Long)**	-38.6238 143.6290	**Existing Plot Custodian**	NA
**Tenure**	Otway National Park	**Existing Plot ID**	NA
**Plot Est. Date**	20 February 2014	**Existing Plot Area**	NA
**Plot Size**	1.0ha (100mx100m)	**Existing Plot Census**	NA

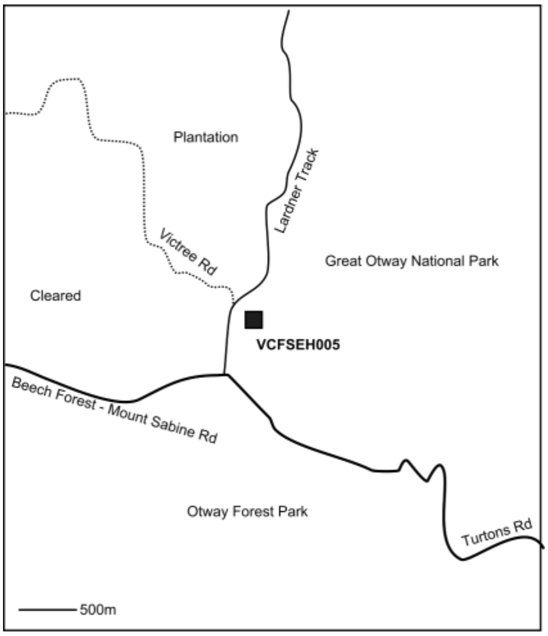

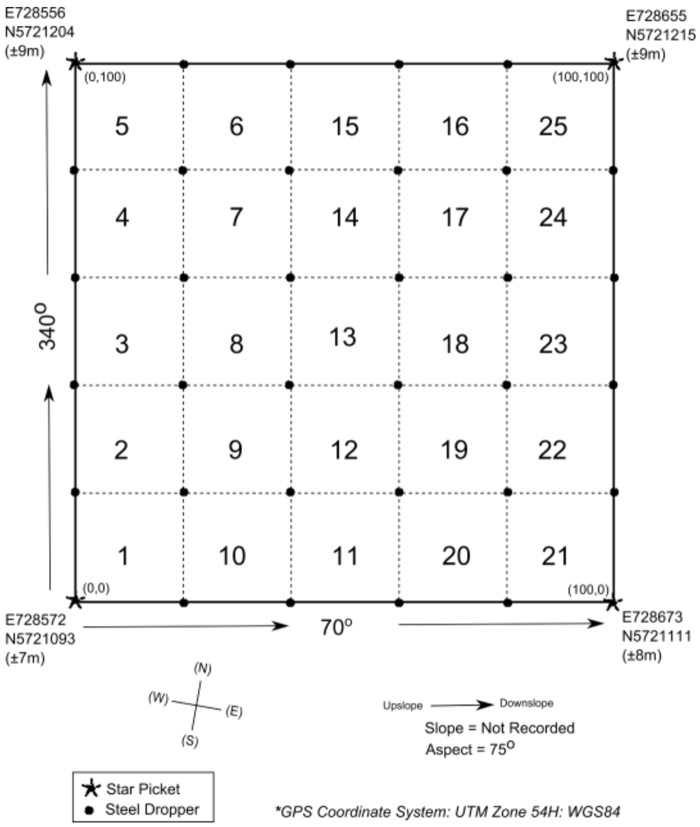

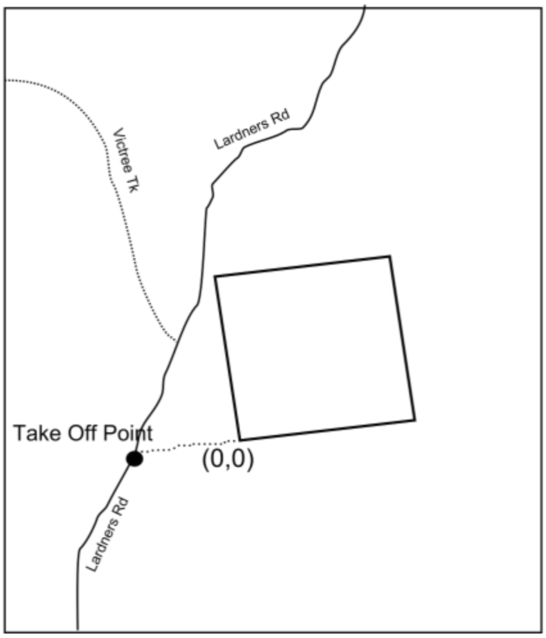

VCFSEH005: Lardner

**Target Eucalypt Species:**	*Eucalyptus regnans*	**High severity fire?**	Yes, 1939 (known fire event, ANU)
**Maximum Tree Height (m)**	82m	**Low severity fire?**	No
**Target Species Growth Stage:**	Mature, 1939 regrowth	**Cut stumps?**	No
**Understorey:**	Wet Sclerophyll	**Other Disturbance?**	No

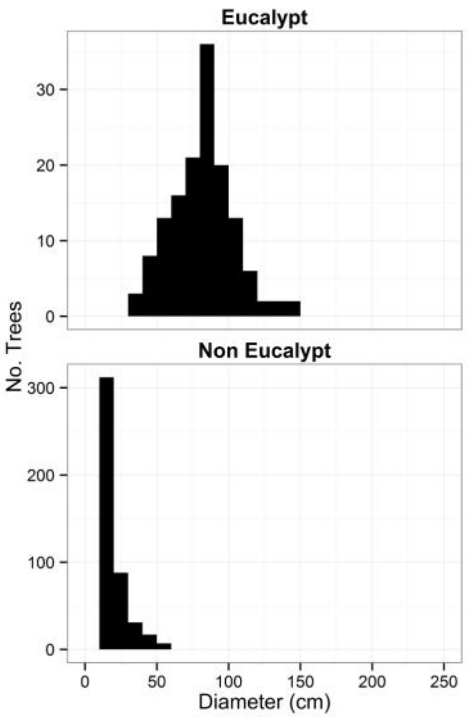

Species	No. Stems	BA (m^2/ha)
Eucalyptus regnans	142	66.1
Acacia melanoxylon	77	7.7
Olearia argophylla	220	4.5
Nematolepis squamea	66	2.4
Hedycarya angustifolia	67	0.8
Pittosporum bicolor	14	0.3
Pomaderris aspera	8	0.1
Coprosma quadrifida	5	0.1

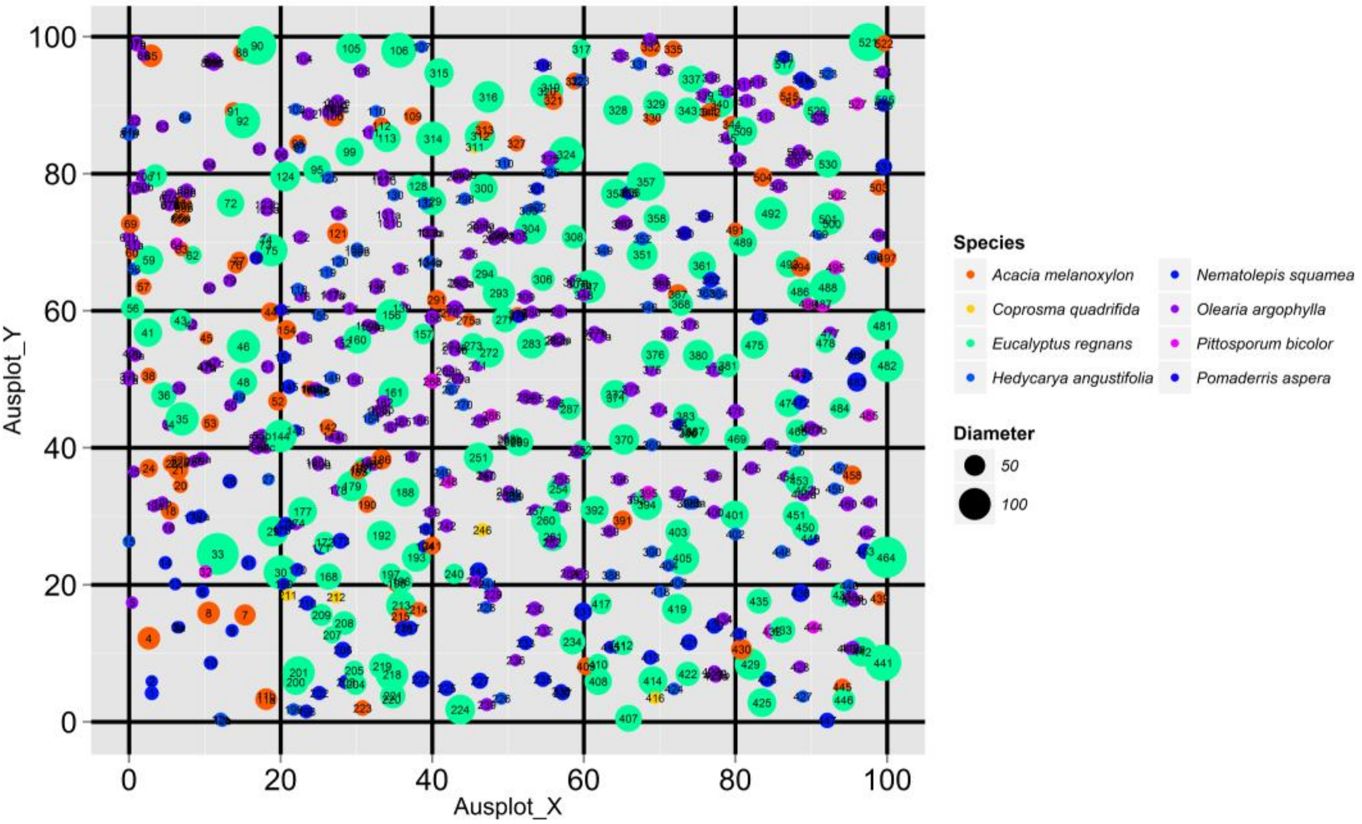

VCFSEH006: Black Spur/ANU363

**AusPlot ID**	VCFSEH006	**Elevation**	580m
**AusPlot Name**	Black Spur/ANU363	**Aspect**	80°
**State**	Victoria	**Slope**	Not recorded; Moderate
**Bioregion**	South East Highlands	**Landform Element**	Midslope
**Location (UTM)**	55 H 378765 5838478	**MAT, MAP**	11.7 ^{o}C, 1445 mm
**Location (Lat/Long)**	-37.5936 145.6263	**Existing Plot Custodian**	Australian National University (Lindenmayer)
**Tenure**	Yarra Ranges Nat. Park	**Existing Plot ID**	Ecological Research Plot 363
**Plot Est. Date**	05 April 2014	**Existing Plot Area**	2.0 ha
**Plot Size**	1.0ha (100mx100m)	**Existing Plot Census**	Early 1980's, ongoing.

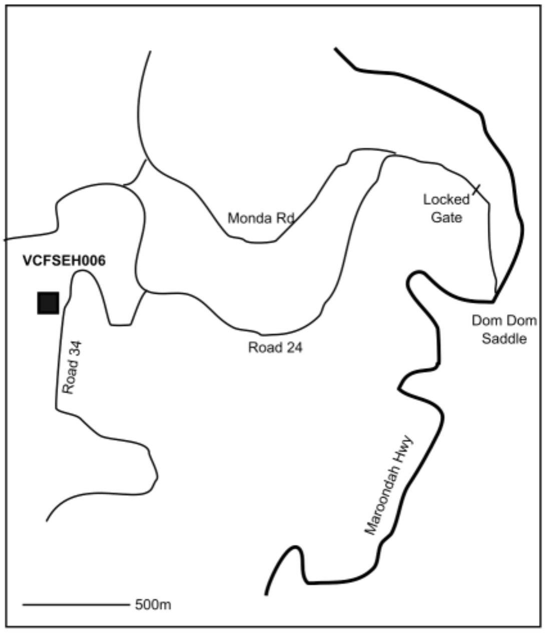

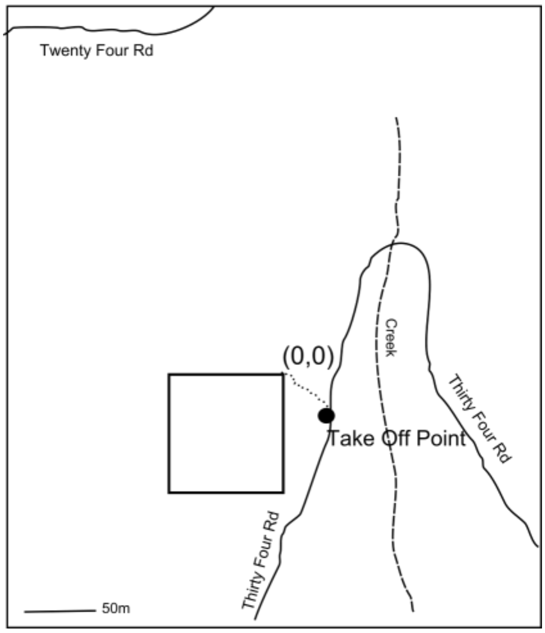

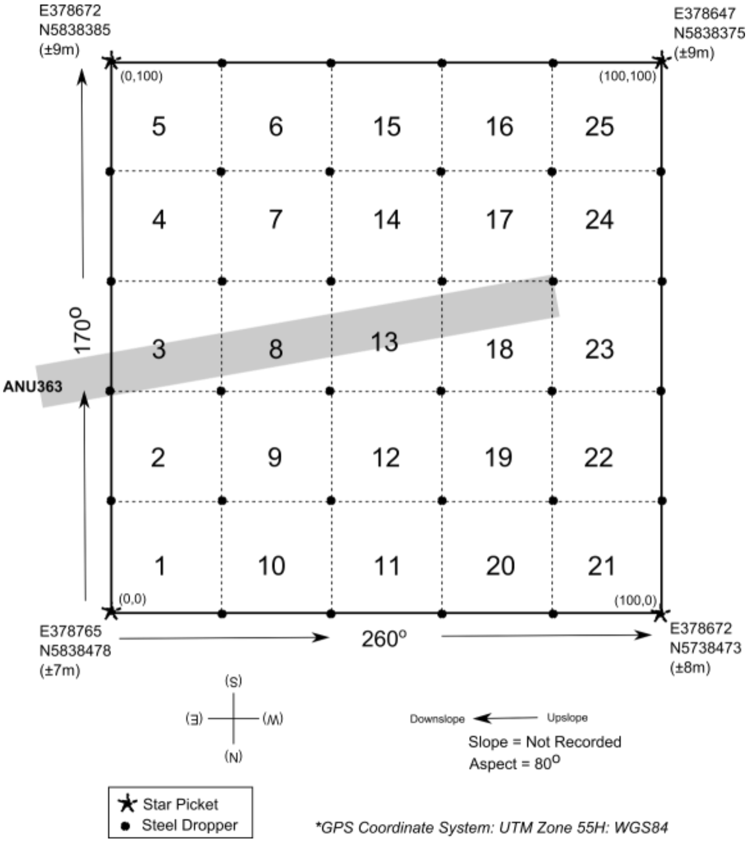

VCFSEH006: Black Spur/ANU363

**Target Eucalypt Species:**	*Eucalyptus regnans*	**High severity fire?**	Yes, 1939 (known fire event, ANU)
**Maximum Tree Height (m)**	78m	**Low severity fire?**	Yes, 2009 low-medium severity wildfire
**Target Species Growth Stage:**	Mature, 1939 regrowth	**Cut stumps?**	No
**Understorey:**	Wet Sclerophyll	**Other Disturbance?**	No

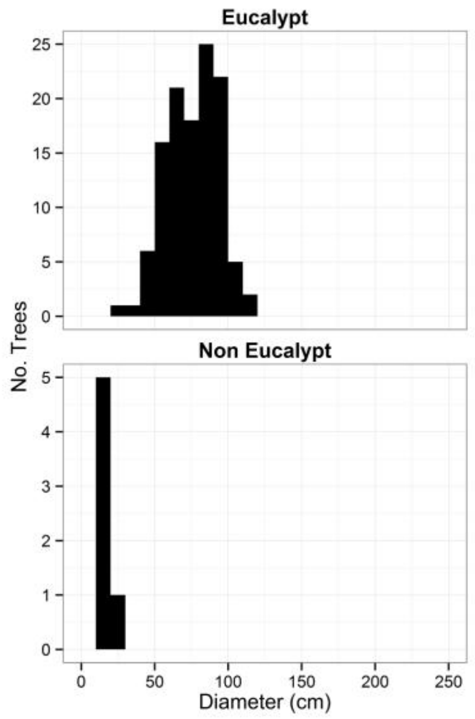

Species	No. Stems	BA (m^2/ha)
Eucalyptus regnans	117	49.9
Acacia dealbata	5	0.1
Persoonia arborea	1	<0.1

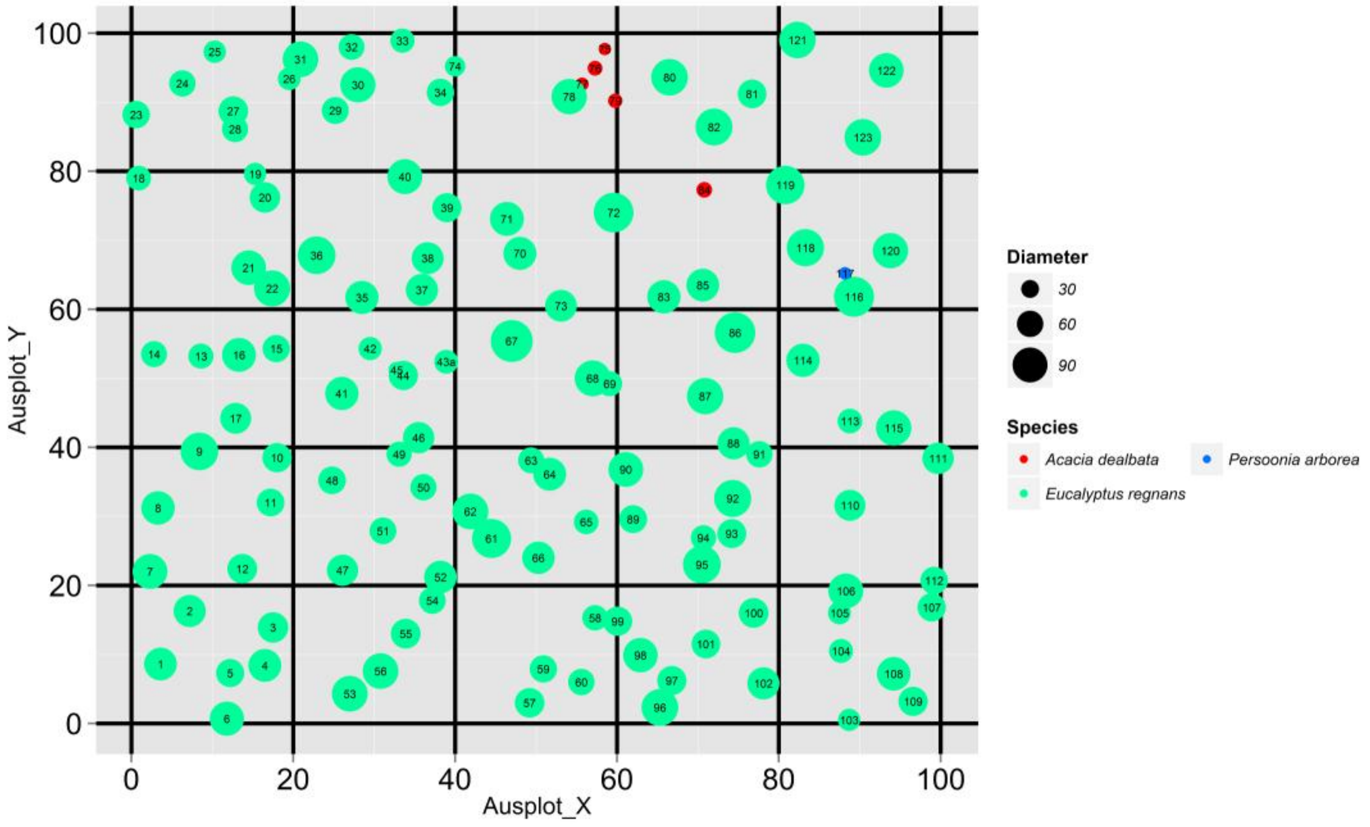

VCFSEH007: Monda/ANU589

**AusPlot ID**	VCFSEH007	**Elevation**	579m
**AusPlot Name**	Monda/ANU589	**Aspect**	190°
**State**	Victoria	**Slope**	Not recorded; Gently Inclined
**Bioregion**	South East Highlands	**Landform Element**	Ridge
**Location (UTM)**	55 H 379931 5838904	**MAT, MAP**	11.6 ^{o}C, 1468 mm
**Location (Lat/Long)**	-37.5893 145.6396	**Existing Plot Custodian**	Australian National University (Lindenmayer)
**Tenure**	Yarra Ranges Nat. Park	**Existing Plot ID**	Ecological Research Plot 589
**Plot Est. Date**	13 April 2014	**Existing Plot Area**	2.0 ha
**Plot Size**	1.0ha (100mx100m)	**Existing Plot Census**	Early 1980’s, ongoing.

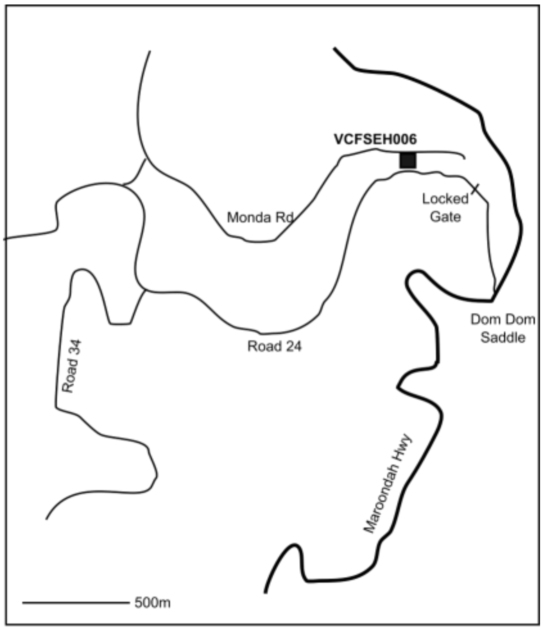

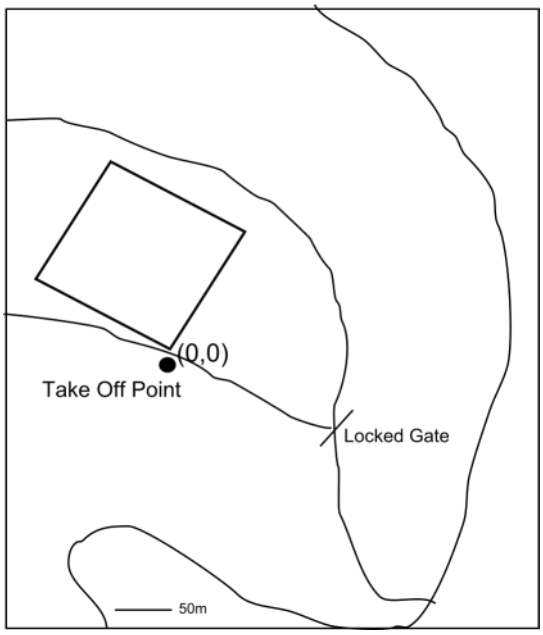

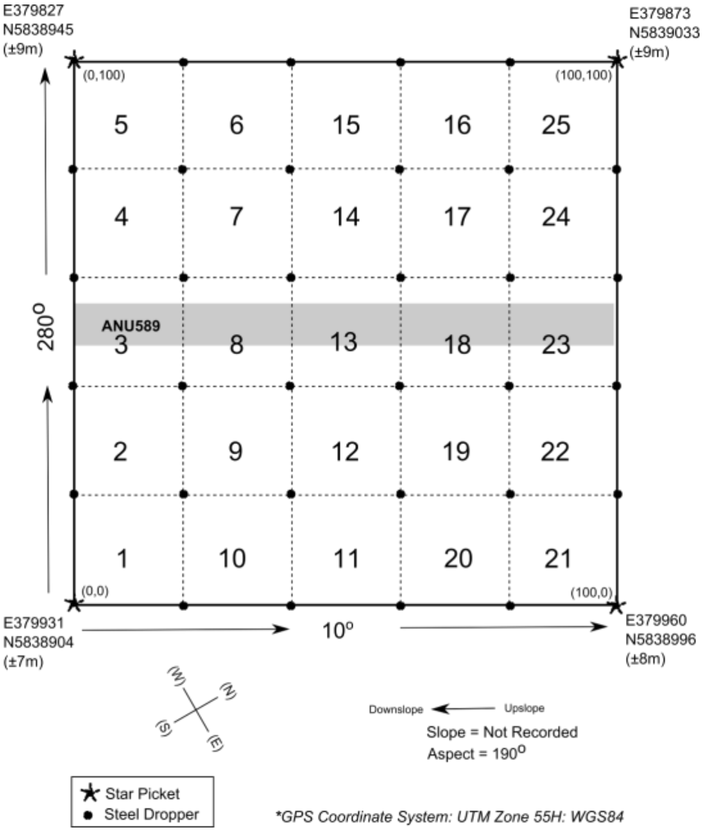

VCFSEH007: Monda/ANU589

**Target Eucalypt Species:**	*Eucalyptus regnans*	**High severity fire?**	Yes, 1939 (known fire event, ANU)
**Maximum Tree Height (m)**	83m	**Low severity fire?**	No
**Target Species Growth Stage:**	Mature, 1939 regrowth	**Cut stumps?**	No
**Understorey:**	Wet Sclerophyll	**Other Disturbance?**	No

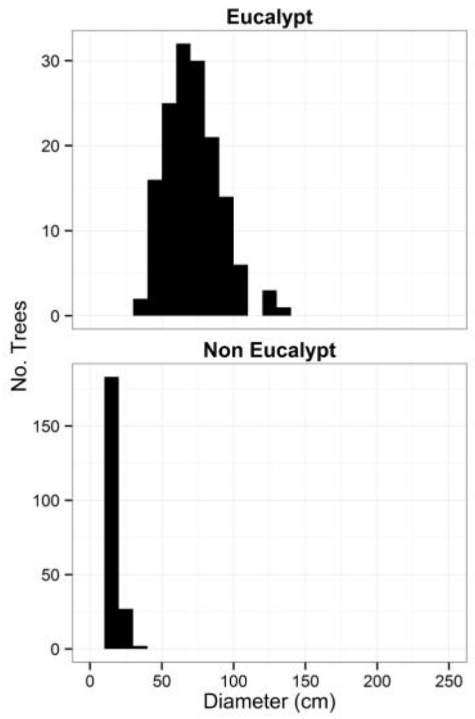

Species	No. Stems	BA (m^2/ha)
Eucalyptus regnans	150	58.1
Pomaderris aspera	113	2.3
Olearia argophylla	48	0.9
Acacia melanoxylon	12	0.5
Lomatia fraseri	26	0.3
Bedfordia arborescens	11	0.3
Tasmannia lanceolata	2	<0.1

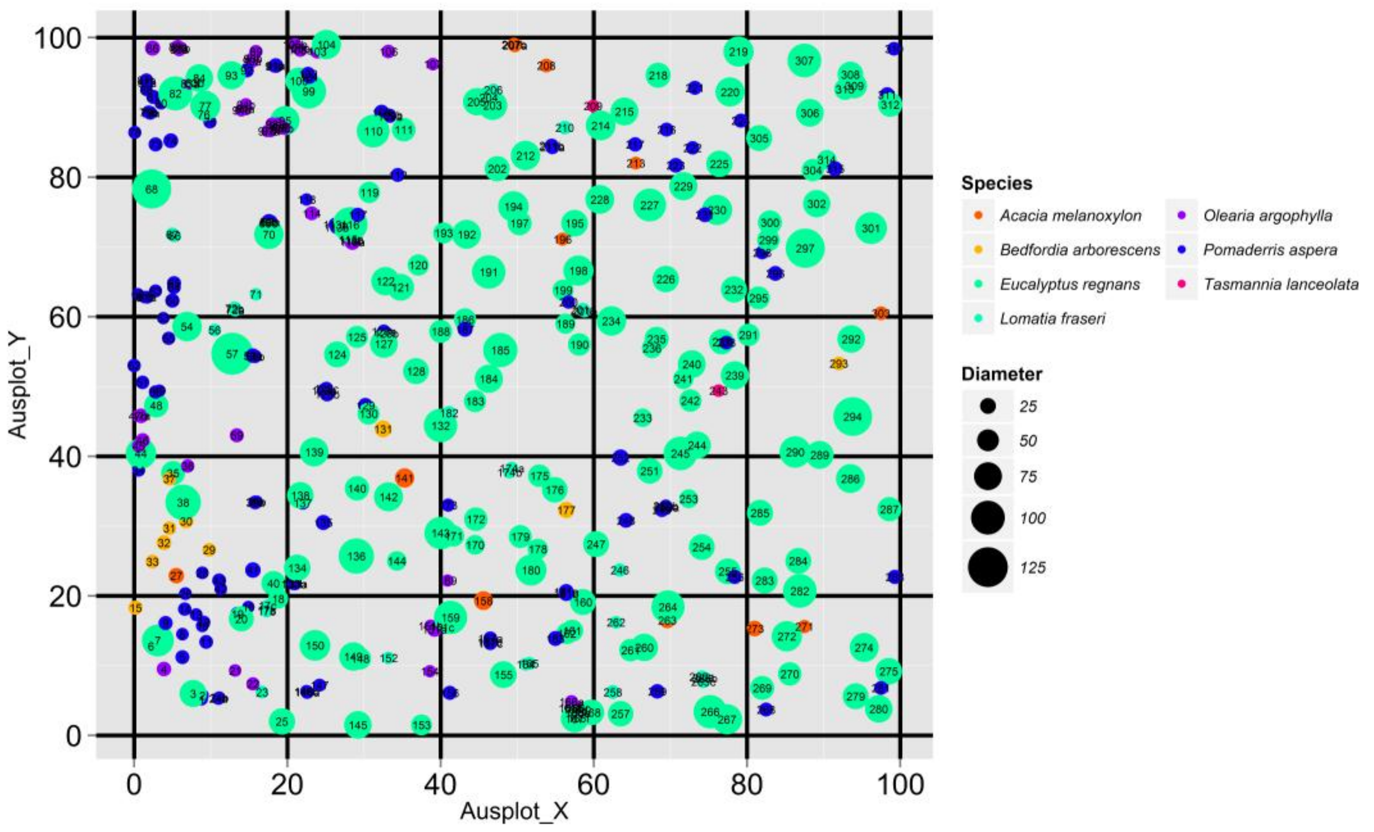

VCFSEH008: HardyCreek

**AusPlot ID**	VCFSEH008	**Elevation**	830m
**AusPlot Name**	Toolangi	**Aspect**	25°
**State**	Victoria	**Slope**	12°; Gently Inclined
**Bioregion**	South East Highlands	**Landform Element**	Ridge
**Location (UTM)**	55 H 371638 5840980	**MAT, MAP**	10.5 ^{o}C, 1641 mm
**Location (Lat/Long)**	-37.5693 145.5465	**Existing Plot Custodian**	VicForests
**Tenure**	Multiple-Use Forest	**Existing Plot ID**	Permanent Inventory Plot V44 (V44/1)
**Plot Est. Date**	28 April 2014	**Existing Plot Area**	2.0 ha
**Plot Size**	1.0ha (100mx100m)	**Existing Plot Census**	1970,71,74,76,77,80,81,83,86,90,91,94,97,98,00,06

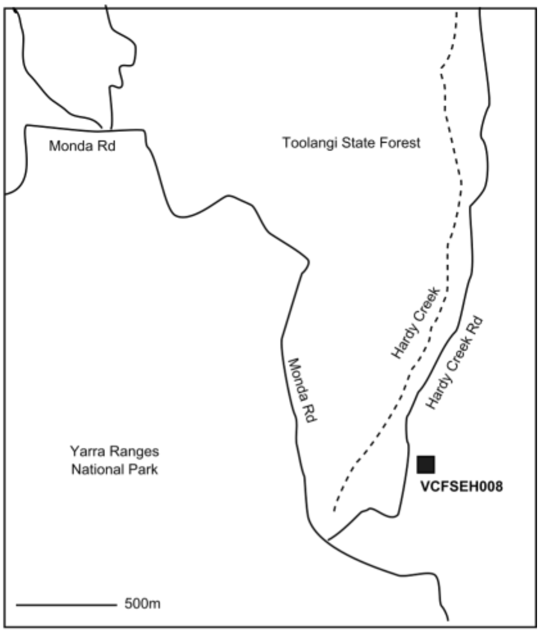

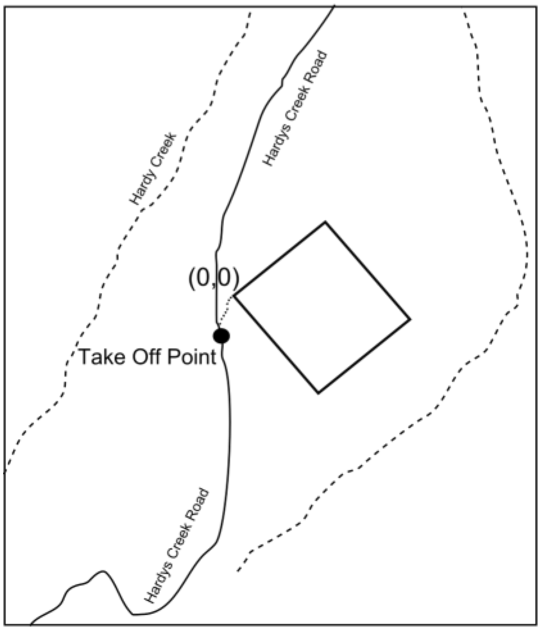

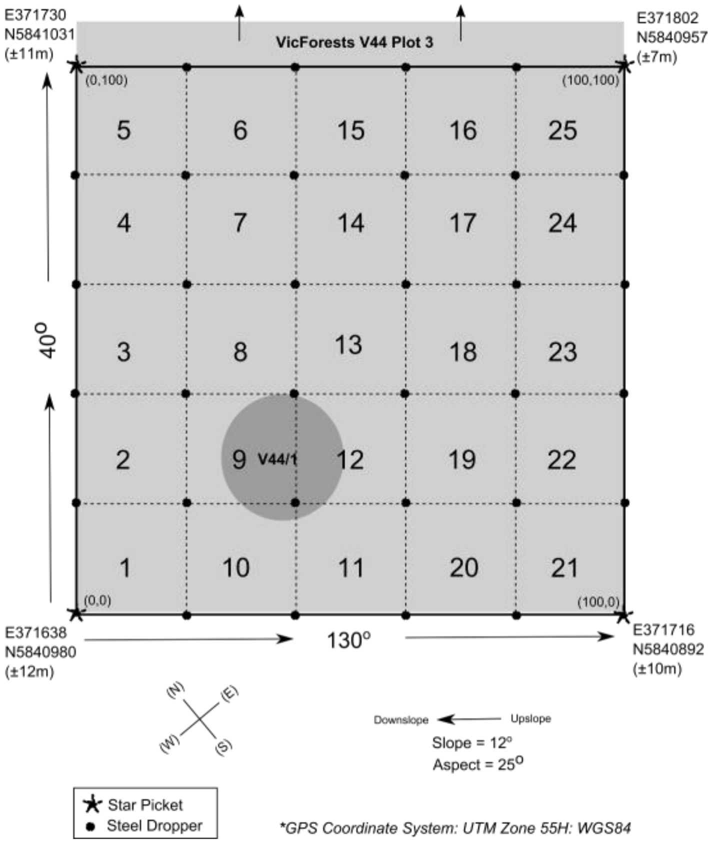

VCFSEH008: HardyCreek

**Target Eucalypt Species:**	*Eucalyptus regnans*	**High severity fire?**	Yes, 1939 (known fire event, ANU)
**Maximum Tree Height (m)**	89m	**Low severity fire?**	No
**Target Species Growth Stage:**	Mature, 1939 regrowth	**Cut stumps?**	No
**Understorey:**	Wet Sclerophyll	**Other Disturbance?**	No

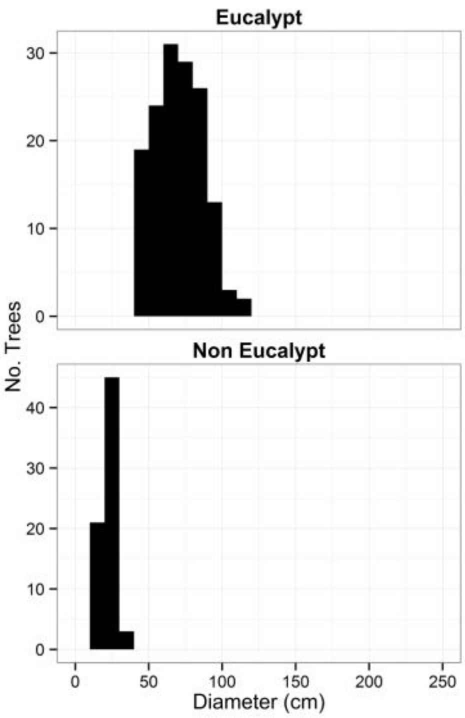

Species	No. Stems	BA (m^2/ha)
Eucalyptus regnans	145	53.1
Acacia melanoxylon	62	2.7
Eucalyptus nitens	2	1.2
Correa lawrenceana	2	<0.1
Pittosporum bicolor	2	<0.1
Persoonia arborea	1	<0.1
Lomatia fraseri	1	<0.1
Tasmannia lanceolata	1	<0.1

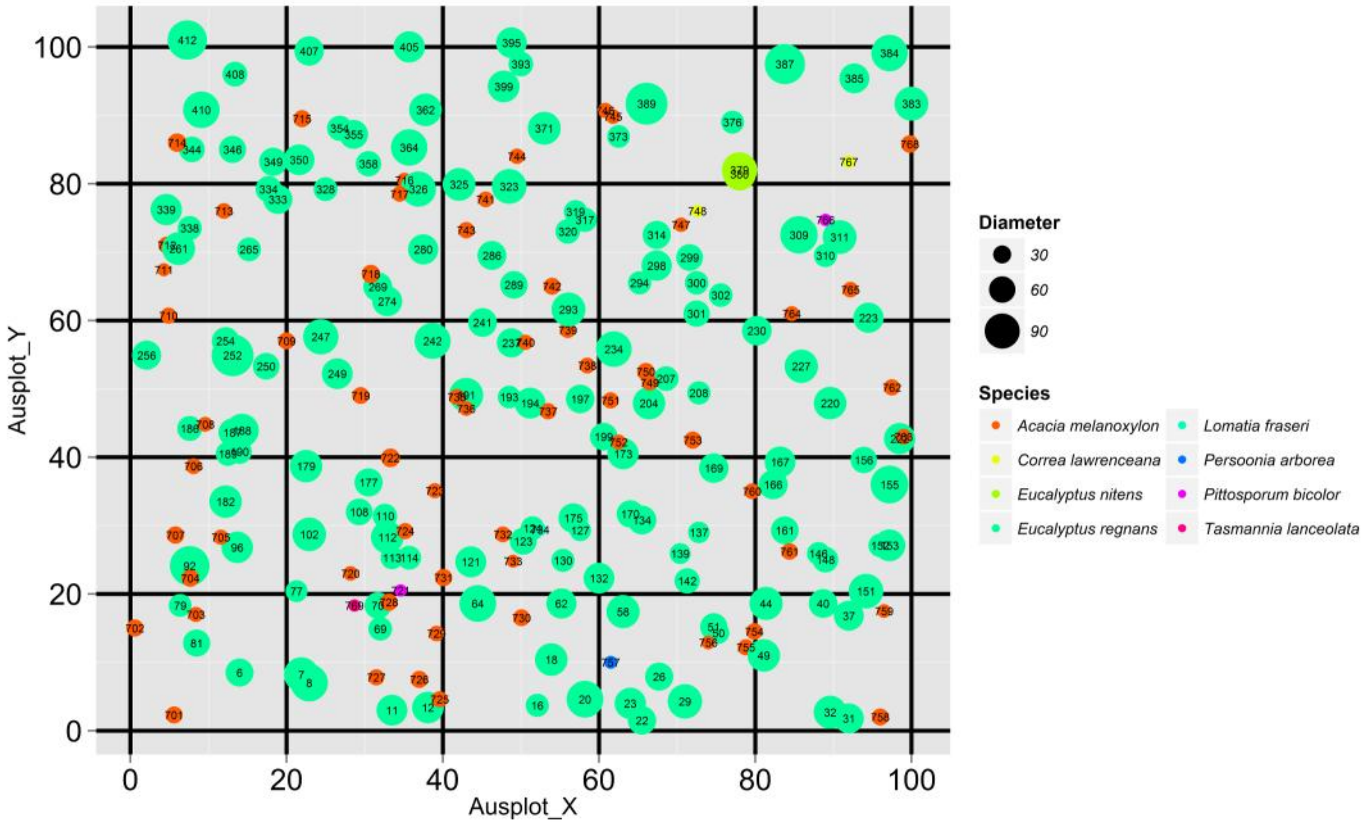

Southern New South Wales (NSF)

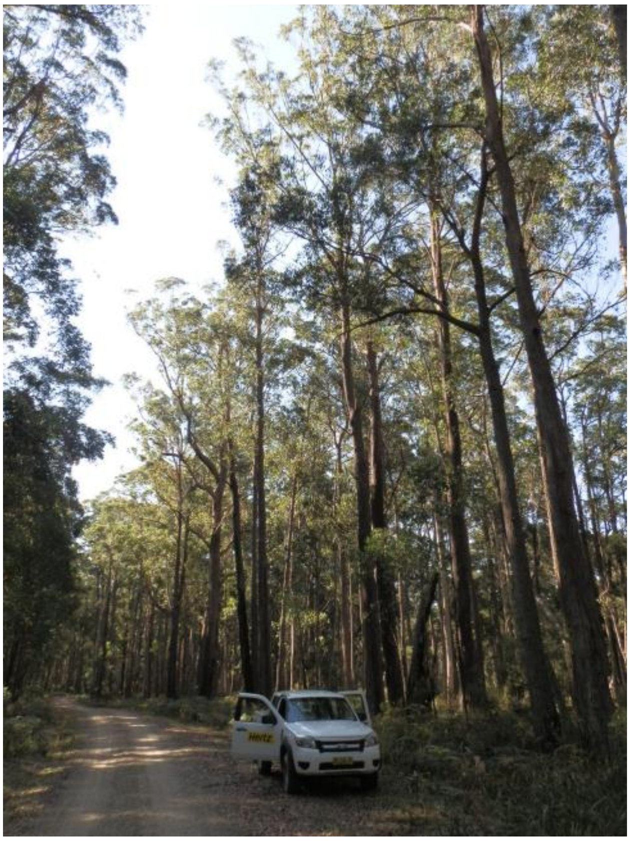

Eucalyptus fastigata at NSFSEC001 (Bombala)

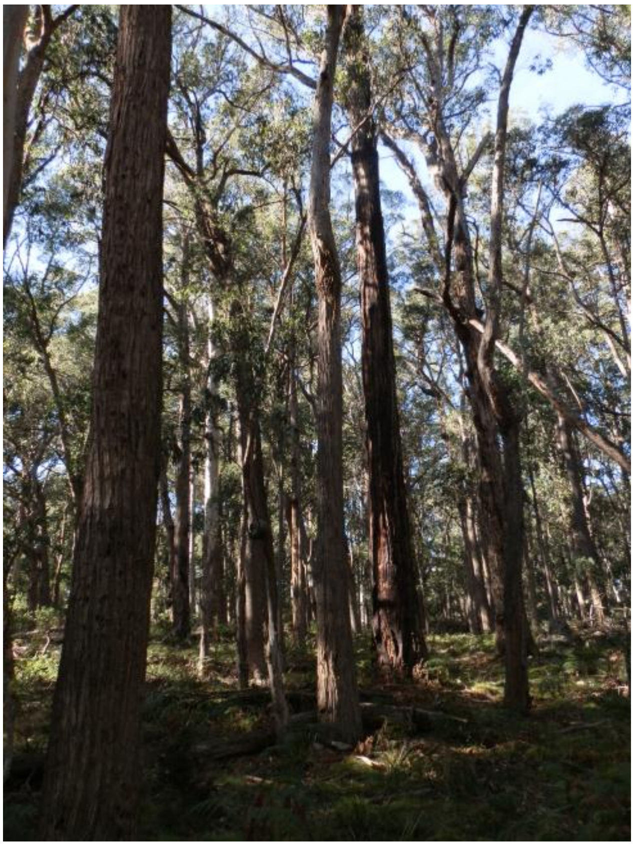

Mixed *Eucalypt* forest at at NSFSEC002 (Bombala)

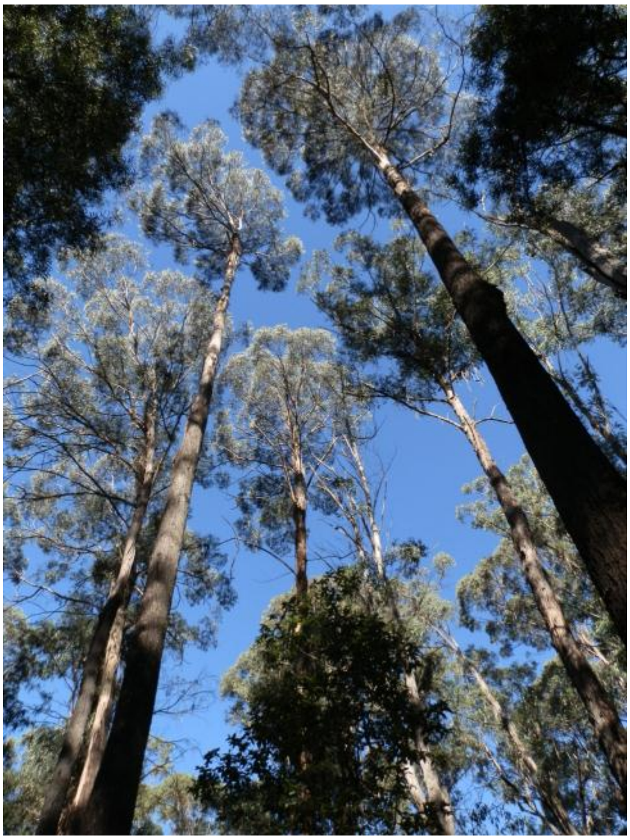

Eucalyptus fastigata at NSFSEC004 (Bombala)

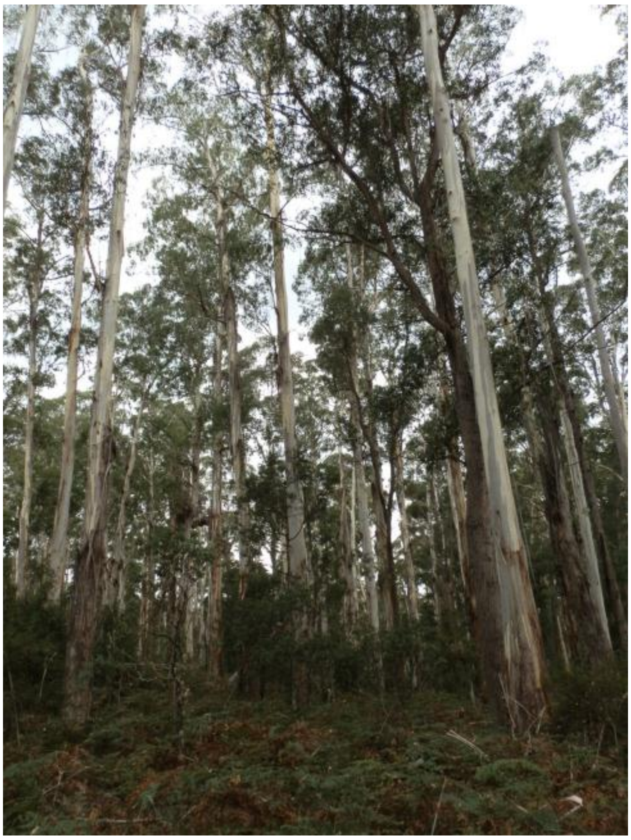

Eucalyptus viminalis at NSFSEC003 (Bombala)

Southern New South Wales (NSF)

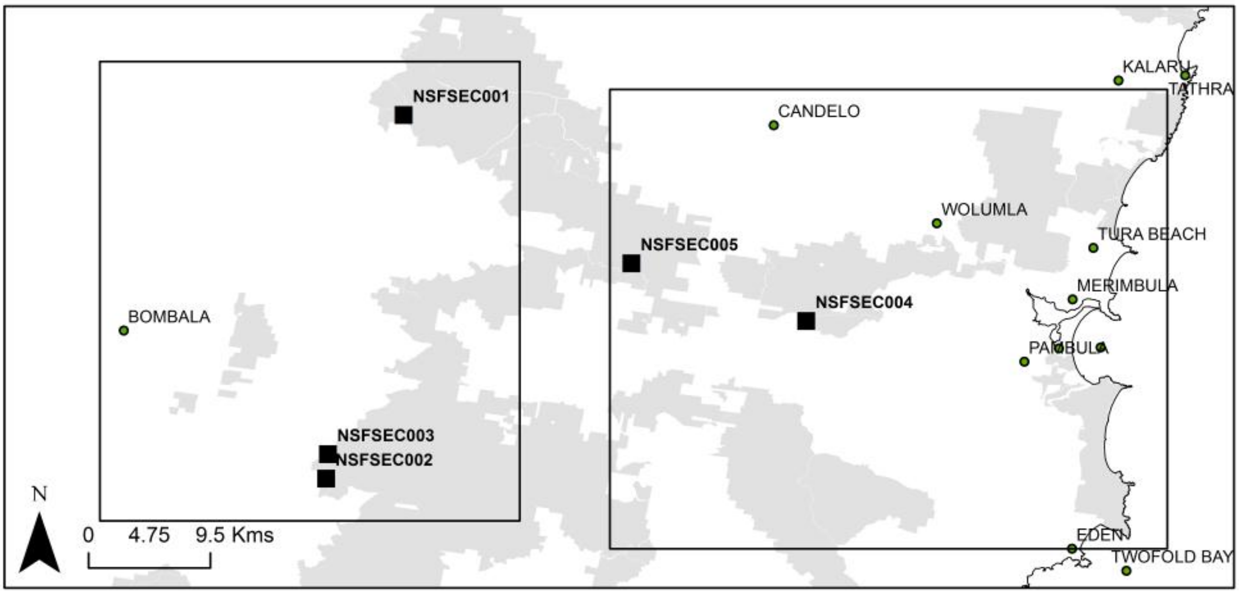

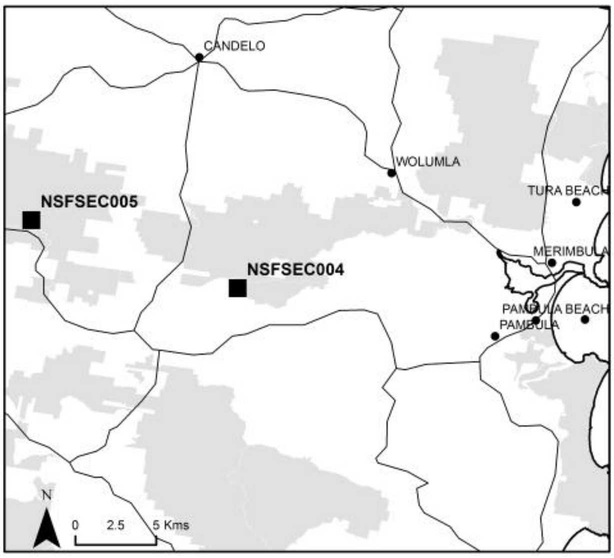

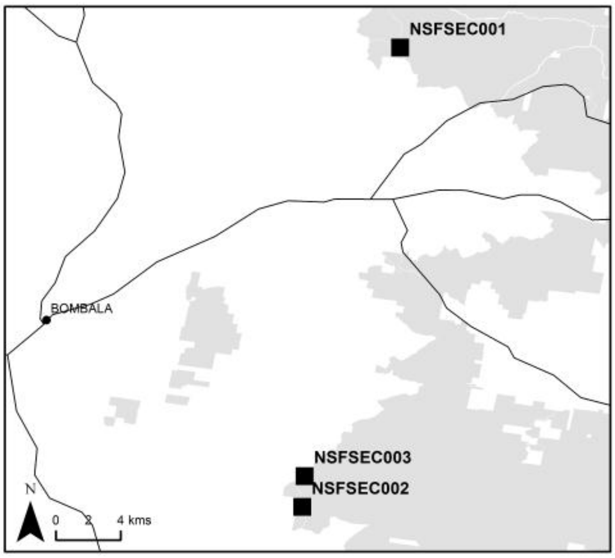

NSFSEC001: Newline

**AusPlot ID**	NSFSEC001	**Elevation**	955m
**AusPlot Name**	Newline	**Aspect**	260°
**State**	New South Wales	**Slope**	4°; Gently Inclined
**Bioregion**	South East Corner	**Landform Element**	Ridge
**Location (UTM)**	55 H 717368 5929078	**MAT, MAP**	10.0 ^{o}C, 853 mm
**Location (Lat/Long)**	-36.7591 149.4351	**Existing Plot Custodian**	NA
**Tenure**	South East Forests NP	**Existing Plot ID**	NA
**Plot Est. Date**	10 May 2014	**Existing Plot Area**	NA
**Plot Size**	1.0ha (100mx100m)	**Existing Plot Census**	NA

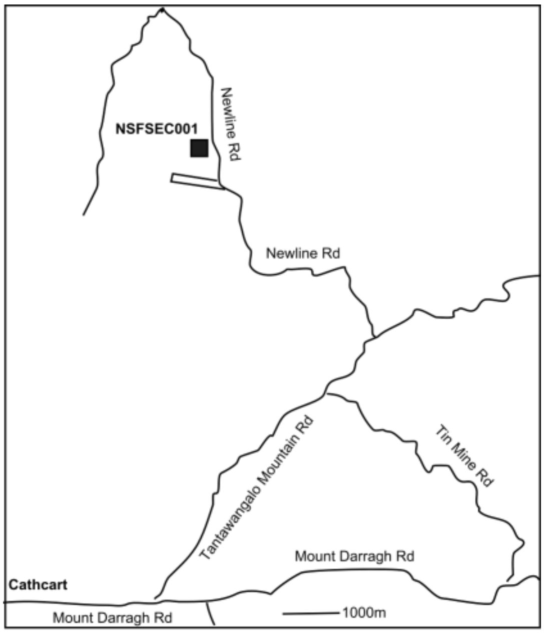

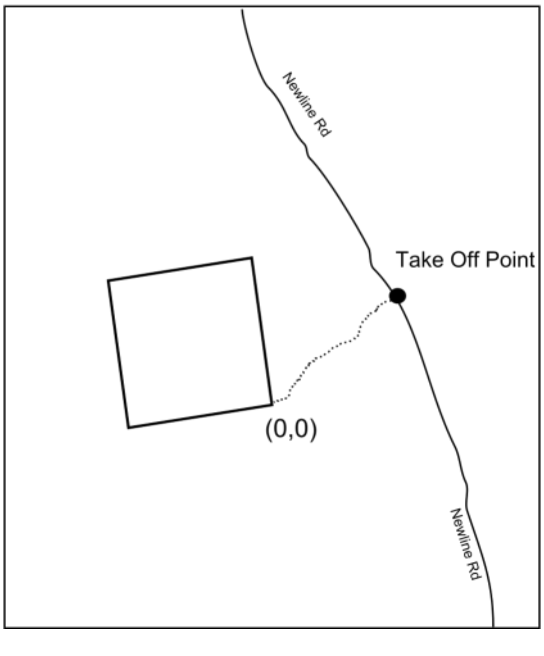

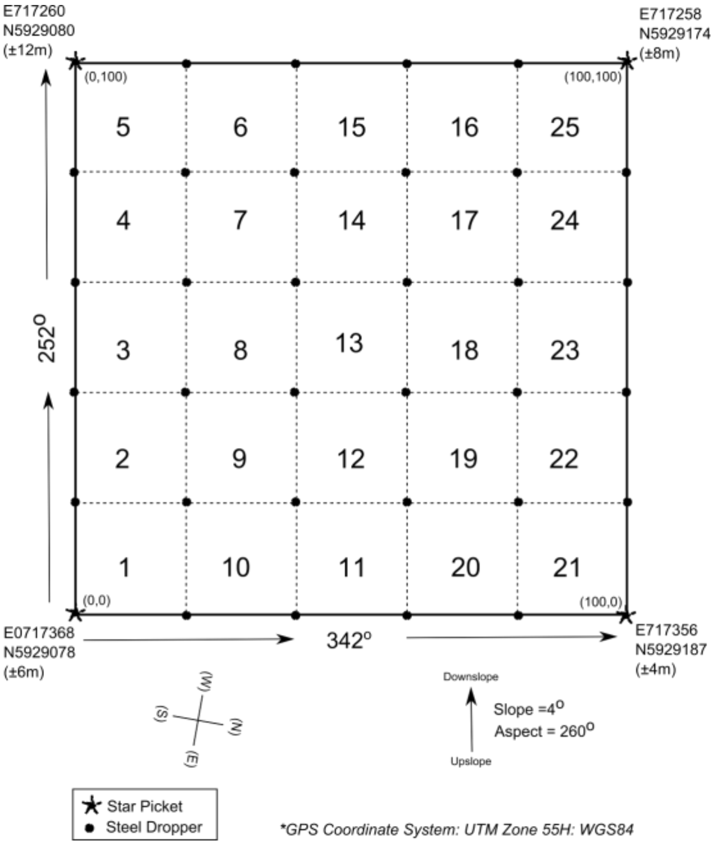

NSFSEC001: Newline

**Target Eucalypt Species:**	*Eucalyptus fastigata*	**High severity fire?**	Unknown
**Maximum Tree Height (m)**	49m	**Low severity fire?**	Yes, frequency unknown (Fire Scars)
**Target Species Growth Stage:**	Mature	**Cut stumps?**	Yes, 12 chainsawed stumps
**Understorey:**	Wet Sclerophyll	**Other Disturbance?**	No

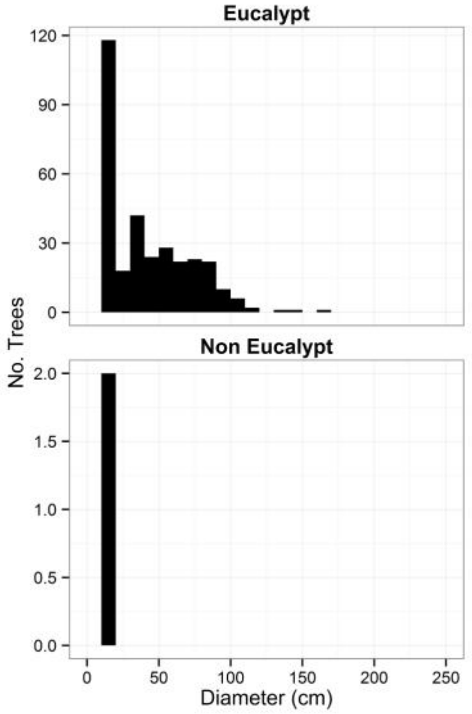

Species	No. Stems	BA (m^2/ha)
Eucalyptus fastigata	144	35.5
Eucalyptus cypellocarpa	91	20.4
Eucalyptus obliqua	64	7.7
Eucalyptus radiata	19	0.6
Persoonia silvatica	1	<0.1
Acacia dealbata	1	<0.1

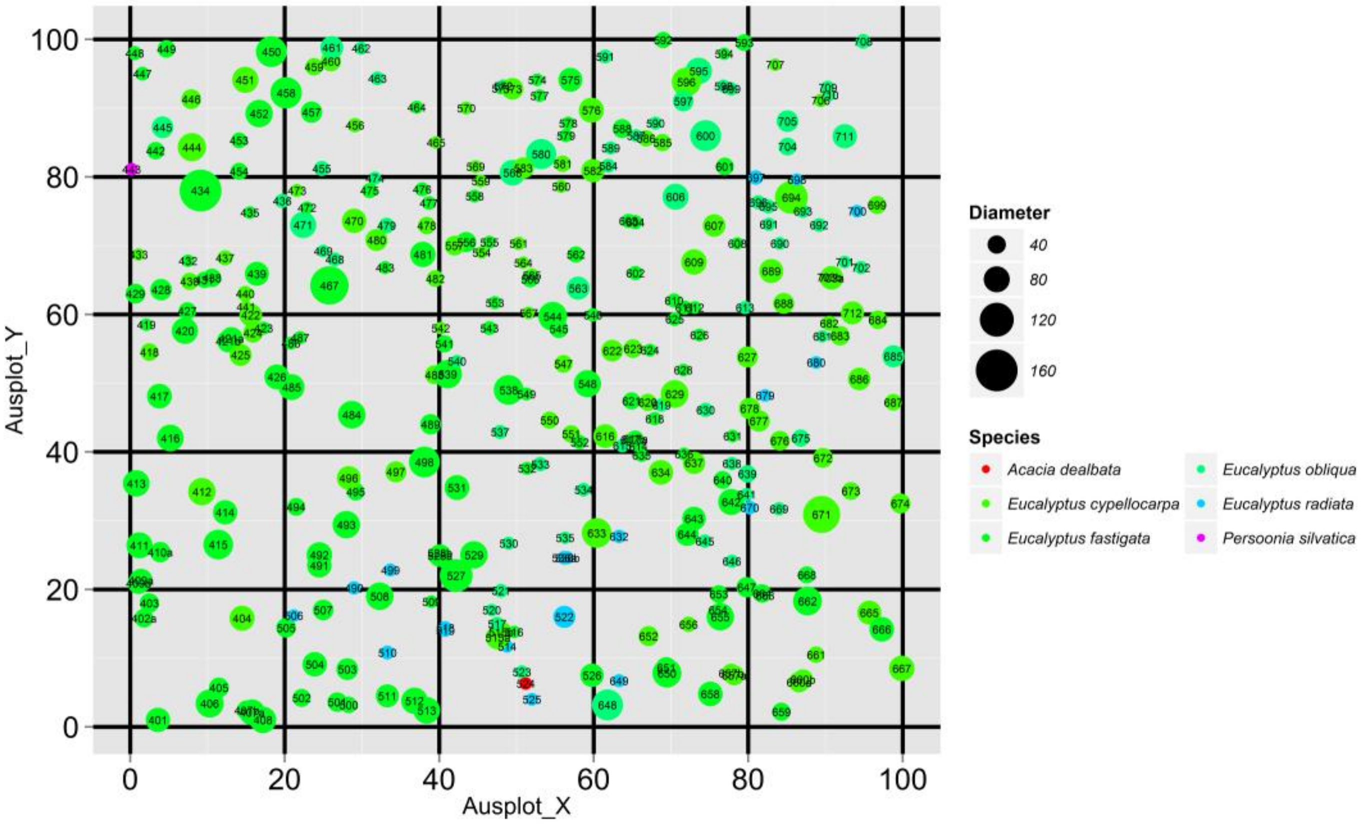

NSFSEC002: Waratah Mix

**AusPlot ID**	NSFSEC002	**Elevation**	828m
**AusPlot Name**	Waratah Mix	**Aspect**	345°
**State**	New South Wales	**Slope**	3°; Gently Inclined
**Bioregion**	South East Corner	**Landform Element**	Flat Ridge
**Location (UTM)**	55 H 711875 5901031	**MAT, MAP**	10.4 ^{o}C, 917 mm
**Location (Lat/Long)**	-36.9969 149.3821	**Existing Plot Custodian**	Rod Kavanagh (ForestsNSW)
**Tenure**	South East Forests NP	**Existing Plot ID**	Ecological Research Plot
**Plot Est. Date**	18 May 2014	**Existing Plot Area**	100ha
**Plot Size**	1.0ha (100mx100m)	**Existing Plot Census**	1984 onwards.

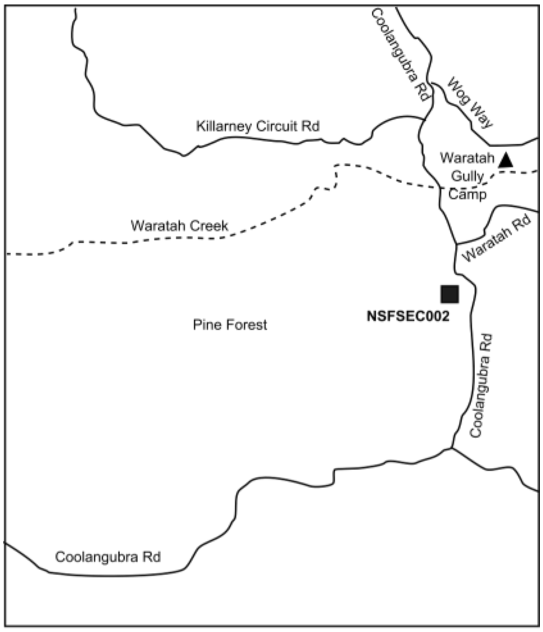

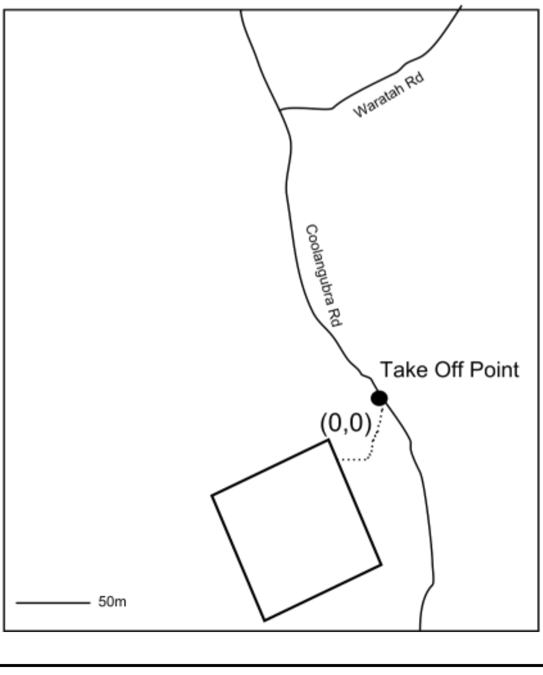

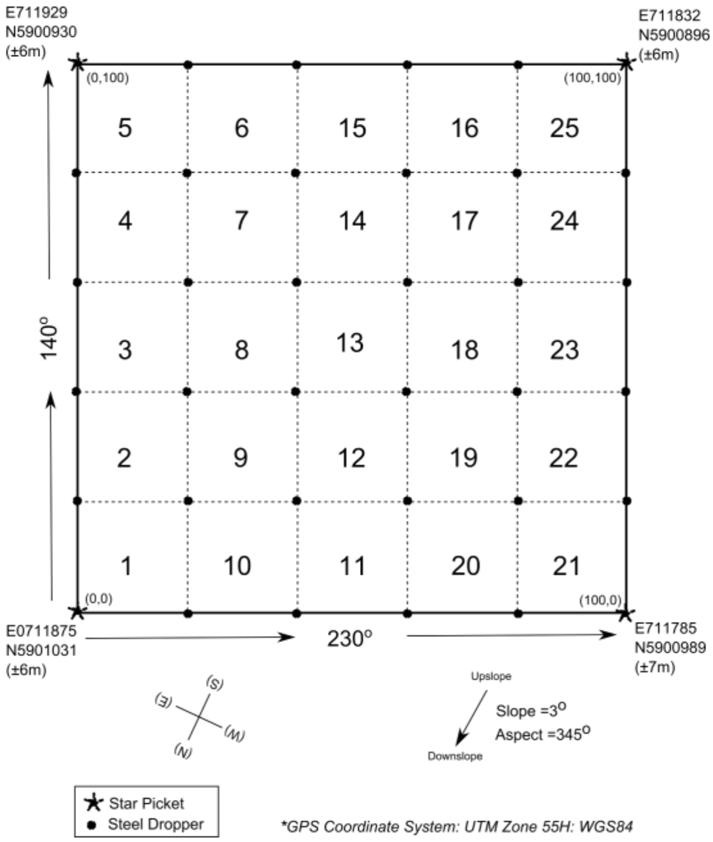

NSFSEC002: Waratah Mix

**Target Eucalypt Species:**	*Eucalyptus fastigata*	**High severity fire?**	Unknown
**Maximum Tree Height (m)**	48m	**Low severity fire?**	Yes, frequency unknown (Fire Scars)
**Target Species Growth Stage:**	Mature	**Cut stumps?**	No
**Understorey:**	Wet Sclerophyll	**Other Disturbance?**	No

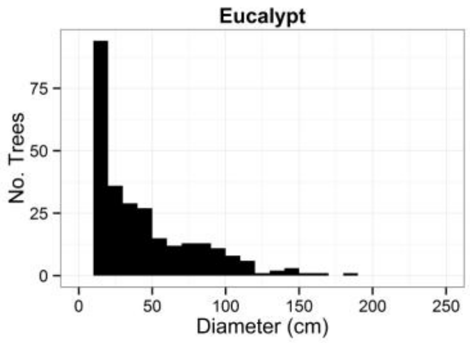

Species	No. Stems	BA (m^2/ha)
Eucalyptus fastigata	67	20.7
Eucalyptus obliqua	59	19.4
Eucalyptus cypellocarpa	46	13.3
Eucalyptus radiata	93	9.6
Eucalyptus viminalis	7	0.9
Eucalyptus ovata	1	<0.1

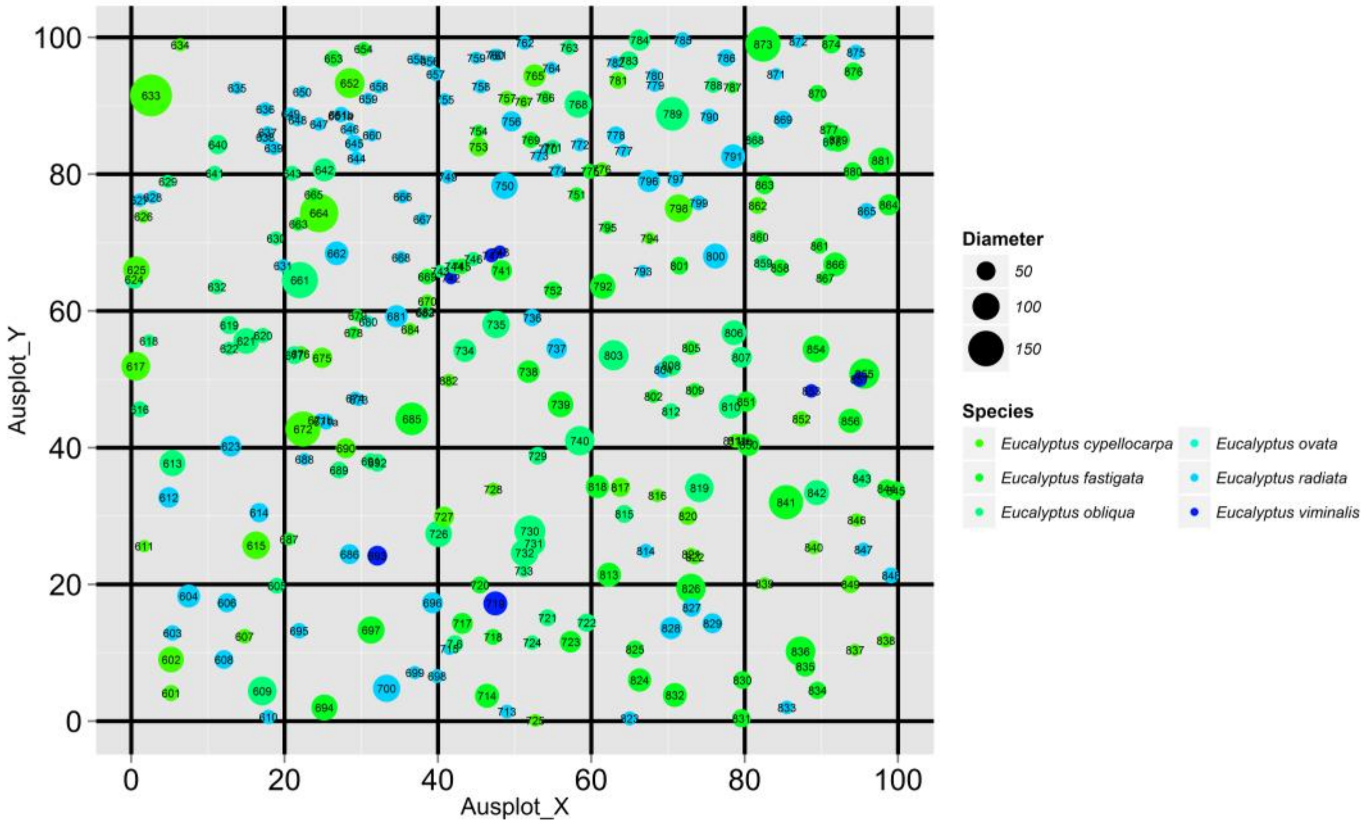

NSFSEC003: WogWay

**AusPlot ID**	NSFSEC003	**Elevation**	845m
**AusPlot Name**	WogWay	**Aspect**	170°
**State**	New South Wales	**Slope**	15°; Moderate
**Bioregion**	South East Corner	**Landform Element**	Midslope
**Location (UTM)**	55 H 711994 5902761	**MAT, MAP**	10.9 °C, 911 mm
**Location (Lat/Long)**	-37.0140 149.3808	**Existing Plot Custodian**	NA
**Tenure**	South East Forests NP	**Existing Plot ID**	NA
**Plot Est. Date**	16 May 2014	**Existing Plot Area**	NA
**Plot Size**	1.0ha (100mx100m)	**Existing Plot Census**	NA

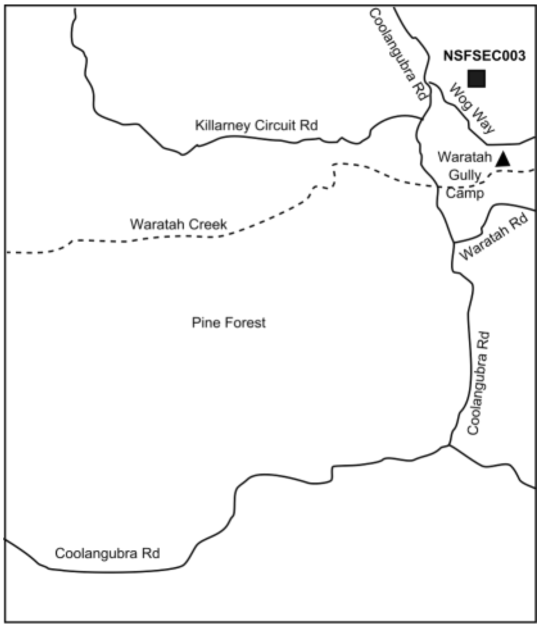

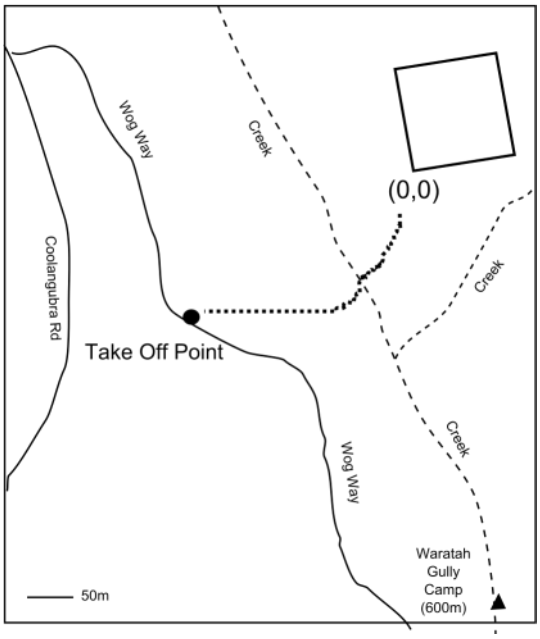

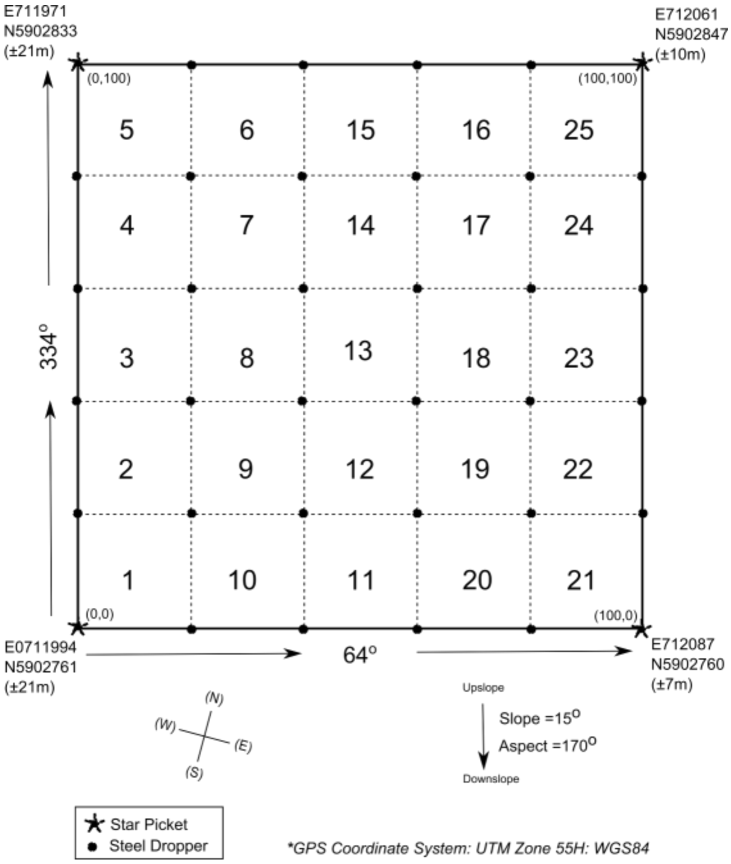

NSFSEC003: WogWay

**Target Eucalypt Species:**	*Eucalyptus fastigata*	**High severity fire?**	Unknown
**Maximum Tree Height (m)**	58m	**Low severity fire?**	Yes, frequency unknown (Fire Scars)
**Target Species Growth Stage:**	Mature	**Cut stumps?**	No
**Understorey:**	Wet Sclerophyll	**Other Disturbance?**	No

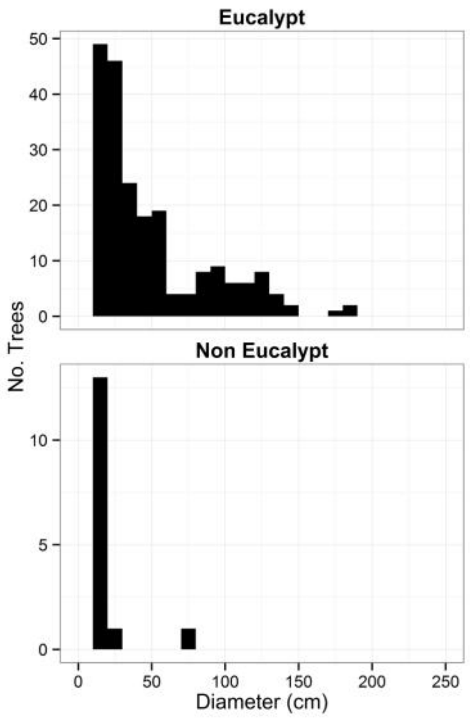

Species	No. Stems	BA (m^2/ha)
Eucalyptus viminalis	118	29.2
Eucalyptus fastigata	72	18.3
Eucalyptus obliqua	20	14.3
Acacia dealbata	5	0.5
Prostanthera lasianthos	6	0.1
Lomatia myricoides	4	<0.1

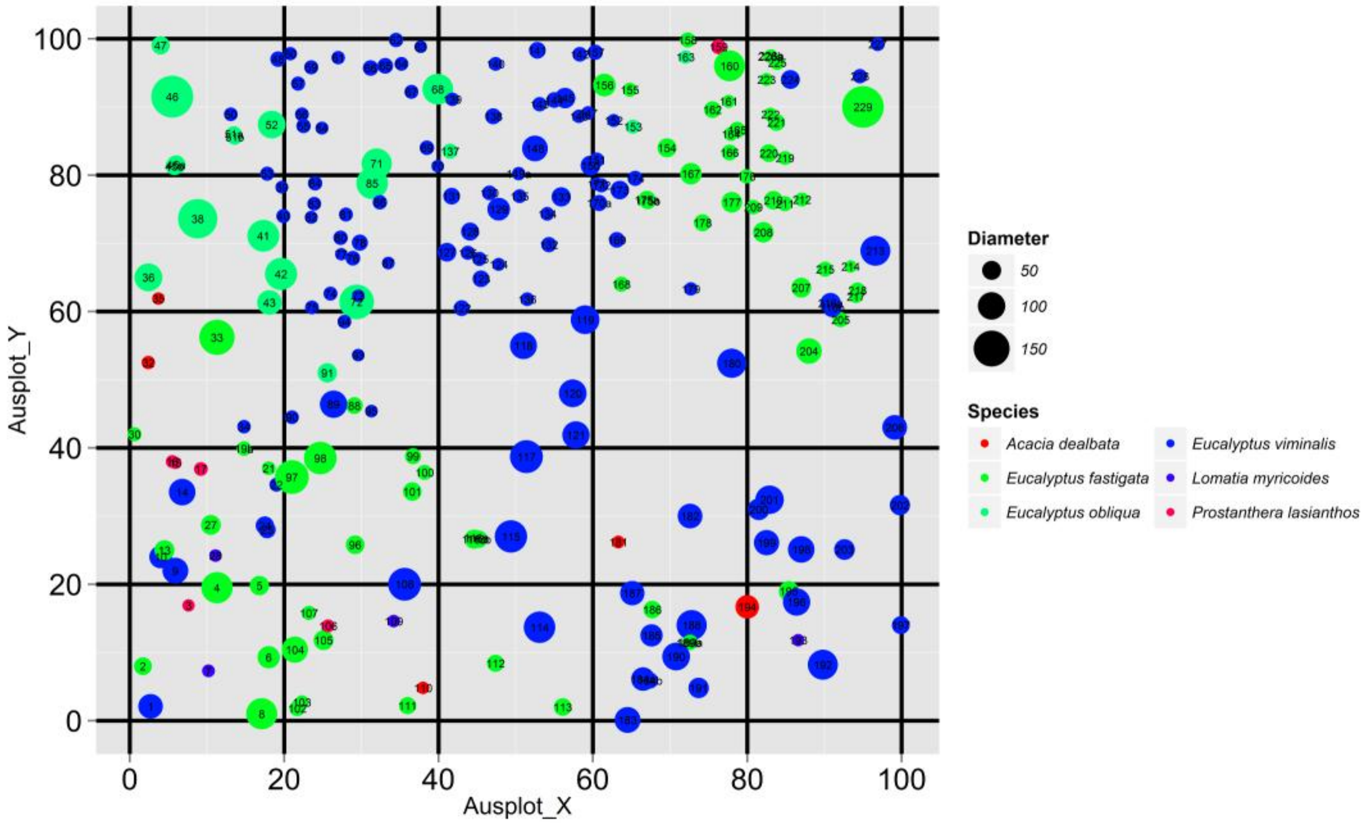

NSFSEC004: Goodenia

**AusPlot ID**	NSFSEC004	**Elevation**	420m
**AusPlot Name**	Goodenia	**Aspect**	65°
**State**	New South Wales	**Slope**	15°; Moderate
**Bioregion**	South East Corner	**Landform Element**	Midslope, Gully, Ridge
**Location (UTM)**	55 H 742155 5912353	**MAT, MAP**	13.1 ^{o}C, 1000 mm
**Location (Lat/Long)**	-36.9035 149.7176	**Existing Plot Custodian**	NA
**Tenure**	South East Forests NP	**Existing Plot ID**	NA
**Plot Est. Date**	12 May 2014	**Existing Plot Area**	NA
**Plot Size**	1.0ha (100mx100m)	**Existing Plot Census**	NA

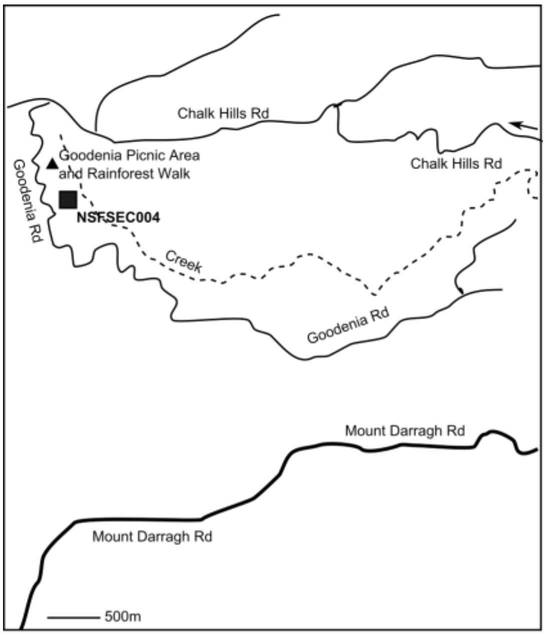

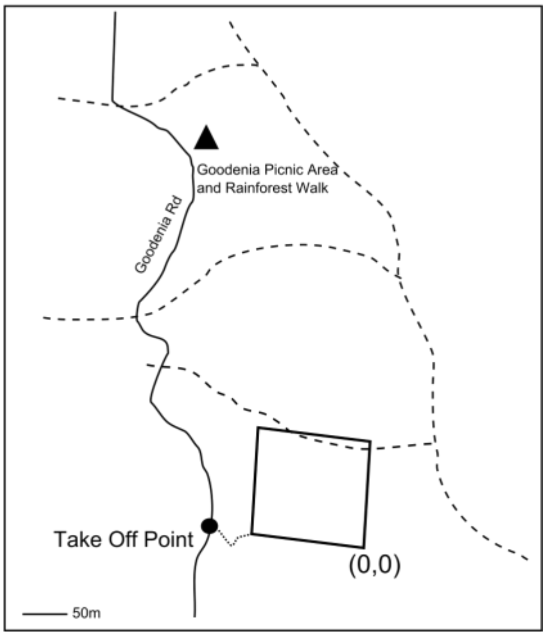

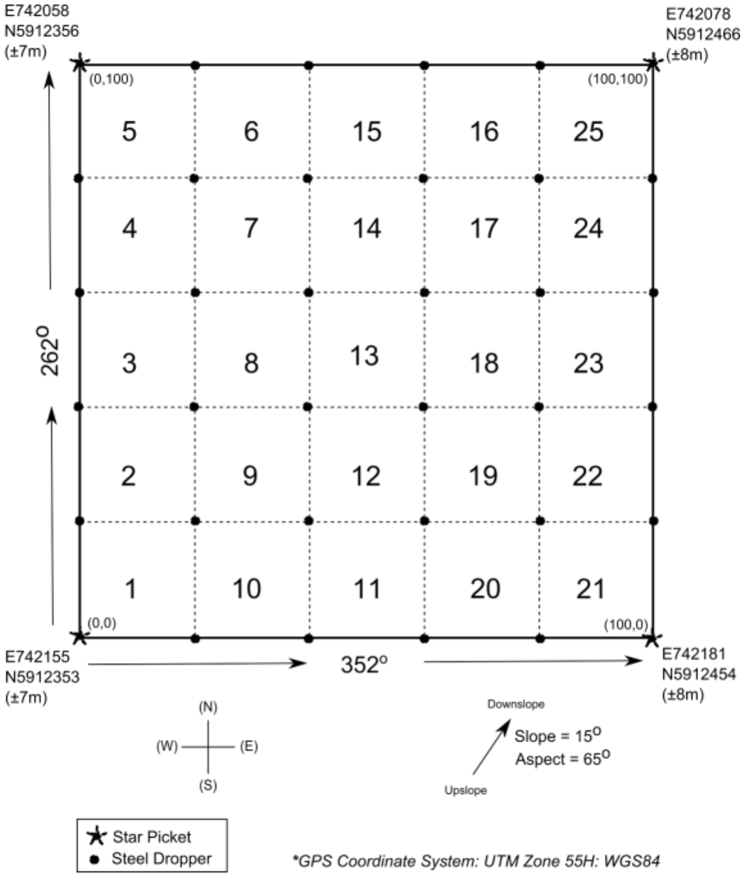

NSFSEC004: Goodenia

**Target Eucalypt Species:**	*Eucalyptus fastigata*	**High severity fire?**	Unknown
**Maximum Tree Height (m)**	64m	**Low severity fire?**	Yes, frequency unknown (Fire Scars)
**Target Species Growth Stage:**	Mature	**Cut stumps?**	No
**Understorey:**	Wet Sclerophyll	**Other Disturbance?**	No

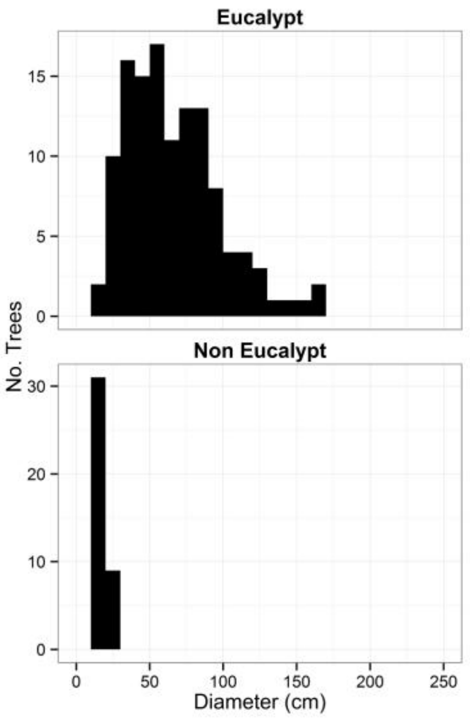

Species	No. Stems	BA (m^2/ha)
Eucalyptus fastigata	91	33.9
Eucalyptus cypellocarpa	8	9.5
Eucalyptus sieberi	22	7.0
Acacia melanoxylon	19	0.5
Olearia argophylla	10	0.1
Elaeocarpus holopetalus	6	0.1
Doryphora sassafras	4	0.1
Pittosporum undulatum	1	<0.1

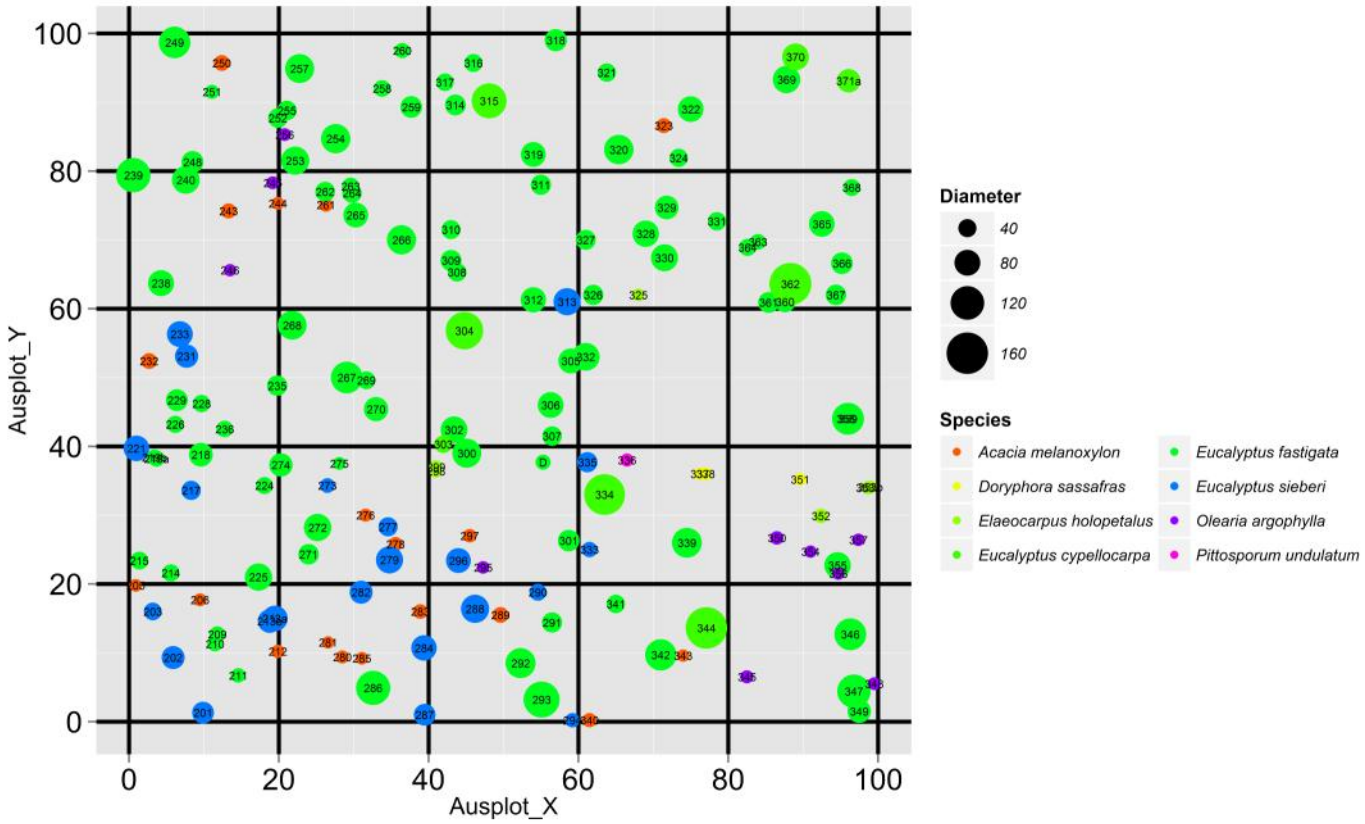

NSFSEC005: Candelo

**AusPlot ID**	NSFSEC005	**Elevation**	645m
**AusPlot Name**	Candelo	**Aspect**	27°
**State**	New South Wales	**Slope**	14°; Moderate
**Bioregion**	South East Corner	**Landform Element**	Midslope
**Location (UTM)**	55 H 731303 5917163	**MAT, MAP**	11.9 ^{o}C, 953 mm
**Location (Lat/Long)**	-36.8631 149.5949	**Existing Plot Custodian**	NA
**Tenure**	South East Forests NP	**Existing Plot ID**	NA
**Plot Est. Date**	20 May 2014	**Existing Plot Area**	NA
**Plot Size**	1.0ha (100mx100m)	**Existing Plot Census**	NA

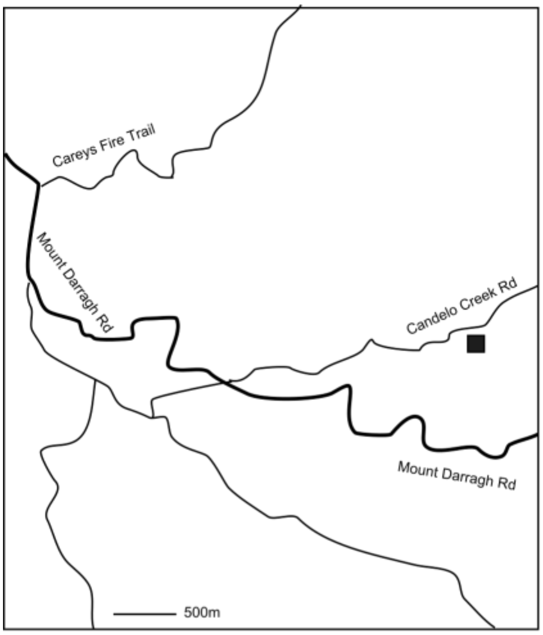

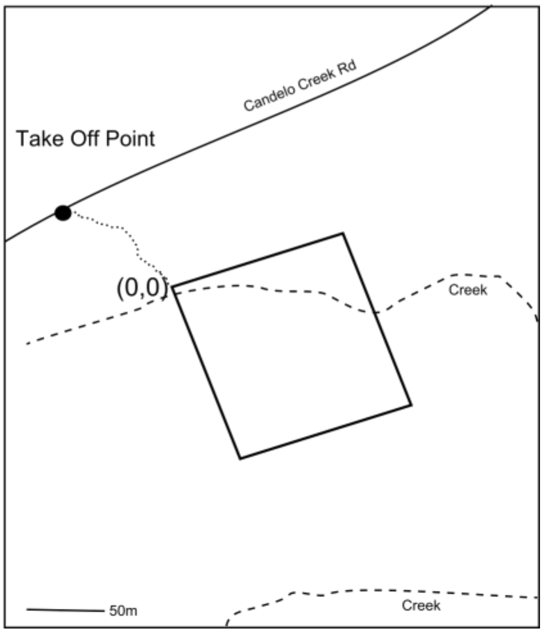

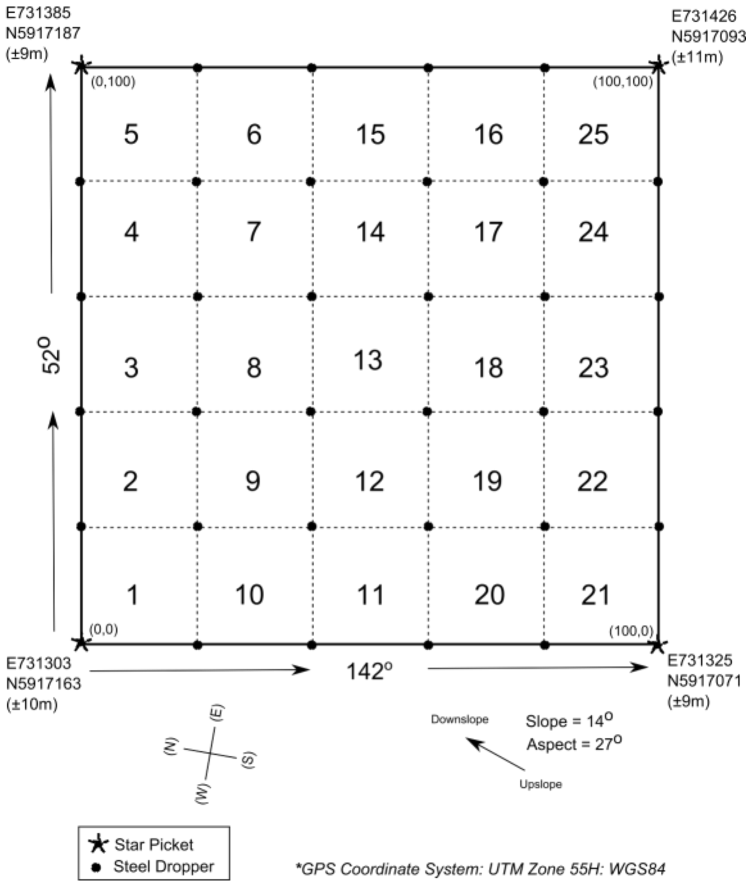

NSFSEC005: Candelo

**Target Eucalypt Species:**	*Eucalyptus obliqua*	**High severity fire?**	Unknown
**Maximum Tree Height (m)**	57m	**Low severity fire?**	Yes, frequency unknown (Fire Scars)
**Target Species Growth Stage:**	Mature	**Cut stumps?**	No
**Understorey:**	Wet Sclerophyll	**Other Disturbance?**	No

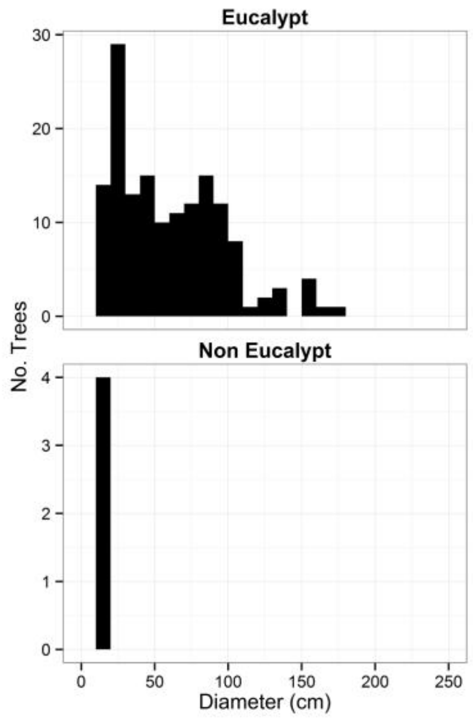

Species	No. Stems	BA (m^2/ha)
Eucalyptus obliqua	122	47.5
Eucalyptus cypellocarpa	21	8.8
Eucalyptus fastigata	5	1.2
Eucalyptus sieberi	3	0.1
Acacia melanoxylon	2	<0.1
Pittosporum undulatum	1	<0.1
Bedfordia arborescens	1	<0.1

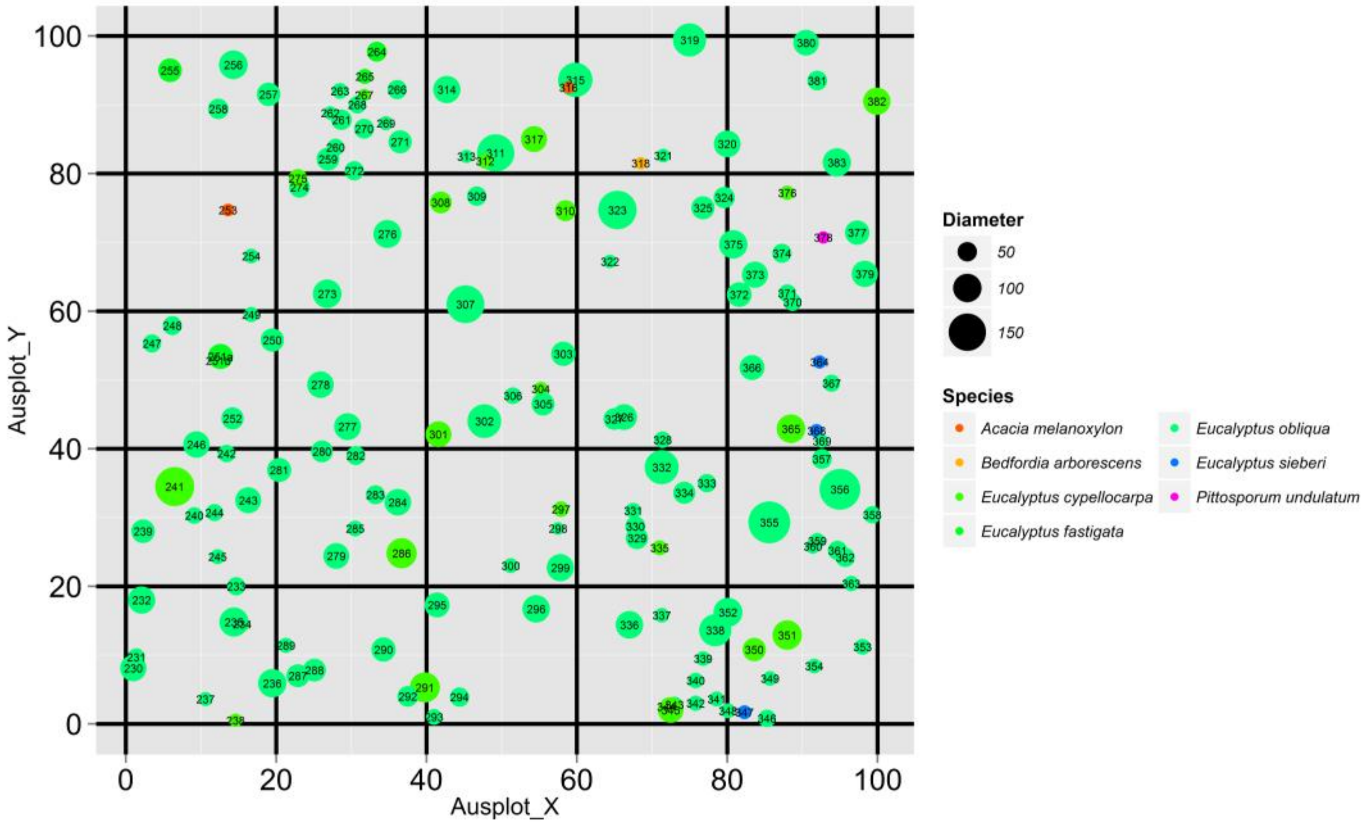

Northern

New South Wales (NSF)

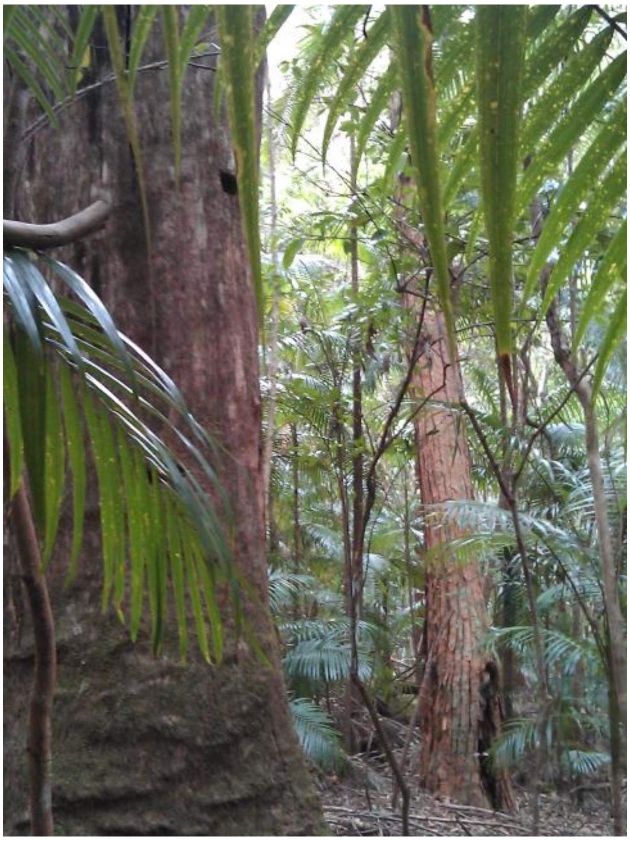

Eucalyptus pilularis at NSFNNC005 (Port Macquarie)

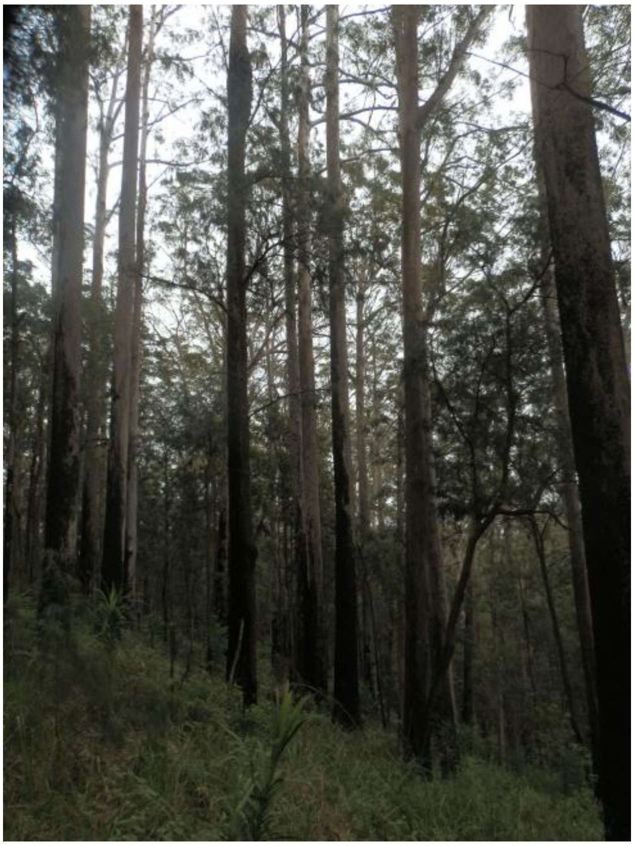

Eucalyptus pilularis at NSFNNC002 (A-Tree)

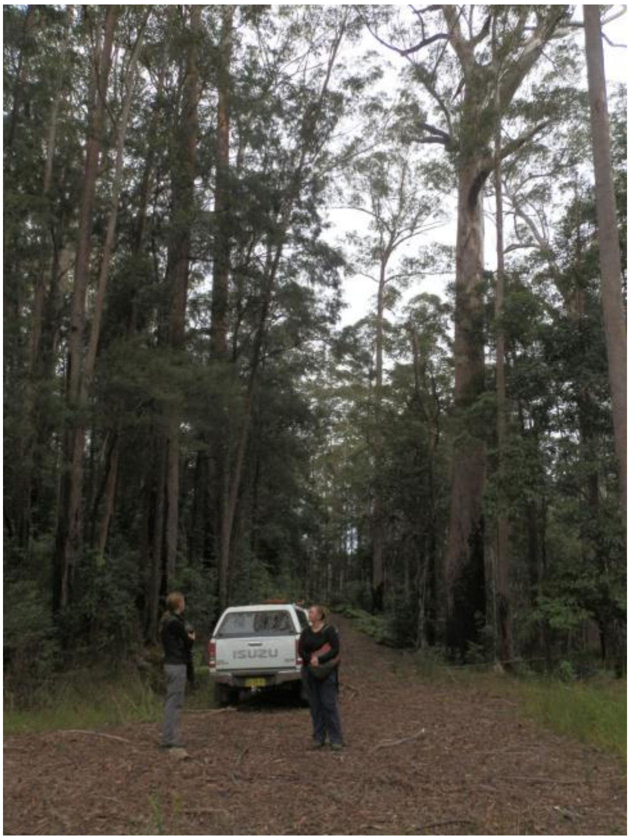

Eucalyptus pilularis at NSFNNC001 (Mines Road)

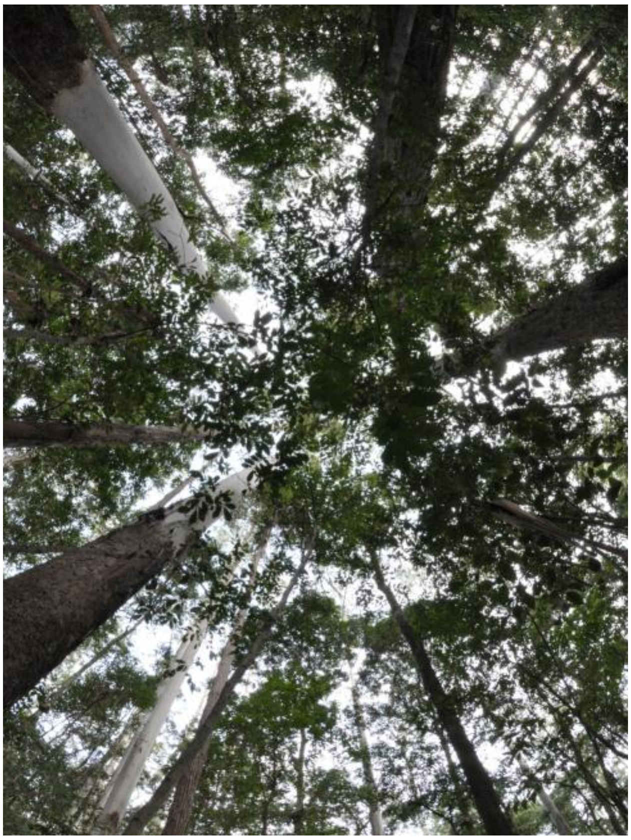

Eucalyptus grandis at NSFNNC008 (O'Sullivans *)*

Northern
New South Wales (NSF)

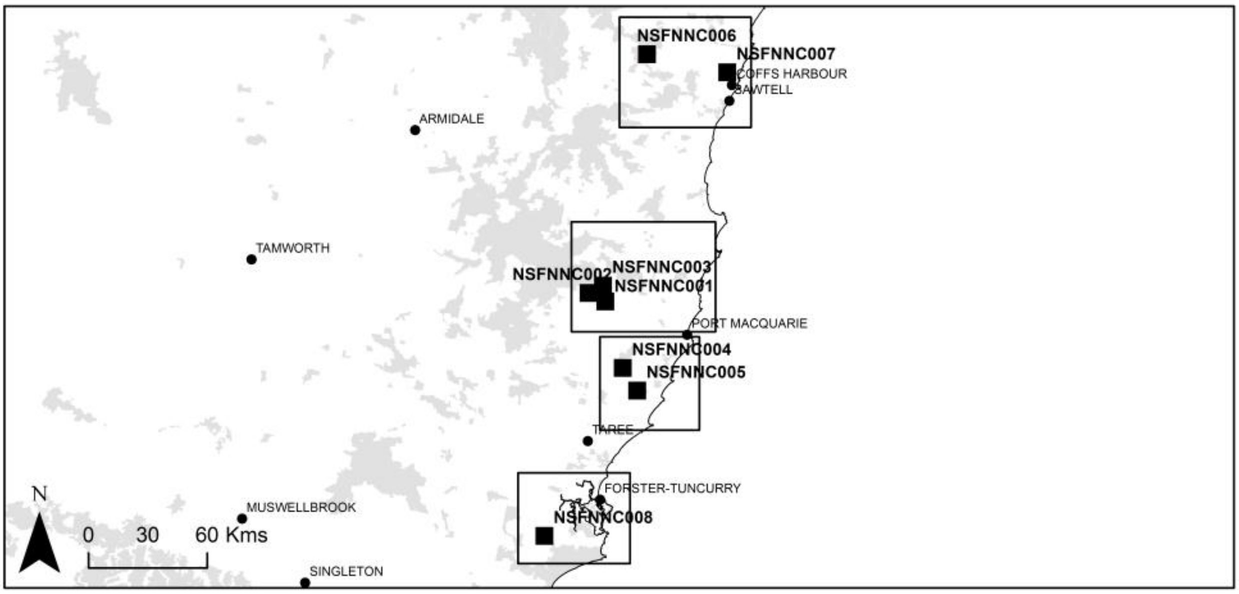

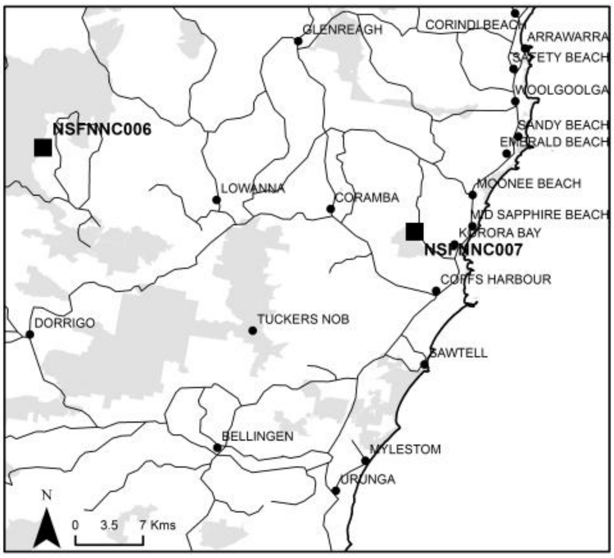

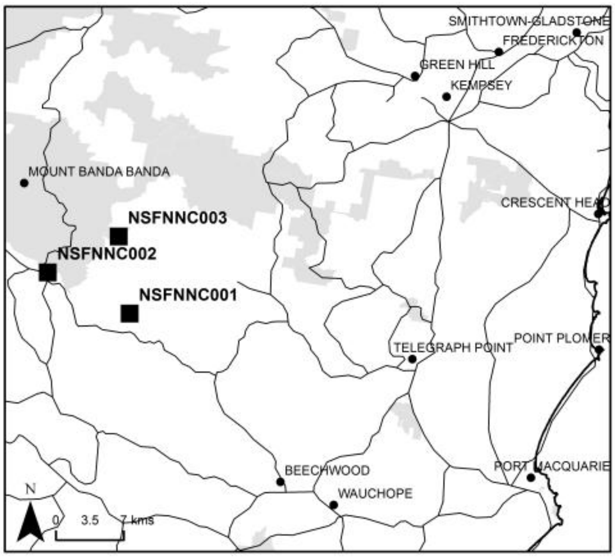

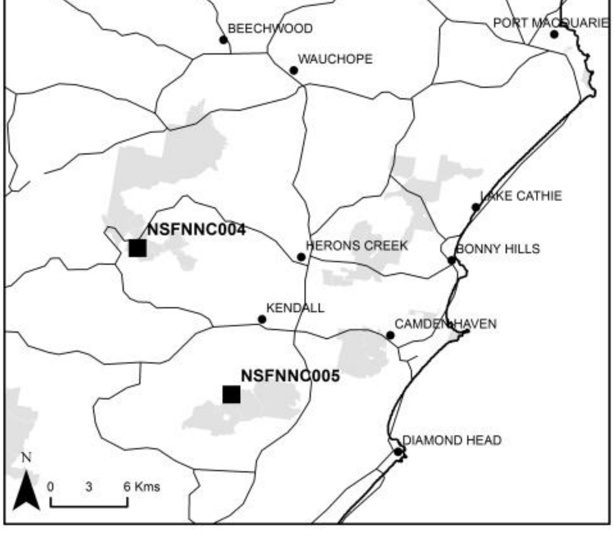

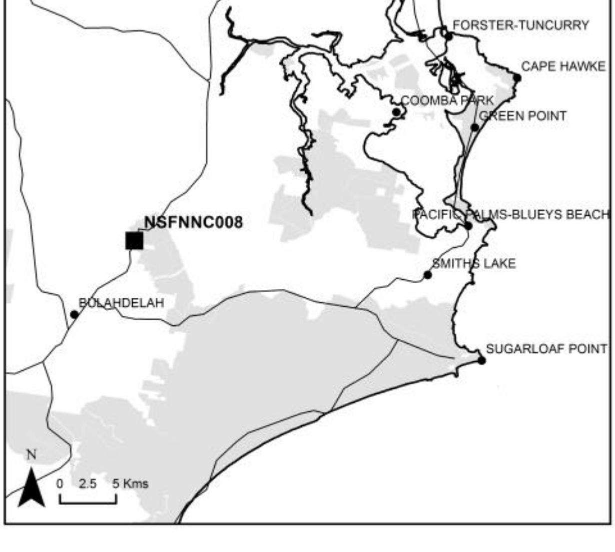

NSFNNC001: Mines Road

**AusPlot ID**	NSFNNC001	**Elevation**	535m
**AusPlot Name**	Mines Road	**Aspect**	335°
**State**	New South Wales	**Slope**	14°; Moderate to Steep
**Bioregion**	NSW North Coast	**Landform Element**	Ridge
**Location (UTM)**	56 J 455876 6539200	**MAT, MAP**	15.4 ^{o}C, 1434 mm
**Location (Lat/Long)**	-31.2803 152.5368	**Existing Plot Custodian**	ForestsNSW
**Tenure**	Mines Rd Flora Reserve	**Existing Plot ID**	Mines Rd Flora Reserve
**Plot Est. Date**	20 September 2013	**Existing Plot Area**	NA
**Plot Size**	1.0ha (100mx100m)	**Existing Plot Census**	Flora Surveys

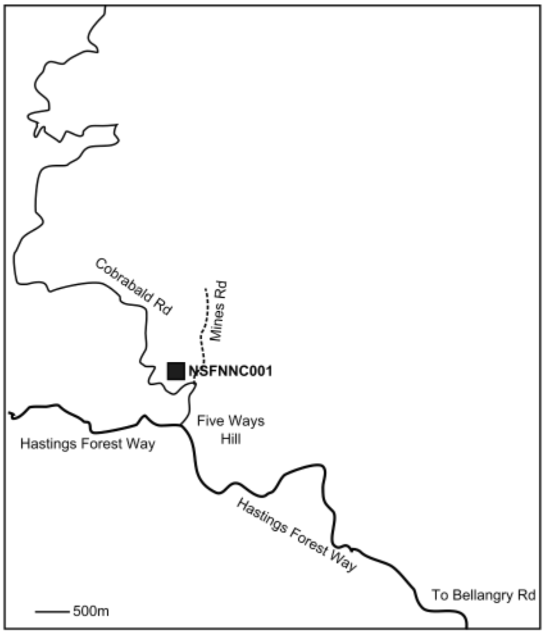

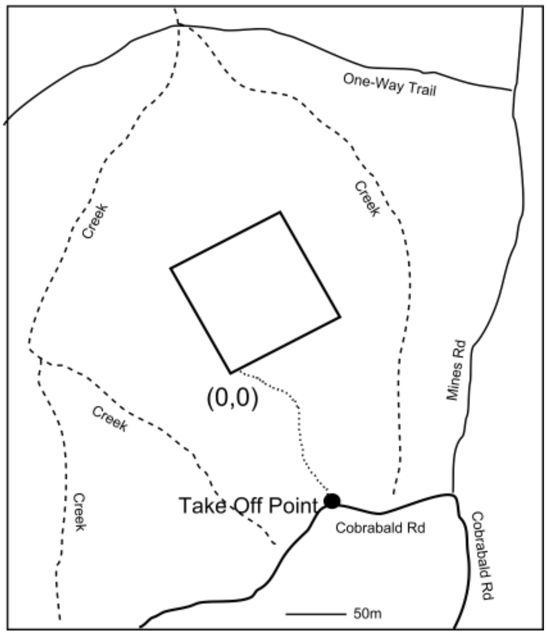

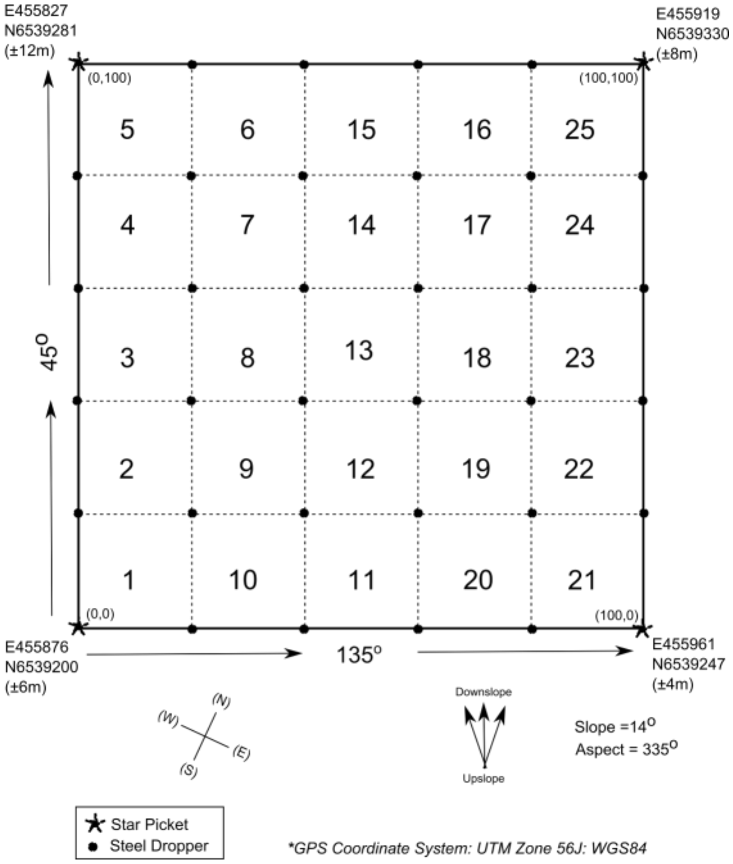

NSFNNC001: Mines Road

**Target Eucalypt Species:**	*Eucalyptus pilularis*	**High severity fire?**	Unknown
**Maximum Tree Height (m)**	56m	**Low severity fire?**	Yes, frequency unknown (Fire Scars)
			Most recent fire in last 2-4 years
**Target Species Growth Stage:**	Mature	**Cut stumps?**	No
**Understorey:**	Wet Sclerophyll	**Other Disturbance?**	No

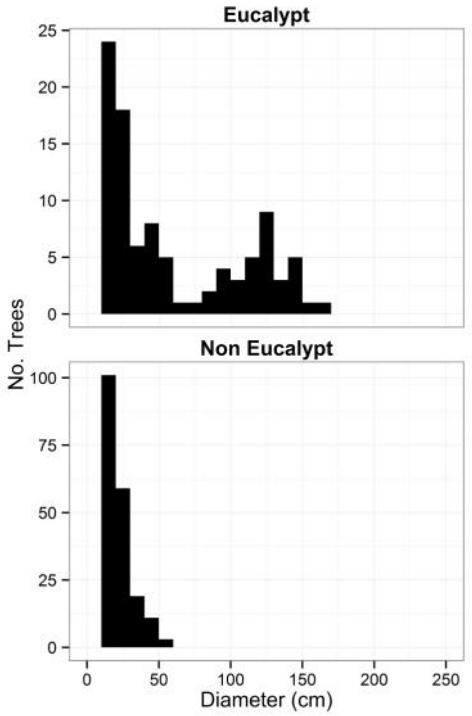

Species	No. Stems	BA (m^2/ha)
Eucalyptus pilularis	78	41.8
Allocasuarina torulosa	152	7.8
Eucalyptus microcorys	20	2.6
Synoum glandulosum	55	1.0
Cryptocarya rigida	38	0.5
Schizomeria ovata	19	0.4
Elaeocarpus reticulatus	16	0.2
Archontophoenix cunninghamiana	5	0.1
Trochocarpa laurina	11	0.1
Endiandra sieberi	8	0.1
Archihodomyrtus beckleri	9	0.1
Caldcluvia paniculosa	3	0.1
Rhodamnia rubescens	4	0.1
Eupomatia laurina	4	0.1
Eucalyptus sp.	2	<0.1
UNNCUnk	2	<0.1

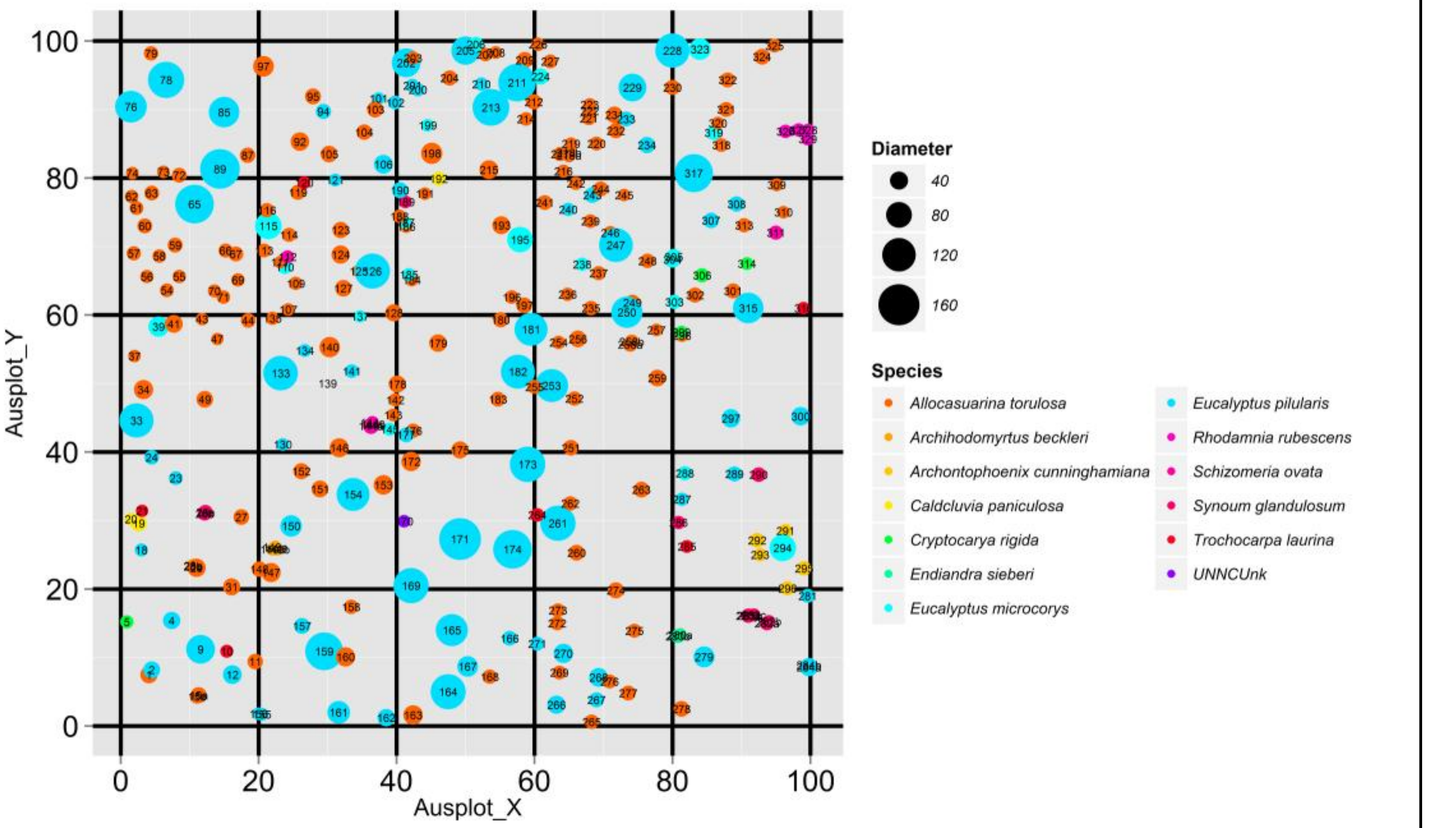

NSFNNC002: A-Tree

**AusPlot ID**	NSFNNC002	**Elevation**	631m
**AusPlot Name**	A-Tree	**Aspect**	10°
**State**	New South Wales	**Slope**	$15\text{-}30^{o}$; Moderate to Steep
**Bioregion**	NSW North Coast	**Landform Element**	Ridge and Upper Slope
**Location (UTM)**	56 J 448656 6543494	**MAT, MAP**	15.3 ^{o}C, 1375 mm
**Location (Lat/Long)**	-31.2421 152.4609	**Existing Plot Custodian**	ForestsNSW
**Tenure**	Willi Willi NP	**Existing Plot ID**	A-Tree Flora Reserve
**Plot Est. Date**	26 September 2013	**Existing Plot Area**	NA
**Plot Size**	1.0ha (100mx100m)	**Existing Plot Census**	Flora Surveys and Tree Inventory

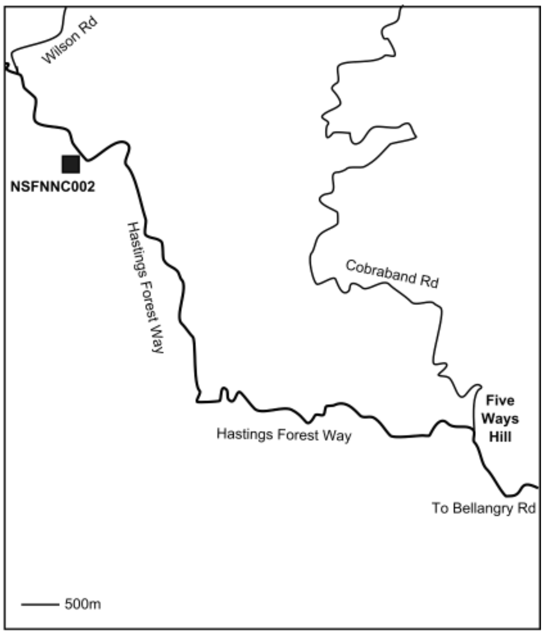

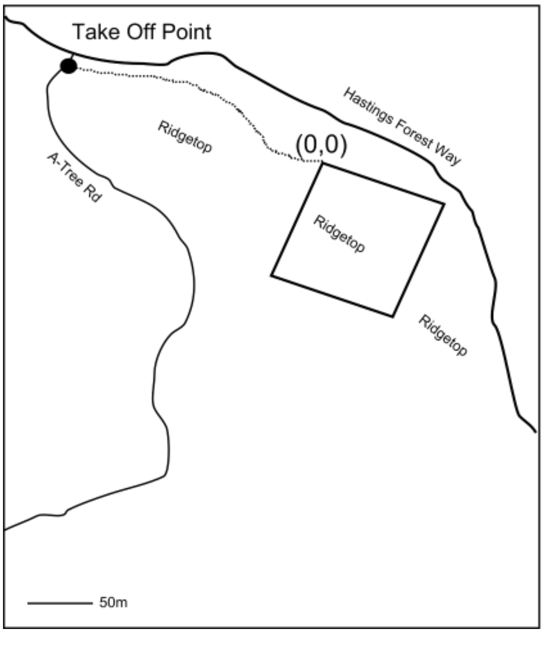

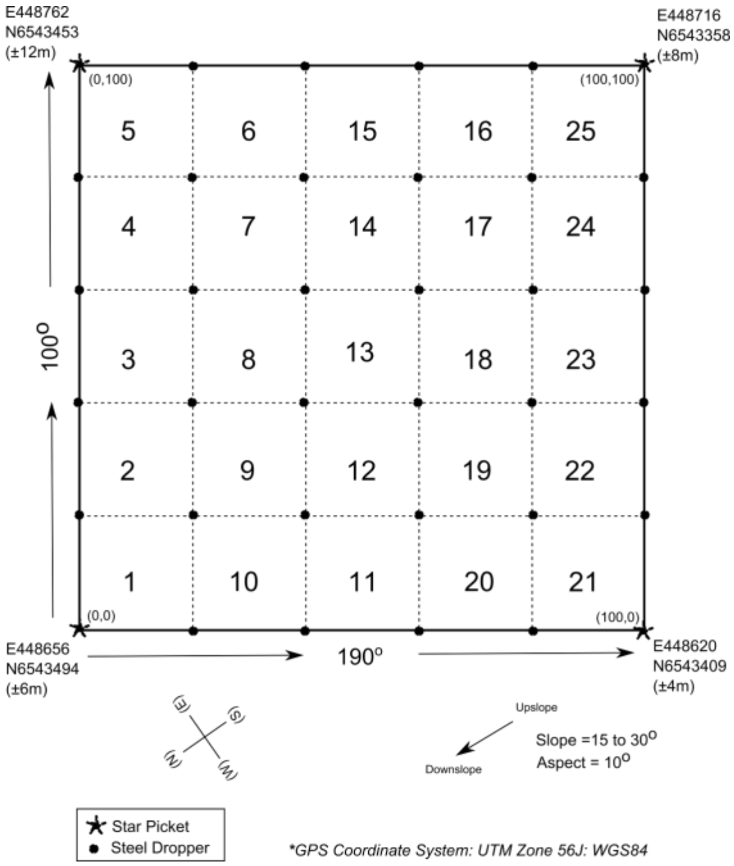

NSFNNC002: A-Tree

**Target Eucalypt Species:**	*Eucalyptus pilularis*	**High severity fire?**	Unknown
**Maximum Tree Height (m)**	66m	**Low severity fire?**	Yes, frequency unknown (Fire Scars)
**Target Species Growth Stage:**	Mature	**Cut stumps?**	Yes, 5 chainsawed stumps
**Understorey:**	Wet Sclerophyll	**Other Disturbance?**	No

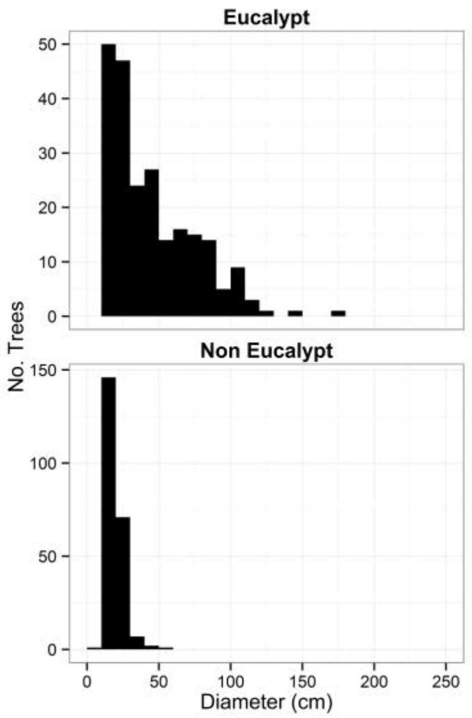

Species	No. Stems	BA (m^2/ha)
Eucalyptus pilularis	165	45.1
Allocasuarina torulosa	226	6.8
Eucalyptus microcorys	52	4.6
Eucalyptus saligna	9	2.1
Lophostemon sp.	1	0.1
UNNCU14	1	<0.1
UNNCU15	1	<0.1
Eucalyptus sp.	1	<0.1
Trochocarpa laurina	1	<0.1
Elaeocarpus reticulatus	1	<0.1

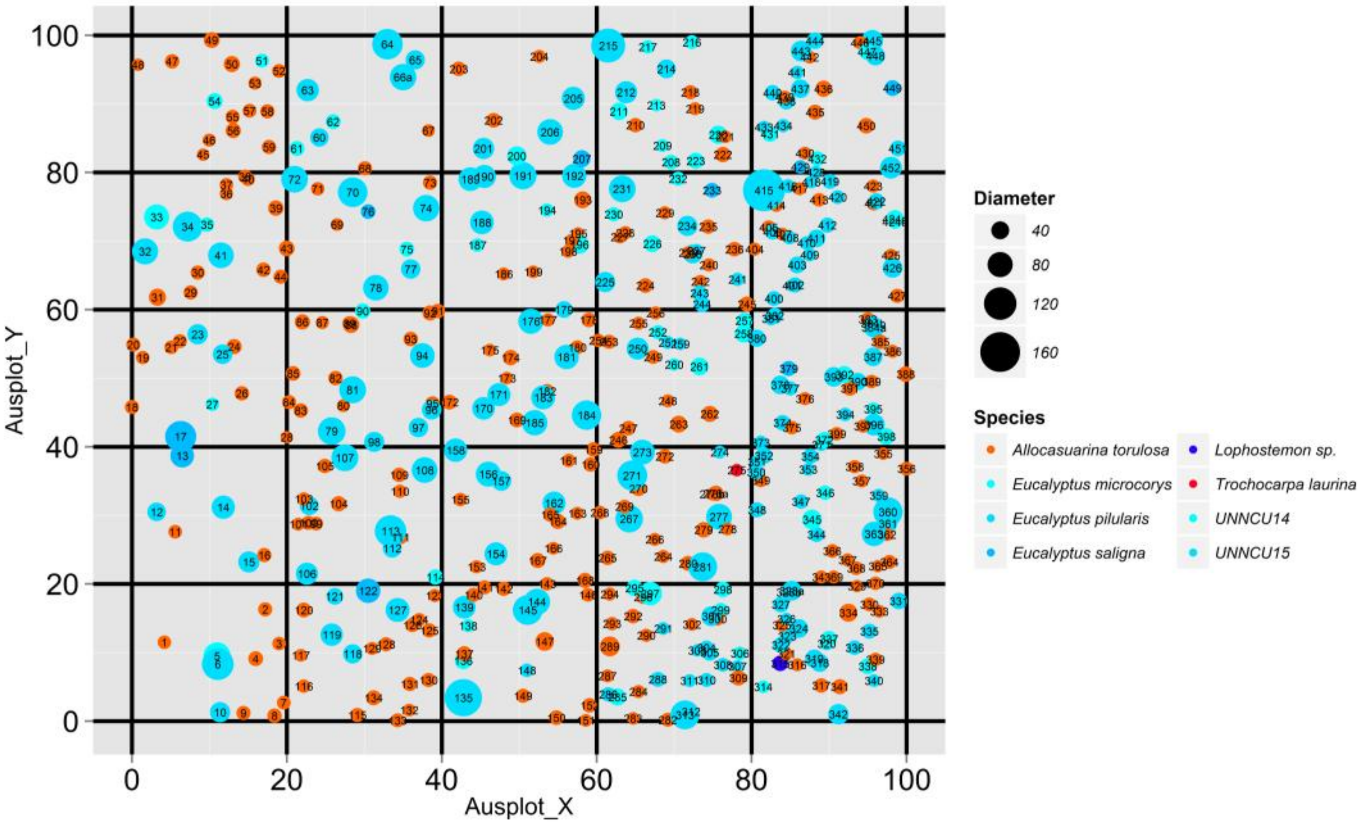

NSFNNC003: Tinebank

**AusPlot ID**	NSFNNC003	**Elevation**	600m
**AusPlot Name**	Tinebank	**Aspect**	147°
**State**	New South Wales	**Slope**	Not Recorded; Moderate to Steep
**Bioregion**	NSW North Coast	**Landform Element**	Midslope
**Location (UTM)**	56 J 454822 6547263	**MAT, MAP**	16.2 ^oC, 1362 mm
**Location (Lat/Long)**	-31.2086 152.5267	**Existing Plot Custodian**	ForestsNSW
**Tenure**	Willi Will NP	**Existing Plot ID**	Tinebank Flora Reserve
**Plot Est. Date**	01 October 2013	**Existing Plot Area**	NA
**Plot Size**	1.0ha (100mx100m)	**Existing Plot Census**	Flora Surveys

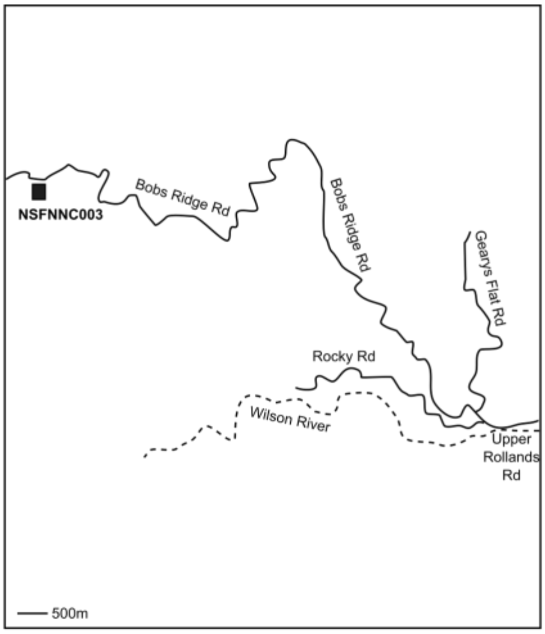

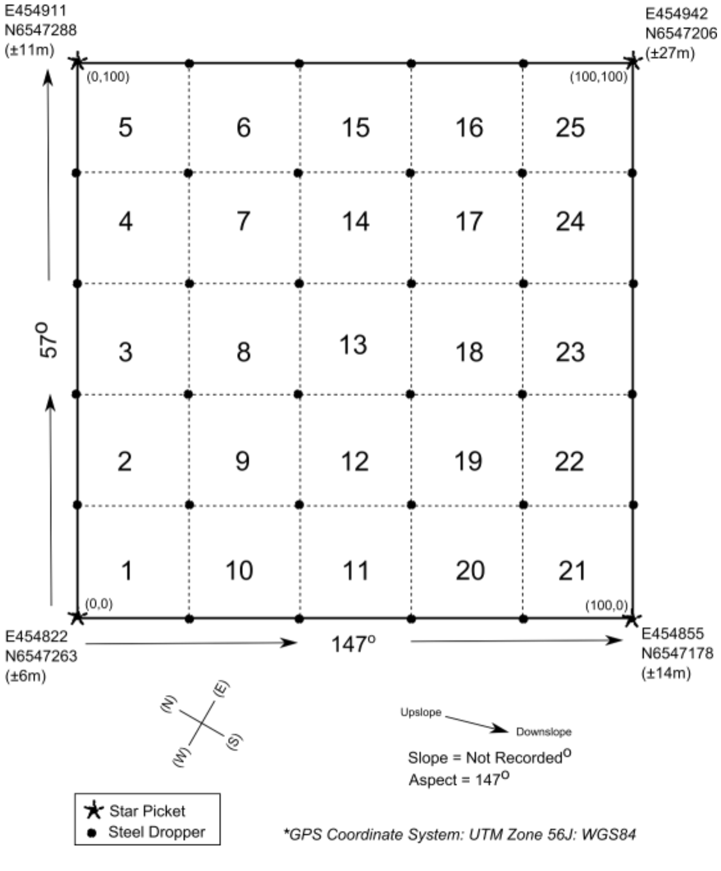

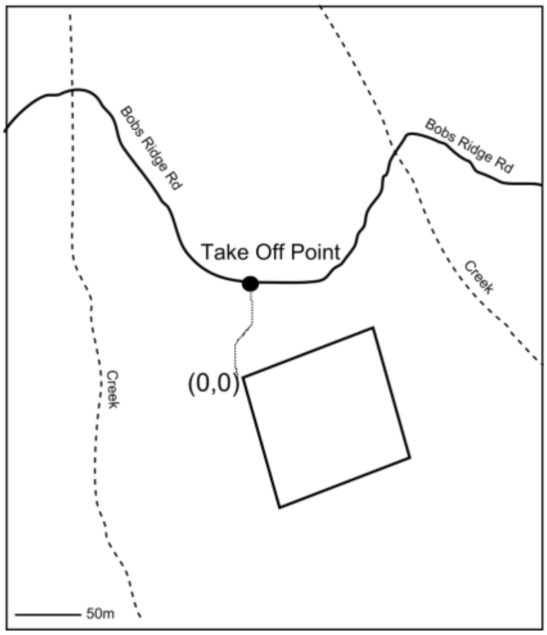

NSFNNC003: Tinebank

Target Eucalypt Species: *Eucalyptus pilularis*

Maximum Tree Height (m) 74m

Target Species Growth Stage: Mature

Understorey: Wet Sclerophyll

High severity fire? Unknown

Low severity fire? No

Cut stumps? No

Other Disturbance? No

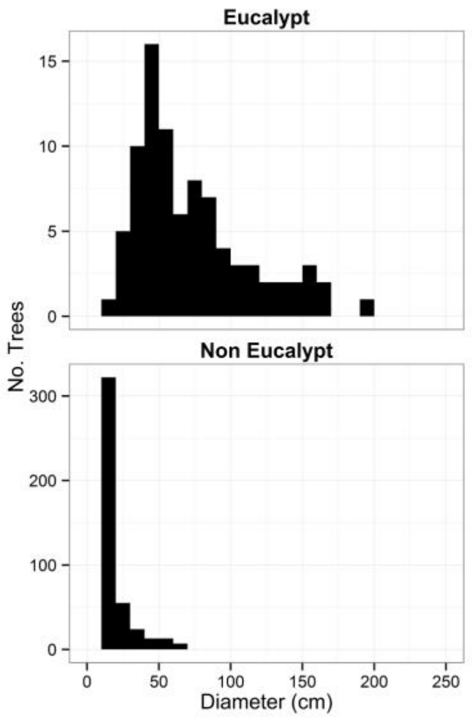

Species	No. Stems	BA (m^2/ha)
Eucalyptus pilularis	27	31.3
Syncarpia glomulifera	59	14.8
Allocasuarina torulosa	43	7.9
Cryptocarya rigida	209	3.1
UNNCU22	39	1.6
UNNCU19	45	1.3
UNNCU18	39	0.8
Acacia schinoides	13	0.7
Synoum glandulosum	27	0.5
UNNCU21	5	0.4
Melicope hayesii	7	0.1
Caldcluvia paniculosa	2	0.1
Callicoma serratifolia	4	0.1
UNNCU23	1	<0.1

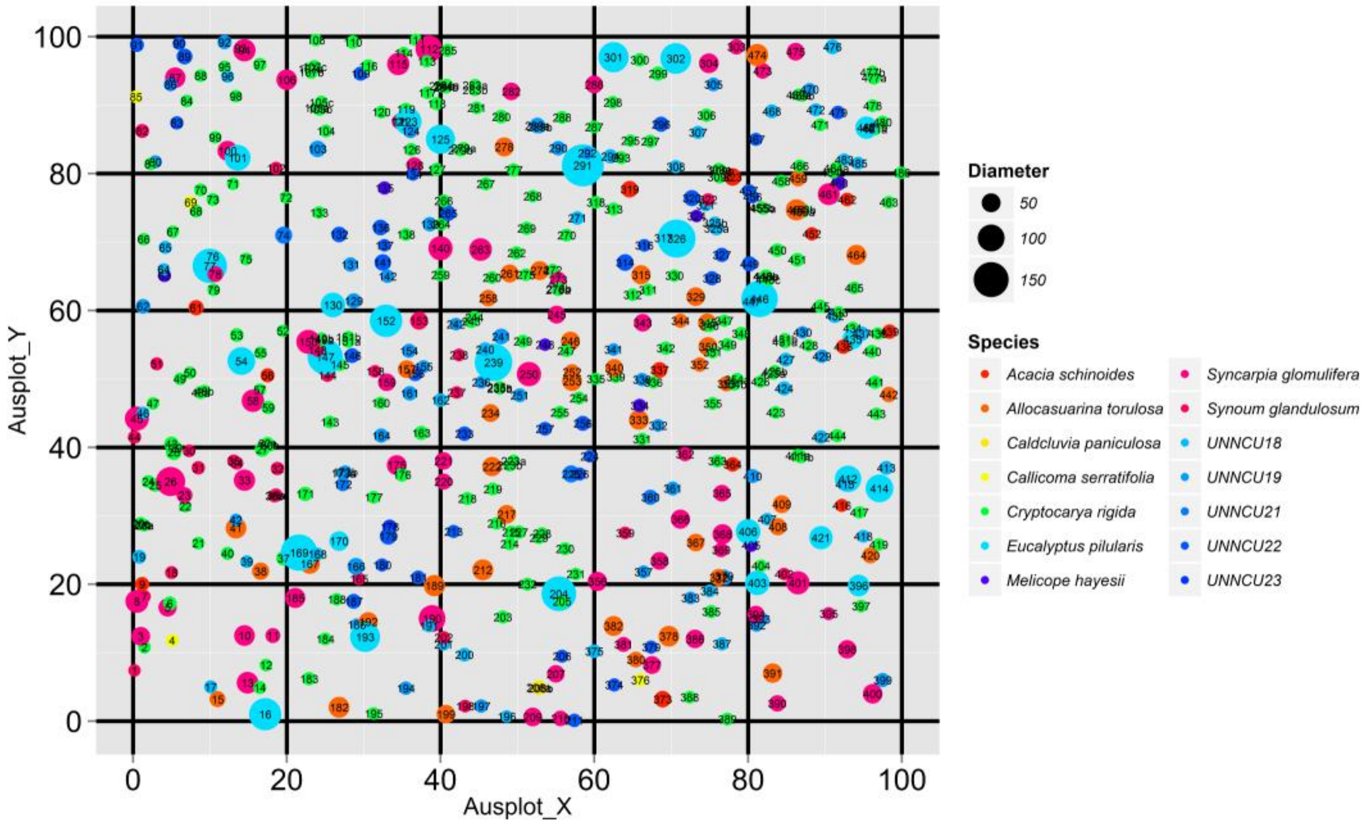

NSFNNC004: Lorne

**AusPlot ID**	NSFNNC004	**Elevation**	283m
**AusPlot Name**	Lorne	**Aspect**	160°
**State**	New South Wales	**Slope**	8°; Gently Inclined
**Bioregion**	NSW North Coast	**Landform Element**	Midslope
**Location (UTM)**	56 J 463887 6504736	**MAT, MAP**	16.5 ^{o}C, 1457 mm
**Location (Lat/Long)**	-31.5828 152.6164	**Existing Plot Custodian**	ForestsNSW
**Tenure**	Bago Bluff NP	**Existing Plot ID**	Lorne Flora Reserve
**Plot Est. Date**	06 October 2013	**Existing Plot Area**	NA
**Plot Size**	1.0ha (100mx100m)	**Existing Plot Census**	Flora Surveys

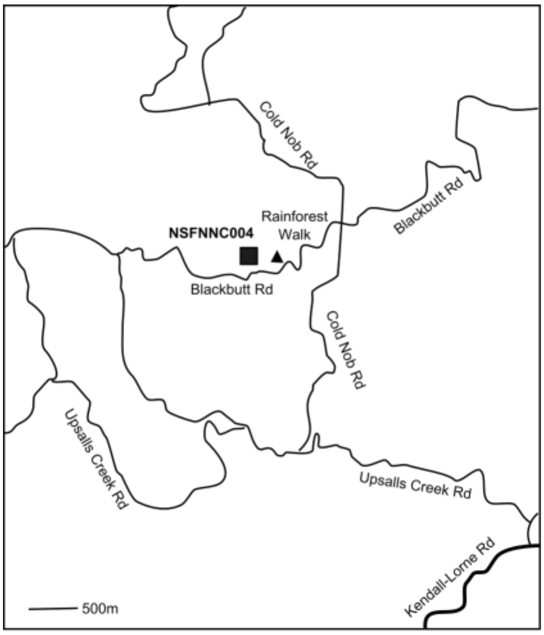

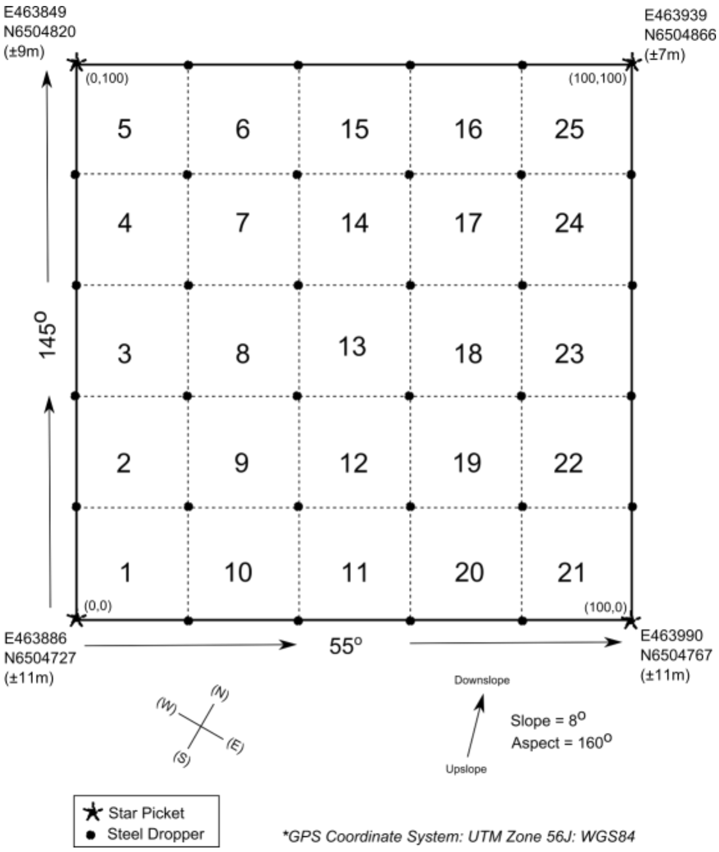

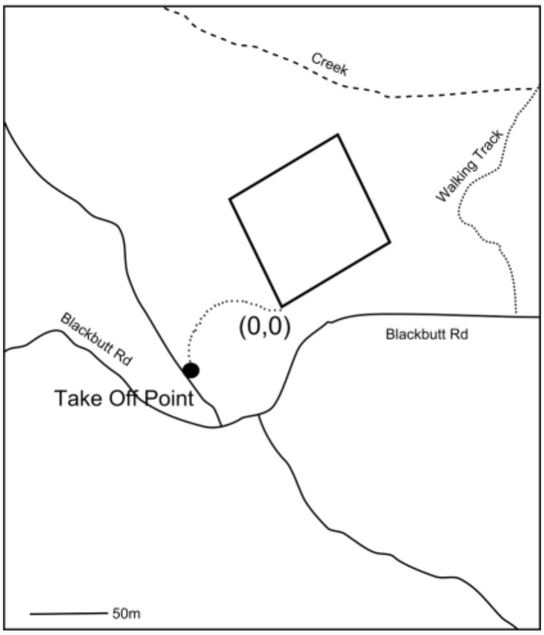

NSFNNC004: Lorne

**Target Eucalypt Species:**	*Eucalyptus pilularis*	**High severity fire?**	Unknown
**Maximum Tree Height (m)**	74m	**Low severity fire?**	Yes, frequency unknown (Fire Scars)
**Target Species Growth Stage:**	Mature	**Cut stumps?**	Yes, 6 chainsaw, 7 axe
**Understorey:**	Wet Sclerophyll	**Other Disturbance?**	No

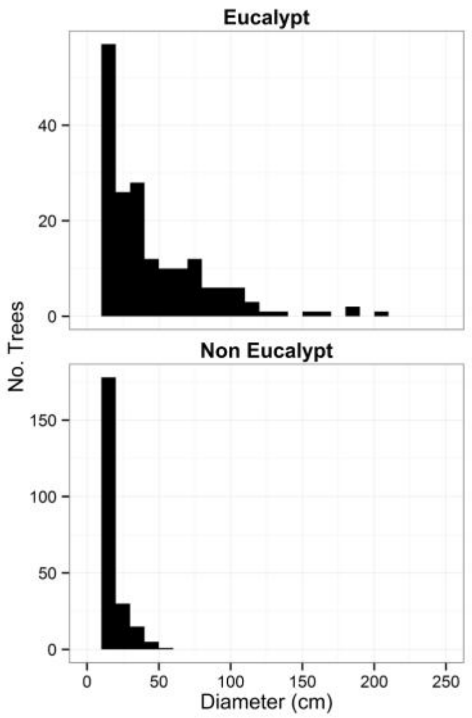

Species	No. Stems	BA (m^2/ha)
Eucalyptus pilularis	47	30.0
Eucalyptus microcorys	125	17.1
Allocasuarina torulosa	97	4.6
Syncarpia glomulifera	2	1.0
Eucalyptus saligna	10	0.7
Cryptocarya glaucescens	48	0.7
Cryptocarya rigida	51	0.6
Archontophoenix cunninghamiana	6	0.1
Synoum glandulosum	6	0.1
Schizomeria ovata	3	0.1
Cryptocarya microneura	3	<0.1
Acmena smithii	1	<0.1
UNNCU18	3	<0.1
Caldcluvia paniculosa	1	<0.1
Trochocarpa laurina	3	<0.1
Pilidiostigma glabrum	2	<0.1
UNNCU29	1	<0.1
Endiandra discolor	1	<0.1
UNNCU26	1	<0.1
Callistemon salignus	1	<0.1
Pittosporum undulatum	1	<0.1

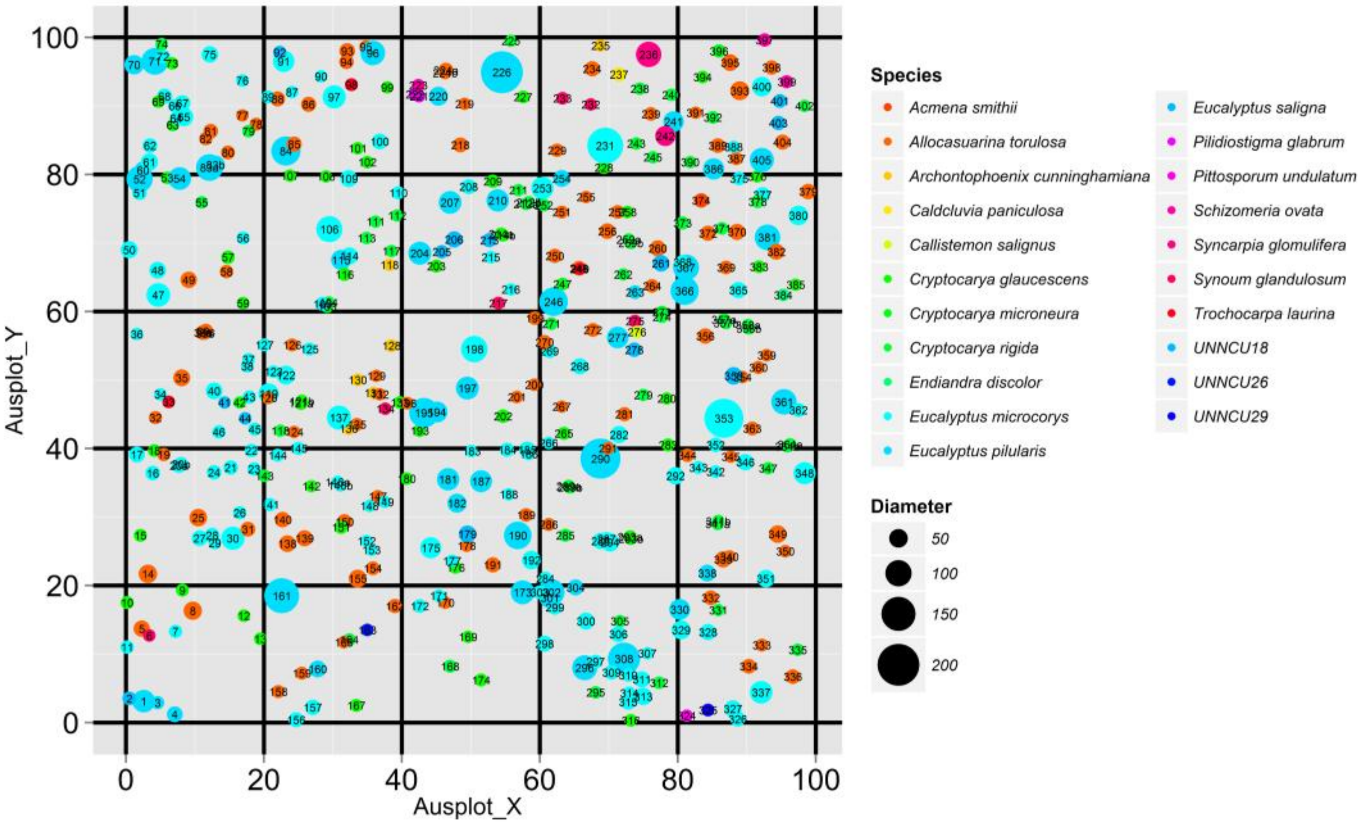

NSFNNC005: Bird Tree

**AusPlot ID**	NSFNNC005	**Elevation**	352m
**AusPlot Name**	Bird Tree	**Aspect**	350°
**State**	New South Wales	**Slope**	6°; Gently Inclined
**Bioregion**	NSW North Coast	**Landform Element**	Midslope
**Location (UTM)**	56 J 469954 6494251	**MAT, MAP**	16.1 ^oC, 1542 mm
**Location (Lat/Long)**	-31.6858 152.6825	**Existing Plot Custodian**	NA
**Tenure**	Middle Brother NP	**Existing Plot ID**	NA
**Plot Est. Date**	30 October 2013	**Existing Plot Area**	NA
**Plot Size**	1.0ha (100mx100m)	**Existing Plot Census**	NA

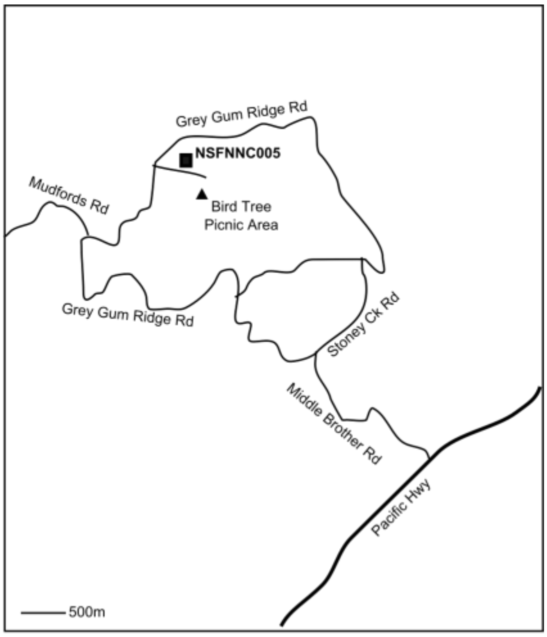

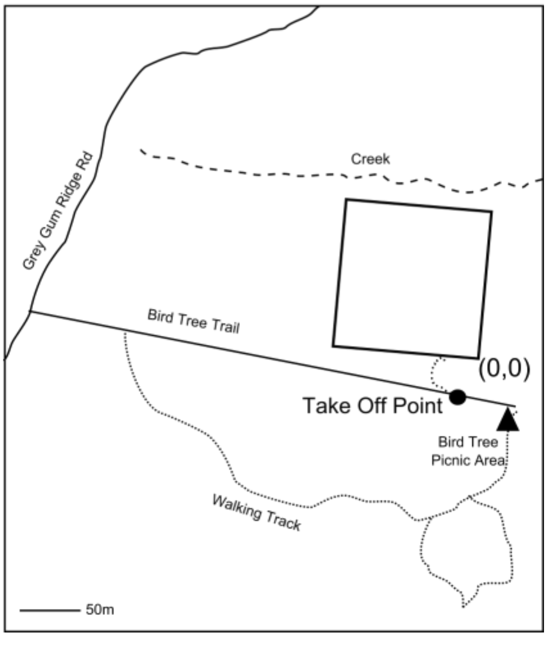

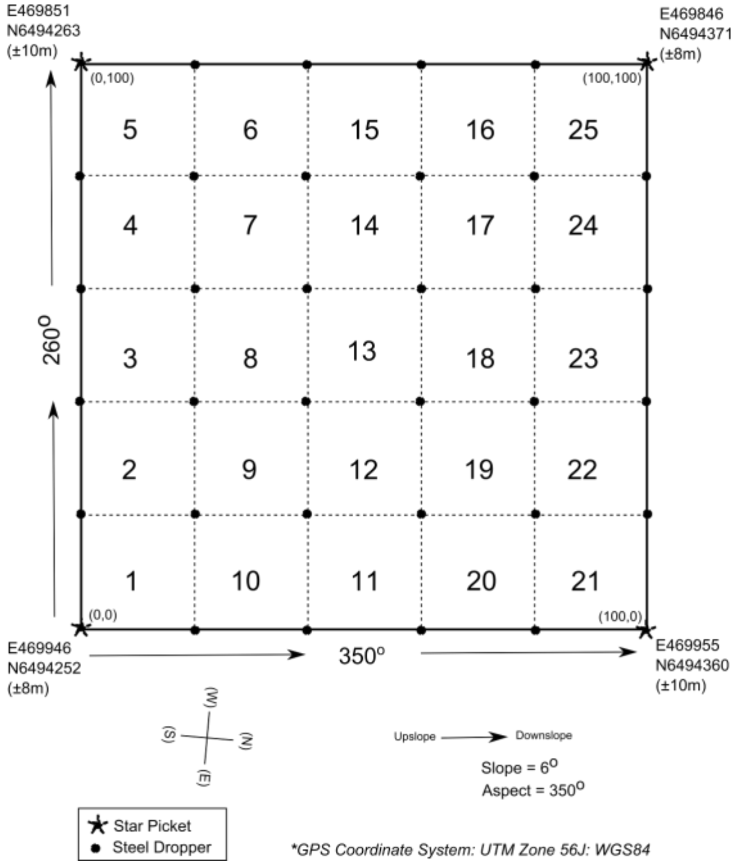

NSFNNC005: Bird Tree

**Target Eucalypt Species:**	*Eucalyptus pilularis*	**High severity fire?**	Unknown
**Maximum Tree Height (m)**	77m	**Low severity fire?**	Yes, frequency unknown (Fire Scars)
**Target Species Growth Stage:**	Mature	**Cut stumps?**	Yes, 18 axe stumps
**Understorey:**	Wet Sclerophyll/Rainforest	**Other Disturbance?**	No

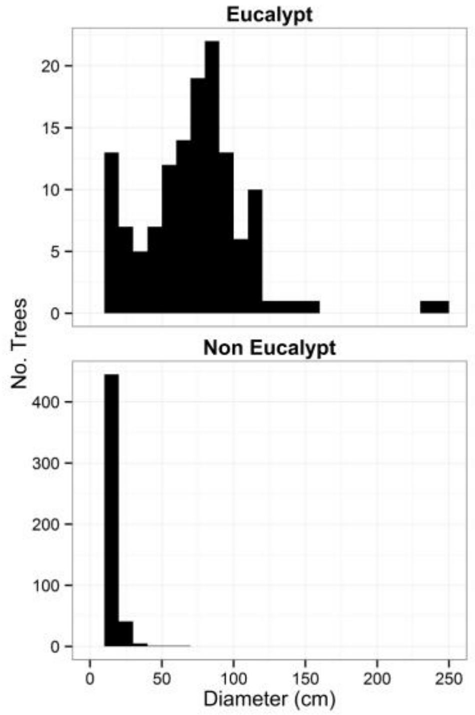

Species	No. Stems	BA (m^2/ha)
Eucalyptus pilularis	73	48.1
Eucalyptus microcorys	27	9.9
Syncarpia glomulifera	31	9.3
Schizomeria ovata	92	2.0
Allocasuarina torulosa	41	1.6
Archontophoenix cunninghamiana	116	1.4
Cryptocarya rigida	107	1.3
Synoum glandulosum	69	0.8
Lophostemon sp.	3	0.7
Cryptocarya microneura	12	0.6
Cryptocarya glaucescens	21	0.5
Caldcluvia paniculosa	4	0.4
Eupomatia laurina	7	0.1
UNNCU38	5	0.1
UNNCU40	4	0.1
Neolitsea dealbata	4	0.1
Cissus hypoglauca	3	<0.1
Archihodomyrtus beckleri	4	<0.1
Syzygium oleosum	3	<0.1
UNNCU34	1	<0.1
Clerodendrum floribundum	1	<0.1

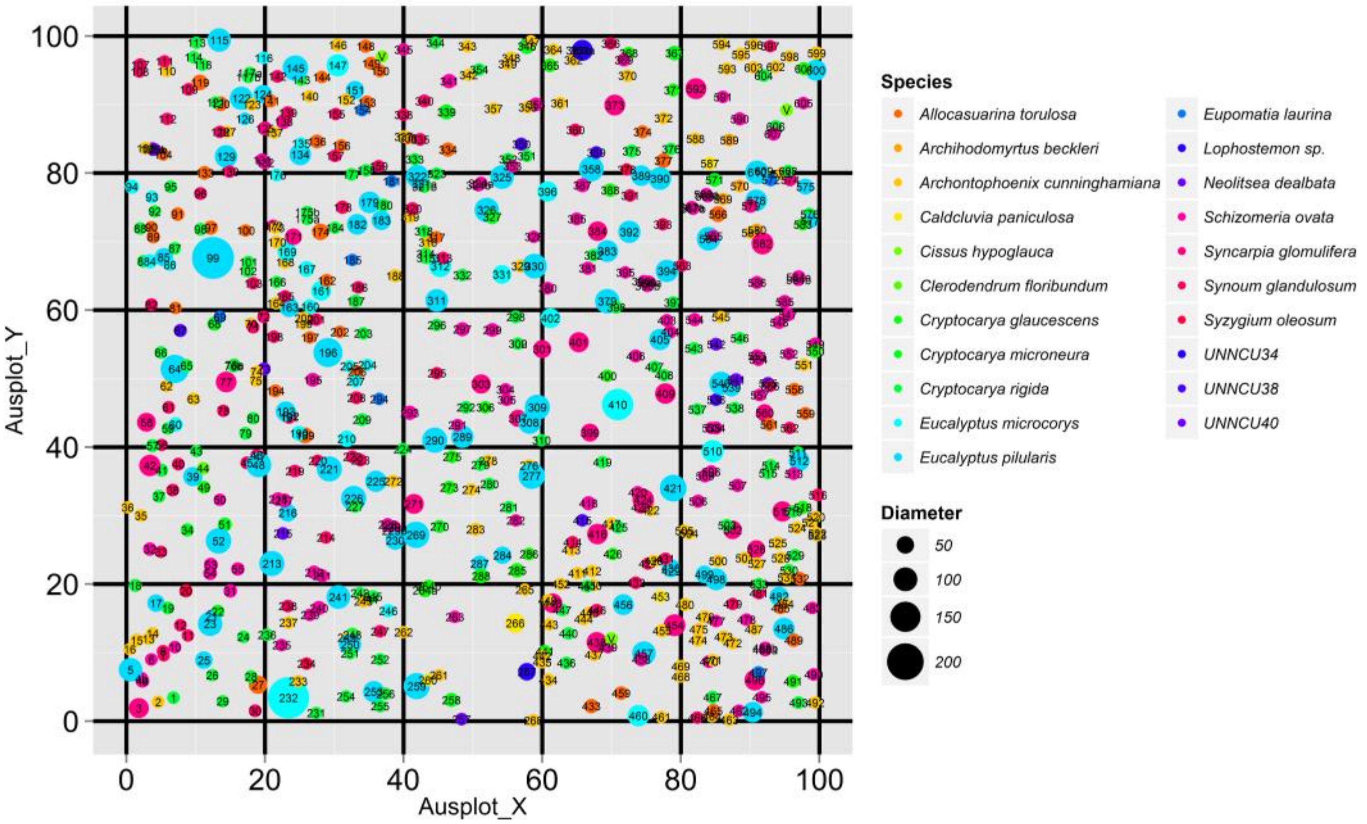

NSFNNC006: Black Bull

**AusPlot ID**	NSFNNC006	**Elevation**	683m
**AusPlot Name**	Black Bull	**Aspect**	180°
**State**	New South Wales	**Slope**	Not Recorded; Moderate
**Bioregion**	NSW North Coast	**Landform Element**	Midslope
**Location (UTM)**	56 J 473680 6663723	**MAT, MAP**	15.5 ^{o}C, 1895 mm
**Location (Lat/Long)**	-30.1578 152.7270	**Existing Plot Custodian**	Forests NSW
**Tenure**	Nymboi-Binderay NP	**Existing Plot ID**	Black Bull Flora Reserve
**Plot Est. Date**	07 November 2013	**Existing Plot Area**	NA
**Plot Size**	1.0ha (100mx100m)	**Existing Plot Census**	Flora Surveys

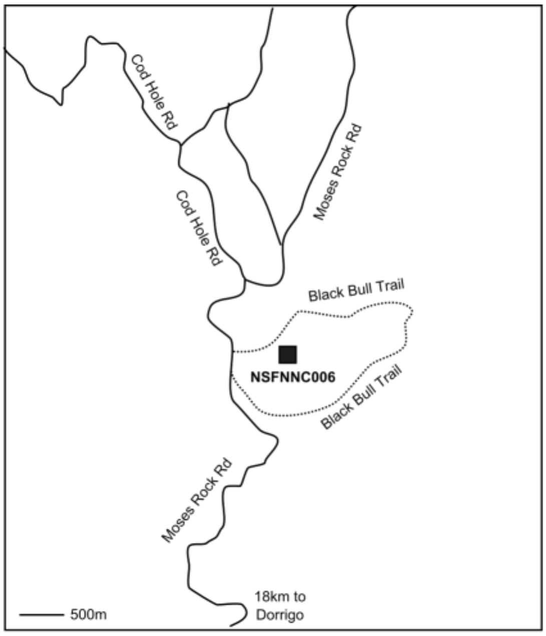

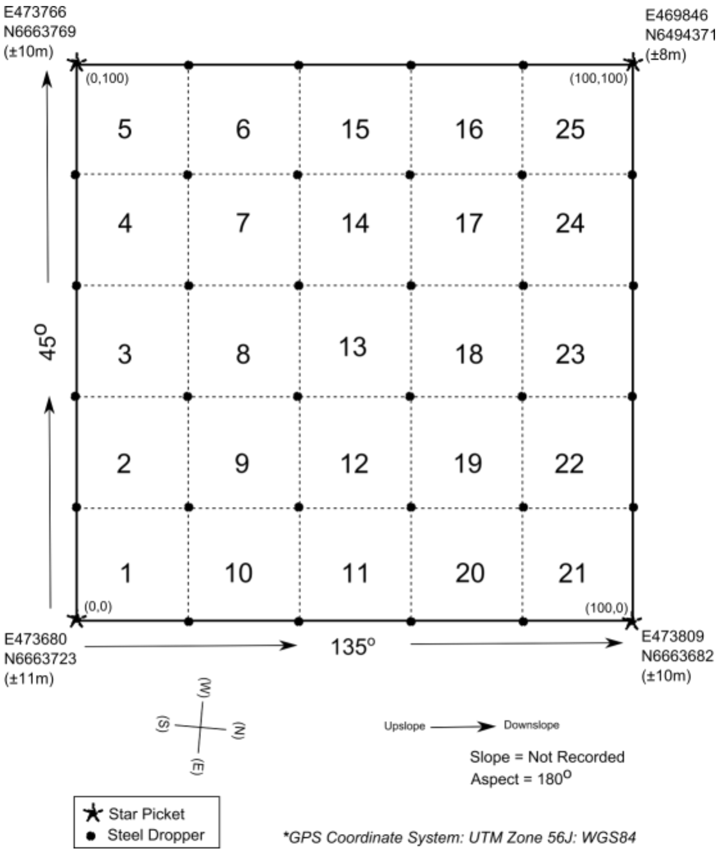

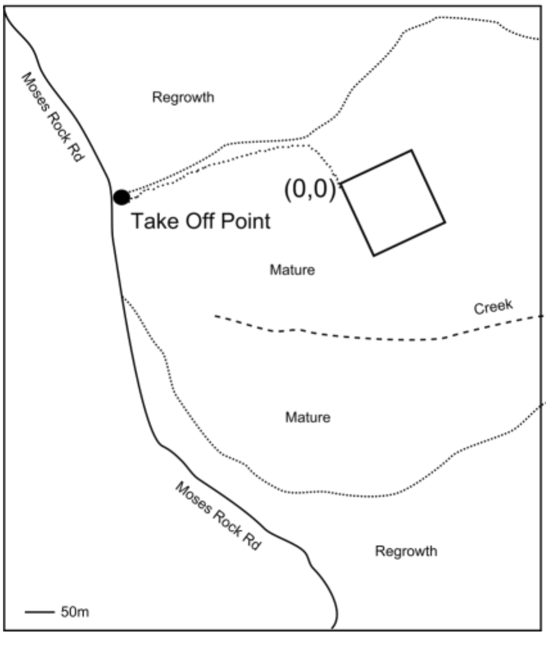

NSFNNC006: Black Bull

**Target Eucalypt Species:**	*Eucalyptus pilularis*	**High severity fire?**	Unknown
**Maximum Tree Height (m)**	70m	**Low severity fire?**	Yes, frequency unknown (Fire Scars)
**Target Species Growth Stage:**	Mature	**Cut stumps?**	No
**Understorey:**	Wet Sclerophyll	**Other Disturbance?**	No

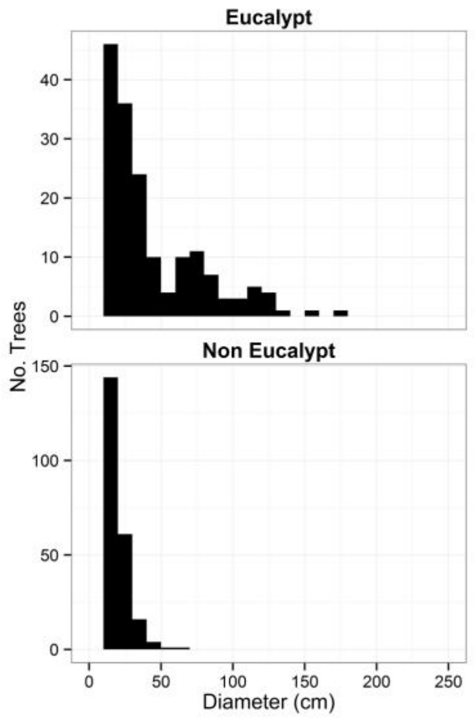

Species	No. Stems	BA (m^2/ha)
Eucalyptus pilularis	58	18.8
Eucalyptus andrewsii	17	12.0
Allocasuarina torulosa	185	7.2
Syncarpia glomulifera	79	5.0
Eucalyptus saligna	12	3.6
Trochocarpa laurina	24	0.3
UNNCU38	7	0.1
Acacia maidenii	5	0.1
Elaeocarpus reticulatus	2	<0.1
Persoonia conjuncta	1	<0.1
Callitris sp.	1	<0.1
Neolitsea dealbata	1	<0.1
Schizomeria ovata	1	<0.1

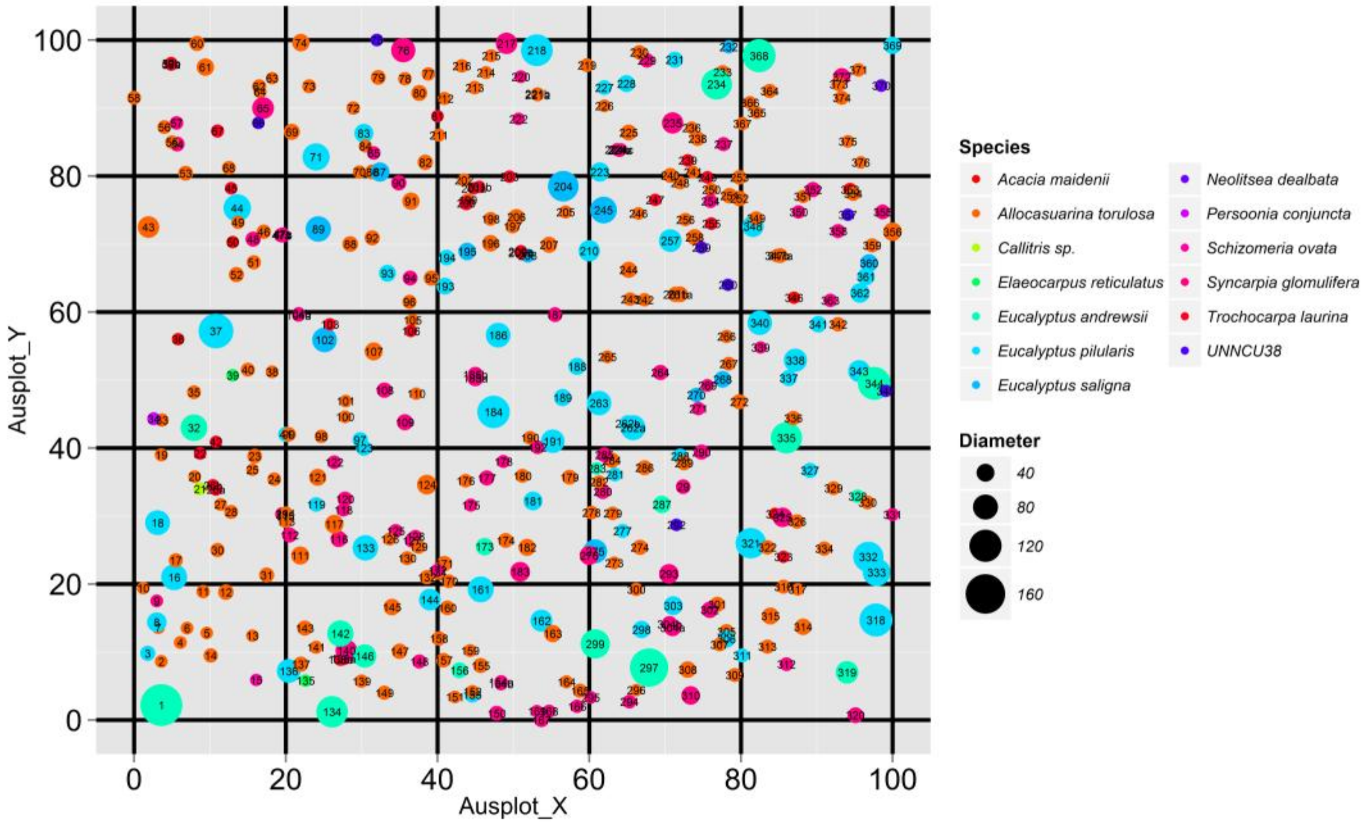

NSFNNC007: Bruxner

**AusPlot ID**	NSFNNC007	**Elevation**	188m
**AusPlot Name**	Bruxner	**Aspect**	270°
**State**	New South Wales	**Slope**	Not Recorded; Moderate
**Bioregion**	NSW North Coast	**Landform Element**	Midslope
**Location (UTM)**	56 J 508833 6654596	**MAT, MAP**	18.0 ^{o}C, 1872 mm
**Location (Lat/Long)**	-30.2401 153.0918	**Existing Plot Custodian**	Forests NSW
**Tenure**	Bruxner Flora Reserve	**Existing Plot ID**	Bruxner Flora Reserve
**Plot Est. Date**	13 November 2013	**Existing Plot Area**	NA
**Plot Size**	1.0ha (100mx100m)	**Existing Plot Census**	Flora Surveys

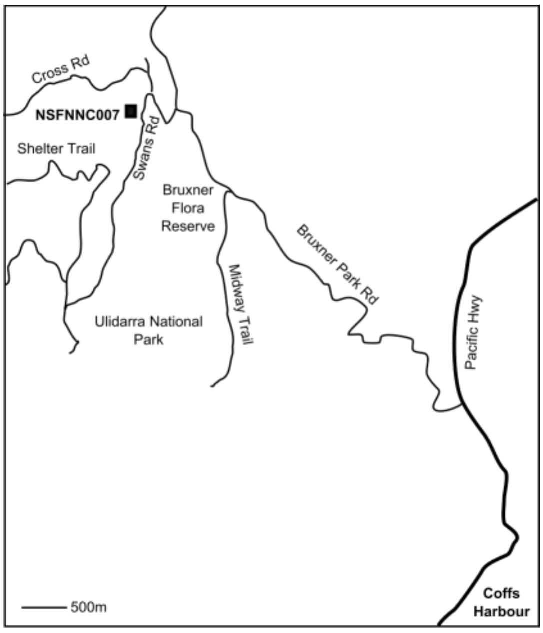

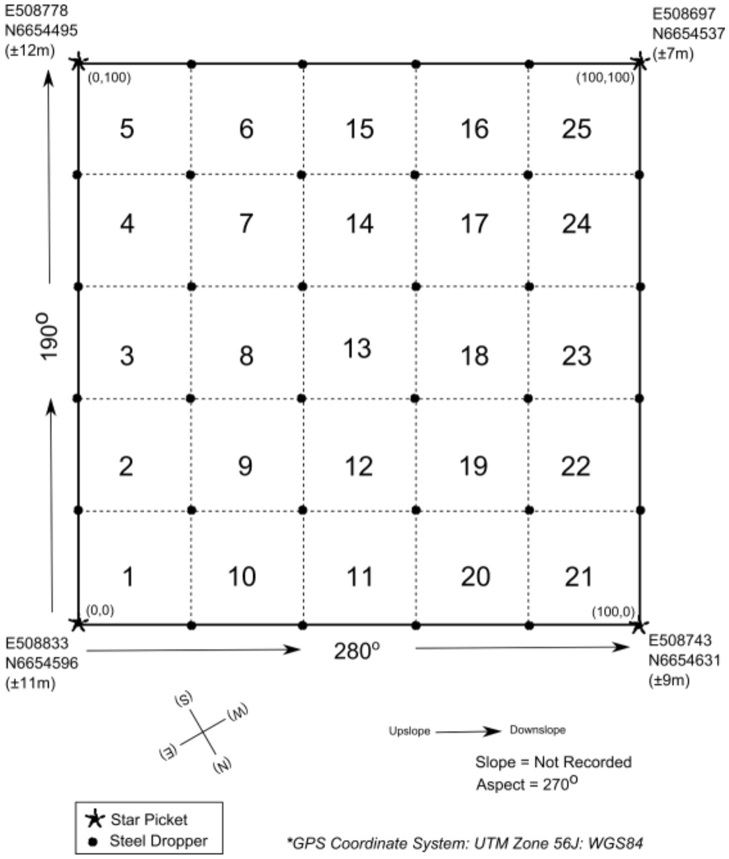

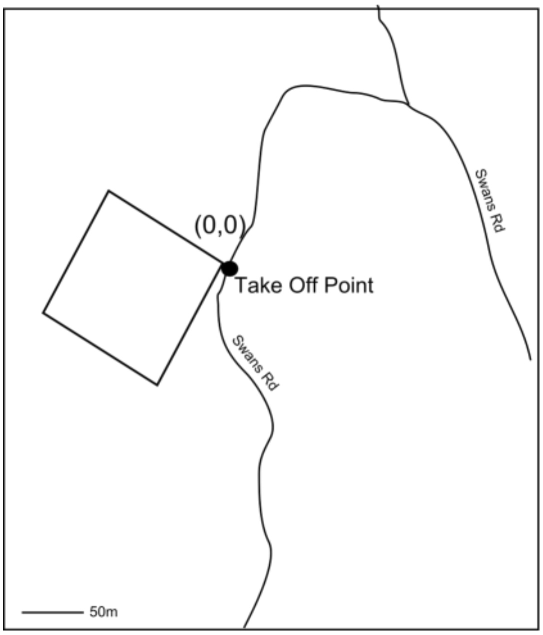

NSFNNC007: Bruxner

**Target Eucalypt Species:**	*Eucalyptus grandis*	**High severity fire?**	Unknown
**Maximum Tree Height (m)**	65m	**Low severity fire?**	No
**Target Species Growth Stage:**	Mature	**Cut stumps?**	Yes, 12 axe stumps
**Understorey:**	Rainforest	**Other Disturbance?**	Yes, 11 trees ringbarked

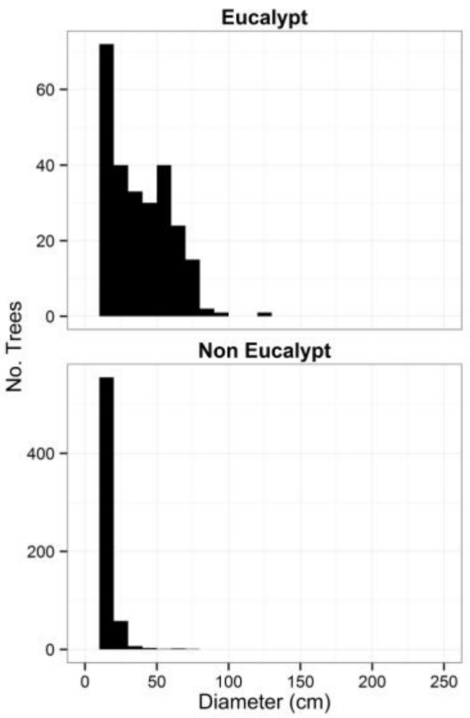

Species	No. Stems	BA (m^2/ha)
Eucalyptus grandis	151	29.1
Eucalyptus pilularis	12	4.2
Lophostemon sp.	56	2.5
Archontophoenix cunninghamiana	134	2.2
Syncarpia glomulifera	26	2.0
Ceratopetalum apetalum	112	1.7
Geissois benthamii	40	1.4
UNNCUSM	63	1.3
Allocasuarina torulosa	29	0.9
Caldcluvia paniculosa	41	0.6
Eucalyptus microcorys	12	0.5
Niemeyeria whitei	19	0.5
Schizomeria ovata	29	0.4
Litsea australis	23	0.4
Guioa semiglauca	6	0.3
Orites excelsus	5	0.2
Callicoma serratifolia	7	0.1
Litsea reticulata	7	0.1
Synoum glandulosum	7	0.1
Alphitonia excelsa	6	0.1
Cryptocarya rigida	1	0.1
Cryptocarya bidwillii	3	0.1
Trochocarpa laurina	2	0.1
Acacia maidenii	1	0.1

Unidentified species: UNNCUnk, UNNCG15, UNNCMYT2, UNNCG2, UNNCG22, UNNCG4, UNNCG9, UNNCPALM2, UNNCG18, UNNCG16, UNNCG10, UNNCG7, UNNCG6, UNNCG21, UNNCU19, UNNCG20

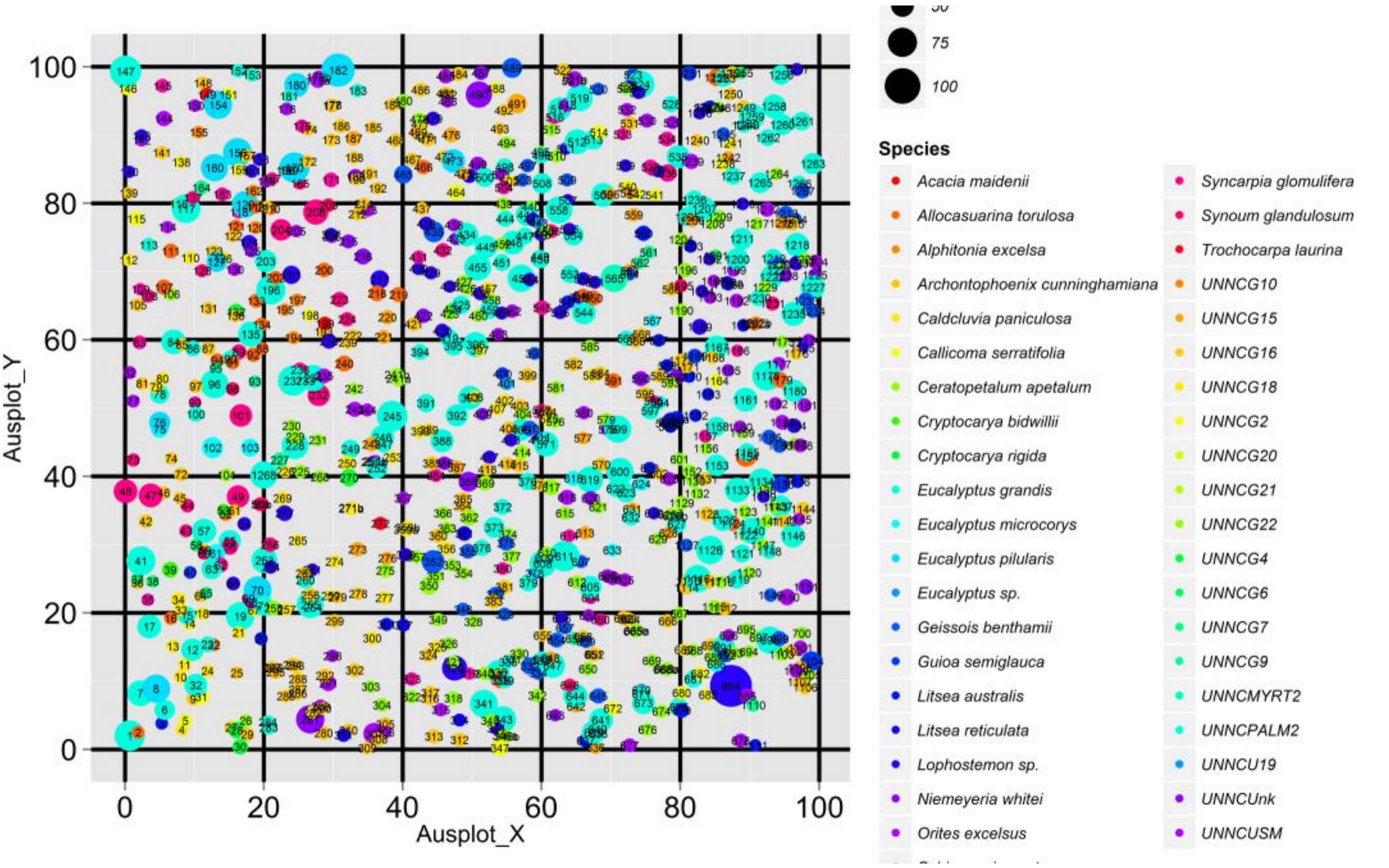

NSFNNC008: O’Sullivans

**AusPlot ID**	NSFNNC008	**Elevation**	75m
**AusPlot Name**	O’Sullivans	**Aspect**	180°
**State**	New South Wales	**Slope**	3°; Gently Inclined
**Bioregion**	NSW North Coast	**Landform Element**	Lower Slope
**Location (UTM)**	56 J 430275 6421029	**MAT, MAP**	17.6 ^{o}C, 1323 mm
**Location (Lat/Long)**	-32.3455 152.2605	**Existing Plot Custodian**	NA
**Tenure**	Bruxner Flora Reserve	**Existing Plot ID**	NA
**Plot Est. Date**	20 November 2013	**Existing Plot Area**	NA
**Plot Size**	1.0ha (100mx100m)	**Existing Plot Census**	NA

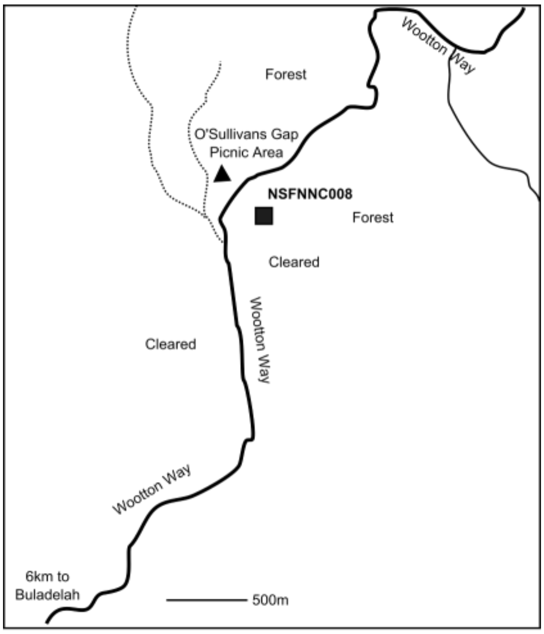

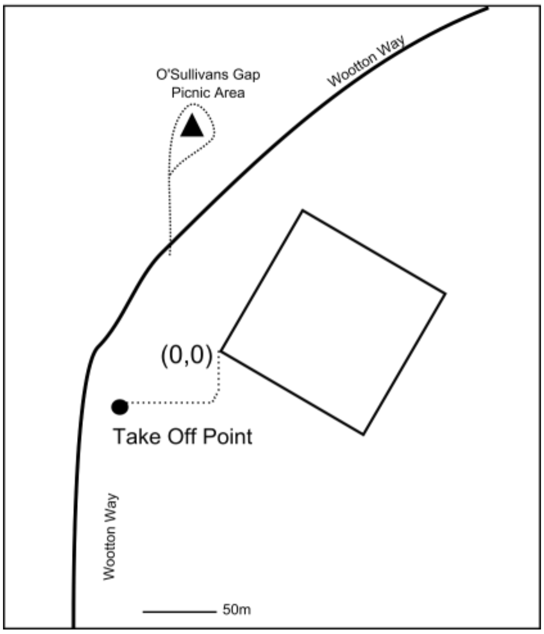

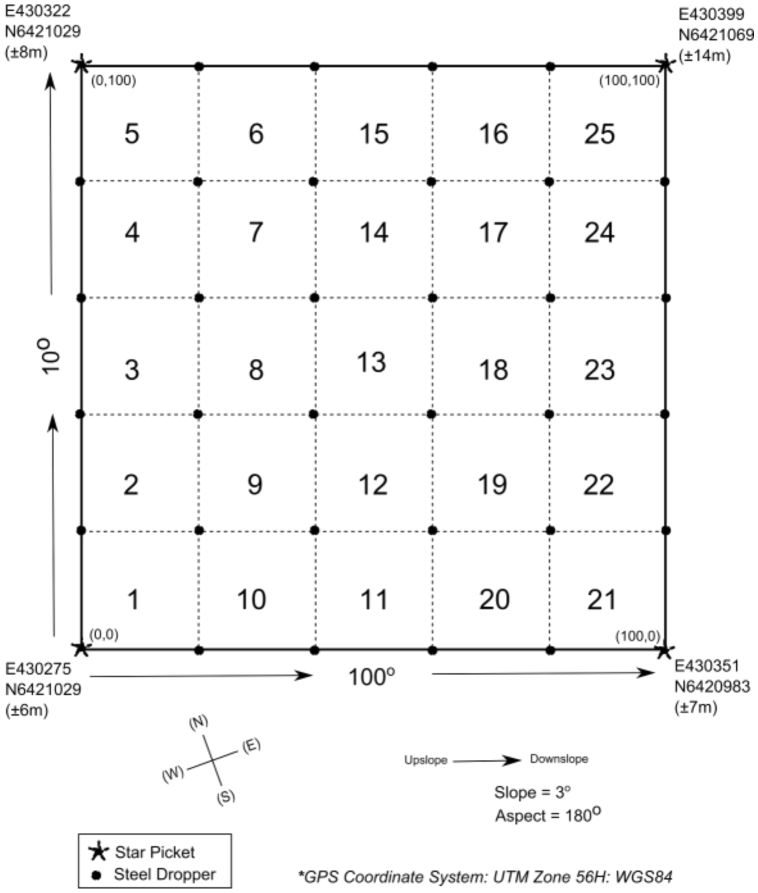

NSFNNC008: O'Sullivans

Target Eucalypt Species: *Eucalyptus grandis*

Maximum Tree Height (m) 66m

Target Species Growth Stage: Mature

Understorey: Wet Sclerophyll/Rainforest

High severity fire? Unknown

Low severity fire? Yes, frequency unknown (Fire Scars)

Cut stumps? Yes, 9 chainsaw and axe stumps

Other Disturbance? No

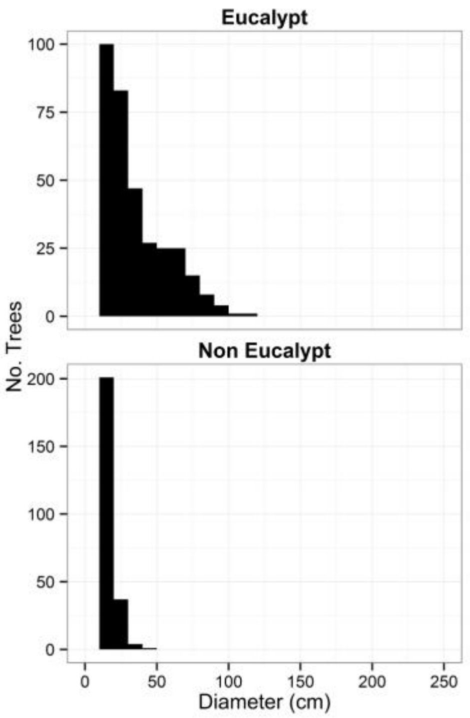

Species	No. Stems	BA (m^2/ha)
Eucalyptus grandis	108	27.8
UNNCEUC4	63	6.8
Syncarpia glomulifera	87	3.6
UNNCEUC1	37	2.8
Cryptocarya microneura	93	2.4
UNNCEUC3	23	2.0
Cryptocarya glaucescens	47	0.9
Allocasuarina torulosa	23	0.7
Lophostemon sp.	13	0.6
Acmena smithii	26	0.5
Caldcluvia paniculosa	15	0.2
UNNCEUC2	5	0.2
UNNCG28	12	0.1
UNNCG24	2	0.1
Schizomeria ovata	7	0.1
Synoum glandulosum	5	0.1
Ceratopetalum apetalum	2	0.1
Cryptocarya rigida	3	0.1
UNNCG26	3	0.1
Callicoma serratifolia	1	0.1
Niemeyeria whitei	1	0.1

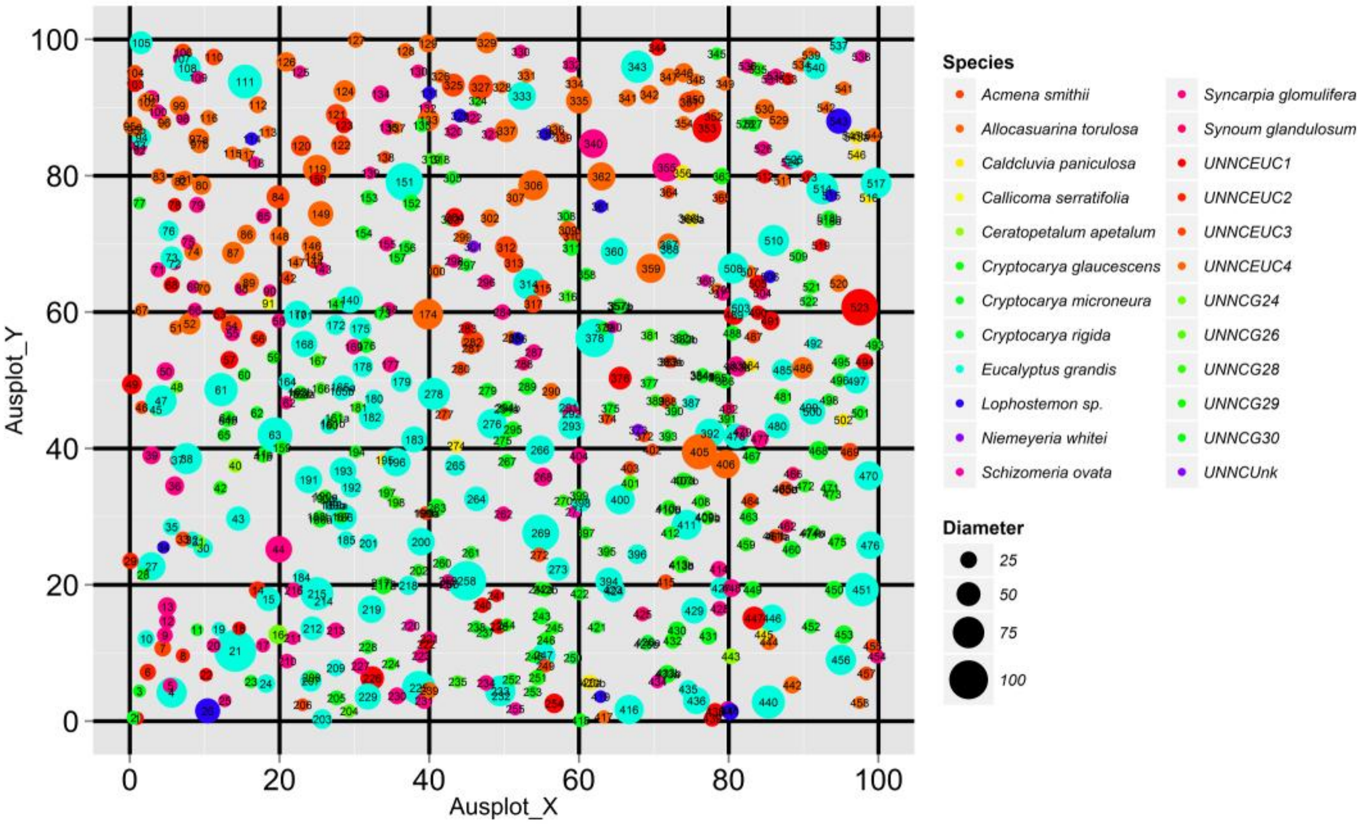

Far North Queensland (QDF)

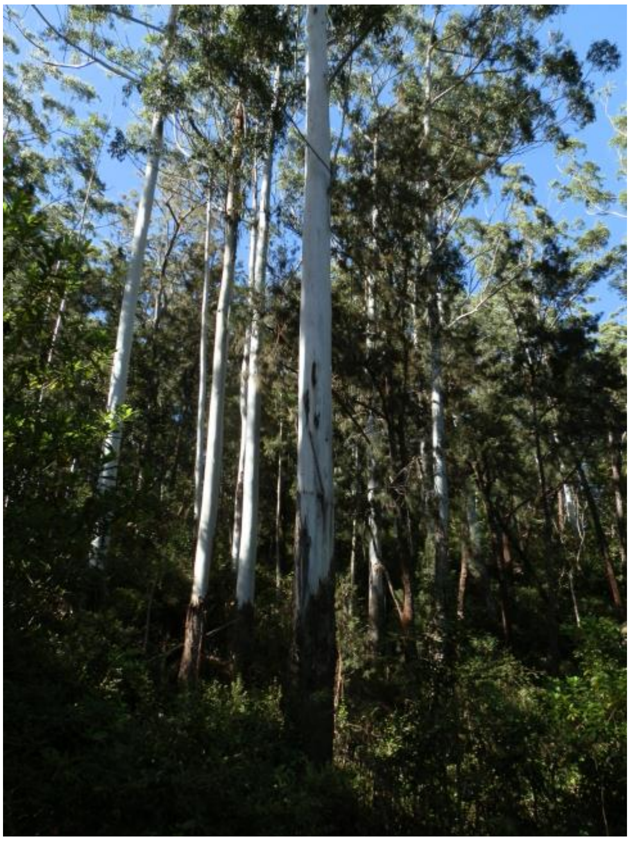

Eucalyptus grandis at QDFWET003 (Lamb Range)

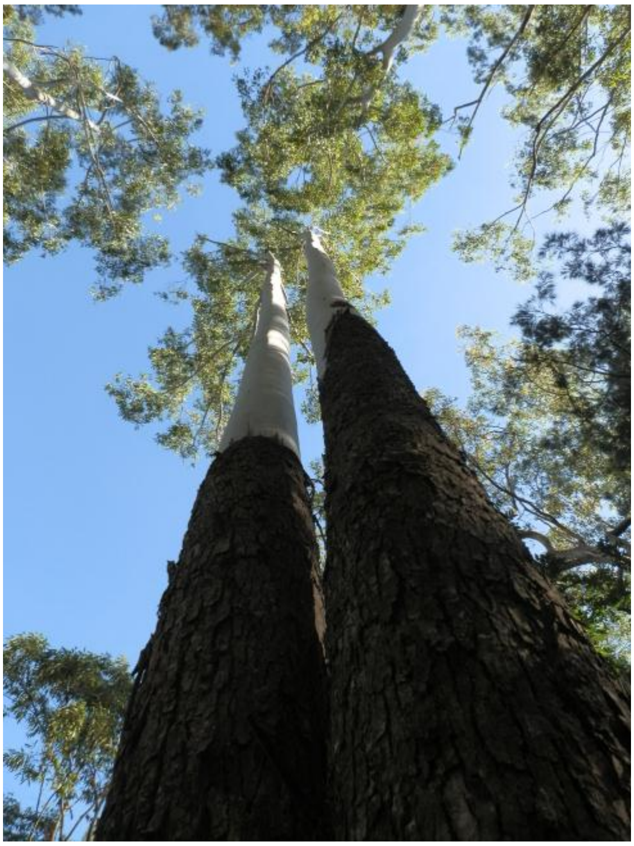

Eucalyptus grandis at QDFWET003 (Lamb Range)

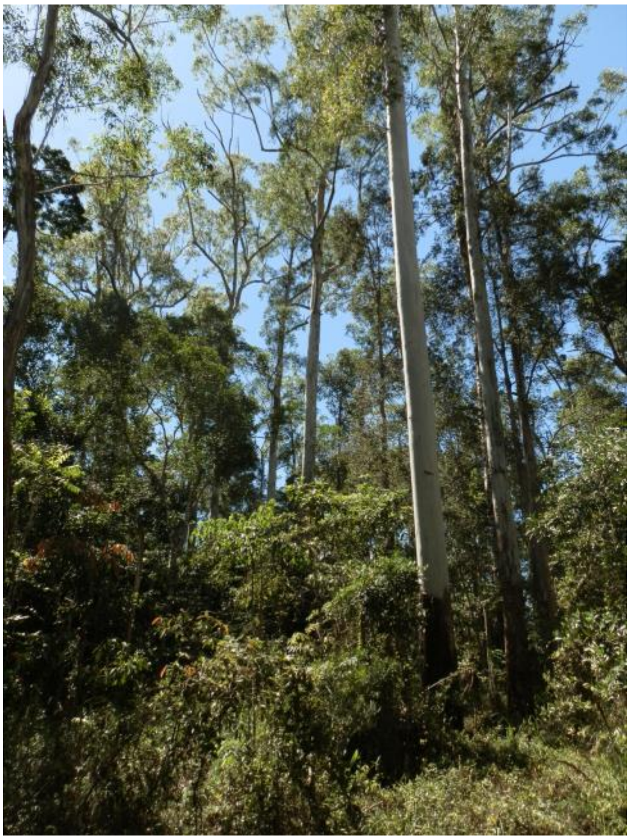

Eucalyptus grandis at QDFWET004 (Koombaloomba)

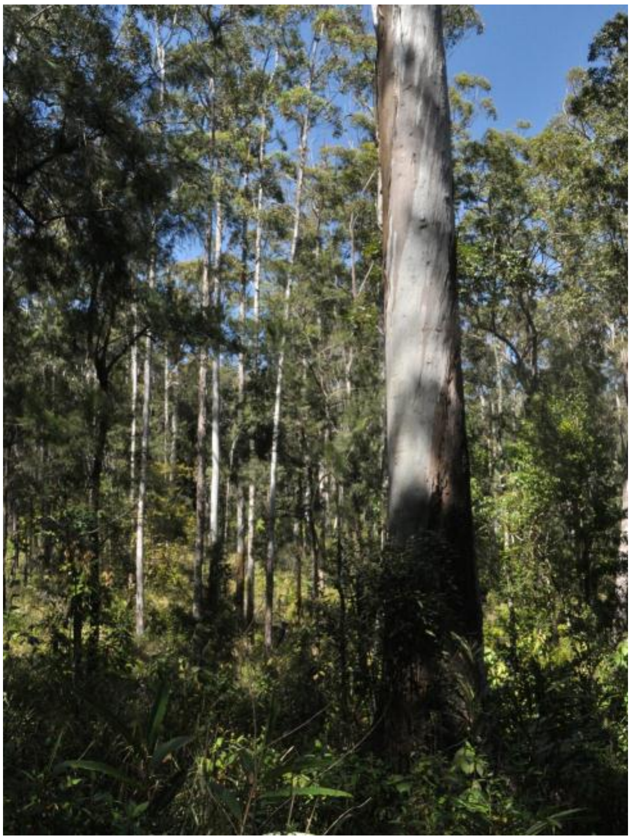

Eucalyptus grandis at QDFWET002 (Longlands Gap)

Far North Queensland (QDF)

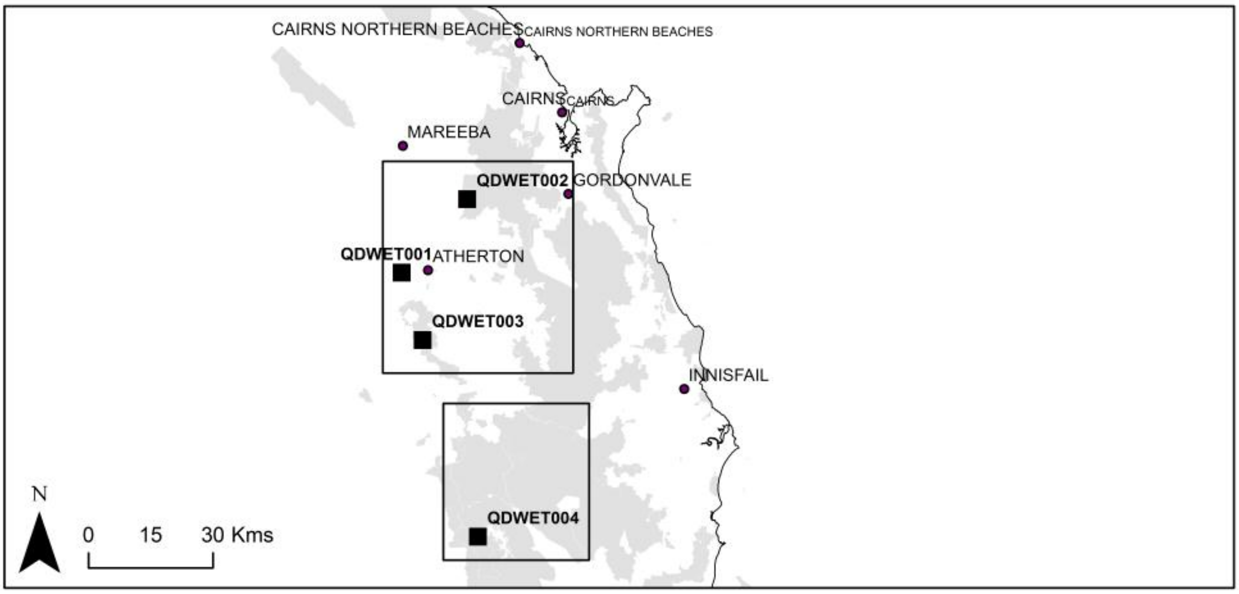

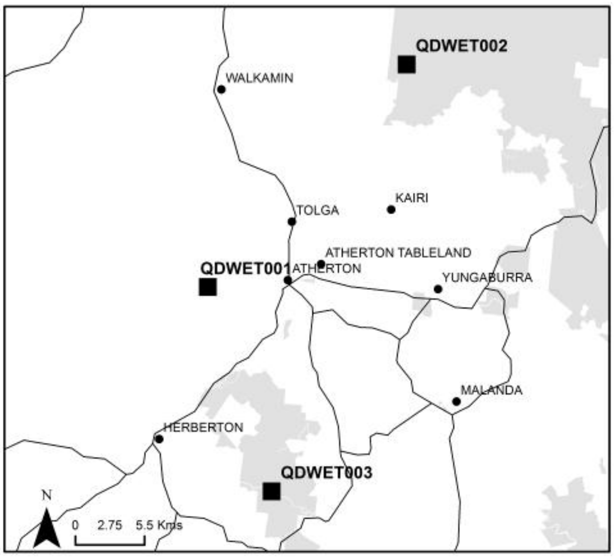

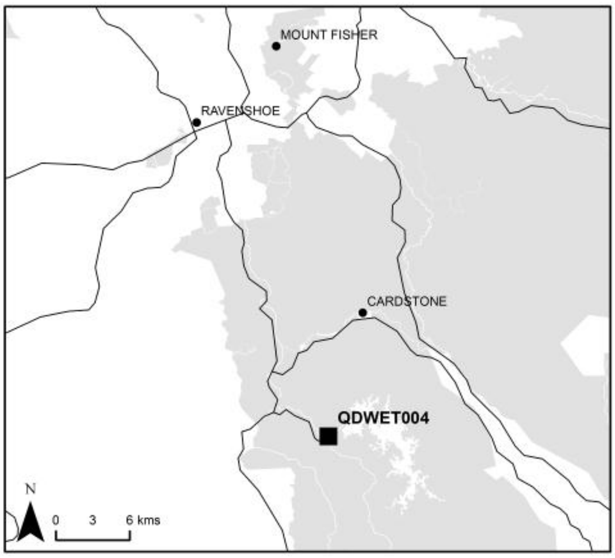

QDFWET001: Baldy

**AusPlot ID**	QDFWET001	**Elevation**	1058m
**AusPlot Name**	Baldy	**Aspect**	270°
**State**	Queensland	**Slope**	2°; Gently Inclined
**Bioregion**	Wet Tropics	**Landform Element**	Lower Slope
**Location (UTM)**	55 K 331915 8089923	**MAT, MAP**	19.4 ^{o}C, 1726 mm
**Location (Lat/Long)**	–17.2698 145.4187	**Existing Plot Custodian**	NA
**Tenure**	Baldy Forest Reserve	**Existing Plot ID**	NA
**Plot Est. Date**	27 August 2014	**Existing Plot Area**	NA
**Plot Size**	1.0ha (100mx100m)	**Existing Plot Census**	NA

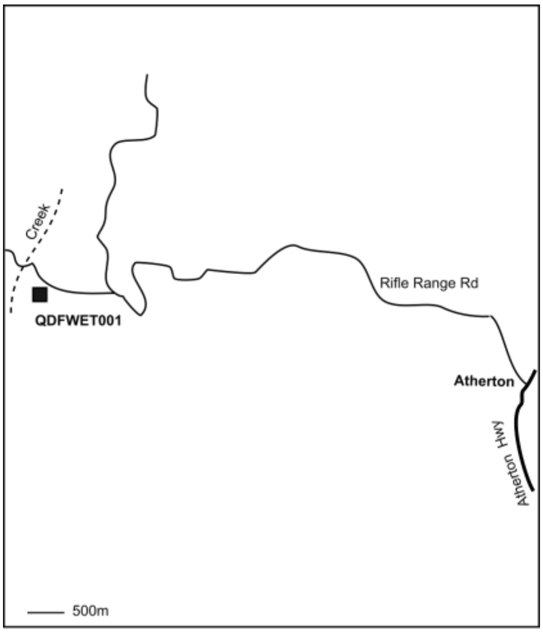

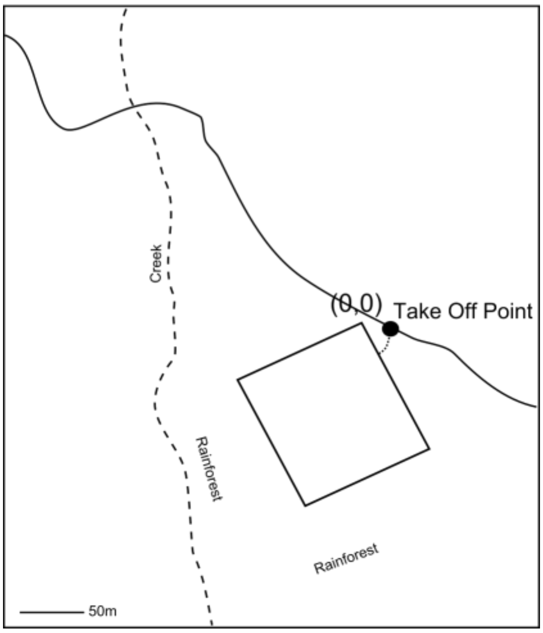

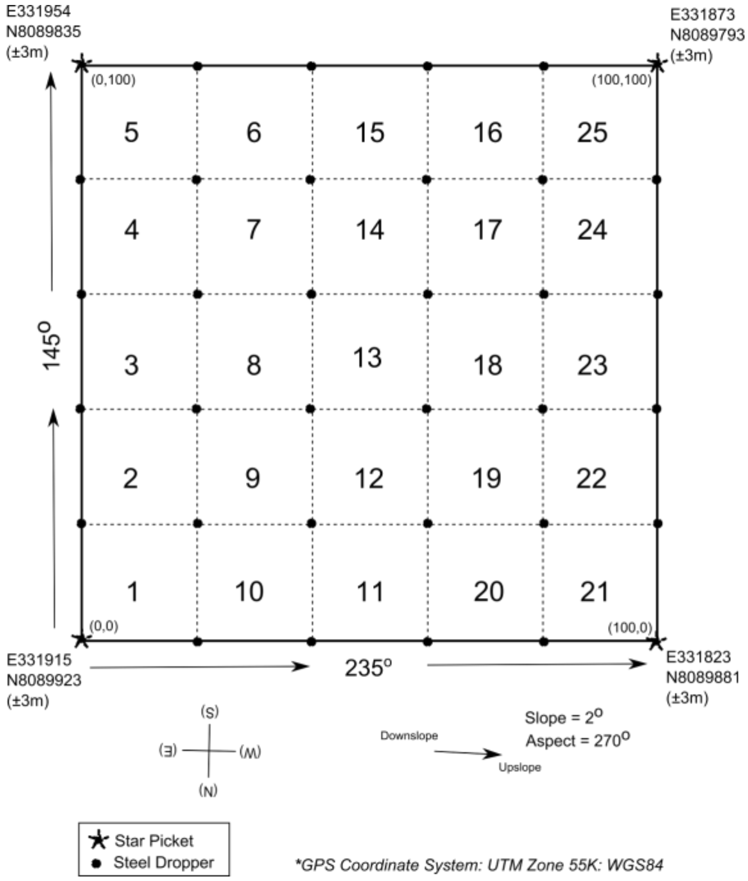

QDFWET001: Baldy

**Target Eucalypt Species:**	*Eucalyptus grandis*	**High severity fire?**	Unknown
**Maximum Tree Height (m)**	50m	**Low severity fire?**	Yes, frequency unknown (Fire Scars)
**Target Species Growth Stage:**	Mature	**Cut stumps?**	Yes, 2 cut stumps
**Understorey:**	Grass/Rainforest	**Other Disturbance?**	No

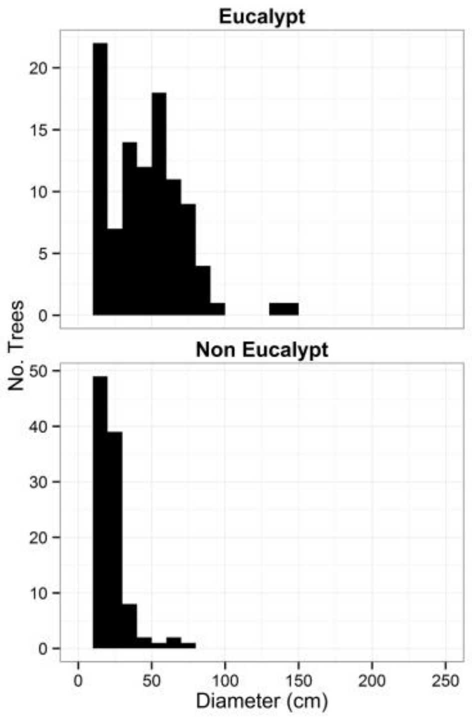

Species	No. Stems	BA (m^2/ha)
Eucalyptus grandis	64	18.3
Corymbia intermedia	36	3.9
Flindersia brayleyana	12	1.6
Allocasuarina torulosa	34	1.5
Acacia selsa	6	0.4
Euroschinus falcata	7	0.2
Archontophoenix cunninghamiana	5	0.2
Guioa lacioneura	4	0.2
Glochidion hylandii	1	0.1
Guioa acutifolia	3	0.1
Alstonia muelleriana	2	0.1
Banksia aquilonia	1	0.1
Stenocarpus sinuatus	1	0.1
Litsea connorsii	1	0.1
Glochidion harveyanum	3	0.1
Litsea leefeana	2	0.1
UWETLive	3	0.1
Alstonia scholaris	2	<0.1
Neolitsea dealbata	2	<0.1
Melicope sp.	1	<0.1
Lomatia fraxinifolia	1	<0.1
Toona australis	2	<0.1
Syzygium oleosum	2	<0.1
Dendrocnide photinophylla	2	<0.1

Other Species include Rhodamnia sacilifolia, Darlingia darlingiana, Geijera salicifolia, Cordia dichotoma, Casearia sp., each recorded once and with basal area <0.1

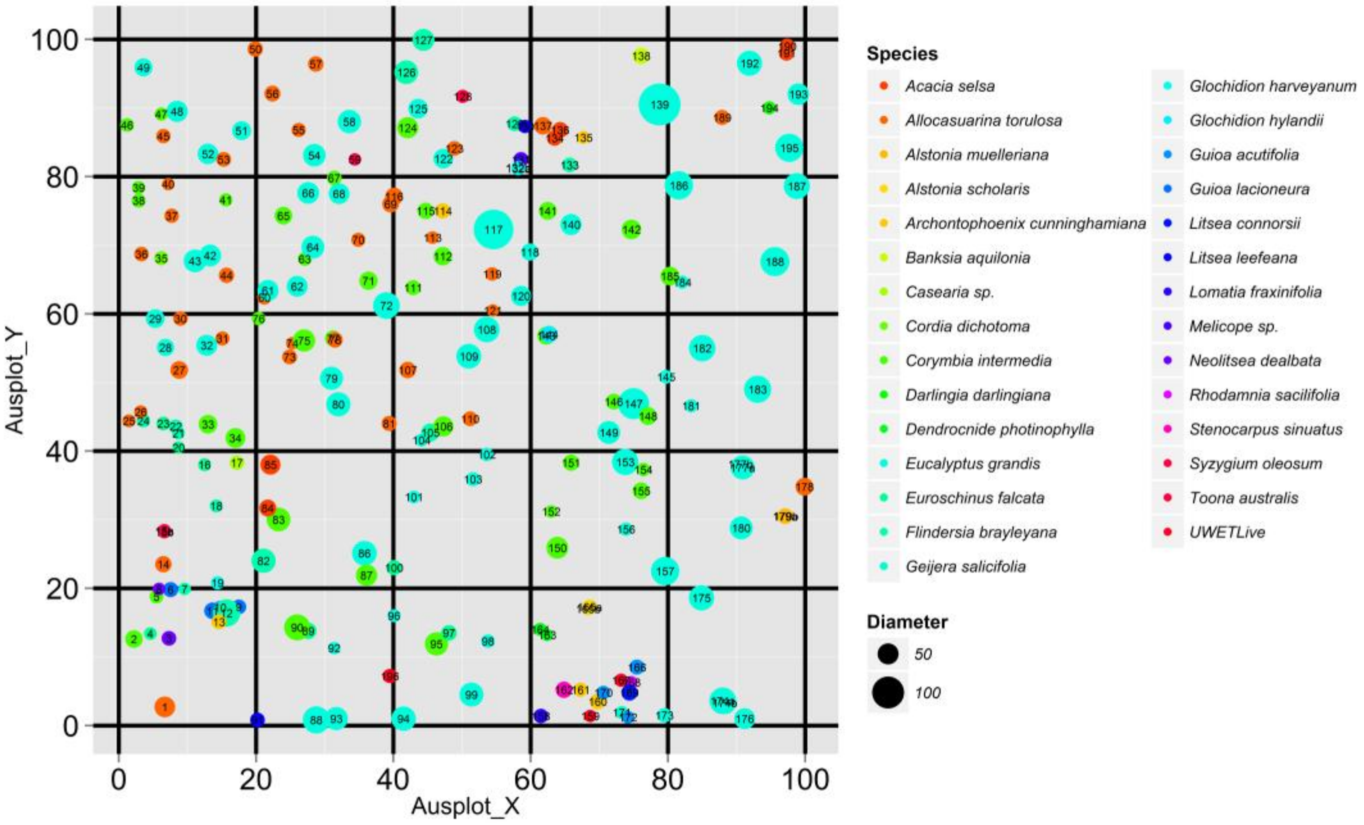

QDFWET002: Longlands

**AusPlot ID**	QDFWET002	**Elevation**	1048m
**AusPlot Name**	Herberton	**Aspect**	360°
**State**	Queensland	**Slope**	5°; Gently Inclined
**Bioregion**	Wet Tropics	**Landform Element**	Upper Slope
**Location (UTM)**	55 K 336964 8073780	**MAT, MAP**	19.4 ^{o}C, 1375 mm
**Location (Lat/Long)**	–17.4158 145.4644	**Existing Plot Custodian**	NA
**Tenure**	Herberton R. NP	**Existing Plot ID**	NA
**Plot Est. Date**	31 August 2014	**Existing Plot Area**	NA
**Plot Size**	1.0ha (100mx100m)	**Existing Plot Census**	NA

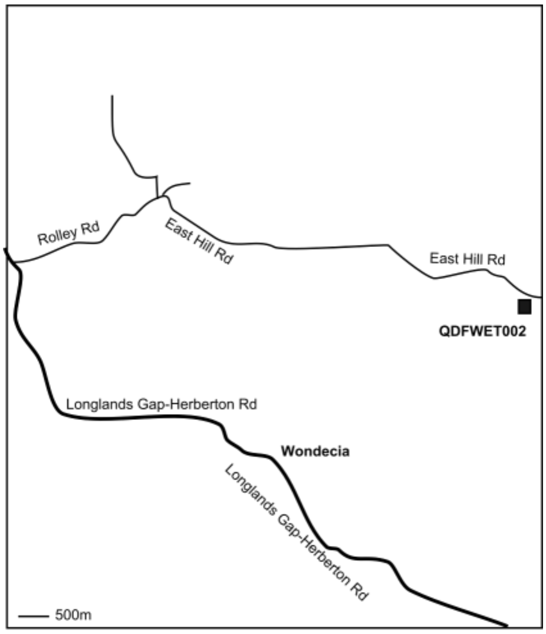

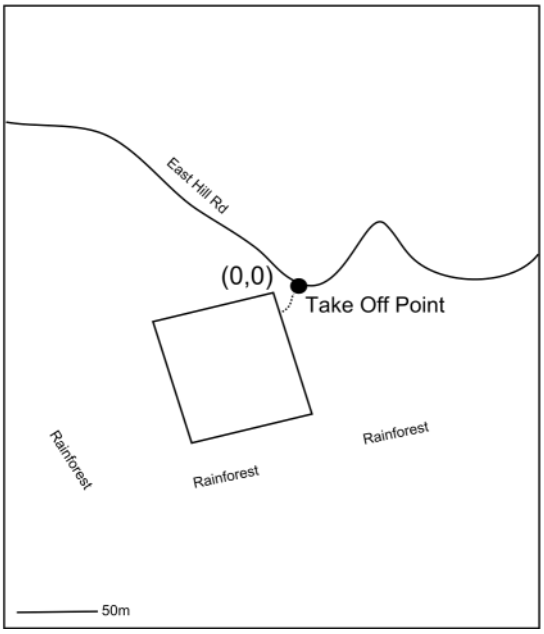

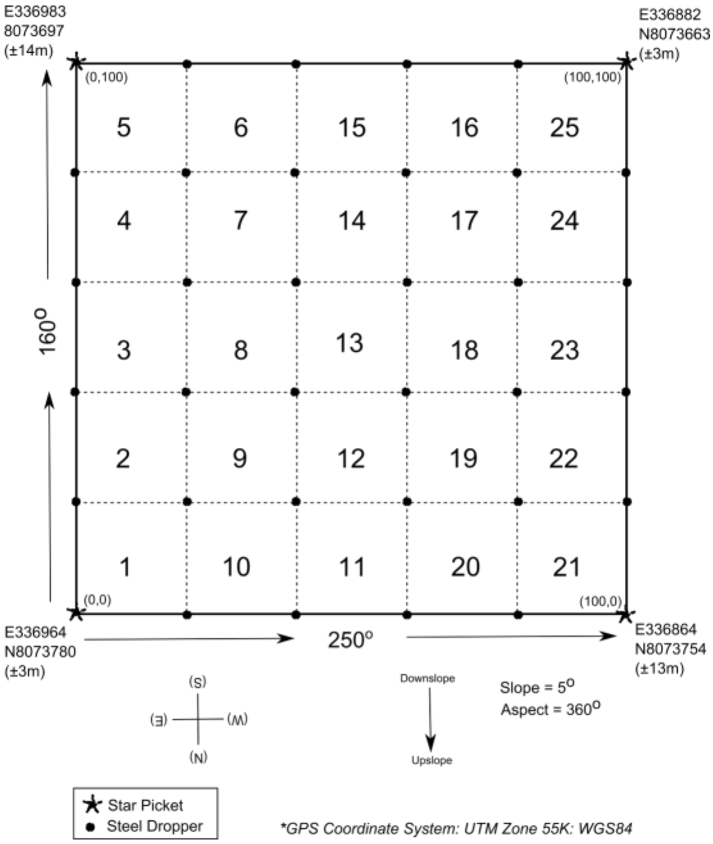

QDFWET002: Longlands

**Target Eucalypt Species:**	*Eucalyptus grandis*	**High severity fire?**	Unknown
**Maximum Tree Height (m)**	40m	**Low severity fire?**	Yes, frequency unknown (Fire Scars)
**Target Species Growth Stage:**	Mature	**Cut stumps?**	Yes, 9 cut stumps
**Understorey:**	Grass	**Other Disturbance?**	No

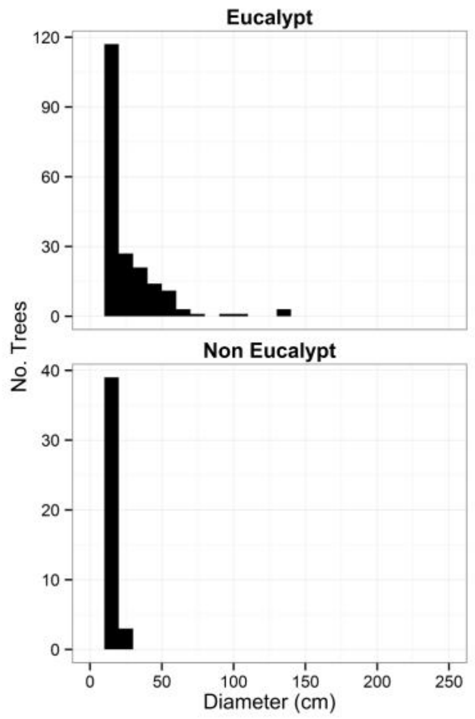

Species	No. Stems	BA (m^2/ha)
Eucalyptus grandis	75	11.6
Corymbia intermedia	124	5.3
Acacia melanoxylon	42	0.6

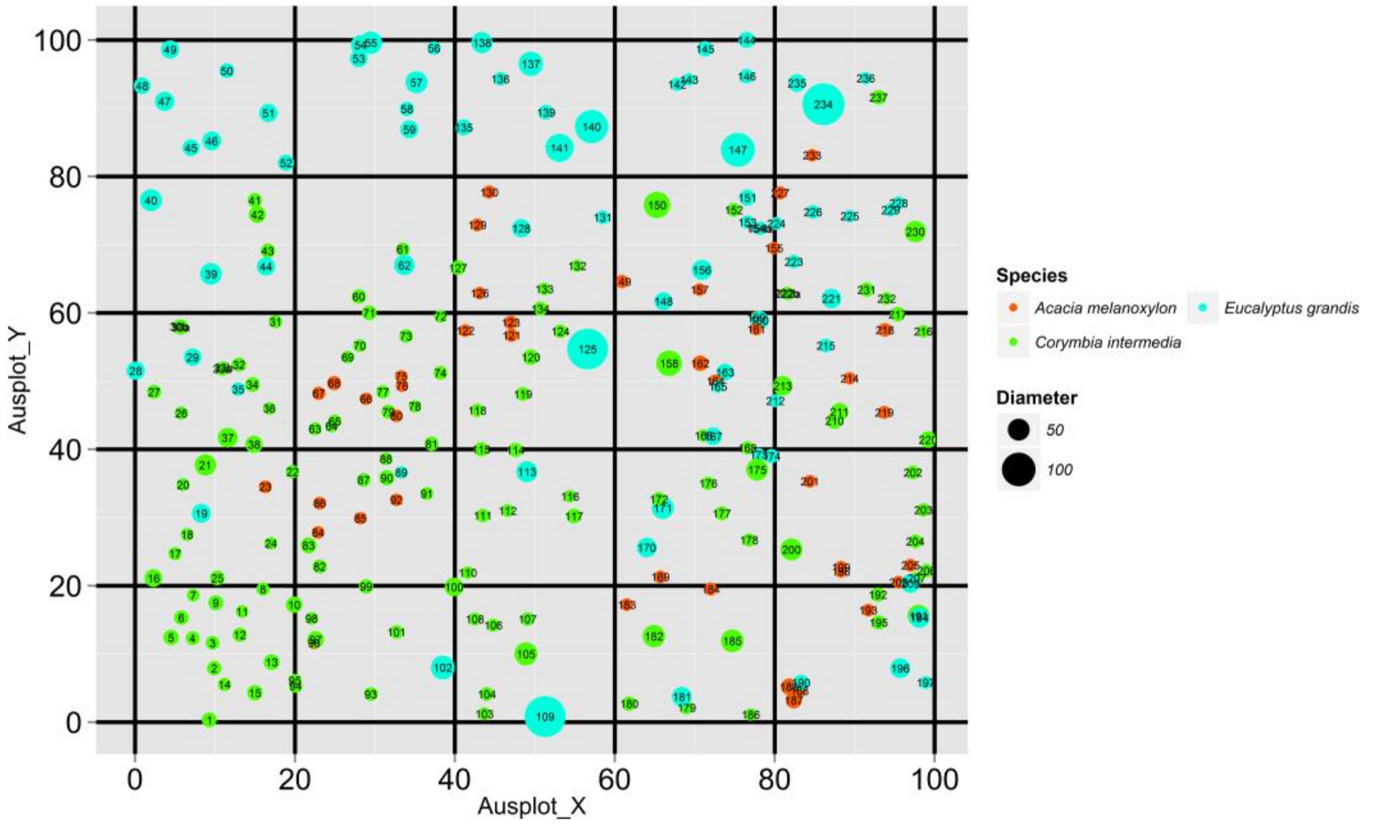

QDFWET003: Lamb Range

**AusPlot ID**	QDFWET003	**Elevation**	1148m
**AusPlot Name**	Lamb Range	**Aspect**	360°
**State**	Queensland	**Slope**	20°; Steep
**Bioregion**	Wet Tropics	**Landform Element**	Midslope
**Location (UTM)**	55 K 346920 8107629	**MAT, MAP**	18.9 ^{o}C, 1601 mm
**Location (Lat/Long)**	–17.1107 145.5609	**Existing Plot Custodian**	NA
**Tenure**	Danbulla NP	**Existing Plot ID**	NA
**Plot Est. Date**	9 September 2014	**Existing Plot Area**	NA
**Plot Size**	1.0ha (100mx100m)	**Existing Plot Census**	NA

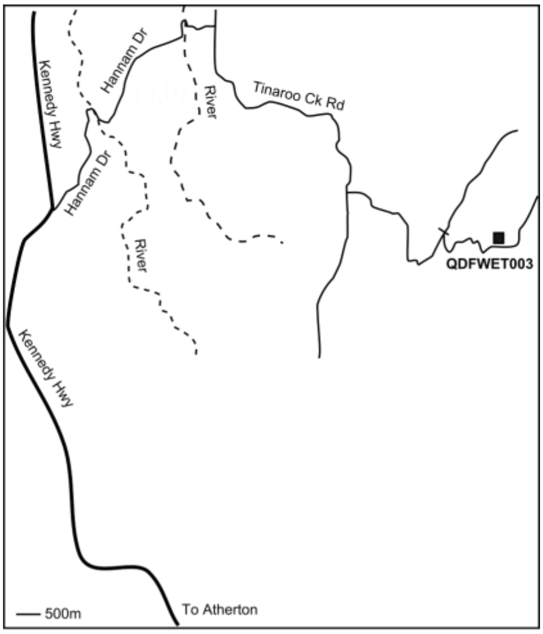

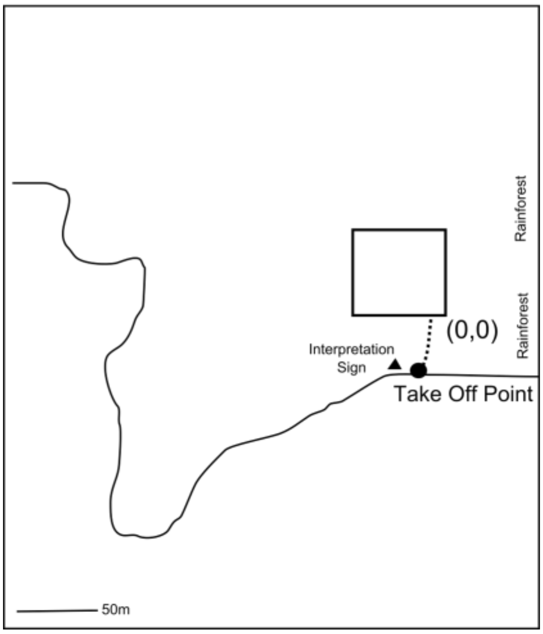

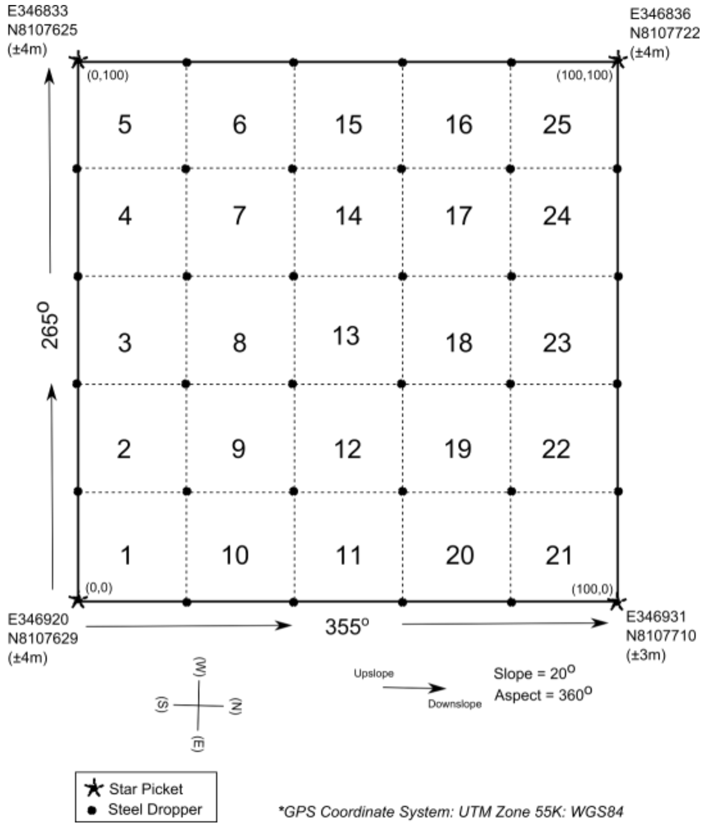

QDFWET003: Lamb Range

**Target Eucalypt Species:**	*Eucalyptus grandis*	**High severity fire?**	Unknown
**Maximum Tree Height (m)**	48m	**Low severity fire?**	Yes, frequency unknown (Fire Scars)
**Target Species Growth Stage:**	Mature	**Cut stumps?**	Yes, 11 cut stumps
**Understorey:**	Wet Sclerophyll/Rainforest	**Other Disturbance?**	No

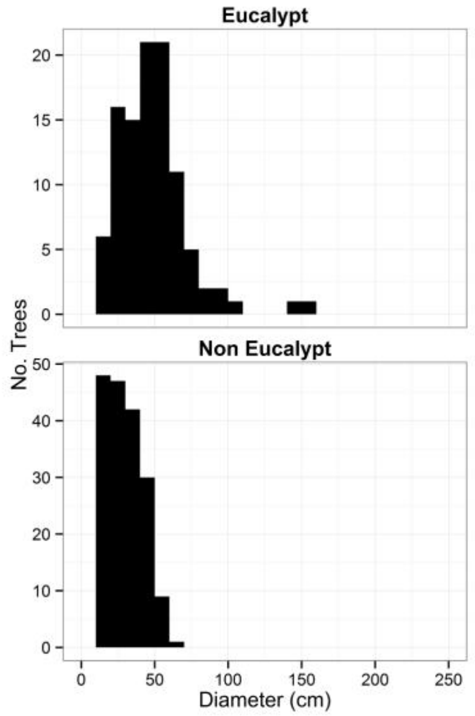

Species	No. Stems	BA (m^2/ha)
Eucalyptus grandis	91	21.3
Allocasuarina torulosa	152	13.2
Corymbia intermedia	4	1.0
Eucalyptus resinifera	7	0.7
Banksia	5	0.6
Glochidion harveyanum	11	0.3
Flindersia brayleyana	2	0.2
Acacia melanoxylon	1	0.1
Hedycarya angustifolia	1	<0.1
Hedycarya loxocarya	1	<0.1
Schizomeria whitei	1	<0.1
Glochidion Harveyanum	1	<0.1
Polyscias sp	1	<0.1
Toona australis	1	<0.1

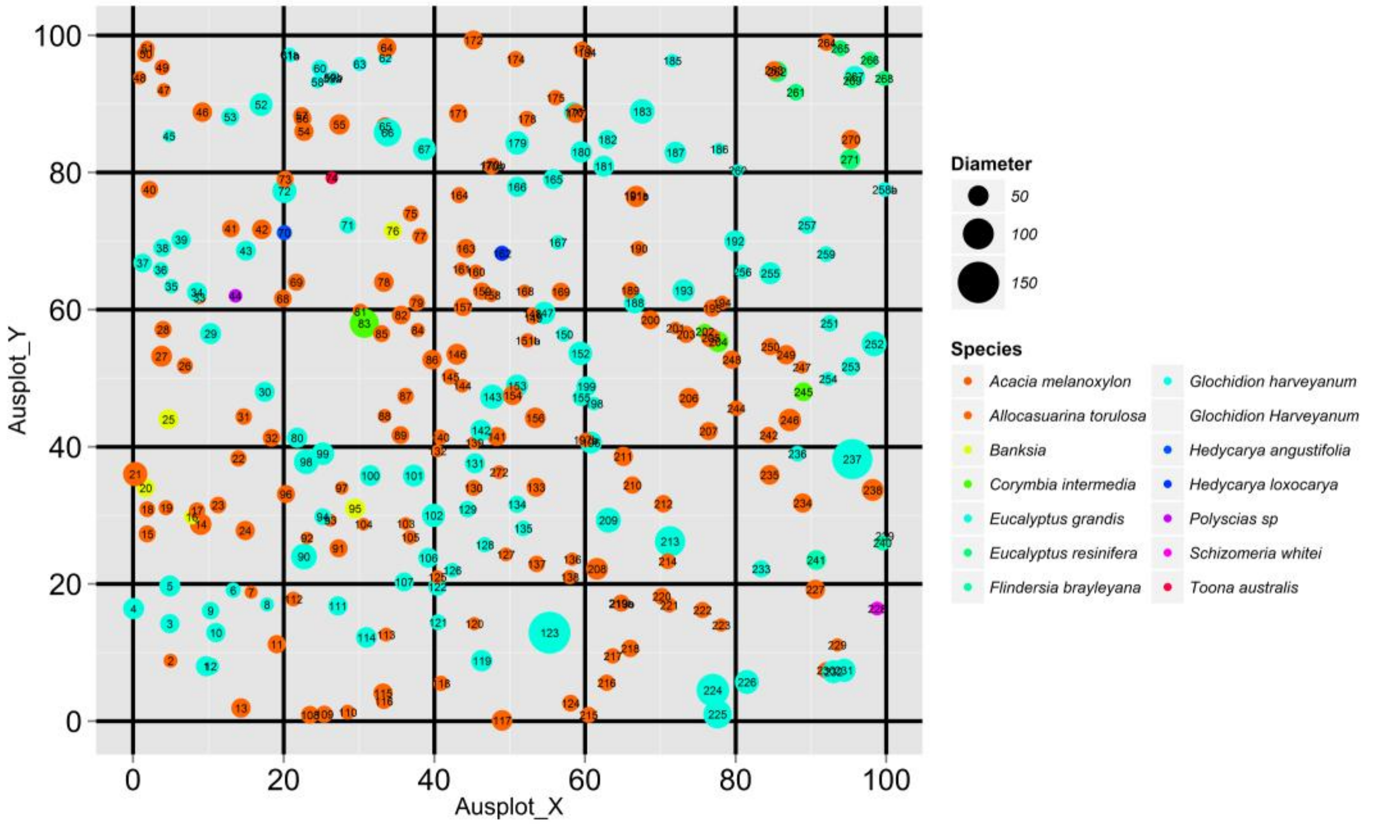

QDFWET004: Koombaloomba

**AusPlot ID**	QDFWET004	**Elevation**	795m
**AusPlot Name**	Koombaloomba	**Aspect**	0°
**State**	Queensland	**Slope**	360°; Steep
**Bioregion**	Wet Tropics	**Landform Element**	Upper Slope
**Location (UTM)**	55 K 350005 8026818	**MAT, MAP**	20.5 ^oC, 1732 mm
**Location (Lat/Long)**	-17.8416 145.5843	**Existing Plot Custodian**	NA
**Tenure**	Koombaloomba NP	**Existing Plot ID**	NA
**Plot Est. Date**	12 September 2014	**Existing Plot Area**	NA
**Plot Size**	1.0ha (100mx100m)	**Existing Plot Census**	NA

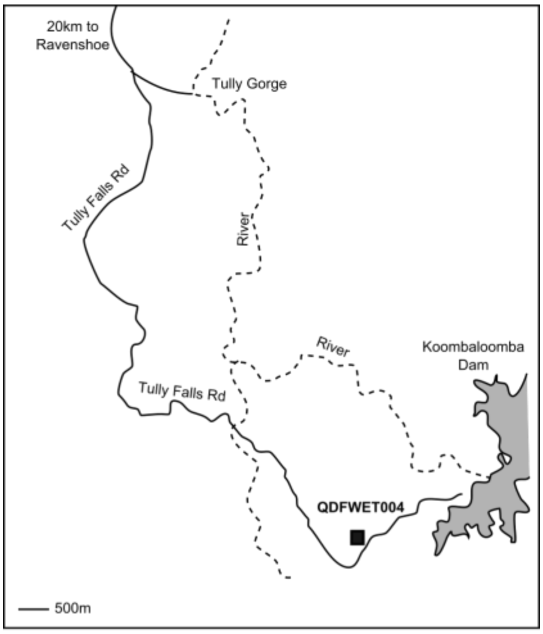

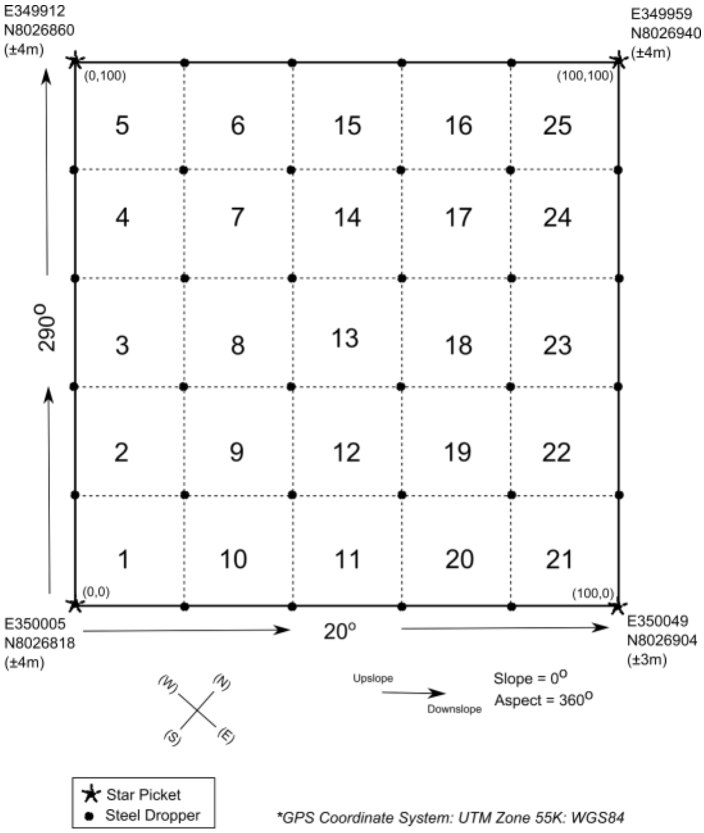

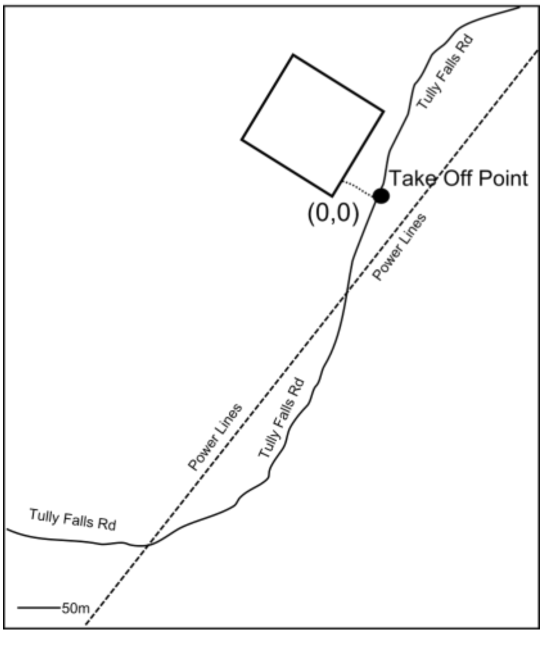

QDFWET004: Koombaloomba

**Target Eucalypt Species:**	*Eucalyptus grandis*	**High severity fire?**	Unknown
**Maximum Tree Height (m)**	47m	**Low severity fire?**	Yes, frequency unknown (Fire Scars)
**Target Species Growth Stage:**	Mature	**Cut stumps?**	Yes, 15 cut stumps
**Understorey:**	Rainforest	**Other Disturbance?**	Pigs damaging understorey

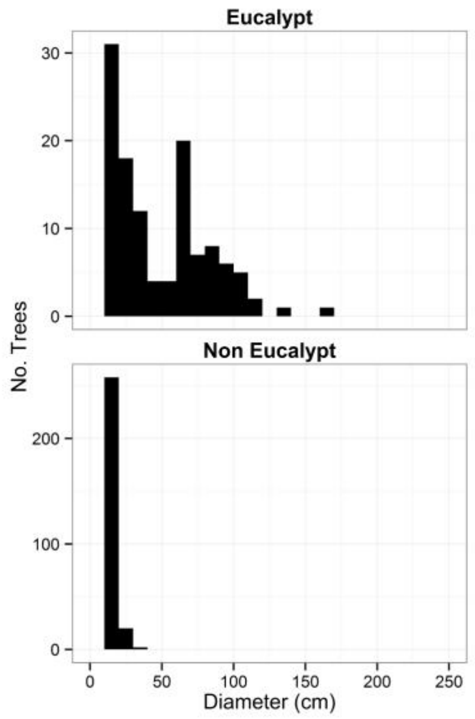

Species	No. Stems	BA (m^2/ha)
Eucalyptus grandis	68	23.1
Syncarpia glomulifera	35	5.6
Acacia melanoxylon	181	3.4
Corymbia intermedia	12	1.9
Eucalyptus resinifera	4	1.4
Polyscias elegans	35	0.4
Sloanea langii	25	0.3
Commersonia bartramia	11	0.2
UWETLive	4	0.1
Acacia cincinnata	3	0.1
Litsea leefeana	3	<0.1
Endiandra hypotephra	1	<0.1
Guioa acutifolia	3	<0.1
Acronychia acronychiodes	3	<0.1
Alphitonia petriei	1	<0.1
Mischocarpus lachnocarpus	1	<0.1
Gmelina fasciculiflora	1	<0.1
Alstonia muelleriana	1	<0.1
Melicope sp.	1	<0.1
Symplocos cochinchiensis	1	<0.1
Euroschinus falcata	1	<0.1
Neolitsea dealbata	1	<0.1
Litsea connorsii	1	<0.1
Cupaniopsis foveolata	1	<0.1
Glochidion sp.	1	<0.1

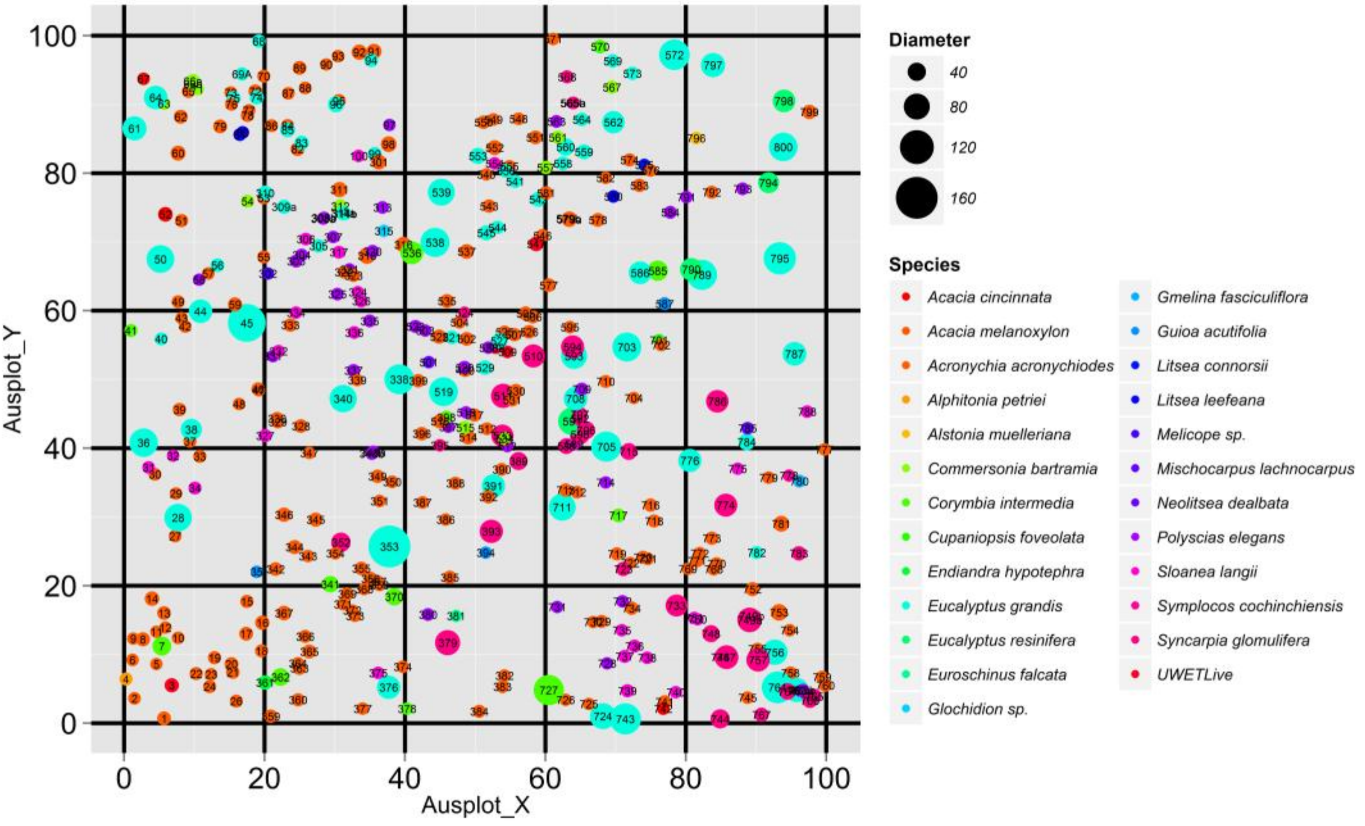

Tasmania (TCF)

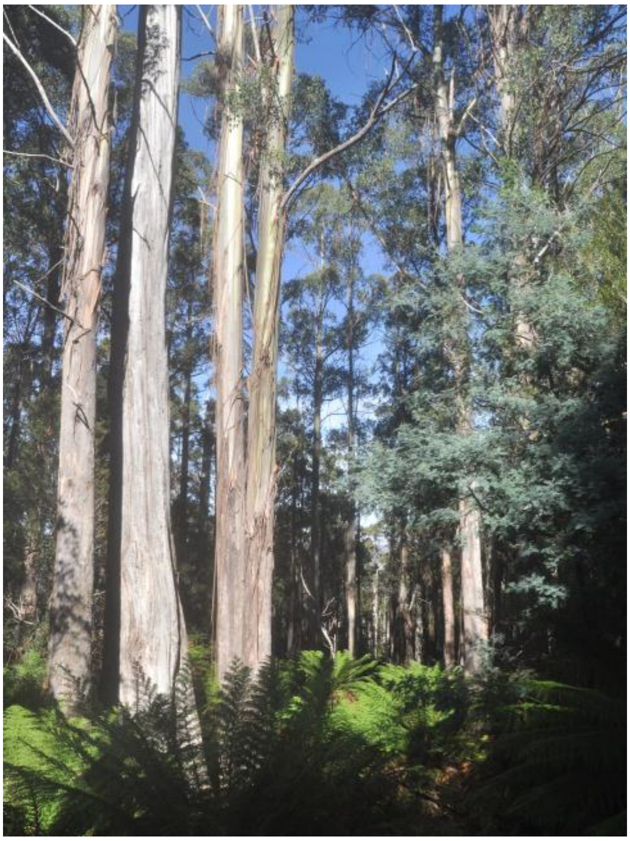

Eucalyptus regnans at TCFTSR003 (Styx Valley)

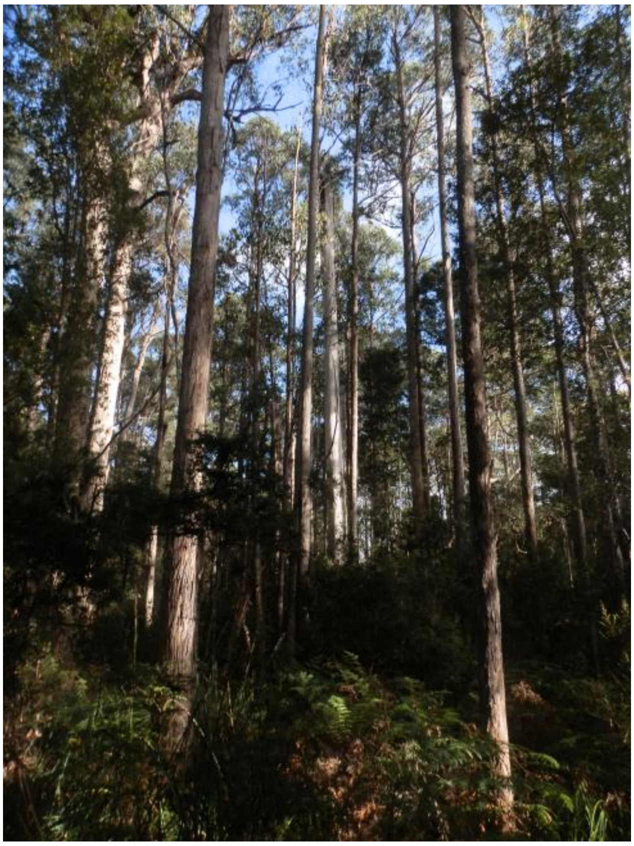

Eucalyptus regnans at TCFTSR004 (Weld)

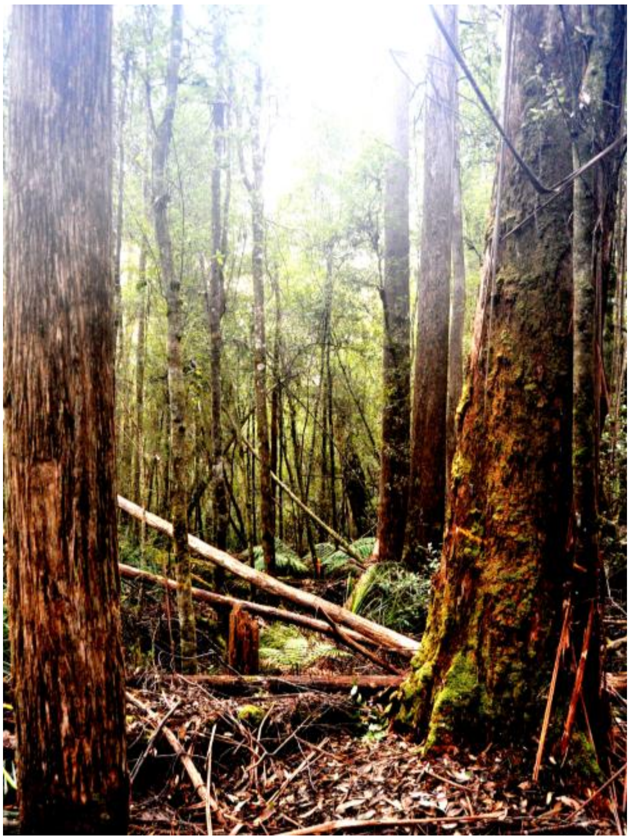

Eucalyptus

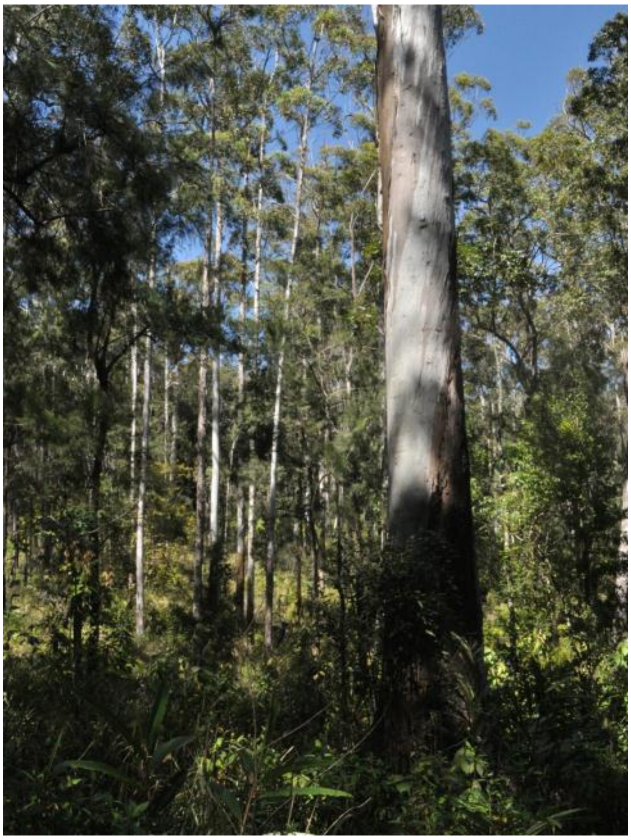

Eucalyptus

Tasmania (TCF)

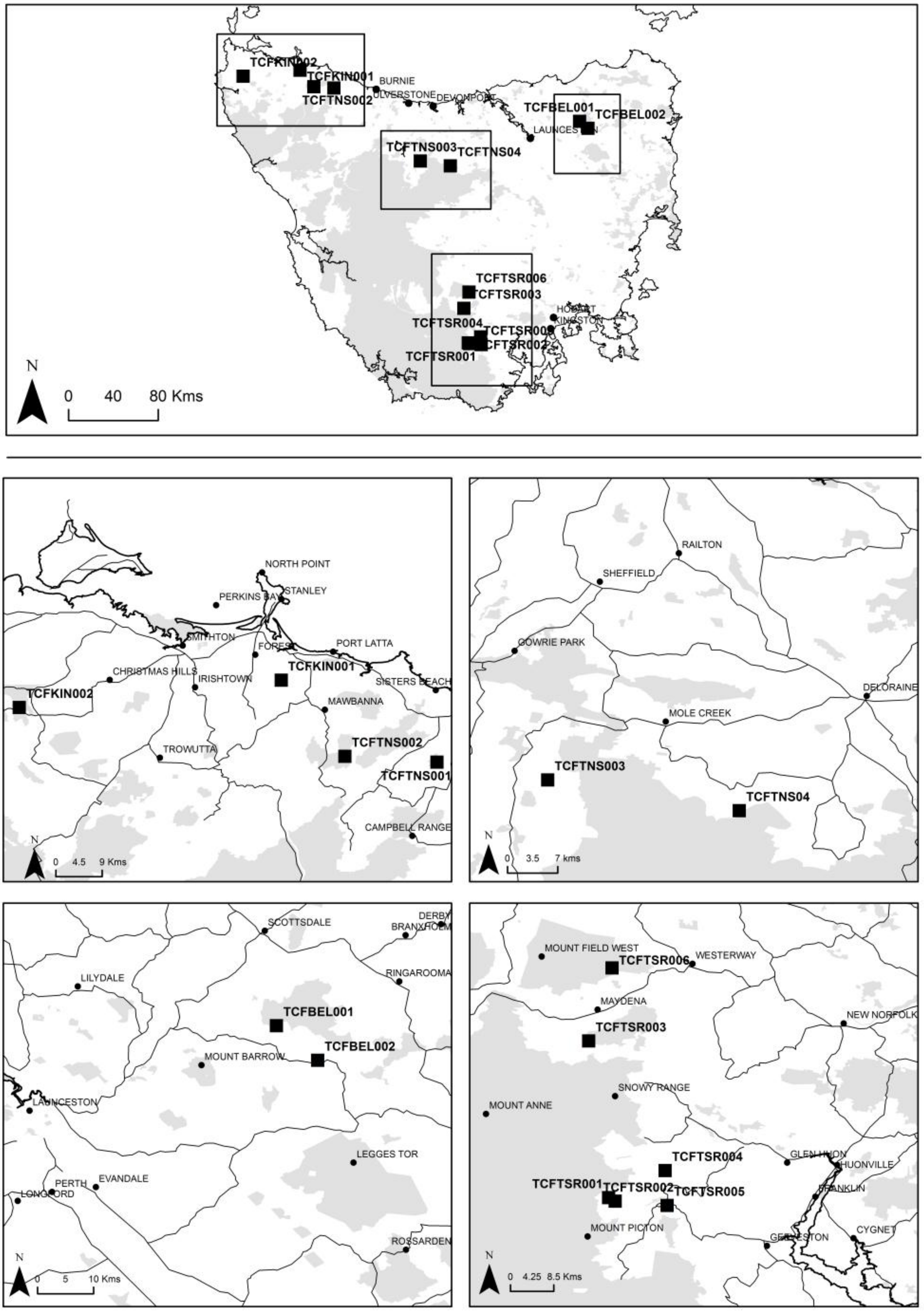

TCFTSR001: Bird Track

**AusPlot ID**	TCFTSR001	**Elevation**	212m
**AusPlot Name**	Bird Track	**Aspect**	180°
**State**	Tasmania	**Slope**	15°; Moderate
**Bioregion**	Tas. Southern Ranges	**Landform Element**	Midslope
**Location (UTM)**	55 G 470964 5229193	**MAT, MAP**	10.3 ^{o}C, 1466 mm
**Location (Lat/Long)**	-43.0891 146.6435	**Existing Plot Custodian**	Forestry Tasmania
**Tenure**	World Heritage Area	**Existing Plot ID**	Wildfire Chronosequence Plot 1934S
**Plot Est. Date**	25 January 2012	**Existing Plot Area**	0.25ha (50mx50m)
**Plot Size**	1.0ha (100mx100m)	**Existing Plot Census**	2007

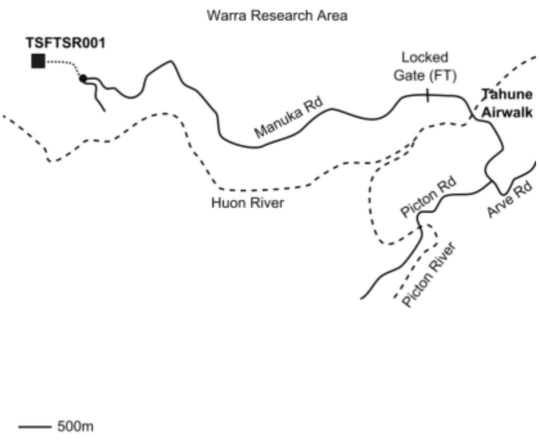

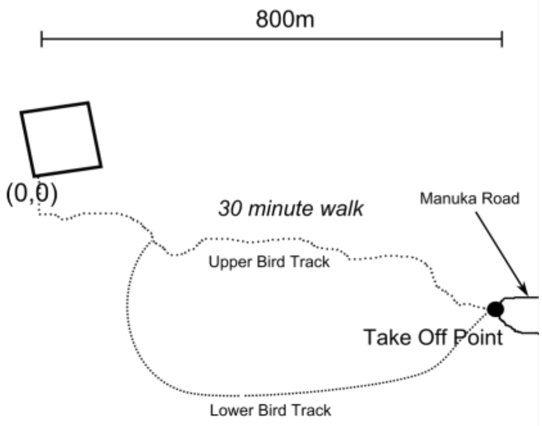

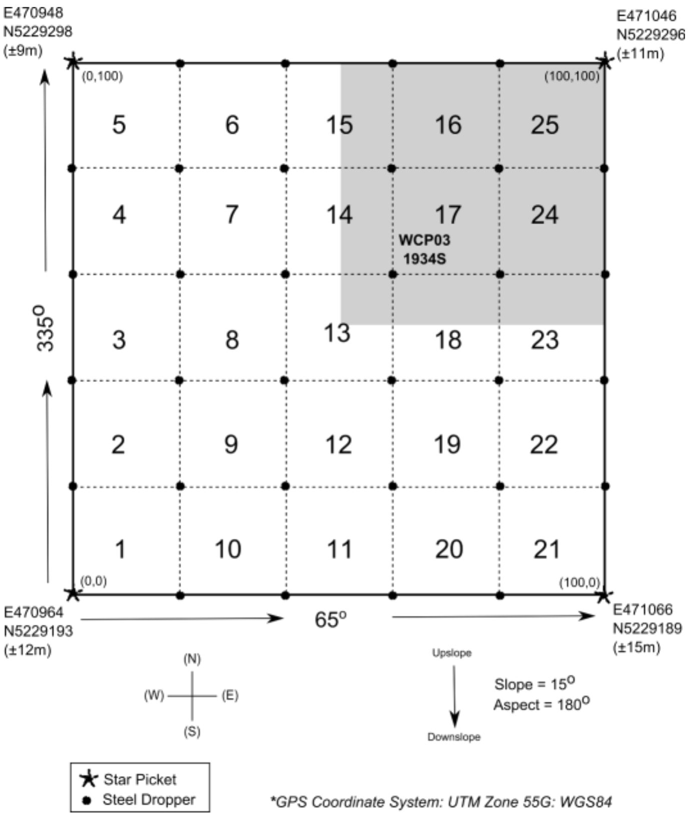

TCFTSR001: Bird Track

**Target Eucalypt Species:**	*Eucalyptus obliqua*	**High severity fire?**	Yes, 1934 (known fire event)
**Maximum Tree Height (m)**	59m	**Low severity fire?**	No
**Target Species Growth Stage:**	Mature, 1934 regrowth	**Cut stumps?**	No
**Understorey:**	Rainforest	**Other Disturbance?**	No

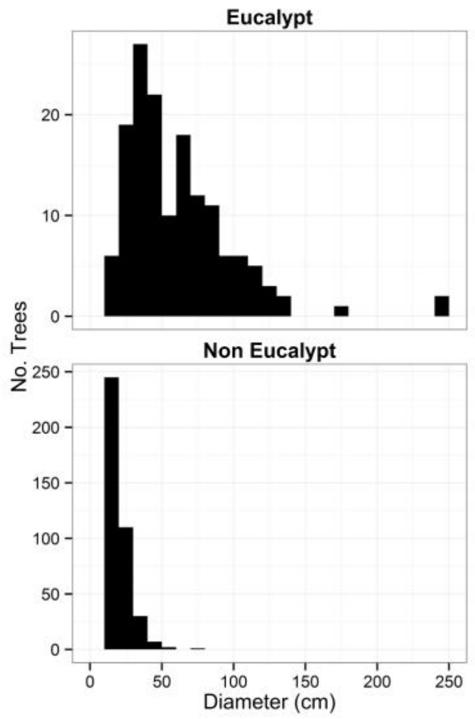

Species	No. Stems	BA (m^2/ha)
Eucalyptus obliqua	149	58.0
Nothofagus cunninghamii	219	7.0
Acacia melanoxylon	58	4.2
Atherosperma moschatum	38	1.3
Eucryphia lucida	16	0.3
Monotoca glauca	27	0.3
Phyllocladus aspleniifolius	20	0.3
Acacia dealbata	4	0.2
Nematolepis squamea	3	0.1
Olearia argophylla	3	0.1
Eucalyptus regnans	1	0.1
Tasmannia lanceolata	5	<0.1
Pittosporum bicolor	2	<0.1

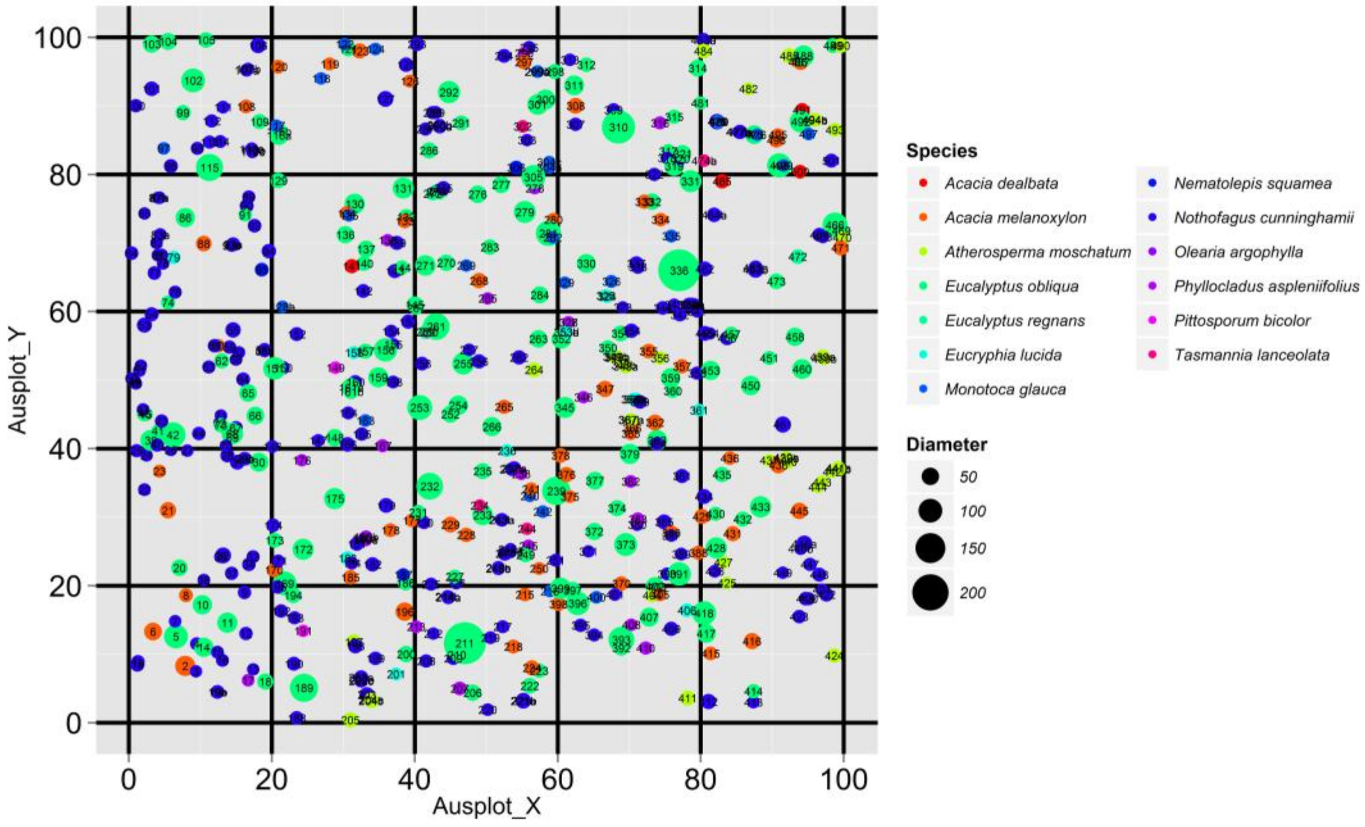

TCFTSR002: Warra Supersite

**AusPlot ID**	TCFTSR002	**Elevation**	111m
**AusPlot Name**	Warra Supersite	**Aspect**	180°
**State**	Tasmania	**Slope**	3°; Gently Inclined
**Bioregion**	Tas. Southern Ranges	**Landform Element**	Flats
**Location (UTM)**	55 G 471794 5228473	**MAT, MAP**	11.2 ^{o}C, 1364 mm
**Location (Lat/Long)**	-43.0959 146.6534	**Existing Plot Custodian**	Forestry Tasmania
**Tenure**	World Heritage Area	**Existing Plot ID**	Warra Supersite
**Plot Est. Date**	25 April 2012	**Existing Plot Area**	1.6ha (100mx1600m)
**Plot Size**	1.6ha (100mx100m)	**Existing Plot Census**	Ecological Surveys and Flux Tower

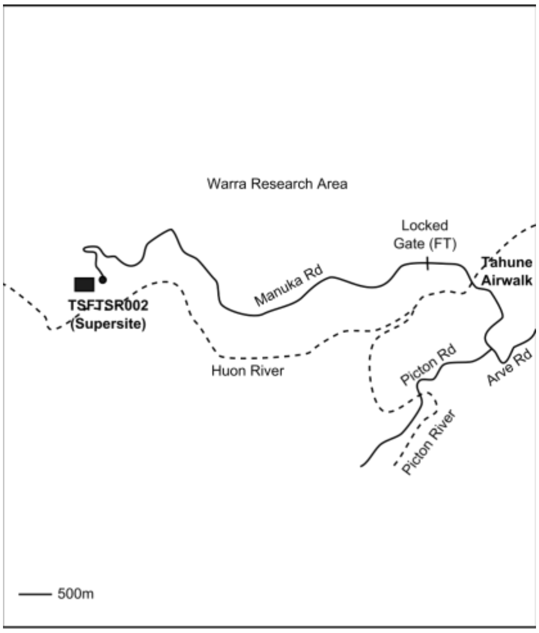

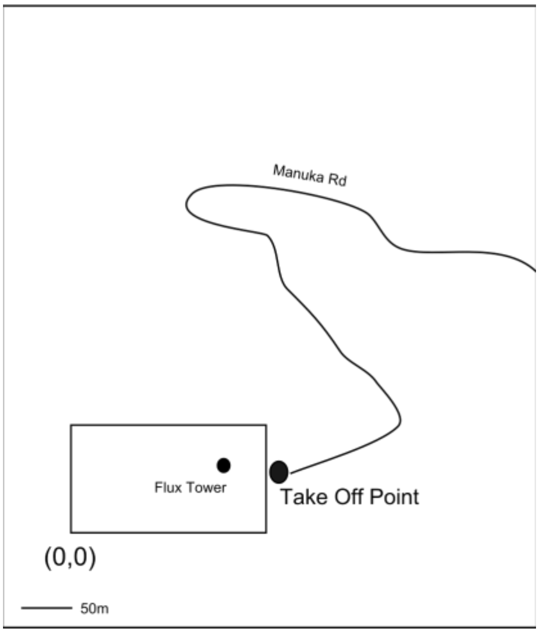

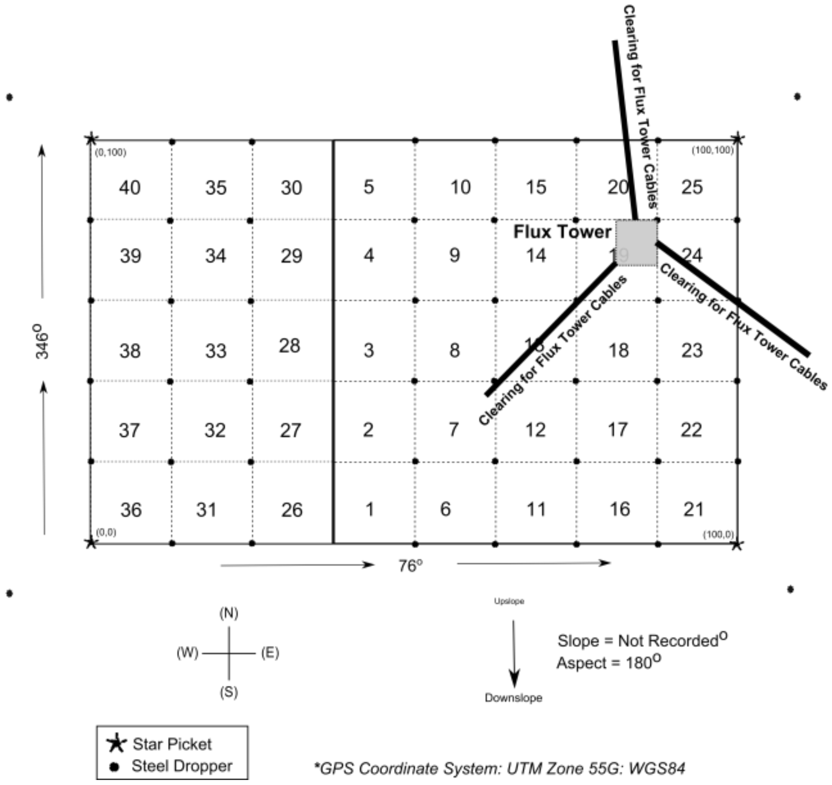

Note: 1.6ha plot with atypical subplot configuration.

60m Tall Flux Tower affects plots 11-25

Plots 1-10 and 26-40 are largely undisturbed (i.e. 0,0 to 100,100).

TCFTSR002: Warra Supersite

**Target Eucalypt Species:**	*Eucalyptus obliqua*	**High severity fire?**	Yes, 1898 (known fire event)
**Maximum Tree Height (m)**	>60m	**Low severity fire?**	No
**Target Species Growth Stage:**	Mature, 1898 regrowth	**Cut stumps?**	Yes, trees removed for Flux Tower
**Understorey:**	Rainforest	**Other Disturbance?**	Infrastructure for Supersite

Note: 1.6ha plot with atypical subplot configuration

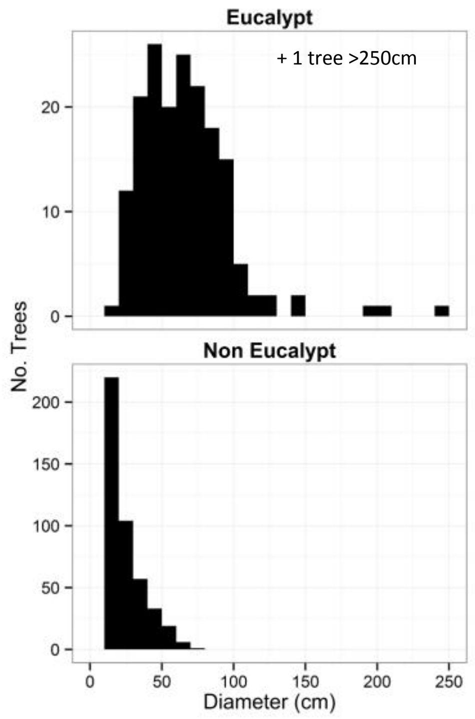

Species	No. Stems	BA (m^2/ha)
Eucalyptus obliqua	176	56.3
Acacia melanoxylon	120	10.1
Nothofagus cunninghamii	146	3.5
Pomaderris apetala	119	1.7
Atherosperma moschatum	26	0.6
Meleleuca ericifolia	8	0.2
Monotoca glauca	6	0.1
Pittosporum bicolor	6	0.1
Phyllocladus aspleniifolius	6	0.1
Eucryphia lucida	1	<0.1
Tasmannia lanceolata	1	<0.1
Acacia verticillata	1	<0.1

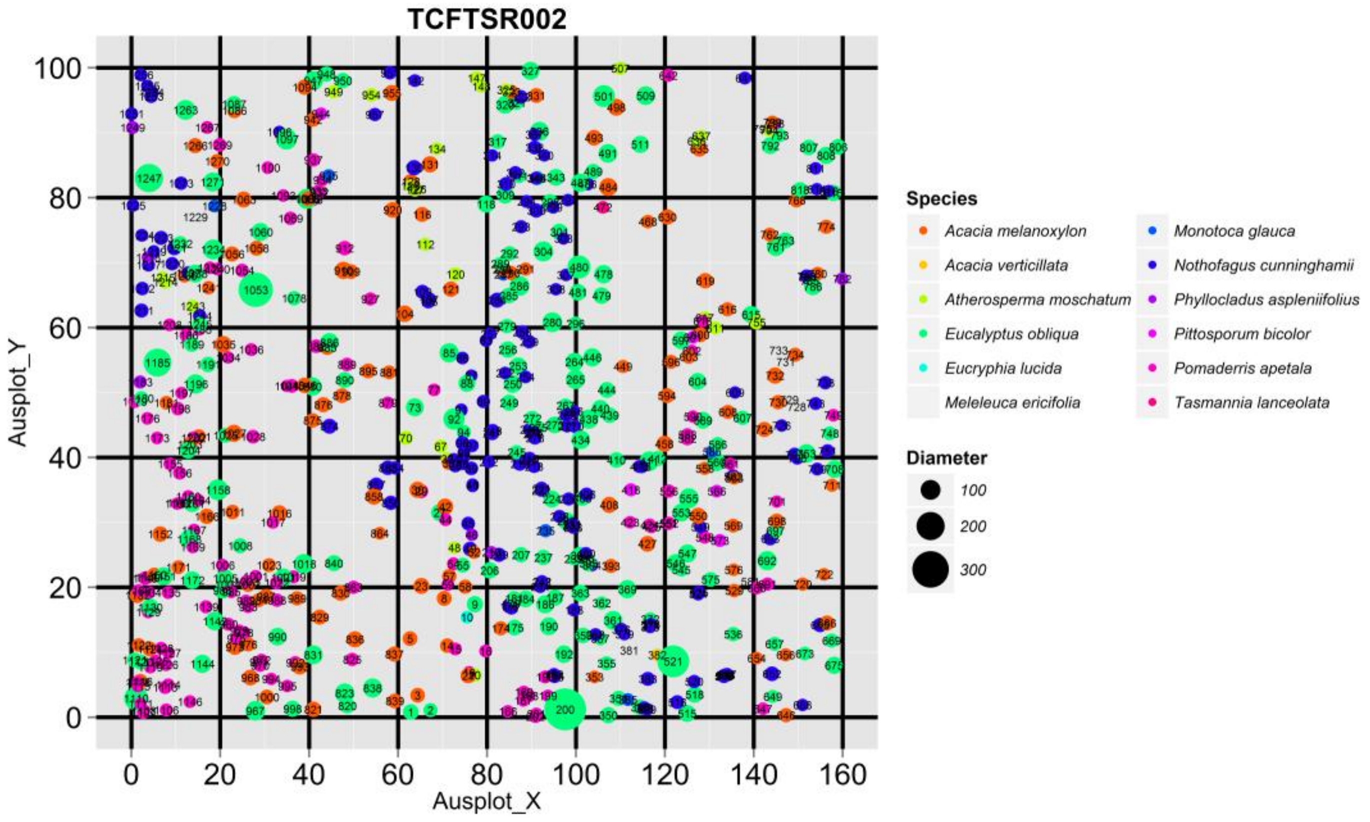

TCFTSR003: North Styx

**AusPlot ID**	TCFTSR003	**Elevation**	560m
**AusPlot Name**	North Styx	**Aspect**	185°
**State**	Tasmania	**Slope**	9°; Moderate
**Bioregion**	Tas. Southern Ranges	**Landform Element**	Midslope
**Location (UTM)**	55 G 468022 5260045	**MAT, MAP**	9.7 ^{o}C, 1299 mm
**Location (Lat/Long)**	-42.8118 146.6083	**Existing Plot Custodian**	NA
**Tenure**	Forest Reserve	**Existing Plot ID**	NA
**Plot Est. Date**	19 April 2013	**Existing Plot Area**	NA
**Plot Size**	1.0ha (100mx100m)	**Existing Plot Census**	NA

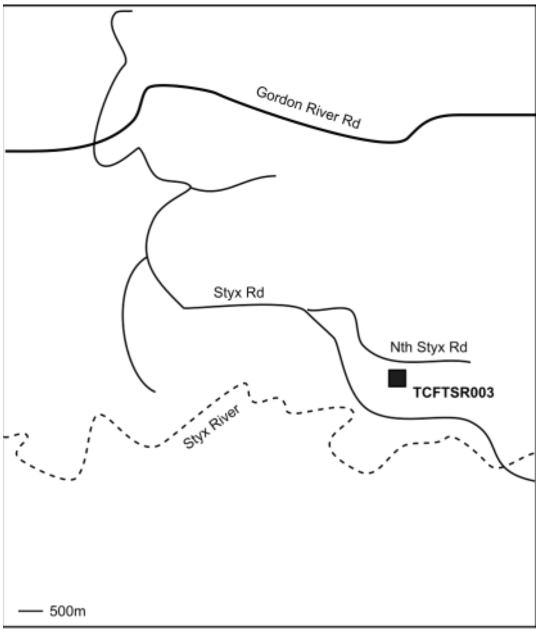

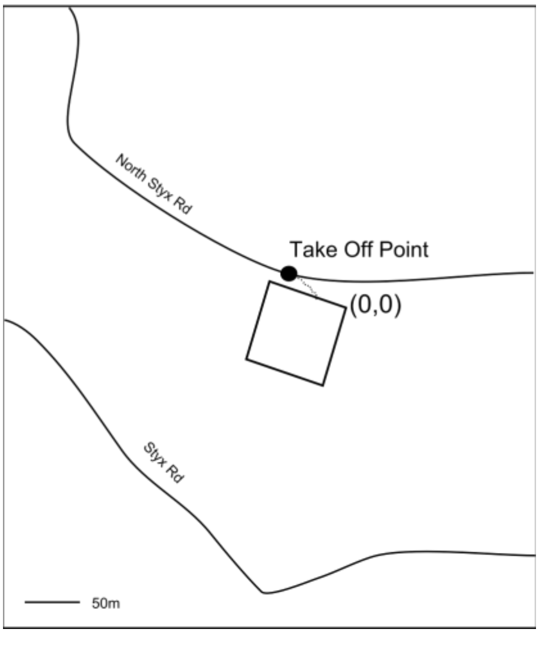

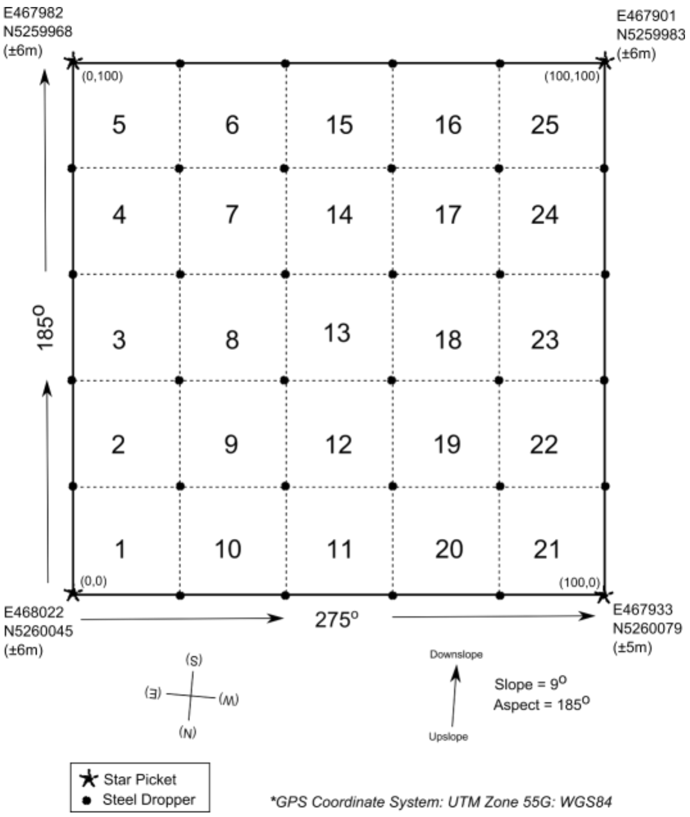

TCFTSR003: North Styx

**Target Eucalypt Species:**	*Eucalyptus regnans*	**High severity fire?**	Yes, probably 1934 (known fire event)
**Maximum Tree Height (m)**	57m	**Low severity fire?**	No
**Target Species Growth Stage:**	Mature, 1934 regrowth	**Cut stumps?**	No
**Understorey:**	Wet Sclerophyll/Rainforest	**Other Disturbance?**	No

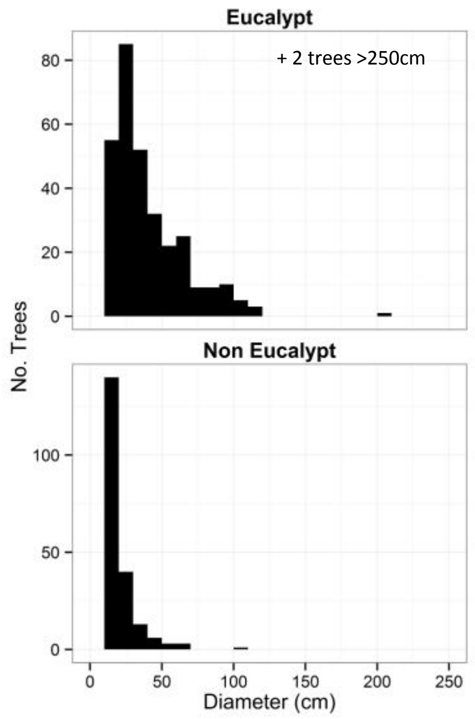

Species	No. Stems	BA (m^2/ha)
Eucalyptus regnans	244	61.7
Eucalyptus delegatensis	66	6.4
Nothofagus cunninghamii	66	4.0
Atherosperma moschatum	37	2.3
Monotoca glauca	51	0.6
Phyllocladus aspleniifolius	27	0.5
Nematolepis squamea	10	0.3
Acacia dealbata	3	0.3
Acacia melanoxylon	2	0.3
Pittosporum bicolor	5	0.2
Olearia argophylla	5	0.2

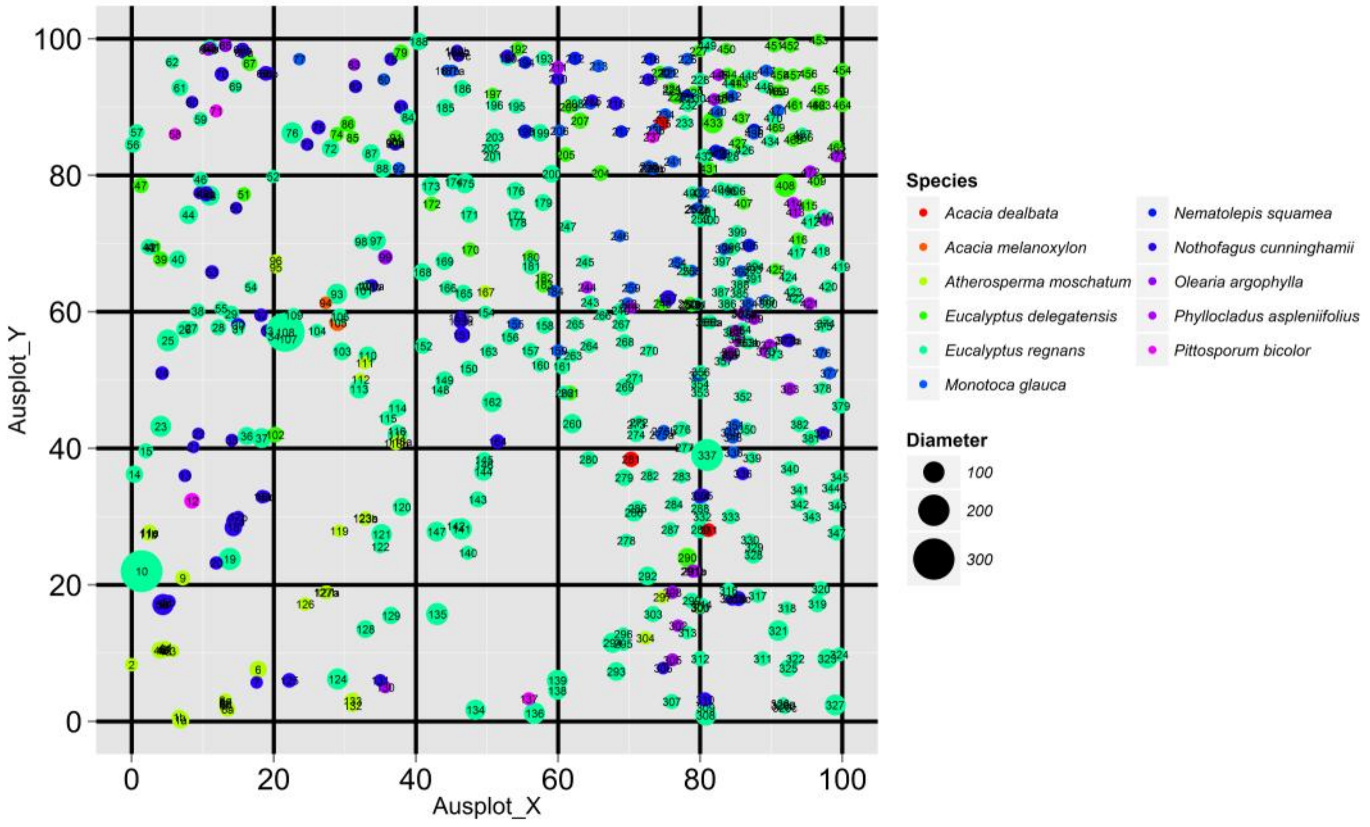

TCFTSR004: Weld

**AusPlot ID**	TCFTSR004	**Elevation**	87m
**AusPlot Name**	Weld	**Aspect**	185°
**State**	Tasmania	**Slope**	Not Recorded^{o}; Gently to Moderate
**Bioregion**	Tas. Southern Ranges	**Landform Element**	Lower Slope
**Location (UTM)**	55 G 479137 5234556	**MAT, MAP**	11.0 ^{o}C, 1228 mm
**Location (Lat/Long)**	-43.0411 146.7435	**Existing Plot Custodian**	NA
**Tenure**	Forest Reserve	**Existing Plot ID**	NA
**Plot Est. Date**	01 May 2013	**Existing Plot Area**	NA
**Plot Size**	1.0ha (100mx100m)	**Existing Plot Census**	NA

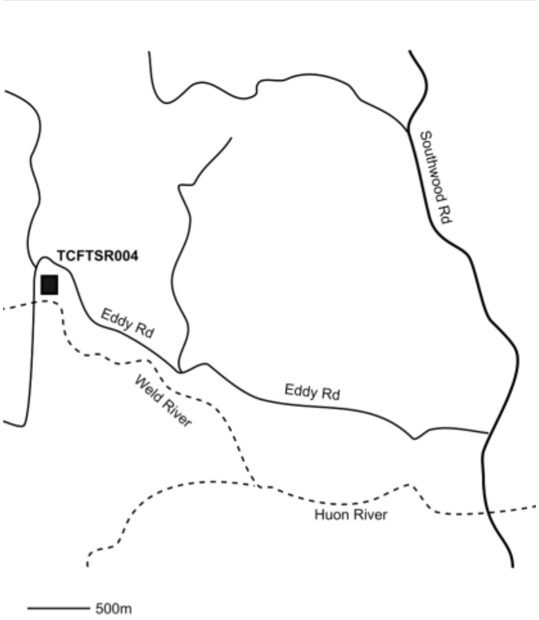

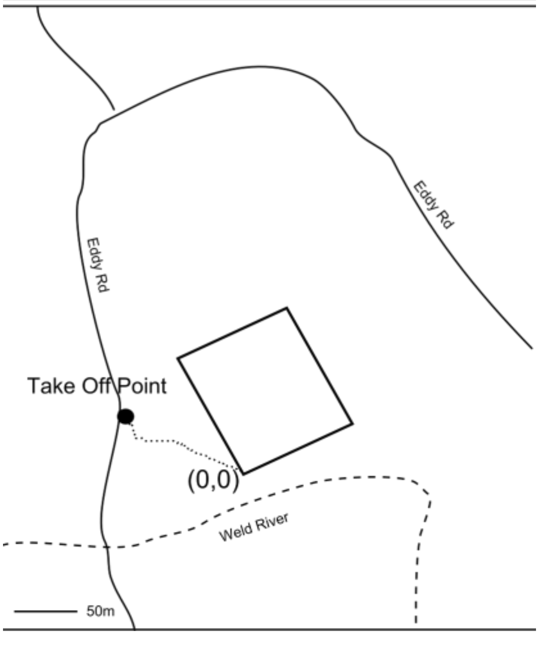

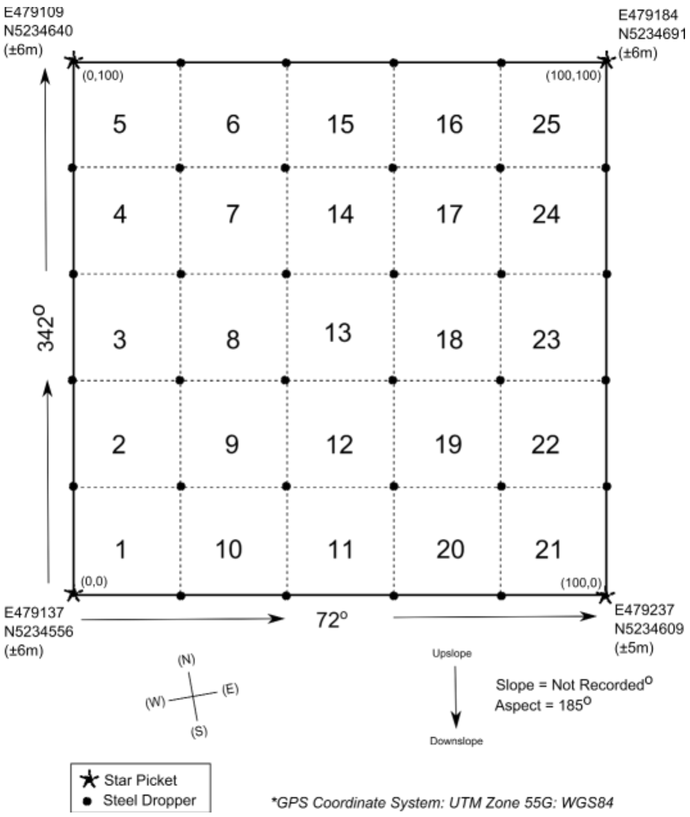

TCFTSR004: Weld

Target Eucalypt Species: *Eucalyptus regnans*

Maximum Tree Height (m) 60m

Target Species Growth Stage: Mature, 1934 regrowth

Understorey: Wet Sclerophyll/Rainforest

High severity fire? Yes, 1934 (known fire event)

Low severity fire? No

Cut stumps? No

Other Disturbance? No

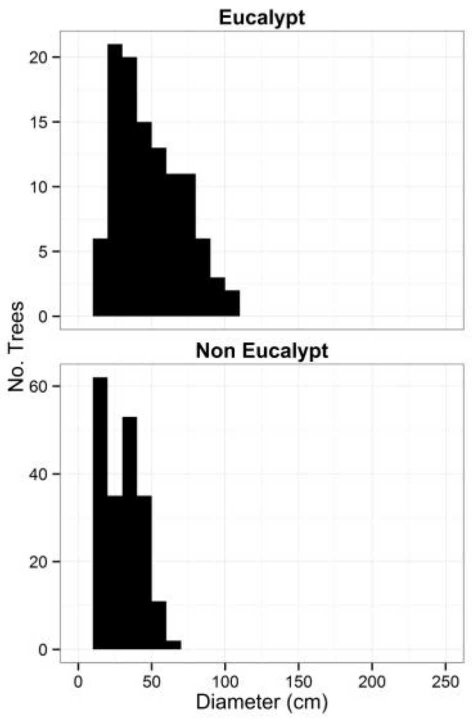

Species	No. Stems	BA (m^2/ha)
Eucalyptus regnans	79	20.5
Acacia melanoxylon	144	15.0
Eucalyptus obliqua	29	3.5
Pomaderris apetala	28	0.6
Nothofagus cunninghamii	5	0.3
Pittosporum bicolor	4	0.2
Atherosperma moschatum	5	0.2
Leptospermum spp	6	0.1
Anopterus glandulosus	4	0.1
Monotoca glauca	2	<0.1

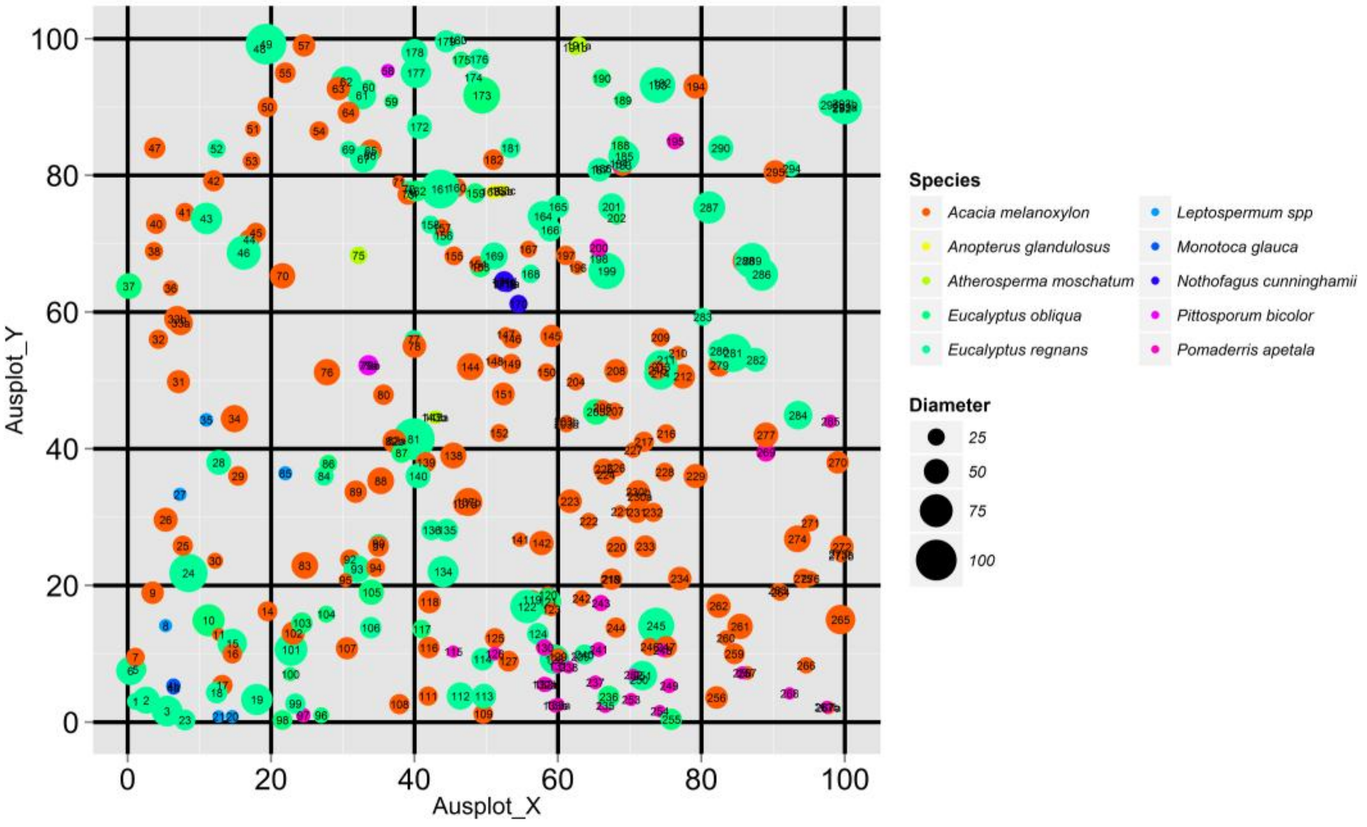

TCFTSR005: Arve/ZigZag

**AusPlot ID**	TCFTSR005	**Elevation**	284m
**AusPlot Name**	Arve/ZigZag	**Aspect**	310°
**State**	Tasmania	**Slope**	8°; Moderate
**Bioregion**	Tas. Southern Ranges	**Landform Element**	Midslope
**Location (UTM)**	55 G 479360 5227846	**MAT, MAP**	10.2 ^{o}C, 1381 mm
**Location (Lat/Long)**	-43.1028 146.7472	**Existing Plot Custodian**	Forestry Tasmania
**Tenure**	Research Reserve	**Existing Plot ID**	Wildfire Chronosequence Plot WCP09 /898N
**Plot Est. Date**	14 May 2013	**Existing Plot Area**	0.25ha (50x50m)
**Plot Size**	1.0ha (100mx100m)	**Existing Plot Census**	2007

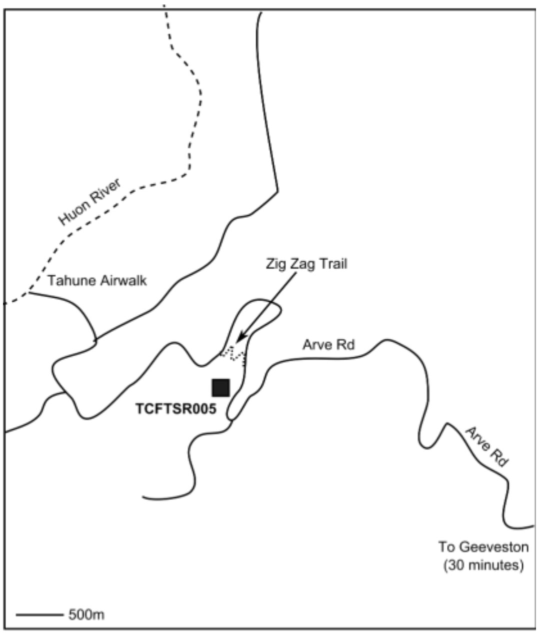

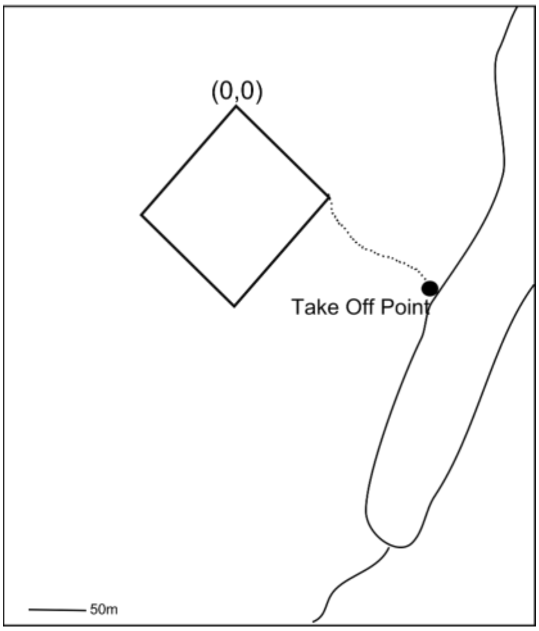

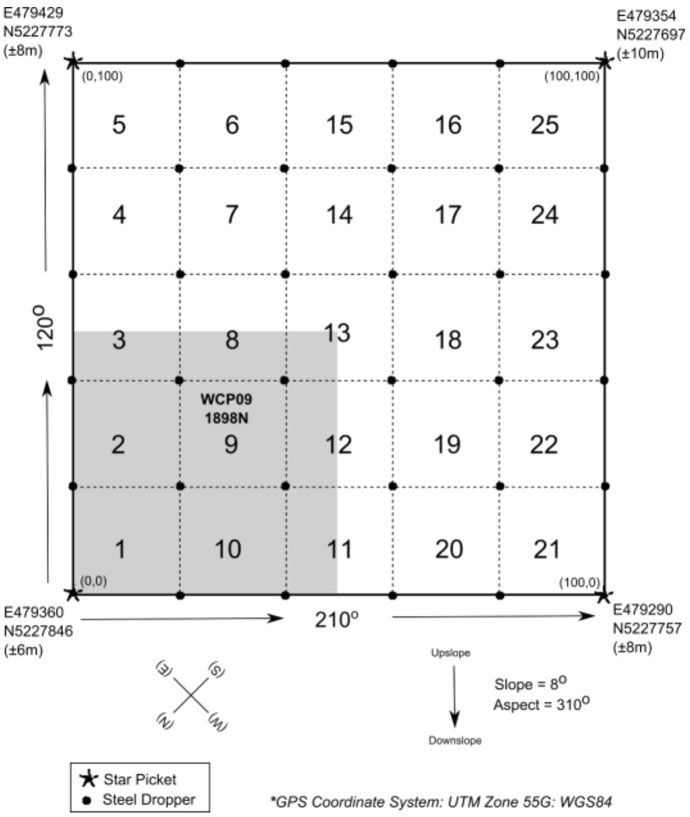

TCFTSR005: Arve/ZigZag

**Target Eucalypt Species:**	*Eucalyptus obliqua*	**High severity fire?**	Yes, 1898 (known fire event)
**Maximum Tree Height (m)**	66m	**Low severity fire?**	No
**Target Species Growth Stage:**	Mature, 1898 regrowth	**Cut stumps?**	No
**Understorey:**	Rainforest	**Other Disturbance?**	No

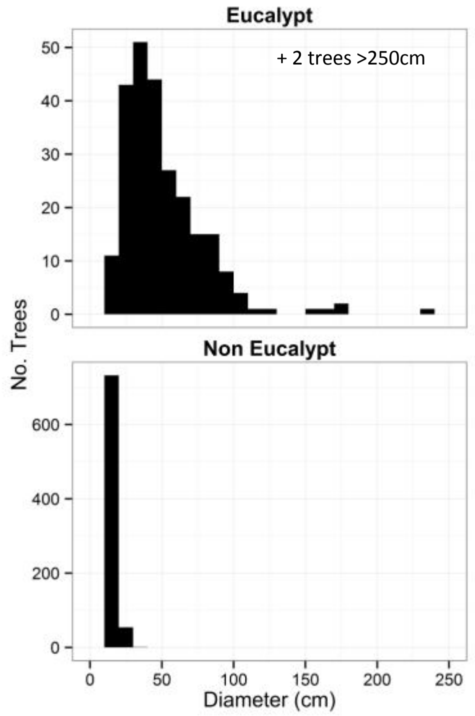

Species	No. Stems	BA (m^2/ha)
Eucalyptus obliqua	235	67.3
Pomaderris apetala	502	7.1
Nematolepis squamea	269	5.8
Eucalyptus regnans	14	3.3
Olearia argophylla	8	0.2
Acacia dealbata	1	0.1
Atherosperma moschatum	5	0.1
Pittosporum bicolor	1	<0.1
Acacia verticillata	1	<0.1

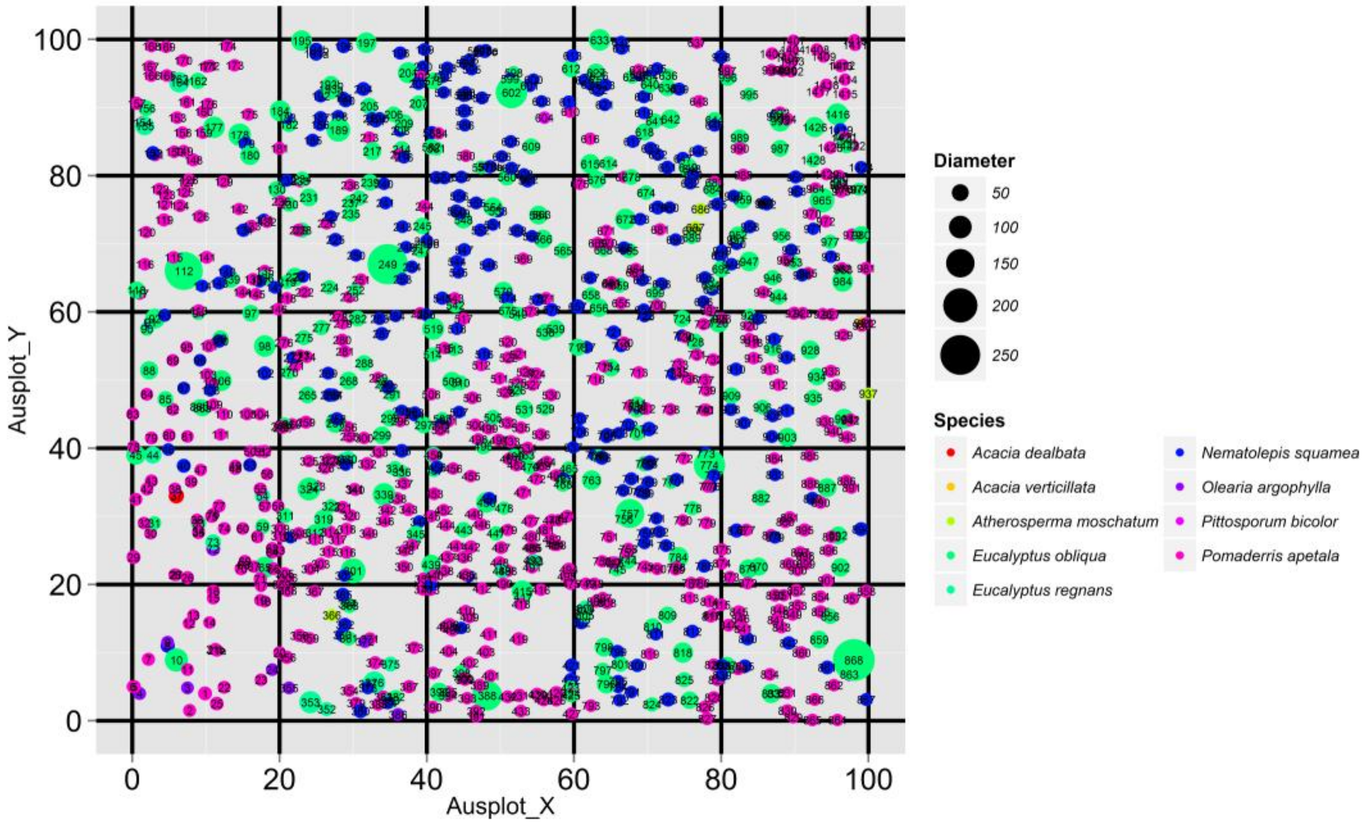

TCFTSR006: Mt Field

**AusPlot ID**	TCFTSR006	**Elevation**	843m
**AusPlot Name**	Mt Field	**Aspect**	180°
**State**	Tasmania	**Slope**	Not Recorded; Moderate
**Bioregion**	Tas. Southern Ranges	**Landform Element**	Midslope
**Location (UTM)**	55 G 471267 5274331	**MAT, MAP**	6.6 ^{o}C, 1309 mm
**Location (Lat/Long)**	-42.6829 146.6492	**Existing Plot Custodian**	NA
**Tenure**	National Park	**Existing Plot ID**	NA
**Plot Est. Date**	1 December 2014	**Existing Plot Area**	NA
**Plot Size**	1.0ha (100mx100m)	**Existing Plot Census**	NA

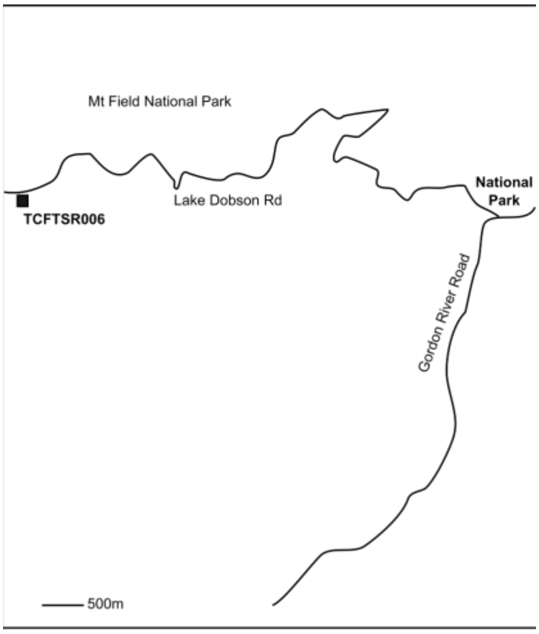

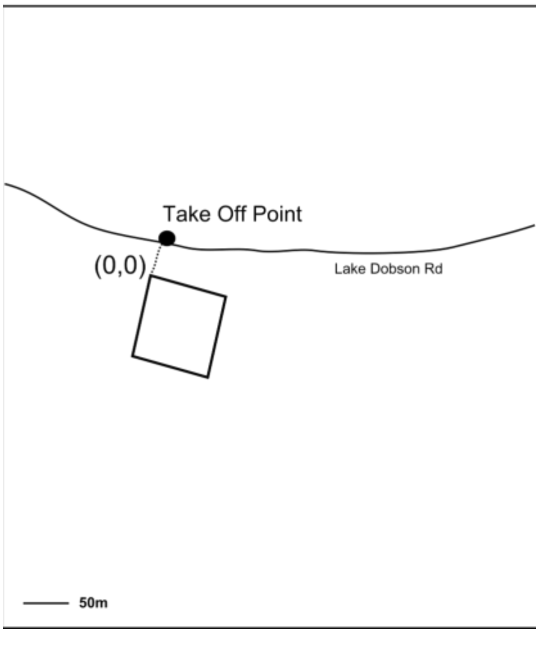

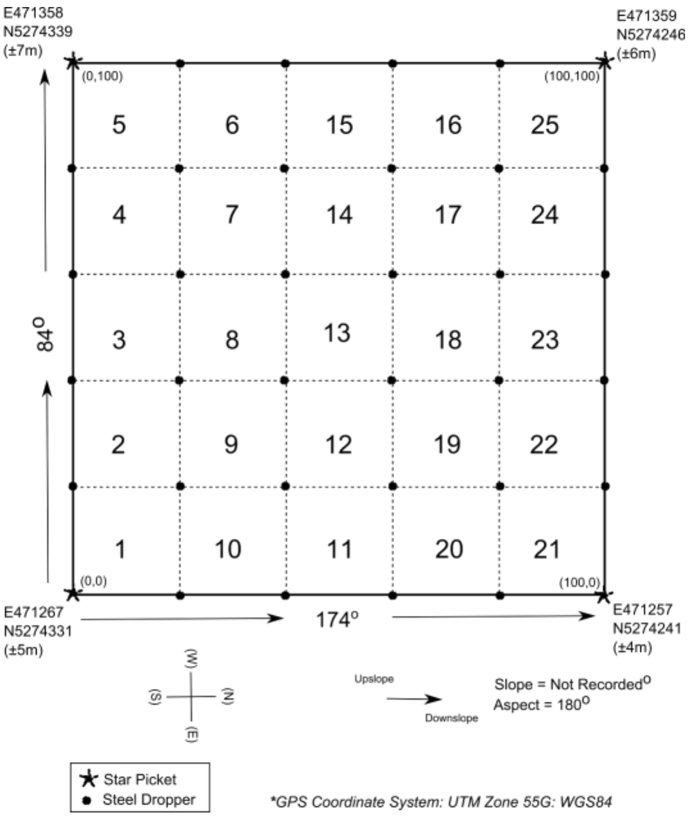

TCFTSR006: Mt Field

**Target Eucalypt Species:**	*Eucalyptus delegatensis*	**High severity fire?**	Unknown, possible stand replacing pre-1900
**Maximum Tree Height (m)**	51m	**Low severity fire?**	No
**Target Species Growth Stage:**	Mature	**Cut stumps?**	Yes, 4 stumps.
**Understorey:**	Rainforest	**Other Disturbance?**	Snig track to east of plot

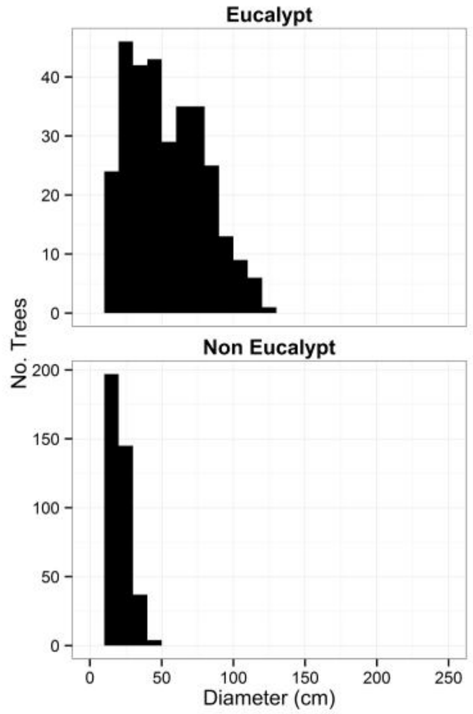

Species	No. Stems	BA (m^2/ha)
Eucalyptus delegatensis	113	52.5
Eucalyptus coccifera	55	20.2
Eucalyptus subcrenulata	107	9.4
Phyllocladus aspleniifolius	183	6.7
Leptospermum lanigerum	103	5.7
Eucalyptus urnigera	34	3.1
Atherosperma moschatum	37	0.7
Pittosporum bicolor	37	0.5
Nematolepis squamea	8	0.1
Ozothamnus antennaria	7	0.1
Nothofagus cunninghamii	5	0.1
Coprosma nitida	2	<0.1
Hakea lissosperma	1	<0.1

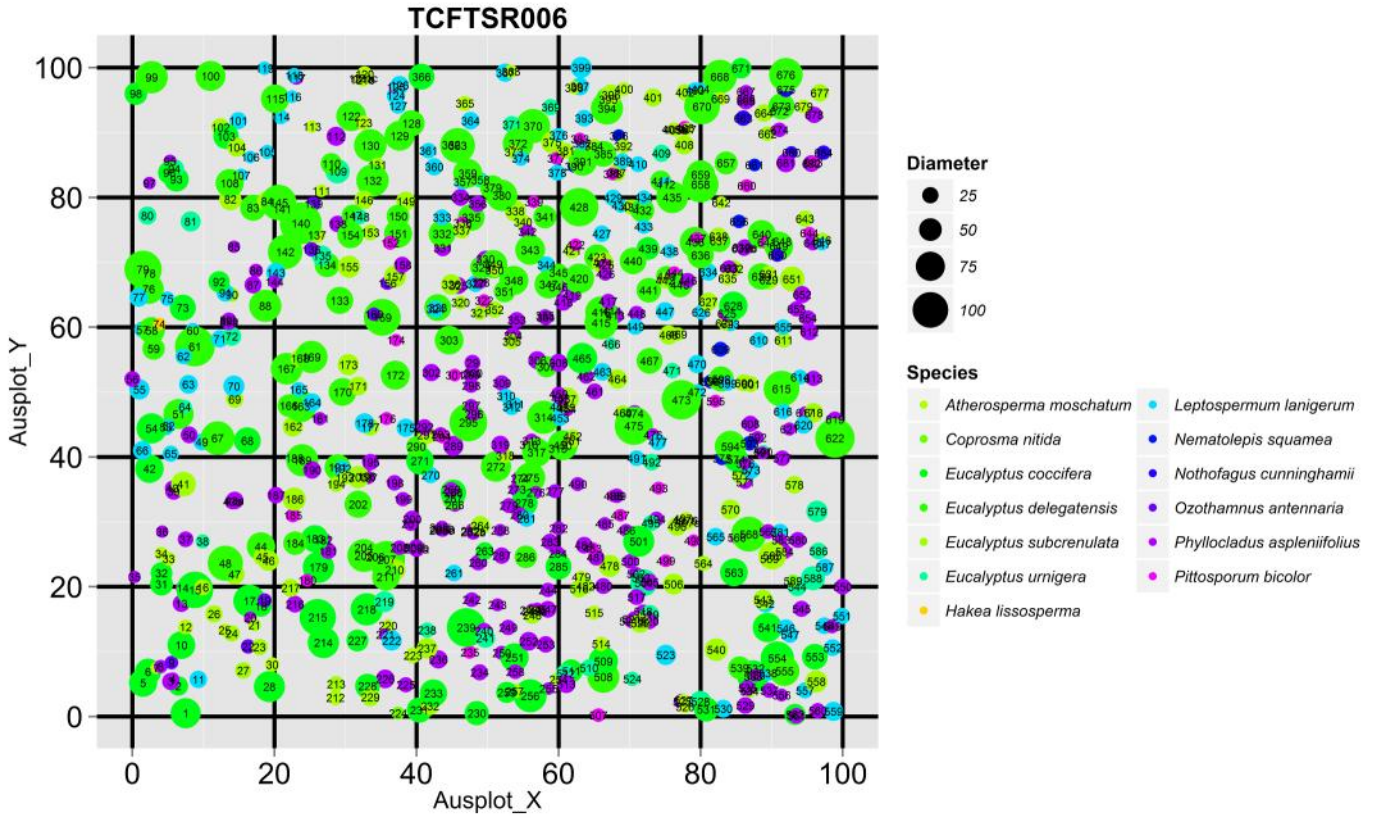

TCFKIN001: Black River

**AusPlot ID**	TCFKIN001	**Elevation**	49m
**AusPlot Name**	Black River	**Aspect**	180°
**State**	Tasmania	**Slope**	Not Recorded; Gentle
**Bioregion**	King	**Landform Element**	Ridge and Upper Slope
**Location (UTM)**	55 G 355571 5470581	**MAT, MAP**	12.4 ^{o}C, 1139 mm
**Location (Lat/Long)**	–40.9525 145.2852	**Existing Plot Custodian**	Forestry Tasmania
**Tenure**	TFA Future Reserve	**Existing Plot ID**	Permanent Inventory Plot 955
**Plot Est. Date**	03 November 2014	**Existing Plot Area**	0.20ha (100x20m)
**Plot Size**	1.0ha (100mx100m)	**Existing Plot Census**	1972, 1976, 1987, 1998

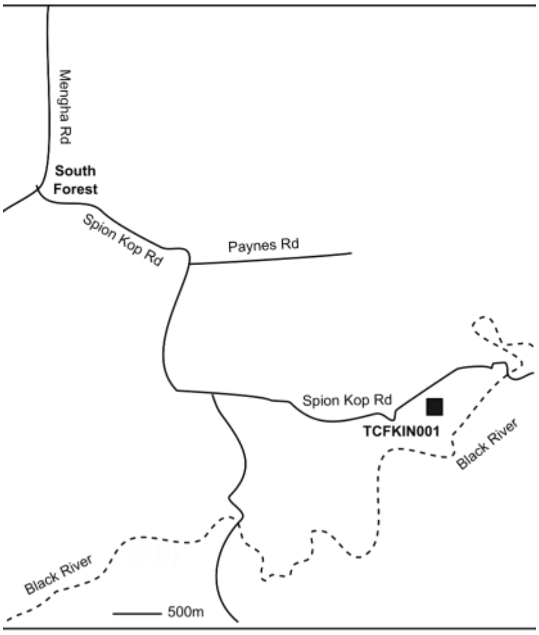

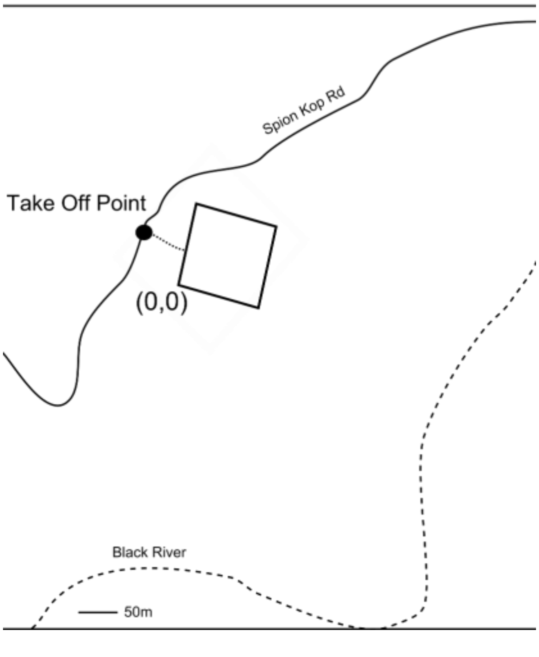

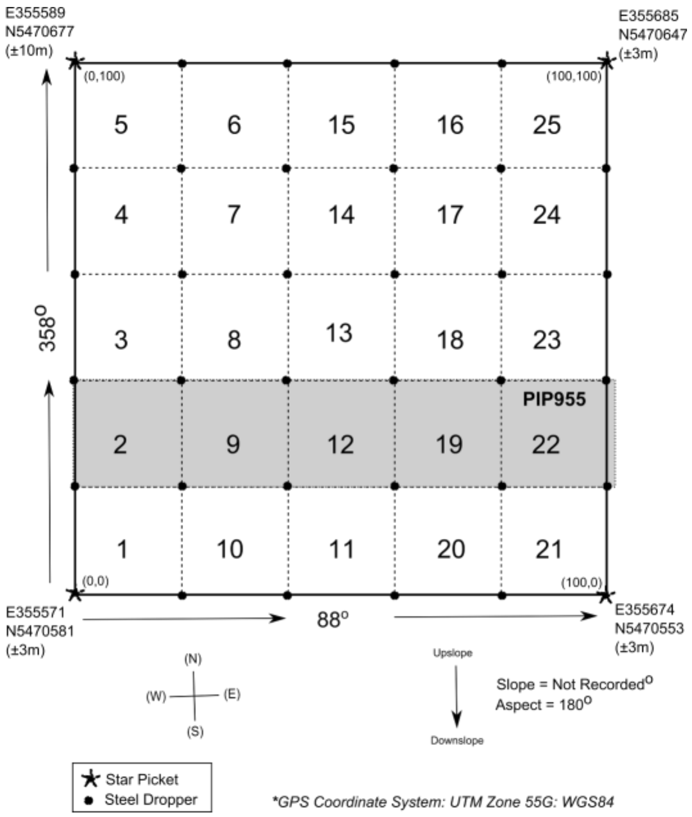

TCFKIN001: Black River

**Target Eucalypt Species:**	*Eucalyptus obliqua*	**High severity fire?**	Yes, 1926 (known fire event, from PIP955)
**Maximum Tree Height (m)**	57m	**Low severity fire?**	No
**Target Species Growth Stage:**	Mature, 1926 regrowth	**Cut stumps?**	No
**Understorey:**	Wet Sclerophyll	**Other Disturbance?**	No

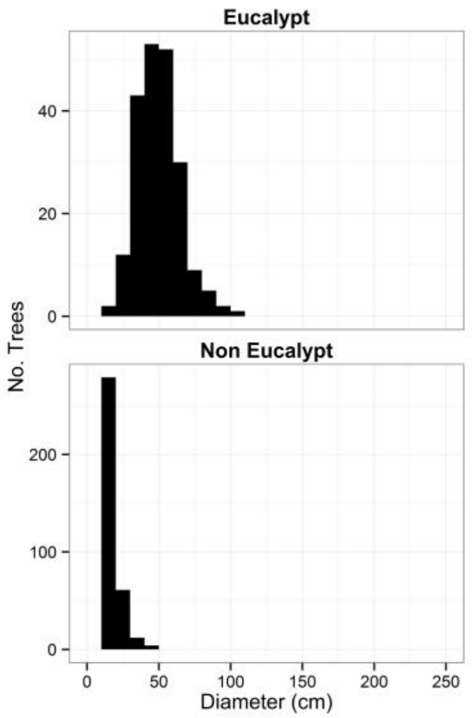

Species	No. Stems	BA (m^2/ha)
Eucalyptus obliqua	148	30.7
Eucalyptus ovata	61	11.8
Nematolepis squamea	128	3.3
Monotoca glauca	121	2.2
Acacia mucronata	37	1.9
Leptospermum scoparium	49	0.9
Acacia melanoxylon	5	0.6
Zieria arborescens	16	0.2

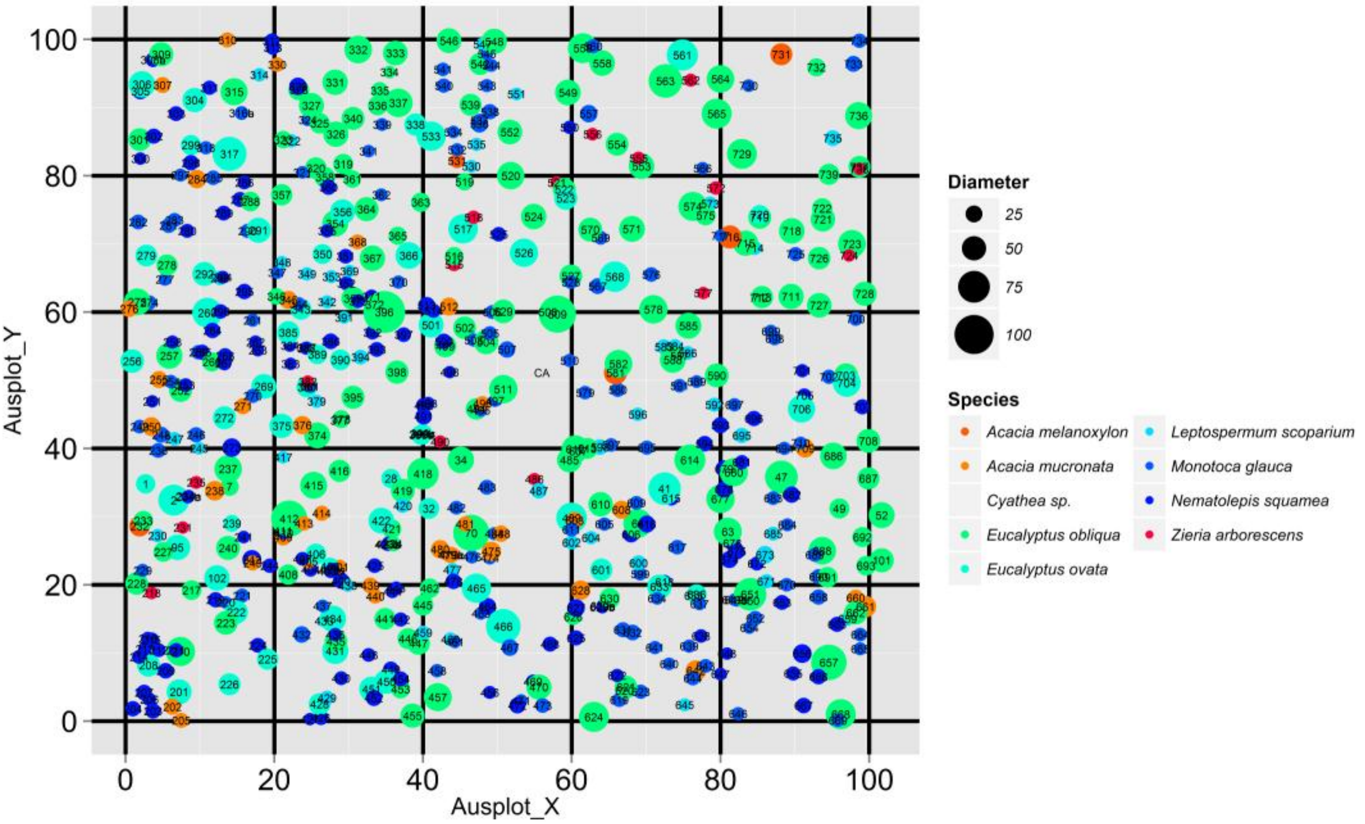

TCFKIN002: Bond Tier

**AusPlot ID**	TCFKIN002	**Elevation**	54m
**AusPlot Name**	Bond Tiers	**Aspect**	240°
**State**	Tasmania	**Slope**	Not Recorded; Gentle
**Bioregion**	King	**Landform Element**	Lower Slope
**Location (UTM)**	55 G 318377 5464267	**MAT, MAP**	12.3 ^{o}C, 1275 mm
**Location (Lat/Long)**	-40.9526 144.8420	**Existing Plot Custodian**	NA
**Tenure**	Regional Reserve	**Existing Plot ID**	NA
**Plot Est. Date**	17 November 2014	**Existing Plot Area**	NA
**Plot Size**	1.0ha (100mx100m)	**Existing Plot Census**	NA

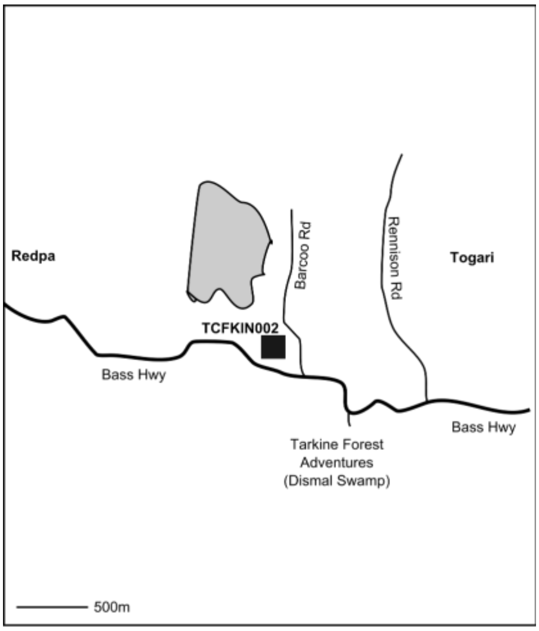

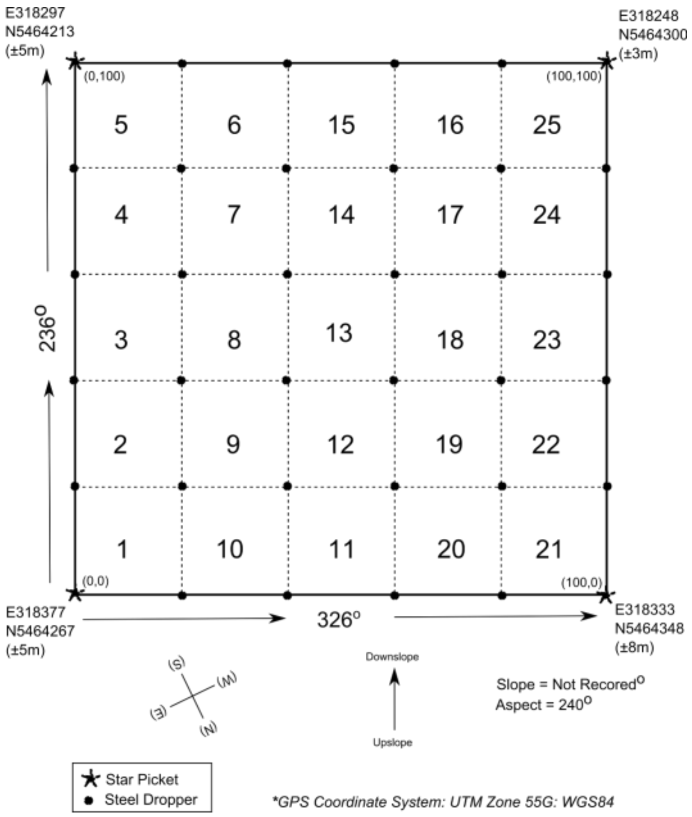

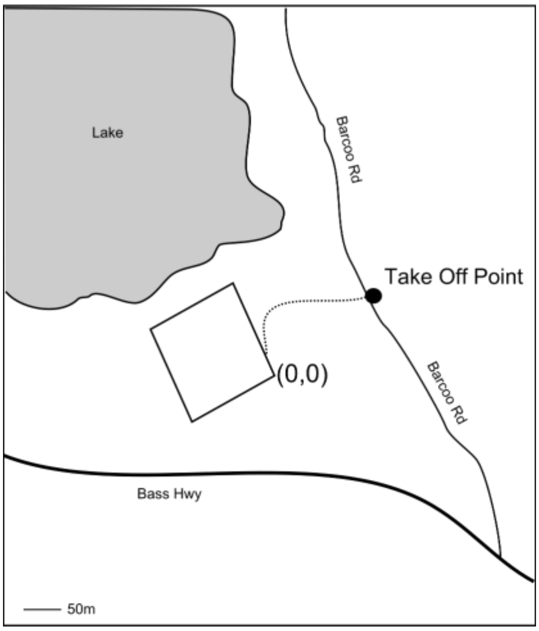

TCFKIN002: Bond Tier

**Target Eucalypt Species:**	*Eucalyptus obliqua*	**High severity fire?**	Unknown, possibly 1920's
**Maximum Tree Height (m)**	64m	**Low severity fire?**	No
**Target Species Growth Stage:**	Mature, (1920's?)	**Cut stumps?**	No
**Understorey:**	Wet Sclerophyll	**Other Disturbance?**	No

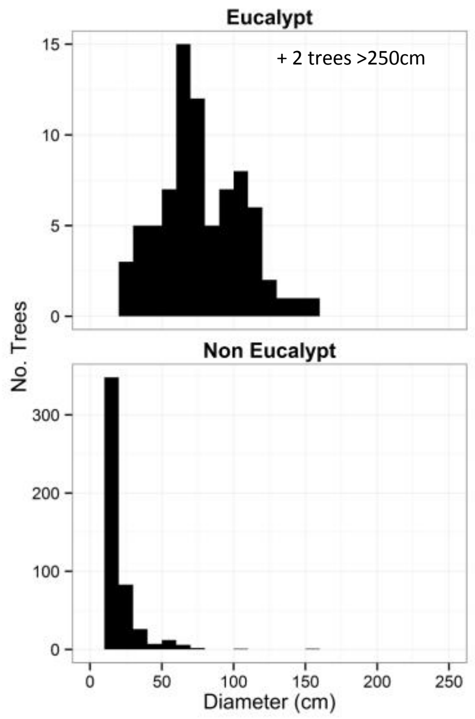

Species	No. Stems	BA (m^2/ha)
Eucalyptus obliqua	80	50.0
Acacia melanoxylon	47	7.5
Nothofagus cunninghamii	71	5.6
Pomaderris apetala	179	4.3
Olearia argophylla	115	2.2
Pittosporum bicolor	62	1.3
Nematolepis squamea	8	0.2
Monotoca glauca	1	<0.1
Atherosperma moschatum	2	<0.1
Leptospermum scoparium	1	<0.1

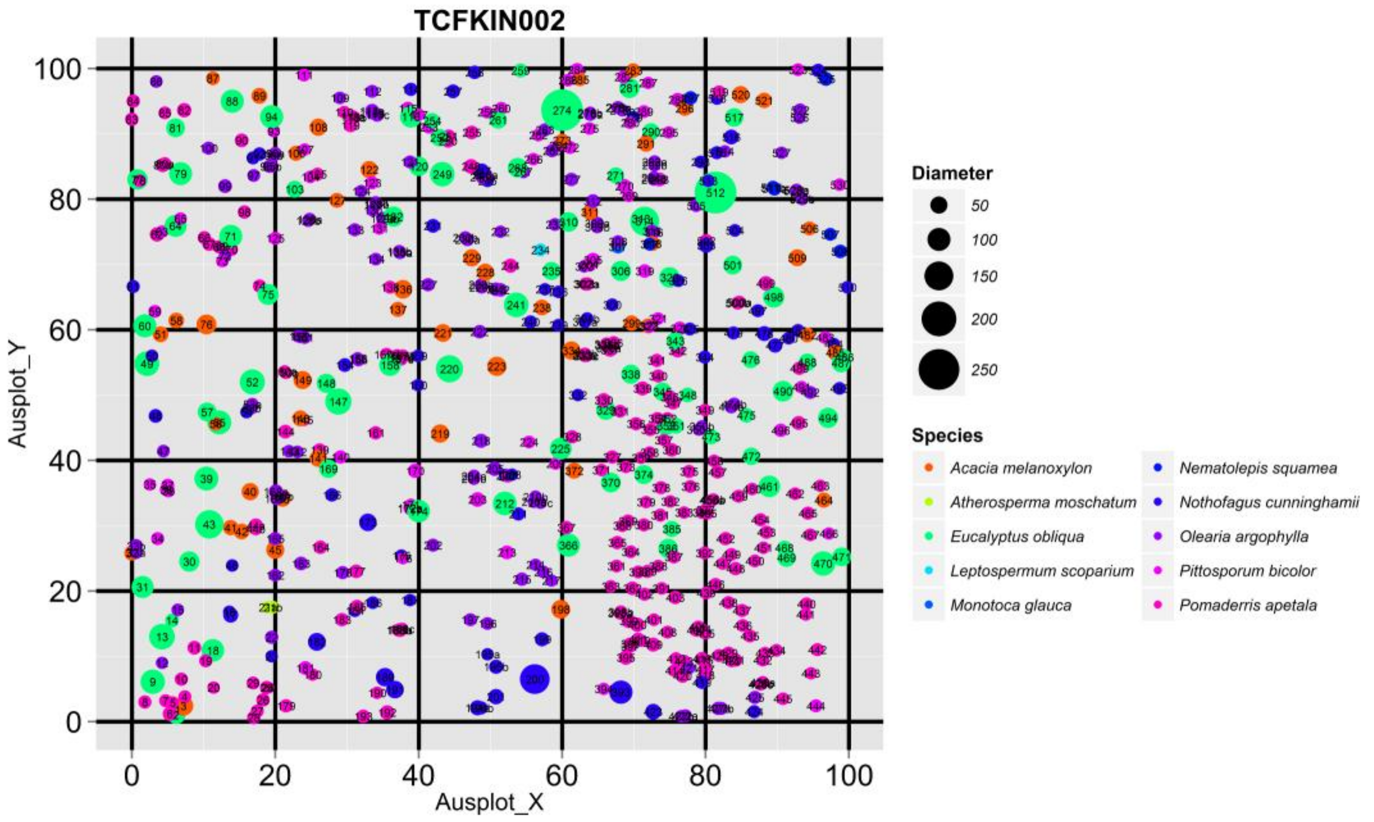

TCFBEL001: Mt Maurice

**AusPlot ID**	TCFBEL001	**Elevation**	752m
**AusPlot Name**	Mt Maurice	**Aspect**	324°
**State**	Tasmania	**Slope**	Not Recorded; Moderate
**Bioregion**	Ben Lomond	**Landform Element**	Midslope
**Location (UTM)**	55 G 545065 5426540	**MAT, MAP**	8.7 ^{o}C, 1371 mm
**Location (Lat/Long)**	-41.3113 147.5383	**Existing Plot Custodian**	NA
**Tenure**	TFA Future Reserve	**Existing Plot ID**	NA
**Plot Est. Date**	8 December 2014	**Existing Plot Area**	NA
**Plot Size**	1.0ha (100mx100m)	**Existing Plot Census**	NA

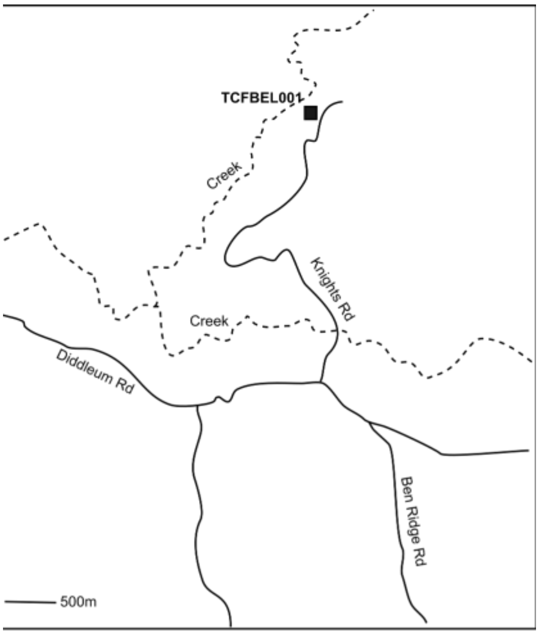

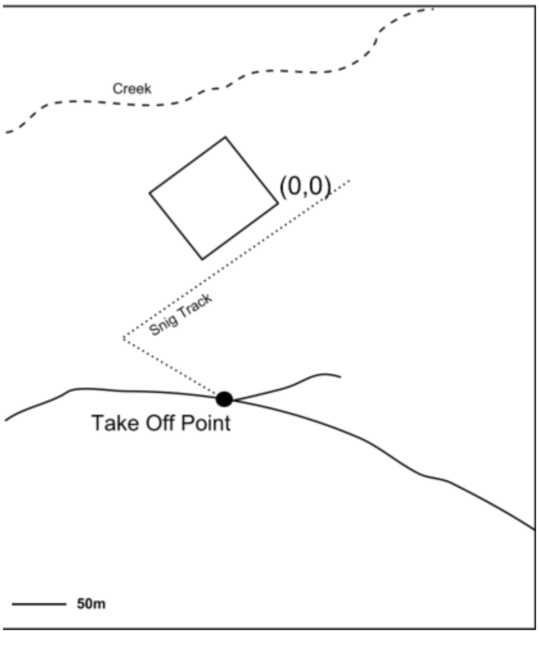

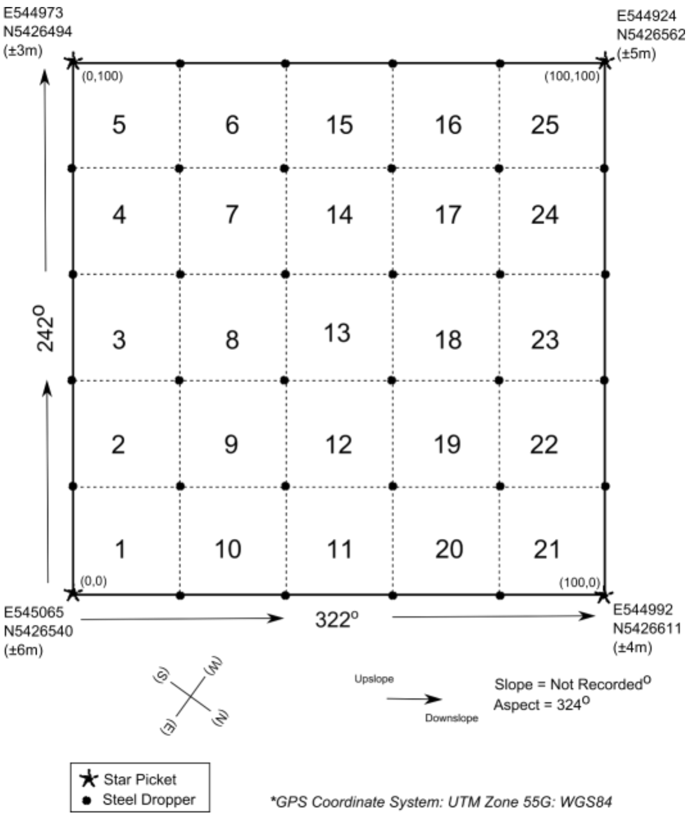

TCFBEL001: Mt Maurice

**Target Eucalypt Species:**	*Eucalyptus delegatensis*	**High severity fire?**	Unknown
**Maximum Tree Height (m)**	51m	**Low severity fire?**	No
**Target Species Growth Stage:**	Mature	**Cut stumps?**	Yes, 28 stumps, 17 Axe, 11 Chainsaw
**Understorey:**	Wet Sclerophyll	**Other Disturbance?**	Snig Tracks

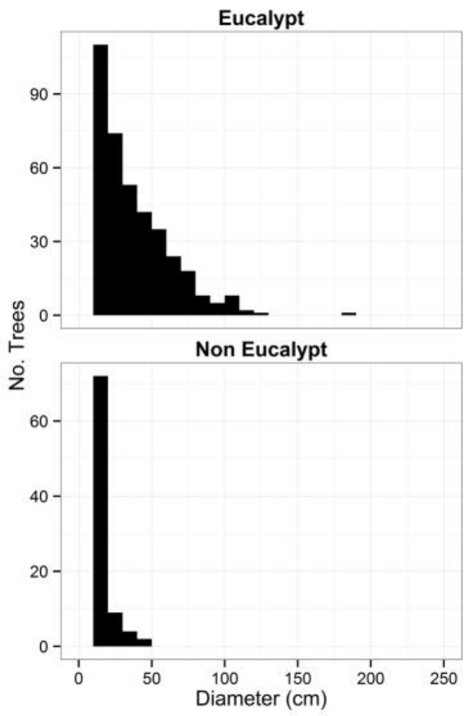

Species	No. Stems	BA (m^2/ha)
Eucalyptus delegatensis	295	53.0
Eucalyptus dalrympleana	86	7.3
Acacia dealbata	32	1.3
Leptospermum lanigerum	34	0.5
Phyllocladus aspleniifolius	5	0.1
Tasmannia lanceolata	6	0.1
Persoonia muelleri	4	<0.1
Monotoca glauca	3	<0.1
Pittosporum bicolor	2	<0.1
Nothofagus cunninghamii	1	<0.1

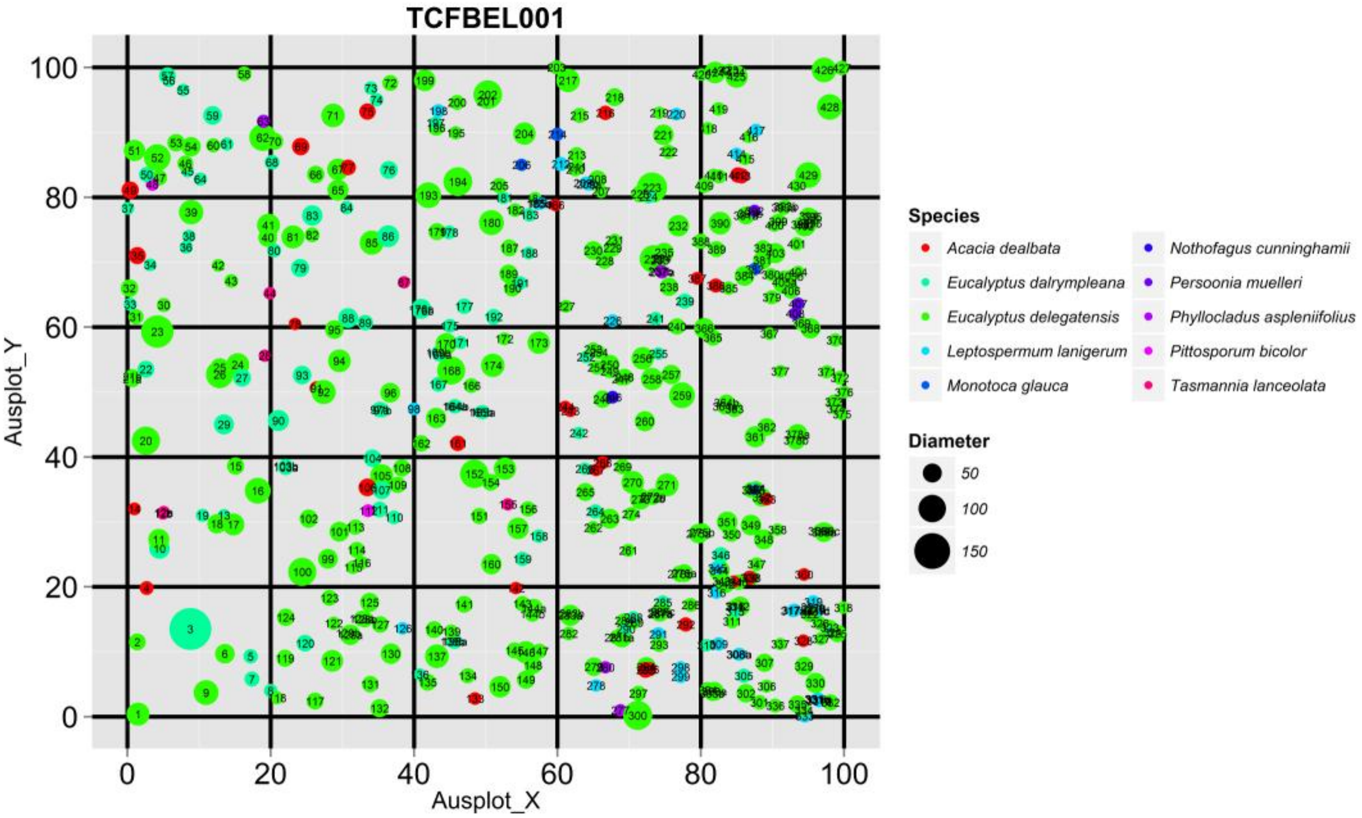

TCFBEL002: Ben Ridge

**AusPlot ID**	TCFBEL002	**Elevation**	910m
**AusPlot Name**	Ben Ridge	**Aspect**	223°
**State**	Tasmania	**Slope**	Not Recorded; Gentle to Steep
**Bioregion**	Ben Lomond	**Landform Element**	Ridge and Upper Slope
**Location (UTM)**	55 G 550390 5420282	**MAT, MAP**	8.6 ^{o}C, 1350 mm
**Location (Lat/Long)**	–41.3673 147.6025	**Existing Plot Custodian**	NA
**Tenure**	TFA Future Reserve	**Existing Plot ID**	NA
**Plot Est. Date**	8 January 2015	**Existing Plot Area**	NA
**Plot Size**	1.0ha (100mx100m)	**Existing Plot Census**	NA

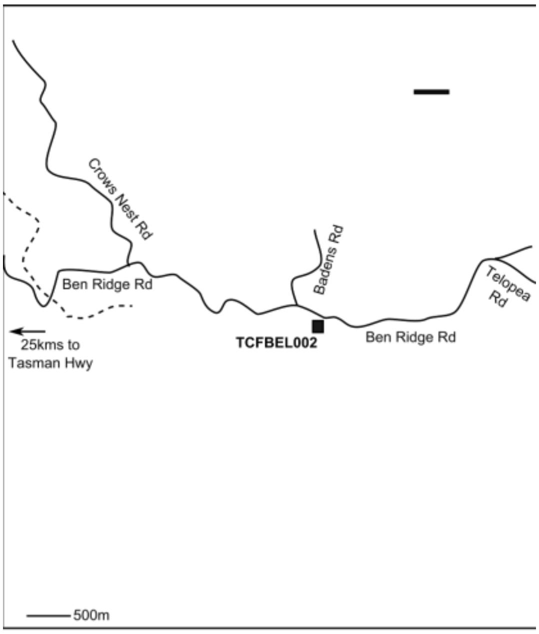

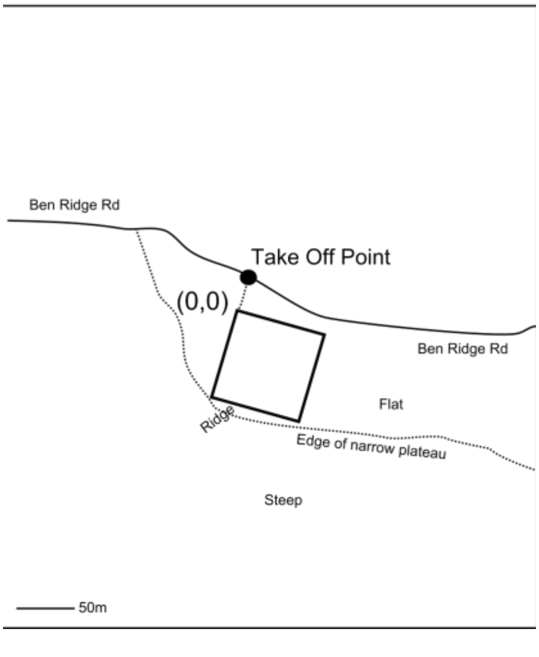

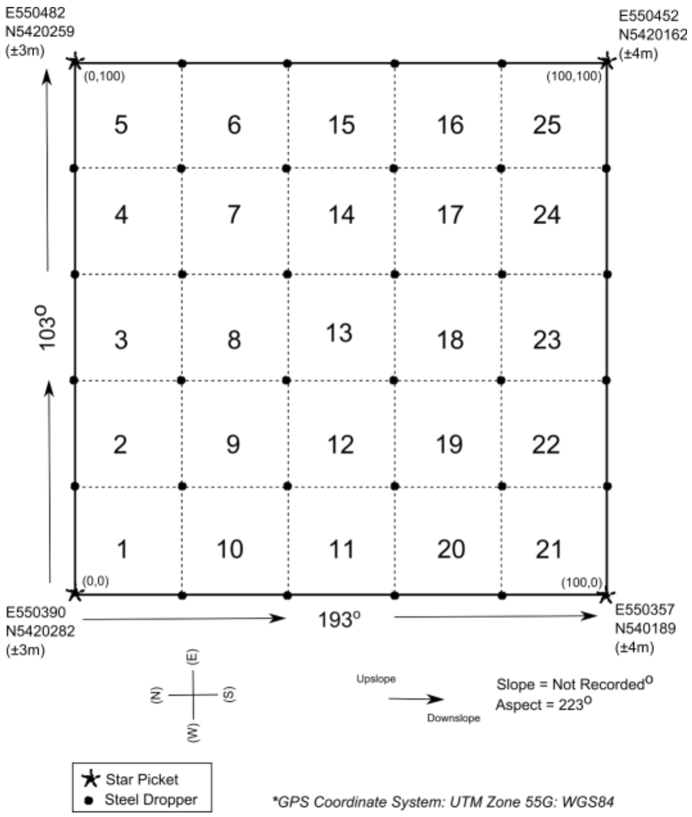

TCFBEL002: Ben Ridge

**Target Eucalypt Species:**	*Eucalyptus delegatensis*	**High severity fire?**	Unknown
**Maximum Tree Height (m)**	47m	**Low severity fire?**	No
**Target Species Growth Stage:**	Mature	**Cut stumps?**	Yes, 25 Stumps, 12 Chainsaw, 13 Axe Cut
**Understorey:**	Wet Sclerophyll	**Other Disturbance?**	Snig Tracks

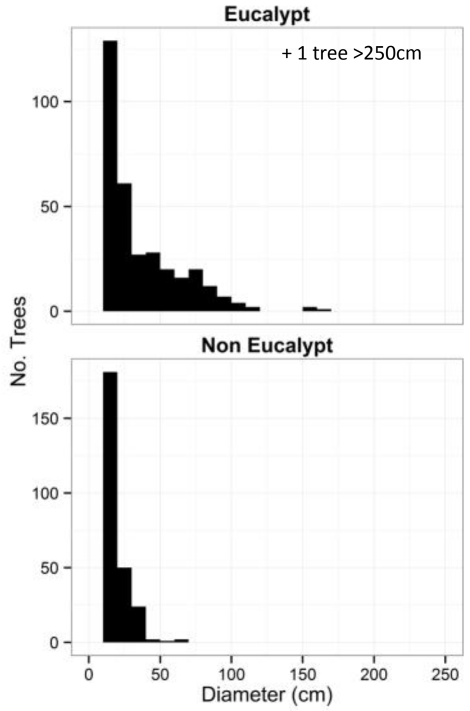

Species	No. Stems	BA (m^2/ha)
Eucalyptus delegatensis	330	58.1
Acacia dealbata	114	6.2
Tasmannia lanceolata	138	1.9
Pittosporum bicolor	2	0.1
Persoonia muelleri	4	<0.1
Pomaderris apetala	2	<0.1

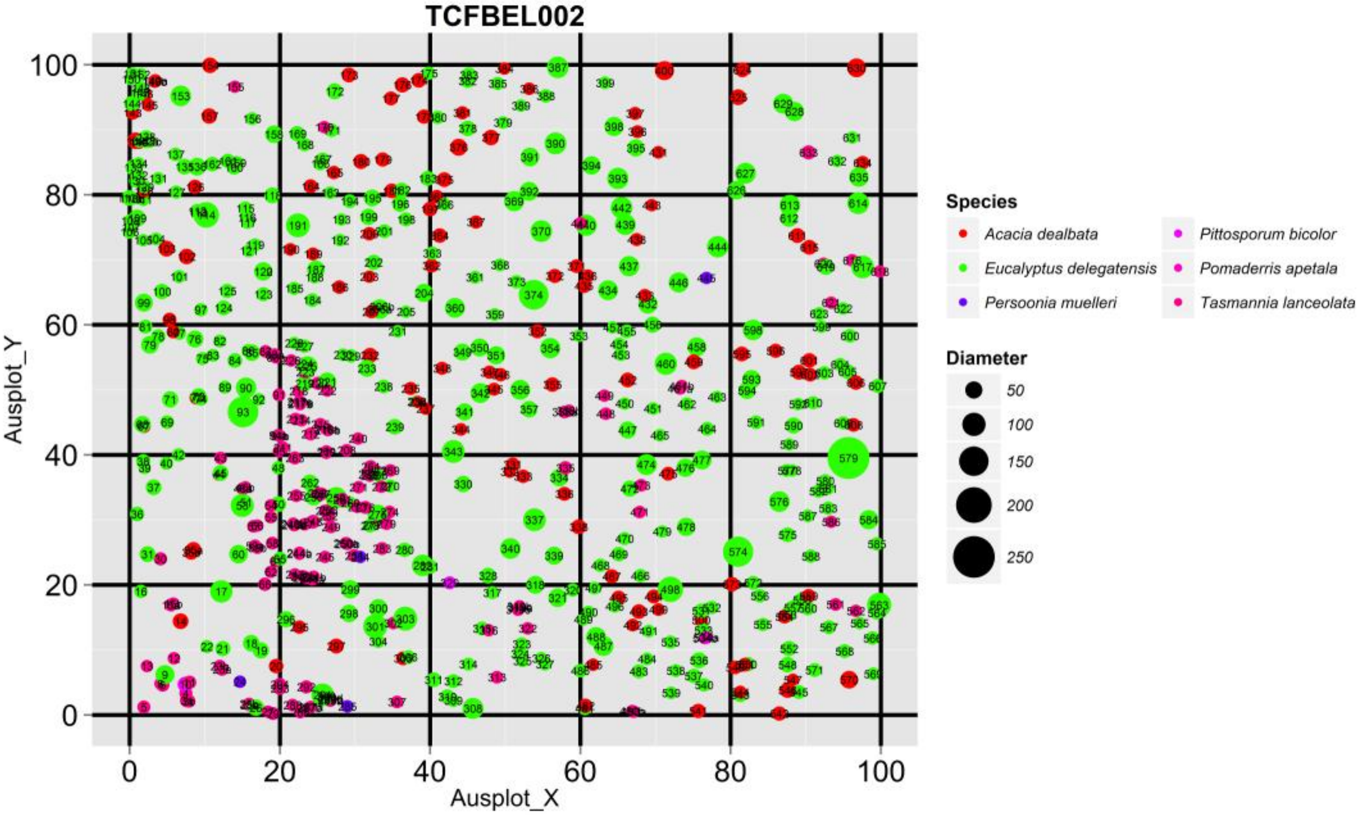

TCFTNS001: Flowerdale

**AusPlot ID**	TCFTNS001	**Elevation**	206m
**AusPlot Name**	Flowerdale	**Aspect**	90°
**State**	Tasmania	**Slope**	Not Recorded^{o}; Flat
**Bioregion**	Tas. Northern Slopes	**Landform Element**	Ridge
**Location (UTM)**	55 G 379490 5455259	**MAT, MAP**	11.4 ^{o}C, 1406 mm
**Location (Lat/Long)**	-41.0449 145.5661	**Existing Plot Custodian**	Forestry Tasmania
**Tenure**	Flowerdale Forest Res.	**Existing Plot ID**	Permanent Inventory Plot 600
**Plot Est. Date**	27 March 2012	**Existing Plot Area**	0.2ha (20mx100m)
**Plot Size**	1.0ha (100mx100m)	**Existing Plot Census**	1970,1974,1985,1997

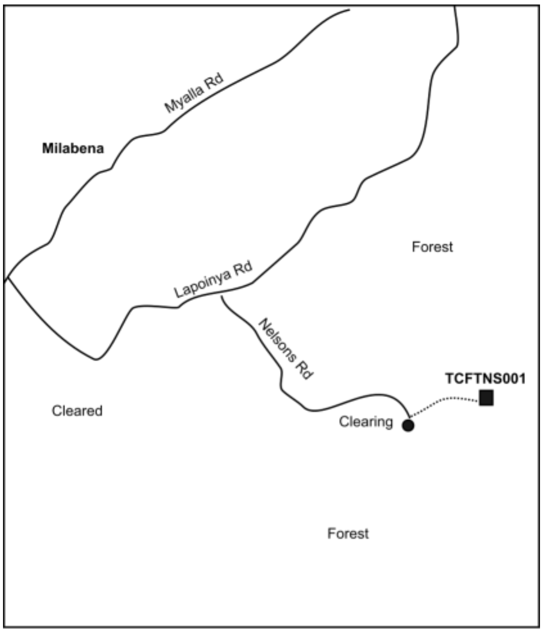

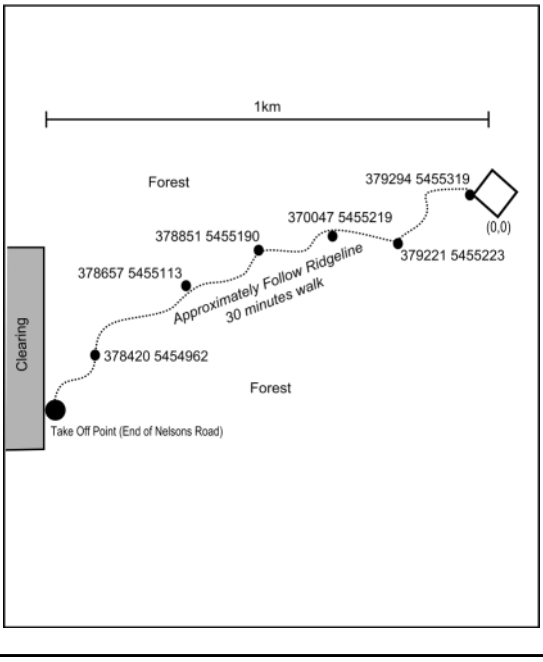

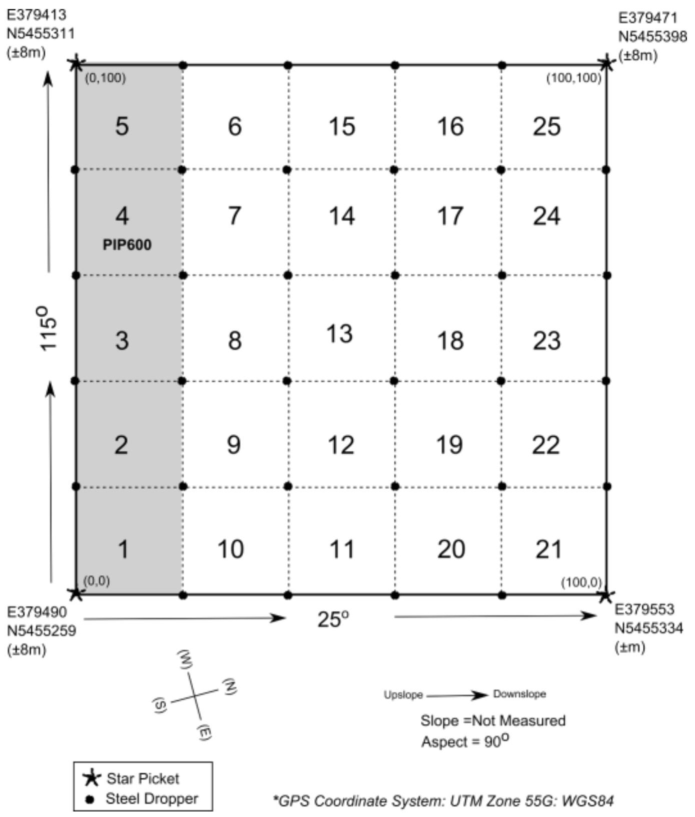

TCFTNS001: Flowerdale

**Target Eucalypt Species:**	*Eucalyptus obliqua*	**High severity fire?**	Yes, approx. 1925 (from PIP600)
**Maximum Tree Height (m)**	59m	**Low severity fire?**	No
**Target Species Growth Stage:**	Mature, 1925 regrowth	**Cut stumps?**	Yes, 4 axe stumps
**Understorey:**	Wet Sclerophyll	**Other Disturbance?**	No

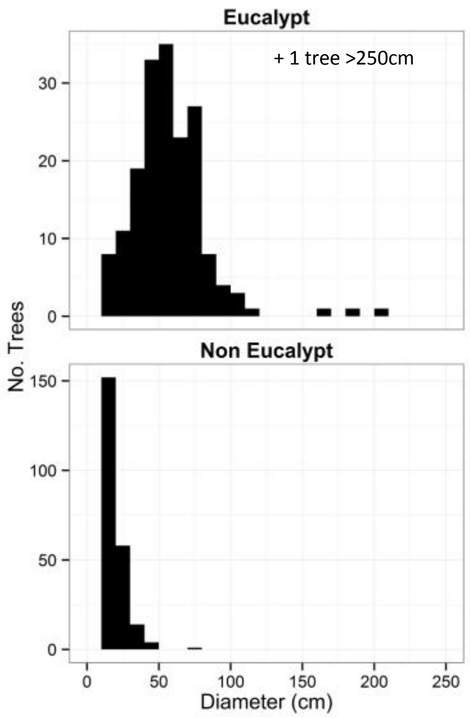

Species	No. Stems	BA (m^2/ha)
Eucalyptus obliqua	176	59.4
Acacia melanoxylon	44	3.3
Nematolepis squamea	92	2.5
Pomaderris apetala	49	1.2
Olearia argophylla	18	0.3
Pittosporum bicolor	11	0.3
Monotoca glauca	12	0.2
Acacia verticillata	2	<0.1
Coprosma spp	1	<0.1

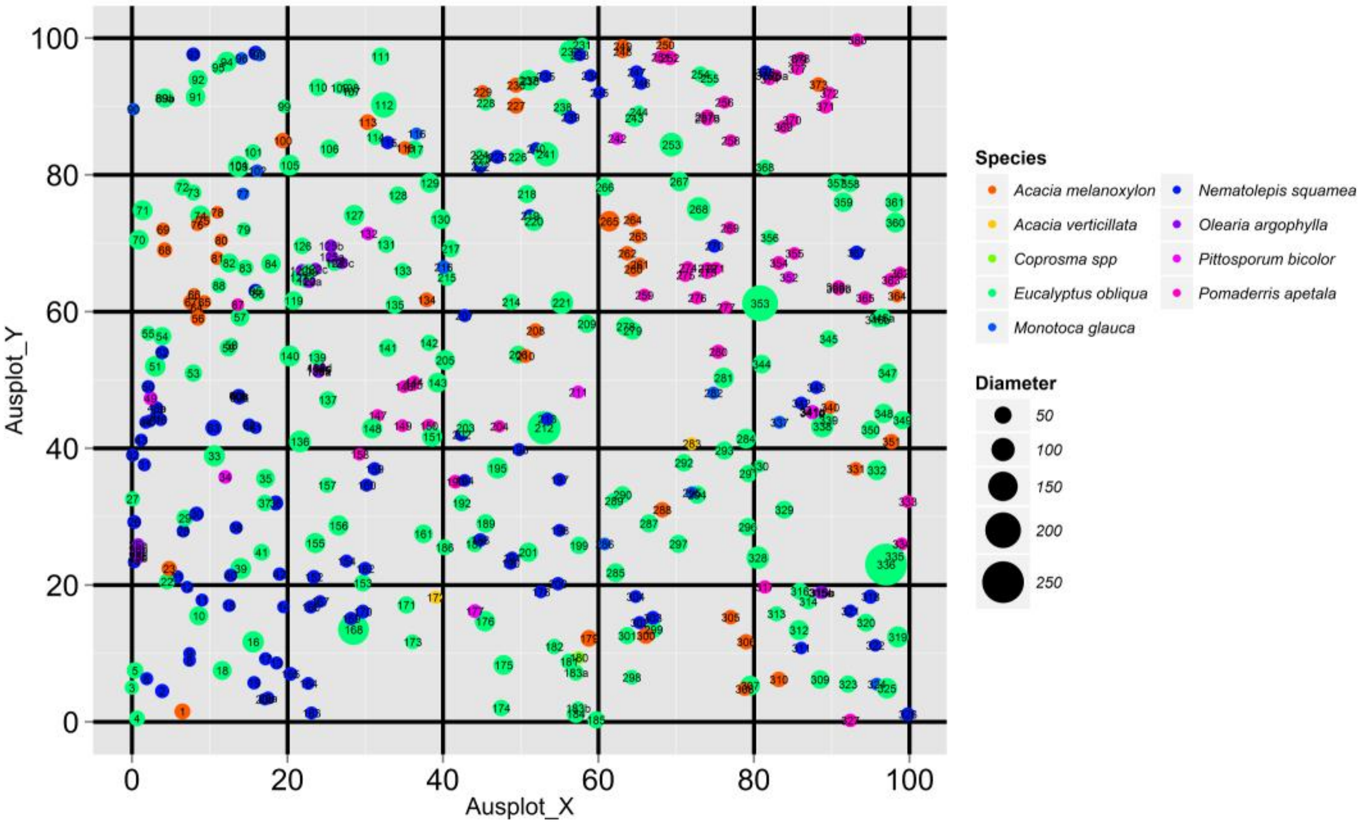

TCFTNS002: Dip River

**AusPlot ID**	TCFTNS002	**Elevation**	247m
**AusPlot Name**	Dip River	**Aspect**	360°
**State**	Tasmania	**Slope**	1°; Flat
**Bioregion**	Tas. Northern Slopes	**Landform Element**	Lower Slopes
**Location (UTM)**	55 G 365934 5456172	**MAT, MAP**	11.2 ^{o}C, 1477 mm
**Location (Lat/Long)**	-41.0345 145.4055	**Existing Plot Custodian**	Forestry Tasmania
**Tenure**	Dip River Forest Res.	**Existing Plot ID**	Permanent Inventory Plot 957
**Plot Est. Date**	19 August 2012	**Existing Plot Area**	0.2ha (20mx100m)
**Plot Size**	1.0ha (100mx100m)	**Existing Plot Census**	1972,1976,1986,1998

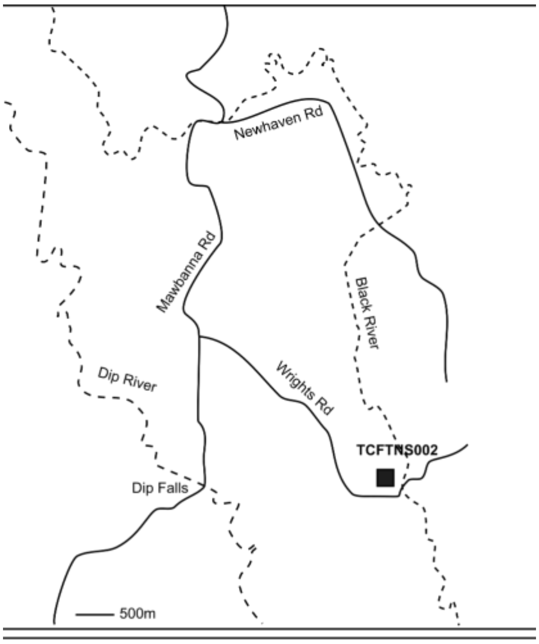

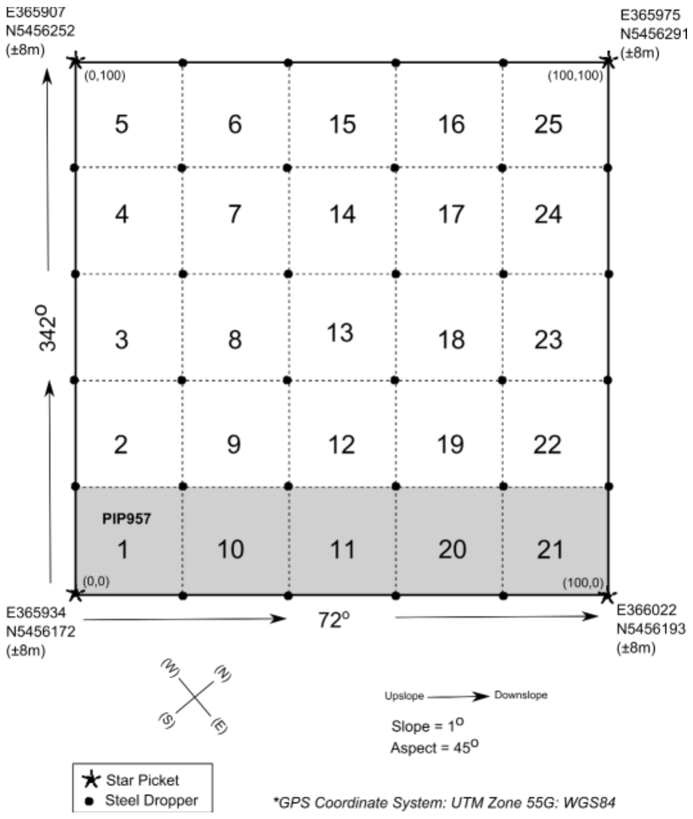

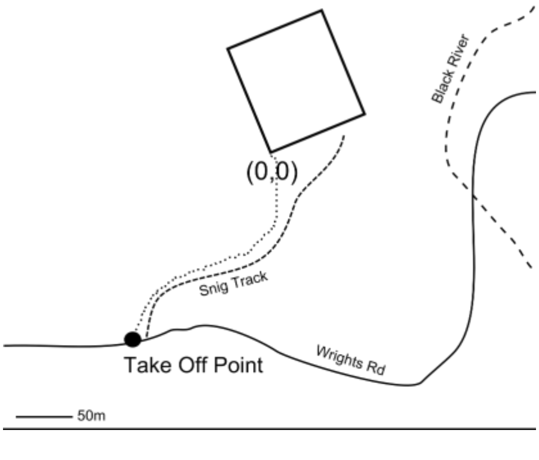

TCFTNS002: Dip River

**Target Eucalypt Species:**	*Eucalyptus obliqua*	**High severity fire?**	Yes, approx. 1923 (from PIP957)
**Maximum Tree Height (m)**	66m	**Low severity fire?**	No
**Target Species Growth Stage:**	Mature, 1923 regrowth	**Cut stumps?**	No
**Understorey:**	Wet Sclerophyll	**Other Disturbance?**	No

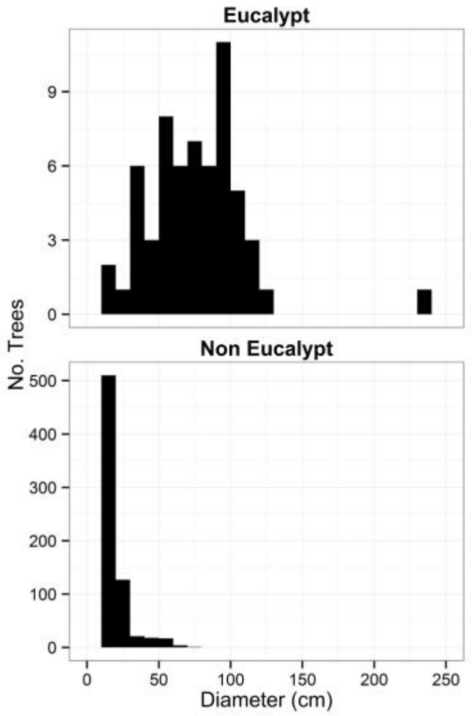

Species	No. Stems	BA (m^2/ha)
Eucalyptus obliqua	60	32.6
Pomaderris apetala	593	13.1
Acacia melanoxylon	60	9.8
Olearia argophylla	26	0.8
Atherosperma moschatum	10	0.6
Nothofagus cunninghamii	7	0.3
Pittosporum bicolor	2	0.1

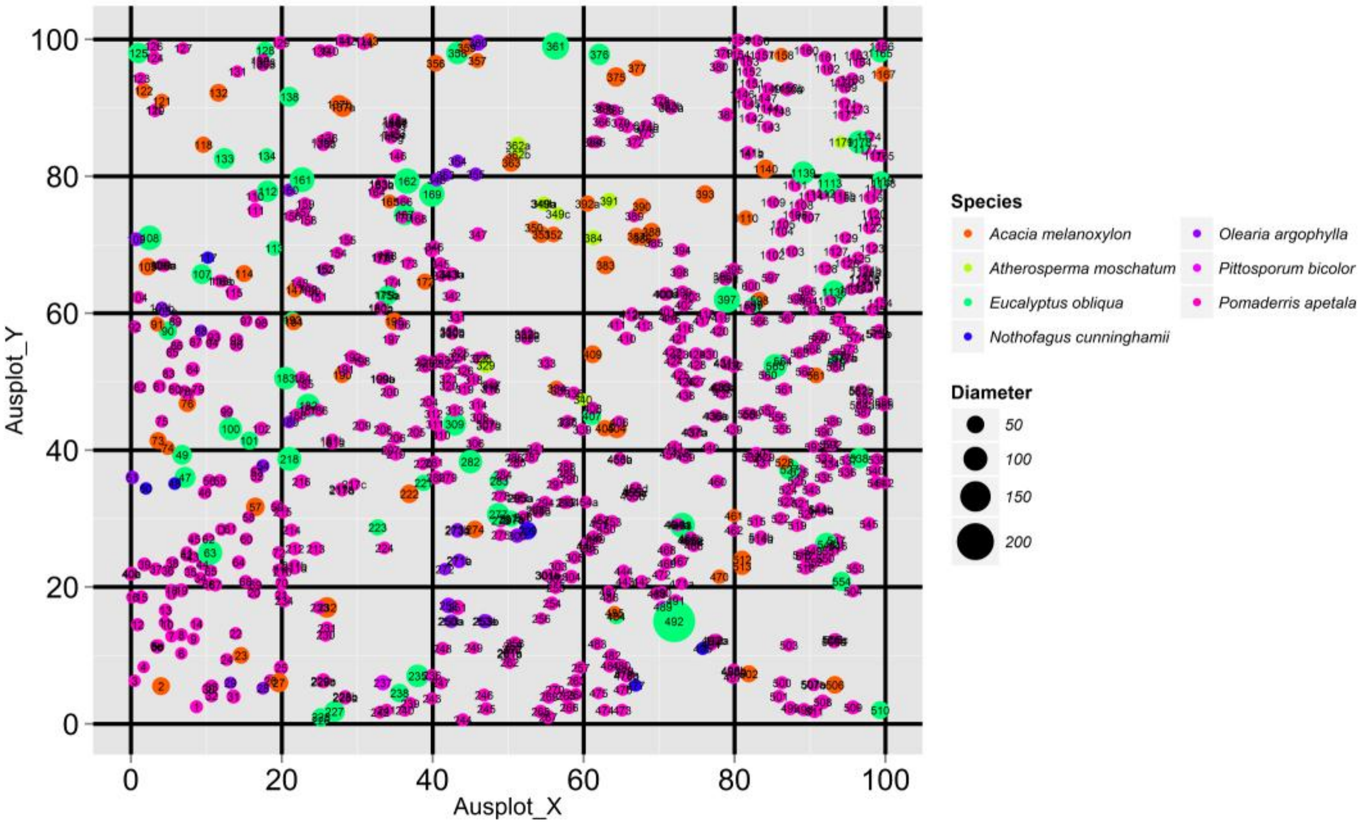

TCFTNS003: McKenzie

**AusPlot ID**	TCFTNS003	**Elevation**	790m
**AusPlot Name**	McKenzie	**Aspect**	180°
**State**	Tasmania	**Slope**	6°
**Bioregion**	Tas. Northern Slopes	**Landform Element**	Gentle to moderate slope
**Location (UTM)**	55 G 438224 5391043	**MAT, MAP**	7.5 ^{o}C, 1723 mm
**Location (Lat/Long)**	-41.6299 146.2583	**Existing Plot Custodian**	NA
**Tenure**	TFA Future Reserve	**Existing Plot ID**	NA
**Plot Est. Date**	20 January 2015	**Existing Plot Area**	NA
**Plot Size**	1.0ha (100mx100m)	**Existing Plot Census**	NA

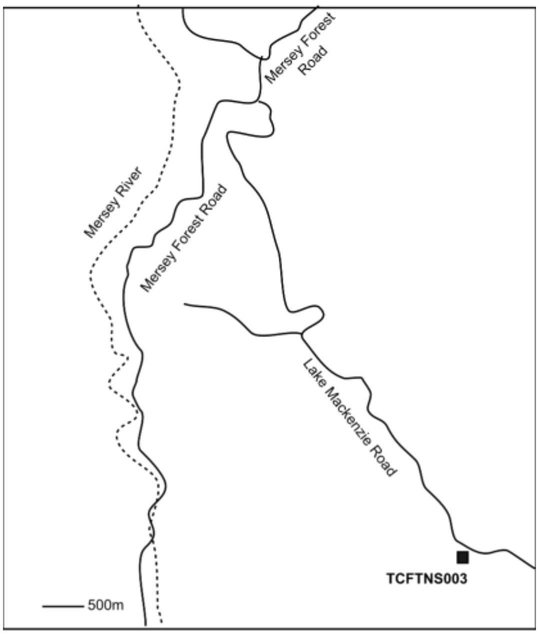

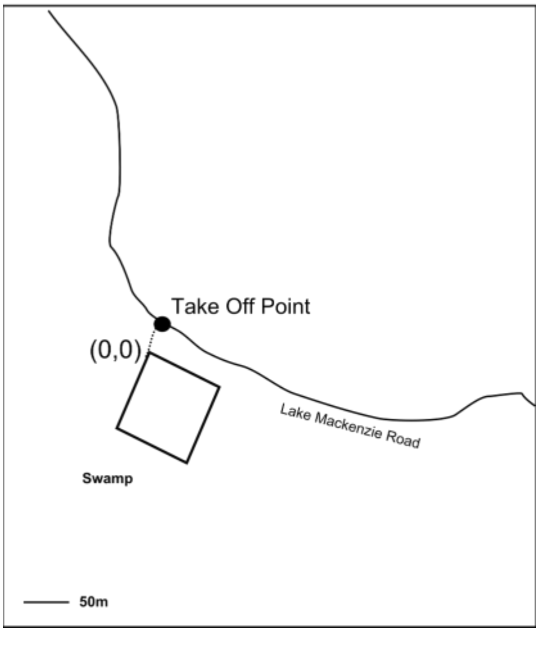

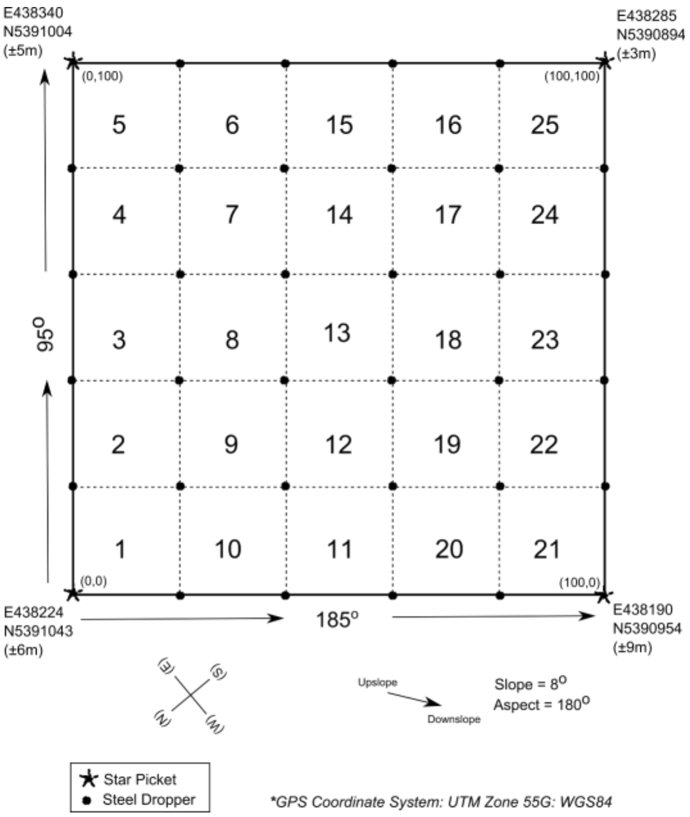

TCFTNS003: McKenzie

**Target Eucalypt Species:**	*Eucalyptus delegatensis*	**High severity fire?**	Unknown
**Maximum Tree Height (m)**	51m	**Low severity fire?**	No
**Target Species Growth Stage:**	Mature	**Cut stumps?**	Yes, 7 stumps, Axe Cut (>100cm DBH)
**Understorey:**	Rainforest/Sclerophyll	**Other Disturbance?**	Very old snig track

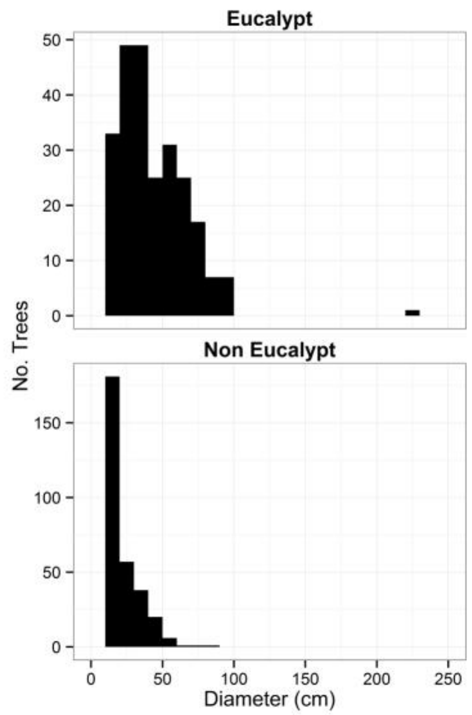

Species	No. Stems	BA (m^2/ha)
Eucalyptus delegatensis	241	46.9
Acacia dealbata	73	8.3
Leptospermum lanigerum	113	3.4
Nematolepis squamea	72	1.4
Nothofagus cunninghamii	17	1.0
Eucalyptus dalrympleana	3	0.7
Atherosperma moschatum	11	0.6
Pittosporum bicolor	11	0.2
Olearia argophylla	3	0.1
Monotoca glauca	4	<0.1
Phyllocladus aspleniifolius	1	<0.1

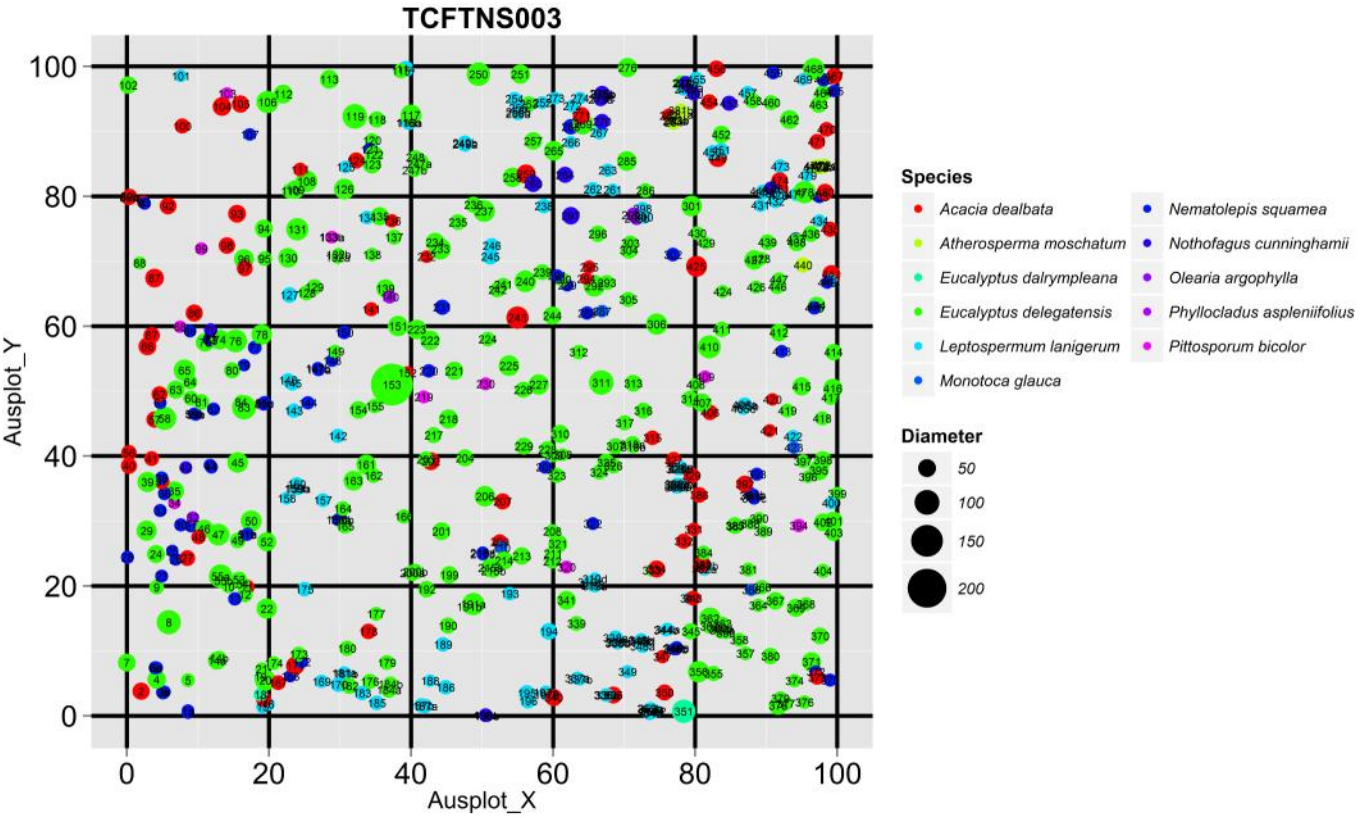

TCFTNS004: Caveside

**AusPlot ID**	TCFTNS004	**Elevation**	691m
**AusPlot Name**	Caveside	**Aspect**	295°
**State**	Tasmania	**Slope**	5°
**Bioregion**	Tas. Northern Slopes	**Landform Element**	Gentle Slope
**Location (UTM)**	55 G 458482 5386756	**MAT, MAP**	7.4 ^{o}C, 1368 mm
**Location (Lat/Long)**	-41.6698 146.5012	**Existing Plot Custodian**	NA
**Tenure**	TFA Future Reserve	**Existing Plot ID**	NA
**Plot Est. Date**	03 February 2015	**Existing Plot Area**	NA
**Plot Size**	1.0ha (100mx100m)	**Existing Plot Census**	NA

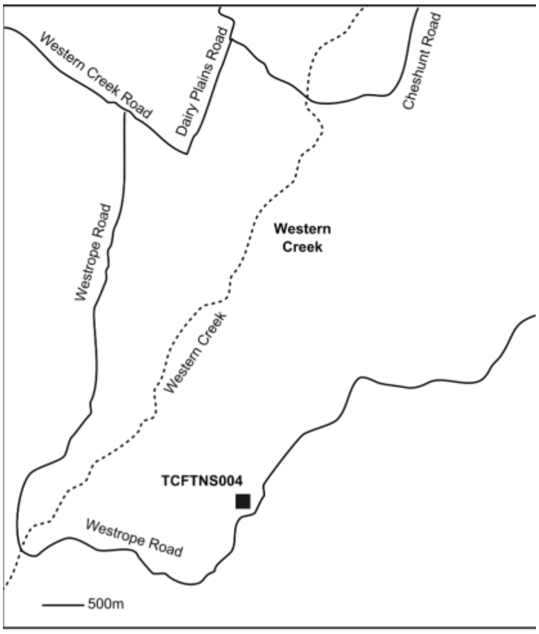

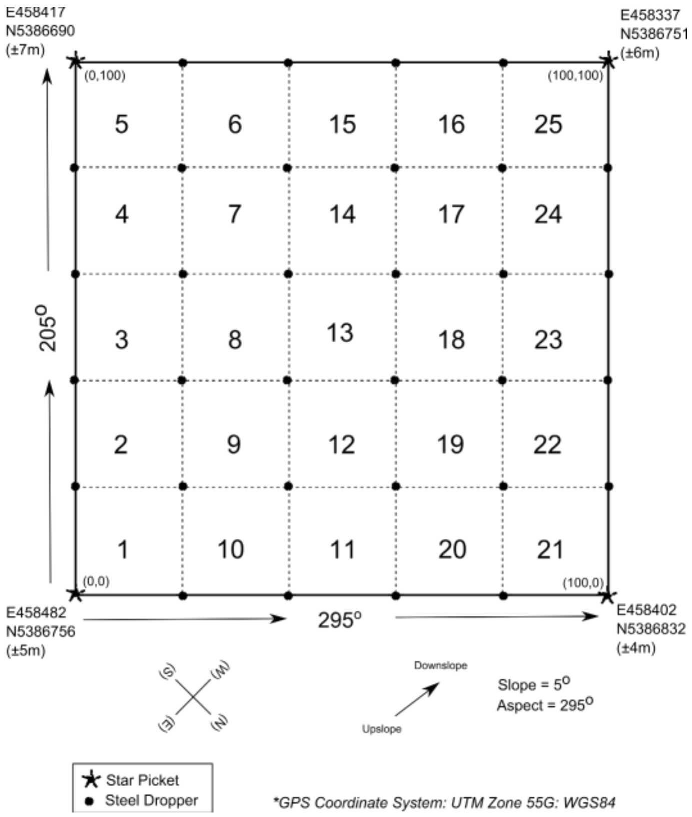

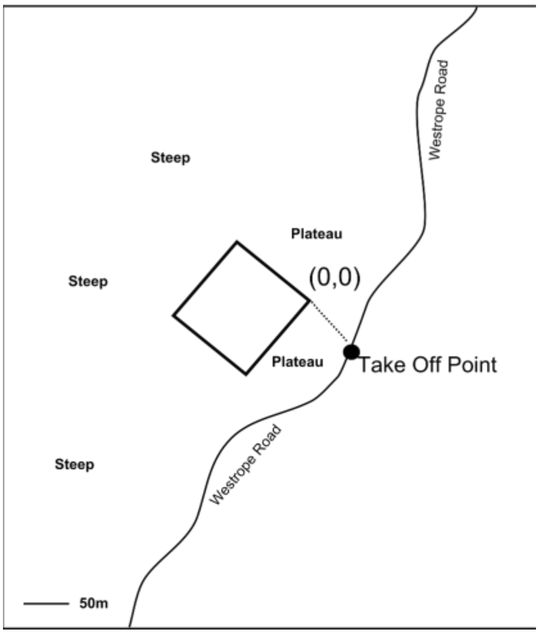

TCFTNS004: Caveside

**Target Eucalypt Species:**	*Eucalyptus delegatensis*	**High severity fire?**	Unknown
**Maximum Tree Height (m)**	40m	**Low severity fire?**	No
**Target Species Growth Stage:**	Mature	**Cut stumps?**	Yes, 13 Stumps, Axe Cut (>100cm DBH)
**Understorey:**	Rainforest/Sclerophyll	**Other Disturbance?**	Not observed

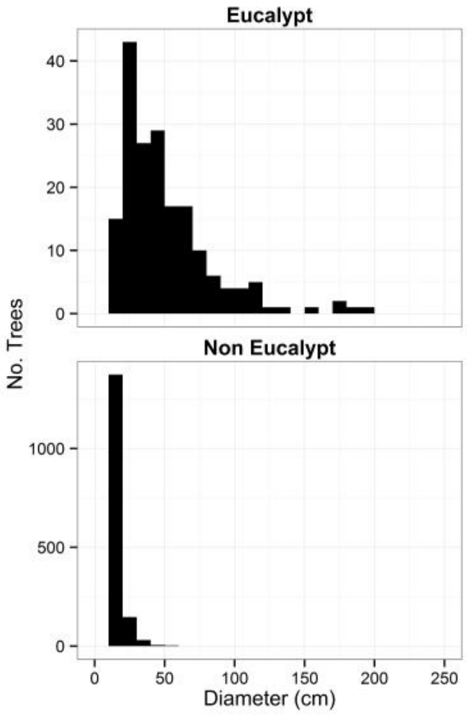

Species	No. Stems	BA (m^2/ha)
Eucalyptus delegatensis	107	35.5
Eucalyptus obliqua	77	17.7
Pomaderris apetala	889	14.2
Nothofagus cunninghamii	268	9.2
Bedfordia salicina	320	4.9
Acacia dealbata	22	2.3
Olearia argophylla	47	0.6
Leptospermum lanigerum	10	0.3
Atherosperma moschatum	2	<0.1
Phyllocladus aspleniifolius	2	<0.1
Pittosporum bicolor	1	<0.1

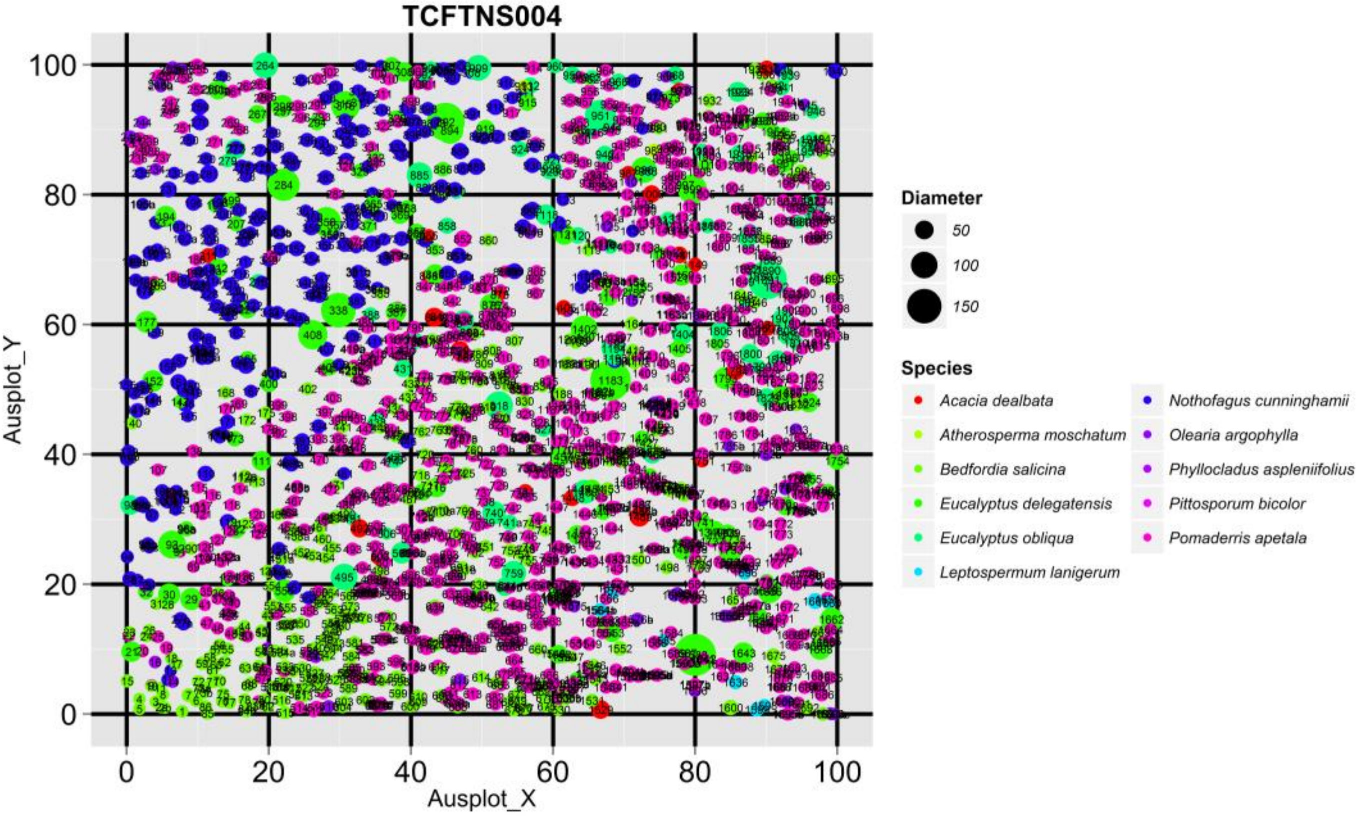

Supplement: S2 Text — Location, configuration, stand attributes and baseline data for 48 forest plots (115 pages). (PDF) [file pone.0137811.s002.pdf]
