# Supplementary material for: Macroecology of Australian Tall Eucalypt Forests: Baseline Data from a Continental-Scale Permanent Plot Network
Source: PLoS One. 2015 Sep 14;10(9):e0137811. doi: 10.1371/journal.pone.0137811 (PMC4569531; doi:10.1371/journal.pone.0137811)
Supplement: S3 Text — Description of methodology and analysis undertaken to classify the Prior et al. (2011) Permanent Growth Plot network into three height classes based on mean annual temperature and mean annual precipitation. (PDF) [file pone.0137811.s003.pdf]

S3 Text: Predicting eucalypt height as a function of climate for the Prior et al. (2011) Permanent Growth Plot Network.

The Permanent Growth Plot Network was compiled by Prior et al. (2011) to analyse eucalypt tree growth in relation to climate across Australia (Bowman and Prior 2014, Prior and Bowman 2014). It consists of growth data from permanent plots established in temperate eucalpyt forests by Australian State Government forestry organisations. The PGP dataset comprises repeated measurements of DBH (diameter at breast height) of tagged trees within 2409 plots of known area and location. Tree height was also measured for a subset of trees.

We generated a height-climate relationship for temperate eucalypt forests using the subset of the Permanent Growth Plot network that contained height information. We predicted maximum height as a function of mean annual temperature (MAT) and mean annual preciptiation (MAP) using a generalised additve model using the mgcv package in R (Figure S3). MAT and MAP was obtained for each pemanent growth plot from the WORLDCLIM dataset (Hijmans et al. 2005). Maximum height was the height of the tallest tree measured in each of the plots. To filter out young, regrowing forests, we only considered plots containing trees with a minimum diameter of 40 cm. This model is shown in Figure S3 and explained 27.3% of the deviance in the data.

We then partitioned the Permenant Growth Plot Network into three height classes (25-35 m, 35-45 m, >45 m) to contextualise where the Ausplots Forest Monitoring Network fits within the wider eucalypt forest estate. To do this, we used the modelled relationship to predict maximum height for all of the permanent growth plots using site specific MAT and MAP. We then categorised each plot as 25-35 m, 35-45 m and >45 m based on predicted maximum height.

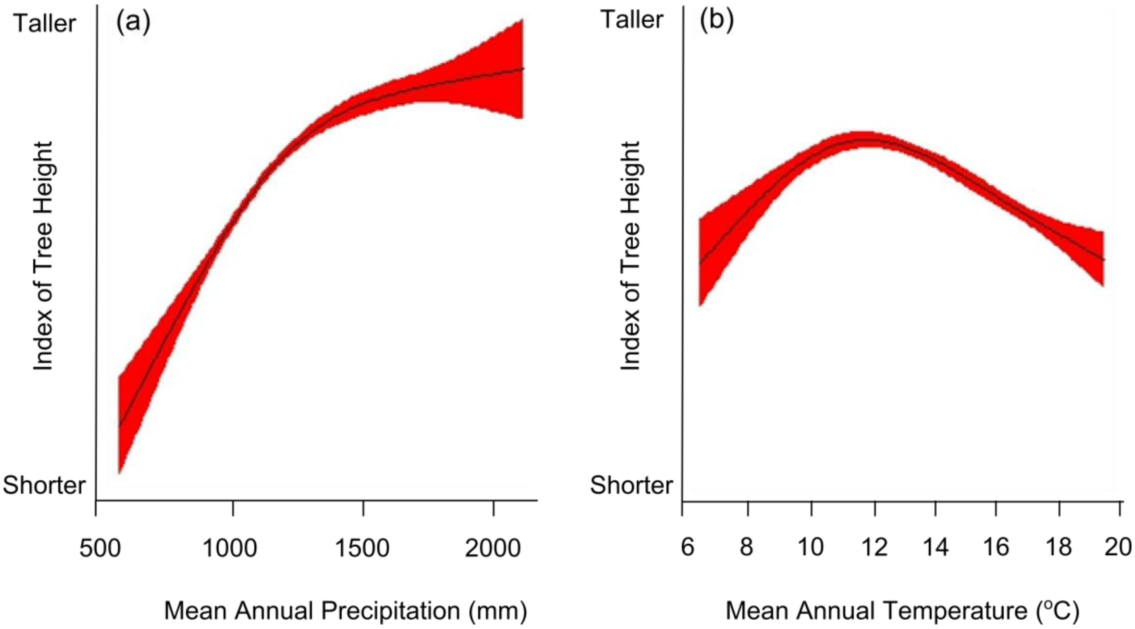

Figure S3: Index of maximum height as a function of (a) mean annual precipitation and (b) mean annual temperature (*temp*).

References

Prior LD, Williamson G, & Bowman DM (2011) Using permanent forestry plots to understand the possible effects of climate change on Australia's production forest estate. Department of Agriculture, Fisheries and Forestry, Canberra, December 2011

Bowman DM, Williamson GJ, Keenan RJ, Prior LD (2014) A warmer world will reduce tree growth in evergreen broadleaf forests: evidence from Australian temperate and subtropical eucalypt forests. Glob Ecol Biogeogr 23: 925–934

Prior LD, Bowman DM (2014) Big eucalypts grow more slowly in a warm climate: evidence of an interaction between tree size and temperature. Glob Chang Biol 20: 2793–2799
