# Supplementary material for: Macroecology of Australian Tall Eucalypt Forests: Baseline Data from a Continental-Scale Permanent Plot Network
Source: PLoS One. 2015 Sep 14;10(9):e0137811. doi: 10.1371/journal.pone.0137811 (PMC4569531; doi:10.1371/journal.pone.0137811)
Supplement: S4 Text — (PDF) [file pone.0137811.s004.pdf]

S4 Text: Scientific Research Permits issued during the establishment of the Ausplots Forest Monitoring Network 2012-2015.

State	Land Management Agency	Permit Type	Permit Number	Date of Issue
Western Australia	Department of Parks and Wildlife: Science and Conservation Division	Research conducted under permit issued under Dr Lachie McCaw, Principal Research Scientist and Program Leader of Ecosystem Science	Permit held by Dr Lachie McCaw, Principal Research Scientist.	August 2012
Tasmania	Forestry Tasmania	State Forest Activity Permit	FT62552	March 2013
Tasmania	Forestry Tasmania	State Forest Activity Permit	FT62552	April 2013
Tasmania	Forestry Tasmania	State Forest Activity Permit	FT62552	May 2013
Tasmania	Forestry Tasmania	State Forest Activity Permit	FT62552 (#1345)	September 2014
Tasmania	Forestry Tasmania	State Forest Activity Permit	FT62552 (#1345)	October 2014
Tasmania	Department of Primary Industries, Parks, Water and Environment	Permit for Taking of Native Flora	FL14221	September 2014
New South Wales	Office of Environment and Heritage, NSW National Parks and Wildlife Service	Scientific Licence	SL101219	September 2013
New South Wales	Forestry Corporation NSW	Special Purposes Permit for Research	SPPR0030	September 2013
Victoria	VicForests	Data Access Licence Agreement	AG4139-14	March 2014
Victoria	Department of Environment and Primary Industries	Research Permit/Permit to take Protected Flora	10007035	March 2014
Queensland	Department of National Parks, Recreation, Sport and Racing	Permit to Collect Biological or Geological Material	WIF414784214	July 2014
Queensland	Department of National Parks, Recreation, Sport and Racing	Take, Use, Keep or Interfere with Cultural or Natural Resources (Scientific Purpose)	WITK14639714	July 2014
