# Supplementary material for: Macroecology of Australian Tall Eucalypt Forests: Baseline Data from a Continental-Scale Permanent Plot Network
Source: PLoS One. 2015 Sep 14;10(9):e0137811. doi: 10.1371/journal.pone.0137811 (PMC4569531; doi:10.1371/journal.pone.0137811)
Supplement: S1 Table — The means (and range) of climatic variables and elevation are presented. MAT = Mean Annual Temperature. MAP = Mean Annual Precipitation. (PDF) [file pone.0137811.s005.pdf]

S1 Table: Details and environmental parameters of 48 plots in the Ausplots Forest Monitoring Network. The means (and range) of climatic variables and elevation are presented. MAT = Mean Annual Temperature. MAP = Mean Annual Precipitation.

AusPlot ID (Biome[+])	Plot Name	Tenure[#]	Location (Lat/Long)	Census Date	Plot size (ha)	Target Species	Growth Stage[&]	Existing Plot[%]	Climate Class[$]	Elevation (m)	MAT (°C)	MAP (mm)
**Northern NSW**												
NSFNNC001	Mines Rd	FR	-31.280 152.536	Sep 2013	1.0	*E. pilularis*	Mature	FL	Warm,wet	535	15.4	1434
NSFNNC002	A-Tree	NP	-31.242 152.460	Sep 2013	1.0	*E. pilularis*	Mature	FL	Warm,wet	631	15.3	1375
NSFNNC003	Tinebank	NP	-31.208 152.526	Oct 2013	1.0	*E. pilularis*	Mature	FL	Warm,wet	600	16.2	1362
NSFNNC004	Lorne	NP	-31.582 152.616	Oct 2013	1.0	*E. pilularis*	Mature	FL	Warm,wet	283	16.5	1457
NSFNNC005	Bird Tree	NP	-31.685 152.682	Nov 2013	1.0	*E. pilularis*	Mature	-	Warm,wet	352	16.1	1542
NSFNNC006	Black Bull	NP	-30.157 152.727	Nov 2013	1.0	*E. pilularis*	Mature	FL	Warm,wet	683	15.5	1895
NSFNNC007	Bruxner	FR	-30.240 153.091	Nov 2013	1.0	*E. grandis*	Mature	FL	Warm,wet	188	18.0	1872
NSFNNC008	OSullivans	NP	-32.345 152.260	Nov 2013	1.0	*E. grandis*	Mature	-	Warm,wet	75	17.6	1323
**Southern NSW**												
NSFSEC001	Newline	NP	-36.759 149.435	May 2014	1.0	*E. fastigata*	Mature	-	Cool/Wet	955	10.0	853
NSFSEC002	Waratah	NP	-36.996 149.382	May 2014	1.0	*E. fastigata*	Mature	FL,FA	Cool/Wet	828	10.4	917
NSFSEC003	Wog Way	NP	-37.014 149.380	May 2014	1.0	*E. fastigata*	Mature	-	Cool/Wet	845	10.9	911
NSFSEC004	Goodenia	NP	-36.903 149.717	May 2014	1.0	*E. fastigata*	Mature	-	Cool/Wet	420	13.1	1000
NSFSEC005	Candelo	NP	-36.863 149.594	May 2014	1.0	*E. obliqua*	Mature	-	Cool/Wet	645	11.9	953
**Victoria**												
VCFSEH001	Toolangi	RR	-37.530 145.516	Feb 2014	1.0	*E. regnans*	1939	FL,FA	Cool/Wet	337	11.2	1502
VCFSEH002	Ada	RR	-37.804 145.867	Feb 2014	1.0	*E. regnans*	1939	FL,FA	Cool/Wet	784	10.3	1714
VCFSEH003	Weeaproinah	NP	-38.643 143.474	Mar 2014	1.0	*E. regnans*	1939	N	Cool/Wet	471	11.2	1869
VCFSEH004	Turtons	NP	-38.639 143.701	Mar 2014	1.0	*E. regnans*	1898	N	Cool/Wet	480	11.1	1654
VCFSEH005	Lardners	NP	-38.623 143.629	Mar 2014	1.0	*E. regnans*	1939	N	Cool/Wet	531	11.3	1701
VCFSEH006	Black Spur	NP	-37.593 145.626	Apr 2014	1.0	*E. regnans*	1939	FL,FA	Cool/Wet	580	11.7	1445
VCFSEH007	Monda Rd	NP	-37.589 145.639	Apr 2014	1.0	*E. regnans*	1939	FL,FA	Cool/Wet	579	11.6	1468
VCFSEH008	Hardy Creek	FR	-37.569 145.546	Apr 2014	1.0	*E. regnans*	1939	IN (1970)	Cool/Wet	863	10.5	1641
**Far North Queensland**												
QDWET001	Mt Baldy	FR	-17.269 145.418	Aug 2014	1.0	*E. grandis*	Mature	FL	Hot,seasonal	1058	19.4	1726
QDWET002	Longlands	NP	-17.415 145.464	Aug 2014	1.0	*E. grandis*	Mature	-	Hot,seasonal	1048	19.4	1376
QDWET003	Lamb Range	NP	-17.110 145.560	Aug 2014	1.0	*E. grandis*	Mature	FL	Hot,seasonal	1148	18.9	1601
QDWET004	Koombaloomba	NP	-17.841 145.584	Aug 2014	1.0	*E. grandis*	Mature	-	Hot,seasonal	795	20.5	1732
**Western Australia**												
WAFWAR001	Carey	NP	-34.386 115.845	Sep 2012	1.0	*E. diversicolor*	1852	IN (1982)	Warm,seasonal	164	15.3	1098
WAFWAR002	Dombakup	FR	-34.576 115.982	Sep 2012	1.0	*E. diversicolor*	1857	IN (1981)	Warm,seasonal	93	15.2	1163
WAFWAR003	Warren	NP	-34.545 115.952	Sep 2012	1.0	*E. diversicolor*	1854	IN (1981)	Warm,seasonal	134	14.8	1175
WAFWAR004	Dawson	FR	-34.849 116.687	Sep 2012	1.0	*E. diversicolor*	1937	IN (1982)	Warm,seasonal	148	15.2	1105
WAFWAR005	Giants	NP	-34.980 116.879	Oct 2012	1.0	*E. jacksonii*	1937/OG	IN (1964)	Warm,seasonal	158	15.1	1132
WAFWAR006	Sutton	NP	-34.448 116.249	Nov 2012	1.0	*E. diversicolor*	1917	IN (1981)	Warm,seasonal	142	15.0	1006
WAFWAR007	Mt Frankland	NP	-34.824 116.873	Nov 2012	1.0	*E. diversicolor*	Mature	N	Warm,seasonal	239	14.8	1026
WAFWAR008	Mt Clare	NP	-34.995 116.650	Nov 2012	1.0	*E. jacksonii*	OG	N	Warm,seasonal	165	15.0	1204
WAFWA009	Collins	FR	-34.507 116.124	Nov 2012	1.0	*E. diversicolor*	Mature	IN (1964)	Warm,seasonal	136	14.9	1120
**Low Elevation Tasmania**												
TCFTSR001	Bird Track	WHA	-43.089 146.643	Jan 2012	1.0	*E. obliqua*	1934	IN (2007)	Cool/Wet	212	10.3	1466
TCFTSR002	Supersite	WHA	-43.095 146.653	Apr 2012	1.6	*E. obliqua*	1898	FLUX	Cool/Wet	111	11.2	1364
TCFTSR003	Nth Styx	FR	-42.811 146.608	Apr 2013	1.0	*E. regnans*	1934	N	Cool/Wet	560	9.7	1299
TCFTSR004	Weld R.	FR	-43.041 146.743	Apr 2013	1.0	*E. regnans*	1934	N	Cool/Wet	87	11.0	1228
TCFTSR005	Arve V.	FR	-43.102 146.747	May 2013	1.0	*E. obliqua*	1898	IN (2007)	Cool/Wet	284	10.2	1381
TCFKIN001	BlackRiver	FR	-40.903 145.285	Oct 2014	1.0	*E. obliqua*	1920's	IN (1972)	Cool/Wet	49	12.4	1139
TCFKIN002	BondTier	FR	-40.952 144.842	Oct 2014	1.0	*E. obliqua*	1920's	Y	Cool/Wet	54	12.3	1275
TCFTNS001	Flowerdale	FR	-41.044 145.566	Mar 2012	1.0	*E. obliqua*	1920's	IN (1970)	Cool/Wet	206	11.4	1406
TCFTNS002	Dip River	FR	-41.034 145.405	Apr 2012	1.0	*E. obliqua*	1920's	IN (1976)	Cool/Wet	247	11.2	1477
**High Elevation Tasmania**												
TCFTNS003	McKenzie	TFA	-41.629 146.258	Jan 2015	1.0	*E. delegatensis*	Mature	N	Cold	790	7.5	1723
TCFTNS004	Caveside	TFA	-41.669 146.501	Feb 2015	1.0	*E. delegatensis*	Mature	N	Cold	691	7.4	1368
TCFBEL001	Mt Maurice	TFA	-41.311 147.538	Dec 2014	1.0	*E. delegatensis*	Mature	N	Cold	752	8.7	1371
TCFBEL002	Ben Ridge	TFA	-41.367 147.602	Jan 2015	1.0	*E. delegatensis*	Mature	N	Cold	910	8.6	1350
TCFTSR006	Mt Field	WHA	-42.682 146.649	Dec 2014	1.0	*E. delegatensis*	Mature	N	Cold	843	6.6	1309

Foototes to S1 Table

[+]***:*** *IBRA Bioregion denoted by letters 4-6 of Plot ID: BEL = Ben Lomond; KIN = King; NNC = NSW North Coast; SEC = South East Corner; SEH = South East Highlands; TNS = Tasmanian North Slopes; TSR = Tasmanian Southern Ranges; WAR = Warren; WET = Wet Tropics.*

[#]***:*** *Tenure: NP= National Park; FR = Forest Reserve; WHA=World Heritage Area; TFA = Tasmanian Forest Agreement Future Reserve; MUF = Multiple Use Forest**.***

[%]***:*** *FL=Flora Survey; FA = Fauna Survey; IN = Inventory (first year of measurement); FLUX = Flux Tower.*

[&]*: OG = Old Growth; Mature = Mature; Date = known stand establishment date.*

[$]***:*** *Hutchinson Agro-Climate Classifications and their relationship to the global Koppen Climate Classification scheme are described in S6 Table*
