# Supplementary material for: Macroecology of Australian Tall Eucalypt Forests: Baseline Data from a Continental-Scale Permanent Plot Network
Source: PLoS One. 2015 Sep 14;10(9):e0137811. doi: 10.1371/journal.pone.0137811 (PMC4569531; doi:10.1371/journal.pone.0137811)
Supplement: S2 Table — The means (and range) are presented for eucalypt (including Eucalyptus spp. and Corymbia spp.) and non-eucalypt species. (PDF) [file pone.0137811.s006.pdf]

S2 Table: Stand structural attributes of 48 plots in the Ausplots Forest Monitoring Network. The means (and range) are presented for eucalypt (including *Eucalyptus* spp. and *Corymbia* spp.) and non-eucalypt species.

AusPlot ID	Plot Name	Canopy Cover (%)	Mean Height Dominants (m)	Maximum Height (m)		Stocking density (stems/ha)		Basal Area (m^2/ha)		Above Ground Live Carbon (tC ha^{-1})	
		All Trees	Eucalypt	Eucalypt	Non-Euc	Eucalypt	Non-Euc	Eucalypt	Non-Euc	Eucalypt	Non-Euc
**Northern NSW**											
NSFNNC001	Mines Rd	58	45	57	34	101	326	44.4	10.7	277	34
NSFNNC002	A-Tree	48	51	66	24	228	230	51.9	6.8	267	20
NSFNNC003	Tinebank	77	57	74	44	86	434	46.1	16.7	281	58
NSFNNC004	Lorne	74	57	75	29	184	229	48.8	6.3	282	20
NSFNNC005	Bird Tree	79	59	77	27	134	494	68.0	9.0	399	26
NSFNNC006	Black Bull	72	49	70	38	166	227	39.4	7.7	213	24
NSFNNC007	Bruxner	80	51	65	19	258	627	38.2	13.2	169	39
NSFNNC008	OSullivans	76	54	72	29	336	243	43.8	5.3	194	15
**Southern NSW**											
NSFSEC001	Newline	63	40	50	8	318	2	64.2	<0.1	318	<1
NSFSEC002	Waratah	71	40	49	-	273	0	63.8	<0.1	345	<1
NSFSEC003	Wog Way	63	48	59	8	210	15	61.8	0.7	345	<1
NSFSEC004	Goodenia	56	50	64	21	121	40	50.4	0.8	354	3
NSFSEC005	Candelo	62	47	58	-	151	4	57.5	<0.1	279	2
**Victoria**											
VCFSEH001	Toolangi	68	60	72	31	122	132	59.5	2.2	318	6
VCFSEH002	Ada	82	66	82	30	177	250	49.1	7.4	232	22
VCFSEH003	Weeaproinah	74	60	77	42	246	573	56.1	20.2	253	63
VCFSEH004	Turtons	80	74	88	52	80	427	56.7	22.4	334	80
VCFSEH005	Lardners	84	65	83	40	242	457	66.1	15.9	351	52
VCFSEH006	Black Spur	75	68	78	-	117	6	49.9	0.1	257	0
VCFSEH007	Monda Rd	73	68	83	22	150	212	58.1	4.4	296	12
VCFSEH008	Hardy Creek	81	69	89	32	147	69	54.3	2.7	270	8
**Far North Queensland**											
QDWET001	Mt Baldy	59	41	56	46	100	102	22.2	5.0	109	18
QDWET002	Longlands	55	28	40	17	199	42	16.9	0.6	77	2
QDWET003	Lamb Range	71	38	48	28	102	177	23.1	14.6	113	53
QDWET004	Koombaloomba	78	37	47	21	119	280	32.0	4.6	170	12
**Western Australia**											
WAFWAR001	Carey	67	65	76	15	74	45	52.0	0.5	323	1
WAFWAR002	Dombakup	66	59	69	23	84	218	51.1	11.9	304	43
WAFWAR003	Warren	66	57	65	15	106	43	61.3	0.5	380	1
WAFWAR004	Dawson	62	46	55	21	85	87	28.9	5.6	155	19
WAFWAR005	Giants	64	40	53	21	169	102	*	7.6	*	*
WAFWAR006	Sutton	65	45	56	16	226	27	41.7	0.3	204	1
WAFWAR007	Mt Frankland	82	50	66	22	169	26	46.4	1.7	284	6
WAFWAR008	Mt Clare	77	50	62	28	47	64	*	14.3	*	*
WAFWA009	Collins	75	57	77	16	87	30	38.6	1.2	241	4
**Low Elevation Tasmania**											
TCFTSR001	Bird Track	84	52	59	33	150	395	58.1	13.9	339	44
TCFTSR002	Supersite	-	-	-	-	79	170	56.3	16.3	384	61
TCFTSR003	Nth Styx	71	39	58	41	310	206	68.2	8.6	400	31
TCFTSR004	Weld R.	-	44	60	34	108	198	24.0	16.3	113	60
TCFTSR005	Arve V.	75	49	66	24	249	787	70.5	13.3	386	35
TCFKIN001	BlackRiver	75	46	57	-	209	356	42.5	9.0	186	27
TCFKIN002	BondTier	83	46	64	39	80	504	50.0	21.6	317	81
TCFTNS001	Flowerdale	-	50	59	31	176	229	59.4	7.7	330	25
TCFTNS002	Dip River	-	54	66	49	60	698	32.6	24.7	194	82
**High Elevation Tasmania**											
TCFTNS003	McKenzie	72	43	51	37	244	305	47.6	15.0	232	52
TCFTNS004	Caveside	-	33	40	25	184	1561	53.2	31.6	299	90
TCFBEL001	Mt Maurice	74	42	51	-	381	87	60.3	2.1	285	6
TCFBEL002	Ben Ridge	72	38	47	30	330	260	58.1	8.3	310	27
TCFTSR006	Mt Field	73	42	52	23	309	383	85.2	14.0	426	44

Notes to S2 table

** BA and AGC was not calculated for two plots in Western Australia (WAFWAR005 and WAFWAR008) because of the effect of extremely prominent buttressing of Eucalyptus jacksonii trees on calculations of AGC derived from diameter measurements.*
