# Supplementary material for: Macroecology of Australian Tall Eucalypt Forests: Baseline Data from a Continental-Scale Permanent Plot Network
Source: PLoS One. 2015 Sep 14;10(9):e0137811. doi: 10.1371/journal.pone.0137811 (PMC4569531; doi:10.1371/journal.pone.0137811)
Supplement: S3 Table — Only those classificatons relevant to the Ausplots Forests Monitoring Network are shown. (PDF) [file pone.0137811.s007.pdf]

S3 Table: The Hutchinson agro-climatic classification and its relationship to the global Koppen-Geiger climate classification. Only those classifications relevant to the Ausplots Forests Monitoring Network are shown.

Code	Name	Description
B	Cold	Very cold winters with short, warm summers that are wet enough to support significant growth. Includes Köppen class D (snow climates) and cooler portions of Köppen class Cf (temperate with sufficient precipitation in all months).
D	Cool,wet	Cold, growth-limiting winter with sufficient moisture for crop growth in some non-winter months, mostly occurring in mid-latitude locations with maritime influence. Includes parts of Köppen classes Cf, Cw, Df and Dw (temperate and snow climates with sufficient moisture all year or with a winter dry season).
E	Warm,<br>seasonally wet/dry	Long, hot summers and mild winters, with significant moisture limits on growth. These include the Mediterranean and adjacent inland climates (where the dry season is in summer) and mid-latitude eastern continental climates with wetter summers and drier winters. Includes parts of Köppen classes BS, BW, Cw and Cs (dry and temperate climates with a dry summer or winter).
F	Warm,wet	Long, hot summers and mild winters with a moisture regime that is non-limiting for most of the year. These climates are mainly associated with mid-latitude eastern maritime locations and some tropical high latitude locations. Includes parts of Köppen classes Cf, Cw and Aw (temperate and tropical climates with a summer or winter dry season).
I	Hot,<br>sesonally wet/dry	High temperature for most of the year and a markedly seasonal moisture regime. Includes Köppen classes Aw and parts of Bs and Cw (tropical and temperate climates with a summer or winter dry season).

References

Hutchinson MF, McIntyre S, Hobbs RJ, Stein JL, Garnett S, Kinloch J (2005) Integrating a global agro-climatic classification with bioregional boundaries in Australia. Glob Ecol Biogeog 14: 197-212.
