# Supplementary material for: Macroecology of Australian Tall Eucalypt Forests: Baseline Data from a Continental-Scale Permanent Plot Network
Source: PLoS One. 2015 Sep 14;10(9):e0137811. doi: 10.1371/journal.pone.0137811 (PMC4569531; doi:10.1371/journal.pone.0137811)
Supplement: S4 Table — Euc = Eucalypt, Scl = Wet Sclerophyll, RF = Rainforest. Unidentified species are prefixed with UNN and have been vouchered for identification by local herbaria. (PDF) [file pone.0137811.s008.pdf]

S4 Table: List of species censused in the Ausplots Forest Monitoring Network and their respective community guild. Euc = Eucalypt, Scl = Wet Sclerophyll, RF = Rainforest. Unidentified species are prefixed with UNN and have been vouchered for identification by local herbaria.

Species	Guild	Species	Guild	Species	Guild
Acacia cincinnata	Scl	*Corymbia calophylla*	Euc	*Eupomatia laurina*	RF
Acacia dealbata	Scl	*Corymbia intermedia*	Euc	*Euroschinus falcata*	RF
Acacia maidenii	Scl	*Cryptocarya bidwillii*	RF	*Flindersia brayleyana*	RF
Acacia melanoxylon	Scl	*Cryptocarya glaucescens*	RF	*Geijera salicifolia*	RF
Acacia mucronata	Scl	*Cryptocarya microneura*	RF	*Geissois benthamii*	RF
Acacia pentadenia	Scl	*Cryptocarya rigida*	RF	*Glochidion harveyanum*	RF
Acacia schinoides	Scl	*Cupaniopsis foveolata*	RF	*Glochidion hylandii*	RF
Acacia selsa	Scl	*Darlingia darlingiana*	RF	*Glochidion sp.*	RF
Acacia verticillata	Scl	*Dendrocnide photinophylla*	RF	*Gmelina fasciculiflora*	RF
Acmena smithii	RF	*Doryphora sassafras*	RF	*Guioa acutifolia*	RF
Acronychia acronychiodes	RF	*Elaeocarpus holopetalus*	RF	*Guioa lacioneura*	RF
Agonis flexuosa	Scl	*Elaeocarpus reticulatus*	RF	*Guioa semiglauca*	RF
Allocasuarina decussata	Scl	*Endiandra discolor*	RF	*Hakea lissosperma*	Scl
Allocasuarina torulosa	Scl	*Endiandra hypotephra*	RF	*Hedycarya angustifolia*	RF
Alphitonia excelsa	RF	*Endiandra sieberi*	RF	*Hedycarya loxocarya*	RF
Alphitonia petriei	RF	*Eucalyptus andrewsii*	Euc	*Leptospermum lanigerum*	Scl
Alstonia muelleriana	RF	*Eucalyptus coccifera*	Euc	*Leptospermum scoparium*	Scl
Alstonia scholaris	RF	*Eucalyptus cypellocarpa*	Euc	*Leptospermum spp*	Scl
Anopterus glandulosus	RF	*Eucalyptus dalrympleana*	Euc	*Litsea australis*	RF
Archihodomyrtus beckleri	RF	*Eucalyptus delegatensis*	Euc	*Litsea connorsii*	RF
Archontophoenix cunninghamiana	RF	*Eucalyptus diversicolor*	Euc	*Litsea leefeana*	RF
Atherosperma moschatum	RF	*Eucalyptus fastigata*	Euc	*Litsea reticulata*	RF
Banksia	Scl	*Eucalyptus grandis*	Euc	*Lomatia fraseri*	Scl
Banksia aquilonia	Scl	*Eucalyptus guilfoylei*	Euc	*Lomatia fraxinifolia*	RF
Bedfordia arborescens	Scl	*Eucalyptus jacksonii*	Euc	*Lomatia myricoides*	Scl
Bedfordia salicina	Scl	*Eucalyptus microcorys*	Euc	*Lophostemon sp.*	RF
Caldcluvia paniculosa	RF	*Eucalyptus nitens*	Euc	*Meleleuca ericifolia*	Scl
Callicoma serratifolia	RF	*Eucalyptus obliqua*	Euc	*Melicope hayesii*	RF
Callistemon salignus	Scl	*Eucalyptus ovata*	Euc	*Melicope sp.*	RF
Callitris sp.	Scl	*Eucalyptus pilularis*	Euc	*Mischocarpus lachnocarpus*	RF
Casearia sp.	RF	*Eucalyptus radiata*	Euc	*Monotoca glauca*	Scl
Ceratopetalum apetalum	RF	*Eucalyptus regnans*	Euc	*Nematolepis squamea*	Scl
Cissus hypoglauca	RF	*Eucalyptus resinifera*	Euc	*Neolitsea dealbata*	RF
Clerodendrum floribundum	RF	*Eucalyptus saligna*	Euc	*Niemeyeria whitei*	RF
Commersonia bartramia	RF	*Eucalyptus sieberi*	Euc	*Nothofagus cunninghamii*	RF
Coprosma nitida	Scl	*Eucalyptus sp.*	Euc	*Olearia argophylla*	Scl
Coprosma quadrifida	Scl	*Eucalyptus subcrenulata*	Euc	*Orites excelsus*	RF
Coprosma spp	Scl	*Eucalyptus urnigera*	Euc	*Ozothamnus antennaria*	Scl
Cordia dichotoma	RF	*Eucalyptus viminalis*	Euc	*Persoonia arborea*	Scl
Correa lawrenceana	Scl	*Eucryphia lucida*	RF	*Persoonia conjuncta*	Scl

S4 Table continued: List of species censused in the Ausplots Forest Monitoring Network and their community guild. Euc = Eucalypt, Scl = Wet Sclerophyll, RF = Rainforest. Unidentified species are prefixed with UNN and have been vouchered for identification by local herbaria.

Species	Guild	Species	Guild
Persoonia muelleri	Scl	*UNNCEUC1*	Euc
Persoonia silvatica	Scl	*UNNCEUC3*	Euc
Phyllocladus aspleniifolius	RF	*UNNCEUC4*	Euc
Pilidiostigma glabrum	RF	*UNNCG10*	RF
Pittosporum bicolor	Scl	*UNNCG15*	RF
Pittosporum undulatum	RF	*UNNCG16*	RF
Polyscias elegans	RF	*UNNCG18*	RF
Polyscias sp	RF	*UNNCG2*	RF
Pomaderris apetala	Scl	*UNNCG20*	RF
Pomaderris aspera	Scl	*UNNCG21*	RF
Prostanthera lasianthos	Scl	*UNNCG22*	RF
Rhodamnia rubescens	RF	*UNNCG24*	RF
Rhodamnia sacilifolia	RF	*UNNCG26*	RF
Schizomeria ovata	RF	*UNNCG28*	RF
Schizomeria whitei	RF	*UNNCG30*	RF
Sloanea langii	RF	*UNNCG4*	RF
Stenocarpus sinuatus	RF	*UNNCG6*	RF
Symplocos cochinchiensis	RF	*UNNCG7*	RF
Syncarpia glomulifera	RF	*UNNCG9*	RF
Synoum glandulosum	RF	*UNNCMYRT2*	RF
Syzygium oleosum	RF	*UNNCPALM2*	RF
Tasmannia lanceolata	Scl	*UNNCU14*	RF
Toona australis	RF	*UNNCU15*	RF
Trochocarpa laurina	RF	*UNNCU18*	RF
Trymalium odoratissimum	Scl	*UNNCU19*	RF
Zieria arborescens	Scl	*UNNCU21*	RF
		UNNCU22	RF
		UNNCU23	RF
		UNNCU26	RF
		UNNCU29	RF
		UNNCU34	RF
		UNNCU38	RF
		UNNCU40	RF
		UNNCUnk	RF
		UNNCUSM	RF
		UVCFUS1	Scl
		UVCFUS2	Scl
		UVCFUS3	Scl
		UWETLive	RF
