# Supplementary material for: Macroecology of Australian Tall Eucalypt Forests: Baseline Data from a Continental-Scale Permanent Plot Network
Source: PLoS One. 2015 Sep 14;10(9):e0137811. doi: 10.1371/journal.pone.0137811 (PMC4569531; doi:10.1371/journal.pone.0137811)
Supplement: S5 Table — RDe = relative density, RF = relative frequency, RDo = relative dominance, IV = Importance Value. (PDF) [file pone.0137811.s009.pdf]

S5 Table: Importance Values for forest community guilds (Eucalypt, Rainforest, Wet Sclerophyll) for each region and for the Ausplots Forest Monitoring Network. RDe = relative density, RF = relative frequency, RDo = relative dominance, IV = Importance Value.

	No. Species	Rf	RDe	RDo	IV
**Far North Queensland**					
Eucalypt	4	35.7	46.4	79.2	161.3
Rainforest	39	26.8	15.6	4.0	46.4
Sclerophyll	6	35.7	37.9	16.8	90.5
**Northern NSW**					
Eucalypt	10	33.3	33.0	82.6	148.9
Rainforest	65	33.3	48.0	9.0	90.3
Sclerophyll	6	33.3	19.0	8.4	60.7
**Southern NSW**					
Eucalypt	7	45.5	94.6	99.5	239.5
Rainforest	3	18.2	1.1	0.1	19.3
Sclerophyll	7	36.4	4.3	0.5	41.1
**Low Elevation Tasmania**					
Eucalypt	3	35.7	28.5	77.8	142.0
Rainforest	5	27.8	13.3	4.6	45.7
Sclerophyll	15	35.7	58.2	17.6	111.5
**High Elevation Tasmania**					
Eucalypt	6	35.7	35.8	81.1	152.6
Rainforest	3	28.6	13.2	4.9	46.6
Sclerophyll	13	35.7	51.0	14.0	100.8
**Victoria**					
Eucalypt	3	38.5	35.7	85.6	159.8
Rainforest	1	24.0	6.3	0.6	31.0
Sclerophyll	18	38.5	58.0	13.7	110.2
**Western Australia**					
Eucalypt	4	50.0	62.0	91.8	203.8
Rainforest	0	0.0	0.0	0.0	0.0
Sclerophyll	5	50.0	38.0	8.2	96.2
**Total**					
Eucalypt	30	46.2	39.2	85.5	170.9
Rainforest	108	27.9	17.7	3.3	48.9
Sclerophyll	47	26.0	43.1	11.6	80.7
