# Supplementary material for: Macroecology of Australian Tall Eucalypt Forests: Baseline Data from a Continental-Scale Permanent Plot Network
Source: PLoS One. 2015 Sep 14;10(9):e0137811. doi: 10.1371/journal.pone.0137811 (PMC4569531; doi:10.1371/journal.pone.0137811)

S1 Fig: Distribution of live aboveground carbon (AGC; tC ha^{-1}) in *Eucalyptus* species across diameter classes in the Ausplots Forests Monitoring Network.

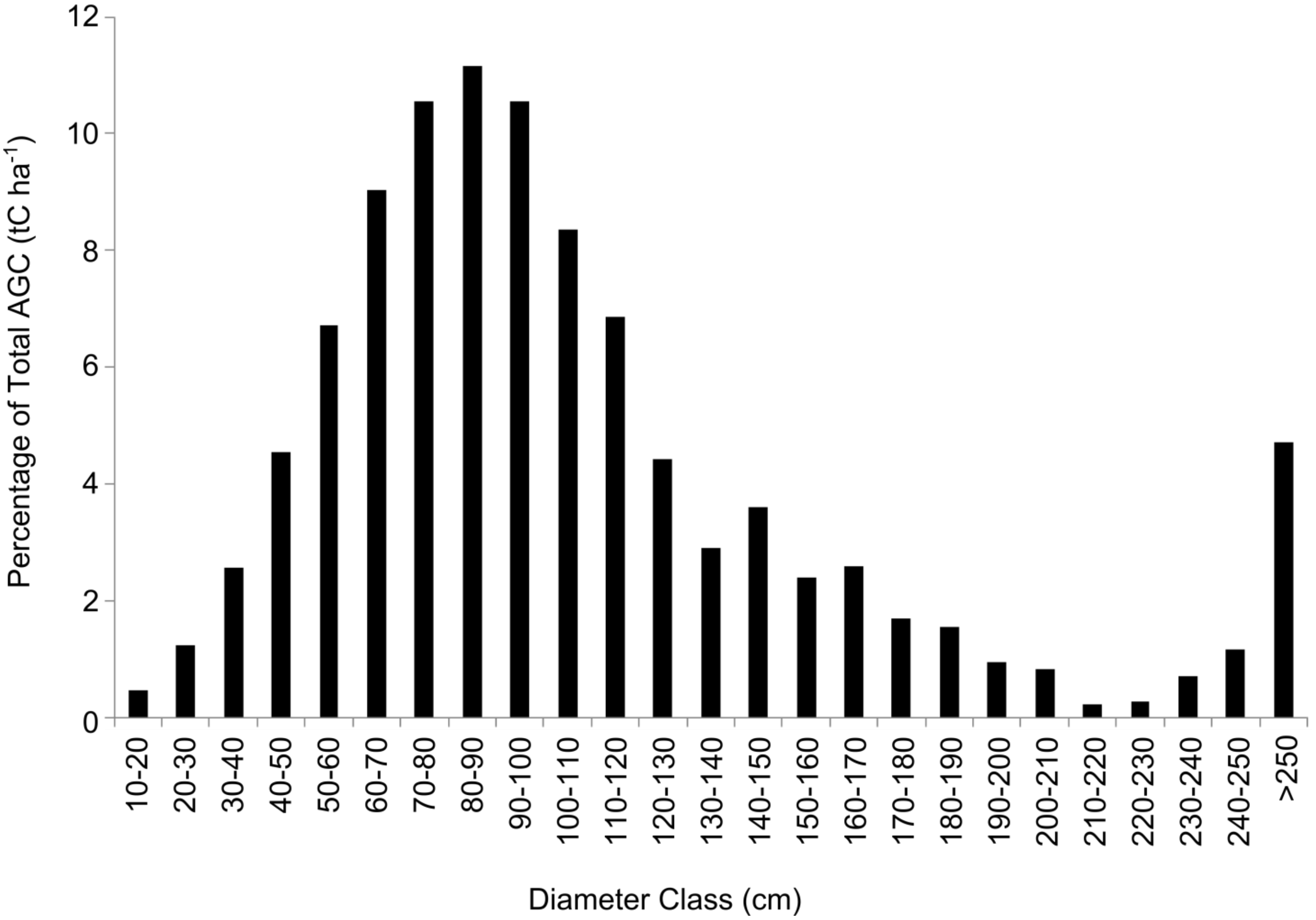

Supplement: S1 Fig — (PDF) [file pone.0137811.s010.pdf]
